# Diagnosis of Pancreatic Ductal Adenocarcinoma and Chronic Pancreatitis by Measurement of microRNA Abundance in Blood and Tissue

**DOI:** 10.1371/journal.pone.0034151

**Published:** 2012-04-12

**Authors:** Andrea S. Bauer, Andreas Keller, Eithne Costello, William Greenhalf, Melanie Bier, Anne Borries, Markus Beier, John Neoptolemos, Markus Büchler, Jens Werner, Nathalia Giese, Jörg D. Hoheisel

**Affiliations:** 1 Division of Functional Genome Analysis, Deutsches Krebsforschungszentrum, Heidelberg, Germany; 2 Biomarker Discovery Center Heidelberg, Heidelberg, Germany; 3 The National Institute for Health Research Pancreas Biomedical Research Unit and the Liverpool Experimental Cancer Medicine Center, Royal Liverpool University Hospital, Liverpool, United Kingdom; 4 Department of Surgery, University of Heidelberg, Heidelberg, Germany; Baylor University Medical Center, United States of America

## Abstract

A solid process for diagnosis could have a substantial impact on the successful treatment of pancreatic cancer, for which currently mortality is nearly identical to incidence. Variations in the abundance of all microRNA molecules from peripheral blood cells and pancreas tissues were analyzed on microarrays and in part validated by real-time PCR assays. In total, 245 samples from two clinical centers were studied that were obtained from patients with pancreatic ductal adenocarcinoma or chronic pancreatitis and from healthy donors. Utilizing the minimally invasive blood test, receiver operating characteristic (ROC) curves and the corresponding area under the curve (AUC) analysis demonstrated very high sensitivity and specificity of a distinction between healthy people and patients with either cancer or chronic pancreatitis; respective AUC values of 0.973 and 0.950 were obtained. Confirmative and partly even more discriminative diagnosis could be performed on tissue samples with AUC values of 1.0 and 0.937, respectively. In addition, discrimination between cancer and chronic pancreatitis was achieved (AUC = 0.875). Also, several miRNAs were identified that exhibited abundance variations in both tissue and blood samples. The results could have an immediate diagnostic value for the evaluation of tumor reoccurrence in patients, who have undergone curative surgical resection, and for people with a familial risk of pancreatic cancer.

## Introduction

Pancreatic cancer is one of the most aggressive and malignant tumor entities with a five-year survival rate of less than 5% [1]. Most patients die within a year of diagnosis. The poor prognosis is caused by the lack of both appropriate markers for early diagnosis and effective treatment options for the late stages that are consequently seen in clinics. Since mortality is nearly identical with incidence, pancreatic cancer is the fourth to fifth most common cause of cancer-related deaths in industrialized countries. Extensive studies have been performed in order to identify biomarkers for the disease. Some have been or are currently being evaluated, such as CEACAM-1, MIC-1, PAM4 and CA19-9; the last is the only blood-borne biomarker in routine clinical use for management of pancreatic cancer. At the level of messenger RNA (mRNA), quite a few, including some very specific molecular variations have been found in tissues [2]. However, none of these markers has proven helpful in facilitating diagnosis [3], largely due to the real difficulty in obtaining biopsy material and the dilemma that the performance of biopsies is inappropriate without prior indication of disease.

More recently, microRNAs (miRNAs) have gained attention as possible biomarkers. They belong to the group of small non-coding RNAs and have essential functions in various biological processes [4]. In addition, the molecules are stable in comparison to mRNA, which is of considerable importance for the robustness of diagnostic assays. About 1000 miRNAs are believed to occur in humans and are considered to form a distinct layer of regulation of cellular function. Several miRNAs were found to be associated with tumor-relevant processes [5]. Similar to mRNA profiling, miRNA signatures exhibited distinctive expression variations in pancreatic tumor samples, chronic pancreatitis tissue and normal pancreas [6], [7]. Expression abnormalities in pancreatic endocrine and acinar tumors were associated with distinctive pathologic features and clinical behavior [8]. Also, a relationship of the expression of particular miRNA and survival of patients with pancreatic adenocarcinoma was reported [9]. The transcripts miR-21, miR-155, miR-203, miR-210 and miR-222 were described as potential predictors of survival [10], [11]. However, the tissue-based miRNA studies also suffer from the fact that invasive action is required to acquire material for analysis, and as such, tissue-based miRNA profiling does not offer significant progress compared to messenger RNA profiling but for the superior stability of miRNA.

Markers that occur in peripheral blood or other body fluids would be best for detection. For various tumor entities, extracellular nucleic acids have been found in serum, for example [12]. In part, they have their origin in circulating tumor cells. Moreover, the actual tumor cells themselves could be isolated from blood and used as a means for diagnosis and prognosis [13]. Recently, accumulating data have become available which indicate that a diagnosis of different forms of disease, including cancers, may be possible by analyzing the miRNA levels in serum (e.g., [14], [15]). For pancreatic cancer, an analysis of the variations in plasma of four miRNAs has been reported [16]. In a slightly different approach, we studied variations of the miRNA levels in blood cells. In a multicenter study [17], combining 454 samples, we were recently able to discriminate between 13 disease conditions and healthy control individuals. In extension to this, we analyzed here the actual power of miRNA signatures in blood cells for diagnosis and compared them to signatures obtained from pancreatic tissue samples. Both the blood and tissue samples were taken from patients with pancreatic ductal adenocarcinoma, chronic pancreatitis or from healthy individuals. From the results, highly accurate molecular classifiers could be defined. In addition, the variations observed in blood and tissue were compared in order to detect possible functional connections between them. The blood analysis permitted distinction of healthy and diseased pancreas but did not yield sufficient information to separate pancreatic inflammation from cancer. The tissue analysis, however, showed differences between inflammation and tumor. In combination, this process may enable a differential and initially minimally invasive identification of disease, in particular for the evaluation of tumor reoccurrence in patients, who have undergone curative surgical resection, and for people with a familial risk of pancreatic cancer.

## Results

### miRNA profiles

**Figure 1:**
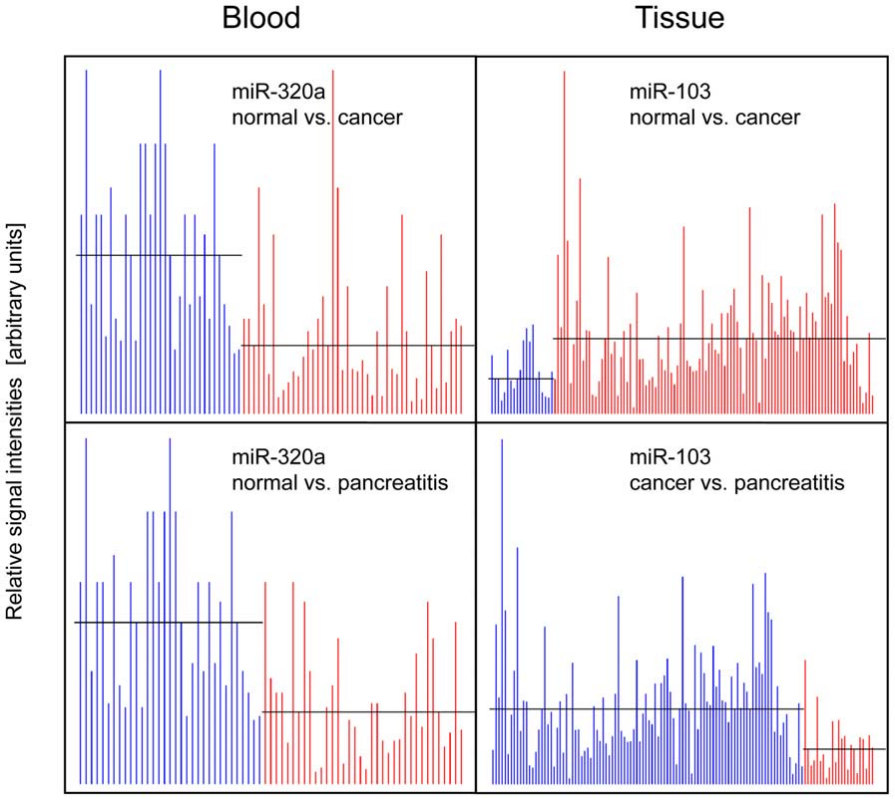
Barplots detailing intensity values recorded for particular miRNAs that exhibited significant expression variations. Only one typical result each is shown for blood and tissue. In the two panels on the left, the intensity values of miR-320a in blood samples are presented. The two panels to the right show the values of miRNA-103 as recorded in tissue samples. The horizontal lines in each panel represent the respective median.

**Figure 2:**
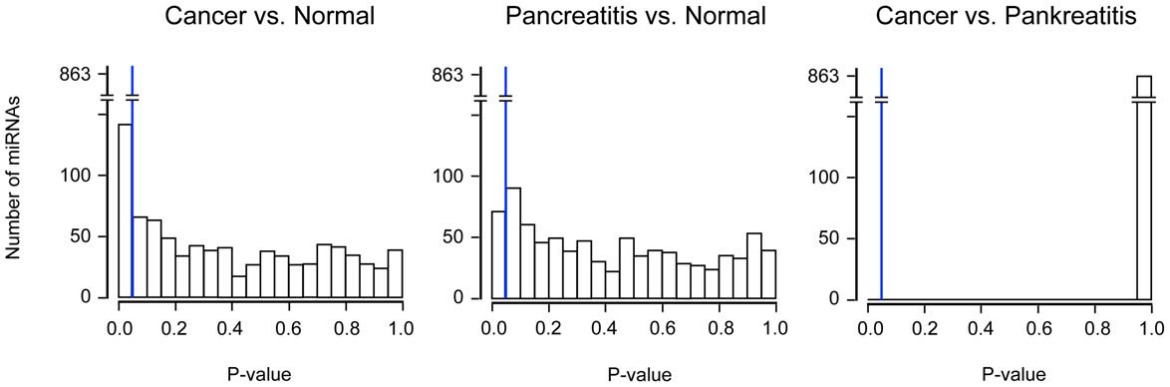
Number of significantly informative miRNAs in blood. The distribution of the 863 miRNAs across the entire p-value range is shown. The blue line denotes a significance value of 0.05. The bar to the left therefore indicates the number of significantly differentially expressed miRNAs. For classification purposes, however, also molecules of less significance could be informative.

In total, the complete miRNA signatures of 245 samples were analyzed. Relevant sample information is shown in Tab. S1. The 129 tissue samples consisted of 94 samples of pancreatic ductal adenocarcinoma, 19 samples of chronic pancreatitis and 16 normal tissues. All donors of normal tissue had died from complications other than pancreatic disease. Histology was done on all tissue samples in order to use only material of good and matching quality. The 116 blood samples were collected from a separate group of people: 45 samples were from patients with pancreatic ductal adenocarcinoma, 38 from chronic pancreatitis patients and 33 from healthy individuals. MicroRNA abundance was analyzed using the Geniom Real Time Analyzer and the Geniom biochip miRNA homo sapiens. Since the miRBase database, from which the sequences were taken, was upgraded twice from version 12.0 to 14.0 during the course of this work, the 863 miRNAs that were consistently present in all three versions were used for the final data analysis. Overall, 159 and 215 miRNAs exhibited significant expression variations in either blood or tissue, respectively (see Tab. S2); typical results are shown in Fig. 1. For independent confirmation, 398 real-time PCR assays were performed in triplicate. Taking into account the well-established variation between experimental systems, observed in particular for individual RNA-molecules, the results were in overall agreement with the information obtained from the microarray analyses (see Tab. S3). The significantly informative miRNAs were unevenly distributed between the pair-wise comparisons. In blood, 87 and 18 miRNAs differentiated between patients with ductal adenocarcinoma or chronic pancreatitis, respectively, and healthy individuals. Another 54 miRNAs were significantly different in both cancer and chronic pancreatitis compared to normal samples. However, there was not a single miRNA in blood that permitted drawing a distinction between the two diseases (Fig. 2; Tab. S2). Conversely, there were 100 miRNAs found in the tissue samples that permitted a separation of patients with ductal adenocarcinoma from those with chronic pancreatitis. In opposition to this, a mere six miRNAs (miR-7, miR-151-3p, miR-194, miR-486-5p, miR-514 and miR-1206) were discriminative only between chronic pancreatitis and normal tissue, substantially fewer than in the blood assay. Another 29 miRNAs were shared by cancer and pancreatitis as less specific markers for a disease of the pancreas (Tab. S2).

### Definition of classifiers

**Figure 3:**
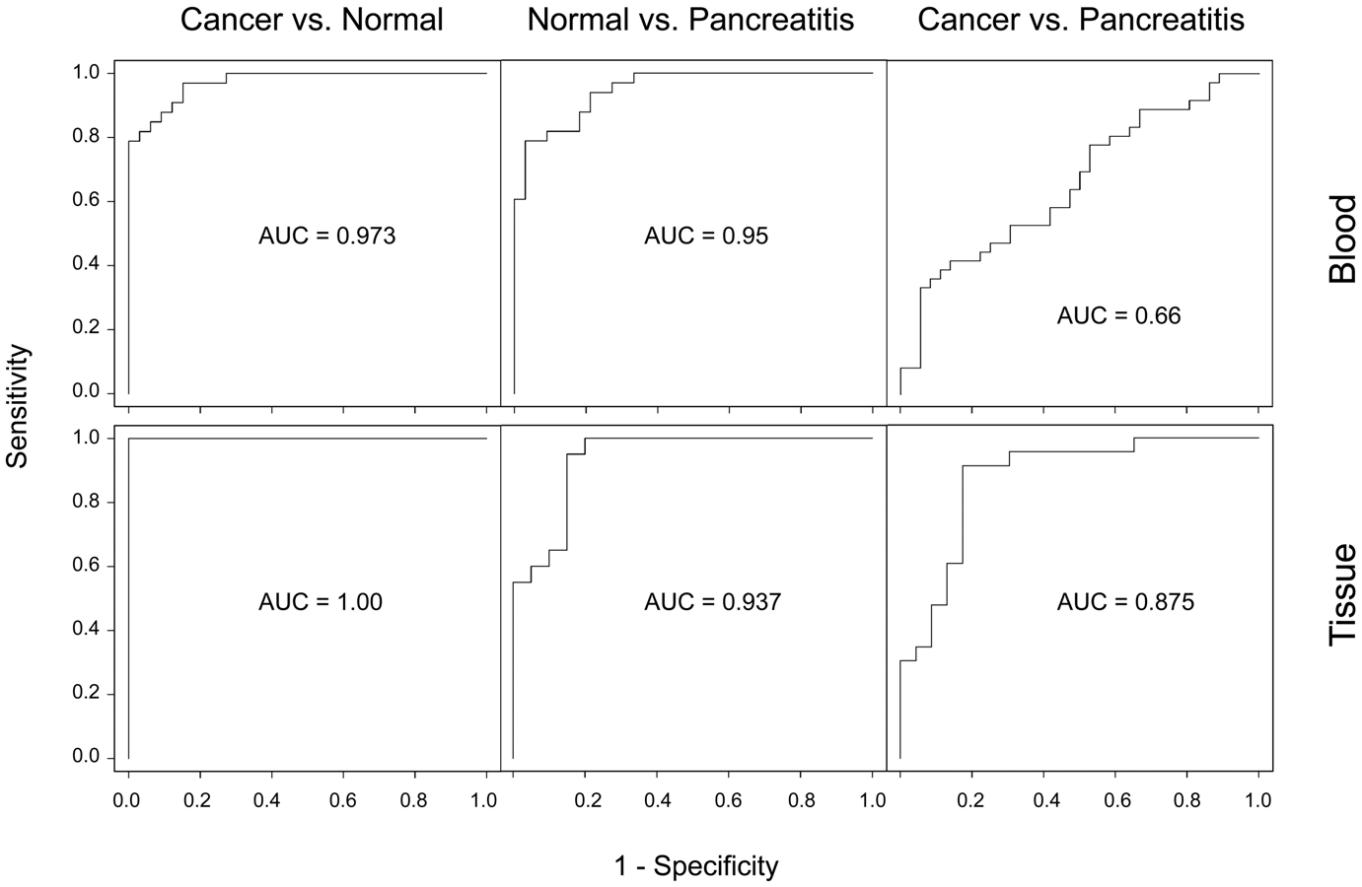
ROC curves calculated on the basis of the miRNA measurements. Receiver operating characteristic (ROC) curves are a widely accepted indicator of diagnostic utility. Measure of accuracy is the corresponding area under the ROC curve, denoted as AUC. It ranges in value from 0.5 (chance) to 1.0 (perfect discrimination).

No particular miRNA, however, was informative enough for a diagnosis of an individual patient because of the individual degree of variation, as apparent from the barplot results in Fig. 1 for example, although some are highly relevant population-wide or for a larger cohort such as the overall group of people examined in this study. Mutual Information (MI) [18] is a good measure to estimate the diagnostic information content of single biomarkers. However, even the miRNAs with the highest MI values were not sufficiently able to differentiate between healthy and diseased individuals with high specificity. For example, miR-320a (Fig. 1) separates blood cells of tumor patients from blood cells of healthy individuals with a specificity of 73% only, although exhibiting a high MI of 0.37 and an adjusted p-value of 0.002. In order to improve assay reliability and robustness, the predictive power of multiple miRNAs was combined by using statistical learning techniques. Support Vector Machines (SVM) were applied and a hypothesis test based on subset selection was carried out. For statistical significance, 100 repetitions of standard 10-fold cross validation were done. Likewise, 100 repetitions were computed for control permutation tests, in which samples with randomly assigned class labels were classified. The classification values obtained for accuracy, specificity and sensitivity are shown in Fig. S1 and visualized as receiver operating characteristics (ROC) curves in Fig. 3. In blood, area under the curve (AUC) values of 0.973 and 0.950 demonstrate the very high sensitivity and specificity of discriminating between healthy people and patients with cancer or chronic pancreatitis, respectively. The blood-based discrimination of cancer and pancreatitis proved no better than chance as indicated by the low AUC value and the similar results of the permutation tests (Fig. S1). For the other comparisons, however, significant figures were obtained, while the control permutation tests produced values that correspond to chance, as expected. This demonstrates that the real figures are not due to an over-fitting of the statistical model. Confirmative and in part even better diagnosis could be achieved with tissue samples. The AUC values for discriminating between healthy people and patients with cancer or chronic pancreatitis are 1.0 and 0.937, respectively. In addition, discrimination between cancer and chronic pancreatitis was achieved (AUC = 0.875). The results are consistent with the findings of unsupervised cluster analyses, also confirming the suitability of miRNA signatures for diagnostic purposes (not shown). The molecules producing the best classification results are labeled in Tab. S2. Dependent on the specific comparison, variable numbers were required. In tissue, only five molecules were sufficient for discrimination between cancer and normal, while 36 molecules were found necessary for the blood-based analysis.

**Figure 4:**
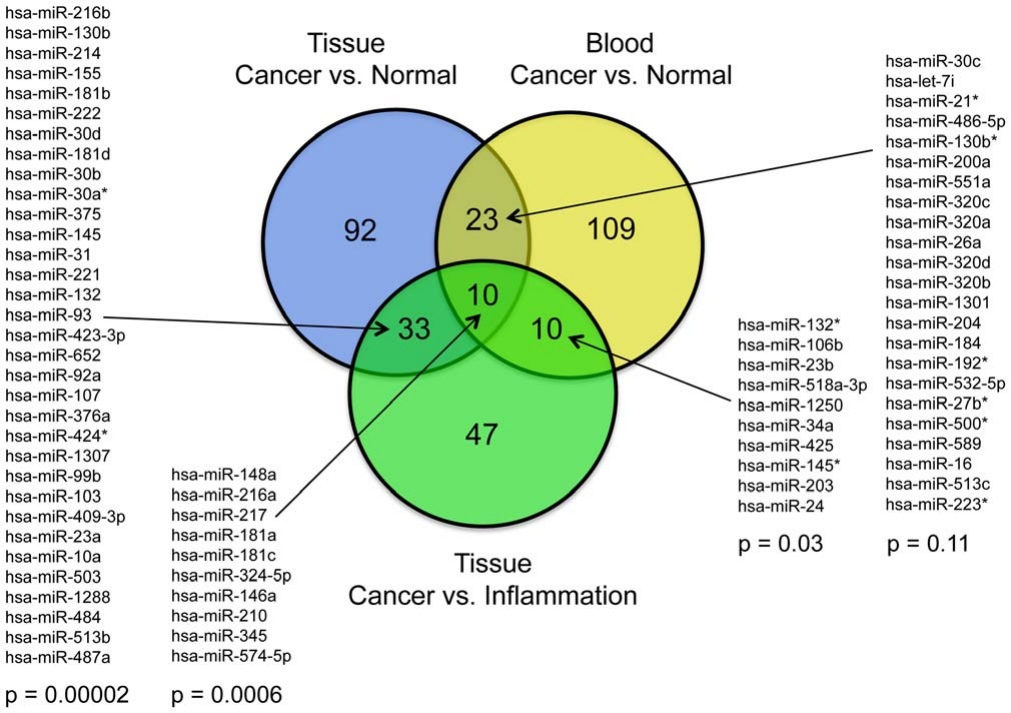
Venn diagram of the overlap of miRNA biomarkers. The miRNAs that are significant and specific for a blood-based diagnosis in patients with cancer versus healthy donors are shown in the yellow circle. The blue circle represents the markers that discriminate cancer and normal tissue, while the green circle stands for the markers that permit a distinction of cancer and inflammation in tissue, which is not possible in blood. The overlap in miRNAs between the tissue marker sets is significant; the relevant molecules are named.

The specificity of the identified miRNA marker molecules is documented in a Venn diagram (Fig. 4). To check whether any overlap of miRNA variations that are part of all three sets is statistically significant, 100,000 permutation tests were performed. The only very significant overlap for a pair-wise comparison was detected for miRNAs in tissue that were significant in both the cancer versus normal and cancer versus chronic pancreatitis comparisons. Here, we found 33 overlapping miRNAs while by chance only 17 would be expected (p = 0.00002). Likewise very significant was the overlap between all three sets. Here, the analysis predicted an average overlap of 3 miRNAs by chance while we computed an overlap of 10 miRNAs (p = 0.0006). The relevant miRNA molecules are listed in Fig. 4.

## Discussion

The measurement of miRNAs in blood offers an option for the non-invasive detection of chronic pancreatitis or pancreatic cancer. The accuracy of discrimination from any other disease is at least 81.3% [17], even if there is no prior knowledge about the disease status. Even without a therapeutic advance, this result is likely to have clinical consequences by increasing the number of curative resections. Also, for the evaluation of tumor reoccurrence in patients, who have undergone curative surgical resection, and for people with a familial risk of pancreatic cancer, the miRNA analysis could have a substantial effect on survival. The miRNA assay combines a minimally invasive nature with a robustness of the analyte molecules. The miRNA signatures exhibited enough reproducibility between patients for a robust detection of disease. The number of marker molecules required for such a result is nevertheless small enough to fit to assay systems used in routine diagnostics, in particular real-time PCR [19]. Both the blood and the tissue profiling could be supplemented by other data, such as messenger RNA profiles on tissue biopsy samples [20] or the resected tumor, produced from the very RNA-preparations utilized for miRNA detection. Also the testing of other body fluids and the combination with other analyte forms should improve the solidity and accuracy of non-invasive testing even further. For example, particular protein signatures in urine were recently reported [21], [22] that were indicative for pancreatic ductal carcinoma.

The analysis presented here was aimed at the establishment of a diagnostic modality. The apparent overlap of differentially expressed miRNAs in blood cells and tissues (Fig. 4) may indicate a functional relationship. Functional aspects of the miRNA level variations or groups thereof need to be studied further in order to elucidate the reason why clear variations can be found in both blood and tissue of diseased people and if the changes have a functional connection. In tissue, we also studied the mRNA level variations in the same set of RNA preparations. A comparative analysis of the miRNA and mRNA profiles is ongoing and will provide additional information on the functional effects of changes of miRNA expression in tumors and inflamed pancreas.

The hypothesis exists that inflammation, such as chronic pancreatitis, could be required as one step toward the development of pancreatic cancer. Given the fact that not all cases of pancreatic cancer are associated with chronic pancreatitis, it could be that other forms of inflammation have a similar effect. Although the actual inflammation may have occurred earlier and even gone unnoticed phenotypically, it may be possible that a miRNA signature, which is similar to the one observed for chronic pancreatitis, could serve as an indicator for an increased risk of developing pancreatic cancer, while the profiles of other inflammation diseases, such as the ones of chronic sarcoidosis, periodontitis and chronic obstructive pulmonary disease, which are clearly different [17], do not implicate a risk. Further studies are required to substantiate this assumption.

## Materials and Methods

### Ethics statement

For all samples analyzed, written informed consent was obtained from the patients and healthy individuals. Ethical approval was obtained from local ethics committees at the universities of Liverpool and Heidelberg; positive ethics votes 301/2001 (tissue) and 159/2002 (blood) of 28 December 2007.

### Tissue and blood samples

All patients included in this study were of stages II, III and IV and were untreated at the time of sample collection. The pancreatic tissue specimens were collected during surgery. The samples were snap-frozen in liquid nitrogen directly after resection and subsequently stored at −80°C until used for RNA preparation. For RNA isolation from blood, 5 ml blood were collected in PAXgene Blood RNA tubes (BD, Franklin Lakes, USA). No cancer treatment had been administered prior to sample collection.

### RNA isolation

Patient blood samples were centrifuged at 5000 g for 10 min at room temperature. For the isolation of total RNA including miRNA from the cell pellet, the miRNeasy kit (Qiagen, Hilden, Germany) was used according to the manufacturer's instructions. The resulting RNA was stored at −80°C.

Tissue samples were cut into slices of 20 µm thickness with a cryotome Leica CM 1850 UV at −34°C. During the process, also thinner slices were produced and studied histologically to assure that similar tissue qualities were used; the images can be made available. The 20 µm slices were submerged in liquid nitrogen and gently ground by three turns with a polypropylene micropestle (Eppendorf, Hamburg, Germany) that fits into 2 ml Eppendorf tubes. Total RNA was isolated with the AllPrep Isolation kit (Qiagen, Hilden, Germany), following a slightly modified protocol. Eighty percent ethanol was used for washing in order to obtain the small miRNA molecules. RNA integrity was evaluated using an Agilent 2100 Bioanalyzer (Agilent Technologies, Palo Alto, USA).

### Biochip hybridization and data processing

Samples were analyzed with the Geniom Realtime Analyzer (febit biomed, Heidelberg, Germany) using the Geniom Biochip miRNA homo sapiens. Each array contained seven replicates of all miRNAs as annotated in the Sanger miRBase [23]. Since the miRBase was upgraded twice from version 12.0 to 14.0 during the course of this work, the 863 miRNAs that were consistently present in all three versions were used for the final data analysis. Hybridization was at 42°C for 16 h in 2.5 M TMAC, 1× MES, 20 mM EDTA, pH 8, 0.01% Tween-20, 0.1 µg/µl herring sperm DNA and 33 ng/µl of directly labeled control oligonucleotides. Sample labeling with biotin was carried out by a microfluidic-based enzymatic on-chip protocol [24] and Flash-Tag RNA labeling (Genisphere, Hatfield, USA) according to manufacturer's protocol. Subsequently, the biochip was washed automatically and a program for signal enhancement was processed using a streptavidin-phycoerythrin conjugate (Invitrogen, Karlsruhe, Germany) and biotinylated anti-streptavidin goat antibody (Vector Laboratories, Peterborough, UK). The resulting images of the signal intensities were evaluated using the Geniom Wizard Software. Following background correction, the median was calculated from the seven replicate intensity values of each miRNA. For data normalization, variance stabilizing normalization (VSN) [25] was applied. Checking the technical reproducibility between experiments exhibited a mean correlation factor of 0.97. The microarray data were deposited in the publicly accessible database Gene Expression Omnibus (GEO; http://www.ncbi.nlm.nih.gov/projects/geo/, GSE24279). Also, the information is attached as Tab. S4.

### Evaluation by real-time PCR

Each RNA sample was checked for genomic DNA contaminations that could obscure the measurement. Total RNA was converted to cDNA using the miScript Reverse Transcriptase mix (Qiagen, Hilden, Germany) following the manufacturer's instructions. Quantification by real-time PCR was performed on a Light Cycler 480 instrument (Roche Diagnostics, Mannheim, Germany) using the miRNA SYBR Green PCR Kit and the QuantiTect primer assays (Qiagen). In tissue, 8 miRNAs were analyzed in 9 normal and 22 cancer samples. In blood, 5 miRNAs were confirmed in 10 samples each of ductal adenocarcinoma, chronic pancreatitis and healthy. Each analysis was done in triplicate, totaling 1194 reactions (Tab. S3).

### Statistical analysis

All statistical analyses were performed using R (http://www.R-project.org). Hierarchical clustering analysis was performed with the *hclust* function and *Heatplus* package. For top-down complete linkage clustering the Euclidian distance was used. The resulting dendrograms were cut at different heights and Fisher's exact test [26] (unpaired, two-tailed) was applied to compute p-values. To detect miRNAs that showed significant expression variations, we applied hypothesis testing for pair-wise comparisons. Following verification of an approximately normal distribution using a Shapiro-Wilk test [27], non-parametric Wilcoxon-Mann-Whithney tests [28] (unpaired, two-tailed) were computed for each miRNA. The resulting p-values were adjusted for multiple testing by Benjamini-Hochberg adjustment [29], [30].

Classification of samples using miRNA patterns was carried out using Support Vector Machines (SMV) as implemented in the R e1071 package [31] together with a miRNA subset selection technique. The most common SVM kernels, including linear SVMs, radial basis function, sigmoid kernels, as well as quadratic and polynomials were applied. The results presented in this study rely on the radial basis function SVM, showing superior performance compared to other kernels. The measured miRNA profiles were classified using 100 independent repetitions of 10-fold cross-validation. As a result, the mean accuracy, specificity, and sensitivity were calculated together with the 95% confidence intervals. To check for a so-called overtraining, we applied non-parametric permutation tests. For this, the class labels were sampled randomly and classifications were carried out using the permuted class labels. For computing the ROC curves, we used the bioconductor R package “ROC”.

## Supporting Information

Figure S1 **Boxplot presentation of classification results.**
(DOC)

Table S1 **Patient information.**
(DOC)

Table S2 **miRNAs that are significantly different in abundance.**
(DOC)

Table S3 **Results of the RT-PCR analysis.**
(DOC)

Table S4 **Basic data set of miRNA expression.**
(DOC)
