# Supplementary material for: Diagnosis of Pancreatic Ductal Adenocarcinoma and Chronic Pancreatitis by Measurement of microRNA Abundance in Blood and Tissue
Source: PLoS One. 2012 Apr 12;7(4):e34151. doi: 10.1371/journal.pone.0034151 (PMC3325244; doi:10.1371/journal.pone.0034151)
Supplement: Figure S1 — Boxplot presentation of classification results. (DOC) [file pone.0034151.s001.doc]

**Diagnosis of pancreatic ductal adenocarcinoma and chronic pancreatitis by measurement of microRNA abundance in blood and tissue**

**Supplemental Figure S1:** Boxplot presentation of classification results.

**
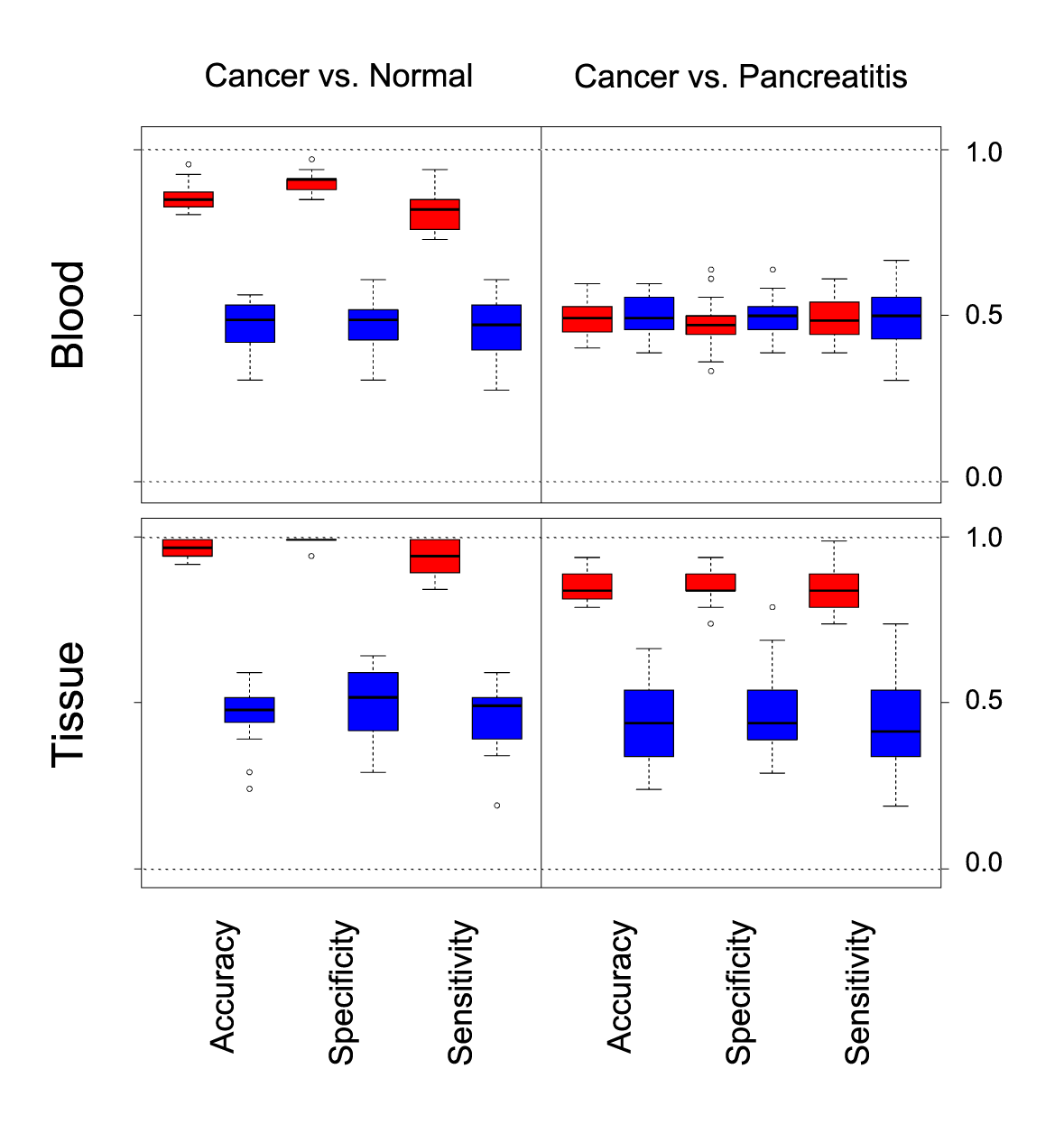
**

The results of blood- (top row) and tissue-based analyses (bottom row) are shown. The red boxes indicate the classification accuracy, specificity and sensitivity that can be achieved with miRNA signatures. The blue boxes show the corresponding result of a random permutation test.
