# Supplementary material for: Diagnosis of Pancreatic Ductal Adenocarcinoma and Chronic Pancreatitis by Measurement of microRNA Abundance in Blood and Tissue
Source: PLoS One. 2012 Apr 12;7(4):e34151. doi: 10.1371/journal.pone.0034151 (PMC3325244; doi:10.1371/journal.pone.0034151)
Supplement: Table S1 — Patient information. (DOC) [file pone.0034151.s002.doc]

**Diagnosis of pancreatic ductal adenocarcinoma and chronic pancreatitis by measurement of microRNA abundance in blood and tissue**

**Supplemental Table S1: Patient information**

Patient information of tissue samples:

| **ID** | **sex** | **age** | **histologie** | **tumor histologie** | **T** | **N** | **M** |
| --- | --- | --- | --- | --- | --- | --- | --- |
| Ge0014 | female | 58 | tumor of the pancreas | ductal adenocarcinoma | 4 | 1 | 0 |
| Ge0017 | female | 59 | tumor of the pancreas | ductal adenocarcinoma | 3 | 1 | 1 |
| Ge0018 | male | 64 | tumor of the pancreas | ductal adenocarcinoma | 3 | 1 | 0 |
| Ge0031 | male | 61 | tumor of the pancreas | ductal adenocarcinoma |  |  | 1 |
| Ge0036 | female | 67 | tumor of the pancreas | ductal adenocarcinoma | 3 | 1 | 0 |
| Ge0040 | male | 71 | tumor of the pancreas | ductal adenocarcinoma | 3 | 1 | 0 |
| Ge0041 | female | 70 | tumor of the pancreas | ductal adenocarcinoma | 3 | 1 | 1 |
| Ge0043 | female | 76 | tumor of the pancreas | ductal adenocarcinoma | 3 | 1 | 0 |
| Ge0046 | male | 60 | tumor of the pancreas | ductal adenocarcinoma | 3 | 1 | 0 |
| Ge0061 | female | 51 | tumor of the pancreas | ductal adenocarcinoma | 3 | 1 | 0 |
| Ge0078 | male | 63 | tumor of the pancreas | ductal adenocarcinoma | 3 | 1 | 0 |
| Ge0079 | female | 70 | tumor of the pancreas | ductal adenocarcinoma | 1 | 1 | 0 |
| Ge0080 | female | 70 | tumor of the pancreas | ductal adenocarcinoma | 3 | 1 | 0 |
| Ge0083 | male | 56 | tumor of the pancreas | ductal adenocarcinoma | 3 | 1 | 0 |
| Ge0095 | female | 65 | tumor of the pancreas | ductal adenocarcinoma | 3 | 0 | 0 |
| Ge0099 | female | 75 | tumor of the pancreas | ductal adenocarcinoma | 3 | 1 | 0 |
| Ge0101 | male | 65 | tumor of the pancreas | ductal adenocarcinoma | 3 | 1 | 1 |
| Ge0109 | male | 51 | tumor of the pancreas | ductal adenocarcinoma | 3 | 1 | 0 |
| Ge0111 | male | 47 | tumor of the pancreas | ductal adenocarcinoma | 3 | 1 | 0 |
| Ge0128 | male | 71 | tumor of the pancreas | ductal adenocarcinoma | 3 | 1 | 0 |
| Ge0143 | male | 55 | tumor of the pancreas | ductal adenocarcinoma | 3 | 1 | 0 |
| Ge0151 | female | 59 | tumor of the pancreas | ductal adenocarcinoma | 3 | 1 | 1 |
| Ge0152 | male | 59 | tumor of the pancreas | ductal adenocarcinoma | 3 | 1 | 0 |
| Ge0162 | male | 47 | tumor of the pancreas | ductal adenocarcinoma | 3 | 0 | 0 |
| Ge0164 | male | 42 | tumor of the pancreas | ductal adenocarcinoma | 3 | 1 | 0 |
| Ge0165 | male | 49 | tumor of the pancreas | ductal adenocarcinoma | 3 | 1 | 0 |
| Ge0167 | female | 40 | tumor of the pancreas | ductal adenocarcinoma | 3 | 1 | 0 |
| Ge0180 | male | 61 | tumor of the pancreas | ductal adenocarcinoma | 3 | 1 | 0 |
| Ge0184 | female | 43 | tumor of the pancreas | ductal adenocarcinoma | 3 | 1 | 0 |
| Ge0185 | male | 66 | tumor of the pancreas | ductal adenocarcinoma | 3 | 0 | 0 |
| Ge0202 | female | 59 | tumor of the pancreas | ductal adenocarcinoma | 3 | 0 | 0 |
| Ge0205 | male | 70 | tumor of the pancreas | ductal adenocarcinoma | 3 | 1 | 0 |
| Ge0234 | male | 56 | tumor of the pancreas | ductal adenocarcinoma | 3 | 1 | 0 |
| Ge0267 | female | 65 | tumor of the pancreas | ductal adenocarcinoma | 3 | 1 | 0 |
| Ge0288 | female | 54 | tumor of the pancreas | ductal adenocarcinoma | 3 | 1 | 1 |
| Ge0294 | female | 48 | tumor of the pancreas | ductal adenocarcinoma | 3 | 1 | 0 |
| Ge0308 | male | 63 | tumor of the pancreas | ductal adenocarcinoma | 3 | 1 | 0 |
| Ge0329 | male | 70 | tumor of the pancreas | ductal adenocarcinoma | 4 | 1 | 0 |
| Ge0382 | male | n.a. | tumor of the pancreas | ductal adenocarcinoma | 3 | 1 | 0 |
| Ge0389 | male | 60 | tumor of the pancreas | ductal adenocarcinoma | 3 | 1 | 0 |
| Ge0404 | male | 48 | tumor of the pancreas | ductal adenocarcinoma | 3 | 1 | 0 |
| Ge0408 | female | 78 | tumor of the pancreas | ductal adenocarcinoma | 3 | 1 | 0 |
| Ge0414 | female | 76 | tumor of the pancreas | ductal adenocarcinoma | 3 | 1 | 0 |
| Ge0416 | female | 74 | tumor of the pancreas | ductal adenocarcinoma | 3 | 0 | 0 |
| Ge0418 | male | 70 | tumor of the pancreas | ductal adenocarcinoma | 3 | 1 | 0 |
| Ge0421 | male | 78 | tumor of the pancreas | ductal adenocarcinoma | 3 | 1 | 0 |
| Ge0511 | male | 63 | tumor of the pancreas | ductal adenocarcinoma | 4 | 1 | 0 |
| Ge0519 | male | 85 | tumor of the pancreas | ductal adenocarcinoma | 3 | 1 | 0 |
| Ge0525 | male | 52 | tumor of the pancreas | ductal adenocarcinoma | 3 | 1 | 0 |
| Ge0534 | male | 56 | tumor of the pancreas | ductal adenocarcinoma | 3 | 1 | 0 |
| Ge0539 | male | 62 | tumor of the pancreas | ductal adenocarcinoma | 4 | 1 | 0 |
| Ge0543 | female | 74 | tumor of the pancreas | ductal adenocarcinoma | 3 | 1 | 0 |
| Ge0546 | male | 44 | tumor of the pancreas | ductal adenocarcinoma | 3 | 1 | 0 |
| Ge0553 | male | 72 | tumor of the pancreas | ductal adenocarcinoma | 3 | 1 | 0 |
| Ge0661 | female | n.a. | tumor of the pancreas | ductal adenocarcinoma | 3 | 1 | 0 |
| Ge0665 | male | 61 | tumor of the pancreas | ductal adenocarcinoma | 3 | 1 | 1 |
| Ge0673 | female | 72 | tumor of the pancreas | ductal adenocarcinoma | 3 | 1 | 0 |
| Ge0681 | male | 45 | tumor of the pancreas | ductal adenocarcinoma | 4 | 1 | 0 |
| Ge0683 | male | 79 | tumor of the pancreas | ductal adenocarcinoma | 3 | 1 | 0 |
| Ge0686 | male | 62 | tumor of the pancreas | ductal adenocarcinoma | 3 | 1 | 0 |
| Ge0687 | male | 62 | tumor of the pancreas | ductal adenocarcinoma | 3 | 1 | 0 |
| Ge0689 | male | 54 | tumor of the pancreas | ductal adenocarcinoma | 3 | 1 | 1 |
| Ge0690 | male | 84 | tumor of the pancreas | ductal adenocarcinoma | 3 | 1 | 0 |
| Ge0693 | male | 68 | tumor of the pancreas | ductal adenocarcinoma | 3 | 1 | 0 |
| Ge0870 | male | 47 | tumor of the pancreas | ductal adenocarcinoma | 3 | 1 | 0 |
| Ge0871 | female | 68 | tumor of the pancreas | ductal adenocarcinoma | 3 | 1 | 0 |
| Ge0354 | male | 73 | tumor of the pancreas | ductal adenocarcinoma |  |  |  |
| Ge0436 | male | 68 | tumor of the pancreas | ductal adenocarcinoma | 2 | 1a | 0 |
| Ge0439 | male | 67 | tumor of the pancreas | ductal adenocarcinoma | 3 | 1 |  |
| Ge0486 | male | 75 | tumor of the pancreas | ductal adenocarcinoma | 3 | 1b |  |
| Ge0493 | female | 62 | tumor of the pancreas | ductal adenocarcinoma | 3 | 0 |  |
| Ge0495 | male | 71 | tumor of the pancreas | ductal adenocarcinoma | 3 | 1b |  |
| Ge0498 | male | 68 | tumor of the pancreas | ductal adenocarcinoma | 3 | 0 |  |
| Ge0504 | male | 62 | tumor of the pancreas | ductal adenocarcinoma |  |  |  |
| Ge0564 | female | 71 | tumor of the pancreas | ductal adenocarcinoma | 3 | 1b |  |
| Ge0565 | male | 54 | tumor of the pancreas | ductal adenocarcinoma | 3 | 1b |  |
| Ge0566 | male | 52 | tumor of the pancreas | ductal adenocarcinoma | 3 | 1b |  |
| Ge0567 | female | 44 | tumor of the pancreas | ductal adenocarcinoma | 3 |  | 1 |
| Ge0568 | male | 64 | tumor of the pancreas | ductal adenocarcinoma | 2 | 0 |  |
| Ge0569 | female | 66 | tumor of the pancreas | ductal adenocarcinoma | 3 | 0 |  |
| Ge0573 | female | 73 | tumor of the pancreas | ductal adenocarcinoma | 3 | 1a |  |
| Ge0574 | male | 65 | tumor of the pancreas | ductal adenocarcinoma | 3 | 1b |  |
| Ge0597 | male | 75 | tumor of the pancreas | ductal adenocarcinoma | 2 |  |  |
| Ge0603 | female | 47 | tumor of the pancreas | ductal adenocarcinoma | 3 | 0 |  |
| Ge0604 | male | 66 | tumor of the pancreas | ductal adenocarcinoma | 3 | 1b |  |
| Ge0605 | male | 64 | tumor of the pancreas | ductal adenocarcinoma | 3 | 1 |  |
| Ge0630 | male | 53 | tumor of the pancreas | ductal adenocarcinoma | 3 | 1a |  |
| Ge0631 | female | 67 | tumor of the pancreas | ductal adenocarcinoma | 3 | 1b |  |
| Ge0637 | male | 62 | tumor of the pancreas | ductal adenocarcinoma | 3 | 1b |  |
| Ge0640 | male | 73 | tumor of the pancreas | ductal adenocarcinoma | 3 | 1b |  |
| Ge0642 | male | 77 | tumor of the pancreas | ductal adenocarcinoma | 3 | 1a |  |
| Ge0644 | n.a. | n.a. | tumor of the pancreas | ductal adenocarcinoma | 2 | 1b |  |
| Ge0648 | female | 75 | tumor of the pancreas | ductal adenocarcinoma | 3 | 1a |  |
| Ge0653 | female | 73 | tumor of the pancreas | ductal adenocarcinoma | 2 | 1 |  |
| Ge0089 | female | 52 | pancreatitis |  |  |  |  |
| Ge0093 | male | 63 | pancreatitis |  |  |  |  |
| Ge0097 | female | 55 | pancreatitis |  |  |  |  |
| Ge0113 | male | 45 | pancreatitis |  |  |  |  |
| Ge0117 | male | 41 | pancreatitis |  |  |  |  |
| Ge0125 | male | 64 | pancreatitis |  |  |  |  |
| Ge0130 | female | 53 | pancreatitis |  |  |  |  |
| Ge0132 | male | 52 | pancreatitis |  |  |  |  |
| Ge0133 | male | 44 | pancreatitis |  |  |  |  |
| Ge0136 | female | 49 | pancreatitis |  |  |  |  |
| Ge0161 | male | 53 | pancreatitis |  |  |  |  |
| Ge0171 | male | 56 | pancreatitis |  |  |  |  |
| Ge0181 | male | 44 | pancreatitis |  |  |  |  |
| Ge0200 | male | 64 | pancreatitis |  |  |  |  |
| Ge0212 | male | 56 | pancreatitis |  |  |  |  |
| Ge0236 | male | 39 | pancreatitis |  |  |  |  |
| Ge0255 | male | 51 | pancreatitis |  |  |  |  |
| Ge0556 | male | 69 | pancreatitis |  |  |  |  |
| Ge0688 | female | 51 | pancreatitis |  |  |  |  |
| Ge0052 | unknown | unknown | donor healthy pancreas |  |  |  |  |
| Ge0055 | unknown | unknown | donor healthy pancreas |  |  |  |  |
| Ge0056 | unknown | unknown | donor healthy pancreas |  |  |  |  |
| Ge0058 | unknown | unknown | donor healthy pancreas |  |  |  |  |
| Ge0141 | unknown | unknown | donor healthy pancreas |  |  |  |  |
| Ge0256 | unknown | unknown | donor healthy pancreas |  |  |  |  |
| Ge0257 | unknown | unknown | donor healthy pancreas |  |  |  |  |
| Ge0259 | unknown | unknown | donor healthy pancreas |  |  |  |  |
| Ge0262 | unknown | unknown | donor healthy pancreas |  |  |  |  |
| Ge0264 | unknown | unknown | donor healthy pancreas |  |  |  |  |
| Ge0423 | unknown | unknown | donor healthy pancreas |  |  |  |  |
| Ge0424 | unknown | unknown | donor healthy pancreas |  |  |  |  |
| Ge0872 | unknown | unknown | donor healthy pancreas |  |  |  |  |
| Ge0873 | unknown | unknown | donor healthy pancreas |  |  |  |  |
| Ge0874 | unknown | unknown | donor healthy pancreas |  |  |  |  |
| Ge0877 | unknown | unknown | donor healthy pancreas |  |  |  |  |

Patient information of blood samples:

| **ID** | **sex** | **age** | **histology** | **tumor histology** | **T** | **N** | **M** |
| --- | --- | --- | --- | --- | --- | --- | --- |
| vb010 | female | 69 | tumor of the pancreas | ductal adenocarcinoma | 4 | 1 | 0 |
| vb100 | female | 57 | tumor of the pancreas | ductal adenocarcinoma | 3 | 1 | 1 |
| vb104 | female | 57 | tumor of the pancreas | ductal adenocarcinoma |  |  |  |
| vb105 | male | 44 | tumor of the pancreas | ductal adenocarcinoma |  |  | 1 |
| vb107 | female | 61 | tumor of the pancreas | ductal adenocarcinoma | 3 | 1 | 0 |
| vb114 | male | 67 | tumor of the pancreas | ductal adenocarcinoma | 3 | 1 | 0 |
| vb115 | male | 77 | tumor of the pancreas | ductal adenocarcinoma | 3 | 0 | 0 |
| vb022 | male | 65 | tumor of the pancreas | ductal adenocarcinoma | 3 | 0 | 0 |
| vb023 | male | 67 | tumor of the pancreas | ductal adenocarcinoma | 3 | 1 | 0 |
| vb025 | female | 68 | tumor of the pancreas | ductal adenocarcinoma | 3 | 1 | 0 |
| vb028 | female | 72 | tumor of the pancreas | ductal adenocarcinoma | 3 | 1 | 0 |
| vb004 | male | 64 | tumor of the pancreas | ductal adenocarcinoma |  |  |  |
| vb043 | female | 55 | tumor of the pancreas | ductal adenocarcinoma |  |  |  |
| vb045 | male | 74 | tumor of the pancreas | ductal adenocarcinoma |  |  |  |
| vb046 | male | 65 | tumor of the pancreas | ductal adenocarcinoma |  |  |  |
| vb051 | female | 69 | tumor of the pancreas | ductal adenocarcinoma |  |  |  |
| vb057 | male | 64 | tumor of the pancreas | ductal adenocarcinoma |  |  |  |
| vb058 | male | 62 | tumor of the pancreas | ductal adenocarcinoma |  |  |  |
| vb059 | male | 64 | tumor of the pancreas | ductal adenocarcinoma |  |  |  |
| vb060 | male | 70 | tumor of the pancreas | ductal adenocarcinoma |  |  |  |
| vb068 | male | 68 | tumor of the pancreas | ductal adenocarcinoma |  |  |  |
| vb007 | male | 70 | tumor of the pancreas | ductal adenocarcinoma | 3 | 1 | 0 |
| vb071 | female | 64 | tumor of the pancreas | ductal adenocarcinoma |  |  |  |
| vb072 | male | 57 | tumor of the pancreas | ductal adenocarcinoma |  |  |  |
| vb073 | female | 60 | tumor of the pancreas | ductal adenocarcinoma |  |  |  |
| vb074 | female | 70 | tumor of the pancreas | ductal adenocarcinoma | 3 | 1 | 0 |
| vb076 | male | 72 | tumor of the pancreas | ductal adenocarcinoma | 3 | 0 |  |
| vb077 | female | 51 | tumor of the pancreas | ductal adenocarcinoma | 3 | 1 | 0 |
| vb008 | female | 69 | tumor of the pancreas | ductal adenocarcinoma | 3 | 1 | 0 |
| vb080 | male | 49 | tumor of the pancreas | ductal adenocarcinoma | 3 | 1 | 0 |
| vb085 | male | 65 | tumor of the pancreas | ductal adenocarcinoma | 3 | 1 | 0 |
| vb087 | female | 60 | tumor of the pancreas | ductal adenocarcinoma | 3 | 1 | 1 |
| vb009 | female | 68 | tumor of the pancreas | ductal adenocarcinoma | 3 | 1 | 0 |
| vb096 | female | 72 | tumor of the pancreas | ductal adenocarcinoma | 3 | 0 | 0 |
| vb098 | male | 64 | tumor of the pancreas | ductal adenocarcinoma | 3 | 1 | 0 |
| vb145 | female | 54 | tumor of the pancreas | ductal adenocarcinoma | 3 | 0 | 0 |
| vb150 | male | 41 | tumor of the pancreas | ductal adenocarcinoma |  |  |  |
| vb152 | female | 69 | tumor of the pancreas | ductal adenocarcinoma | 0 | 0 | 0 |
| vb155 | male | 71 | tumor of the pancreas | ductal adenocarcinoma | 3 | 1 | 0 |
| vb163 | male | 68 | tumor of the pancreas | ductal adenocarcinoma |  |  |  |
| vb167 | male | 60 | tumor of the pancreas | ductal adenocarcinoma | Tis | 0 | 0 |
| vb170 | female | 78 | tumor of the pancreas | ductal adenocarcinoma | 4 | 0 | 0 |
| vb173 | female | 67 | tumor of the pancreas | ductal adenocarcinoma | 3 | 1 | 0 |
| vb027 | male | 62 | tumor of the pancreas | ductal adenocarcinoma |  |  |  |
| vb017 | female | 61 | tumor of the pancreas | ductal adenocarcinoma |  |  |  |
| vb094 | male | 72 | pancreatitis |  |  |  |  |
| vb144 | female | 66 | pancreatitis |  |  |  |  |
| vb147 | female | 38 | pancreatitis |  |  |  |  |
| vb148 | male | 36 | pancreatitis |  |  |  |  |
| vb160 | male | 69 | pancreatitis |  |  |  |  |
| vb161 | male | 38 | pancreatitis |  |  |  |  |
| vb162 | male | 51 | pancreatitis |  |  |  |  |
| vb168 | male | 55 | pancreatitis |  |  |  |  |
| vb169 | male | 50 | pancreatitis |  |  |  |  |
| vb172 | male | 73 | pancreatitis |  |  |  |  |
| vb175 | male | 59 | pancreatitis |  |  |  |  |
| vb062 | female | 53 | pancreatitis |  |  |  |  |
| vb069 | male | 51 | pancreatitis |  |  |  |  |
| vb016 | male | 55 | pancreatitis |  |  |  |  |
| vb015 | male | 43 | pancreatitis |  |  |  |  |
| vb020 | female | 71 | pancreatitis |  |  |  |  |
| vb106 | female | 47 | pancreatitis |  |  |  |  |
| vb108 | male | 70 | pancreatitis |  |  |  |  |
| vb109 | male | 48 | pancreatitis |  |  |  |  |
| vb110 | female | 68 | pancreatitis |  |  |  |  |
| vb112 | male | 53 | pancreatitis |  |  |  |  |
| vb113 | female | 61 | pancreatitis |  |  |  |  |
| vb121 | male | 46 | pancreatitis |  |  |  |  |
| vb021 | female | 53 | pancreatitis |  |  |  |  |
| vb024 | male | 56 | pancreatitis |  |  |  |  |
| vb003 | male | 52 | pancreatitis |  |  |  |  |
| vb044 | male | 54 | pancreatitis |  |  |  |  |
| vb055 | male | 48 | pancreatitis |  |  |  |  |
| vb061 | female | 49 | pancreatitis |  |  |  |  |
| vb066 | male | 47 | pancreatitis |  |  |  |  |
| vb067 | female | 51 | pancreatitis |  |  |  |  |
| vb081 | male | 29 | pancreatitis |  |  |  |  |
| vb086 | male | 46 | pancreatitis |  |  |  |  |
| vb088 | male | 52 | pancreatitis |  |  |  |  |
| vb090 | male | 38 | pancreatitis |  |  |  |  |
| vb092 | female | 52 | pancreatitis |  |  |  |  |
| vb099 | male | 50 | pancreatitis |  |  |  |  |
| vb052 | male | 57 | pancreatitis |  |  |  |  |
| vb131 | male | 24 | donor healthy pancreas |  |  |  |  |
| vb132 | female | 32 | donor healthy pancreas |  |  |  |  |
| vb133 | male | 32 | donor healthy pancreas |  |  |  |  |
| vb134 | female | 44 | donor healthy pancreas |  |  |  |  |
| vb137 | male | 31 | donor healthy pancreas |  |  |  |  |
| vb138 | male | 19 | donor healthy pancreas |  |  |  |  |
| vb139 | male | 23 | donor healthy pancreas |  |  |  |  |
| vb140 | female | 37 | donor healthy pancreas |  |  |  |  |
| vb141 | male | 30 | donor healthy pancreas |  |  |  |  |
| vb142 | female | 39 | donor healthy pancreas |  |  |  |  |
| vb143 | female | 30 | donor healthy pancreas |  |  |  |  |
| b1 | male | 27 | donor healthy pancreas |  |  |  |  |
| b2 | male | 58 | donor healthy pancreas |  |  |  |  |
| b3 | male | 27 | donor healthy pancreas |  |  |  |  |
| b4 | female | 22 | donor healthy pancreas |  |  |  |  |
| b5 | female | 59 | donor healthy pancreas |  |  |  |  |
| b6 | male | 34 | donor healthy pancreas |  |  |  |  |
| b7 | female | 23 | donor healthy pancreas |  |  |  |  |
| b8 | male | 40 | donor healthy pancreas |  |  |  |  |
| b9 | male | 57 | donor healthy pancreas |  |  |  |  |
| b10 | male | 60 | donor healthy pancreas |  |  |  |  |
| b11 | female | 46 | donor healthy pancreas |  |  |  |  |
| b12 | female | 24 | donor healthy pancreas |  |  |  |  |
| b13 | female | 27 | donor healthy pancreas |  |  |  |  |
| b14 | female | 28 | donor healthy pancreas |  |  |  |  |
| b15 | female | 28 | donor healthy pancreas |  |  |  |  |
| b16 | female | 28 | donor healthy pancreas |  |  |  |  |
| b17 | female | 49 | donor healthy pancreas |  |  |  |  |
| b18 | female | 35 | donor healthy pancreas |  |  |  |  |
| b19 | female | 41 | donor healthy pancreas |  |  |  |  |
| b20 | female | 58 | donor healthy pancreas |  |  |  |  |
| b21 | female | 25 | donor healthy pancreas |  |  |  |  |
| b22 | female | 28 | donor healthy pancreas |  |  |  |  |
