# Supplementary material for: Diagnosis of Pancreatic Ductal Adenocarcinoma and Chronic Pancreatitis by Measurement of microRNA Abundance in Blood and Tissue
Source: PLoS One. 2012 Apr 12;7(4):e34151. doi: 10.1371/journal.pone.0034151 (PMC3325244; doi:10.1371/journal.pone.0034151)
Supplement: Table S2 — miRNAs that are significantly different in abundance. (DOC) [file pone.0034151.s003.doc]

**Diagnosis of pancreatic ductal adenocarcinoma and chronic pancreatitis by measurement of microRNA abundance in blood and tissue**

**Supplemental Table S2:**

**miRNAs that are significantly different in abundance**

The molecule order is according to adjusted p values. Molecules that produce the best classification results for discriminating cancer are labelled in green.

Significant miRNAs normal vs. cancer in blood:

|  | **median normal** | **median PDAC** | **qmedian** | **logqmedian** | **wmw_raw p** | **wmw_adj p** | **ttest_raw p** | **ttest_adj p** | **AUC** |
| --- | --- | --- | --- | --- | --- | --- | --- | --- | --- |
| hsa-miR-126S | 1,00 | 34,53 | 0,03 | -3,54 | 0,00 | 0,00 | 0,00 | 0,002 | 0,21 |
| hsa-miR-539 | 1,00 | 21,18 | 0,05 | -3,05 | 0,00 | 0,00 | 0,00 | 0,002 | 0,26 |
| hsa-miR-1826 | 217,48 | 158,04 | 1,38 | 0,32 | 0,00 | 0,00 | 0,00 | 0,002 | 0,77 |
| hsa-miR-320a | 12702,71 | 6067,71 | 2,09 | 0,74 | 0,00 | 0,00 | 0,00 | 0,002 | 0,81 |
| hsa-miR-508-3p | 1,00 | 32,26 | 0,03 | -3,47 | 0,00 | 0,01 | 0,00 | 0,003 | 0,27 |
| hsa-miR-576-5p | 1,00 | 28,68 | 0,03 | -3,36 | 0,00 | 0,01 | 0,00 | 0,003 | 0,26 |
| hsa-miR-130bS | 1,00 | 16,95 | 0,06 | -2,83 | 0,00 | 0,01 | 0,00 | 0,003 | 0,27 |
| hsa-miR-196a | 1,00 | 15,88 | 0,06 | -2,76 | 0,00 | 0,00 | 0,00 | 0,003 | 0,27 |
| hsa-miR-302d | 1,00 | 12,07 | 0,08 | -2,49 | 0,00 | 0,01 | 0,00 | 0,003 | 0,28 |
| hsa-miR-885-5p | 15,63 | 67,47 | 0,23 | -1,46 | 0,00 | 0,01 | 0,00 | 0,003 | 0,25 |
| hsa-miR-196b | 1,00 | 3,09 | 0,32 | -1,13 | 0,00 | 0,00 | 0,00 | 0,003 | 0,27 |
| hsa-miR-223S | 23,28 | 62,56 | 0,37 | -0,99 | 0,00 | 0,02 | 0,00 | 0,003 | 0,29 |
| hsa-miR-23b | 2872,95 | 3749,72 | 0,77 | -0,27 | 0,00 | 0,00 | 0,00 | 0,003 | 0,23 |
| hsa-miR-135a | 1,00 | 1,00 | 1,00 | 0,00 | 0,00 | 0,00 | 0,00 | 0,003 | 0,28 |
| hsa-miR-200a | 118,53 | 84,59 | 1,40 | 0,34 | 0,00 | 0,00 | 0,00 | 0,003 | 0,76 |
| hsa-miR-106b | 14207,24 | 7477,04 | 1,90 | 0,64 | 0,00 | 0,00 | 0,00 | 0,003 | 0,79 |
| hsa-miR-367 | 101,12 | 46,98 | 2,15 | 0,77 | 0,00 | 0,00 | 0,00 | 0,003 | 0,77 |
| hsa-miR-556-5p | 155,87 | 69,82 | 2,23 | 0,80 | 0,00 | 0,00 | 0,00 | 0,003 | 0,77 |
| hsa-miR-541 | 132,68 | 57,16 | 2,32 | 0,84 | 0,00 | 0,00 | 0,00 | 0,003 | 0,82 |
| hsa-miR-601 | 1,00 | 42,11 | 0,02 | -3,74 | 0,00 | 0,01 | 0,00 | 0,005 | 0,26 |
| hsa-miR-135b | 1,00 | 24,02 | 0,04 | -3,18 | 0,00 | 0,01 | 0,00 | 0,005 | 0,27 |
| hsa-miR-184 | 4,70 | 46,21 | 0,10 | -2,29 | 0,00 | 0,01 | 0,00 | 0,005 | 0,28 |
| hsa-miR-301a | 360,04 | 223,01 | 1,61 | 0,48 | 0,00 | 0,00 | 0,00 | 0,005 | 0,76 |
| hsa-miR-532-5p | 237,97 | 128,53 | 1,85 | 0,62 | 0,00 | 0,00 | 0,00 | 0,005 | 0,78 |
| hsa-miR-132S | 1,00 | 8,11 | 0,12 | -2,09 | 0,00 | 0,00 | 0,00 | 0,006 | 0,23 |
| hsa-miR-449b | 39,26 | 71,07 | 0,55 | -0,59 | 0,00 | 0,02 | 0,00 | 0,006 | 0,29 |
| hsa-miR-522S | 169,59 | 93,40 | 1,82 | 0,60 | 0,00 | 0,01 | 0,00 | 0,006 | 0,74 |
| hsa-miR-519b-5p | 163,75 | 87,91 | 1,86 | 0,62 | 0,00 | 0,01 | 0,00 | 0,007 | 0,75 |
| hsa-miR-148a | 804,17 | 559,47 | 1,44 | 0,36 | 0,00 | 0,00 | 0,00 | 0,008 | 0,76 |
| hsa-miR-181c | 161,66 | 83,67 | 1,93 | 0,66 | 0,00 | 0,01 | 0,00 | 0,008 | 0,73 |
| hsa-miR-590-5p | 192,66 | 96,35 | 2,00 | 0,69 | 0,00 | 0,00 | 0,00 | 0,008 | 0,83 |
| hsa-miR-26a-2S | 1,00 | 1,00 | 1,00 | 0,00 | 0,00 | 0,01 | 0,00 | 0,009 | 0,31 |
| hsa-miR-581 | 7,61 | 20,86 | 0,37 | -1,01 | 0,01 | 0,06 | 0,00 | 0,010 | 0,34 |
| hsa-miR-1282 | 1,00 | 1,00 | 1,00 | 0,00 | 0,01 | 0,04 | 0,00 | 0,010 | 0,35 |
| hsa-miR-548b-5p | 1,00 | 1,00 | 1,00 | 0,00 | 0,00 | 0,03 | 0,00 | 0,010 | 0,33 |
| hsa-miR-181a | 626,33 | 287,97 | 2,17 | 0,78 | 0,00 | 0,00 | 0,00 | 0,010 | 0,77 |
| hsa-miR-122 | 10,00 | 49,65 | 0,20 | -1,60 | 0,00 | 0,01 | 0,00 | 0,011 | 0,27 |
| hsa-miR-608 | 64,91 | 178,50 | 0,36 | -1,01 | 0,00 | 0,01 | 0,00 | 0,012 | 0,25 |
| hsa-miR-192S | 130,11 | 82,77 | 1,57 | 0,45 | 0,00 | 0,00 | 0,00 | 0,012 | 0,76 |
| hsa-miR-1303 | 29,28 | 58,98 | 0,50 | -0,70 | 0,00 | 0,02 | 0,00 | 0,013 | 0,30 |
| hsa-miR-491-3p | 102,50 | 57,58 | 1,78 | 0,58 | 0,00 | 0,01 | 0,00 | 0,013 | 0,75 |
| hsa-miR-21S | 119,40 | 59,54 | 2,01 | 0,70 | 0,00 | 0,01 | 0,00 | 0,013 | 0,72 |
| hsa-miR-450a | 1,00 | 32,82 | 0,03 | -3,49 | 0,00 | 0,01 | 0,00 | 0,014 | 0,29 |
| hsa-miR-194S | 46,88 | 83,30 | 0,56 | -0,57 | 0,00 | 0,01 | 0,00 | 0,014 | 0,28 |
| hsa-miR-1909 | 111,34 | 148,29 | 0,75 | -0,29 | 0,00 | 0,01 | 0,00 | 0,014 | 0,28 |
| hsa-miR-374bS | 18,00 | 58,70 | 0,31 | -1,18 | 0,00 | 0,01 | 0,00 | 0,015 | 0,28 |
| hsa-miR-637 | 3,09 | 40,28 | 0,08 | -2,57 | 0,01 | 0,05 | 0,00 | 0,016 | 0,33 |
| hsa-miR-34a | 19,40 | 43,25 | 0,45 | -0,80 | 0,00 | 0,02 | 0,00 | 0,016 | 0,29 |
| hsa-miR-760 | 31,88 | 69,68 | 0,46 | -0,78 | 0,00 | 0,02 | 0,00 | 0,016 | 0,29 |
| hsa-miR-16 | 14207,24 | 17675,49 | 0,80 | -0,22 | 0,00 | 0,02 | 0,00 | 0,016 | 0,30 |
| hsa-miR-211 | 1,00 | 1,00 | 1,00 | 0,00 | 0,00 | 0,03 | 0,00 | 0,016 | 0,34 |
| hsa-miR-1283 | 125,65 | 83,11 | 1,51 | 0,41 | 0,00 | 0,01 | 0,00 | 0,016 | 0,74 |
| hsa-miR-204 | 1,00 | 12,07 | 0,08 | -2,49 | 0,00 | 0,00 | 0,00 | 0,017 | 0,26 |
| hsa-let-7i | 783,99 | 372,43 | 2,11 | 0,74 | 0,00 | 0,01 | 0,00 | 0,017 | 0,75 |
| hsa-miR-518a-3p | 71,35 | 31,75 | 2,25 | 0,81 | 0,00 | 0,01 | 0,00 | 0,017 | 0,73 |
| hsa-miR-595 | 127,83 | 255,04 | 0,50 | -0,69 | 0,00 | 0,02 | 0,00 | 0,018 | 0,30 |
| hsa-miR-1261 | 1,00 | 2,88 | 0,35 | -1,06 | 0,00 | 0,01 | 0,00 | 0,019 | 0,29 |
| hsa-let-7b | 966,00 | 587,21 | 1,65 | 0,50 | 0,00 | 0,02 | 0,00 | 0,019 | 0,71 |
| hsa-miR-513c | 1,00 | 24,95 | 0,04 | -3,22 | 0,00 | 0,01 | 0,00 | 0,020 | 0,29 |
| hsa-miR-1250 | 33,75 | 106,16 | 0,32 | -1,15 | 0,00 | 0,01 | 0,00 | 0,020 | 0,27 |
| hsa-miR-1183 | 100,70 | 177,11 | 0,57 | -0,56 | 0,00 | 0,02 | 0,00 | 0,020 | 0,29 |
| hsa-miR-124S | 119,96 | 87,96 | 1,36 | 0,31 | 0,00 | 0,02 | 0,00 | 0,020 | 0,70 |
| hsa-miR-380S | 99,33 | 71,07 | 1,40 | 0,33 | 0,00 | 0,02 | 0,00 | 0,020 | 0,70 |
| hsa-miR-18a | 1470,36 | 840,00 | 1,75 | 0,56 | 0,00 | 0,01 | 0,00 | 0,020 | 0,72 |
| hsa-miR-208b | 88,16 | 45,40 | 1,94 | 0,66 | 0,00 | 0,01 | 0,00 | 0,020 | 0,72 |
| hsa-miR-206 | 1,00 | 28,07 | 0,04 | -3,33 | 0,00 | 0,01 | 0,00 | 0,021 | 0,28 |
| hsa-miR-373 | 1,00 | 12,75 | 0,08 | -2,55 | 0,01 | 0,03 | 0,00 | 0,022 | 0,33 |
| hsa-miR-133b | 8,18 | 30,14 | 0,27 | -1,30 | 0,02 | 0,07 | 0,00 | 0,022 | 0,34 |
| hsa-miR-556-3p | 1,00 | 2,88 | 0,35 | -1,06 | 0,01 | 0,04 | 0,00 | 0,022 | 0,34 |
| hsa-miR-1268 | 348,62 | 559,47 | 0,62 | -0,47 | 0,00 | 0,01 | 0,00 | 0,022 | 0,26 |
| hsa-miR-133a | 58,33 | 87,91 | 0,66 | -0,41 | 0,01 | 0,05 | 0,00 | 0,022 | 0,32 |
| hsa-miR-1301 | 229,38 | 130,57 | 1,76 | 0,56 | 0,00 | 0,01 | 0,00 | 0,022 | 0,74 |
| hsa-miR-516bS | 12,45 | 46,77 | 0,27 | -1,32 | 0,00 | 0,01 | 0,00 | 0,023 | 0,27 |
| hsa-miR-455-5p | 10,00 | 28,68 | 0,35 | -1,05 | 0,02 | 0,07 | 0,00 | 0,023 | 0,34 |
| hsa-miR-548j | 1,00 | 1,39 | 0,72 | -0,33 | 0,01 | 0,04 | 0,00 | 0,023 | 0,34 |
| hsa-miR-26a | 6386,57 | 7079,20 | 0,90 | -0,10 | 0,01 | 0,05 | 0,00 | 0,023 | 0,32 |
| hsa-miR-216a | 186,36 | 101,33 | 1,84 | 0,61 | 0,00 | 0,01 | 0,00 | 0,023 | 0,75 |
| hsa-miR-335S | 1,00 | 18,63 | 0,05 | -2,92 | 0,00 | 0,01 | 0,00 | 0,024 | 0,28 |
| hsa-miR-9 | 1,00 | 22,60 | 0,04 | -3,12 | 0,03 | 0,12 | 0,00 | 0,025 | 0,37 |
| hsa-miR-769-3p | 1,00 | 18,63 | 0,05 | -2,92 | 0,01 | 0,06 | 0,00 | 0,025 | 0,34 |
| hsa-miR-1306 | 25,67 | 99,14 | 0,26 | -1,35 | 0,02 | 0,08 | 0,00 | 0,025 | 0,34 |
| hsa-miR-520b | 9,72 | 29,16 | 0,33 | -1,10 | 0,04 | 0,12 | 0,00 | 0,025 | 0,36 |
| hsa-miR-1293 | 19,96 | 58,84 | 0,34 | -1,08 | 0,00 | 0,03 | 0,00 | 0,025 | 0,31 |
| hsa-miR-574-5p | 937,44 | 1470,36 | 0,64 | -0,45 | 0,00 | 0,02 | 0,00 | 0,025 | 0,29 |
| hsa-miR-324-5p | 357,27 | 302,11 | 1,18 | 0,17 | 0,01 | 0,04 | 0,00 | 0,025 | 0,68 |
| hsa-miR-566 | 99,14 | 83,30 | 1,19 | 0,17 | 0,01 | 0,06 | 0,00 | 0,025 | 0,67 |
| hsa-miR-487b | 63,75 | 42,32 | 1,51 | 0,41 | 0,01 | 0,04 | 0,00 | 0,025 | 0,69 |
| hsa-miR-891b | 135,09 | 81,04 | 1,67 | 0,51 | 0,00 | 0,01 | 0,00 | 0,025 | 0,72 |
| hsa-miR-146a | 201,85 | 119,72 | 1,69 | 0,52 | 0,00 | 0,02 | 0,00 | 0,025 | 0,71 |
| hsa-miR-551a | 53,30 | 17,02 | 3,13 | 1,14 | 0,00 | 0,01 | 0,00 | 0,025 | 0,73 |
| hsa-miR-509-3p | 1,00 | 16,82 | 0,06 | -2,82 | 0,02 | 0,09 | 0,00 | 0,029 | 0,36 |
| hsa-miR-548d-5p | 1,00 | 14,81 | 0,07 | -2,70 | 0,00 | 0,02 | 0,00 | 0,029 | 0,31 |
| hsa-miR-1262 | 1,00 | 4,42 | 0,23 | -1,49 | 0,00 | 0,02 | 0,00 | 0,029 | 0,32 |
| hsa-miR-920 | 26,82 | 58,61 | 0,46 | -0,78 | 0,05 | 0,14 | 0,00 | 0,029 | 0,37 |
| hsa-miR-490-5p | 147,86 | 106,16 | 1,39 | 0,33 | 0,00 | 0,02 | 0,00 | 0,029 | 0,71 |
| hsa-miR-202S | 112,08 | 71,51 | 1,57 | 0,45 | 0,00 | 0,02 | 0,00 | 0,029 | 0,70 |
| hsa-miR-199b-5p | 1,00 | 14,81 | 0,07 | -2,70 | 0,01 | 0,06 | 0,00 | 0,030 | 0,35 |
| hsa-miR-92a-2S | 1,00 | 27,33 | 0,04 | -3,31 | 0,00 | 0,01 | 0,00 | 0,031 | 0,30 |
| hsa-miR-29b-1S | 1,00 | 12,25 | 0,08 | -2,51 | 0,01 | 0,04 | 0,00 | 0,031 | 0,33 |
| hsa-miR-32S | 29,28 | 106,96 | 0,27 | -1,30 | 0,00 | 0,02 | 0,00 | 0,031 | 0,29 |
| hsa-let-7bS | 1,00 | 2,98 | 0,34 | -1,09 | 0,08 | 0,21 | 0,00 | 0,031 | 0,39 |
| hsa-miR-147 | 32,91 | 83,30 | 0,40 | -0,93 | 0,00 | 0,02 | 0,00 | 0,031 | 0,30 |
| hsa-miR-582-5p | 22,39 | 38,77 | 0,58 | -0,55 | 0,04 | 0,13 | 0,00 | 0,031 | 0,36 |
| hsa-miR-550S | 370,14 | 334,57 | 1,11 | 0,10 | 0,01 | 0,06 | 0,00 | 0,031 | 0,67 |
| hsa-miR-1321 | 1,00 | 16,42 | 0,06 | -2,80 | 0,02 | 0,07 | 0,00 | 0,032 | 0,35 |
| hsa-miR-454S | 9,72 | 41,88 | 0,23 | -1,46 | 0,00 | 0,02 | 0,00 | 0,032 | 0,29 |
| hsa-miR-190 | 1,00 | 1,00 | 1,00 | 0,00 | 0,08 | 0,20 | 0,00 | 0,033 | 0,40 |
| hsa-miR-1257 | 1,00 | 24,02 | 0,04 | -3,18 | 0,01 | 0,05 | 0,00 | 0,035 | 0,33 |
| hsa-miR-1279 | 1,00 | 13,96 | 0,07 | -2,64 | 0,00 | 0,01 | 0,00 | 0,036 | 0,28 |
| hsa-miR-432 | 1,00 | 11,46 | 0,09 | -2,44 | 0,01 | 0,05 | 0,00 | 0,036 | 0,34 |
| hsa-miR-520d-3p | 1,00 | 8,53 | 0,12 | -2,14 | 0,01 | 0,06 | 0,00 | 0,036 | 0,35 |
| hsa-miR-630 | 20,28 | 59,54 | 0,34 | -1,08 | 0,01 | 0,05 | 0,00 | 0,036 | 0,33 |
| hsa-miR-125b-2S | 26,82 | 47,54 | 0,56 | -0,57 | 0,03 | 0,10 | 0,00 | 0,036 | 0,35 |
| hsa-miR-377S | 1,00 | 1,00 | 1,00 | 0,00 | 0,06 | 0,17 | 0,00 | 0,036 | 0,40 |
| hsa-miR-520f | 1,00 | 1,00 | 1,00 | 0,00 | 0,03 | 0,11 | 0,00 | 0,036 | 0,38 |
| hsa-miR-145S | 86,84 | 42,67 | 2,04 | 0,71 | 0,00 | 0,03 | 0,00 | 0,036 | 0,69 |
| hsa-miR-647 | 85,45 | 34,21 | 2,50 | 0,92 | 0,00 | 0,01 | 0,00 | 0,036 | 0,72 |
| hsa-miR-146b-3p | 54,44 | 23,65 | 2,30 | 0,83 | 0,00 | 0,03 | 0,01 | 0,038 | 0,69 |
| hsa-miR-154 | 1,00 | 1,00 | 1,00 | 0,00 | 0,03 | 0,10 | 0,01 | 0,040 | 0,37 |
| hsa-miR-26bS | 1,00 | 12,07 | 0,08 | -2,49 | 0,03 | 0,11 | 0,01 | 0,041 | 0,36 |
| hsa-miR-892a | 1,00 | 12,07 | 0,08 | -2,49 | 0,03 | 0,11 | 0,01 | 0,041 | 0,36 |
| hsa-miR-1249 | 82,77 | 144,13 | 0,57 | -0,55 | 0,02 | 0,07 | 0,01 | 0,041 | 0,34 |
| hsa-miR-624S | 23,81 | 39,16 | 0,61 | -0,50 | 0,11 | 0,27 | 0,01 | 0,041 | 0,40 |
| hsa-miR-636 | 162,06 | 114,56 | 1,41 | 0,35 | 0,00 | 0,02 | 0,01 | 0,042 | 0,70 |
| hsa-miR-187 | 8,11 | 34,21 | 0,24 | -1,44 | 0,05 | 0,14 | 0,01 | 0,043 | 0,37 |
| hsa-miR-221S | 127,27 | 60,81 | 2,09 | 0,74 | 0,00 | 0,01 | 0,01 | 0,044 | 0,72 |
| hsa-miR-1244 | 1,00 | 1,00 | 1,00 | 0,00 | 0,01 | 0,04 | 0,01 | 0,045 | 0,37 |
| hsa-miR-1305 | 116,50 | 87,96 | 1,32 | 0,28 | 0,01 | 0,04 | 0,01 | 0,045 | 0,68 |
| hsa-miR-1265 | 1,00 | 41,88 | 0,02 | -3,73 | 0,00 | 0,02 | 0,01 | 0,046 | 0,29 |
| hsa-miR-135aS | 1,00 | 16,95 | 0,06 | -2,83 | 0,03 | 0,11 | 0,01 | 0,046 | 0,36 |
| hsa-miR-942 | 8,11 | 45,56 | 0,18 | -1,73 | 0,01 | 0,06 | 0,01 | 0,046 | 0,33 |
| hsa-miR-1224-5p | 19,23 | 36,28 | 0,53 | -0,63 | 0,16 | 0,35 | 0,01 | 0,046 | 0,41 |
| hsa-miR-140-3p | 17675,49 | 20902,49 | 0,85 | -0,17 | 0,01 | 0,05 | 0,01 | 0,046 | 0,33 |
| hsa-miR-502-5p | 12,45 | 13,44 | 0,93 | -0,08 | 0,14 | 0,30 | 0,01 | 0,046 | 0,40 |
| hsa-miR-1266 | 120,21 | 100,18 | 1,20 | 0,18 | 0,01 | 0,06 | 0,01 | 0,046 | 0,67 |
| hsa-miR-886-3p | 1,00 | 16,14 | 0,06 | -2,78 | 0,02 | 0,07 | 0,01 | 0,047 | 0,35 |
| hsa-miR-330-5p | 8,11 | 28,23 | 0,29 | -1,25 | 0,01 | 0,04 | 0,01 | 0,047 | 0,32 |
| hsa-miR-1284 | 15,79 | 42,18 | 0,37 | -0,98 | 0,00 | 0,01 | 0,01 | 0,047 | 0,27 |
| hsa-miR-28-3p | 146,74 | 124,99 | 1,17 | 0,16 | 0,02 | 0,08 | 0,01 | 0,047 | 0,66 |
| hsa-miR-767-5p | 199,45 | 149,73 | 1,33 | 0,29 | 0,01 | 0,04 | 0,01 | 0,047 | 0,68 |
| hsa-miR-1233 | 119,96 | 83,67 | 1,43 | 0,36 | 0,00 | 0,02 | 0,01 | 0,049 | 0,70 |

Significant miRNAs normal vs. pancreatitis in blood:

|  | **median normal** | **median pancreatitis** | **qmedian** | **logqmedian** | **wmw_raw p** | **wmw_adj p** | **ttest_raw p** | **ttest_adj p** | **AUC** |
| --- | --- | --- | --- | --- | --- | --- | --- | --- | --- |
| hsa-miR-320a | 12702,71 | 6067,71 | 2,09 | 0,74 | 0,00 | 0,00 | 0,00 | 0,002 | 0,83 |
| hsa-miR-126S | 1,00 | 38,26 | 0,03 | -3,64 | 0,00 | 0,00 | 0,00 | 0,008 | 0,22 |
| hsa-miR-519b-5p | 163,75 | 85,99 | 1,90 | 0,64 | 0,00 | 0,00 | 0,00 | 0,008 | 0,78 |
| hsa-miR-378 | 266,92 | 135,80 | 1,97 | 0,68 | 0,00 | 0,00 | 0,00 | 0,008 | 0,80 |
| hsa-miR-130bS | 1,00 | 25,03 | 0,04 | -3,22 | 0,00 | 0,00 | 0,00 | 0,012 | 0,22 |
| hsa-miR-302d | 1,00 | 16,46 | 0,06 | -2,80 | 0,00 | 0,00 | 0,00 | 0,012 | 0,22 |
| hsa-miR-135a | 1,00 | 10,75 | 0,09 | -2,38 | 0,00 | 0,00 | 0,00 | 0,012 | 0,24 |
| hsa-miR-194S | 46,88 | 98,13 | 0,48 | -0,74 | 0,00 | 0,01 | 0,00 | 0,012 | 0,24 |
| hsa-miR-192S | 130,11 | 102,42 | 1,27 | 0,24 | 0,00 | 0,01 | 0,00 | 0,012 | 0,74 |
| hsa-miR-181a | 626,33 | 269,29 | 2,33 | 0,84 | 0,00 | 0,00 | 0,00 | 0,012 | 0,80 |
| hsa-miR-223S | 23,28 | 76,84 | 0,30 | -1,19 | 0,00 | 0,02 | 0,00 | 0,015 | 0,27 |
| hsa-miR-320d | 559,47 | 284,92 | 1,96 | 0,67 | 0,00 | 0,01 | 0,00 | 0,015 | 0,77 |
| hsa-miR-539 | 1,00 | 18,37 | 0,05 | -2,91 | 0,00 | 0,01 | 0,00 | 0,017 | 0,27 |
| hsa-miR-876-3p | 9,72 | 38,54 | 0,25 | -1,38 | 0,00 | 0,01 | 0,00 | 0,017 | 0,26 |
| hsa-miR-220c | 135,69 | 85,25 | 1,59 | 0,46 | 0,00 | 0,01 | 0,00 | 0,017 | 0,74 |
| hsa-miR-556-5p | 155,87 | 88,09 | 1,77 | 0,57 | 0,00 | 0,01 | 0,00 | 0,019 | 0,76 |
| hsa-miR-548d-5p | 1,00 | 27,83 | 0,04 | -3,33 | 0,00 | 0,01 | 0,00 | 0,023 | 0,26 |
| hsa-miR-142-3p | 18,37 | 87,72 | 0,21 | -1,56 | 0,00 | 0,02 | 0,00 | 0,023 | 0,28 |
| hsa-miR-608 | 64,91 | 155,81 | 0,42 | -0,88 | 0,00 | 0,00 | 0,00 | 0,023 | 0,20 |
| hsa-miR-518d-3p | 98,46 | 49,47 | 1,99 | 0,69 | 0,00 | 0,01 | 0,00 | 0,023 | 0,74 |
| hsa-miR-196b | 1,00 | 1,00 | 1,00 | 0,00 | 0,00 | 0,01 | 0,00 | 0,025 | 0,30 |
| hsa-miR-148a | 804,17 | 604,53 | 1,33 | 0,29 | 0,00 | 0,01 | 0,00 | 0,025 | 0,75 |
| hsa-miR-532-5p | 237,97 | 150,81 | 1,58 | 0,46 | 0,00 | 0,01 | 0,00 | 0,025 | 0,75 |
| hsa-let-7b | 966,00 | 412,79 | 2,34 | 0,85 | 0,00 | 0,01 | 0,00 | 0,025 | 0,75 |
| hsa-miR-556-3p | 1,00 | 18,90 | 0,05 | -2,94 | 0,00 | 0,00 | 0,00 | 0,026 | 0,22 |
| hsa-miR-767-5p | 199,45 | 119,70 | 1,67 | 0,51 | 0,00 | 0,03 | 0,00 | 0,027 | 0,71 |
| hsa-miR-491-3p | 102,50 | 39,92 | 2,57 | 0,94 | 0,00 | 0,01 | 0,00 | 0,027 | 0,75 |
| hsa-miR-454S | 9,72 | 65,37 | 0,15 | -1,91 | 0,00 | 0,02 | 0,00 | 0,029 | 0,28 |
| hsa-miR-196a | 1,00 | 2,32 | 0,43 | -0,84 | 0,00 | 0,01 | 0,00 | 0,029 | 0,29 |
| hsa-miR-181c | 161,66 | 109,71 | 1,47 | 0,39 | 0,00 | 0,03 | 0,00 | 0,029 | 0,70 |
| hsa-miR-518d-5p | 179,62 | 113,63 | 1,58 | 0,46 | 0,00 | 0,02 | 0,00 | 0,029 | 0,72 |
| hsa-miR-145S | 86,84 | 29,23 | 2,97 | 1,09 | 0,00 | 0,01 | 0,00 | 0,029 | 0,74 |
| hsa-miR-146b-3p | 54,44 | 11,41 | 4,77 | 1,56 | 0,00 | 0,01 | 0,00 | 0,029 | 0,77 |
| hsa-miR-548j | 1,00 | 1,00 | 1,00 | 0,00 | 0,01 | 0,07 | 0,00 | 0,030 | 0,35 |
| hsa-miR-590-5p | 192,66 | 122,09 | 1,58 | 0,46 | 0,00 | 0,02 | 0,00 | 0,030 | 0,73 |
| hsa-miR-188-5p | 134,42 | 97,47 | 1,38 | 0,32 | 0,00 | 0,02 | 0,00 | 0,032 | 0,72 |
| hsa-miR-369-5p | 15,63 | 40,85 | 0,38 | -0,96 | 0,01 | 0,06 | 0,00 | 0,034 | 0,32 |
| hsa-miR-518a-3p | 71,35 | 40,96 | 1,74 | 0,55 | 0,00 | 0,02 | 0,00 | 0,034 | 0,72 |
| hsa-miR-508-3p | 1,00 | 11,95 | 0,08 | -2,48 | 0,00 | 0,01 | 0,00 | 0,035 | 0,27 |
| hsa-miR-133b | 8,18 | 35,75 | 0,23 | -1,48 | 0,00 | 0,02 | 0,00 | 0,035 | 0,29 |
| hsa-miR-455-5p | 10,00 | 30,91 | 0,32 | -1,13 | 0,00 | 0,03 | 0,00 | 0,035 | 0,30 |
| hsa-miR-34a | 19,40 | 47,89 | 0,41 | -0,90 | 0,01 | 0,04 | 0,00 | 0,035 | 0,31 |
| hsa-miR-1226 | 55,63 | 93,70 | 0,59 | -0,52 | 0,00 | 0,02 | 0,00 | 0,035 | 0,27 |
| hsa-miR-1268 | 348,62 | 542,84 | 0,64 | -0,44 | 0,00 | 0,02 | 0,00 | 0,035 | 0,28 |
| hsa-miR-132S | 1,00 | 1,00 | 1,00 | 0,00 | 0,00 | 0,00 | 0,00 | 0,035 | 0,29 |
| hsa-miR-1266 | 120,21 | 84,60 | 1,42 | 0,35 | 0,01 | 0,04 | 0,00 | 0,035 | 0,70 |
| hsa-let-7i | 783,99 | 329,43 | 2,38 | 0,87 | 0,00 | 0,02 | 0,00 | 0,035 | 0,72 |
| hsa-miR-1290 | 1,00 | 1,00 | 1,00 | 0,00 | 0,02 | 0,09 | 0,00 | 0,036 | 0,36 |
| hsa-miR-601 | 1,00 | 31,96 | 0,03 | -3,46 | 0,00 | 0,01 | 0,00 | 0,037 | 0,26 |
| hsa-miR-1257 | 1,00 | 23,47 | 0,04 | -3,16 | 0,01 | 0,04 | 0,00 | 0,037 | 0,31 |
| hsa-miR-190 | 1,00 | 3,91 | 0,26 | -1,36 | 0,00 | 0,03 | 0,00 | 0,037 | 0,31 |
| hsa-miR-27bS | 70,14 | 29,81 | 2,35 | 0,86 | 0,00 | 0,02 | 0,00 | 0,037 | 0,73 |
| hsa-miR-1279 | 1,00 | 9,96 | 0,10 | -2,30 | 0,00 | 0,02 | 0,00 | 0,040 | 0,31 |
| hsa-miR-299-3p | 1,00 | 5,42 | 0,18 | -1,69 | 0,12 | 0,28 | 0,00 | 0,040 | 0,40 |
| hsa-miR-581 | 7,61 | 23,31 | 0,33 | -1,12 | 0,02 | 0,10 | 0,00 | 0,040 | 0,35 |
| hsa-miR-520c-3p | 1,00 | 1,00 | 1,00 | 0,00 | 0,00 | 0,03 | 0,00 | 0,040 | 0,34 |
| hsa-miR-210 | 708,07 | 425,35 | 1,66 | 0,51 | 0,00 | 0,02 | 0,00 | 0,040 | 0,72 |
| hsa-miR-204 | 1,00 | 7,13 | 0,14 | -1,96 | 0,00 | 0,01 | 0,00 | 0,041 | 0,29 |
| hsa-miR-135aS | 1,00 | 21,57 | 0,05 | -3,07 | 0,02 | 0,08 | 0,00 | 0,043 | 0,34 |
| hsa-miR-324-3p | 587,21 | 436,16 | 1,35 | 0,30 | 0,00 | 0,03 | 0,00 | 0,043 | 0,71 |
| hsa-miR-199b-5p | 1,00 | 19,64 | 0,05 | -2,98 | 0,01 | 0,06 | 0,00 | 0,044 | 0,33 |
| hsa-miR-513c | 1,00 | 17,04 | 0,06 | -2,84 | 0,01 | 0,04 | 0,00 | 0,044 | 0,31 |
| hsa-miR-1208 | 107,91 | 83,60 | 1,29 | 0,26 | 0,00 | 0,02 | 0,00 | 0,044 | 0,72 |
| hsa-miR-1826 | 217,48 | 119,04 | 1,83 | 0,60 | 0,00 | 0,00 | 0,00 | 0,044 | 0,81 |
| hsa-miR-519aS | 168,00 | 89,92 | 1,87 | 0,63 | 0,00 | 0,02 | 0,00 | 0,044 | 0,72 |
| hsa-miR-1278 | 70,14 | 34,85 | 2,01 | 0,70 | 0,00 | 0,02 | 0,00 | 0,044 | 0,72 |
| hsa-miR-885-5p | 15,63 | 52,68 | 0,30 | -1,22 | 0,00 | 0,01 | 0,00 | 0,045 | 0,25 |
| hsa-miR-23b | 2872,95 | 3467,72 | 0,83 | -0,19 | 0,01 | 0,08 | 0,00 | 0,045 | 0,33 |
| hsa-miR-21S | 119,40 | 77,49 | 1,54 | 0,43 | 0,01 | 0,04 | 0,00 | 0,045 | 0,69 |
| hsa-miR-651 | 1,00 | 25,61 | 0,04 | -3,24 | 0,00 | 0,03 | 0,00 | 0,048 | 0,31 |
| hsa-miR-490-5p | 147,86 | 106,09 | 1,39 | 0,33 | 0,00 | 0,02 | 0,00 | 0,048 | 0,73 |
| hsa-miR-106b | 14207,24 | 8807,11 | 1,61 | 0,48 | 0,00 | 0,03 | 0,00 | 0,048 | 0,70 |

Results for miRNAs in the comparison cancer vs. pancreatitis in blood; none of the molecules showed a significant variation:

|  | **median cancer** | **median pancreatitis** | **qmedian** | **logqmedian** | **wmw_raw p** | **wmw_adj p** | **ttest_raw p** | **ttest_adj p** | **AUC** |
| --- | --- | --- | --- | --- | --- | --- | --- | --- | --- |
| hsa-let-7a | 1135,39 | 1031,43 | 1,10 | 0,10 | 0,82 | 1,00 | 0,70 | 1,00 | 0,48 |
| hsa-let-7aS | 1,00 | 18,90 | 0,05 | -2,94 | 0,58 | 1,00 | 0,62 | 1,00 | 0,47 |
| hsa-let-7b | 587,21 | 412,79 | 1,42 | 0,35 | 0,20 | 1,00 | 0,44 | 1,00 | 0,58 |
| hsa-let-7bS | 2,98 | 17,82 | 0,17 | -1,79 | 0,75 | 1,00 | 0,62 | 1,00 | 0,48 |
| hsa-let-7c | 280,18 | 395,92 | 0,71 | -0,35 | 0,73 | 1,00 | 0,69 | 1,00 | 0,48 |
| hsa-let-7cS | 12,07 | 14,61 | 0,83 | -0,19 | 0,86 | 1,00 | 0,54 | 1,00 | 0,49 |
| hsa-let-7d | 2746,46 | 2434,54 | 1,13 | 0,12 | 0,24 | 1,00 | 0,38 | 1,00 | 0,58 |
| hsa-let-7dS | 43,25 | 51,83 | 0,83 | -0,18 | 0,77 | 1,00 | 0,32 | 1,00 | 0,48 |
| hsa-let-7e | 168,89 | 188,18 | 0,90 | -0,11 | 0,83 | 1,00 | 0,88 | 1,00 | 0,49 |
| hsa-let-7eS | 1,00 | 10,42 | 0,10 | -2,34 | 0,64 | 1,00 | 0,78 | 1,00 | 0,47 |
| hsa-let-7f | 479,74 | 521,27 | 0,92 | -0,08 | 0,76 | 1,00 | 0,91 | 1,00 | 0,48 |
| hsa-let-7f-1S | 12,25 | 11,61 | 1,05 | 0,05 | 0,73 | 1,00 | 0,60 | 1,00 | 0,52 |
| hsa-let-7f-2S | 1,00 | 8,46 | 0,12 | -2,13 | 0,04 | 1,00 | 0,17 | 1,00 | 0,38 |
| hsa-let-7g | 433,88 | 500,40 | 0,87 | -0,14 | 0,88 | 1,00 | 0,62 | 1,00 | 0,49 |
| hsa-let-7gS | 154,29 | 137,80 | 1,12 | 0,11 | 0,69 | 1,00 | 0,52 | 1,00 | 0,53 |
| hsa-let-7i | 372,43 | 329,43 | 1,13 | 0,12 | 0,84 | 1,00 | 0,92 | 1,00 | 0,49 |
| hsa-let-7iS | 191,96 | 162,74 | 1,18 | 0,17 | 0,70 | 1,00 | 0,82 | 1,00 | 0,53 |
| hsa-miR-1 | 20,56 | 19,07 | 1,08 | 0,08 | 0,68 | 1,00 | 0,65 | 1,00 | 0,47 |
| hsa-miR-100 | 156,29 | 113,29 | 1,38 | 0,32 | 0,42 | 1,00 | 0,98 | 1,00 | 0,55 |
| hsa-miR-100S | 30,25 | 47,51 | 0,64 | -0,45 | 0,97 | 1,00 | 0,93 | 1,00 | 0,50 |
| hsa-miR-101 | 615,03 | 638,91 | 0,96 | -0,04 | 0,92 | 1,00 | 0,84 | 1,00 | 0,49 |
| hsa-miR-101S | 122,08 | 110,67 | 1,10 | 0,10 | 0,78 | 1,00 | 0,36 | 1,00 | 0,52 |
| hsa-miR-103 | 7079,20 | 8274,36 | 0,86 | -0,16 | 0,12 | 1,00 | 0,09 | 1,00 | 0,40 |
| hsa-miR-103-as | 162,06 | 164,53 | 0,98 | -0,02 | 0,64 | 1,00 | 0,91 | 1,00 | 0,47 |
| hsa-miR-105 | 50,68 | 35,93 | 1,41 | 0,34 | 0,56 | 1,00 | 0,24 | 1,00 | 0,54 |
| hsa-miR-105S | 1,00 | 31,46 | 0,03 | -3,45 | 0,01 | 1,00 | 0,24 | 1,00 | 0,34 |
| hsa-miR-106a | 7477,04 | 7477,04 | 1,00 | 0,00 | 0,64 | 1,00 | 1,00 | 1,00 | 0,53 |
| hsa-miR-106aS | 142,69 | 136,50 | 1,05 | 0,04 | 0,61 | 1,00 | 0,43 | 1,00 | 0,53 |
| hsa-miR-106b | 7477,04 | 8807,11 | 0,85 | -0,16 | 0,33 | 1,00 | 0,18 | 1,00 | 0,44 |
| hsa-miR-106bS | 161,66 | 179,16 | 0,90 | -0,10 | 0,57 | 1,00 | 0,58 | 1,00 | 0,46 |
| hsa-miR-107 | 1799,95 | 1956,56 | 0,92 | -0,08 | 0,28 | 1,00 | 0,46 | 1,00 | 0,43 |
| hsa-miR-10a | 21,80 | 28,99 | 0,75 | -0,28 | 0,39 | 1,00 | 0,67 | 1,00 | 0,45 |
| hsa-miR-10aS | 38,00 | 48,53 | 0,78 | -0,24 | 0,09 | 1,00 | 0,32 | 1,00 | 0,39 |
| hsa-miR-10b | 62,20 | 77,02 | 0,81 | -0,21 | 0,76 | 1,00 | 0,74 | 1,00 | 0,48 |
| hsa-miR-10bS | 52,19 | 58,79 | 0,89 | -0,12 | 0,34 | 1,00 | 0,32 | 1,00 | 0,44 |
| hsa-miR-1178 | 34,68 | 28,67 | 1,21 | 0,19 | 0,71 | 1,00 | 0,77 | 1,00 | 0,52 |
| hsa-miR-1179 | 15,09 | 32,74 | 0,46 | -0,77 | 0,57 | 1,00 | 0,20 | 1,00 | 0,46 |
| hsa-miR-1180 | 59,54 | 72,84 | 0,82 | -0,20 | 0,71 | 1,00 | 0,35 | 1,00 | 0,48 |
| hsa-miR-1181 | 211,34 | 195,74 | 1,08 | 0,08 | 0,88 | 1,00 | 0,60 | 1,00 | 0,49 |
| hsa-miR-1182 | 16,82 | 9,45 | 1,78 | 0,58 | 0,41 | 1,00 | 0,69 | 1,00 | 0,55 |
| hsa-miR-1183 | 177,11 | 145,56 | 1,22 | 0,20 | 0,28 | 1,00 | 0,30 | 1,00 | 0,57 |
| hsa-miR-1184 | 168,89 | 156,06 | 1,08 | 0,08 | 0,31 | 1,00 | 0,69 | 1,00 | 0,57 |
| hsa-miR-1185 | 10,88 | 14,63 | 0,74 | -0,30 | 0,49 | 1,00 | 0,47 | 1,00 | 0,46 |
| hsa-miR-1197 | 49,89 | 49,51 | 1,01 | 0,01 | 0,91 | 1,00 | 0,82 | 1,00 | 0,49 |
| hsa-miR-1200 | 69,82 | 71,95 | 0,97 | -0,03 | 0,38 | 1,00 | 0,35 | 1,00 | 0,44 |
| hsa-miR-1201 | 49,89 | 70,78 | 0,70 | -0,35 | 0,17 | 1,00 | 0,14 | 1,00 | 0,41 |
| hsa-miR-1202 | 237,03 | 237,40 | 1,00 | 0,00 | 0,46 | 1,00 | 0,87 | 1,00 | 0,45 |
| hsa-miR-1203 | 168,89 | 167,68 | 1,01 | 0,01 | 0,91 | 1,00 | 0,64 | 1,00 | 0,49 |
| hsa-miR-1204 | 1,00 | 14,96 | 0,07 | -2,71 | 0,19 | 1,00 | 0,53 | 1,00 | 0,42 |
| hsa-miR-1205 | 57,16 | 25,59 | 2,23 | 0,80 | 0,14 | 1,00 | 0,15 | 1,00 | 0,60 |
| hsa-miR-1206 | 32,26 | 51,61 | 0,63 | -0,47 | 0,42 | 1,00 | 0,59 | 1,00 | 0,45 |
| hsa-miR-1207-3p | 24,02 | 23,40 | 1,03 | 0,03 | 0,89 | 1,00 | 0,57 | 1,00 | 0,49 |
| hsa-miR-1207-5p | 334,57 | 313,92 | 1,07 | 0,06 | 0,27 | 1,00 | 0,22 | 1,00 | 0,57 |
| hsa-miR-1208 | 82,02 | 83,60 | 0,98 | -0,02 | 0,56 | 1,00 | 0,24 | 1,00 | 0,54 |
| hsa-miR-122 | 49,65 | 43,19 | 1,15 | 0,14 | 0,24 | 1,00 | 0,14 | 1,00 | 0,58 |
| hsa-miR-1224-3p | 169,24 | 160,97 | 1,05 | 0,05 | 0,74 | 1,00 | 0,91 | 1,00 | 0,52 |
| hsa-miR-1224-5p | 36,28 | 17,65 | 2,06 | 0,72 | 0,82 | 1,00 | 0,85 | 1,00 | 0,51 |
| hsa-miR-1225-3p | 123,48 | 177,55 | 0,70 | -0,36 | 0,11 | 1,00 | 0,04 | 1,00 | 0,40 |
| hsa-miR-1225-5p | 74,56 | 74,78 | 1,00 | 0,00 | 0,40 | 1,00 | 0,54 | 1,00 | 0,44 |
| hsa-miR-1226 | 88,91 | 93,70 | 0,95 | -0,05 | 0,35 | 1,00 | 0,25 | 1,00 | 0,44 |
| hsa-miR-1226S | 142,29 | 160,42 | 0,89 | -0,12 | 0,27 | 1,00 | 0,23 | 1,00 | 0,43 |
| hsa-miR-1227 | 58,98 | 45,30 | 1,30 | 0,26 | 0,36 | 1,00 | 0,27 | 1,00 | 0,56 |
| hsa-miR-1228 | 458,61 | 371,14 | 1,24 | 0,21 | 0,48 | 1,00 | 0,45 | 1,00 | 0,55 |
| hsa-miR-1228S | 1091,66 | 917,38 | 1,19 | 0,17 | 0,46 | 1,00 | 0,12 | 1,00 | 0,55 |
| hsa-miR-1229 | 198,36 | 210,29 | 0,94 | -0,06 | 0,71 | 1,00 | 0,57 | 1,00 | 0,48 |
| hsa-miR-122S | 74,02 | 64,99 | 1,14 | 0,13 | 1,00 | 1,00 | 0,94 | 1,00 | 0,50 |
| hsa-miR-1231 | 89,09 | 59,65 | 1,49 | 0,40 | 0,10 | 1,00 | 0,12 | 1,00 | 0,61 |
| hsa-miR-1233 | 83,67 | 100,24 | 0,83 | -0,18 | 0,44 | 1,00 | 0,62 | 1,00 | 0,45 |
| hsa-miR-1234 | 529,41 | 577,89 | 0,92 | -0,09 | 0,82 | 1,00 | 0,50 | 1,00 | 0,52 |
| hsa-miR-1236 | 30,25 | 43,32 | 0,70 | -0,36 | 0,97 | 1,00 | 0,93 | 1,00 | 0,50 |
| hsa-miR-1237 | 101,33 | 122,76 | 0,83 | -0,19 | 0,72 | 1,00 | 0,61 | 1,00 | 0,48 |
| hsa-miR-1238 | 16,14 | 15,19 | 1,06 | 0,06 | 0,93 | 1,00 | 0,66 | 1,00 | 0,49 |
| hsa-miR-124 | 107,51 | 114,62 | 0,94 | -0,06 | 0,94 | 1,00 | 0,59 | 1,00 | 0,50 |
| hsa-miR-1243 | 37,75 | 52,67 | 0,72 | -0,33 | 0,33 | 1,00 | 0,35 | 1,00 | 0,44 |
| hsa-miR-1244 | 1,00 | 1,00 | 1,00 | 0,00 | 0,73 | 1,00 | 0,99 | 1,00 | 0,48 |
| hsa-miR-1245 | 10,25 | 28,61 | 0,36 | -1,03 | 0,27 | 1,00 | 0,94 | 1,00 | 0,43 |
| hsa-miR-1246 | 12,07 | 2,32 | 5,20 | 1,65 | 0,78 | 1,00 | 0,80 | 1,00 | 0,52 |
| hsa-miR-1247 | 69,68 | 74,60 | 0,93 | -0,07 | 0,86 | 1,00 | 0,80 | 1,00 | 0,49 |
| hsa-miR-1248 | 42,11 | 38,90 | 1,08 | 0,08 | 0,58 | 1,00 | 0,98 | 1,00 | 0,46 |
| hsa-miR-1249 | 144,13 | 150,21 | 0,96 | -0,04 | 0,78 | 1,00 | 0,60 | 1,00 | 0,48 |
| hsa-miR-124S | 87,96 | 92,05 | 0,96 | -0,05 | 0,92 | 1,00 | 0,50 | 1,00 | 0,49 |
| hsa-miR-1250 | 106,16 | 86,05 | 1,23 | 0,21 | 0,51 | 1,00 | 0,31 | 1,00 | 0,54 |
| hsa-miR-1251 | 129,39 | 148,13 | 0,87 | -0,14 | 0,11 | 1,00 | 0,18 | 1,00 | 0,40 |
| hsa-miR-1252 | 17,65 | 28,03 | 0,63 | -0,46 | 0,27 | 1,00 | 0,13 | 1,00 | 0,43 |
| hsa-miR-1253 | 83,67 | 81,38 | 1,03 | 0,03 | 0,70 | 1,00 | 0,80 | 1,00 | 0,53 |
| hsa-miR-1254 | 141,10 | 140,32 | 1,01 | 0,01 | 0,40 | 1,00 | 0,52 | 1,00 | 0,45 |
| hsa-miR-1255a | 52,98 | 65,96 | 0,80 | -0,22 | 0,70 | 1,00 | 0,41 | 1,00 | 0,48 |
| hsa-miR-1255b | 70,53 | 78,60 | 0,90 | -0,11 | 0,57 | 1,00 | 0,94 | 1,00 | 0,46 |
| hsa-miR-1256 | 34,21 | 35,59 | 0,96 | -0,04 | 0,68 | 1,00 | 0,92 | 1,00 | 0,53 |
| hsa-miR-1257 | 24,02 | 23,47 | 1,02 | 0,02 | 0,74 | 1,00 | 0,74 | 1,00 | 0,48 |
| hsa-miR-1258 | 1,00 | 1,00 | 1,00 | 0,00 | 0,49 | 1,00 | 0,88 | 1,00 | 0,54 |
| hsa-miR-1259 | 40,89 | 33,34 | 1,23 | 0,20 | 0,75 | 1,00 | 0,66 | 1,00 | 0,52 |
| hsa-miR-125a-3p | 36,39 | 11,22 | 3,24 | 1,18 | 0,18 | 1,00 | 0,86 | 1,00 | 0,59 |
| hsa-miR-125a-5p | 192,39 | 171,19 | 1,12 | 0,12 | 0,66 | 1,00 | 0,85 | 1,00 | 0,53 |
| hsa-miR-125b | 521,37 | 313,68 | 1,66 | 0,51 | 0,24 | 1,00 | 0,64 | 1,00 | 0,58 |
| hsa-miR-125b-1S | 14,07 | 1,00 | 14,07 | 2,64 | 0,46 | 1,00 | 0,43 | 1,00 | 0,55 |
| hsa-miR-125b-2S | 47,54 | 55,18 | 0,86 | -0,15 | 0,78 | 1,00 | 0,57 | 1,00 | 0,48 |
| hsa-miR-126 | 1907,68 | 2252,16 | 0,85 | -0,17 | 0,41 | 1,00 | 0,55 | 1,00 | 0,45 |
| hsa-miR-1260 | 2523,52 | 2580,36 | 0,98 | -0,02 | 0,97 | 1,00 | 0,98 | 1,00 | 0,50 |
| hsa-miR-1261 | 2,88 | 1,00 | 2,88 | 1,06 | 0,70 | 1,00 | 0,75 | 1,00 | 0,52 |
| hsa-miR-1262 | 4,42 | 5,16 | 0,86 | -0,15 | 0,77 | 1,00 | 0,09 | 1,00 | 0,52 |
| hsa-miR-1263 | 74,58 | 87,41 | 0,85 | -0,16 | 0,38 | 1,00 | 0,32 | 1,00 | 0,44 |
| hsa-miR-1264 | 23,72 | 29,71 | 0,80 | -0,23 | 0,87 | 1,00 | 0,71 | 1,00 | 0,49 |
| hsa-miR-1265 | 41,88 | 26,77 | 1,56 | 0,45 | 0,21 | 1,00 | 0,71 | 1,00 | 0,58 |
| hsa-miR-1266 | 100,18 | 84,60 | 1,18 | 0,17 | 0,87 | 1,00 | 0,61 | 1,00 | 0,51 |
| hsa-miR-1267 | 44,42 | 41,67 | 1,07 | 0,06 | 0,92 | 1,00 | 0,55 | 1,00 | 0,51 |
| hsa-miR-1268 | 559,47 | 542,84 | 1,03 | 0,03 | 0,51 | 1,00 | 0,51 | 1,00 | 0,54 |
| hsa-miR-1269 | 45,26 | 40,50 | 1,12 | 0,11 | 0,39 | 1,00 | 0,28 | 1,00 | 0,56 |
| hsa-miR-126S | 34,53 | 38,26 | 0,90 | -0,10 | 0,74 | 1,00 | 0,50 | 1,00 | 0,52 |
| hsa-miR-127-3p | 40,53 | 32,81 | 1,24 | 0,21 | 0,60 | 1,00 | 0,47 | 1,00 | 0,53 |
| hsa-miR-127-5p | 117,68 | 106,30 | 1,11 | 0,10 | 0,63 | 1,00 | 0,98 | 1,00 | 0,53 |
| hsa-miR-1270 | 18,77 | 22,63 | 0,83 | -0,19 | 0,60 | 1,00 | 0,95 | 1,00 | 0,47 |
| hsa-miR-1271 | 208,84 | 228,26 | 0,91 | -0,09 | 0,53 | 1,00 | 0,55 | 1,00 | 0,46 |
| hsa-miR-1272 | 153,23 | 139,38 | 1,10 | 0,09 | 0,70 | 1,00 | 0,54 | 1,00 | 0,53 |
| hsa-miR-1273 | 129,59 | 142,49 | 0,91 | -0,09 | 0,68 | 1,00 | 0,43 | 1,00 | 0,47 |
| hsa-miR-1274a | 109,07 | 84,10 | 1,30 | 0,26 | 0,23 | 1,00 | 0,29 | 1,00 | 0,58 |
| hsa-miR-1274b | 639,04 | 627,03 | 1,02 | 0,02 | 0,76 | 1,00 | 0,63 | 1,00 | 0,52 |
| hsa-miR-1275 | 83,67 | 117,68 | 0,71 | -0,34 | 0,34 | 1,00 | 0,89 | 1,00 | 0,44 |
| hsa-miR-1276 | 46,77 | 52,58 | 0,89 | -0,12 | 0,98 | 1,00 | 0,78 | 1,00 | 0,50 |
| hsa-miR-1277 | 45,26 | 25,89 | 1,75 | 0,56 | 0,42 | 1,00 | 0,94 | 1,00 | 0,55 |
| hsa-miR-1278 | 45,56 | 34,85 | 1,31 | 0,27 | 0,67 | 1,00 | 0,54 | 1,00 | 0,53 |
| hsa-miR-1279 | 13,96 | 9,96 | 1,40 | 0,34 | 0,69 | 1,00 | 0,37 | 1,00 | 0,53 |
| hsa-miR-128 | 421,38 | 451,29 | 0,93 | -0,07 | 1,00 | 1,00 | 1,00 | 1,00 | 0,50 |
| hsa-miR-1280 | 3467,72 | 3835,54 | 0,90 | -0,10 | 0,51 | 1,00 | 0,71 | 1,00 | 0,46 |
| hsa-miR-1281 | 123,76 | 148,13 | 0,84 | -0,18 | 0,50 | 1,00 | 0,73 | 1,00 | 0,46 |
| hsa-miR-1282 | 1,00 | 1,00 | 1,00 | 0,00 | 0,61 | 1,00 | 0,28 | 1,00 | 0,53 |
| hsa-miR-1283 | 83,11 | 95,03 | 0,87 | -0,13 | 0,35 | 1,00 | 0,42 | 1,00 | 0,44 |
| hsa-miR-1284 | 42,18 | 42,09 | 1,00 | 0,00 | 0,82 | 1,00 | 0,56 | 1,00 | 0,49 |
| hsa-miR-1285 | 202,87 | 226,62 | 0,90 | -0,11 | 0,33 | 1,00 | 0,40 | 1,00 | 0,44 |
| hsa-miR-1286 | 93,16 | 114,83 | 0,81 | -0,21 | 0,68 | 1,00 | 0,71 | 1,00 | 0,47 |
| hsa-miR-1287 | 73,72 | 63,47 | 1,16 | 0,15 | 0,47 | 1,00 | 0,56 | 1,00 | 0,55 |
| hsa-miR-1288 | 87,96 | 84,89 | 1,04 | 0,04 | 0,82 | 1,00 | 0,83 | 1,00 | 0,51 |
| hsa-miR-1289 | 101,33 | 105,62 | 0,96 | -0,04 | 0,84 | 1,00 | 0,84 | 1,00 | 0,51 |
| hsa-miR-129-3p | 93,51 | 89,39 | 1,05 | 0,05 | 0,53 | 1,00 | 0,52 | 1,00 | 0,46 |
| hsa-miR-129-5p | 44,42 | 60,83 | 0,73 | -0,31 | 0,66 | 1,00 | 0,98 | 1,00 | 0,47 |
| hsa-miR-1290 | 1,00 | 1,00 | 1,00 | 0,00 | 0,62 | 1,00 | 0,98 | 1,00 | 0,47 |
| hsa-miR-1291 | 144,13 | 119,98 | 1,20 | 0,18 | 0,86 | 1,00 | 0,67 | 1,00 | 0,51 |
| hsa-miR-1292 | 67,30 | 39,45 | 1,71 | 0,53 | 0,37 | 1,00 | 0,43 | 1,00 | 0,56 |
| hsa-miR-1293 | 58,84 | 54,95 | 1,07 | 0,07 | 0,93 | 1,00 | 0,59 | 1,00 | 0,49 |
| hsa-miR-1294 | 17,02 | 11,82 | 1,44 | 0,36 | 0,63 | 1,00 | 0,66 | 1,00 | 0,53 |
| hsa-miR-1295 | 87,91 | 100,48 | 0,87 | -0,13 | 0,15 | 1,00 | 0,11 | 1,00 | 0,41 |
| hsa-miR-1296 | 35,35 | 52,88 | 0,67 | -0,40 | 0,49 | 1,00 | 0,67 | 1,00 | 0,45 |
| hsa-miR-1297 | 37,75 | 25,72 | 1,47 | 0,38 | 0,30 | 1,00 | 0,60 | 1,00 | 0,57 |
| hsa-miR-1298 | 52,53 | 54,12 | 0,97 | -0,03 | 0,57 | 1,00 | 0,44 | 1,00 | 0,46 |
| hsa-miR-1299 | 24,02 | 40,93 | 0,59 | -0,53 | 0,90 | 1,00 | 0,72 | 1,00 | 0,49 |
| hsa-miR-129S | 57,16 | 52,65 | 1,09 | 0,08 | 0,65 | 1,00 | 0,96 | 1,00 | 0,53 |
| hsa-miR-1301 | 130,57 | 147,13 | 0,89 | -0,12 | 0,83 | 1,00 | 0,59 | 1,00 | 0,51 |
| hsa-miR-1302 | 27,33 | 34,85 | 0,78 | -0,24 | 0,46 | 1,00 | 0,69 | 1,00 | 0,45 |
| hsa-miR-1303 | 58,98 | 41,30 | 1,43 | 0,36 | 0,05 | 1,00 | 0,43 | 1,00 | 0,63 |
| hsa-miR-1304 | 62,20 | 77,77 | 0,80 | -0,22 | 0,27 | 1,00 | 0,41 | 1,00 | 0,43 |
| hsa-miR-1305 | 87,96 | 70,71 | 1,24 | 0,22 | 0,86 | 1,00 | 0,42 | 1,00 | 0,51 |
| hsa-miR-1306 | 99,14 | 57,86 | 1,71 | 0,54 | 0,42 | 1,00 | 0,94 | 1,00 | 0,55 |
| hsa-miR-1307 | 77,19 | 69,44 | 1,11 | 0,11 | 0,30 | 1,00 | 0,35 | 1,00 | 0,57 |
| hsa-miR-1308 | 80,88 | 69,87 | 1,16 | 0,15 | 0,39 | 1,00 | 0,40 | 1,00 | 0,56 |
| hsa-miR-130a | 982,48 | 1135,39 | 0,87 | -0,14 | 0,17 | 1,00 | 0,39 | 1,00 | 0,41 |
| hsa-miR-130aS | 99,86 | 104,41 | 0,96 | -0,04 | 0,96 | 1,00 | 0,57 | 1,00 | 0,50 |
| hsa-miR-130b | 828,12 | 890,08 | 0,93 | -0,07 | 0,49 | 1,00 | 0,61 | 1,00 | 0,46 |
| hsa-miR-130bS | 16,95 | 25,03 | 0,68 | -0,39 | 0,46 | 1,00 | 0,59 | 1,00 | 0,45 |
| hsa-miR-132 | 103,98 | 129,10 | 0,81 | -0,22 | 0,19 | 1,00 | 0,22 | 1,00 | 0,42 |
| hsa-miR-1321 | 16,42 | 12,12 | 1,35 | 0,30 | 0,69 | 1,00 | 0,85 | 1,00 | 0,53 |
| hsa-miR-1322 | 125,65 | 94,07 | 1,34 | 0,29 | 0,37 | 1,00 | 0,54 | 1,00 | 0,56 |
| hsa-miR-1323 | 36,39 | 26,04 | 1,40 | 0,33 | 0,19 | 1,00 | 0,53 | 1,00 | 0,59 |
| hsa-miR-1324 | 140,87 | 128,62 | 1,10 | 0,09 | 0,99 | 1,00 | 0,35 | 1,00 | 0,50 |
| hsa-miR-132S | 8,11 | 1,00 | 8,11 | 2,09 | 0,26 | 1,00 | 0,17 | 1,00 | 0,57 |
| hsa-miR-133a | 87,91 | 88,39 | 0,99 | -0,01 | 0,56 | 1,00 | 0,26 | 1,00 | 0,46 |
| hsa-miR-133b | 30,14 | 35,75 | 0,84 | -0,17 | 0,54 | 1,00 | 0,89 | 1,00 | 0,46 |
| hsa-miR-134 | 23,65 | 56,04 | 0,42 | -0,86 | 0,15 | 1,00 | 0,68 | 1,00 | 0,41 |
| hsa-miR-135a | 1,00 | 10,75 | 0,09 | -2,38 | 0,86 | 1,00 | 0,85 | 1,00 | 0,49 |
| hsa-miR-135aS | 16,95 | 21,57 | 0,79 | -0,24 | 0,73 | 1,00 | 0,76 | 1,00 | 0,48 |
| hsa-miR-135b | 24,02 | 8,32 | 2,89 | 1,06 | 0,31 | 1,00 | 0,35 | 1,00 | 0,56 |
| hsa-miR-135bS | 52,19 | 51,89 | 1,01 | 0,01 | 0,71 | 1,00 | 0,71 | 1,00 | 0,48 |
| hsa-miR-136 | 1,00 | 22,54 | 0,04 | -3,12 | 0,18 | 1,00 | 0,35 | 1,00 | 0,42 |
| hsa-miR-136S | 56,35 | 48,46 | 1,16 | 0,15 | 0,80 | 1,00 | 0,62 | 1,00 | 0,52 |
| hsa-miR-137 | 62,20 | 66,65 | 0,93 | -0,07 | 0,89 | 1,00 | 0,83 | 1,00 | 0,49 |
| hsa-miR-138 | 57,47 | 51,96 | 1,11 | 0,10 | 0,50 | 1,00 | 0,36 | 1,00 | 0,54 |
| hsa-miR-138-1S | 110,53 | 115,20 | 0,96 | -0,04 | 0,75 | 1,00 | 0,51 | 1,00 | 0,52 |
| hsa-miR-138-2S | 45,26 | 31,11 | 1,46 | 0,38 | 0,63 | 1,00 | 0,82 | 1,00 | 0,53 |
| hsa-miR-139-3p | 87,58 | 79,26 | 1,10 | 0,10 | 0,53 | 1,00 | 0,50 | 1,00 | 0,54 |
| hsa-miR-139-5p | 164,13 | 164,37 | 1,00 | 0,00 | 0,62 | 1,00 | 0,54 | 1,00 | 0,53 |
| hsa-miR-140-3p | 20902,49 | 20902,49 | 1,00 | 0,00 | 0,57 | 1,00 | 0,86 | 1,00 | 0,54 |
| hsa-miR-140-5p | 9,93 | 29,30 | 0,34 | -1,08 | 0,40 | 1,00 | 0,65 | 1,00 | 0,45 |
| hsa-miR-141 | 41,88 | 34,45 | 1,22 | 0,20 | 0,91 | 1,00 | 0,62 | 1,00 | 0,51 |
| hsa-miR-141S | 109,07 | 109,20 | 1,00 | 0,00 | 0,55 | 1,00 | 0,74 | 1,00 | 0,54 |
| hsa-miR-142-3p | 79,49 | 87,72 | 0,91 | -0,10 | 0,76 | 1,00 | 0,62 | 1,00 | 0,48 |
| hsa-miR-142-5p | 693,08 | 729,18 | 0,95 | -0,05 | 0,46 | 1,00 | 0,60 | 1,00 | 0,45 |
| hsa-miR-143 | 204,77 | 219,81 | 0,93 | -0,07 | 0,13 | 1,00 | 0,92 | 1,00 | 0,40 |
| hsa-miR-143S | 105,02 | 144,85 | 0,73 | -0,32 | 0,07 | 1,00 | 0,33 | 1,00 | 0,38 |
| hsa-miR-144 | 2637,21 | 2295,07 | 1,15 | 0,14 | 0,60 | 1,00 | 0,82 | 1,00 | 0,53 |
| hsa-miR-144S | 587,21 | 574,46 | 1,02 | 0,02 | 0,98 | 1,00 | 0,94 | 1,00 | 0,50 |
| hsa-miR-145 | 192,66 | 136,89 | 1,41 | 0,34 | 0,28 | 1,00 | 0,29 | 1,00 | 0,57 |
| hsa-miR-145S | 42,67 | 29,23 | 1,46 | 0,38 | 0,51 | 1,00 | 0,52 | 1,00 | 0,54 |
| hsa-miR-1468 | 1,00 | 1,00 | 1,00 | 0,00 | 0,71 | 1,00 | 0,49 | 1,00 | 0,48 |
| hsa-miR-1469 | 159,48 | 173,81 | 0,92 | -0,09 | 0,96 | 1,00 | 0,69 | 1,00 | 0,50 |
| hsa-miR-146a | 119,72 | 129,92 | 0,92 | -0,08 | 0,46 | 1,00 | 0,39 | 1,00 | 0,45 |
| hsa-miR-146aS | 96,00 | 92,59 | 1,04 | 0,04 | 0,90 | 1,00 | 0,96 | 1,00 | 0,51 |
| hsa-miR-146b-3p | 23,65 | 11,41 | 2,07 | 0,73 | 0,26 | 1,00 | 0,37 | 1,00 | 0,57 |
| hsa-miR-146b-5p | 154,74 | 156,76 | 0,99 | -0,01 | 0,56 | 1,00 | 0,81 | 1,00 | 0,46 |
| hsa-miR-147 | 83,30 | 52,81 | 1,58 | 0,46 | 0,06 | 1,00 | 0,16 | 1,00 | 0,62 |
| hsa-miR-1470 | 173,68 | 215,60 | 0,81 | -0,22 | 0,17 | 1,00 | 0,21 | 1,00 | 0,41 |
| hsa-miR-1471 | 110,53 | 109,14 | 1,01 | 0,01 | 0,74 | 1,00 | 0,69 | 1,00 | 0,52 |
| hsa-miR-147b | 32,26 | 36,16 | 0,89 | -0,11 | 0,76 | 1,00 | 0,71 | 1,00 | 0,48 |
| hsa-miR-148a | 559,47 | 604,53 | 0,93 | -0,08 | 0,63 | 1,00 | 0,72 | 1,00 | 0,47 |
| hsa-miR-148aS | 42,46 | 30,48 | 1,39 | 0,33 | 0,26 | 1,00 | 0,68 | 1,00 | 0,57 |
| hsa-miR-148b | 458,61 | 486,35 | 0,94 | -0,06 | 0,85 | 1,00 | 0,75 | 1,00 | 0,49 |
| hsa-miR-148bS | 24,51 | 32,35 | 0,76 | -0,28 | 0,57 | 1,00 | 0,86 | 1,00 | 0,46 |
| hsa-miR-149 | 23,44 | 43,21 | 0,54 | -0,61 | 0,48 | 1,00 | 0,33 | 1,00 | 0,45 |
| hsa-miR-149S | 396,36 | 348,63 | 1,14 | 0,13 | 0,23 | 1,00 | 0,23 | 1,00 | 0,58 |
| hsa-miR-150 | 2005,43 | 2106,83 | 0,95 | -0,05 | 0,65 | 1,00 | 0,58 | 1,00 | 0,47 |
| hsa-miR-150S | 89,23 | 50,19 | 1,78 | 0,58 | 0,17 | 1,00 | 0,23 | 1,00 | 0,59 |
| hsa-miR-151-3p | 426,52 | 479,30 | 0,89 | -0,12 | 1,00 | 1,00 | 0,97 | 1,00 | 0,50 |
| hsa-miR-151-5p | 3921,36 | 4726,70 | 0,83 | -0,19 | 0,10 | 1,00 | 0,07 | 1,00 | 0,39 |
| hsa-miR-152 | 191,52 | 199,89 | 0,96 | -0,04 | 0,87 | 1,00 | 0,99 | 1,00 | 0,49 |
| hsa-miR-153 | 119,05 | 132,38 | 0,90 | -0,11 | 0,66 | 1,00 | 0,99 | 1,00 | 0,47 |
| hsa-miR-1537 | 39,44 | 44,11 | 0,89 | -0,11 | 0,88 | 1,00 | 0,67 | 1,00 | 0,51 |
| hsa-miR-1538 | 58,33 | 82,35 | 0,71 | -0,34 | 0,59 | 1,00 | 0,41 | 1,00 | 0,47 |
| hsa-miR-1539 | 84,75 | 97,06 | 0,87 | -0,14 | 0,23 | 1,00 | 0,32 | 1,00 | 0,42 |
| hsa-miR-154 | 1,00 | 1,00 | 1,00 | 0,00 | 0,56 | 1,00 | 0,13 | 1,00 | 0,54 |
| hsa-miR-154S | 57,96 | 50,64 | 1,14 | 0,14 | 0,46 | 1,00 | 0,67 | 1,00 | 0,55 |
| hsa-miR-155 | 67,72 | 77,33 | 0,88 | -0,13 | 0,36 | 1,00 | 0,59 | 1,00 | 0,44 |
| hsa-miR-155S | 67,72 | 66,63 | 1,02 | 0,02 | 0,82 | 1,00 | 0,65 | 1,00 | 0,48 |
| hsa-miR-15a | 3612,86 | 3681,29 | 0,98 | -0,02 | 0,70 | 1,00 | 0,67 | 1,00 | 0,47 |
| hsa-miR-15aS | 124,54 | 120,98 | 1,03 | 0,03 | 0,89 | 1,00 | 0,47 | 1,00 | 0,49 |
| hsa-miR-15b | 14207,24 | 14207,24 | 1,00 | 0,00 | 0,51 | 1,00 | 0,89 | 1,00 | 0,54 |
| hsa-miR-15bS | 74,58 | 83,14 | 0,90 | -0,11 | 0,89 | 1,00 | 0,95 | 1,00 | 0,51 |
| hsa-miR-16 | 17675,49 | 17675,49 | 1,00 | 0,00 | 0,46 | 1,00 | 0,34 | 1,00 | 0,55 |
| hsa-miR-16-1S | 101,63 | 99,16 | 1,02 | 0,02 | 0,78 | 1,00 | 0,98 | 1,00 | 0,48 |
| hsa-miR-16-2S | 80,88 | 66,85 | 1,21 | 0,19 | 0,19 | 1,00 | 0,33 | 1,00 | 0,59 |
| hsa-miR-17 | 8005,76 | 7178,66 | 1,12 | 0,11 | 0,97 | 1,00 | 0,81 | 1,00 | 0,50 |
| hsa-miR-17S | 498,32 | 550,65 | 0,90 | -0,10 | 0,47 | 1,00 | 0,71 | 1,00 | 0,45 |
| hsa-miR-181a | 287,97 | 269,29 | 1,07 | 0,07 | 0,66 | 1,00 | 0,58 | 1,00 | 0,53 |
| hsa-miR-181a-2S | 84,36 | 93,40 | 0,90 | -0,10 | 0,30 | 1,00 | 0,21 | 1,00 | 0,43 |
| hsa-miR-181aS | 70,49 | 54,26 | 1,30 | 0,26 | 0,66 | 1,00 | 0,46 | 1,00 | 0,53 |
| hsa-miR-181b | 40,89 | 33,46 | 1,22 | 0,20 | 0,25 | 1,00 | 0,53 | 1,00 | 0,58 |
| hsa-miR-181c | 83,67 | 109,71 | 0,76 | -0,27 | 0,58 | 1,00 | 0,67 | 1,00 | 0,46 |
| hsa-miR-181cS | 69,82 | 62,15 | 1,12 | 0,12 | 0,65 | 1,00 | 0,66 | 1,00 | 0,47 |
| hsa-miR-181d | 26,46 | 41,26 | 0,64 | -0,44 | 0,31 | 1,00 | 0,40 | 1,00 | 0,43 |
| hsa-miR-182 | 3467,72 | 4503,74 | 0,77 | -0,26 | 0,03 | 1,00 | 0,02 | 1,00 | 0,35 |
| hsa-miR-1825 | 58,84 | 62,35 | 0,94 | -0,06 | 0,95 | 1,00 | 0,89 | 1,00 | 0,50 |
| hsa-miR-1826 | 158,04 | 119,04 | 1,33 | 0,28 | 0,35 | 1,00 | 0,67 | 1,00 | 0,56 |
| hsa-miR-1827 | 29,16 | 26,41 | 1,10 | 0,10 | 0,36 | 1,00 | 0,10 | 1,00 | 0,44 |
| hsa-miR-182S | 39,67 | 29,89 | 1,33 | 0,28 | 0,43 | 1,00 | 0,29 | 1,00 | 0,55 |
| hsa-miR-183 | 259,48 | 377,66 | 0,69 | -0,38 | 0,08 | 1,00 | 0,10 | 1,00 | 0,39 |
| hsa-miR-183S | 113,93 | 125,87 | 0,91 | -0,10 | 0,42 | 1,00 | 0,48 | 1,00 | 0,45 |
| hsa-miR-184 | 46,21 | 23,11 | 2,00 | 0,69 | 0,33 | 1,00 | 0,22 | 1,00 | 0,56 |
| hsa-miR-185 | 20902,49 | 23622,64 | 0,88 | -0,12 | 0,12 | 1,00 | 0,11 | 1,00 | 0,40 |
| hsa-miR-185S | 40,28 | 40,39 | 1,00 | 0,00 | 0,93 | 1,00 | 0,88 | 1,00 | 0,51 |
| hsa-miR-186 | 35,35 | 35,82 | 0,99 | -0,01 | 0,63 | 1,00 | 0,64 | 1,00 | 0,53 |
| hsa-miR-186S | 125,65 | 108,88 | 1,15 | 0,14 | 0,76 | 1,00 | 0,93 | 1,00 | 0,52 |
| hsa-miR-187 | 34,21 | 14,51 | 2,36 | 0,86 | 0,24 | 1,00 | 0,40 | 1,00 | 0,58 |
| hsa-miR-187S | 191,96 | 152,00 | 1,26 | 0,23 | 0,34 | 1,00 | 0,46 | 1,00 | 0,56 |
| hsa-miR-188-3p | 132,95 | 152,93 | 0,87 | -0,14 | 0,49 | 1,00 | 0,77 | 1,00 | 0,45 |
| hsa-miR-188-5p | 127,83 | 97,47 | 1,31 | 0,27 | 0,03 | 1,00 | 0,02 | 1,00 | 0,64 |
| hsa-miR-18a | 840,00 | 828,12 | 1,01 | 0,01 | 0,77 | 1,00 | 0,57 | 1,00 | 0,52 |
| hsa-miR-18aS | 158,04 | 143,54 | 1,10 | 0,10 | 0,53 | 1,00 | 0,60 | 1,00 | 0,54 |
| hsa-miR-18b | 232,10 | 196,77 | 1,18 | 0,17 | 0,57 | 1,00 | 0,58 | 1,00 | 0,54 |
| hsa-miR-18bS | 83,30 | 82,25 | 1,01 | 0,01 | 0,66 | 1,00 | 0,50 | 1,00 | 0,53 |
| hsa-miR-190 | 1,00 | 3,91 | 0,26 | -1,36 | 0,26 | 1,00 | 0,81 | 1,00 | 0,43 |
| hsa-miR-1908 | 494,80 | 415,68 | 1,19 | 0,17 | 0,41 | 1,00 | 0,13 | 1,00 | 0,55 |
| hsa-miR-1909 | 148,29 | 132,03 | 1,12 | 0,12 | 0,06 | 1,00 | 0,02 | 1,00 | 0,62 |
| hsa-miR-1909S | 52,19 | 54,67 | 0,95 | -0,05 | 0,57 | 1,00 | 0,56 | 1,00 | 0,54 |
| hsa-miR-190b | 1,00 | 1,00 | 1,00 | 0,00 | 0,97 | 1,00 | 0,69 | 1,00 | 0,50 |
| hsa-miR-191 | 11503,91 | 11503,91 | 1,00 | 0,00 | 0,24 | 1,00 | 0,35 | 1,00 | 0,42 |
| hsa-miR-1910 | 14,81 | 46,79 | 0,32 | -1,15 | 0,03 | 1,00 | 0,53 | 1,00 | 0,36 |
| hsa-miR-1911 | 45,26 | 28,13 | 1,61 | 0,48 | 0,16 | 1,00 | 0,27 | 1,00 | 0,59 |
| hsa-miR-1911S | 79,86 | 103,24 | 0,77 | -0,26 | 0,74 | 1,00 | 0,68 | 1,00 | 0,48 |
| hsa-miR-1912 | 156,92 | 146,71 | 1,07 | 0,07 | 0,78 | 1,00 | 1,00 | 1,00 | 0,52 |
| hsa-miR-1913 | 308,57 | 334,72 | 0,92 | -0,08 | 0,80 | 1,00 | 0,69 | 1,00 | 0,48 |
| hsa-miR-1914 | 59,54 | 87,33 | 0,68 | -0,38 | 0,09 | 1,00 | 0,24 | 1,00 | 0,39 |
| hsa-miR-1914S | 117,68 | 154,38 | 0,76 | -0,27 | 0,09 | 1,00 | 0,12 | 1,00 | 0,39 |
| hsa-miR-1915 | 521,37 | 469,24 | 1,11 | 0,11 | 0,30 | 1,00 | 0,11 | 1,00 | 0,57 |
| hsa-miR-1915S | 12,75 | 13,87 | 0,92 | -0,08 | 0,77 | 1,00 | 0,39 | 1,00 | 0,48 |
| hsa-miR-191S | 57,33 | 42,46 | 1,35 | 0,30 | 0,32 | 1,00 | 0,17 | 1,00 | 0,57 |
| hsa-miR-192 | 4503,74 | 4831,83 | 0,93 | -0,07 | 0,24 | 1,00 | 0,35 | 1,00 | 0,42 |
| hsa-miR-192S | 82,77 | 102,42 | 0,81 | -0,21 | 1,00 | 1,00 | 0,71 | 1,00 | 0,50 |
| hsa-miR-193a-3p | 174,41 | 148,56 | 1,17 | 0,16 | 0,39 | 1,00 | 0,41 | 1,00 | 0,56 |
| hsa-miR-193a-5p | 59,44 | 75,50 | 0,79 | -0,24 | 0,60 | 1,00 | 0,78 | 1,00 | 0,47 |
| hsa-miR-193b | 84,75 | 100,05 | 0,85 | -0,17 | 0,28 | 1,00 | 0,48 | 1,00 | 0,43 |
| hsa-miR-193bS | 53,67 | 51,44 | 1,04 | 0,04 | 0,91 | 1,00 | 0,98 | 1,00 | 0,51 |
| hsa-miR-194 | 4503,74 | 4726,70 | 0,95 | -0,05 | 0,76 | 1,00 | 0,57 | 1,00 | 0,48 |
| hsa-miR-194S | 83,30 | 98,13 | 0,85 | -0,16 | 0,22 | 1,00 | 0,23 | 1,00 | 0,42 |
| hsa-miR-195 | 741,74 | 729,18 | 1,02 | 0,02 | 0,88 | 1,00 | 0,72 | 1,00 | 0,51 |
| hsa-miR-195S | 77,19 | 64,58 | 1,20 | 0,18 | 0,64 | 1,00 | 0,47 | 1,00 | 0,53 |
| hsa-miR-196a | 15,88 | 2,32 | 6,84 | 1,92 | 0,78 | 1,00 | 0,79 | 1,00 | 0,52 |
| hsa-miR-196aS | 129,59 | 162,46 | 0,80 | -0,23 | 0,15 | 1,00 | 0,65 | 1,00 | 0,41 |
| hsa-miR-196b | 3,09 | 1,00 | 3,09 | 1,13 | 0,50 | 1,00 | 0,48 | 1,00 | 0,54 |
| hsa-miR-197 | 462,92 | 370,89 | 1,25 | 0,22 | 0,11 | 1,00 | 0,11 | 1,00 | 0,60 |
| hsa-miR-198 | 84,09 | 69,93 | 1,20 | 0,18 | 0,67 | 1,00 | 0,72 | 1,00 | 0,53 |
| hsa-miR-199a-3p | 139,10 | 138,20 | 1,01 | 0,01 | 0,92 | 1,00 | 0,93 | 1,00 | 0,49 |
| hsa-miR-199a-5p | 247,41 | 179,47 | 1,38 | 0,32 | 0,44 | 1,00 | 0,49 | 1,00 | 0,55 |
| hsa-miR-199b-3p | 121,90 | 107,97 | 1,13 | 0,12 | 0,20 | 1,00 | 0,26 | 1,00 | 0,58 |
| hsa-miR-199b-5p | 14,81 | 19,64 | 0,75 | -0,28 | 0,78 | 1,00 | 0,94 | 1,00 | 0,48 |
| hsa-miR-19a | 1907,68 | 1981,91 | 0,96 | -0,04 | 0,88 | 1,00 | 0,83 | 1,00 | 0,51 |
| hsa-miR-19aS | 57,96 | 52,16 | 1,11 | 0,11 | 0,74 | 1,00 | 0,84 | 1,00 | 0,52 |
| hsa-miR-19b | 9361,26 | 9651,25 | 0,97 | -0,03 | 0,59 | 1,00 | 0,61 | 1,00 | 0,54 |
| hsa-miR-19b-1S | 90,13 | 71,30 | 1,26 | 0,23 | 0,84 | 1,00 | 0,81 | 1,00 | 0,51 |
| hsa-miR-19b-2S | 1,00 | 1,00 | 1,00 | 0,00 | 0,77 | 1,00 | 0,74 | 1,00 | 0,48 |
| hsa-miR-200a | 84,59 | 71,01 | 1,19 | 0,17 | 0,92 | 1,00 | 0,33 | 1,00 | 0,49 |
| hsa-miR-200aS | 96,00 | 73,25 | 1,31 | 0,27 | 0,59 | 1,00 | 0,59 | 1,00 | 0,54 |
| hsa-miR-200b | 57,33 | 57,16 | 1,00 | 0,00 | 0,79 | 1,00 | 0,98 | 1,00 | 0,48 |
| hsa-miR-200bS | 58,98 | 90,51 | 0,65 | -0,43 | 0,47 | 1,00 | 0,44 | 1,00 | 0,45 |
| hsa-miR-200c | 87,58 | 95,14 | 0,92 | -0,08 | 0,95 | 1,00 | 0,73 | 1,00 | 0,50 |
| hsa-miR-200cS | 32,60 | 34,31 | 0,95 | -0,05 | 0,90 | 1,00 | 0,62 | 1,00 | 0,51 |
| hsa-miR-202 | 1,00 | 8,79 | 0,11 | -2,17 | 0,35 | 1,00 | 0,99 | 1,00 | 0,44 |
| hsa-miR-202S | 71,51 | 72,46 | 0,99 | -0,01 | 0,46 | 1,00 | 0,30 | 1,00 | 0,45 |
| hsa-miR-203 | 40,21 | 32,78 | 1,23 | 0,20 | 0,82 | 1,00 | 0,96 | 1,00 | 0,52 |
| hsa-miR-204 | 12,07 | 7,13 | 1,69 | 0,53 | 0,79 | 1,00 | 0,99 | 1,00 | 0,52 |
| hsa-miR-205 | 67,81 | 54,04 | 1,25 | 0,23 | 0,76 | 1,00 | 0,53 | 1,00 | 0,52 |
| hsa-miR-206 | 28,07 | 9,38 | 2,99 | 1,10 | 0,12 | 1,00 | 0,07 | 1,00 | 0,60 |
| hsa-miR-208a | 80,88 | 77,73 | 1,04 | 0,04 | 0,93 | 1,00 | 0,63 | 1,00 | 0,49 |
| hsa-miR-208b | 45,40 | 71,07 | 0,64 | -0,45 | 0,04 | 1,00 | 0,07 | 1,00 | 0,37 |
| hsa-miR-20a | 4018,37 | 3801,33 | 1,06 | 0,06 | 0,94 | 1,00 | 0,61 | 1,00 | 0,50 |
| hsa-miR-20aS | 119,96 | 154,06 | 0,78 | -0,25 | 0,32 | 1,00 | 0,28 | 1,00 | 0,43 |
| hsa-miR-20b | 2637,21 | 2858,37 | 0,92 | -0,08 | 0,46 | 1,00 | 0,52 | 1,00 | 0,45 |
| hsa-miR-20bS | 64,47 | 77,43 | 0,83 | -0,18 | 0,39 | 1,00 | 0,86 | 1,00 | 0,44 |
| hsa-miR-21 | 998,96 | 1047,22 | 0,95 | -0,05 | 0,88 | 1,00 | 0,78 | 1,00 | 0,51 |
| hsa-miR-210 | 372,43 | 425,35 | 0,88 | -0,13 | 0,23 | 1,00 | 0,99 | 1,00 | 0,42 |
| hsa-miR-211 | 1,00 | 1,00 | 1,00 | 0,00 | 0,57 | 1,00 | 0,49 | 1,00 | 0,53 |
| hsa-miR-212 | 18,49 | 35,17 | 0,53 | -0,64 | 0,20 | 1,00 | 0,28 | 1,00 | 0,42 |
| hsa-miR-214 | 342,78 | 290,46 | 1,18 | 0,17 | 0,62 | 1,00 | 0,68 | 1,00 | 0,53 |
| hsa-miR-214S | 72,82 | 58,75 | 1,24 | 0,21 | 0,88 | 1,00 | 1,00 | 1,00 | 0,51 |
| hsa-miR-215 | 370,14 | 377,46 | 0,98 | -0,02 | 0,60 | 1,00 | 0,51 | 1,00 | 0,47 |
| hsa-miR-216a | 101,33 | 122,52 | 0,83 | -0,19 | 0,43 | 1,00 | 0,42 | 1,00 | 0,45 |
| hsa-miR-216b | 144,13 | 148,77 | 0,97 | -0,03 | 0,71 | 1,00 | 0,95 | 1,00 | 0,48 |
| hsa-miR-217 | 132,95 | 113,40 | 1,17 | 0,16 | 0,16 | 1,00 | 0,52 | 1,00 | 0,59 |
| hsa-miR-218 | 24,51 | 42,35 | 0,58 | -0,55 | 0,76 | 1,00 | 0,91 | 1,00 | 0,48 |
| hsa-miR-218-1S | 110,94 | 86,41 | 1,28 | 0,25 | 0,09 | 1,00 | 0,08 | 1,00 | 0,61 |
| hsa-miR-218-2S | 58,84 | 65,37 | 0,90 | -0,11 | 0,28 | 1,00 | 0,64 | 1,00 | 0,43 |
| hsa-miR-219-1-3p | 23,65 | 12,50 | 1,89 | 0,64 | 0,94 | 1,00 | 0,30 | 1,00 | 0,51 |
| hsa-miR-219-2-3p | 8,53 | 11,25 | 0,76 | -0,28 | 0,74 | 1,00 | 0,57 | 1,00 | 0,48 |
| hsa-miR-219-5p | 61,37 | 53,72 | 1,14 | 0,13 | 0,86 | 1,00 | 0,34 | 1,00 | 0,51 |
| hsa-miR-21S | 59,54 | 77,49 | 0,77 | -0,26 | 0,82 | 1,00 | 0,80 | 1,00 | 0,49 |
| hsa-miR-22 | 6386,57 | 6538,68 | 0,98 | -0,02 | 0,79 | 1,00 | 0,49 | 1,00 | 0,52 |
| hsa-miR-220a | 51,16 | 62,62 | 0,82 | -0,20 | 0,65 | 1,00 | 0,75 | 1,00 | 0,47 |
| hsa-miR-220b | 57,96 | 30,02 | 1,93 | 0,66 | 0,11 | 1,00 | 0,10 | 1,00 | 0,60 |
| hsa-miR-220c | 119,96 | 85,25 | 1,41 | 0,34 | 0,09 | 1,00 | 0,10 | 1,00 | 0,61 |
| hsa-miR-221 | 96,35 | 61,29 | 1,57 | 0,45 | 0,07 | 1,00 | 0,16 | 1,00 | 0,62 |
| hsa-miR-221S | 60,81 | 89,23 | 0,68 | -0,38 | 0,10 | 1,00 | 0,15 | 1,00 | 0,39 |
| hsa-miR-222 | 372,43 | 342,78 | 1,09 | 0,08 | 0,98 | 1,00 | 0,87 | 1,00 | 0,50 |
| hsa-miR-222S | 47,75 | 47,18 | 1,01 | 0,01 | 1,00 | 1,00 | 0,80 | 1,00 | 0,50 |
| hsa-miR-223 | 1799,95 | 1956,56 | 0,92 | -0,08 | 0,88 | 1,00 | 0,75 | 1,00 | 0,49 |
| hsa-miR-223S | 62,56 | 76,84 | 0,81 | -0,21 | 0,87 | 1,00 | 0,83 | 1,00 | 0,49 |
| hsa-miR-224 | 30,74 | 18,09 | 1,70 | 0,53 | 0,62 | 1,00 | 0,59 | 1,00 | 0,53 |
| hsa-miR-22S | 58,84 | 88,37 | 0,67 | -0,41 | 0,05 | 1,00 | 0,04 | 1,00 | 0,37 |
| hsa-miR-23a | 4503,74 | 3749,72 | 1,20 | 0,18 | 0,41 | 1,00 | 0,34 | 1,00 | 0,55 |
| hsa-miR-23aS | 1,00 | 14,44 | 0,07 | -2,67 | 0,65 | 1,00 | 0,62 | 1,00 | 0,47 |
| hsa-miR-23b | 3749,72 | 3467,72 | 1,08 | 0,08 | 0,11 | 1,00 | 0,12 | 1,00 | 0,61 |
| hsa-miR-23bS | 31,75 | 29,17 | 1,09 | 0,09 | 0,88 | 1,00 | 0,61 | 1,00 | 0,49 |
| hsa-miR-24 | 1405,52 | 1470,36 | 0,96 | -0,05 | 1,00 | 1,00 | 0,59 | 1,00 | 0,50 |
| hsa-miR-24-1S | 112,08 | 88,75 | 1,26 | 0,23 | 0,91 | 1,00 | 0,51 | 1,00 | 0,49 |
| hsa-miR-24-2S | 144,60 | 144,13 | 1,00 | 0,00 | 0,91 | 1,00 | 0,50 | 1,00 | 0,51 |
| hsa-miR-25 | 6386,57 | 5591,87 | 1,14 | 0,13 | 0,58 | 1,00 | 0,68 | 1,00 | 0,54 |
| hsa-miR-25S | 74,79 | 37,89 | 1,97 | 0,68 | 0,05 | 1,00 | 0,05 | 1,00 | 0,63 |
| hsa-miR-26a | 7079,20 | 6690,78 | 1,06 | 0,06 | 0,09 | 1,00 | 0,05 | 1,00 | 0,61 |
| hsa-miR-26a-1S | 16,95 | 5,82 | 2,91 | 1,07 | 0,35 | 1,00 | 0,58 | 1,00 | 0,56 |
| hsa-miR-26a-2S | 1,00 | 1,00 | 1,00 | 0,00 | 0,35 | 1,00 | 0,32 | 1,00 | 0,55 |
| hsa-miR-26b | 599,29 | 530,91 | 1,13 | 0,12 | 0,63 | 1,00 | 0,74 | 1,00 | 0,53 |
| hsa-miR-26bS | 12,07 | 6,73 | 1,79 | 0,58 | 0,61 | 1,00 | 0,86 | 1,00 | 0,53 |
| hsa-miR-27a | 357,27 | 363,69 | 0,98 | -0,02 | 0,96 | 1,00 | 1,00 | 1,00 | 0,50 |
| hsa-miR-27aS | 42,04 | 59,36 | 0,71 | -0,35 | 0,07 | 1,00 | 0,06 | 1,00 | 0,38 |
| hsa-miR-27b | 188,75 | 171,43 | 1,10 | 0,10 | 0,23 | 1,00 | 0,24 | 1,00 | 0,58 |
| hsa-miR-27bS | 29,16 | 29,81 | 0,98 | -0,02 | 0,77 | 1,00 | 0,55 | 1,00 | 0,52 |
| hsa-miR-28-3p | 124,99 | 105,11 | 1,19 | 0,17 | 0,60 | 1,00 | 0,70 | 1,00 | 0,53 |
| hsa-miR-28-5p | 342,78 | 405,84 | 0,84 | -0,17 | 0,35 | 1,00 | 0,63 | 1,00 | 0,44 |
| hsa-miR-296-3p | 97,27 | 111,71 | 0,87 | -0,14 | 0,65 | 1,00 | 0,46 | 1,00 | 0,47 |
| hsa-miR-296-5p | 296,61 | 376,78 | 0,79 | -0,24 | 0,80 | 1,00 | 0,91 | 1,00 | 0,48 |
| hsa-miR-297 | 74,98 | 80,75 | 0,93 | -0,07 | 0,77 | 1,00 | 0,30 | 1,00 | 0,52 |
| hsa-miR-298 | 158,82 | 93,75 | 1,69 | 0,53 | 0,15 | 1,00 | 0,29 | 1,00 | 0,59 |
| hsa-miR-299-3p | 3,09 | 5,42 | 0,57 | -0,56 | 0,76 | 1,00 | 0,70 | 1,00 | 0,48 |
| hsa-miR-299-5p | 31,75 | 43,86 | 0,72 | -0,32 | 0,67 | 1,00 | 0,75 | 1,00 | 0,47 |
| hsa-miR-29a | 494,80 | 536,52 | 0,92 | -0,08 | 0,56 | 1,00 | 0,36 | 1,00 | 0,46 |
| hsa-miR-29aS | 4,70 | 1,00 | 4,70 | 1,55 | 0,53 | 1,00 | 0,83 | 1,00 | 0,54 |
| hsa-miR-29b | 298,95 | 319,33 | 0,94 | -0,07 | 0,96 | 1,00 | 0,88 | 1,00 | 0,50 |
| hsa-miR-29b-1S | 12,25 | 10,81 | 1,13 | 0,12 | 0,88 | 1,00 | 0,51 | 1,00 | 0,51 |
| hsa-miR-29b-2S | 57,96 | 62,88 | 0,92 | -0,08 | 0,77 | 1,00 | 0,91 | 1,00 | 0,52 |
| hsa-miR-29c | 370,14 | 425,13 | 0,87 | -0,14 | 0,75 | 1,00 | 0,87 | 1,00 | 0,48 |
| hsa-miR-29cS | 1,00 | 9,40 | 0,11 | -2,24 | 0,77 | 1,00 | 0,92 | 1,00 | 0,48 |
| hsa-miR-300 | 26,46 | 26,13 | 1,01 | 0,01 | 0,46 | 1,00 | 0,46 | 1,00 | 0,45 |
| hsa-miR-301a | 223,01 | 227,04 | 0,98 | -0,02 | 0,85 | 1,00 | 0,59 | 1,00 | 0,49 |
| hsa-miR-301b | 178,50 | 186,78 | 0,96 | -0,05 | 0,84 | 1,00 | 0,63 | 1,00 | 0,49 |
| hsa-miR-302a | 1,00 | 1,00 | 1,00 | 0,00 | 0,96 | 1,00 | 1,00 | 1,00 | 0,50 |
| hsa-miR-302aS | 44,42 | 30,32 | 1,46 | 0,38 | 0,24 | 1,00 | 0,43 | 1,00 | 0,58 |
| hsa-miR-302b | 4,70 | 1,00 | 4,70 | 1,55 | 0,80 | 1,00 | 0,45 | 1,00 | 0,52 |
| hsa-miR-302bS | 20,28 | 14,62 | 1,39 | 0,33 | 0,69 | 1,00 | 0,47 | 1,00 | 0,53 |
| hsa-miR-302c | 25,30 | 31,52 | 0,80 | -0,22 | 0,94 | 1,00 | 0,60 | 1,00 | 0,49 |
| hsa-miR-302cS | 16,82 | 37,37 | 0,45 | -0,80 | 0,41 | 1,00 | 0,39 | 1,00 | 0,45 |
| hsa-miR-302d | 12,07 | 16,46 | 0,73 | -0,31 | 0,82 | 1,00 | 0,61 | 1,00 | 0,49 |
| hsa-miR-302dS | 60,04 | 71,28 | 0,84 | -0,17 | 0,88 | 1,00 | 0,98 | 1,00 | 0,51 |
| hsa-miR-302e | 15,96 | 7,49 | 2,13 | 0,76 | 0,65 | 1,00 | 0,61 | 1,00 | 0,53 |
| hsa-miR-302f | 1,00 | 1,00 | 1,00 | 0,00 | 0,69 | 1,00 | 0,61 | 1,00 | 0,52 |
| hsa-miR-30a | 206,50 | 295,22 | 0,70 | -0,36 | 0,69 | 1,00 | 0,51 | 1,00 | 0,47 |
| hsa-miR-30aS | 30,14 | 51,25 | 0,59 | -0,53 | 0,19 | 1,00 | 0,56 | 1,00 | 0,41 |
| hsa-miR-30b | 7477,04 | 7178,66 | 1,04 | 0,04 | 0,23 | 1,00 | 0,11 | 1,00 | 0,58 |
| hsa-miR-30bS | 43,09 | 56,42 | 0,76 | -0,27 | 0,94 | 1,00 | 0,74 | 1,00 | 0,49 |
| hsa-miR-30c | 2106,83 | 2158,04 | 0,98 | -0,02 | 0,88 | 1,00 | 0,71 | 1,00 | 0,49 |
| hsa-miR-30c-1S | 35,44 | 8,42 | 4,21 | 1,44 | 0,21 | 1,00 | 0,45 | 1,00 | 0,58 |
| hsa-miR-30c-2S | 3,56 | 27,48 | 0,13 | -2,04 | 0,11 | 1,00 | 0,61 | 1,00 | 0,40 |
| hsa-miR-30d | 4936,96 | 4936,96 | 1,00 | 0,00 | 0,76 | 1,00 | 0,48 | 1,00 | 0,48 |
| hsa-miR-30dS | 62,20 | 52,98 | 1,17 | 0,16 | 0,80 | 1,00 | 0,44 | 1,00 | 0,48 |
| hsa-miR-30e | 144,29 | 233,18 | 0,62 | -0,48 | 0,73 | 1,00 | 0,54 | 1,00 | 0,48 |
| hsa-miR-30eS | 69,82 | 50,98 | 1,37 | 0,31 | 0,68 | 1,00 | 0,90 | 1,00 | 0,53 |
| hsa-miR-31 | 164,32 | 158,78 | 1,03 | 0,03 | 0,69 | 1,00 | 0,37 | 1,00 | 0,47 |
| hsa-miR-31S | 147,57 | 154,08 | 0,96 | -0,04 | 0,15 | 1,00 | 0,48 | 1,00 | 0,41 |
| hsa-miR-32 | 156,92 | 153,54 | 1,02 | 0,02 | 0,56 | 1,00 | 0,55 | 1,00 | 0,54 |
| hsa-miR-320a | 6067,71 | 6067,71 | 1,00 | 0,00 | 0,92 | 1,00 | 0,80 | 1,00 | 0,49 |
| hsa-miR-320b | 1212,00 | 1198,43 | 1,01 | 0,01 | 0,71 | 1,00 | 0,61 | 1,00 | 0,52 |
| hsa-miR-320c | 302,11 | 275,49 | 1,10 | 0,09 | 0,56 | 1,00 | 0,50 | 1,00 | 0,54 |
| hsa-miR-320d | 296,61 | 284,92 | 1,04 | 0,04 | 0,34 | 1,00 | 0,23 | 1,00 | 0,56 |
| hsa-miR-323-3p | 45,72 | 32,47 | 1,41 | 0,34 | 0,55 | 1,00 | 0,57 | 1,00 | 0,54 |
| hsa-miR-323-5p | 44,42 | 65,20 | 0,68 | -0,38 | 0,54 | 1,00 | 0,59 | 1,00 | 0,46 |
| hsa-miR-324-3p | 491,01 | 436,16 | 1,13 | 0,12 | 0,56 | 1,00 | 0,47 | 1,00 | 0,54 |
| hsa-miR-324-5p | 302,11 | 287,41 | 1,05 | 0,05 | 1,00 | 1,00 | 0,92 | 1,00 | 0,50 |
| hsa-miR-325 | 47,75 | 26,14 | 1,83 | 0,60 | 0,14 | 1,00 | 0,07 | 1,00 | 0,60 |
| hsa-miR-326 | 103,72 | 76,76 | 1,35 | 0,30 | 0,06 | 1,00 | 0,04 | 1,00 | 0,62 |
| hsa-miR-328 | 52,58 | 44,75 | 1,17 | 0,16 | 0,69 | 1,00 | 0,73 | 1,00 | 0,53 |
| hsa-miR-329 | 58,70 | 64,95 | 0,90 | -0,10 | 0,75 | 1,00 | 0,37 | 1,00 | 0,52 |
| hsa-miR-32S | 106,96 | 65,47 | 1,63 | 0,49 | 0,08 | 1,00 | 0,03 | 1,00 | 0,61 |
| hsa-miR-330-3p | 264,67 | 326,38 | 0,81 | -0,21 | 0,26 | 1,00 | 0,33 | 1,00 | 0,43 |
| hsa-miR-330-5p | 28,23 | 24,27 | 1,16 | 0,15 | 0,62 | 1,00 | 0,59 | 1,00 | 0,53 |
| hsa-miR-331-3p | 666,41 | 583,78 | 1,14 | 0,13 | 0,69 | 1,00 | 0,79 | 1,00 | 0,53 |
| hsa-miR-331-5p | 55,81 | 26,77 | 2,08 | 0,73 | 0,20 | 1,00 | 0,31 | 1,00 | 0,58 |
| hsa-miR-335 | 409,76 | 343,68 | 1,19 | 0,18 | 0,61 | 1,00 | 0,77 | 1,00 | 0,53 |
| hsa-miR-335S | 18,63 | 1,00 | 18,63 | 2,92 | 0,09 | 1,00 | 0,43 | 1,00 | 0,61 |
| hsa-miR-337-3p | 31,75 | 52,98 | 0,60 | -0,51 | 0,19 | 1,00 | 0,15 | 1,00 | 0,41 |
| hsa-miR-337-5p | 36,28 | 33,21 | 1,09 | 0,09 | 0,75 | 1,00 | 0,59 | 1,00 | 0,52 |
| hsa-miR-338-3p | 115,09 | 90,14 | 1,28 | 0,24 | 0,29 | 1,00 | 0,27 | 1,00 | 0,57 |
| hsa-miR-338-5p | 58,33 | 52,89 | 1,10 | 0,10 | 0,93 | 1,00 | 0,82 | 1,00 | 0,51 |
| hsa-miR-339-3p | 265,79 | 283,39 | 0,94 | -0,06 | 0,52 | 1,00 | 0,82 | 1,00 | 0,46 |
| hsa-miR-339-5p | 443,98 | 534,07 | 0,83 | -0,18 | 0,22 | 1,00 | 0,20 | 1,00 | 0,42 |
| hsa-miR-33a | 81,44 | 80,07 | 1,02 | 0,02 | 0,36 | 1,00 | 0,35 | 1,00 | 0,44 |
| hsa-miR-33aS | 82,77 | 81,05 | 1,02 | 0,02 | 0,67 | 1,00 | 0,43 | 1,00 | 0,53 |
| hsa-miR-33b | 168,89 | 145,36 | 1,16 | 0,15 | 0,58 | 1,00 | 0,55 | 1,00 | 0,54 |
| hsa-miR-33bS | 135,69 | 154,05 | 0,88 | -0,13 | 0,43 | 1,00 | 0,42 | 1,00 | 0,45 |
| hsa-miR-340 | 152,22 | 125,53 | 1,21 | 0,19 | 0,15 | 1,00 | 0,37 | 1,00 | 0,59 |
| hsa-miR-340S | 20,86 | 19,93 | 1,05 | 0,05 | 0,91 | 1,00 | 0,54 | 1,00 | 0,51 |
| hsa-miR-342-3p | 3004,33 | 3467,72 | 0,87 | -0,14 | 0,05 | 1,00 | 0,04 | 1,00 | 0,37 |
| hsa-miR-342-5p | 100,18 | 106,95 | 0,94 | -0,07 | 0,78 | 1,00 | 0,74 | 1,00 | 0,48 |
| hsa-miR-345 | 84,59 | 101,60 | 0,83 | -0,18 | 0,41 | 1,00 | 0,80 | 1,00 | 0,45 |
| hsa-miR-346 | 59,44 | 82,93 | 0,72 | -0,33 | 0,05 | 1,00 | 0,02 | 1,00 | 0,37 |
| hsa-miR-34a | 43,25 | 47,89 | 0,90 | -0,10 | 0,88 | 1,00 | 0,63 | 1,00 | 0,51 |
| hsa-miR-34aS | 116,50 | 119,50 | 0,97 | -0,03 | 0,69 | 1,00 | 0,28 | 1,00 | 0,47 |
| hsa-miR-34b | 60,04 | 37,93 | 1,58 | 0,46 | 0,30 | 1,00 | 0,22 | 1,00 | 0,57 |
| hsa-miR-34bS | 42,46 | 67,66 | 0,63 | -0,47 | 0,42 | 1,00 | 0,45 | 1,00 | 0,45 |
| hsa-miR-34c-3p | 95,58 | 92,54 | 1,03 | 0,03 | 0,84 | 1,00 | 0,80 | 1,00 | 0,51 |
| hsa-miR-34c-5p | 59,44 | 74,82 | 0,79 | -0,23 | 0,14 | 1,00 | 0,06 | 1,00 | 0,40 |
| hsa-miR-361-3p | 157,13 | 167,84 | 0,94 | -0,07 | 1,00 | 1,00 | 0,82 | 1,00 | 0,50 |
| hsa-miR-361-5p | 404,48 | 374,83 | 1,08 | 0,08 | 0,80 | 1,00 | 0,93 | 1,00 | 0,48 |
| hsa-miR-362-3p | 225,80 | 224,07 | 1,01 | 0,01 | 0,94 | 1,00 | 0,85 | 1,00 | 0,51 |
| hsa-miR-362-5p | 131,67 | 138,02 | 0,95 | -0,05 | 0,60 | 1,00 | 0,97 | 1,00 | 0,53 |
| hsa-miR-363 | 3312,49 | 3681,29 | 0,90 | -0,11 | 0,23 | 1,00 | 0,45 | 1,00 | 0,42 |
| hsa-miR-363S | 42,04 | 41,19 | 1,02 | 0,02 | 0,79 | 1,00 | 0,78 | 1,00 | 0,52 |
| hsa-miR-365 | 102,02 | 90,40 | 1,13 | 0,12 | 0,80 | 1,00 | 0,62 | 1,00 | 0,52 |
| hsa-miR-367 | 46,98 | 65,75 | 0,71 | -0,34 | 0,13 | 1,00 | 0,13 | 1,00 | 0,40 |
| hsa-miR-367S | 67,47 | 62,89 | 1,07 | 0,07 | 0,88 | 1,00 | 0,74 | 1,00 | 0,49 |
| hsa-miR-369-3p | 1,00 | 8,49 | 0,12 | -2,14 | 0,26 | 1,00 | 0,18 | 1,00 | 0,43 |
| hsa-miR-369-5p | 29,61 | 40,85 | 0,72 | -0,32 | 0,41 | 1,00 | 0,61 | 1,00 | 0,45 |
| hsa-miR-370 | 57,47 | 54,46 | 1,06 | 0,05 | 1,00 | 1,00 | 0,97 | 1,00 | 0,50 |
| hsa-miR-371-3p | 24,39 | 36,87 | 0,66 | -0,41 | 0,40 | 1,00 | 0,79 | 1,00 | 0,45 |
| hsa-miR-371-5p | 20,28 | 39,28 | 0,52 | -0,66 | 0,43 | 1,00 | 0,44 | 1,00 | 0,45 |
| hsa-miR-372 | 24,51 | 32,54 | 0,75 | -0,28 | 0,21 | 1,00 | 0,37 | 1,00 | 0,42 |
| hsa-miR-373 | 12,75 | 3,40 | 3,75 | 1,32 | 0,70 | 1,00 | 0,42 | 1,00 | 0,52 |
| hsa-miR-373S | 20,28 | 24,29 | 0,83 | -0,18 | 0,52 | 1,00 | 0,78 | 1,00 | 0,46 |
| hsa-miR-374a | 494,80 | 517,73 | 0,96 | -0,05 | 0,71 | 1,00 | 0,29 | 1,00 | 0,48 |
| hsa-miR-374aS | 44,42 | 39,04 | 1,14 | 0,13 | 0,15 | 1,00 | 0,15 | 1,00 | 0,59 |
| hsa-miR-374b | 458,61 | 487,24 | 0,94 | -0,06 | 0,48 | 1,00 | 0,28 | 1,00 | 0,45 |
| hsa-miR-374bS | 58,70 | 45,40 | 1,29 | 0,26 | 0,29 | 1,00 | 0,17 | 1,00 | 0,57 |
| hsa-miR-375 | 4,35 | 34,99 | 0,12 | -2,08 | 0,05 | 1,00 | 0,11 | 1,00 | 0,38 |
| hsa-miR-376a | 96,35 | 106,22 | 0,91 | -0,10 | 0,96 | 1,00 | 0,61 | 1,00 | 0,50 |
| hsa-miR-376aS | 1,58 | 37,64 | 0,04 | -3,17 | 0,10 | 1,00 | 0,43 | 1,00 | 0,39 |
| hsa-miR-376b | 45,56 | 52,39 | 0,87 | -0,14 | 0,34 | 1,00 | 0,21 | 1,00 | 0,56 |
| hsa-miR-376c | 82,84 | 68,62 | 1,21 | 0,19 | 0,64 | 1,00 | 0,53 | 1,00 | 0,53 |
| hsa-miR-377 | 154,74 | 109,41 | 1,41 | 0,35 | 0,21 | 1,00 | 0,24 | 1,00 | 0,58 |
| hsa-miR-377S | 1,00 | 1,00 | 1,00 | 0,00 | 0,81 | 1,00 | 0,57 | 1,00 | 0,49 |
| hsa-miR-378 | 168,89 | 135,80 | 1,24 | 0,22 | 0,09 | 1,00 | 0,03 | 1,00 | 0,61 |
| hsa-miR-378S | 46,95 | 42,78 | 1,10 | 0,09 | 0,92 | 1,00 | 0,47 | 1,00 | 0,49 |
| hsa-miR-379 | 1,00 | 4,25 | 0,24 | -1,45 | 0,53 | 1,00 | 0,88 | 1,00 | 0,46 |
| hsa-miR-379S | 43,61 | 52,46 | 0,83 | -0,18 | 0,91 | 1,00 | 0,69 | 1,00 | 0,49 |
| hsa-miR-380 | 12,07 | 1,00 | 12,07 | 2,49 | 0,27 | 1,00 | 0,34 | 1,00 | 0,57 |
| hsa-miR-380S | 71,07 | 86,00 | 0,83 | -0,19 | 0,13 | 1,00 | 0,03 | 1,00 | 0,40 |
| hsa-miR-381 | 96,72 | 93,75 | 1,03 | 0,03 | 0,73 | 1,00 | 0,35 | 1,00 | 0,52 |
| hsa-miR-382 | 41,88 | 46,09 | 0,91 | -0,10 | 0,90 | 1,00 | 0,13 | 1,00 | 0,49 |
| hsa-miR-383 | 67,81 | 57,46 | 1,18 | 0,17 | 0,82 | 1,00 | 0,68 | 1,00 | 0,52 |
| hsa-miR-384 | 106,16 | 75,49 | 1,41 | 0,34 | 0,11 | 1,00 | 0,19 | 1,00 | 0,60 |
| hsa-miR-409-3p | 69,82 | 71,44 | 0,98 | -0,02 | 0,77 | 1,00 | 0,55 | 1,00 | 0,48 |
| hsa-miR-409-5p | 88,91 | 91,11 | 0,98 | -0,02 | 0,95 | 1,00 | 0,88 | 1,00 | 0,50 |
| hsa-miR-410 | 103,72 | 99,00 | 1,05 | 0,05 | 0,91 | 1,00 | 0,68 | 1,00 | 0,51 |
| hsa-miR-411 | 20,56 | 52,08 | 0,39 | -0,93 | 0,06 | 1,00 | 0,18 | 1,00 | 0,38 |
| hsa-miR-411S | 45,88 | 58,58 | 0,78 | -0,24 | 0,37 | 1,00 | 0,64 | 1,00 | 0,44 |
| hsa-miR-412 | 46,95 | 55,65 | 0,84 | -0,17 | 0,16 | 1,00 | 0,16 | 1,00 | 0,41 |
| hsa-miR-421 | 168,89 | 143,90 | 1,17 | 0,16 | 0,18 | 1,00 | 0,87 | 1,00 | 0,59 |
| hsa-miR-422a | 143,38 | 130,46 | 1,10 | 0,09 | 0,44 | 1,00 | 0,48 | 1,00 | 0,55 |
| hsa-miR-423-3p | 1030,54 | 897,32 | 1,15 | 0,14 | 0,24 | 1,00 | 0,25 | 1,00 | 0,58 |
| hsa-miR-423-5p | 1405,52 | 1503,36 | 0,93 | -0,07 | 0,45 | 1,00 | 0,78 | 1,00 | 0,45 |
| hsa-miR-424 | 246,54 | 284,16 | 0,87 | -0,14 | 0,40 | 1,00 | 0,55 | 1,00 | 0,45 |
| hsa-miR-424S | 144,29 | 128,62 | 1,12 | 0,11 | 0,46 | 1,00 | 0,63 | 1,00 | 0,55 |
| hsa-miR-425 | 12702,71 | 12702,71 | 1,00 | 0,00 | 0,77 | 1,00 | 0,74 | 1,00 | 0,52 |
| hsa-miR-425S | 48,88 | 52,39 | 0,93 | -0,07 | 0,46 | 1,00 | 0,58 | 1,00 | 0,45 |
| hsa-miR-429 | 57,96 | 57,13 | 1,01 | 0,01 | 0,36 | 1,00 | 0,09 | 1,00 | 0,44 |
| hsa-miR-431 | 140,87 | 185,66 | 0,76 | -0,28 | 0,23 | 1,00 | 0,66 | 1,00 | 0,42 |
| hsa-miR-431S | 16,82 | 16,54 | 1,02 | 0,02 | 0,78 | 1,00 | 0,25 | 1,00 | 0,52 |
| hsa-miR-432 | 11,46 | 1,00 | 11,46 | 2,44 | 0,38 | 1,00 | 0,49 | 1,00 | 0,55 |
| hsa-miR-432S | 52,35 | 63,82 | 0,82 | -0,20 | 0,48 | 1,00 | 0,47 | 1,00 | 0,45 |
| hsa-miR-433 | 97,83 | 60,16 | 1,63 | 0,49 | 0,02 | 1,00 | 0,07 | 1,00 | 0,65 |
| hsa-miR-448 | 118,00 | 118,00 | 1,00 | 0,00 | 0,75 | 1,00 | 0,52 | 1,00 | 0,52 |
| hsa-miR-449a | 1,00 | 1,00 | 1,00 | 0,00 | 0,59 | 1,00 | 0,93 | 1,00 | 0,53 |
| hsa-miR-449b | 71,07 | 45,40 | 1,57 | 0,45 | 0,05 | 1,00 | 0,16 | 1,00 | 0,63 |
| hsa-miR-450a | 32,82 | 35,71 | 0,92 | -0,08 | 0,86 | 1,00 | 0,64 | 1,00 | 0,51 |
| hsa-miR-450b-3p | 16,82 | 18,99 | 0,89 | -0,12 | 0,83 | 1,00 | 0,52 | 1,00 | 0,49 |
| hsa-miR-450b-5p | 56,35 | 57,84 | 0,97 | -0,03 | 0,75 | 1,00 | 0,98 | 1,00 | 0,48 |
| hsa-miR-451 | 1212,00 | 1305,42 | 0,93 | -0,07 | 0,89 | 1,00 | 0,37 | 1,00 | 0,51 |
| hsa-miR-452 | 28,68 | 77,16 | 0,37 | -0,99 | 0,04 | 1,00 | 0,07 | 1,00 | 0,37 |
| hsa-miR-452S | 306,69 | 263,64 | 1,16 | 0,15 | 0,89 | 1,00 | 0,86 | 1,00 | 0,51 |
| hsa-miR-453 | 39,44 | 46,96 | 0,84 | -0,17 | 0,80 | 1,00 | 0,89 | 1,00 | 0,48 |
| hsa-miR-454 | 168,89 | 152,24 | 1,11 | 0,10 | 0,55 | 1,00 | 0,95 | 1,00 | 0,54 |
| hsa-miR-454S | 41,88 | 65,37 | 0,64 | -0,45 | 0,49 | 1,00 | 0,44 | 1,00 | 0,45 |
| hsa-miR-455-3p | 114,56 | 136,43 | 0,84 | -0,17 | 0,38 | 1,00 | 0,86 | 1,00 | 0,44 |
| hsa-miR-455-5p | 28,68 | 30,91 | 0,93 | -0,07 | 0,32 | 1,00 | 0,15 | 1,00 | 0,44 |
| hsa-miR-483-3p | 31,75 | 25,73 | 1,23 | 0,21 | 0,58 | 1,00 | 0,78 | 1,00 | 0,54 |
| hsa-miR-483-5p | 159,10 | 203,15 | 0,78 | -0,24 | 0,89 | 1,00 | 0,28 | 1,00 | 0,51 |
| hsa-miR-484 | 5736,38 | 5736,38 | 1,00 | 0,00 | 0,99 | 1,00 | 0,74 | 1,00 | 0,50 |
| hsa-miR-485-3p | 74,42 | 67,35 | 1,10 | 0,10 | 0,89 | 1,00 | 0,61 | 1,00 | 0,51 |
| hsa-miR-485-5p | 32,53 | 50,39 | 0,65 | -0,44 | 0,08 | 1,00 | 0,24 | 1,00 | 0,39 |
| hsa-miR-486-3p | 274,61 | 389,40 | 0,71 | -0,35 | 0,03 | 1,00 | 0,03 | 1,00 | 0,36 |
| hsa-miR-486-5p | 39283,31 | 39283,31 | 1,00 | 0,00 | 0,21 | 1,00 | 0,24 | 1,00 | 0,53 |
| hsa-miR-487a | 71,07 | 74,60 | 0,95 | -0,05 | 0,78 | 1,00 | 0,95 | 1,00 | 0,48 |
| hsa-miR-487b | 42,32 | 37,05 | 1,14 | 0,13 | 0,95 | 1,00 | 0,60 | 1,00 | 0,50 |
| hsa-miR-488 | 3,29 | 1,00 | 3,29 | 1,19 | 0,32 | 1,00 | 0,25 | 1,00 | 0,56 |
| hsa-miR-488S | 60,56 | 50,54 | 1,20 | 0,18 | 0,99 | 1,00 | 0,82 | 1,00 | 0,50 |
| hsa-miR-489 | 172,92 | 130,64 | 1,32 | 0,28 | 0,38 | 1,00 | 0,22 | 1,00 | 0,56 |
| hsa-miR-490-3p | 90,13 | 91,36 | 0,99 | -0,01 | 0,69 | 1,00 | 0,70 | 1,00 | 0,47 |
| hsa-miR-490-5p | 106,16 | 106,09 | 1,00 | 0,00 | 0,59 | 1,00 | 0,66 | 1,00 | 0,53 |
| hsa-miR-491-3p | 57,58 | 39,92 | 1,44 | 0,37 | 0,72 | 1,00 | 0,80 | 1,00 | 0,52 |
| hsa-miR-491-5p | 79,09 | 94,77 | 0,83 | -0,18 | 0,58 | 1,00 | 0,82 | 1,00 | 0,46 |
| hsa-miR-492 | 70,51 | 73,70 | 0,96 | -0,04 | 0,36 | 1,00 | 0,17 | 1,00 | 0,44 |
| hsa-miR-493 | 40,39 | 30,08 | 1,34 | 0,29 | 0,76 | 1,00 | 0,82 | 1,00 | 0,48 |
| hsa-miR-493S | 25,93 | 22,44 | 1,16 | 0,14 | 0,67 | 1,00 | 0,91 | 1,00 | 0,53 |
| hsa-miR-494 | 69,82 | 69,87 | 1,00 | 0,00 | 0,88 | 1,00 | 0,92 | 1,00 | 0,49 |
| hsa-miR-495 | 84,09 | 91,93 | 0,91 | -0,09 | 0,55 | 1,00 | 0,41 | 1,00 | 0,46 |
| hsa-miR-496 | 77,79 | 109,30 | 0,71 | -0,34 | 0,12 | 1,00 | 0,20 | 1,00 | 0,40 |
| hsa-miR-497 | 154,29 | 179,94 | 0,86 | -0,15 | 0,72 | 1,00 | 0,77 | 1,00 | 0,48 |
| hsa-miR-497S | 96,72 | 132,00 | 0,73 | -0,31 | 0,16 | 1,00 | 0,10 | 1,00 | 0,41 |
| hsa-miR-498 | 36,81 | 48,88 | 0,75 | -0,28 | 0,31 | 1,00 | 0,52 | 1,00 | 0,43 |
| hsa-miR-499-3p | 119,96 | 129,57 | 0,93 | -0,08 | 0,82 | 1,00 | 0,46 | 1,00 | 0,51 |
| hsa-miR-499-5p | 18,49 | 19,10 | 0,97 | -0,03 | 0,95 | 1,00 | 0,91 | 1,00 | 0,50 |
| hsa-miR-500 | 148,29 | 170,46 | 0,87 | -0,14 | 0,94 | 1,00 | 0,84 | 1,00 | 0,49 |
| hsa-miR-500S | 209,48 | 203,82 | 1,03 | 0,03 | 0,51 | 1,00 | 0,61 | 1,00 | 0,54 |
| hsa-miR-501-3p | 238,92 | 305,34 | 0,78 | -0,25 | 0,10 | 1,00 | 0,25 | 1,00 | 0,39 |
| hsa-miR-501-5p | 51,28 | 59,09 | 0,87 | -0,14 | 0,72 | 1,00 | 0,96 | 1,00 | 0,48 |
| hsa-miR-502-3p | 580,35 | 565,98 | 1,03 | 0,03 | 0,51 | 1,00 | 0,29 | 1,00 | 0,46 |
| hsa-miR-502-5p | 13,44 | 15,54 | 0,87 | -0,14 | 0,88 | 1,00 | 0,38 | 1,00 | 0,51 |
| hsa-miR-503 | 249,10 | 240,31 | 1,04 | 0,04 | 0,57 | 1,00 | 0,51 | 1,00 | 0,54 |
| hsa-miR-504 | 24,02 | 39,95 | 0,60 | -0,51 | 0,16 | 1,00 | 0,14 | 1,00 | 0,41 |
| hsa-miR-505 | 27,33 | 27,75 | 0,98 | -0,02 | 0,49 | 1,00 | 0,37 | 1,00 | 0,54 |
| hsa-miR-505S | 136,97 | 152,52 | 0,90 | -0,11 | 0,40 | 1,00 | 0,58 | 1,00 | 0,45 |
| hsa-miR-506 | 49,65 | 59,17 | 0,84 | -0,18 | 0,70 | 1,00 | 0,96 | 1,00 | 0,47 |
| hsa-miR-507 | 21,07 | 25,31 | 0,83 | -0,18 | 0,77 | 1,00 | 0,61 | 1,00 | 0,48 |
| hsa-miR-508-3p | 32,26 | 11,95 | 2,70 | 0,99 | 0,86 | 1,00 | 0,89 | 1,00 | 0,51 |
| hsa-miR-508-5p | 111,62 | 113,36 | 0,98 | -0,02 | 0,61 | 1,00 | 0,49 | 1,00 | 0,47 |
| hsa-miR-509-3-5p | 201,24 | 186,72 | 1,08 | 0,07 | 0,40 | 1,00 | 0,82 | 1,00 | 0,56 |
| hsa-miR-509-3p | 16,82 | 1,00 | 16,82 | 2,82 | 0,10 | 1,00 | 0,04 | 1,00 | 0,60 |
| hsa-miR-509-5p | 202,46 | 185,18 | 1,09 | 0,09 | 0,99 | 1,00 | 0,64 | 1,00 | 0,50 |
| hsa-miR-510 | 96,35 | 97,19 | 0,99 | -0,01 | 0,88 | 1,00 | 0,43 | 1,00 | 0,51 |
| hsa-miR-511 | 18,63 | 28,03 | 0,66 | -0,41 | 0,14 | 1,00 | 0,34 | 1,00 | 0,40 |
| hsa-miR-512-3p | 1,00 | 19,23 | 0,05 | -2,96 | 0,15 | 1,00 | 0,57 | 1,00 | 0,41 |
| hsa-miR-512-5p | 57,96 | 60,09 | 0,96 | -0,04 | 0,99 | 1,00 | 0,98 | 1,00 | 0,50 |
| hsa-miR-513a-3p | 66,49 | 48,79 | 1,36 | 0,31 | 0,44 | 1,00 | 0,80 | 1,00 | 0,55 |
| hsa-miR-513a-5p | 52,19 | 36,02 | 1,45 | 0,37 | 0,08 | 1,00 | 0,02 | 1,00 | 0,61 |
| hsa-miR-513b | 25,86 | 30,74 | 0,84 | -0,17 | 0,99 | 1,00 | 0,44 | 1,00 | 0,50 |
| hsa-miR-513c | 24,95 | 17,04 | 1,46 | 0,38 | 0,73 | 1,00 | 0,52 | 1,00 | 0,52 |
| hsa-miR-514 | 34,84 | 64,13 | 0,54 | -0,61 | 0,11 | 1,00 | 0,32 | 1,00 | 0,40 |
| hsa-miR-515-3p | 1,00 | 16,46 | 0,06 | -2,80 | 0,47 | 1,00 | 0,81 | 1,00 | 0,46 |
| hsa-miR-515-5p | 156,29 | 121,71 | 1,28 | 0,25 | 0,45 | 1,00 | 0,89 | 1,00 | 0,55 |
| hsa-miR-516a-3p | 16,14 | 20,32 | 0,79 | -0,23 | 0,82 | 1,00 | 0,93 | 1,00 | 0,51 |
| hsa-miR-516a-5p | 144,29 | 158,43 | 0,91 | -0,09 | 0,71 | 1,00 | 0,93 | 1,00 | 0,52 |
| hsa-miR-516b | 31,75 | 38,51 | 0,82 | -0,19 | 0,76 | 1,00 | 0,46 | 1,00 | 0,52 |
| hsa-miR-516bS | 46,77 | 49,78 | 0,94 | -0,06 | 0,14 | 1,00 | 0,16 | 1,00 | 0,60 |
| hsa-miR-517a | 45,26 | 33,28 | 1,36 | 0,31 | 0,24 | 1,00 | 0,27 | 1,00 | 0,58 |
| hsa-miR-517b | 30,58 | 25,35 | 1,21 | 0,19 | 0,77 | 1,00 | 0,47 | 1,00 | 0,48 |
| hsa-miR-517c | 15,09 | 20,32 | 0,74 | -0,30 | 0,89 | 1,00 | 0,93 | 1,00 | 0,49 |
| hsa-miR-517S | 168,89 | 164,51 | 1,03 | 0,03 | 0,53 | 1,00 | 0,83 | 1,00 | 0,54 |
| hsa-miR-518a-3p | 31,75 | 40,96 | 0,78 | -0,25 | 0,75 | 1,00 | 0,93 | 1,00 | 0,48 |
| hsa-miR-518a-5p | 169,24 | 135,63 | 1,25 | 0,22 | 0,06 | 1,00 | 0,47 | 1,00 | 0,62 |
| hsa-miR-518b | 106,96 | 85,18 | 1,26 | 0,23 | 0,88 | 1,00 | 0,47 | 1,00 | 0,49 |
| hsa-miR-518c | 87,91 | 97,87 | 0,90 | -0,11 | 0,30 | 1,00 | 0,40 | 1,00 | 0,43 |
| hsa-miR-518cS | 58,33 | 48,04 | 1,21 | 0,19 | 0,74 | 1,00 | 0,91 | 1,00 | 0,52 |
| hsa-miR-518d-3p | 52,98 | 49,47 | 1,07 | 0,07 | 0,40 | 1,00 | 0,19 | 1,00 | 0,55 |
| hsa-miR-518d-5p | 110,53 | 113,63 | 0,97 | -0,03 | 0,56 | 1,00 | 0,24 | 1,00 | 0,54 |
| hsa-miR-518e | 77,19 | 80,69 | 0,96 | -0,04 | 0,91 | 1,00 | 0,65 | 1,00 | 0,51 |
| hsa-miR-518eS | 152,22 | 127,96 | 1,19 | 0,17 | 0,88 | 1,00 | 0,71 | 1,00 | 0,49 |
| hsa-miR-518f | 74,58 | 45,81 | 1,63 | 0,49 | 0,14 | 1,00 | 0,23 | 1,00 | 0,60 |
| hsa-miR-518fS | 121,90 | 89,92 | 1,36 | 0,30 | 0,17 | 1,00 | 0,21 | 1,00 | 0,59 |
| hsa-miR-519a | 12,07 | 21,23 | 0,57 | -0,56 | 0,59 | 1,00 | 0,96 | 1,00 | 0,47 |
| hsa-miR-519aS | 126,92 | 89,92 | 1,41 | 0,34 | 0,17 | 1,00 | 0,17 | 1,00 | 0,59 |
| hsa-miR-519b-3p | 39,67 | 44,54 | 0,89 | -0,12 | 0,85 | 1,00 | 0,69 | 1,00 | 0,51 |
| hsa-miR-519b-5p | 87,91 | 85,99 | 1,02 | 0,02 | 0,94 | 1,00 | 0,74 | 1,00 | 0,50 |
| hsa-miR-519c-3p | 21,27 | 32,48 | 0,65 | -0,42 | 0,86 | 1,00 | 0,63 | 1,00 | 0,51 |
| hsa-miR-519c-5p | 127,55 | 102,06 | 1,25 | 0,22 | 0,36 | 1,00 | 0,42 | 1,00 | 0,56 |
| hsa-miR-519d | 57,16 | 50,28 | 1,14 | 0,13 | 0,61 | 1,00 | 0,22 | 1,00 | 0,47 |
| hsa-miR-519e | 1,00 | 1,19 | 0,84 | -0,18 | 0,19 | 1,00 | 0,26 | 1,00 | 0,42 |
| hsa-miR-519eS | 35,35 | 25,03 | 1,41 | 0,35 | 0,90 | 1,00 | 0,29 | 1,00 | 0,51 |
| hsa-miR-520a-3p | 11,84 | 28,66 | 0,41 | -0,88 | 0,25 | 1,00 | 0,38 | 1,00 | 0,43 |
| hsa-miR-520a-5p | 96,72 | 69,99 | 1,38 | 0,32 | 0,19 | 1,00 | 0,66 | 1,00 | 0,59 |
| hsa-miR-520b | 29,16 | 31,52 | 0,93 | -0,08 | 0,90 | 1,00 | 0,81 | 1,00 | 0,49 |
| hsa-miR-520c-3p | 1,00 | 1,00 | 1,00 | 0,00 | 0,70 | 1,00 | 0,31 | 1,00 | 0,52 |
| hsa-miR-520c-5p | 125,30 | 128,03 | 0,98 | -0,02 | 0,78 | 1,00 | 0,62 | 1,00 | 0,52 |
| hsa-miR-520d-3p | 8,53 | 3,91 | 2,18 | 0,78 | 0,91 | 1,00 | 0,38 | 1,00 | 0,51 |
| hsa-miR-520d-5p | 40,28 | 37,93 | 1,06 | 0,06 | 0,70 | 1,00 | 0,57 | 1,00 | 0,48 |
| hsa-miR-520e | 1,00 | 3,68 | 0,27 | -1,30 | 0,33 | 1,00 | 0,72 | 1,00 | 0,44 |
| hsa-miR-520f | 1,00 | 1,00 | 1,00 | 0,00 | 0,92 | 1,00 | 0,93 | 1,00 | 0,49 |
| hsa-miR-520g | 45,72 | 58,01 | 0,79 | -0,24 | 0,17 | 1,00 | 0,11 | 1,00 | 0,41 |
| hsa-miR-520h | 22,60 | 27,74 | 0,81 | -0,20 | 0,29 | 1,00 | 0,56 | 1,00 | 0,43 |
| hsa-miR-521 | 74,02 | 82,30 | 0,90 | -0,11 | 0,73 | 1,00 | 0,57 | 1,00 | 0,48 |
| hsa-miR-522 | 9,37 | 5,82 | 1,61 | 0,48 | 0,74 | 1,00 | 0,96 | 1,00 | 0,52 |
| hsa-miR-522S | 93,40 | 83,37 | 1,12 | 0,11 | 0,94 | 1,00 | 0,71 | 1,00 | 0,51 |
| hsa-miR-523 | 116,50 | 126,39 | 0,92 | -0,08 | 0,33 | 1,00 | 0,39 | 1,00 | 0,44 |
| hsa-miR-523S | 132,42 | 127,14 | 1,04 | 0,04 | 0,56 | 1,00 | 0,57 | 1,00 | 0,54 |
| hsa-miR-524-3p | 15,88 | 20,04 | 0,79 | -0,23 | 0,77 | 1,00 | 0,96 | 1,00 | 0,48 |
| hsa-miR-524-5p | 51,89 | 49,36 | 1,05 | 0,05 | 0,35 | 1,00 | 0,21 | 1,00 | 0,44 |
| hsa-miR-525-3p | 62,56 | 56,84 | 1,10 | 0,10 | 0,81 | 1,00 | 0,42 | 1,00 | 0,52 |
| hsa-miR-525-5p | 88,91 | 98,13 | 0,91 | -0,10 | 0,46 | 1,00 | 0,27 | 1,00 | 0,45 |
| hsa-miR-526a | 100,91 | 81,59 | 1,24 | 0,21 | 0,94 | 1,00 | 0,80 | 1,00 | 0,51 |
| hsa-miR-526b | 39,44 | 30,81 | 1,28 | 0,25 | 0,75 | 1,00 | 0,40 | 1,00 | 0,48 |
| hsa-miR-526bS | 3,72 | 10,75 | 0,35 | -1,06 | 0,79 | 1,00 | 0,78 | 1,00 | 0,48 |
| hsa-miR-527 | 158,43 | 128,80 | 1,23 | 0,21 | 0,53 | 1,00 | 0,59 | 1,00 | 0,54 |
| hsa-miR-532-3p | 3004,33 | 2809,70 | 1,07 | 0,07 | 0,80 | 1,00 | 0,75 | 1,00 | 0,52 |
| hsa-miR-532-5p | 128,53 | 150,81 | 0,85 | -0,16 | 0,74 | 1,00 | 0,63 | 1,00 | 0,48 |
| hsa-miR-539 | 21,18 | 18,37 | 1,15 | 0,14 | 0,75 | 1,00 | 0,73 | 1,00 | 0,52 |
| hsa-miR-541 | 57,16 | 56,12 | 1,02 | 0,02 | 0,83 | 1,00 | 0,70 | 1,00 | 0,51 |
| hsa-miR-541S | 1,00 | 1,00 | 1,00 | 0,00 | 0,59 | 1,00 | 0,91 | 1,00 | 0,47 |
| hsa-miR-542-3p | 74,58 | 83,31 | 0,90 | -0,11 | 0,31 | 1,00 | 0,54 | 1,00 | 0,43 |
| hsa-miR-542-5p | 74,60 | 98,13 | 0,76 | -0,27 | 0,44 | 1,00 | 0,42 | 1,00 | 0,45 |
| hsa-miR-543 | 46,21 | 42,86 | 1,08 | 0,08 | 0,57 | 1,00 | 0,49 | 1,00 | 0,54 |
| hsa-miR-544 | 36,28 | 55,39 | 0,66 | -0,42 | 0,23 | 1,00 | 0,35 | 1,00 | 0,42 |
| hsa-miR-545 | 94,35 | 99,25 | 0,95 | -0,05 | 0,98 | 1,00 | 0,53 | 1,00 | 0,50 |
| hsa-miR-545S | 20,56 | 10,11 | 2,03 | 0,71 | 0,74 | 1,00 | 0,45 | 1,00 | 0,52 |
| hsa-miR-548a-3p | 62,56 | 72,11 | 0,87 | -0,14 | 0,53 | 1,00 | 0,46 | 1,00 | 0,46 |
| hsa-miR-548a-5p | 17,02 | 11,31 | 1,51 | 0,41 | 0,79 | 1,00 | 0,57 | 1,00 | 0,52 |
| hsa-miR-548b-3p | 42,11 | 32,15 | 1,31 | 0,27 | 0,17 | 1,00 | 0,12 | 1,00 | 0,59 |
| hsa-miR-548b-5p | 1,00 | 11,29 | 0,09 | -2,42 | 0,94 | 1,00 | 0,55 | 1,00 | 0,50 |
| hsa-miR-548c-3p | 45,26 | 40,32 | 1,12 | 0,12 | 0,90 | 1,00 | 0,81 | 1,00 | 0,51 |
| hsa-miR-548c-5p | 18,49 | 21,84 | 0,85 | -0,17 | 0,99 | 1,00 | 0,87 | 1,00 | 0,50 |
| hsa-miR-548d-3p | 24,02 | 24,53 | 0,98 | -0,02 | 0,63 | 1,00 | 0,24 | 1,00 | 0,47 |
| hsa-miR-548d-5p | 14,81 | 27,83 | 0,53 | -0,63 | 0,26 | 1,00 | 0,35 | 1,00 | 0,43 |
| hsa-miR-548e | 42,11 | 66,61 | 0,63 | -0,46 | 0,02 | 1,00 | 0,44 | 1,00 | 0,35 |
| hsa-miR-548f | 27,33 | 33,68 | 0,81 | -0,21 | 0,35 | 1,00 | 0,19 | 1,00 | 0,44 |
| hsa-miR-548g | 47,65 | 30,53 | 1,56 | 0,45 | 0,27 | 1,00 | 0,51 | 1,00 | 0,57 |
| hsa-miR-548h | 16,82 | 19,30 | 0,87 | -0,14 | 0,88 | 1,00 | 0,60 | 1,00 | 0,49 |
| hsa-miR-548i | 1,00 | 2,36 | 0,42 | -0,86 | 0,81 | 1,00 | 0,49 | 1,00 | 0,48 |
| hsa-miR-548j | 1,39 | 1,00 | 1,39 | 0,33 | 0,78 | 1,00 | 0,30 | 1,00 | 0,48 |
| hsa-miR-548k | 21,07 | 20,73 | 1,02 | 0,02 | 0,70 | 1,00 | 0,55 | 1,00 | 0,48 |
| hsa-miR-548l | 1,00 | 1,00 | 1,00 | 0,00 | 0,94 | 1,00 | 0,59 | 1,00 | 0,50 |
| hsa-miR-548m | 1,00 | 1,52 | 0,66 | -0,42 | 1,00 | 1,00 | 0,54 | 1,00 | 0,50 |
| hsa-miR-548n | 40,98 | 27,29 | 1,50 | 0,41 | 0,30 | 1,00 | 0,19 | 1,00 | 0,57 |
| hsa-miR-548o | 125,65 | 105,74 | 1,19 | 0,17 | 0,48 | 1,00 | 0,47 | 1,00 | 0,55 |
| hsa-miR-548p | 127,55 | 113,90 | 1,12 | 0,11 | 0,79 | 1,00 | 0,86 | 1,00 | 0,52 |
| hsa-miR-549 | 62,36 | 62,18 | 1,00 | 0,00 | 0,42 | 1,00 | 0,63 | 1,00 | 0,55 |
| hsa-miR-550 | 98,57 | 120,70 | 0,82 | -0,20 | 0,18 | 1,00 | 0,22 | 1,00 | 0,41 |
| hsa-miR-550S | 334,57 | 313,92 | 1,07 | 0,06 | 0,98 | 1,00 | 0,69 | 1,00 | 0,50 |
| hsa-miR-551a | 17,02 | 29,57 | 0,58 | -0,55 | 0,22 | 1,00 | 0,23 | 1,00 | 0,42 |
| hsa-miR-551b | 60,04 | 50,96 | 1,18 | 0,16 | 0,95 | 1,00 | 0,57 | 1,00 | 0,50 |
| hsa-miR-551bS | 58,70 | 28,80 | 2,04 | 0,71 | 0,20 | 1,00 | 0,24 | 1,00 | 0,58 |
| hsa-miR-552 | 12,25 | 21,13 | 0,58 | -0,55 | 0,38 | 1,00 | 0,43 | 1,00 | 0,44 |
| hsa-miR-553 | 1,00 | 1,00 | 1,00 | 0,00 | 0,70 | 1,00 | 0,98 | 1,00 | 0,48 |
| hsa-miR-554 | 100,18 | 100,82 | 0,99 | -0,01 | 0,75 | 1,00 | 0,48 | 1,00 | 0,52 |
| hsa-miR-555 | 42,04 | 39,62 | 1,06 | 0,06 | 0,80 | 1,00 | 0,23 | 1,00 | 0,52 |
| hsa-miR-556-3p | 2,88 | 18,90 | 0,15 | -1,88 | 0,11 | 1,00 | 0,33 | 1,00 | 0,40 |
| hsa-miR-556-5p | 69,82 | 88,09 | 0,79 | -0,23 | 0,37 | 1,00 | 0,49 | 1,00 | 0,44 |
| hsa-miR-557 | 31,75 | 23,89 | 1,33 | 0,28 | 0,64 | 1,00 | 0,71 | 1,00 | 0,53 |
| hsa-miR-558 | 87,91 | 86,61 | 1,01 | 0,01 | 0,56 | 1,00 | 0,38 | 1,00 | 0,54 |
| hsa-miR-559 | 51,16 | 51,96 | 0,98 | -0,02 | 0,71 | 1,00 | 0,41 | 1,00 | 0,48 |
| hsa-miR-561 | 23,65 | 24,45 | 0,97 | -0,03 | 0,77 | 1,00 | 0,64 | 1,00 | 0,48 |
| hsa-miR-562 | 20,56 | 39,21 | 0,52 | -0,65 | 0,12 | 1,00 | 0,16 | 1,00 | 0,40 |
| hsa-miR-563 | 29,61 | 12,60 | 2,35 | 0,85 | 0,28 | 1,00 | 0,39 | 1,00 | 0,57 |
| hsa-miR-564 | 122,08 | 116,44 | 1,05 | 0,05 | 0,62 | 1,00 | 0,27 | 1,00 | 0,47 |
| hsa-miR-566 | 83,30 | 70,51 | 1,18 | 0,17 | 0,61 | 1,00 | 0,95 | 1,00 | 0,53 |
| hsa-miR-567 | 80,88 | 59,17 | 1,37 | 0,31 | 0,57 | 1,00 | 0,53 | 1,00 | 0,54 |
| hsa-miR-568 | 68,46 | 44,28 | 1,55 | 0,44 | 0,34 | 1,00 | 0,94 | 1,00 | 0,56 |
| hsa-miR-569 | 3,95 | 23,30 | 0,17 | -1,78 | 0,07 | 1,00 | 0,64 | 1,00 | 0,38 |
| hsa-miR-570 | 80,88 | 80,75 | 1,00 | 0,00 | 0,73 | 1,00 | 0,60 | 1,00 | 0,48 |
| hsa-miR-571 | 58,33 | 66,22 | 0,88 | -0,13 | 0,36 | 1,00 | 0,60 | 1,00 | 0,44 |
| hsa-miR-572 | 87,96 | 67,30 | 1,31 | 0,27 | 0,07 | 1,00 | 0,18 | 1,00 | 0,62 |
| hsa-miR-573 | 47,40 | 39,79 | 1,19 | 0,18 | 0,42 | 1,00 | 0,42 | 1,00 | 0,55 |
| hsa-miR-574-3p | 1533,93 | 1907,68 | 0,80 | -0,22 | 0,96 | 1,00 | 0,93 | 1,00 | 0,50 |
| hsa-miR-574-5p | 1470,36 | 1567,57 | 0,94 | -0,06 | 0,40 | 1,00 | 0,20 | 1,00 | 0,56 |
| hsa-miR-575 | 129,20 | 149,98 | 0,86 | -0,15 | 0,63 | 1,00 | 0,41 | 1,00 | 0,47 |
| hsa-miR-576-3p | 5,40 | 16,82 | 0,32 | -1,14 | 0,53 | 1,00 | 0,46 | 1,00 | 0,46 |
| hsa-miR-576-5p | 28,68 | 5,88 | 4,88 | 1,59 | 0,52 | 1,00 | 0,70 | 1,00 | 0,54 |
| hsa-miR-577 | 21,50 | 35,60 | 0,60 | -0,50 | 0,52 | 1,00 | 0,37 | 1,00 | 0,46 |
| hsa-miR-578 | 20,86 | 6,54 | 3,19 | 1,16 | 0,39 | 1,00 | 0,76 | 1,00 | 0,55 |
| hsa-miR-579 | 24,02 | 30,67 | 0,78 | -0,24 | 0,87 | 1,00 | 0,29 | 1,00 | 0,49 |
| hsa-miR-580 | 36,28 | 31,36 | 1,16 | 0,15 | 0,63 | 1,00 | 0,29 | 1,00 | 0,53 |
| hsa-miR-581 | 20,86 | 23,31 | 0,89 | -0,11 | 0,75 | 1,00 | 0,79 | 1,00 | 0,52 |
| hsa-miR-582-3p | 70,51 | 65,96 | 1,07 | 0,07 | 0,62 | 1,00 | 0,61 | 1,00 | 0,53 |
| hsa-miR-582-5p | 38,77 | 35,52 | 1,09 | 0,09 | 0,69 | 1,00 | 0,29 | 1,00 | 0,53 |
| hsa-miR-583 | 45,88 | 73,61 | 0,62 | -0,47 | 0,33 | 1,00 | 0,15 | 1,00 | 0,44 |
| hsa-miR-584 | 59,44 | 39,92 | 1,49 | 0,40 | 0,74 | 1,00 | 0,83 | 1,00 | 0,52 |
| hsa-miR-585 | 1,00 | 2,36 | 0,42 | -0,86 | 0,74 | 1,00 | 0,60 | 1,00 | 0,48 |
| hsa-miR-586 | 39,67 | 39,04 | 1,02 | 0,02 | 0,74 | 1,00 | 0,67 | 1,00 | 0,48 |
| hsa-miR-587 | 56,35 | 50,42 | 1,12 | 0,11 | 0,87 | 1,00 | 0,80 | 1,00 | 0,49 |
| hsa-miR-588 | 125,65 | 109,50 | 1,15 | 0,14 | 0,48 | 1,00 | 0,99 | 1,00 | 0,55 |
| hsa-miR-589 | 58,84 | 53,83 | 1,09 | 0,09 | 0,64 | 1,00 | 1,00 | 1,00 | 0,53 |
| hsa-miR-589S | 88,16 | 82,35 | 1,07 | 0,07 | 0,48 | 1,00 | 0,52 | 1,00 | 0,45 |
| hsa-miR-590-3p | 8,53 | 19,18 | 0,44 | -0,81 | 0,35 | 1,00 | 0,54 | 1,00 | 0,44 |
| hsa-miR-590-5p | 96,35 | 122,09 | 0,79 | -0,24 | 0,47 | 1,00 | 0,62 | 1,00 | 0,45 |
| hsa-miR-591 | 40,89 | 25,18 | 1,62 | 0,49 | 0,08 | 1,00 | 0,50 | 1,00 | 0,61 |
| hsa-miR-592 | 51,89 | 49,67 | 1,04 | 0,04 | 0,99 | 1,00 | 0,36 | 1,00 | 0,50 |
| hsa-miR-593 | 4,70 | 8,68 | 0,54 | -0,61 | 0,72 | 1,00 | 0,27 | 1,00 | 0,52 |
| hsa-miR-593S | 278,52 | 265,18 | 1,05 | 0,05 | 0,75 | 1,00 | 0,90 | 1,00 | 0,48 |
| hsa-miR-595 | 255,04 | 223,87 | 1,14 | 0,13 | 0,67 | 1,00 | 0,23 | 1,00 | 0,53 |
| hsa-miR-596 | 96,25 | 86,05 | 1,12 | 0,11 | 0,66 | 1,00 | 0,23 | 1,00 | 0,53 |
| hsa-miR-597 | 88,91 | 72,42 | 1,23 | 0,21 | 0,20 | 1,00 | 0,25 | 1,00 | 0,58 |
| hsa-miR-598 | 67,72 | 79,51 | 0,85 | -0,16 | 0,14 | 1,00 | 0,14 | 1,00 | 0,40 |
| hsa-miR-599 | 51,89 | 49,82 | 1,04 | 0,04 | 1,00 | 1,00 | 0,89 | 1,00 | 0,50 |
| hsa-miR-600 | 77,19 | 80,86 | 0,95 | -0,05 | 0,92 | 1,00 | 0,54 | 1,00 | 0,51 |
| hsa-miR-601 | 42,11 | 31,96 | 1,32 | 0,28 | 0,62 | 1,00 | 0,42 | 1,00 | 0,53 |
| hsa-miR-602 | 115,09 | 131,90 | 0,87 | -0,14 | 0,43 | 1,00 | 0,30 | 1,00 | 0,45 |
| hsa-miR-603 | 150,85 | 155,62 | 0,97 | -0,03 | 0,68 | 1,00 | 0,79 | 1,00 | 0,47 |
| hsa-miR-604 | 67,30 | 53,34 | 1,26 | 0,23 | 0,62 | 1,00 | 0,90 | 1,00 | 0,53 |
| hsa-miR-605 | 27,33 | 26,77 | 1,02 | 0,02 | 0,82 | 1,00 | 0,57 | 1,00 | 0,49 |
| hsa-miR-606 | 81,44 | 88,46 | 0,92 | -0,08 | 0,17 | 1,00 | 0,06 | 1,00 | 0,41 |
| hsa-miR-607 | 83,67 | 81,44 | 1,03 | 0,03 | 0,80 | 1,00 | 0,81 | 1,00 | 0,52 |
| hsa-miR-608 | 178,50 | 155,81 | 1,15 | 0,14 | 0,91 | 1,00 | 0,76 | 1,00 | 0,51 |
| hsa-miR-609 | 1,00 | 1,00 | 1,00 | 0,00 | 0,54 | 1,00 | 0,39 | 1,00 | 0,54 |
| hsa-miR-610 | 32,60 | 32,36 | 1,01 | 0,01 | 0,38 | 1,00 | 0,51 | 1,00 | 0,56 |
| hsa-miR-611 | 96,14 | 105,69 | 0,91 | -0,09 | 0,94 | 1,00 | 0,54 | 1,00 | 0,49 |
| hsa-miR-612 | 82,02 | 69,93 | 1,17 | 0,16 | 0,92 | 1,00 | 0,85 | 1,00 | 0,51 |
| hsa-miR-613 | 12,07 | 11,41 | 1,06 | 0,06 | 0,79 | 1,00 | 0,84 | 1,00 | 0,48 |
| hsa-miR-614 | 37,75 | 32,93 | 1,15 | 0,14 | 0,86 | 1,00 | 0,63 | 1,00 | 0,51 |
| hsa-miR-615-3p | 41,88 | 25,35 | 1,65 | 0,50 | 0,23 | 1,00 | 0,18 | 1,00 | 0,58 |
| hsa-miR-615-5p | 48,88 | 33,60 | 1,45 | 0,37 | 0,39 | 1,00 | 0,38 | 1,00 | 0,56 |
| hsa-miR-616 | 31,44 | 11,31 | 2,78 | 1,02 | 0,03 | 1,00 | 0,03 | 1,00 | 0,63 |
| hsa-miR-616S | 55,81 | 59,45 | 0,94 | -0,06 | 0,52 | 1,00 | 0,56 | 1,00 | 0,46 |
| hsa-miR-617 | 16,82 | 29,32 | 0,57 | -0,56 | 0,18 | 1,00 | 0,15 | 1,00 | 0,41 |
| hsa-miR-618 | 24,26 | 20,54 | 1,18 | 0,17 | 0,81 | 1,00 | 0,55 | 1,00 | 0,52 |
| hsa-miR-619 | 77,79 | 75,98 | 1,02 | 0,02 | 0,91 | 1,00 | 0,69 | 1,00 | 0,51 |
| hsa-miR-620 | 14,81 | 17,04 | 0,87 | -0,14 | 0,83 | 1,00 | 0,71 | 1,00 | 0,49 |
| hsa-miR-621 | 266,92 | 224,07 | 1,19 | 0,18 | 0,61 | 1,00 | 0,93 | 1,00 | 0,53 |
| hsa-miR-622 | 88,74 | 95,60 | 0,93 | -0,07 | 0,96 | 1,00 | 0,72 | 1,00 | 0,50 |
| hsa-miR-623 | 71,07 | 57,82 | 1,23 | 0,21 | 0,38 | 1,00 | 0,21 | 1,00 | 0,56 |
| hsa-miR-624 | 82,77 | 77,97 | 1,06 | 0,06 | 0,86 | 1,00 | 0,92 | 1,00 | 0,51 |
| hsa-miR-624S | 39,16 | 39,04 | 1,00 | 0,00 | 0,86 | 1,00 | 0,98 | 1,00 | 0,51 |
| hsa-miR-625 | 78,26 | 109,27 | 0,72 | -0,33 | 0,25 | 1,00 | 0,24 | 1,00 | 0,43 |
| hsa-miR-625S | 233,52 | 220,02 | 1,06 | 0,06 | 0,61 | 1,00 | 0,25 | 1,00 | 0,53 |
| hsa-miR-626 | 30,14 | 35,13 | 0,86 | -0,15 | 0,59 | 1,00 | 0,87 | 1,00 | 0,47 |
| hsa-miR-627 | 163,75 | 133,41 | 1,23 | 0,20 | 0,48 | 1,00 | 0,89 | 1,00 | 0,55 |
| hsa-miR-628-3p | 129,59 | 108,81 | 1,19 | 0,17 | 0,86 | 1,00 | 0,92 | 1,00 | 0,49 |
| hsa-miR-628-5p | 70,49 | 90,04 | 0,78 | -0,24 | 0,46 | 1,00 | 0,43 | 1,00 | 0,45 |
| hsa-miR-629 | 57,47 | 60,84 | 0,94 | -0,06 | 0,93 | 1,00 | 0,64 | 1,00 | 0,49 |
| hsa-miR-629S | 118,00 | 155,31 | 0,76 | -0,27 | 0,42 | 1,00 | 0,98 | 1,00 | 0,45 |
| hsa-miR-630 | 59,54 | 63,16 | 0,94 | -0,06 | 0,75 | 1,00 | 0,51 | 1,00 | 0,48 |
| hsa-miR-631 | 153,75 | 140,96 | 1,09 | 0,09 | 0,55 | 1,00 | 0,65 | 1,00 | 0,54 |
| hsa-miR-632 | 24,02 | 38,46 | 0,62 | -0,47 | 0,51 | 1,00 | 0,52 | 1,00 | 0,46 |
| hsa-miR-633 | 76,63 | 106,35 | 0,72 | -0,33 | 0,24 | 1,00 | 0,29 | 1,00 | 0,42 |
| hsa-miR-634 | 109,07 | 153,98 | 0,71 | -0,34 | 0,28 | 1,00 | 0,93 | 1,00 | 0,43 |
| hsa-miR-635 | 101,33 | 96,76 | 1,05 | 0,05 | 0,50 | 1,00 | 0,54 | 1,00 | 0,46 |
| hsa-miR-636 | 114,56 | 129,88 | 0,88 | -0,13 | 0,57 | 1,00 | 0,41 | 1,00 | 0,46 |
| hsa-miR-637 | 40,28 | 18,54 | 2,17 | 0,78 | 0,69 | 1,00 | 0,40 | 1,00 | 0,53 |
| hsa-miR-638 | 270,31 | 239,65 | 1,13 | 0,12 | 0,38 | 1,00 | 0,23 | 1,00 | 0,56 |
| hsa-miR-639 | 57,33 | 59,97 | 0,96 | -0,05 | 0,97 | 1,00 | 0,39 | 1,00 | 0,50 |
| hsa-miR-640 | 101,63 | 101,06 | 1,01 | 0,01 | 0,63 | 1,00 | 0,97 | 1,00 | 0,53 |
| hsa-miR-641 | 45,40 | 66,28 | 0,68 | -0,38 | 0,19 | 1,00 | 0,31 | 1,00 | 0,41 |
| hsa-miR-642 | 51,16 | 32,16 | 1,59 | 0,46 | 0,47 | 1,00 | 0,50 | 1,00 | 0,55 |
| hsa-miR-643 | 52,53 | 64,88 | 0,81 | -0,21 | 0,18 | 1,00 | 0,54 | 1,00 | 0,41 |
| hsa-miR-644 | 6,77 | 29,60 | 0,23 | -1,47 | 0,05 | 1,00 | 0,25 | 1,00 | 0,38 |
| hsa-miR-645 | 74,02 | 56,04 | 1,32 | 0,28 | 0,54 | 1,00 | 0,79 | 1,00 | 0,54 |
| hsa-miR-646 | 214,27 | 211,43 | 1,01 | 0,01 | 0,83 | 1,00 | 0,99 | 1,00 | 0,51 |
| hsa-miR-647 | 34,21 | 59,35 | 0,58 | -0,55 | 0,21 | 1,00 | 0,22 | 1,00 | 0,42 |
| hsa-miR-648 | 27,33 | 39,05 | 0,70 | -0,36 | 0,27 | 1,00 | 0,38 | 1,00 | 0,43 |
| hsa-miR-649 | 24,02 | 20,43 | 1,18 | 0,16 | 0,65 | 1,00 | 0,47 | 1,00 | 0,53 |
| hsa-miR-650 | 137,68 | 159,70 | 0,86 | -0,15 | 0,14 | 1,00 | 0,09 | 1,00 | 0,40 |
| hsa-miR-651 | 20,28 | 25,61 | 0,79 | -0,23 | 0,62 | 1,00 | 0,66 | 1,00 | 0,47 |
| hsa-miR-652 | 1030,54 | 990,38 | 1,04 | 0,04 | 0,31 | 1,00 | 0,26 | 1,00 | 0,57 |
| hsa-miR-653 | 25,93 | 46,28 | 0,56 | -0,58 | 0,38 | 1,00 | 0,68 | 1,00 | 0,44 |
| hsa-miR-654-3p | 23,65 | 19,43 | 1,22 | 0,20 | 0,56 | 1,00 | 0,19 | 1,00 | 0,54 |
| hsa-miR-654-5p | 142,10 | 175,99 | 0,81 | -0,21 | 0,23 | 1,00 | 0,13 | 1,00 | 0,42 |
| hsa-miR-655 | 46,95 | 44,72 | 1,05 | 0,05 | 0,45 | 1,00 | 0,78 | 1,00 | 0,55 |
| hsa-miR-656 | 40,67 | 40,50 | 1,00 | 0,00 | 0,79 | 1,00 | 0,85 | 1,00 | 0,52 |
| hsa-miR-657 | 58,33 | 64,71 | 0,90 | -0,10 | 0,84 | 1,00 | 0,78 | 1,00 | 0,49 |
| hsa-miR-658 | 60,68 | 41,32 | 1,47 | 0,38 | 0,22 | 1,00 | 0,18 | 1,00 | 0,58 |
| hsa-miR-659 | 87,02 | 90,07 | 0,97 | -0,03 | 0,61 | 1,00 | 0,85 | 1,00 | 0,53 |
| hsa-miR-660 | 357,27 | 450,35 | 0,79 | -0,23 | 0,10 | 1,00 | 0,51 | 1,00 | 0,39 |
| hsa-miR-661 | 45,26 | 62,39 | 0,73 | -0,32 | 0,26 | 1,00 | 0,99 | 1,00 | 0,43 |
| hsa-miR-662 | 71,74 | 58,46 | 1,23 | 0,20 | 0,56 | 1,00 | 0,48 | 1,00 | 0,54 |
| hsa-miR-663 | 289,34 | 279,32 | 1,04 | 0,04 | 0,76 | 1,00 | 0,63 | 1,00 | 0,48 |
| hsa-miR-663b | 74,02 | 84,73 | 0,87 | -0,14 | 0,12 | 1,00 | 0,13 | 1,00 | 0,40 |
| hsa-miR-664 | 267,75 | 274,08 | 0,98 | -0,02 | 0,75 | 1,00 | 0,74 | 1,00 | 0,48 |
| hsa-miR-664S | 69,96 | 68,61 | 1,02 | 0,02 | 0,75 | 1,00 | 0,69 | 1,00 | 0,52 |
| hsa-miR-665 | 115,09 | 115,14 | 1,00 | 0,00 | 0,79 | 1,00 | 0,65 | 1,00 | 0,48 |
| hsa-miR-668 | 59,44 | 75,67 | 0,79 | -0,24 | 0,66 | 1,00 | 0,95 | 1,00 | 0,47 |
| hsa-miR-671-3p | 51,16 | 53,48 | 0,96 | -0,04 | 0,56 | 1,00 | 0,27 | 1,00 | 0,54 |
| hsa-miR-671-5p | 67,72 | 66,16 | 1,02 | 0,02 | 0,80 | 1,00 | 0,61 | 1,00 | 0,52 |
| hsa-miR-675 | 158,96 | 160,97 | 0,99 | -0,01 | 0,84 | 1,00 | 0,43 | 1,00 | 0,49 |
| hsa-miR-7 | 43,61 | 50,11 | 0,87 | -0,14 | 0,74 | 1,00 | 0,51 | 1,00 | 0,48 |
| hsa-miR-7-1S | 287,97 | 260,51 | 1,11 | 0,10 | 0,81 | 1,00 | 0,97 | 1,00 | 0,52 |
| hsa-miR-7-2S | 40,21 | 25,86 | 1,55 | 0,44 | 0,48 | 1,00 | 0,51 | 1,00 | 0,55 |
| hsa-miR-708 | 57,16 | 47,74 | 1,20 | 0,18 | 0,63 | 1,00 | 0,61 | 1,00 | 0,53 |
| hsa-miR-708S | 72,05 | 67,46 | 1,07 | 0,07 | 0,43 | 1,00 | 0,34 | 1,00 | 0,45 |
| hsa-miR-720 | 2295,07 | 2349,97 | 0,98 | -0,02 | 0,92 | 1,00 | 0,63 | 1,00 | 0,49 |
| hsa-miR-744 | 454,29 | 465,00 | 0,98 | -0,02 | 0,63 | 1,00 | 0,43 | 1,00 | 0,47 |
| hsa-miR-744S | 47,75 | 56,95 | 0,84 | -0,18 | 0,97 | 1,00 | 0,81 | 1,00 | 0,50 |
| hsa-miR-758 | 56,35 | 49,53 | 1,14 | 0,13 | 0,97 | 1,00 | 0,66 | 1,00 | 0,50 |
| hsa-miR-760 | 69,68 | 62,50 | 1,11 | 0,11 | 0,17 | 1,00 | 0,05 | 1,00 | 0,59 |
| hsa-miR-765 | 60,04 | 61,75 | 0,97 | -0,03 | 0,52 | 1,00 | 0,13 | 1,00 | 0,54 |
| hsa-miR-766 | 487,22 | 393,16 | 1,24 | 0,21 | 0,84 | 1,00 | 0,51 | 1,00 | 0,51 |
| hsa-miR-767-3p | 23,65 | 34,14 | 0,69 | -0,37 | 0,90 | 1,00 | 0,47 | 1,00 | 0,49 |
| hsa-miR-767-5p | 149,73 | 119,70 | 1,25 | 0,22 | 0,54 | 1,00 | 0,36 | 1,00 | 0,54 |
| hsa-miR-769-3p | 18,63 | 10,49 | 1,78 | 0,57 | 0,79 | 1,00 | 0,46 | 1,00 | 0,52 |
| hsa-miR-769-5p | 1,00 | 2,36 | 0,42 | -0,86 | 0,55 | 1,00 | 0,46 | 1,00 | 0,46 |
| hsa-miR-770-5p | 64,28 | 67,66 | 0,95 | -0,05 | 0,68 | 1,00 | 0,37 | 1,00 | 0,53 |
| hsa-miR-802 | 42,04 | 49,05 | 0,86 | -0,15 | 0,38 | 1,00 | 0,63 | 1,00 | 0,44 |
| hsa-miR-873 | 40,89 | 37,14 | 1,10 | 0,10 | 0,75 | 1,00 | 0,31 | 1,00 | 0,52 |
| hsa-miR-874 | 182,21 | 182,44 | 1,00 | 0,00 | 0,49 | 1,00 | 0,68 | 1,00 | 0,55 |
| hsa-miR-875-3p | 43,25 | 38,81 | 1,11 | 0,11 | 0,56 | 1,00 | 0,52 | 1,00 | 0,54 |
| hsa-miR-875-5p | 40,89 | 59,17 | 0,69 | -0,37 | 0,50 | 1,00 | 0,58 | 1,00 | 0,46 |
| hsa-miR-876-3p | 17,02 | 38,54 | 0,44 | -0,82 | 0,11 | 1,00 | 0,24 | 1,00 | 0,40 |
| hsa-miR-876-5p | 21,07 | 28,00 | 0,75 | -0,28 | 0,53 | 1,00 | 0,54 | 1,00 | 0,46 |
| hsa-miR-877 | 83,67 | 112,88 | 0,74 | -0,30 | 0,92 | 1,00 | 0,63 | 1,00 | 0,51 |
| hsa-miR-877S | 105,68 | 87,09 | 1,21 | 0,19 | 0,78 | 1,00 | 0,81 | 1,00 | 0,52 |
| hsa-miR-885-3p | 200,34 | 196,25 | 1,02 | 0,02 | 0,57 | 1,00 | 0,38 | 1,00 | 0,54 |
| hsa-miR-885-5p | 67,47 | 52,68 | 1,28 | 0,25 | 0,62 | 1,00 | 0,88 | 1,00 | 0,53 |
| hsa-miR-886-3p | 16,14 | 9,51 | 1,70 | 0,53 | 0,68 | 1,00 | 0,90 | 1,00 | 0,53 |
| hsa-miR-886-5p | 67,72 | 66,71 | 1,02 | 0,02 | 0,65 | 1,00 | 0,38 | 1,00 | 0,53 |
| hsa-miR-887 | 111,62 | 125,50 | 0,89 | -0,12 | 0,46 | 1,00 | 0,34 | 1,00 | 0,45 |
| hsa-miR-888 | 60,81 | 68,86 | 0,88 | -0,12 | 0,60 | 1,00 | 0,76 | 1,00 | 0,47 |
| hsa-miR-888S | 42,46 | 43,79 | 0,97 | -0,03 | 0,71 | 1,00 | 0,68 | 1,00 | 0,48 |
| hsa-miR-889 | 31,44 | 22,57 | 1,39 | 0,33 | 0,80 | 1,00 | 0,30 | 1,00 | 0,52 |
| hsa-miR-890 | 21,07 | 9,32 | 2,26 | 0,81 | 0,16 | 1,00 | 0,17 | 1,00 | 0,59 |
| hsa-miR-891a | 91,40 | 103,90 | 0,88 | -0,13 | 0,59 | 1,00 | 0,78 | 1,00 | 0,47 |
| hsa-miR-891b | 81,04 | 77,37 | 1,05 | 0,05 | 0,93 | 1,00 | 0,48 | 1,00 | 0,49 |
| hsa-miR-892a | 12,07 | 1,00 | 12,07 | 2,49 | 0,53 | 1,00 | 0,95 | 1,00 | 0,54 |
| hsa-miR-892b | 40,98 | 62,16 | 0,66 | -0,42 | 0,36 | 1,00 | 0,48 | 1,00 | 0,44 |
| hsa-miR-9 | 22,60 | 7,56 | 2,99 | 1,09 | 0,56 | 1,00 | 0,35 | 1,00 | 0,54 |
| hsa-miR-920 | 58,61 | 44,67 | 1,31 | 0,27 | 0,94 | 1,00 | 0,93 | 1,00 | 0,49 |
| hsa-miR-921 | 63,19 | 61,91 | 1,02 | 0,02 | 0,82 | 1,00 | 0,42 | 1,00 | 0,51 |
| hsa-miR-922 | 116,34 | 103,98 | 1,12 | 0,11 | 0,66 | 1,00 | 0,78 | 1,00 | 0,53 |
| hsa-miR-924 | 20,56 | 28,03 | 0,73 | -0,31 | 0,25 | 1,00 | 0,58 | 1,00 | 0,43 |
| hsa-miR-92a | 12702,71 | 11503,91 | 1,10 | 0,10 | 0,38 | 1,00 | 0,46 | 1,00 | 0,56 |
| hsa-miR-92a-1S | 25,30 | 42,14 | 0,60 | -0,51 | 0,33 | 1,00 | 0,31 | 1,00 | 0,44 |
| hsa-miR-92a-2S | 27,33 | 46,96 | 0,58 | -0,54 | 0,74 | 1,00 | 0,61 | 1,00 | 0,48 |
| hsa-miR-92b | 225,80 | 210,38 | 1,07 | 0,07 | 0,92 | 1,00 | 0,86 | 1,00 | 0,51 |
| hsa-miR-92bS | 136,51 | 155,04 | 0,88 | -0,13 | 0,95 | 1,00 | 0,82 | 1,00 | 0,50 |
| hsa-miR-93 | 3312,49 | 3154,75 | 1,05 | 0,05 | 0,89 | 1,00 | 0,75 | 1,00 | 0,51 |
| hsa-miR-933 | 174,04 | 154,32 | 1,13 | 0,12 | 0,47 | 1,00 | 0,35 | 1,00 | 0,55 |
| hsa-miR-934 | 91,56 | 80,05 | 1,14 | 0,13 | 0,27 | 1,00 | 0,48 | 1,00 | 0,57 |
| hsa-miR-935 | 12,75 | 34,36 | 0,37 | -0,99 | 0,05 | 1,00 | 0,04 | 1,00 | 0,37 |
| hsa-miR-936 | 60,81 | 56,07 | 1,08 | 0,08 | 0,55 | 1,00 | 0,36 | 1,00 | 0,54 |
| hsa-miR-937 | 69,96 | 88,67 | 0,79 | -0,24 | 0,50 | 1,00 | 0,99 | 1,00 | 0,46 |
| hsa-miR-938 | 96,42 | 93,02 | 1,04 | 0,04 | 0,65 | 1,00 | 0,68 | 1,00 | 0,53 |
| hsa-miR-939 | 77,19 | 65,17 | 1,18 | 0,17 | 0,64 | 1,00 | 0,80 | 1,00 | 0,53 |
| hsa-miR-93S | 1030,54 | 1014,75 | 1,02 | 0,02 | 0,70 | 1,00 | 0,46 | 1,00 | 0,53 |
| hsa-miR-940 | 215,62 | 197,69 | 1,09 | 0,09 | 0,59 | 1,00 | 0,71 | 1,00 | 0,46 |
| hsa-miR-941 | 115,69 | 141,27 | 0,82 | -0,20 | 0,63 | 1,00 | 0,82 | 1,00 | 0,47 |
| hsa-miR-942 | 45,56 | 49,70 | 0,92 | -0,09 | 0,58 | 1,00 | 0,89 | 1,00 | 0,46 |
| hsa-miR-943 | 56,35 | 42,02 | 1,34 | 0,29 | 0,10 | 1,00 | 0,04 | 1,00 | 0,61 |
| hsa-miR-944 | 35,35 | 36,17 | 0,98 | -0,02 | 0,48 | 1,00 | 0,25 | 1,00 | 0,45 |
| hsa-miR-95 | 12,07 | 28,80 | 0,42 | -0,87 | 0,21 | 1,00 | 0,58 | 1,00 | 0,42 |
| hsa-miR-96 | 171,04 | 184,45 | 0,93 | -0,08 | 0,47 | 1,00 | 0,20 | 1,00 | 0,45 |
| hsa-miR-96S | 158,82 | 136,59 | 1,16 | 0,15 | 0,68 | 1,00 | 0,97 | 1,00 | 0,53 |
| hsa-miR-98 | 77,79 | 51,88 | 1,50 | 0,41 | 0,13 | 1,00 | 0,11 | 1,00 | 0,60 |
| hsa-miR-99a | 119,40 | 84,89 | 1,41 | 0,34 | 0,48 | 1,00 | 0,53 | 1,00 | 0,55 |
| hsa-miR-99aS | 37,02 | 55,16 | 0,67 | -0,40 | 0,18 | 1,00 | 0,39 | 1,00 | 0,41 |
| hsa-miR-99b | 124,39 | 153,34 | 0,81 | -0,21 | 0,37 | 1,00 | 0,47 | 1,00 | 0,44 |
| hsa-miR-99bS | 78,56 | 71,75 | 1,09 | 0,09 | 0,86 | 1,00 | 0,37 | 1,00 | 0,51 |
| hsa-miR-9S | 103,72 | 90,46 | 1,15 | 0,14 | 0,47 | 1,00 | 0,88 | 1,00 | 0,55 |

Significant miRNAs normal vs. cancer in tissue:

|  | **median normal** | **median PDAC** | **qmedian** | **logqmedian** | **wmw_raw p** | **wmw_adj p** | **ttest_raw p** | **ttest_adj p** | **AUC** |
| --- | --- | --- | --- | --- | --- | --- | --- | --- | --- |
| hsa-miR-31_PM | 34,91 | 373,22 | 0,09 | -2,37 | 0,00 | 0,00 | 0,00 | 0,000 | 0,13 |
| hsa-miR-181a_PM | 219,86 | 2163,39 | 0,10 | -2,29 | 0,00 | 0,00 | 0,00 | 0,000 | 0,04 |
| hsa-miR-155_PM | 90,83 | 768,43 | 0,12 | -2,14 | 0,00 | 0,00 | 0,00 | 0,000 | 0,04 |
| hsa-miR-181c_PM | 106,75 | 705,79 | 0,15 | -1,89 | 0,00 | 0,00 | 0,00 | 0,000 | 0,05 |
| hsa-miR-214_PM | 241,87 | 1591,71 | 0,15 | -1,88 | 0,00 | 0,00 | 0,00 | 0,000 | 0,03 |
| hsa-miR-150_PM | 145,41 | 843,19 | 0,17 | -1,76 | 0,00 | 0,00 | 0,00 | 0,000 | 0,07 |
| hsa-miR-222_PM | 310,67 | 1418,53 | 0,22 | -1,52 | 0,00 | 0,00 | 0,00 | 0,000 | 0,07 |
| hsa-miR-181b_PM | 172,65 | 719,03 | 0,24 | -1,43 | 0,00 | 0,00 | 0,00 | 0,000 | 0,07 |
| hsa-miR-145_PM | 700,57 | 2905,49 | 0,24 | -1,42 | 0,00 | 0,00 | 0,00 | 0,000 | 0,11 |
| hsa-miR-221_PM | 457,91 | 1633,62 | 0,28 | -1,27 | 0,00 | 0,00 | 0,00 | 0,000 | 0,13 |
| hsa-miR-181d_PM | 64,37 | 197,33 | 0,33 | -1,12 | 0,00 | 0,00 | 0,00 | 0,000 | 0,08 |
| hsa-miR-199a-3p_PM | 1040,60 | 2869,60 | 0,36 | -1,01 | 0,00 | 0,00 | 0,00 | 0,000 | 0,21 |
| hsa-miR-214S_PM | 31,94 | 86,96 | 0,37 | -1,00 | 0,00 | 0,00 | 0,00 | 0,000 | 0,12 |
| hsa-miR-146a_PM | 133,50 | 360,14 | 0,37 | -0,99 | 0,00 | 0,00 | 0,00 | 0,000 | 0,18 |
| hsa-miR-424S_PM | 16,77 | 44,81 | 0,37 | -0,98 | 0,00 | 0,00 | 0,00 | 0,000 | 0,22 |
| hsa-miR-125b_PM | 3691,13 | 9814,74 | 0,38 | -0,98 | 0,00 | 0,00 | 0,00 | 0,000 | 0,18 |
| hsa-miR-92a_PM | 283,93 | 738,89 | 0,38 | -0,96 | 0,00 | 0,00 | 0,00 | 0,000 | 0,20 |
| hsa-miR-132_PM | 129,65 | 336,38 | 0,39 | -0,95 | 0,00 | 0,00 | 0,00 | 0,000 | 0,14 |
| hsa-miR-199a-5p_PM | 197,39 | 494,37 | 0,40 | -0,92 | 0,00 | 0,00 | 0,00 | 0,000 | 0,21 |
| hsa-miR-15b_PM | 612,47 | 1530,60 | 0,40 | -0,92 | 0,00 | 0,00 | 0,00 | 0,000 | 0,17 |
| hsa-miR-93_PM | 383,83 | 955,32 | 0,40 | -0,91 | 0,00 | 0,00 | 0,00 | 0,000 | 0,19 |
| hsa-miR-210_PM | 57,83 | 132,71 | 0,44 | -0,83 | 0,00 | 0,00 | 0,00 | 0,000 | 0,21 |
| hsa-let-7i_PM | 1484,10 | 3343,15 | 0,44 | -0,81 | 0,00 | 0,00 | 0,00 | 0,000 | 0,17 |
| hsa-miR-107_PM | 1086,46 | 2386,68 | 0,46 | -0,79 | 0,00 | 0,00 | 0,00 | 0,000 | 0,20 |
| hsa-miR-103_PM | 1063,53 | 2322,21 | 0,46 | -0,78 | 0,00 | 0,00 | 0,00 | 0,000 | 0,24 |
| hsa-miR-125a-5p_PM | 672,30 | 1463,76 | 0,46 | -0,78 | 0,00 | 0,00 | 0,00 | 0,000 | 0,21 |
| hsa-miR-199b-3p_PM | 1462,20 | 3150,24 | 0,46 | -0,77 | 0,00 | 0,00 | 0,00 | 0,000 | 0,22 |
| hsa-miR-361-3p_PM | 30,80 | 64,10 | 0,48 | -0,73 | 0,00 | 0,00 | 0,00 | 0,000 | 0,17 |
| hsa-miR-127-3p_PM | 150,14 | 311,49 | 0,48 | -0,73 | 0,00 | 0,00 | 0,00 | 0,000 | 0,22 |
| hsa-miR-23a_PM | 3954,89 | 8066,58 | 0,49 | -0,71 | 0,00 | 0,01 | 0,00 | 0,000 | 0,26 |
| hsa-miR-100_PM | 784,15 | 1583,38 | 0,50 | -0,70 | 0,00 | 0,00 | 0,00 | 0,000 | 0,22 |
| hsa-miR-10a_PM | 225,68 | 455,33 | 0,50 | -0,70 | 0,00 | 0,01 | 0,00 | 0,000 | 0,26 |
| hsa-miR-106bS_PM | 24,08 | 48,57 | 0,50 | -0,70 | 0,00 | 0,00 | 0,00 | 0,000 | 0,14 |
| hsa-miR-1301_PM | 19,57 | 39,04 | 0,50 | -0,69 | 0,00 | 0,01 | 0,00 | 0,000 | 0,26 |
| hsa-miR-143S_PM | 13,37 | 26,58 | 0,50 | -0,69 | 0,00 | 0,00 | 0,00 | 0,000 | 0,21 |
| hsa-miR-21S_PM | 32,81 | 64,44 | 0,51 | -0,68 | 0,00 | 0,00 | 0,00 | 0,000 | 0,18 |
| hsa-miR-324-3p_PM | 45,73 | 89,16 | 0,51 | -0,67 | 0,00 | 0,00 | 0,00 | 0,000 | 0,16 |
| hsa-miR-423-3p_PM | 248,91 | 475,77 | 0,52 | -0,65 | 0,00 | 0,00 | 0,00 | 0,000 | 0,19 |
| hsa-miR-34bS_PM | 25,91 | 48,91 | 0,53 | -0,64 | 0,00 | 0,00 | 0,00 | 0,000 | 0,25 |
| hsa-miR-886-5p_PM | 59,21 | 111,55 | 0,53 | -0,63 | 0,00 | 0,00 | 0,00 | 0,000 | 0,20 |
| hsa-miR-342-5p_PM | 36,56 | 66,51 | 0,55 | -0,60 | 0,00 | 0,00 | 0,00 | 0,000 | 0,15 |
| hsa-miR-1307_PM | 35,30 | 63,50 | 0,56 | -0,59 | 0,00 | 0,00 | 0,00 | 0,000 | 0,23 |
| hsa-miR-320a_PM | 524,69 | 937,00 | 0,56 | -0,58 | 0,00 | 0,01 | 0,00 | 0,000 | 0,26 |
| hsa-miR-324-5p_PM | 83,06 | 146,39 | 0,57 | -0,57 | 0,00 | 0,00 | 0,00 | 0,000 | 0,12 |
| hsa-miR-331-3p_PM | 151,41 | 264,05 | 0,57 | -0,56 | 0,00 | 0,00 | 0,00 | 0,000 | 0,23 |
| hsa-miR-708_PM | 30,43 | 52,91 | 0,58 | -0,55 | 0,00 | 0,00 | 0,00 | 0,000 | 0,24 |
| hsa-miR-320d_PM | 431,79 | 746,05 | 0,58 | -0,55 | 0,00 | 0,01 | 0,00 | 0,000 | 0,26 |
| hsa-miR-320c_PM | 482,46 | 816,19 | 0,59 | -0,53 | 0,00 | 0,01 | 0,00 | 0,000 | 0,26 |
| hsa-miR-320b_PM | 484,49 | 803,87 | 0,60 | -0,51 | 0,00 | 0,01 | 0,00 | 0,000 | 0,26 |
| hsa-miR-501-5p_PM | 9,35 | 14,90 | 0,63 | -0,47 | 0,00 | 0,03 | 0,00 | 0,000 | 0,30 |
| hsa-miR-193a-5p_PM | 53,27 | 83,90 | 0,63 | -0,45 | 0,00 | 0,00 | 0,00 | 0,000 | 0,24 |
| hsa-miR-92b_PM | 76,90 | 120,46 | 0,64 | -0,45 | 0,00 | 0,00 | 0,00 | 0,000 | 0,22 |
| hsa-miR-744_PM | 63,65 | 99,71 | 0,64 | -0,45 | 0,00 | 0,00 | 0,00 | 0,000 | 0,24 |
| hsa-miR-652_PM | 99,07 | 153,76 | 0,64 | -0,44 | 0,00 | 0,00 | 0,00 | 0,000 | 0,20 |
| hsa-miR-503_PM | 50,19 | 75,96 | 0,66 | -0,41 | 0,00 | 0,01 | 0,00 | 0,000 | 0,26 |
| hsa-miR-532-3p_PM | 75,23 | 113,31 | 0,66 | -0,41 | 0,00 | 0,00 | 0,00 | 0,000 | 0,23 |
| hsa-miR-1290_PM | 34,81 | 52,03 | 0,67 | -0,40 | 0,00 | 0,00 | 0,00 | 0,000 | 0,25 |
| hsa-miR-874_PM | 61,48 | 91,84 | 0,67 | -0,40 | 0,00 | 0,00 | 0,00 | 0,000 | 0,25 |
| hsa-miR-342-3p_PM | 647,49 | 960,99 | 0,67 | -0,39 | 0,00 | 0,01 | 0,00 | 0,000 | 0,26 |
| hsa-miR-99b_PM | 871,46 | 1292,48 | 0,67 | -0,39 | 0,00 | 0,00 | 0,00 | 0,000 | 0,23 |
| hsa-miR-345_PM | 34,26 | 49,64 | 0,69 | -0,37 | 0,00 | 0,00 | 0,00 | 0,000 | 0,24 |
| hsa-miR-484_PM | 110,63 | 157,83 | 0,70 | -0,36 | 0,00 | 0,02 | 0,00 | 0,000 | 0,29 |
| hsa-miR-1246_PM | 123,69 | 163,71 | 0,76 | -0,28 | 0,00 | 0,00 | 0,00 | 0,000 | 0,25 |
| hsa-miR-628-5p_PM | 17,84 | 23,33 | 0,76 | -0,27 | 0,01 | 0,06 | 0,00 | 0,000 | 0,32 |
| hsa-miR-339-3p_PM | 107,14 | 58,14 | 1,84 | 0,61 | 0,00 | 0,00 | 0,00 | 0,000 | 0,83 |
| hsa-miR-30c-2S_PM | 84,29 | 44,15 | 1,91 | 0,65 | 0,00 | 0,00 | 0,00 | 0,000 | 0,86 |
| hsa-miR-30d_PM | 6898,00 | 3076,71 | 2,24 | 0,81 | 0,00 | 0,00 | 0,00 | 0,000 | 0,92 |
| hsa-miR-30aS_PM | 146,68 | 57,90 | 2,53 | 0,93 | 0,00 | 0,00 | 0,00 | 0,000 | 0,90 |
| hsa-miR-30a_PM | 3930,33 | 1423,22 | 2,76 | 1,02 | 0,00 | 0,00 | 0,00 | 0,000 | 0,89 |
| hsa-miR-30c_PM | 2661,66 | 854,03 | 3,12 | 1,14 | 0,00 | 0,00 | 0,00 | 0,000 | 0,92 |
| hsa-miR-30b_PM | 1923,94 | 589,57 | 3,26 | 1,18 | 0,00 | 0,00 | 0,00 | 0,000 | 0,91 |
| hsa-miR-375_PM | 8053,86 | 2005,31 | 4,02 | 1,39 | 0,00 | 0,00 | 0,00 | 0,000 | 0,89 |
| hsa-miR-182_PM | 337,57 | 74,39 | 4,54 | 1,51 | 0,00 | 0,00 | 0,00 | 0,000 | 0,92 |
| hsa-miR-130b_PM | 1212,50 | 137,37 | 8,83 | 2,18 | 0,00 | 0,00 | 0,00 | 0,000 | 0,98 |
| hsa-miR-148a_PM | 2568,17 | 200,99 | 12,78 | 2,55 | 0,00 | 0,00 | 0,00 | 0,000 | 0,99 |
| hsa-miR-200c_PM | 17155,85 | 4637,25 | 3,70 | 1,31 | 0,00 | 0,00 | 0,00 | 0,000 | 0,97 |
| hsa-miR-216a_PM | 4228,46 | 71,48 | 59,16 | 4,08 | 0,00 | 0,00 | 0,00 | 0,000 | 0,98 |
| hsa-miR-216b_PM | 7990,26 | 50,15 | 159,33 | 5,07 | 0,00 | 0,00 | 0,00 | 0,000 | 0,98 |
| hsa-miR-517a_PM | 19,68 | 36,85 | 0,53 | -0,63 | 0,00 | 0,02 | 0,00 | 0,001 | 0,29 |
| hsa-miR-1247_PM | 27,67 | 45,34 | 0,61 | -0,49 | 0,01 | 0,04 | 0,00 | 0,001 | 0,31 |
| hsa-miR-302b_PM | 7,92 | 12,65 | 0,63 | -0,47 | 0,01 | 0,06 | 0,00 | 0,001 | 0,32 |
| hsa-miR-620_PM | 10,91 | 17,03 | 0,64 | -0,44 | 0,01 | 0,03 | 0,00 | 0,001 | 0,30 |
| hsa-miR-136S_PM | 11,80 | 17,87 | 0,66 | -0,42 | 0,01 | 0,04 | 0,00 | 0,001 | 0,31 |
| hsa-miR-488_PM | 9,87 | 13,65 | 0,72 | -0,32 | 0,02 | 0,07 | 0,00 | 0,001 | 0,33 |
| hsa-miR-500S_PM | 63,96 | 87,77 | 0,73 | -0,32 | 0,00 | 0,02 | 0,00 | 0,001 | 0,30 |
| hsa-miR-200b_PM | 4083,42 | 1952,43 | 2,09 | 0,74 | 0,00 | 0,00 | 0,00 | 0,001 | 0,83 |
| hsa-miR-130a_PM | 721,52 | 338,49 | 2,13 | 0,76 | 0,00 | 0,00 | 0,00 | 0,001 | 0,81 |
| hsa-miR-148aS_PM | 82,95 | 36,23 | 2,29 | 0,83 | 0,00 | 0,00 | 0,00 | 0,001 | 0,84 |
| hsa-miR-493S_PM | 22,90 | 37,96 | 0,60 | -0,51 | 0,01 | 0,06 | 0,00 | 0,002 | 0,32 |
| hsa-miR-223S_PM | 13,77 | 21,97 | 0,63 | -0,47 | 0,01 | 0,04 | 0,00 | 0,002 | 0,31 |
| hsa-miR-409-3p_PM | 82,97 | 125,50 | 0,66 | -0,41 | 0,00 | 0,00 | 0,00 | 0,002 | 0,24 |
| hsa-miR-140-3p_PM | 400,05 | 571,83 | 0,70 | -0,36 | 0,02 | 0,08 | 0,00 | 0,002 | 0,33 |
| hsa-miR-502-3p_PM | 73,15 | 103,05 | 0,71 | -0,34 | 0,02 | 0,08 | 0,00 | 0,002 | 0,33 |
| hsa-miR-224_PM | 81,69 | 104,04 | 0,79 | -0,24 | 0,02 | 0,07 | 0,00 | 0,002 | 0,33 |
| hsa-miR-532-5p_PM | 105,41 | 133,67 | 0,79 | -0,24 | 0,00 | 0,02 | 0,00 | 0,002 | 0,29 |
| hsa-miR-379_PM | 134,94 | 75,80 | 1,78 | 0,58 | 0,00 | 0,00 | 0,00 | 0,002 | 0,81 |
| hsa-miR-551a_PM | 79,17 | 37,05 | 2,14 | 0,76 | 0,00 | 0,00 | 0,00 | 0,002 | 0,78 |
| hsa-miR-217_PM | 2244,87 | 66,87 | 33,57 | 3,51 | 0,00 | 0,00 | 0,00 | 0,002 | 0,98 |
| hsa-miR-425_PM | 241,76 | 306,55 | 0,79 | -0,24 | 0,02 | 0,07 | 0,00 | 0,003 | 0,33 |
| hsa-miR-99a_PM | 757,56 | 943,35 | 0,80 | -0,22 | 0,06 | 0,19 | 0,00 | 0,003 | 0,37 |
| hsa-miR-338-5p_PM | 65,64 | 32,56 | 2,02 | 0,70 | 0,00 | 0,00 | 0,00 | 0,003 | 0,79 |
| hsa-let-7e_PM | 2097,57 | 3255,68 | 0,64 | -0,44 | 0,06 | 0,19 | 0,00 | 0,004 | 0,37 |
| hsa-miR-193a-3p_PM | 63,96 | 38,08 | 1,68 | 0,52 | 0,00 | 0,00 | 0,00 | 0,004 | 0,78 |
| hsa-miR-365_PM | 213,03 | 108,79 | 1,96 | 0,67 | 0,00 | 0,00 | 0,00 | 0,004 | 0,86 |
| hsa-miR-215_PM | 376,81 | 101,62 | 3,71 | 1,31 | 0,00 | 0,00 | 0,00 | 0,004 | 0,94 |
| hsa-miR-192_PM | 1025,62 | 232,22 | 4,42 | 1,49 | 0,00 | 0,00 | 0,00 | 0,004 | 0,93 |
| hsa-miR-505_PM | 24,91 | 49,45 | 0,50 | -0,69 | 0,00 | 0,03 | 0,00 | 0,005 | 0,30 |
| hsa-miR-501-3p_PM | 60,83 | 74,83 | 0,81 | -0,21 | 0,01 | 0,06 | 0,00 | 0,005 | 0,32 |
| hsa-miR-411S_PM | 87,56 | 60,65 | 1,44 | 0,37 | 0,00 | 0,00 | 0,00 | 0,005 | 0,79 |
| hsa-miR-642_PM | 16,16 | 22,19 | 0,73 | -0,32 | 0,02 | 0,07 | 0,00 | 0,006 | 0,33 |
| hsa-miR-135a_PM | 10,73 | 13,45 | 0,80 | -0,23 | 0,07 | 0,21 | 0,00 | 0,006 | 0,37 |
| hsa-miR-432_PM | 49,94 | 62,55 | 0,80 | -0,23 | 0,06 | 0,18 | 0,00 | 0,006 | 0,36 |
| hsa-miR-30e_PM | 574,64 | 297,04 | 1,93 | 0,66 | 0,00 | 0,00 | 0,00 | 0,006 | 0,80 |
| hsa-miR-29c_PM | 657,63 | 264,56 | 2,49 | 0,91 | 0,00 | 0,00 | 0,00 | 0,006 | 0,82 |
| hsa-miR-1294_PM | 11,77 | 16,15 | 0,73 | -0,32 | 0,03 | 0,12 | 0,00 | 0,007 | 0,35 |
| hsa-miR-768-5p_PM | 4240,65 | 5200,94 | 0,82 | -0,20 | 0,19 | 0,41 | 0,00 | 0,007 | 0,41 |
| hsa-miR-23b_PM | 6638,49 | 9309,45 | 0,71 | -0,34 | 0,02 | 0,08 | 0,00 | 0,008 | 0,33 |
| hsa-miR-421_PM | 27,53 | 36,54 | 0,75 | -0,28 | 0,01 | 0,04 | 0,00 | 0,009 | 0,31 |
| hsa-miR-96S_PM | 10,74 | 13,53 | 0,79 | -0,23 | 0,03 | 0,10 | 0,00 | 0,009 | 0,34 |
| hsa-miR-27b_PM | 2277,29 | 1526,33 | 1,49 | 0,40 | 0,00 | 0,00 | 0,00 | 0,009 | 0,76 |
| hsa-miR-376a_PM | 154,18 | 98,45 | 1,57 | 0,45 | 0,00 | 0,00 | 0,00 | 0,009 | 0,78 |
| hsa-miR-26a_PM | 13601,13 | 8165,26 | 1,67 | 0,51 | 0,00 | 0,01 | 0,00 | 0,009 | 0,74 |
| hsa-miR-183_PM | 74,68 | 42,65 | 1,75 | 0,56 | 0,00 | 0,00 | 0,00 | 0,009 | 0,77 |
| hsa-miR-335_PM | 123,48 | 40,74 | 3,03 | 1,11 | 0,00 | 0,00 | 0,00 | 0,009 | 0,88 |
| hsa-miR-599_PM | 16,16 | 19,25 | 0,84 | -0,18 | 0,05 | 0,18 | 0,00 | 0,010 | 0,36 |
| hsa-miR-130bS_PM | 51,06 | 27,34 | 1,87 | 0,62 | 0,00 | 0,00 | 0,00 | 0,010 | 0,78 |
| hsa-miR-574-5p_PM | 13,15 | 22,14 | 0,59 | -0,52 | 0,01 | 0,04 | 0,00 | 0,013 | 0,31 |
| hsa-miR-590-3p_PM | 6,97 | 10,19 | 0,68 | -0,38 | 0,02 | 0,08 | 0,00 | 0,013 | 0,33 |
| hsa-miR-29cS_PM | 100,31 | 64,79 | 1,55 | 0,44 | 0,00 | 0,00 | 0,00 | 0,013 | 0,75 |
| hsa-miR-410_PM | 28,37 | 12,89 | 2,20 | 0,79 | 0,00 | 0,00 | 0,00 | 0,013 | 0,76 |
| hsa-miR-765_PM | 76,50 | 127,83 | 0,60 | -0,51 | 0,02 | 0,09 | 0,00 | 0,014 | 0,34 |
| hsa-miR-28-3p_PM | 177,79 | 227,10 | 0,78 | -0,24 | 0,05 | 0,18 | 0,00 | 0,015 | 0,36 |
| hsa-miR-659_PM | 56,24 | 39,75 | 1,41 | 0,35 | 0,00 | 0,01 | 0,00 | 0,015 | 0,73 |
| hsa-miR-30bS_PM | 54,68 | 36,75 | 1,49 | 0,40 | 0,00 | 0,01 | 0,00 | 0,015 | 0,73 |
| hsa-miR-422a_PM | 159,51 | 193,64 | 0,82 | -0,19 | 0,08 | 0,23 | 0,00 | 0,016 | 0,38 |
| hsa-miR-625_PM | 104,43 | 69,38 | 1,51 | 0,41 | 0,00 | 0,01 | 0,00 | 0,016 | 0,74 |
| hsa-miR-933_PM | 17,29 | 30,28 | 0,57 | -0,56 | 0,01 | 0,06 | 0,00 | 0,017 | 0,32 |
| hsa-miR-346_PM | 16,16 | 26,76 | 0,60 | -0,50 | 0,01 | 0,04 | 0,00 | 0,017 | 0,31 |
| hsa-miR-607_PM | 56,36 | 44,61 | 1,26 | 0,23 | 0,00 | 0,01 | 0,00 | 0,017 | 0,74 |
| hsa-miR-204_PM | 69,91 | 39,70 | 1,76 | 0,57 | 0,00 | 0,01 | 0,00 | 0,018 | 0,73 |
| hsa-miR-609_PM | 8,35 | 10,53 | 0,79 | -0,23 | 0,01 | 0,07 | 0,00 | 0,020 | 0,33 |
| hsa-miR-483-5p_PM | 432,12 | 620,10 | 0,70 | -0,36 | 0,17 | 0,39 | 0,00 | 0,021 | 0,40 |
| hsa-miR-647_PM | 16,31 | 17,74 | 0,92 | -0,08 | 0,14 | 0,35 | 0,00 | 0,022 | 0,40 |
| hsa-miR-191_PM | 2309,41 | 2468,13 | 0,94 | -0,07 | 0,13 | 0,34 | 0,00 | 0,022 | 0,39 |
| hsa-miR-767-5p_PM | 9,82 | 17,84 | 0,55 | -0,60 | 0,01 | 0,07 | 0,00 | 0,023 | 0,33 |
| hsa-miR-33b_PM | 9,52 | 15,01 | 0,63 | -0,46 | 0,02 | 0,08 | 0,00 | 0,023 | 0,33 |
| hsa-miR-34a_PM | 360,30 | 517,53 | 0,70 | -0,36 | 0,02 | 0,09 | 0,00 | 0,023 | 0,34 |
| hsa-miR-615-5p_PM | 15,11 | 20,24 | 0,75 | -0,29 | 0,06 | 0,18 | 0,00 | 0,025 | 0,36 |
| hsa-miR-184_PM | 116,46 | 84,58 | 1,38 | 0,32 | 0,00 | 0,01 | 0,00 | 0,025 | 0,73 |
| hsa-miR-141_PM | 154,38 | 51,56 | 2,99 | 1,10 | 0,00 | 0,00 | 0,00 | 0,026 | 0,87 |
| hsa-miR-1244_PM | 73,89 | 103,54 | 0,71 | -0,34 | 0,05 | 0,18 | 0,00 | 0,027 | 0,36 |
| hsa-miR-513a-3p_PM | 10,47 | 13,26 | 0,79 | -0,24 | 0,07 | 0,20 | 0,00 | 0,027 | 0,37 |
| hsa-miR-29b_PM | 156,56 | 63,93 | 2,45 | 0,90 | 0,00 | 0,00 | 0,00 | 0,027 | 0,80 |
| hsa-miR-632_PM | 12,75 | 25,14 | 0,51 | -0,68 | 0,01 | 0,06 | 0,01 | 0,029 | 0,32 |
| hsa-miR-1288_PM | 77,47 | 42,35 | 1,83 | 0,60 | 0,00 | 0,01 | 0,01 | 0,031 | 0,72 |
| hsa-miR-219-2-3p_PM | 26,97 | 15,89 | 1,70 | 0,53 | 0,00 | 0,01 | 0,01 | 0,033 | 0,74 |
| hsa-miR-509-3p_PM | 19,22 | 27,91 | 0,69 | -0,37 | 0,02 | 0,08 | 0,01 | 0,034 | 0,33 |
| hsa-miR-302f_PM | 16,87 | 20,21 | 0,83 | -0,18 | 0,11 | 0,29 | 0,01 | 0,034 | 0,39 |
| hsa-miR-200a_PM | 470,02 | 224,96 | 2,09 | 0,74 | 0,00 | 0,00 | 0,01 | 0,038 | 0,78 |
| hsa-miR-580_PM | 7,32 | 11,77 | 0,62 | -0,48 | 0,01 | 0,06 | 0,01 | 0,039 | 0,32 |
| hsa-miR-133b_PM | 24,26 | 38,50 | 0,63 | -0,46 | 0,01 | 0,06 | 0,01 | 0,039 | 0,32 |
| hsa-miR-10aS_PM | 38,48 | 50,26 | 0,77 | -0,27 | 0,06 | 0,18 | 0,01 | 0,039 | 0,36 |
| hsa-miR-518d-3p_PM | 25,37 | 32,92 | 0,77 | -0,26 | 0,09 | 0,27 | 0,01 | 0,040 | 0,38 |
| hsa-miR-520c-3p_PM | 8,76 | 10,82 | 0,81 | -0,21 | 0,05 | 0,16 | 0,01 | 0,040 | 0,36 |
| hsa-miR-27bS_PM | 44,12 | 31,32 | 1,41 | 0,34 | 0,00 | 0,02 | 0,01 | 0,040 | 0,71 |
| hsa-miR-584_PM | 29,03 | 35,33 | 0,82 | -0,20 | 0,12 | 0,32 | 0,01 | 0,042 | 0,39 |
| hsa-miR-504_PM | 22,95 | 34,50 | 0,67 | -0,41 | 0,00 | 0,02 | 0,01 | 0,044 | 0,29 |
| hsa-miR-1267_PM | 74,05 | 39,91 | 1,86 | 0,62 | 0,00 | 0,01 | 0,01 | 0,044 | 0,73 |
| hsa-miR-615-3p_PM | 12,65 | 16,74 | 0,76 | -0,28 | 0,03 | 0,11 | 0,01 | 0,045 | 0,34 |
| hsa-miR-145S_PM | 22,67 | 36,71 | 0,62 | -0,48 | 0,01 | 0,07 | 0,01 | 0,048 | 0,33 |
| hsa-miR-1248_PM | 16,16 | 24,57 | 0,66 | -0,42 | 0,06 | 0,19 | 0,01 | 0,049 | 0,37 |
| hsa-miR-664_PM | 48,28 | 33,31 | 1,45 | 0,37 | 0,00 | 0,02 | 0,01 | 0,049 | 0,71 |

Significant miRNAs normal vs. pancreatitis in tissue:

|  | **median normal** | **median pancreatitis** | **qmedian** | **logqmedian** | **wmw_raw p** | **wmw_adj p** | **ttest_raw p** | **ttest_adj p** | **AUC** |
| --- | --- | --- | --- | --- | --- | --- | --- | --- | --- |
| hsa-miR-200c_PM | 17155,85 | 8051,05 | 2,13 | 0,76 | 0,00 | 0,00 | 0,00 | 0,002 | 0,87 |
| hsa-miR-130b_PM | 1212,50 | 306,50 | 3,96 | 1,38 | 0,00 | 0,00 | 0,00 | 0,004 | 0,87 |
| hsa-miR-200b_PM | 4083,42 | 2274,82 | 1,80 | 0,59 | 0,00 | 0,00 | 0,00 | 0,005 | 0,89 |
| hsa-miR-182_PM | 337,57 | 88,74 | 3,80 | 1,34 | 0,00 | 0,00 | 0,00 | 0,005 | 0,95 |
| hsa-miR-183_PM | 74,68 | 37,78 | 1,98 | 0,68 | 0,00 | 0,00 | 0,00 | 0,006 | 0,85 |
| hsa-miR-26a_PM | 13601,13 | 7894,34 | 1,72 | 0,54 | 0,00 | 0,01 | 0,00 | 0,017 | 0,82 |
| hsa-miR-148a_PM | 2568,17 | 1195,99 | 2,15 | 0,76 | 0,00 | 0,00 | 0,00 | 0,022 | 0,92 |
| hsa-miR-193a-3p_PM | 63,96 | 37,06 | 1,73 | 0,55 | 0,00 | 0,01 | 0,00 | 0,023 | 0,82 |
| hsa-miR-335_PM | 123,48 | 50,09 | 2,47 | 0,90 | 0,00 | 0,00 | 0,00 | 0,023 | 0,84 |
| hsa-miR-100_PM | 784,15 | 1470,04 | 0,53 | -0,63 | 0,00 | 0,01 | 0,00 | 0,028 | 0,19 |
| hsa-miR-361-3p_PM | 30,80 | 55,29 | 0,56 | -0,59 | 0,00 | 0,01 | 0,00 | 0,028 | 0,19 |
| hsa-miR-30d_PM | 6898,00 | 4757,99 | 1,45 | 0,37 | 0,00 | 0,02 | 0,00 | 0,028 | 0,79 |
| hsa-miR-27bS_PM | 44,12 | 24,72 | 1,78 | 0,58 | 0,00 | 0,01 | 0,00 | 0,028 | 0,80 |
| hsa-miR-216b_PM | 7990,26 | 2658,42 | 3,01 | 1,10 | 0,00 | 0,01 | 0,00 | 0,028 | 0,82 |
| hsa-miR-215_PM | 376,81 | 116,75 | 3,23 | 1,17 | 0,00 | 0,00 | 0,00 | 0,028 | 0,96 |
| hsa-miR-192_PM | 1025,62 | 293,06 | 3,50 | 1,25 | 0,00 | 0,00 | 0,00 | 0,028 | 0,93 |
| hsa-miR-216a_PM | 4228,46 | 1196,17 | 3,54 | 1,26 | 0,00 | 0,01 | 0,00 | 0,031 | 0,80 |
| hsa-miR-551a_PM | 79,17 | 49,64 | 1,59 | 0,47 | 0,00 | 0,01 | 0,00 | 0,032 | 0,82 |
| hsa-miR-181d_PM | 64,37 | 136,44 | 0,47 | -0,75 | 0,00 | 0,02 | 0,00 | 0,033 | 0,21 |
| hsa-miR-194_PM | 777,64 | 267,27 | 2,91 | 1,07 | 0,00 | 0,00 | 0,00 | 0,033 | 0,91 |
| hsa-miR-339-3p_PM | 107,14 | 68,26 | 1,57 | 0,45 | 0,00 | 0,02 | 0,00 | 0,038 | 0,79 |
| hsa-miR-151-3p_PM | 249,36 | 149,18 | 1,67 | 0,51 | 0,00 | 0,03 | 0,00 | 0,038 | 0,78 |
| hsa-miR-514_PM | 14,85 | 31,91 | 0,47 | -0,76 | 0,00 | 0,05 | 0,00 | 0,042 | 0,23 |
| hsa-miR-181b_PM | 172,65 | 313,04 | 0,55 | -0,60 | 0,00 | 0,02 | 0,00 | 0,042 | 0,20 |
| hsa-miR-532-3p_PM | 75,23 | 117,61 | 0,64 | -0,45 | 0,00 | 0,01 | 0,00 | 0,042 | 0,20 |
| hsa-miR-29c_PM | 657,63 | 332,06 | 1,98 | 0,68 | 0,00 | 0,01 | 0,00 | 0,042 | 0,83 |
| hsa-miR-181c_PM | 106,75 | 295,35 | 0,36 | -1,02 | 0,00 | 0,05 | 0,00 | 0,045 | 0,23 |
| hsa-miR-30aS_PM | 146,68 | 112,59 | 1,30 | 0,26 | 0,00 | 0,07 | 0,00 | 0,045 | 0,75 |
| hsa-miR-217_PM | 2244,87 | 543,82 | 4,13 | 1,42 | 0,00 | 0,00 | 0,00 | 0,045 | 0,84 |
| hsa-miR-1206_PM | 19,89 | 41,07 | 0,48 | -0,73 | 0,00 | 0,01 | 0,00 | 0,046 | 0,20 |
| hsa-miR-214_PM | 241,87 | 718,11 | 0,34 | -1,09 | 0,00 | 0,00 | 0,00 | 0,048 | 0,16 |
| hsa-miR-486-5p_PM | 32,91 | 91,50 | 0,36 | -1,02 | 0,00 | 0,01 | 0,00 | 0,048 | 0,19 |
| hsa-miR-200a_PM | 470,02 | 205,12 | 2,29 | 0,83 | 0,00 | 0,00 | 0,00 | 0,048 | 0,84 |
| hsa-miR-7_PM | 85,79 | 18,21 | 4,71 | 1,55 | 0,00 | 0,00 | 0,00 | 0,048 | 0,88 |
| hsa-miR-130a_PM | 721,52 | 440,52 | 1,64 | 0,49 | 0,00 | 0,04 | 0,00 | 0,049 | 0,77 |

Significant miRNAs cancer vs. pancreatitis in tissue:

|  | **median PDAC** | **median pancreatitis** | **qmedian** | **logqmedian** | **wmw_raw p** | **wmw_adj p** | **ttest_raw p** | **ttest_adj p** | **AUC** |
| --- | --- | --- | --- | --- | --- | --- | --- | --- | --- |
| hsa-miR-99b_PM | 1292,48 | 967,16 | 1,34 | 0,29 | 0,00 | 0,01 | 0,00 | 0,000 | 0,73 |
| hsa-miR-181d_PM | 197,33 | 136,44 | 1,45 | 0,37 | 0,00 | 0,01 | 0,00 | 0,000 | 0,74 |
| hsa-miR-28-3p_PM | 227,10 | 153,66 | 1,48 | 0,39 | 0,00 | 0,01 | 0,00 | 0,000 | 0,74 |
| hsa-miR-24_PM | 3023,53 | 2037,82 | 1,48 | 0,39 | 0,00 | 0,01 | 0,00 | 0,000 | 0,74 |
| hsa-miR-649_PM | 25,71 | 16,32 | 1,57 | 0,45 | 0,00 | 0,04 | 0,00 | 0,000 | 0,70 |
| hsa-miR-10a_PM | 455,33 | 270,23 | 1,68 | 0,52 | 0,00 | 0,02 | 0,00 | 0,000 | 0,71 |
| hsa-miR-132_PM | 336,38 | 187,13 | 1,80 | 0,59 | 0,00 | 0,02 | 0,00 | 0,000 | 0,72 |
| hsa-miR-224_PM | 104,04 | 57,29 | 1,82 | 0,60 | 0,00 | 0,00 | 0,00 | 0,000 | 0,79 |
| hsa-miR-93_PM | 955,32 | 499,35 | 1,91 | 0,65 | 0,00 | 0,00 | 0,00 | 0,000 | 0,79 |
| hsa-miR-23b_PM | 9309,45 | 4780,66 | 1,95 | 0,67 | 0,00 | 0,01 | 0,00 | 0,000 | 0,74 |
| hsa-miR-324-5p_PM | 146,39 | 74,68 | 1,96 | 0,67 | 0,00 | 0,00 | 0,00 | 0,000 | 0,76 |
| hsa-miR-34a_PM | 517,53 | 262,44 | 1,97 | 0,68 | 0,00 | 0,00 | 0,00 | 0,000 | 0,77 |
| hsa-miR-106b_PM | 115,34 | 57,73 | 2,00 | 0,69 | 0,00 | 0,01 | 0,00 | 0,000 | 0,74 |
| hsa-miR-17_PM | 375,48 | 187,63 | 2,00 | 0,69 | 0,00 | 0,01 | 0,00 | 0,000 | 0,75 |
| hsa-miR-23a_PM | 8066,58 | 3991,61 | 2,02 | 0,70 | 0,00 | 0,00 | 0,00 | 0,000 | 0,76 |
| hsa-miR-146b-5p_PM | 766,55 | 378,28 | 2,03 | 0,71 | 0,00 | 0,02 | 0,00 | 0,000 | 0,71 |
| hsa-miR-103_PM | 2322,21 | 1079,29 | 2,15 | 0,77 | 0,00 | 0,00 | 0,00 | 0,000 | 0,79 |
| hsa-miR-210_PM | 132,71 | 60,70 | 2,19 | 0,78 | 0,00 | 0,00 | 0,00 | 0,000 | 0,76 |
| hsa-miR-107_PM | 2386,68 | 1051,38 | 2,27 | 0,82 | 0,00 | 0,00 | 0,00 | 0,000 | 0,81 |
| hsa-miR-181b_PM | 719,03 | 313,04 | 2,30 | 0,83 | 0,00 | 0,00 | 0,00 | 0,000 | 0,78 |
| hsa-miR-106a_PM | 374,47 | 162,13 | 2,31 | 0,84 | 0,00 | 0,01 | 0,00 | 0,000 | 0,74 |
| hsa-miR-181c_PM | 705,79 | 295,35 | 2,39 | 0,87 | 0,00 | 0,00 | 0,00 | 0,000 | 0,76 |
| hsa-miR-221_PM | 1633,62 | 511,29 | 3,20 | 1,16 | 0,00 | 0,00 | 0,00 | 0,000 | 0,82 |
| hsa-miR-222_PM | 1418,53 | 382,15 | 3,71 | 1,31 | 0,00 | 0,00 | 0,00 | 0,000 | 0,86 |
| hsa-miR-31_PM | 373,22 | 94,28 | 3,96 | 1,38 | 0,00 | 0,00 | 0,00 | 0,000 | 0,76 |
| hsa-miR-425_PM | 306,55 | 224,41 | 1,37 | 0,31 | 0,00 | 0,04 | 0,00 | 0,001 | 0,70 |
| hsa-miR-203_PM | 24,08 | 15,67 | 1,54 | 0,43 | 0,00 | 0,04 | 0,00 | 0,001 | 0,69 |
| hsa-miR-454_PM | 19,32 | 11,75 | 1,64 | 0,50 | 0,00 | 0,02 | 0,00 | 0,001 | 0,72 |
| hsa-miR-337-5p_PM | 42,62 | 24,08 | 1,77 | 0,57 | 0,00 | 0,01 | 0,00 | 0,001 | 0,74 |
| hsa-miR-423-3p_PM | 475,77 | 263,32 | 1,81 | 0,59 | 0,00 | 0,01 | 0,00 | 0,001 | 0,74 |
| hsa-miR-143_PM | 826,34 | 279,57 | 2,96 | 1,08 | 0,00 | 0,00 | 0,00 | 0,001 | 0,77 |
| hsa-miR-218-2S_PM | 470,14 | 825,40 | 0,57 | -0,56 | 0,00 | 0,00 | 0,00 | 0,002 | 0,22 |
| hsa-miR-345_PM | 49,64 | 34,32 | 1,45 | 0,37 | 0,00 | 0,01 | 0,00 | 0,002 | 0,75 |
| hsa-miR-15aS_PM | 18,46 | 11,25 | 1,64 | 0,50 | 0,00 | 0,01 | 0,00 | 0,002 | 0,73 |
| hsa-miR-20a_PM | 240,99 | 105,19 | 2,29 | 0,83 | 0,00 | 0,01 | 0,00 | 0,002 | 0,73 |
| hsa-miR-181a_PM | 2163,39 | 899,87 | 2,40 | 0,88 | 0,00 | 0,00 | 0,00 | 0,002 | 0,77 |
| hsa-miR-424S_PM | 44,81 | 27,86 | 1,61 | 0,48 | 0,00 | 0,04 | 0,00 | 0,003 | 0,69 |
| hsa-miR-185_PM | 114,56 | 63,95 | 1,79 | 0,58 | 0,00 | 0,03 | 0,00 | 0,003 | 0,71 |
| hsa-miR-518a-3p_PM | 16,82 | 8,33 | 2,02 | 0,70 | 0,00 | 0,01 | 0,00 | 0,003 | 0,74 |
| hsa-miR-548g_PM | 16,09 | 10,46 | 1,54 | 0,43 | 0,04 | 0,19 | 0,00 | 0,004 | 0,64 |
| hsa-miR-25_PM | 536,04 | 319,07 | 1,68 | 0,52 | 0,01 | 0,05 | 0,00 | 0,004 | 0,68 |
| hsa-miR-92a_PM | 738,89 | 434,33 | 1,70 | 0,53 | 0,00 | 0,03 | 0,00 | 0,004 | 0,70 |
| hsa-miR-323-3p_PM | 114,94 | 144,78 | 0,79 | -0,23 | 0,00 | 0,03 | 0,00 | 0,005 | 0,30 |
| hsa-miR-216a_PM | 71,48 | 1196,17 | 0,06 | -2,82 | 0,00 | 0,00 | 0,00 | 0,006 | 0,22 |
| hsa-miR-148a_PM | 200,99 | 1195,99 | 0,17 | -1,78 | 0,00 | 0,00 | 0,00 | 0,006 | 0,22 |
| hsa-miR-652_PM | 153,76 | 106,15 | 1,45 | 0,37 | 0,00 | 0,02 | 0,00 | 0,006 | 0,72 |
| hsa-miR-22S_PM | 56,32 | 34,95 | 1,61 | 0,48 | 0,00 | 0,04 | 0,00 | 0,006 | 0,69 |
| hsa-miR-216b_PM | 50,15 | 2658,42 | 0,02 | -3,97 | 0,00 | 0,00 | 0,00 | 0,007 | 0,20 |
| hsa-miR-330-3p_PM | 1174,77 | 1742,42 | 0,67 | -0,39 | 0,00 | 0,01 | 0,00 | 0,007 | 0,26 |
| hsa-miR-409-3p_PM | 125,50 | 89,90 | 1,40 | 0,33 | 0,00 | 0,03 | 0,00 | 0,007 | 0,70 |
| hsa-miR-20b_PM | 128,61 | 86,56 | 1,49 | 0,40 | 0,00 | 0,04 | 0,00 | 0,007 | 0,69 |
| hsa-miR-21_PM | 1453,48 | 702,28 | 2,07 | 0,73 | 0,00 | 0,03 | 0,00 | 0,007 | 0,71 |
| hsa-miR-924_PM | 27,55 | 16,32 | 1,69 | 0,52 | 0,01 | 0,05 | 0,00 | 0,008 | 0,69 |
| hsa-miR-92b_PM | 120,46 | 89,90 | 1,34 | 0,29 | 0,01 | 0,05 | 0,00 | 0,009 | 0,68 |
| hsa-miR-1307_PM | 63,50 | 44,66 | 1,42 | 0,35 | 0,00 | 0,02 | 0,00 | 0,009 | 0,72 |
| hsa-miR-422a_PM | 193,64 | 131,00 | 1,48 | 0,39 | 0,00 | 0,04 | 0,00 | 0,009 | 0,70 |
| hsa-miR-28-5p_PM | 289,12 | 219,38 | 1,32 | 0,28 | 0,02 | 0,14 | 0,00 | 0,010 | 0,65 |
| hsa-miR-591_PM | 12,48 | 8,36 | 1,49 | 0,40 | 0,01 | 0,06 | 0,00 | 0,010 | 0,68 |
| hsa-miR-194_PM | 417,85 | 267,27 | 1,56 | 0,45 | 0,03 | 0,15 | 0,00 | 0,010 | 0,65 |
| hsa-miR-574-5p_PM | 22,14 | 13,31 | 1,66 | 0,51 | 0,00 | 0,04 | 0,00 | 0,010 | 0,69 |
| hsa-miR-152_PM | 205,70 | 149,97 | 1,37 | 0,32 | 0,02 | 0,11 | 0,00 | 0,011 | 0,66 |
| hsa-miR-186_PM | 29,65 | 16,32 | 1,82 | 0,60 | 0,01 | 0,09 | 0,00 | 0,011 | 0,67 |
| hsa-miR-324-3p_PM | 89,16 | 68,38 | 1,30 | 0,27 | 0,01 | 0,07 | 0,00 | 0,013 | 0,67 |
| hsa-miR-145_PM | 2905,49 | 1091,54 | 2,66 | 0,98 | 0,00 | 0,00 | 0,00 | 0,014 | 0,77 |
| hsa-miR-331-3p_PM | 264,05 | 194,21 | 1,36 | 0,31 | 0,04 | 0,18 | 0,00 | 0,015 | 0,64 |
| hsa-miR-30d_PM | 3076,71 | 4757,99 | 0,65 | -0,44 | 0,00 | 0,01 | 0,00 | 0,016 | 0,27 |
| hsa-miR-487a_PM | 63,87 | 89,90 | 0,71 | -0,34 | 0,00 | 0,01 | 0,00 | 0,017 | 0,27 |
| hsa-miR-34bS_PM | 48,91 | 39,36 | 1,24 | 0,22 | 0,05 | 0,21 | 0,00 | 0,020 | 0,63 |
| hsa-miR-191_PM | 2468,13 | 2131,11 | 1,16 | 0,15 | 0,03 | 0,15 | 0,00 | 0,021 | 0,65 |
| hsa-miR-10aS_PM | 50,26 | 41,54 | 1,21 | 0,19 | 0,02 | 0,12 | 0,00 | 0,021 | 0,66 |
| hsa-miR-503_PM | 75,96 | 41,44 | 1,83 | 0,61 | 0,00 | 0,03 | 0,00 | 0,021 | 0,70 |
| hsa-miR-7_PM | 34,41 | 18,21 | 1,89 | 0,64 | 0,04 | 0,21 | 0,00 | 0,021 | 0,63 |
| hsa-miR-217_PM | 66,87 | 543,82 | 0,12 | -2,10 | 0,00 | 0,00 | 0,00 | 0,023 | 0,19 |
| hsa-miR-1248_PM | 24,57 | 16,52 | 1,49 | 0,40 | 0,08 | 0,30 | 0,00 | 0,024 | 0,62 |
| hsa-miR-484_PM | 157,83 | 99,01 | 1,59 | 0,47 | 0,00 | 0,03 | 0,00 | 0,024 | 0,70 |
| hsa-miR-499-5p_PM | 17,94 | 15,53 | 1,16 | 0,14 | 0,24 | 0,53 | 0,00 | 0,026 | 0,58 |
| hsa-miR-99bS_PM | 52,69 | 41,07 | 1,28 | 0,25 | 0,03 | 0,16 | 0,00 | 0,026 | 0,65 |
| hsa-miR-22_PM | 2126,68 | 1623,61 | 1,31 | 0,27 | 0,11 | 0,36 | 0,00 | 0,027 | 0,61 |
| hsa-miR-561_PM | 14,57 | 10,56 | 1,38 | 0,32 | 0,03 | 0,16 | 0,00 | 0,027 | 0,65 |
| hsa-miR-214S_PM | 86,96 | 60,42 | 1,44 | 0,36 | 0,01 | 0,05 | 0,00 | 0,028 | 0,68 |
| hsa-miR-145S_PM | 36,71 | 25,50 | 1,44 | 0,36 | 0,00 | 0,04 | 0,00 | 0,028 | 0,69 |
| hsa-let-7i_PM | 3343,15 | 2125,88 | 1,57 | 0,45 | 0,01 | 0,06 | 0,00 | 0,029 | 0,68 |
| hsa-miR-297_PM | 10,11 | 8,04 | 1,26 | 0,23 | 0,10 | 0,34 | 0,00 | 0,031 | 0,61 |
| hsa-miR-21S_PM | 64,44 | 47,46 | 1,36 | 0,31 | 0,01 | 0,08 | 0,00 | 0,031 | 0,67 |
| hsa-miR-1244_PM | 103,54 | 58,59 | 1,77 | 0,57 | 0,01 | 0,06 | 0,00 | 0,031 | 0,68 |
| hsa-miR-15b_PM | 1530,60 | 847,70 | 1,81 | 0,59 | 0,01 | 0,05 | 0,00 | 0,032 | 0,68 |
| hsa-miR-125a-5p_PM | 1463,76 | 921,80 | 1,59 | 0,46 | 0,02 | 0,10 | 0,00 | 0,033 | 0,66 |
| hsa-miR-634_PM | 233,90 | 406,76 | 0,58 | -0,55 | 0,00 | 0,01 | 0,00 | 0,037 | 0,28 |
| hsa-miR-923_PM | 16830,66 | 26201,05 | 0,64 | -0,44 | 0,00 | 0,03 | 0,00 | 0,037 | 0,29 |
| hsa-miR-34b_PM | 123,00 | 166,64 | 0,74 | -0,30 | 0,01 | 0,05 | 0,00 | 0,038 | 0,31 |
| hsa-miR-155_PM | 768,43 | 200,22 | 3,84 | 1,34 | 0,00 | 0,00 | 0,00 | 0,038 | 0,78 |
| hsa-miR-361-5p_PM | 416,52 | 305,51 | 1,36 | 0,31 | 0,09 | 0,31 | 0,00 | 0,040 | 0,61 |
| hsa-miR-375_PM | 2005,31 | 6030,88 | 0,33 | -1,10 | 0,00 | 0,03 | 0,00 | 0,041 | 0,29 |
| hsa-miR-1290_PM | 52,03 | 37,38 | 1,39 | 0,33 | 0,02 | 0,13 | 0,00 | 0,041 | 0,66 |
| hsa-miR-514_PM | 18,23 | 31,91 | 0,57 | -0,56 | 0,00 | 0,01 | 0,00 | 0,042 | 0,26 |
| hsa-miR-130b_PM | 137,37 | 306,50 | 0,45 | -0,80 | 0,00 | 0,01 | 0,00 | 0,043 | 0,27 |
| hsa-miR-30aS_PM | 57,90 | 112,59 | 0,51 | -0,67 | 0,00 | 0,03 | 0,01 | 0,046 | 0,29 |
| hsa-miR-299-5p_PM | 91,67 | 132,05 | 0,69 | -0,37 | 0,00 | 0,01 | 0,01 | 0,046 | 0,27 |
| hsa-miR-151-3p_PM | 178,76 | 149,18 | 1,20 | 0,18 | 0,08 | 0,29 | 0,01 | 0,046 | 0,62 |
| hsa-miR-382_PM | 96,42 | 75,39 | 1,28 | 0,25 | 0,03 | 0,17 | 0,01 | 0,046 | 0,64 |
