# Supplementary material for: Diagnosis of Pancreatic Ductal Adenocarcinoma and Chronic Pancreatitis by Measurement of microRNA Abundance in Blood and Tissue
Source: PLoS One. 2012 Apr 12;7(4):e34151. doi: 10.1371/journal.pone.0034151 (PMC3325244; doi:10.1371/journal.pone.0034151)
Supplement: Table S3 — Results of the RT-PCR analysis. (DOC) [file pone.0034151.s004.doc]

**Diagnosis of pancreatic ductal adenocarcinoma and chronic pancreatitis by measurement of**

**microRNA abundance in blood and tissue**

**Supplemental Table S3: Results of the RT-PCR analysis**

All analyses were done in triplicate. The respective mean and error are listed. An overall comparison to the microarray data is shown below.

| **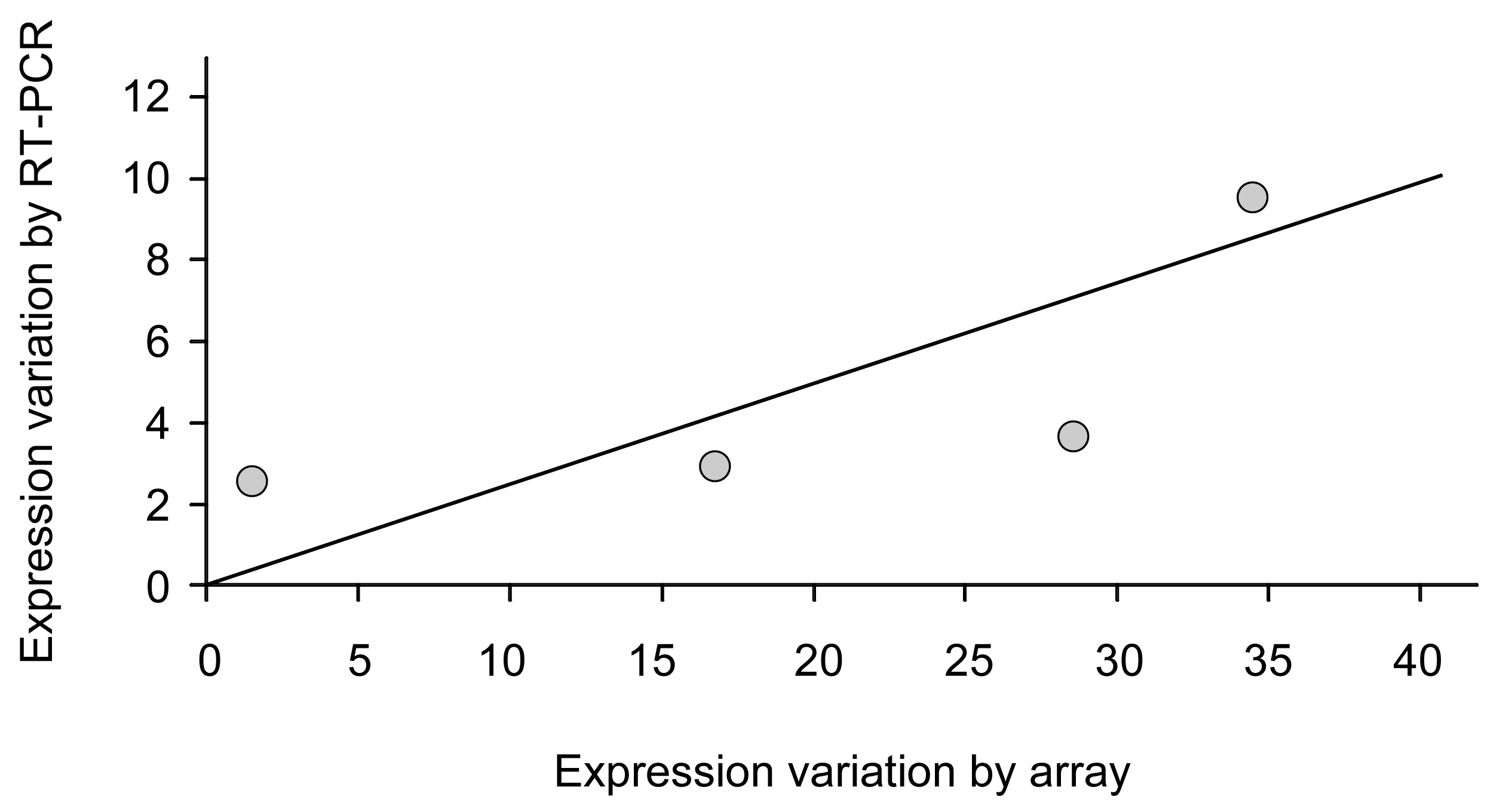** |  | 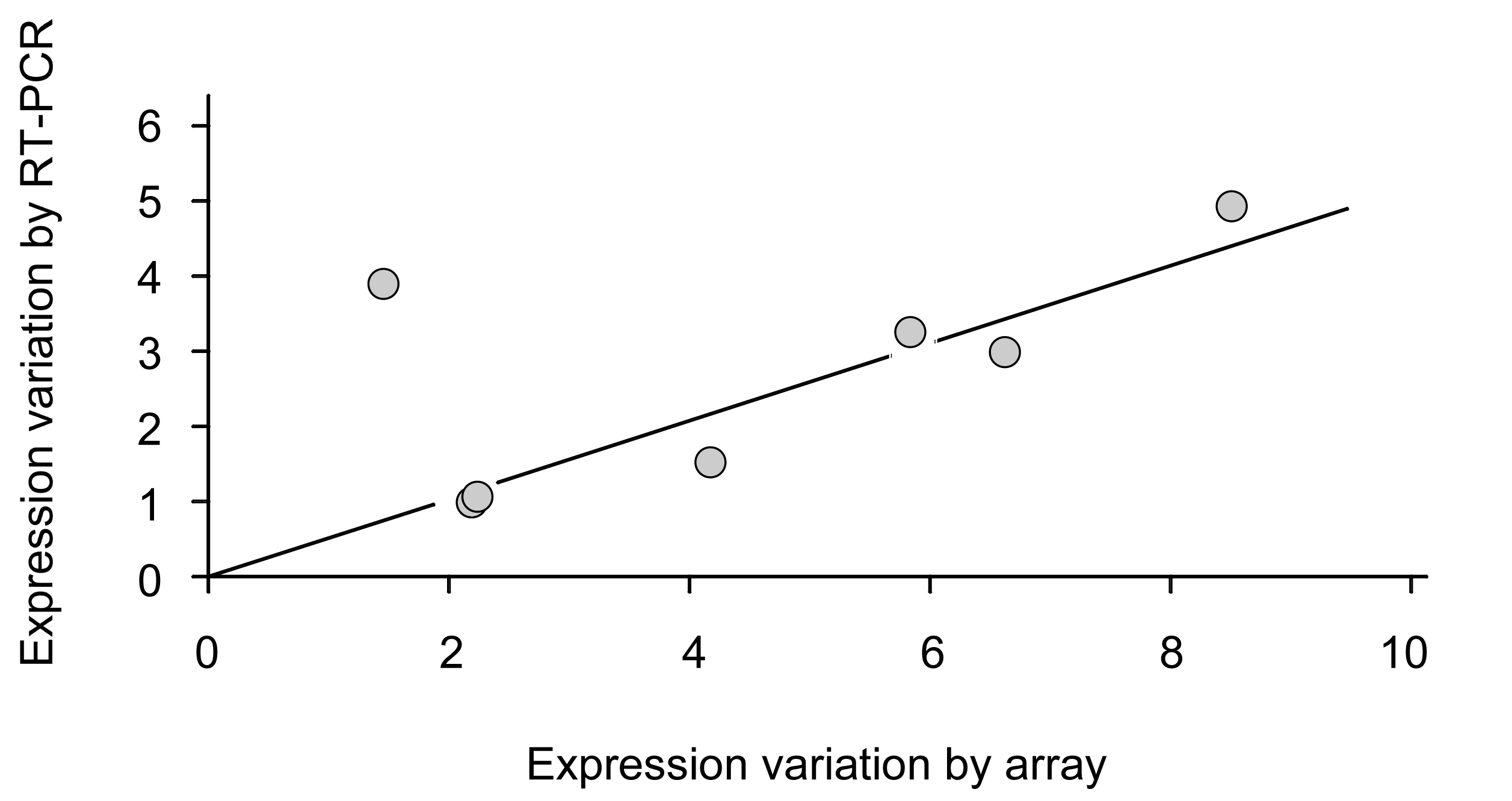 |
| --- | --- | --- |
| Correlation of RT-PCR and microarray data. The values of miRNA expression variation in blood samples are shown. The result of a miRNA that did not exhibit significant differences between cancer patients and healthy donors is not shown. Each data point represents the mean calculated from 30 sample analyses, each done in triplicate. |  | Correlation of RT-PCR and microarray data. The values of miRNA expression variation in tissue samples are shown. The result of a miRNA that did not exhibit significant differences between cancer patients and healthy donors is not shown. Each data point represents the mean calculated from 31 sample analyses, each done in triplicate. |

**Results of RT-PCR on tissue samples**

| Experiment: 2009-08-17_2_run bauer Active filter: SYBR Green I / HRM Dye (483-533) | | | | | | |  |  |
| --- | --- | --- | --- | --- | --- | --- | --- | --- |
| Chart | Pairing | Sample Name | Targets | References | Mean Cp | CP Error | Mean Cp | Cp Error |
| True |  | Kontrolle | miR-145 | RNU6B | 22,31576208 | 0,524628478 | 18,05133337 | 0,109835873 |
| True | Ge014 | Ge014 | miR-145 | RNU6B | 18,76481863 | 0,086614924 | 15,50742163 | 0,318017618 |
| True | Ge017 | Ge017 | miR-145 | RNU6B | 20,02493659 | 0,098195975 | 17,5562034 | 0,266013049 |
| True | Ge018 | Ge018 | miR-145 | RNU6B | 19,52837567 | 0,204240154 | 16,84584188 | 0,232862241 |
| True | Ge036 | Ge036 | miR-145 | RNU6B | 21,86023088 | 0,28033827 | 17,71130268 | 0,063094222 |
| True | Ge055 | Ge055 | miR-145 | RNU6B | 23,2460941 | 0,219571761 | 18,08315479 | 0,042197509 |
| True | Ge056 | Ge056 | miR-145 | RNU6B | 23,29922174 | 0,048421227 | 19,45246798 | 0,285088526 |
| True | Ge078 | Ge078 | miR-145 | RNU6B | 21,61343358 | 0,089362976 | 15,29984323 | 0,147962446 |
| True | Ge079 | Ge079 | miR-145 | RNU6B | 22,74132504 | 0,078048976 | 17,6960476 | 0,335869437 |
| True | Ge080 | Ge080 | miR-145 | RNU6B | 21,04546807 | 0,123431266 | 18,03344538 | 0,027601474 |
| True | Ge083c | Ge083c | miR-145 | RNU6B | 20,88529113 | 0,052268725 | 16,63852505 | 0,148293582 |

| Experiment: 090817_Auswertung Active filter: SYBR Green I / HRM Dye (483-533) | | | | | |  |  |  |
| --- | --- | --- | --- | --- | --- | --- | --- | --- |
| Chart | Pairing | Sample Name | Targets | References | Mean Cp | CP Error | Mean Cp | Cp Error |
| True |  | Kontrolle | miR-145 | RNU 6 B | 22,31576208 | 0,524628478 | 18,80409372 | 0,136433792 |
| True | Ge087c | Ge087c | miR-145 | RNU 6 B | 20,00789219 | 0,165794614 | 16,59261607 | 0,101502908 |
| True | Ge153 | Ge153 | miR-145 | RNU 6 B | 18,59438493 | 0,528639261 | 18,08354809 | 0,15428689 |
| True | Ge107 | Ge107 | miR-145 | RNU 6 B | 19,63464909 | 0,040356567 | 16,86851106 | 0,145934495 |
| True | Ge109 | Ge109 | miR-145 | RNU 6 B | 21,25567902 | 0,181593652 | 16,98927533 | 0,28259798 |
| True | Ge111 | Ge111 | miR-145 | RNU 6 B | 19,63277038 | 0,049487907 | 16,89502032 | 0,028785599 |
| True | Ge128 | Ge128 | miR-145 | RNU 6 B | 20,6561923 | 0,118058248 | 17,03335341 | 0,052668507 |
| True | Ge143 | Ge143 | miR-145 | RNU 6 B | 20,49020111 | 1,082163133 | 17,66076626 | 0,04138156 |
| True | Ge151 | Ge151 | miR-145 | RNU 6 B | 19,06829433 | 0,131598342 | 17,04930201 | 0,039866606 |
| True | Ge157 | Ge157 | miR-145 | RNU 6 B | 20,56991882 | 0,025568188 | 18,62911209 | 0,121656022 |
| True | Ge159 | Ge159 | miR-145 | RNU 6 B | 20,65228327 | 0,086132675 | 18,20017584 | 0,05325767 |

| Experiment: 2009-08-17_2_run bauer Active filter: SYBR Green I / HRM Dye (483-533) | | | | | | |  |  |
| --- | --- | --- | --- | --- | --- | --- | --- | --- |
| Chart | Pairing | Sample Name | Targets | References | Mean Cp | CP Error | Mean Cp | Cp Error |
| True |  | Kontrolle | miR-145 | RNU 6 B | 22,31576208 | 0,524628478 | 18,16149347 | 0,356508585 |
| True | Ge162 | Ge0162 | miR-145 | RNU 6 B | 20,4201399 | 0,198624874 | 17,89784614 | 0,106714024 |
| True | Ge165 | Ge0165 | miR-145 | RNU 6 B | 19,58172367 | 0,075575418 | 17,4714012 | 0,153195217 |
| True | Ge184 | Ge0184 | miR-145 | RNU 6 B | 19,87762564 | 0,146179111 | 18,58360552 | 0,126190313 |
| True | Ge185 | Ge0185 | miR-145 | RNU 6 B | 19,55835611 | 0,067902927 | 17,87817919 | 0,323009224 |
| True | Ge189 | Ge0189 | miR-145 | RNU 6 B | 21,71240235 | 0,052236248 | 19,6697471 | 0,09064289 |
| True | Ge194 | Ge0194 | miR-145 | RNU 6 B | 21,40235497 | 0,064215288 | 17,52033046 | 0,33498365 |
| True | Ge217 | Ge0217 | miR-145 | RNU 6 B | 20,34168596 | 0,024445091 | 16,56048257 | 0,424971642 |
| True | Ge234 | Ge0234 | miR-145 | RNU 6 B | 20,29938766 | 0,046649752 | 18,7193683 | 0,154563146 |
| True | Ge238 | Ge0238 | miR-145 | RNU 6 B | 23,46492708 | 0,13720164 | 17,37674864 | 0,114899256 |
| True | Ge256 | Ge0256 | miR-145 | RNU 6 B | 21,92938951 | 0,01845114 | 18,55969438 | 0,360963284 |

| Experiment: 2009-08-17_2_run bauer Active filter: SYBR Green I / HRM Dye (483-533) | | | | | | |  |  |
| --- | --- | --- | --- | --- | --- | --- | --- | --- |
| Chart | Pairing | Sample Name | Targets | References | Mean Cp | CP Error | Mean Cp | Cp Error |
| True |  | Kontrolle | miR-145 | RNU 6 B | 22,31576208 | 0,524628478 | 18,38576467 | 0,051178979 |
| True | Ge257 | Ge0257 | miR-145 | RNU 6 B | 21,45625026 | 0,129600021 | 19,57170959 | 0,124323842 |
| True | Ge259 | Ge0259 | miR-145 | RNU 6 B | 21,50446393 | 0,061268144 | 19,29752585 | 0,232975695 |
| True | Ge262 | Ge0262 | miR-145 | RNU 6 B | 22,06629828 | 0,035518807 | 19,55758626 | 0,018544486 |
| True | Ge264 | Ge0264 | miR-145 | RNU 6 B | 21,95188471 | 0,070794172 | 18,74416721 | 0,111272268 |
| True | Ge288 | Ge0288 | miR-145 | RNU 6 B | 19,74721168 | 0,032391216 | 17,59809473 | 0,052996629 |
| True | Ge301 | Ge0301 | miR-145 | RNU 6 B | 17,9348154 | 0,204662934 | 17,34236398 | 0,021564228 |
| True | Ge325 | Ge0325 | miR-145 | RNU 6 B | 22,70504396 | 0,078717112 | 18,10204836 | 0,173935098 |
| True | Ge326 | Ge0326 | miR-145 | RNU 6 B | 21,96104421 | 0,029688221 | 17,97363246 | 0,190682387 |
| True | Ge423 | Ge0423 | miR-145 | RNU 6 B | 22,3758374 | 0,168164538 | 18,2152126 | 0,040401979 |
| True | Ge424 | Ge0424 | miR-145 | RNU 6 B | 22,18769646 | 0,156553429 | 17,04059801 | 0,265255193 |

| Experiment: 2009-08-18_run bauer Active filter: SYBR Green I / HRM Dye (483-533) | | | | | | |  |  |
| --- | --- | --- | --- | --- | --- | --- | --- | --- |
| Chart | Pairing | Sample Name | Targets | References | Mean Cp | CP Error | Mean Cp | Cp Error |
| True |  | Kontrolle | miR-150 | RNU6B | 23,88315034 | 0,246293507 | 18,05133337 | 0,109835873 |
| True | Ge014 | Ge014 | miR-150 | RNU6B | 22,8324584 | 0,064096542 | 15,50742163 | 0,318017618 |
| True | Ge017 | Ge017 | miR-150 | RNU6B | 19,81483812 | 0,016175902 | 17,5562034 | 0,266013049 |
| True | Ge018 | Ge018 | miR-150 | RNU6B | 21,52402879 | 0,032475637 | 16,84584188 | 0,232862241 |
| True | Ge036 | Ge036 | miR-150 | RNU6B | 23,29328131 | 0,082607857 | 17,71130268 | 0,063094222 |
| True | Ge055 | Ge055 | miR-150 | RNU6B | 25,38233065 | 0,069184409 | 18,08315479 | 0,042197509 |
| True | Ge056 | Ge056 | miR-150 | RNU6B | 25,87004609 | 0,068746912 | 19,45246798 | 0,285088526 |
| True | Ge078 | Ge078 | miR-150 | RNU6B | 21,12076792 | 0,063349731 | 15,29984323 | 0,147962446 |
| True | Ge079 | Ge079 | miR-150 | RNU6B | 25,69568439 | 0,04278197 | 17,6960476 | 0,335869437 |
| True | Ge080 | Ge080 | miR-150 | RNU6B | 23,07184981 | 0,162550239 | 18,03344538 | 0,027601474 |
| True | Ge083c | Ge083c | miR-150 | RNU6B | 21,41829946 | 0,152481894 | 16,63852505 | 0,148293582 |

| Experiment: 090818_Auswertung Active filter: SYBR Green I / HRM Dye (483-533) | | | | | |  |  |  |
| --- | --- | --- | --- | --- | --- | --- | --- | --- |
| Chart | Pairing | Sample Name | Targets | References | Mean Cp | CP Error | Mean Cp | Cp Error |
| True |  | Kontrolle | miR-150 | RNU 6 B | 23,88315034 | 0,246293507 | 18,43898288 | 0,078613228 |
| True | Ge087c | Ge087c | miR-150 | RNU 6 B | 22,51391209 | 0,28018643 | 16,19467461 | 0,169021206 |
| True | Ge153 | Ge153 | miR-150 | RNU 6 B | 17,70696159 | 0,21005217 | 17,87100505 | 0,076418966 |
| True | Ge107 | Ge107 | miR-150 | RNU 6 B | 21,84930169 | 0,082161164 | 16,41808033 | 0,29593839 |
| True | Ge109 | Ge109 | miR-150 | RNU 6 B | 24,19001406 | 0,09135523 | 16,81344821 | 0,084637205 |
| True | Ge111 | Ge111 | miR-150 | RNU 6 B | 19,8333198 | 0,080278834 | 16,78937175 | 0,076122682 |
| True | Ge128 | Ge128 | miR-150 | RNU 6 B | 20,56650111 | 0,06772501 | 16,74224448 | 0,254968684 |
| True | Ge143 | Ge143 | miR-150 | RNU 6 B | 20,53032803 | 0,300586421 | 17,37952549 | 0,079776716 |
| True | Ge151 | Ge151 | miR-150 | RNU 6 B | 18,97606645 | 0,197464658 | 16,90848151 | 0,037374411 |
| True | Ge157 | Ge157 | miR-150 | RNU 6 B | 19,20677925 | 0,174535263 | 18,10012552 | 0,064252615 |
| True | Ge159 | Ge159 | miR-150 | RNU 6 B | 22,62278054 | 0,298545235 | 17,68498633 | 0,048671773 |

| Experiment: 090818_Auswertung Active filter: SYBR Green I / HRM Dye (483-533) | | | | | |  |  |  |
| --- | --- | --- | --- | --- | --- | --- | --- | --- |
| Chart | Pairing | Sample Name | Targets | References | Mean Cp | CP Error | Mean Cp | Cp Error |
| True |  | Kontrolle | miR-150 | RNU 6 B | 23,88315034 | 0,246293507 | 18,16149347 | 0,356508585 |
| True | Ge162 | Ge0162 | miR-150 | RNU 6 B | 20,91043634 | 0,122506646 | 17,89784614 | 0,106714024 |
| True | Ge165 | Ge0165 | miR-150 | RNU 6 B | 20,35036026 | 0,085303693 | 17,4714012 | 0,153195217 |
| True | Ge184 | Ge0184 | miR-150 | RNU 6 B | 22,8779126 | 0,063936036 | 18,58360552 | 0,126190313 |
| True | Ge185 | Ge0185 | miR-150 | RNU 6 B | 23,13643742 | 0,044690533 | 17,87817919 | 0,323009224 |
| True | Ge189 | Ge0189 | miR-150 | RNU 6 B | 23,59140171 | 0,04846817 | 19,6697471 | 0,09064289 |
| True | Ge194 | Ge0194 | miR-150 | RNU 6 B | 23,26334193 | 0,032311935 | 17,52033046 | 0,33498365 |
| True | Ge217 | Ge0217 | miR-150 | RNU 6 B | 22,58395424 | 0,058427279 | 16,56048257 | 0,424971642 |
| True | Ge234 | Ge0234 | miR-150 | RNU 6 B | 21,19591225 | 0,01264425 | 18,7193683 | 0,154563146 |
| True | Ge238 | Ge0238 | miR-150 | RNU 6 B | 23,33089852 | 0,012265321 | 17,37674864 | 0,114899256 |
| True | Ge256 | Ge0256 | miR-150 | RNU 6 B | 25,61551275 | 0,074041469 | 18,55969438 | 0,360963284 |

| Experiment: 090818_Auswertung Active filter: SYBR Green I / HRM Dye (483-533) | | | | | |  |  |  |
| --- | --- | --- | --- | --- | --- | --- | --- | --- |
| Chart | Pairing | Sample Name | Targets | References | Mean Cp | CP Error | Mean Cp | Cp Error |
| True |  | Kontrolle | miR-150 | RNU 6 B | 23,88315034 | 0,246293507 | 18,38576467 | 0,051178979 |
| True | Ge257 | Ge0257 | miR-150 | RNU 6 B | 23,88553371 | 0,117874875 | 19,57170959 | 0,124323842 |
| True | Ge259 | Ge0259 | miR-150 | RNU 6 B | 23,44343174 | 0,14986102 | 19,29752585 | 0,232975695 |
| True | Ge262 | Ge0262 | miR-150 | RNU 6 B | 23,94811775 | 0,062290998 | 19,55758626 | 0,018544486 |
| True | Ge264 | Ge0264 | miR-150 | RNU 6 B | 23,67625725 | 0,211943335 | 18,74416721 | 0,111272268 |
| True | Ge288 | Ge0288 | miR-150 | RNU 6 B | 21,20673977 | 0,042579337 | 17,59809473 | 0,052996629 |
| True | Ge301 | Ge0301 | miR-150 | RNU 6 B | 19,62165068 | 0,112544214 | 17,34236398 | 0,021564228 |
| True | Ge325 | Ge0325 | miR-150 | RNU 6 B | 24,0705593 | 0,111629808 | 18,10204836 | 0,173935098 |
| True | Ge326 | Ge0326 | miR-150 | RNU 6 B | 25,4074753 | 0,192801519 | 17,97363246 | 0,190682387 |
| True | Ge423 | Ge0423 | miR-150 | RNU 6 B | 24,66916385 | 0,153504245 | 18,2152126 | 0,040401979 |
| True | Ge424 | Ge0424 | miR-150 | RNU 6 B | 23,62357952 | 0,105428025 | 17,04059801 | 0,265255193 |

| Experiment: 090617_Auswertung Active filter: SYBR Green I / HRM Dye (483-533) | | | | | |  |  |  |
| --- | --- | --- | --- | --- | --- | --- | --- | --- |
| Chart | Pairing | Sample Name | Targets | References | Mean Cp | CP Error | Mean Cp | Cp Error |
| True |  | Kontrolle | mi R-31 | RNU6B | 25,94687722 | 1,455047441 | 18,05133337 | 0,109835873 |
| True | Ge014 | Ge014 | mi R-31 | RNU6B | 25,62675748 | 0,32857173 | 15,50742163 | 0,318017618 |
| True | Ge017 | Ge017 | mi R-31 | RNU6B | 24,49232296 | 0,395312854 | 17,5562034 | 0,266013049 |
| True | Ge018 | Ge018 | mi R-31 | RNU6B | 24,06741724 | 0,085797156 | 16,84584188 | 0,232862241 |
| True | Ge036 | Ge036 | mi R-31 | RNU6B | 26,99269124 | 1,735404001 | 17,71130268 | 0,063094222 |
| True | Ge055 | Ge055 | mi R-31 | RNU6B | 26,84261012 | 1,35070757 | 18,08315479 | 0,042197509 |
| True | Ge056 | Ge056 | mi R-31 | RNU6B | 25,96319247 | 2,119842773 | 19,45246798 | 0,285088526 |
| True | Ge078 | Ge078 | mi R-31 | RNU6B | 25,44009524 | 0,781688227 | 15,29984323 | 0,147962446 |
| True | Ge079 | Ge079 | mi R-31 | RNU6B | 26,79518416 | 3,508779989 | 17,6960476 | 0,335869437 |
| True | Ge080 | Ge080 | mi R-31 | RNU6B | 26,01527902 | 2,491419856 | 18,03344538 | 0,027601474 |
| True | Ge083c | Ge083c | mi R-31 | RNU6B | 25,27516994 | 0,702100383 | 16,63852505 | 0,148293582 |

| Experiment: 090619_Auswertung Active filter: SYBR Green I / HRM Dye (483-533) | | | | | |  |  |  |
| --- | --- | --- | --- | --- | --- | --- | --- | --- |
| Chart | Pairing | Sample Name | Targets | References | Mean Cp | CP Error | Mean Cp | Cp Error |
| True |  | Kontrolle | mi R-31 | RNU 6 B | 28,60654732 | 0,119805056 | 18,80409372 | 0,136433792 |
| True | Ge087c | Ge087c | mi R-31 | RNU 6 B | 24,95981214 | 0,19128755 | 16,59261607 | 0,101502908 |
| True | Ge153 | Ge153 | mi R-31 | RNU 6 B | 25,08305965 | 0,054312807 | 18,08354809 | 0,15428689 |
| True | Ge107 | Ge107 | mi R-31 | RNU 6 B | 29,78683107 | 0,377465325 | 16,86851106 | 0,145934495 |
| True | Ge109 | Ge109 | mi R-31 | RNU 6 B | 27,58845577 | 0,088430595 | 16,98927533 | 0,28259798 |
| True | Ge111 | Ge111 | mi R-31 | RNU 6 B | 25,4214133 | 0,117145723 | 16,89502032 | 0,028785599 |
| True | Ge128 | Ge128 | mi R-31 | RNU 6 B | 26,67863276 | 0,314213097 | 17,03335341 | 0,052668507 |
| True | Ge143 | Ge143 | mi R-31 | RNU 6 B | 26,15652343 | 0,798484641 | 17,66076626 | 0,04138156 |
| True | Ge151 | Ge151 | mi R-31 | RNU 6 B | 28,46847502 | 0,138471931 | 17,04930201 | 0,039866606 |
| True | Ge157 | Ge157 | mi R-31 | RNU 6 B | 26,0938058 | 0,090530408 | 18,62911209 | 0,121656022 |
| True | Ge159 | Ge159 | mi R-31 | RNU 6 B | 23,59461993 | 0,057988495 | 18,20017584 | 0,05325767 |

| Experiment: 090626_Auswertung Active filter: SYBR Green I / HRM Dye (483-533) | | | | | |  |  |  |
| --- | --- | --- | --- | --- | --- | --- | --- | --- |
| Chart | Pairing | Sample Name | Targets | References | Mean Cp | CP Error | Mean Cp | Cp Error |
| True |  | Kontrolle | HS_miR_103_1 | RNU6B | 24,77134535 | 0,249929947 | 18,05133337 | 0,109835873 |
| True | Ge014 | Ge014 | HS_miR_103_1 | RNU6B | 21,81599684 | 0,208140557 | 15,50742163 | 0,318017618 |
| True | Ge017 | Ge017 | HS_miR_103_1 | RNU6B | 22,75001091 | 0,02462979 | 17,5562034 | 0,266013049 |
| True | Ge018 | Ge018 | HS_miR_103_1 | RNU6B | 22,00227458 | 0,04923879 | 16,84584188 | 0,232862241 |
| True | Ge036 | Ge036 | HS_miR_103_1 | RNU6B | 24,50720831 | 0,050538792 | 17,71130268 | 0,063094222 |
| True | Ge055 | Ge055 | HS_miR_103_1 | RNU6B | 25,79527011 | 0,055678294 | 18,08315479 | 0,042197509 |
| True | Ge056 | Ge056 | HS_miR_103_1 | RNU6B | 26,58685111 | 0,048902283 | 19,45246798 | 0,285088526 |
| True | Ge078 | Ge078 | HS_miR_103_1 | RNU6B | 23,2890106 | 0,072118327 | 15,29984323 | 0,147962446 |
| True | Ge079 | Ge079 | HS_miR_103_1 | RNU6B | 26,12034631 | 0,049906797 | 17,6960476 | 0,335869437 |
| True | Ge080 | Ge080 | HS_miR_103_1 | RNU6B | 24,90217005 | 0,258047769 | 18,03344538 | 0,027601474 |
| True | Ge083c | Ge083c | HS_miR_103_1 | RNU6B | 23,69664139 | 0,11662921 | 16,63852505 | 0,148293582 |

| Experiment: 090622_Auswertung Active filter: SYBR Green I / HRM Dye (483-533) | | | | | |  |  |  |
| --- | --- | --- | --- | --- | --- | --- | --- | --- |
| Chart | Pairing | Sample Name | Targets | References | Mean Cp | CP Error | Mean Cp | Cp Error |
| True |  | Kontrolle | HS_miR_103_1 | RNU 6 B | 23,77845864 | 1,47834395 | 18,43898288 | 0,078613228 |
| True | Ge087c | Ge087c | HS_miR_103_1 | RNU 6 B | 23,93190857 | 0,1041138 | 16,19467461 | 0,169021206 |
| True | Ge153 | Ge153 | HS_miR_103_1 | RNU 6 B | 22,41231381 | 0,073067683 | 17,87100505 | 0,076418966 |
| True | Ge107 | Ge107 | HS_miR_103_1 | RNU 6 B | 21,39539606 | 0,128026725 | 16,41808033 | 0,29593839 |
| True | Ge109 | Ge109 | HS_miR_103_1 | RNU 6 B | 23,89380272 | 0,019148984 | 16,81344821 | 0,084637205 |
| True | Ge111 | Ge111 | HS_miR_103_1 | RNU 6 B | 22,96600199 | 0,026698044 | 16,78937175 | 0,076122682 |
| True | Ge128 | Ge128 | HS_miR_103_1 | RNU 6 B | 24,30715757 | 0,116125082 | 16,74224448 | 0,254968684 |
| True | Ge143 | Ge143 | HS_miR_103_1 | RNU 6 B | 23,52507839 | 0,067820095 | 17,37952549 | 0,079776716 |
| True | Ge151 | Ge151 | HS_miR_103_1 | RNU 6 B | 23,39428235 | 0,074453737 | 16,90848151 | 0,037374411 |
| True | Ge157 | Ge157 | HS_miR_103_1 | RNU 6 B | 23,86808351 | 0,100192424 | 18,10012552 | 0,064252615 |
| True | Ge159 | Ge159 | HS_miR_103_1 | RNU 6 B | 22,82944876 | 0,059624984 | 17,68498633 | 0,048671773 |

| Experiment: 090715_Auswertung Active filter: SYBR Green I / HRM Dye (483-533) | | | | | |  |  |  |
| --- | --- | --- | --- | --- | --- | --- | --- | --- |
| Chart | Pairing | Sample Name | Targets | References | Mean Cp | CP Error | Mean Cp | Cp Error |
| True |  | Kontrolle | HS_miR_103_1 | RNU 6 B | 25,04640497 | 0,069906557 | 18,16149347 | 0,356508585 |
| True | Ge162 | Ge0162 | HS_miR_103_1 | RNU 6 B | 23,82982399 | 0,17399609 | 17,89784614 | 0,106714024 |
| True | Ge165 | Ge0165 | HS_miR_103_1 | RNU 6 B | 23,83258215 | 0,096353832 | 17,4714012 | 0,153195217 |
| True | Ge184 | Ge0184 | HS_miR_103_1 | RNU 6 B | 24,99677293 | 0,164849875 | 18,58360552 | 0,126190313 |
| True | Ge185 | Ge0185 | HS_miR_103_1 | RNU 6 B | 23,76786342 | 0,063216022 | 17,87817919 | 0,323009224 |
| True | Ge189 | Ge0189 | HS_miR_103_1 | RNU 6 B | 24,38499535 | 0,241536683 | 19,6697471 | 0,09064289 |
| True | Ge194 | Ge0194 | HS_miR_103_1 | RNU 6 B | 21,84716673 | 0,19628249 | 17,52033046 | 0,33498365 |
| True | Ge217 | Ge0217 | HS_miR_103_1 | RNU 6 B | 22,79119522 | 0,242613056 | 16,56048257 | 0,424971642 |
| True | Ge234 | Ge0234 | HS_miR_103_1 | RNU 6 B | 23,49546164 | 0,37662188 | 18,7193683 | 0,154563146 |
| True | Ge238 | Ge0238 | HS_miR_103_1 | RNU 6 B | 24,65244135 | 0,545604308 | 17,37674864 | 0,114899256 |
| True | Ge256 | Ge0256 | HS_miR_103_1 | RNU 6 B | 24,46688288 | 0,143349699 | 18,55969438 | 0,360963284 |

| Experiment: 090701_Auswertung Active filter: SYBR Green I / HRM Dye (483-533) | | | | | |  |  |  |
| --- | --- | --- | --- | --- | --- | --- | --- | --- |
| Chart | Pairing | Sample Name | Targets | References | Mean Cp | CP Error | Mean Cp | Cp Error |
| True |  | Kontrolle | HS_miR_103_1 | RNU 6 B | 24,48954489 | 0,141068049 | 18,38576467 | 0,051178979 |
| True | Ge0257 | Ge0257 | HS_miR_103_1 | RNU 6 B | 25,00889393 | 0,129266958 | 19,57170959 | 0,124323842 |
| True | Ge0259 | Ge0259 | HS_miR_103_1 | RNU 6 B | 24,444015 | 0,165767391 | 19,29752585 | 0,232975695 |
| True | Ge0262 | Ge0262 | HS_miR_103_1 | RNU 6 B | 24,03226544 | 0,117622527 | 19,55758626 | 0,018544486 |
| True | Ge0264 | Ge0264 | HS_miR_103_1 | RNU 6 B | 23,31255421 | 0,050784909 | 18,74416721 | 0,111272268 |
| True | Ge0288 | Ge0288 | HS_miR_103_1 | RNU 6 B | 21,74304992 | 0,039446329 | 17,59809473 | 0,052996629 |
| True | Ge0301 | Ge0301 | HS_miR_103_1 | RNU 6 B | 20,93989923 | 0,062865229 | 17,34236398 | 0,021564228 |
| True | Ge0325 | Ge0325 | HS_miR_103_1 | RNU 6 B | 26,15945379 | 0,045501539 | 18,10204836 | 0,173935098 |
| True | Ge0326 | Ge0326 | HS_miR_103_1 | RNU 6 B | 25,5749412 | 0,055260448 | 17,97363246 | 0,190682387 |
| True | Ge0423 | Ge0423 | HS_miR_103_1 | RNU 6 B | 24,32692128 | 0,097142154 | 18,2152126 | 0,040401979 |
| True | Ge0424 | Ge0424 | HS_miR_103_1 | RNU 6 B | 24,92972539 | 0,138513772 | 17,04059801 | 0,265255193 |

| Experiment: 090626_Auswertung Active filter: SYBR Green I / HRM Dye (483-533) | | | | | |  |  |  |
| --- | --- | --- | --- | --- | --- | --- | --- | --- |
| Chart | Pairing | Sample Name | Targets | References | Mean Cp | CP Error | Mean Cp | Cp Error |
| True |  | Kontrolle | HS_miR_107_1 | RNU6B | 25,12756816 | 0,378488434 | 18,05133337 | 0,109835873 |
| True | Ge014 | Ge014 | HS_miR_107_1 | RNU6B | 21,77873632 | 0,141231427 | 15,50742163 | 0,318017618 |
| True | Ge017 | Ge017 | HS_miR_107_1 | RNU6B | 22,78376048 | 0,081587468 | 17,5562034 | 0,266013049 |
| True | Ge018 | Ge018 | HS_miR_107_1 | RNU6B | 21,90311193 | 0,065619591 | 16,84584188 | 0,232862241 |
| True | Ge036 | Ge036 | HS_miR_107_1 | RNU6B | 24,45304705 | 0,007727745 | 17,71130268 | 0,063094222 |
| True | Ge055 | Ge055 | HS_miR_107_1 | RNU6B | 25,77132489 | 0,049133467 | 18,08315479 | 0,042197509 |
| True | Ge056 | Ge056 | HS_miR_107_1 | RNU6B | 26,59515997 | 0,031923842 | 19,45246798 | 0,285088526 |
| True | Ge078 | Ge078 | HS_miR_107_1 | RNU6B | 23,27306704 | 0,016968227 | 15,29984323 | 0,147962446 |
| True | Ge079 | Ge079 | HS_miR_107_1 | RNU6B | 26,10559726 | 0,02504649 | 17,6960476 | 0,335869437 |
| True | Ge080 | Ge080 | HS_miR_107_1 | RNU6B | 25,09291509 | 0,074343522 | 18,03344538 | 0,027601474 |
| True | Ge083c | Ge083c | HS_miR_107_1 | RNU6B | 23,63049338 | 0,054196927 | 16,63852505 | 0,148293582 |

| Experiment: 090622_Auswertung Active filter: SYBR Green I / HRM Dye (483-533) | | | | | |  |  |  |
| --- | --- | --- | --- | --- | --- | --- | --- | --- |
| Chart | Pairing | Sample Name | Targets | References | Mean Cp | CP Error | Mean Cp | Cp Error |
| True |  | Kontrolle | HS_miR_107_1 | RNU 6 B | 24,62840226 | 0,06820008 | 18,43898288 | 0,078613228 |
| True | Ge087c | Ge087c | HS_miR_107_1 | RNU 6 B | 23,94268533 | 0,106892586 | 16,19467461 | 0,169021206 |
| True | Ge153 | Ge153 | HS_miR_107_1 | RNU 6 B | 22,29927384 | 0,167302219 | 17,87100505 | 0,076418966 |
| True | Ge107 | Ge107 | HS_miR_107_1 | RNU 6 B | 21,41503279 | 0,134244773 | 16,41808033 | 0,29593839 |
| True | Ge109 | Ge109 | HS_miR_107_1 | RNU 6 B | 23,77590927 | 0,035286201 | 16,81344821 | 0,084637205 |
| True | Ge111 | Ge111 | HS_miR_107_1 | RNU 6 B | 23,06211233 | 0,00551391 | 16,78937175 | 0,076122682 |
| True | Ge128 | Ge128 | HS_miR_107_1 | RNU 6 B | 24,21779791 | 0,096774962 | 16,74224448 | 0,254968684 |
| True | Ge143 | Ge143 | HS_miR_107_1 | RNU 6 B | 23,63675883 | 0,082481196 | 17,37952549 | 0,079776716 |
| True | Ge151 | Ge151 | HS_miR_107_1 | RNU 6 B | 23,53373904 | 0,078108705 | 16,90848151 | 0,037374411 |
| True | Ge157 | Ge157 | HS_miR_107_1 | RNU 6 B | 23,93622403 | 0,069921318 | 18,10012552 | 0,064252615 |
| True | Ge159 | Ge159 | HS_miR_107_1 | RNU 6 B | 22,84625698 | 0,024755509 | 17,68498633 | 0,048671773 |

| Experiment: 090715_Auswertung Active filter: SYBR Green I / HRM Dye (483-533) | | | | | |  |  |  |
| --- | --- | --- | --- | --- | --- | --- | --- | --- |
| Chart | Pairing | Sample Name | Targets | References | Mean Cp | CP Error | Mean Cp | Cp Error |
| True |  | Kontrolle | HS_miR_107_1 | RNU 6 B | 24,79344246 | 0,274897466 | 18,16149347 | 0,356508585 |
| True | Ge162 | Ge0162 | HS_miR_107_1 | RNU 6 B | 24,03925097 | 0,267751342 | 17,89784614 | 0,106714024 |
| True | Ge165 | Ge0165 | HS_miR_107_1 | RNU 6 B | 23,84110219 | 0,083261644 | 17,4714012 | 0,153195217 |
| True | Ge184 | Ge0184 | HS_miR_107_1 | RNU 6 B | 25,01858203 | 0,260498924 | 18,58360552 | 0,126190313 |
| True | Ge185 | Ge0185 | HS_miR_107_1 | RNU 6 B | 23,80640805 | 0,206205838 | 17,87817919 | 0,323009224 |
| True | Ge189 | Ge0189 | HS_miR_107_1 | RNU 6 B | 24,5085615 | 0,181852536 | 19,6697471 | 0,09064289 |
| True | Ge194 | Ge0194 | HS_miR_107_1 | RNU 6 B | 22,00139737 | 0,084184497 | 17,52033046 | 0,33498365 |
| True | Ge217 | Ge0217 | HS_miR_107_1 | RNU 6 B | 22,81111023 | 0,063449592 | 16,56048257 | 0,424971642 |
| True | Ge234 | Ge0234 | HS_miR_107_1 | RNU 6 B | 23,72488424 | 0,186776935 | 18,7193683 | 0,154563146 |
| True | Ge238 | Ge0238 | HS_miR_107_1 | RNU 6 B | 24,57106411 | 0,505645565 | 17,37674864 | 0,114899256 |
| True | Ge256 | Ge0256 | HS_miR_107_1 | RNU 6 B | 24,22070812 | 0,052174351 | 18,55969438 | 0,360963284 |

| Experiment: 090701_Auswertung Active filter: SYBR Green I / HRM Dye (483-533) | | | | | |  |  |  |
| --- | --- | --- | --- | --- | --- | --- | --- | --- |
| Chart | Pairing | Sample Name | Targets | References | Mean Cp | CP Error | Mean Cp | Cp Error |
| True |  | Kontrolle | HS_miR_107_1 | RNU 6 B | 24,52755167 | 0,056047144 | 18,38576467 | 0,051178979 |
| True | Ge0257 | Ge0257 | HS_miR_107_1 | RNU 6 B | 24,9770848 | 0,143132283 | 19,57170959 | 0,124323842 |
| True | Ge0259 | Ge0259 | HS_miR_107_1 | RNU 6 B | 24,6271204 | 0,116915627 | 19,29752585 | 0,232975695 |
| True | Ge0262 | Ge0262 | HS_miR_107_1 | RNU 6 B | 23,90361392 | 0,026156223 | 19,55758626 | 0,018544486 |
| True | Ge0264 | Ge0264 | HS_miR_107_1 | RNU 6 B | 23,53560857 | 0,01354926 | 18,74416721 | 0,111272268 |
| True | Ge0288 | Ge0288 | HS_miR_107_1 | RNU 6 B | 21,84696581 | 0,149120054 | 17,59809473 | 0,052996629 |
| True | Ge0301 | Ge0301 | HS_miR_107_1 | RNU 6 B | 21,04262421 | 0,137953167 | 17,34236398 | 0,021564228 |
| True | Ge0325 | Ge0325 | HS_miR_107_1 | RNU 6 B | 26,17541678 | 0,092390778 | 18,10204836 | 0,173935098 |
| True | Ge0326 | Ge0326 | HS_miR_107_1 | RNU 6 B | 25,64903218 | 0,071184834 | 17,97363246 | 0,190682387 |
| True | Ge0423 | Ge0423 | HS_miR_107_1 | RNU 6 B | 24,41805561 | 0,060216682 | 18,2152126 | 0,040401979 |
| True | Ge0424 | Ge0424 | HS_miR_107_1 | RNU 6 B | 24,89778441 | 0,011357098 | 17,04059801 | 0,265255193 |

| Experiment: 090626_Auswertung Active filter: SYBR Green I / HRM Dye (483-533) | | | | | |  |  |  |
| --- | --- | --- | --- | --- | --- | --- | --- | --- |
| Chart | Pairing | Sample Name | Targets | References | Mean Cp | CP Error | Mean Cp | Cp Error |
| True |  | Kontrolle | HS_miR_155_1 | RNU6B | 31,40126171 | 0,666433752 | 18,05133337 | 0,109835873 |
| True | Ge014 | Ge014 | HS_miR_155_1 | RNU6B | 27,33997305 | 0,205754097 | 15,50742163 | 0,318017618 |
| True | Ge017 | Ge017 | HS_miR_155_1 | RNU6B | 25,69474346 | 0,082742772 | 17,5562034 | 0,266013049 |
| True | Ge018 | Ge018 | HS_miR_155_1 | RNU6B | 25,88870039 | 0,100234092 | 16,84584188 | 0,232862241 |
| True | Ge036 | Ge036 | HS_miR_155_1 | RNU6B | 28,87470966 | 0,138809176 | 17,71130268 | 0,063094222 |
| True | Ge055 | Ge055 | HS_miR_155_1 | RNU6B | 30,99988126 | 2,023314253 | 18,08315479 | 0,042197509 |
| True | Ge056 | Ge056 | HS_miR_155_1 | RNU6B | 32,36710664 | 2,525419032 | 19,45246798 | 0,285088526 |
| True | Ge078 | Ge078 | HS_miR_155_1 | RNU6B | 26,66669652 | 0,034443164 | 15,29984323 | 0,147962446 |
| True | Ge079 | Ge079 | HS_miR_155_1 | RNU6B | 32,47966649 | 0,134820118 | 17,6960476 | 0,335869437 |
| True | Ge080 | Ge080 | HS_miR_155_1 | RNU6B | 28,22174368 | 0,048001165 | 18,03344538 | 0,027601474 |
| True | Ge083c | Ge083c | HS_miR_155_1 | RNU6B | 28,01058947 | 0,019345756 | 16,63852505 | 0,148293582 |

| Experiment: 090622_Auswertung Active filter: SYBR Green I / HRM Dye (483-533) | | | | | |  |  |  |
| --- | --- | --- | --- | --- | --- | --- | --- | --- |
| Chart | Pairing | Sample Name | Targets | References | Mean Cp | CP Error | Mean Cp | Cp Error |
| True |  | Kontrolle | HS_miR_155_1 | RNU 6 B | 30,86130316 | 0,029888368 | 18,43898288 | 0,078613228 |
| True | Ge087c | Ge087c | HS_miR_155_1 | RNU 6 B | 27,41360685 | 0,132237386 | 16,19467461 | 0,169021206 |
| True | Ge153 | Ge153 | HS_miR_155_1 | RNU 6 B | 25,58482221 | 0,047482023 | 17,87100505 | 0,076418966 |
| True | Ge107 | Ge107 | HS_miR_155_1 | RNU 6 B | 27,20924744 | 0,119841564 | 16,41808033 | 0,29593839 |
| True | Ge109 | Ge109 | HS_miR_155_1 | RNU 6 B | 28,00958389 | 0,040318463 | 16,81344821 | 0,084637205 |
| True | Ge111 | Ge111 | HS_miR_155_1 | RNU 6 B | 26,38683749 | 0,095288793 | 16,78937175 | 0,076122682 |
| True | Ge128 | Ge128 | HS_miR_155_1 | RNU 6 B | 27,70091057 | 0,099601404 | 16,74224448 | 0,254968684 |
| True | Ge143 | Ge143 | HS_miR_155_1 | RNU 6 B | 26,24157745 | 0,094451894 | 17,37952549 | 0,079776716 |
| True | Ge151 | Ge151 | HS_miR_155_1 | RNU 6 B | 25,82045426 | 0,117715366 | 16,90848151 | 0,037374411 |
| True | Ge157 | Ge157 | HS_miR_155_1 | RNU 6 B | 26,2637978 | 0,089316969 | 18,10012552 | 0,064252615 |
| True | Ge159 | Ge159 | HS_miR_155_1 | RNU 6 B | 26,91550125 | 0,036087856 | 17,68498633 | 0,048671773 |

| Experiment: 090625_Auswertung Active filter: SYBR Green I / HRM Dye (483-533) | | | | | |  |  |  |
| --- | --- | --- | --- | --- | --- | --- | --- | --- |
| Chart | Pairing | Sample Name | Targets | References | Mean Cp | CP Error | Mean Cp | Cp Error |
| True |  | Kontrolle | HS_miR_155_1 | RNU 6 B | 30,98117657 | 0,047467541 | 18,16149347 | 0,356508585 |
| True | Ge0162 | Ge0162 | HS_miR_155_1 | RNU 6 B | 26,90564804 | 0,13348686 | 17,89784614 | 0,106714024 |
| True | Ge0165 | Ge0165 | HS_miR_155_1 | RNU 6 B | 26,79683102 | 0,097454649 | 17,4714012 | 0,153195217 |
| True | Ge0184 | Ge0184 | HS_miR_155_1 | RNU 6 B | 28,07818267 | 0,027035992 | 18,58360552 | 0,126190313 |
| True | Ge0185 | Ge0185 | HS_miR_155_1 | RNU 6 B | 28,14335787 | 0,333313256 | 17,87817919 | 0,323009224 |
| True | Ge0189 | Ge0189 | HS_miR_155_1 | RNU 6 B | 26,61932956 | 0,356672628 | 19,6697471 | 0,09064289 |
| True | Ge0194 | Ge0194 | HS_miR_155_1 | RNU 6 B | 28,10265072 | 0,063690111 | 17,52033046 | 0,33498365 |
| True | Ge0217 | Ge0217 | HS_miR_155_1 | RNU 6 B | 27,33633 | 0,102954933 | 16,56048257 | 0,424971642 |
| True | Ge0234 | Ge0234 | HS_miR_155_1 | RNU 6 B | 27,26445106 | 0,214084625 | 18,7193683 | 0,154563146 |
| True | Ge0238 | Ge0238 | HS_miR_155_1 | RNU 6 B | 27,56674361 | 0,047213813 | 17,37674864 | 0,114899256 |
| True | Ge0256 | Ge0256 | HS_miR_155_1 | RNU 6 B | 32,60995702 | 0,358005998 | 18,55969438 | 0,360963284 |

| Experiment: 090701_Auswertung Active filter: SYBR Green I / HRM Dye (483-533) | | | | | |  |  |  |
| --- | --- | --- | --- | --- | --- | --- | --- | --- |
| Chart | Pairing | Sample Name | Targets | References | Mean Cp | CP Error | Mean Cp | Cp Error |
| True |  | Kontrolle | HS_miR_155_1 | RNU 6 B | 30,91558876 | 0,120025865 | 18,38576467 | 0,051178979 |
| True | Ge0257 | Ge0257 | HS_miR_155_1 | RNU 6 B | 30,33610728 | 0,176439528 | 19,57170959 | 0,124323842 |
| True | Ge0259 | Ge0259 | HS_miR_155_1 | RNU 6 B | 29,79524812 | 0,109229946 | 19,29752585 | 0,232975695 |
| True | Ge0262 | Ge0262 | HS_miR_155_1 | RNU 6 B | 29,84234573 | 0,064839139 | 19,55758626 | 0,018544486 |
| True | Ge0264 | Ge0264 | HS_miR_155_1 | RNU 6 B | 29,59855096 | 0,086595088 | 18,74416721 | 0,111272268 |
| True | Ge0288 | Ge0288 | HS_miR_155_1 | RNU 6 B | 26,0560584 | 0,114296294 | 17,59809473 | 0,052996629 |
| True | Ge0301 | Ge0301 | HS_miR_155_1 | RNU 6 B | 24,70374799 | 0,064044776 | 17,34236398 | 0,021564228 |
| True | Ge0325 | Ge0325 | HS_miR_155_1 | RNU 6 B | 29,13450812 | 0,107492914 | 18,10204836 | 0,173935098 |
| True | Ge0326 | Ge0326 | HS_miR_155_1 | RNU 6 B | 29,22306304 | 0,126675953 | 17,97363246 | 0,190682387 |
| True | Ge0423 | Ge0423 | HS_miR_155_1 | RNU 6 B | 31,05067247 | 0,073789478 | 18,2152126 | 0,040401979 |
| True | Ge0424 | Ge0424 | HS_miR_155_1 | RNU 6 B | 31,3021081 | 0,253854376 | 17,04059801 | 0,265255193 |

| Experiment: 090617_Auswertung Active filter: SYBR Green I / HRM Dye (483-533) | | | | | |  |  |  |
| --- | --- | --- | --- | --- | --- | --- | --- | --- |
| Chart | Pairing | Sample Name | Targets | References | Mean Cp | CP Error | Mean Cp | Cp Error |
| True |  | Kontrolle | mi R-214 | RNU6B | 22,786539 | 0,338753768 | 18,05133337 | 0,109835873 |
| True | Ge014 | Ge014 | mi R-214 | RNU6B | 18,5206923 | 0,183776693 | 15,50742163 | 0,318017618 |
| True | Ge017 | Ge017 | mi R-214 | RNU6B | 20,73349257 | 0,296418514 | 17,5562034 | 0,266013049 |
| True | Ge018 | Ge018 | mi R-214 | RNU6B | 20,02264486 | 0,441755798 | 16,84584188 | 0,232862241 |
| True | Ge036 | Ge036 | mi R-214 | RNU6B | 21,47746036 | 0,233302432 | 17,71130268 | 0,063094222 |
| True | Ge055 | Ge055 | mi R-214 | RNU6B | 23,33960472 | 0,38976927 | 18,08315479 | 0,042197509 |
| True | Ge056 | Ge056 | mi R-214 | RNU6B | 23,76858217 | 0,80862162 | 19,45246798 | 0,285088526 |
| True | Ge078 | Ge078 | mi R-214 | RNU6B | 21,75513775 | 0,027902616 | 15,29984323 | 0,147962446 |
| True | Ge079 | Ge079 | mi R-214 | RNU6B | 22,30816975 | 0,75307936 | 17,6960476 | 0,335869437 |
| True | Ge080 | Ge080 | mi R-214 | RNU6B | 21,34228954 | 0,19438228 | 18,03344538 | 0,027601474 |
| True | Ge083c | Ge083c | mi R-214 | RNU6B | 20,45904446 | 0,39636557 | 16,63852505 | 0,148293582 |

| Experiment: 090619_Auswertung Active filter: SYBR Green I / HRM Dye (483-533) | | | | | |  |  |  |
| --- | --- | --- | --- | --- | --- | --- | --- | --- |
| Chart | Pairing | Sample Name | Targets | References | Mean Cp | CP Error | Mean Cp | Cp Error |
| True |  | Kontrolle | mi R-214 | RNU 6 B | 23,40028646 | 0,083750455 | 18,80409372 | 0,136433792 |
| True | Ge087c | Ge087c | mi R-214 | RNU 6 B | 20,58560582 | 0,08885478 | 16,59261607 | 0,101502908 |
| True | Ge153 | Ge153 | mi R-214 | RNU 6 B | 18,25256569 | 0,087651985 | 18,08354809 | 0,15428689 |
| True | Ge107 | Ge107 | mi R-214 | RNU 6 B | 20,74854981 | 0,107918944 | 16,86851106 | 0,145934495 |
| True | Ge109 | Ge109 | mi R-214 | RNU 6 B | 21,70069059 | 0,028941965 | 16,98927533 | 0,28259798 |
| True | Ge111 | Ge111 | mi R-214 | RNU 6 B | 20,23875592 | 0,244048282 | 16,89502032 | 0,028785599 |
| True | Ge128 | Ge128 | mi R-214 | RNU 6 B | 20,74177249 | 0,013017472 | 17,03335341 | 0,052668507 |
| True | Ge143 | Ge143 | mi R-214 | RNU 6 B | 20,04879257 | 0,172155755 | 17,66076626 | 0,04138156 |
| True | Ge151 | Ge151 | mi R-214 | RNU 6 B | 19,62421568 | 0,020703745 | 17,04930201 | 0,039866606 |
| True | Ge157 | Ge157 | mi R-214 | RNU 6 B | 19,87134853 | 0,006507364 | 18,62911209 | 0,121656022 |
| True | Ge159 | Ge159 | mi R-214 | RNU 6 B | 21,18834324 | 0,058559578 | 18,20017584 | 0,05325767 |

| Experiment: 090625_Auswertung Active filter: SYBR Green I / HRM Dye (483-533) | | | | | |  |  |  |
| --- | --- | --- | --- | --- | --- | --- | --- | --- |
| Chart | Pairing | Sample Name | Targets | References | Mean Cp | CP Error | Mean Cp | Cp Error |
| True |  | Kontrolle | mi R-214 | RNU 6 B | 23,39977637 | 0,078584231 | 18,16149347 | 0,356508585 |
| True | Ge0162 | Ge0162 | mi R-214 | RNU 6 B | 20,35386538 | 0,175652706 | 17,89784614 | 0,106714024 |
| True | Ge0165 | Ge0165 | mi R-214 | RNU 6 B | 20,0324054 | 0,060477526 | 17,4714012 | 0,153195217 |
| True | Ge0184 | Ge0184 | mi R-214 | RNU 6 B | 19,78146881 | 0,064734296 | 18,58360552 | 0,126190313 |
| True | Ge0185 | Ge0185 | mi R-214 | RNU 6 B | 19,60650917 | 0,101983255 | 17,87817919 | 0,323009224 |
| True | Ge0189 | Ge0189 | mi R-214 | RNU 6 B | 22,68631347 | 0,038132887 | 19,6697471 | 0,09064289 |
| True | Ge0194 | Ge0194 | mi R-214 | RNU 6 B | 22,52313709 | 0,140064431 | 17,52033046 | 0,33498365 |
| True | Ge0217 | Ge0217 | mi R-214 | RNU 6 B | 21,49141786 | 0,259380382 | 16,56048257 | 0,424971642 |
| True | Ge0234 | Ge0234 | mi R-214 | RNU 6 B | 21,85528162 | 0,193044191 | 18,7193683 | 0,154563146 |
| True | Ge0238 | Ge0238 | mi R-214 | RNU 6 B | 23,18301276 | 0,024359821 | 17,37674864 | 0,114899256 |
| True | Ge0256 | Ge0256 | mi R-214 | RNU 6 B | 23,67914322 | 0,058082265 | 18,55969438 | 0,360963284 |

| Experiment: 090716_Auswertung Active filter: SYBR Green I / HRM Dye (483-533) | | | | | |  |  |  |
| --- | --- | --- | --- | --- | --- | --- | --- | --- |
| Chart | Pairing | Sample Name | Targets | References | Mean Cp | CP Error | Mean Cp | Cp Error |
| True |  | Kontrolle | mi R-214_1 | RNU 6 B | 23,29406353 | 0,174776752 | 18,38576467 | 0,051178979 |
| True | Ge0257 | Ge0257 | mi R-214_1 | RNU 6 B | 23,43436205 | 0,161250898 | 19,57170959 | 0,124323842 |
| True | Ge0259 | Ge0259 | mi R-214_1 | RNU 6 B | 23,12564946 | 0,030607101 | 19,29752585 | 0,232975695 |
| True | Ge0262 | Ge0262 | mi R-214_1 | RNU 6 B | 23,53710163 | 0,224158012 | 19,55758626 | 0,018544486 |
| True | Ge0264 | Ge0264 | mi R-214_1 | RNU 6 B | 22,98096954 | 0,039728043 | 18,74416721 | 0,111272268 |
| True | Ge0288 | Ge0288 | mi R-214_1 | RNU 6 B | 20,8545552 | 0,150301464 | 17,59809473 | 0,052996629 |
| True | Ge0301 | Ge0301 | mi R-214_1 | RNU 6 B | 19,05732753 | 0,107760115 | 17,34236398 | 0,021564228 |
| True | Ge0325 | Ge0325 | mi R-214_1 | RNU 6 B | 23,01075899 | 0,020960033 | 18,10204836 | 0,173935098 |
| True | Ge0326 | Ge0326 | mi R-214_1 | RNU 6 B | 22,46004214 | 0,03951105 | 17,97363246 | 0,190682387 |
| True | Ge0423 | Ge0423 | mi R-214_1 | RNU 6 B | 22,94382321 | 0,02100042 | 18,2152126 | 0,040401979 |
| True | Ge0424 | Ge0424 | mi R-214_1 | RNU 6 B | 22,6838616 | 0,311197475 | 17,04059801 | 0,265255193 |

| Experiment: 090626_Auswertung Active filter: SYBR Green I / HRM Dye (483-533) | | | | | |  |  |  |
| --- | --- | --- | --- | --- | --- | --- | --- | --- |
| Chart | Pairing | Sample Name | Targets | References | Mean Cp | CP Error | Mean Cp | Cp Error |
| True |  | Kontrolle | HS_miR_451_1 | RNU6B | 27,15910725 | 0,397703063 | 18,05133337 | 0,109835873 |
| True | Ge014 | Ge014 | HS_miR_451_1 | RNU6B | 29,52466082 | 0,022825137 | 15,50742163 | 0,318017618 |
| True | Ge017 | Ge017 | HS_miR_451_1 | RNU6B | 21,93173404 | 0,017169275 | 17,5562034 | 0,266013049 |
| True | Ge018 | Ge018 | HS_miR_451_1 | RNU6B | 24,95574076 | 0,060012448 | 16,84584188 | 0,232862241 |
| True | Ge036 | Ge036 | HS_miR_451_1 | RNU6B | 28,0582856 | 0,160933065 | 17,71130268 | 0,063094222 |
| True | Ge055 | Ge055 | HS_miR_451_1 | RNU6B | 29,19223122 | 0,485529345 | 18,08315479 | 0,042197509 |
| True | Ge056 | Ge056 | HS_miR_451_1 | RNU6B | 28,44439473 | 0,10121417 | 19,45246798 | 0,285088526 |
| True | Ge078 | Ge078 | HS_miR_451_1 | RNU6B | 24,96438515 | 0,125395286 | 15,29984323 | 0,147962446 |
| True | Ge079 | Ge079 | HS_miR_451_1 | RNU6B | 24,36156309 | 0,064923818 | 17,6960476 | 0,335869437 |
| True | Ge080 | Ge080 | HS_miR_451_1 | RNU6B | 29,52453219 | 0,171173212 | 18,03344538 | 0,027601474 |
| True | Ge083c | Ge083c | HS_miR_451_1 | RNU6B | 28,56328804 | 0,02914648 | 16,63852505 | 0,148293582 |

| Experiment: 090716_Auswertung Active filter: SYBR Green I / HRM Dye (483-533) | | | | | |  |  |  |
| --- | --- | --- | --- | --- | --- | --- | --- | --- |
| Chart | Pairing | Sample Name | Targets | References | Mean Cp | CP Error | Mean Cp | Cp Error |
| True |  | Kontrolle | HS_miR_451_1 | RNU 6 B | 26,84843224 | 0,02589482 | 18,43898288 | 0,078613228 |
| True | Ge087c | Ge087c | HS_miR_451_1 | RNU 6 B | 28,63131718 | 0,114812583 | 16,19467461 | 0,169021206 |
| True | Ge153 | Ge153 | HS_miR_451_1 | RNU 6 B | 24,69338227 | 0,035239633 | 17,87100505 | 0,076418966 |
| True | Ge107 | Ge107 | HS_miR_451_1 | RNU 6 B | 24,34352037 | 0,075739391 | 16,41808033 | 0,29593839 |
| True | Ge109 | Ge109 | HS_miR_451_1 | RNU 6 B | 26,23124336 | 0,061765715 | 16,81344821 | 0,084637205 |
| True | Ge111 | Ge111 | HS_miR_451_1 | RNU 6 B | 25,37570184 | 0,079362957 | 16,78937175 | 0,076122682 |
| True | Ge128 | Ge128 | HS_miR_451_1 | RNU 6 B | 24,87118594 | 0,124013449 | 16,74224448 | 0,254968684 |
| True | Ge143 | Ge143 | HS_miR_451_1 | RNU 6 B | 28,89583907 | 0,211911261 | 17,37952549 | 0,079776716 |
| True | Ge151 | Ge151 | HS_miR_451_1 | RNU 6 B | 27,17202676 | 0,095153238 | 16,90848151 | 0,037374411 |
| True | Ge157 | Ge157 | HS_miR_451_1 | RNU 6 B | 25,89474924 | 0,048836963 | 18,10012552 | 0,064252615 |
| True | Ge159 | Ge159 | HS_miR_451_1 | RNU 6 B | 26,6910106 | 0,148711859 | 17,68498633 | 0,048671773 |

| Experiment: 090716_Auswertung Active filter: SYBR Green I / HRM Dye (483-533) | | | | | |  |  |  |
| --- | --- | --- | --- | --- | --- | --- | --- | --- |
| Chart | Pairing | Sample Name | Targets | References | Mean Cp | CP Error | Mean Cp | Cp Error |
| True |  | Kontrolle | HS_miR_451_1 | RNU 6 B | 26,84843224 | 0,02589482 | 18,38576467 | 0,051178979 |
| True | Ge0257 | Ge0257 | HS_miR_451_1 | RNU 6 B | 26,55169167 | 0,252545088 | 19,57170959 | 0,124323842 |
| True | Ge0259 | Ge0259 | HS_miR_451_1 | RNU 6 B | 27,14209272 | 0,171655499 | 19,29752585 | 0,232975695 |
| True | Ge0262 | Ge0262 | HS_miR_451_1 | RNU 6 B | 28,01065941 | 0,172351795 | 19,55758626 | 0,018544486 |
| True | Ge0264 | Ge0264 | HS_miR_451_1 | RNU 6 B | 28,18227025 | 0,04476142 | 18,74416721 | 0,111272268 |
| True | Ge0288 | Ge0288 | HS_miR_451_1 | RNU 6 B | 21,91071361 | 0,111201205 | 17,59809473 | 0,052996629 |
| True | Ge0301 | Ge0301 | HS_miR_451_1 | RNU 6 B | 23,86238683 | 0,088183193 | 17,34236398 | 0,021564228 |
| True | Ge0325 | Ge0325 | HS_miR_451_1 | RNU 6 B | 29,97348949 | 0,119531152 | 18,10204836 | 0,173935098 |
| True | Ge0326 | Ge0326 | HS_miR_451_1 | RNU 6 B | 28,21707339 | 0,066935266 | 17,97363246 | 0,190682387 |
| True | Ge0423 | Ge0423 | HS_miR_451_1 | RNU 6 B | 29,3425176 | 0,326629456 | 18,2152126 | 0,040401979 |
| True | Ge0424 | Ge0424 | HS_miR_451_1 | RNU 6 B | 31,09367509 | 0,176357992 | 17,04059801 | 0,265255193 |

| Experiment: 090716_Auswertung Active filter: SYBR Green I / HRM Dye (483-533) | | | | | |  |  |  |
| --- | --- | --- | --- | --- | --- | --- | --- | --- |
| Chart | Pairing | Sample Name | Targets | References | Mean Cp | CP Error | Mean Cp | Cp Error |
| True |  | Kontrolle | HS_miR_451_1 | RNU 6 B | 26,84843224 | 0,02589482 | 18,38576467 | 0,051178979 |
| True | Ge0257 | Ge0257 | HS_miR_451_1 | RNU 6 B | 26,55169167 | 0,252545088 | 19,57170959 | 0,124323842 |
| True | Ge0259 | Ge0259 | HS_miR_451_1 | RNU 6 B | 27,14209272 | 0,171655499 | 19,29752585 | 0,232975695 |
| True | Ge0262 | Ge0262 | HS_miR_451_1 | RNU 6 B | 28,01065941 | 0,172351795 | 19,55758626 | 0,018544486 |
| True | Ge0264 | Ge0264 | HS_miR_451_1 | RNU 6 B | 28,18227025 | 0,04476142 | 18,74416721 | 0,111272268 |
| True | Ge0288 | Ge0288 | HS_miR_451_1 | RNU 6 B | 21,91071361 | 0,111201205 | 17,59809473 | 0,052996629 |
| True | Ge0301 | Ge0301 | HS_miR_451_1 | RNU 6 B | 23,86238683 | 0,088183193 | 17,34236398 | 0,021564228 |
| True | Ge0325 | Ge0325 | HS_miR_451_1 | RNU 6 B | 29,97348949 | 0,119531152 | 18,10204836 | 0,173935098 |
| True | Ge0326 | Ge0326 | HS_miR_451_1 | RNU 6 B | 28,21707339 | 0,066935266 | 17,97363246 | 0,190682387 |
| True | Ge0423 | Ge0423 | HS_miR_451_1 | RNU 6 B | 29,3425176 | 0,326629456 | 18,2152126 | 0,040401979 |
| True | Ge0424 | Ge0424 | HS_miR_451_1 | RNU 6 B | 31,09367509 | 0,176357992 | 17,04059801 | 0,265255193 |

**Results of RT-PCR on blood samples**

Experiment: 2010_04_27_2_bauer Run Auswertung Active filter: SYBR Green I / HRM Dye (483-533)

Chart Pairing Sample Name Targets References Mean Cp CP Error Mean Cp Cp Error

True Kontrolle Hs_miR-126*_1 RNU 6 B 27,3853223 0,079948153 24,39301095 0,246702475

True C4/C4 Vb142 Hs_miR-126*_1 RNU 6 B 29,08611944 0,085508774 26,53860947 0,1890463

True C5/C5 Vb143 Hs_miR-126*_1 RNU 6 B 29,36055505 0,079549267 26,56791356 1,325848025

True C6/C6 Vb134 Hs_miR-126*_1 RNU 6 B 26,58979935 1,034954003 25,27544766 1,999717941

True C7/C7 Vb088 Hs_miR-126*_1 RNU 6 B 26,93710294 0,21924982 29,00612027 2,684202972

True C8/C8 Vb100 Hs_miR-126*_1 RNU 6 B 28,51170235 0,198943956 31,02540875 2,152551617

True C9/C9 Vb099 Hs_miR-126*_1 RNU 6 B 28,11392306 0,049820707 30,39284231 1,380428702

True C10/C10 Vb139 Hs_miR-126*_1 RNU 6 B 27,82054849 0,236772101 27,0676239 0,58407504

True C11/C11 Vb092 Hs_miR-126*_1 RNU 6 B 28,00479286 0,710962583 30,53905167 1,339458602

True C12/C12 Vb155 Hs_miR-126*_1 RNU 6 B 27,14964844 0,084029109 27,75705942 2,28324141

True C13/C13 Vb072 Hs_miR-126*_1 RNU 6 B 27,59938916 0,099002598 31,10740862 0,811319129

True F4/F4 Vb141 Hs_miR-126*_1 RNU 6 B 27,58674348 0,148003116 25,54029789 3,787527211

True F5/F5 Vb137 Hs_miR-126*_1 RNU 6 B 28,24489246 0,254016997 28,06248193 1,167540595

True F6/F6 Vb132 Hs_miR-126*_1 RNU 6 B 28,0249379 0,420113191 28,08705348 2,326903381

True F7/F7 Vb045 Hs_miR-126*_1 RNU 6 B 27,06671208 0,613830364 31,90419213 0,990948948

True F8/F8 Vb059 Hs_miR-126*_1 RNU 6 B 26,38535621 0,109133976 29,1817375 0,186545955

True F9/F9 Vb161 Hs_miR-126*_1 RNU 6 B 28,42246708 0,215934548 29,94269177 0,807914509

True F10/F10 Vb094 Hs_miR-126*_1 RNU 6 B 27,96642314 0,085132157 30,34457085 0,78997889

True F11/F11 Vb096 Hs_miR-126*_1 RNU 6 B 28,53853086 0,337983003 31,44715996 1,667537302

True F12/F12 Vb108 Hs_miR-126*_1 RNU 6 B 27,04498129 0,048828685 27,87056218 2,920421943

True F13/F13 Vb138 Hs_miR-126*_1 RNU 6 B 29,00267468 0,069846893 29,52742858 1,236019532

True I4/I4 Vb140 Hs_miR-126*_1 RNU 6 B 29,98335669 0,080198514 30,93931658 0,620090976

True I5/I5 Vb167 Hs_miR-126*_1 RNU 6 B 28,41646005 0,198403619 28,95717478 1,121583915

True I6/I6 Vb081 Hs_miR-126*_1 RNU 6 B 26,56321352 0,93542752 28,41105075 1,344984731

True I7/I7 Vb131 Hs_miR-126*_1 RNU 6 B 28,70228367 0,196663506 29,55734153 0,602085397

True I8/I8 Vb058 Hs_miR-126*_1 RNU 6 B 26,50177536 0,037899008 29,46442257 0,963830109

True I9/I9 Vb080 Hs_miR-126*_1 RNU 6 B 26,5385979 0,201306569 28,36684459 0,497888347

True I10/I10 Vb121 Hs_miR-126*_1 RNU 6 B 26,88866582 0,213660523 27,30070626 0,451179424

True I11/I11 Vb086 Hs_miR-126*_1 RNU 6 B 27,30622062 0,177213444 29,8274652 1,467077335

True I12/I12 Vb071 Hs_miR-126*_1 RNU 6 B 27,03387253 0,020852912 30,09518211 1,112668174

True I13/I13 Vb062 Hs_miR-126*_1 RNU 6 B 26,511827 0,091446464 29,03160067 0,714782861

Experiment: 2010_04_28_1_bauer Run Auswertung Active filter: SYBR Green I / HRM Dye (483-533)

Chart Pairing Sample Name Targets References Mean Cp CP Error Mean Cp Cp Error

True Kontrolle Hs_miR-130b*_1 RNU 6 B 26,92871193 0,267553783 24,39301095 0,246702475

True C4/C4 Vb142 Hs_miR-130b*_1 RNU 6 B 27,27074871 0,24814731 26,53860947 0,1890463

True C5/C5 Vb143 Hs_miR-130b*_1 RNU 6 B 27,17442492 0,256521926 26,56791356 1,325848025

True C6/C6 Vb134 Hs_miR-130b*_1 RNU 6 B 27,42319273 0,187316945 25,27544766 1,999717941

True C7/C7 Vb088 Hs_miR-130b*_1 RNU 6 B 27,74258541 0,11794617 29,00612027 2,684202972

True C8/C8 Vb100 Hs_miR-130b*_1 RNU 6 B 27,59566318 0,470461778 31,02540875 2,152551617

True C9/C9 Vb099 Hs_miR-130b*_1 RNU 6 B 27,86042655 0,171551001 30,39284231 1,380428702

True C10/C10 Vb139 Hs_miR-130b*_1 RNU 6 B 27,22928286 0,199900604 27,0676239 0,58407504

True C11/C11 Vb092 Hs_miR-130b*_1 RNU 6 B 27,60967416 0,074025911 30,53905167 1,339458602

True C12/C12 Vb155 Hs_miR-130b*_1 RNU 6 B 27,90437366 0,081873865 27,75705942 2,28324141

True C13/C13 Vb072 Hs_miR-130b*_1 RNU 6 B 27,17869608 0,121865303 31,10740862 0,811319129

True F4/F4 Vb141 Hs_miR-130b*_1 RNU 6 B 27,61238131 0,283163086 25,54029789 3,787527211

True F5/F5 Vb137 Hs_miR-130b*_1 RNU 6 B 27,46898785 0,20425278 28,06248193 1,167540595

True F6/F6 Vb132 Hs_miR-130b*_1 RNU 6 B 27,94944768 0,483381719 28,08705348 2,326903381

True F7/F7 Vb045 Hs_miR-130b*_1 RNU 6 B 27,79368155 0,283667414 31,90419213 0,990948948

True F8/F8 Vb059 Hs_miR-130b*_1 RNU 6 B 27,61719056 0,08865074 29,1817375 0,186545955

True F9/F9 Vb161 Hs_miR-130b*_1 RNU 6 B 27,81289345 0,214388136 29,94269177 0,807914509

True F10/F10 Vb094 Hs_miR-130b*_1 RNU 6 B 27,63101577 0,084292793 30,34457085 0,78997889

True F11/F11 Vb096 Hs_miR-130b*_1 RNU 6 B 27,8822392 0,436836389 31,44715996 1,667537302

True F12/F12 Vb108 Hs_miR-130b*_1 RNU 6 B 27,6337231 0,081607352 27,87056218 2,920421943

True F13/F13 Vb138 Hs_miR-130b*_1 RNU 6 B 27,7250619 0,233919633 29,52742858 1,236019532

True I4/I4 Vb140 Hs_miR-130b*_1 RNU 6 B 27,23830379 0,373507145 30,93931658 0,620090976

True I5/I5 Vb167 Hs_miR-130b*_1 RNU 6 B 27,54050914 0,107043037 28,95717478 1,121583915

True I6/I6 Vb081 Hs_miR-130b*_1 RNU 6 B 27,34912856 0,085870923 28,41105075 1,344984731

True I7/I7 Vb131 Hs_miR-130b*_1 RNU 6 B 27,4614542 0,454656249 29,55734153 0,602085397

True I8/I8 Vb058 Hs_miR-130b*_1 RNU 6 B 27,41356403 0,151888268 29,46442257 0,963830109

True I9/I9 Vb080 Hs_miR-130b*_1 RNU 6 B 27,40432288 0,215112 28,36684459 0,497888347

True I10/I10 Vb121 Hs_miR-130b*_1 RNU 6 B 27,49615 0,137680562 27,30070626 0,451179424

True I11/I11 Vb086 Hs_miR-130b*_1 RNU 6 B 27,55253365 0,366845312 29,8274652 1,467077335

True I12/I12 Vb071 Hs_miR-130b*_1 RNU 6 B 27,35678278 0,264703228 30,09518211 1,112668174

True I13/I13 Vb062 Hs_miR-130b*_1 RNU 6 B 27,44410396 0,202971679 29,03160067 0,714782861

Experiment: 2010_04_23_bauer Run Auswertung Active filter: SYBR Green I / HRM Dye (483-533)

Chart Pairing Sample Name Targets References Mean Cp CP Error Mean Cp Cp Error

True Kontrolle HS_miR_150_1 RNU 6 B 24,5874664 0,033973181 24,39301095 0,246702475

True C4/C4 Vb142 HS_miR_150_1 RNU 6 B 26,69073468 0,083073773 26,53860947 0,1890463

True C5/C5 Vb143 HS_miR_150_1 RNU 6 B 24,83535115 0,423592401 26,56791356 1,325848025

True C6/C6 Vb134 HS_miR_150_1 RNU 6 B 25,59266116 0,060053138 25,27544766 1,999717941

True C7/C7 Vb088 HS_miR_150_1 RNU 6 B 25,5012132 0,306668465 29,00612027 2,684202972

True C8/C8 Vb100 HS_miR_150_1 RNU 6 B 24,01199669 0,201915216 31,02540875 2,152551617

True C9/C9 Vb099 HS_miR_150_1 RNU 6 B 25,32187659 0,1323879 30,39284231 1,380428702

True C10/C10 Vb139 HS_miR_150_1 RNU 6 B 24,69527738 0,169101212 27,0676239 0,58407504

True C11/C11 Vb092 HS_miR_150_1 RNU 6 B 24,89811045 0,037593779 30,53905167 1,339458602

True C12/C12 Vb155 HS_miR_150_1 RNU 6 B 23,72428603 0,382840939 27,75705942 2,28324141

True C13/C13 Vb072 HS_miR_150_1 RNU 6 B 24,24231037 0,125440132 31,10740862 0,811319129

True F4/F4 Vb141 HS_miR_150_1 RNU 6 B 0 0 25,54029789 3,787527211

True F5/F5 Vb137 HS_miR_150_1 RNU 6 B 23,98405375 0,274375197 28,06248193 1,167540595

True F6/F6 Vb132 HS_miR_150_1 RNU 6 B 25,09057739 0,085808161 28,08705348 2,326903381

True F7/F7 Vb045 HS_miR_150_1 RNU 6 B 24,63714726 0,365994081 31,90419213 0,990948948

True F8/F8 Vb059 HS_miR_150_1 RNU 6 B 24,52325378 0,182765019 29,1817375 0,186545955

True F9/F9 Vb161 HS_miR_150_1 RNU 6 B 25,46400932 0,212394713 29,94269177 0,807914509

True F10/F10 Vb094 HS_miR_150_1 RNU 6 B 24,75944449 0,045876887 30,34457085 0,78997889

True F11/F11 Vb096 HS_miR_150_1 RNU 6 B 25,58895827 0,159103831 31,44715996 1,667537302

True F12/F12 Vb108 HS_miR_150_1 RNU 6 B 24,83850146 0,182634632 27,87056218 2,920421943

True F13/F13 Vb138 HS_miR_150_1 RNU 6 B 25,47415081 0,227829129 29,52742858 1,236019532

True I4/I4 Vb140 HS_miR_150_1 RNU 6 B 26,70376616 0,030368491 30,93931658 0,620090976

True I5/I5 Vb167 HS_miR_150_1 RNU 6 B 25,95039798 0,129901495 28,95717478 1,121583915

True I6/I6 Vb081 HS_miR_150_1 RNU 6 B 25,10873015 0,09742133 28,41105075 1,344984731

True I7/I7 Vb131 HS_miR_150_1 RNU 6 B 25,33326753 0,225386584 29,55734153 0,602085397

True I8/I8 Vb058 HS_miR_150_1 RNU 6 B 24,47657012 0,018462677 29,46442257 0,963830109

True I9/I9 Vb080 HS_miR_150_1 RNU 6 B 24,57455113 0,047266368 28,36684459 0,497888347

True I10/I10 Vb121 HS_miR_150_1 RNU 6 B 24,34439161 0,227927044 27,30070626 0,451179424

True I11/I11 Vb086 HS_miR_150_1 RNU 6 B 25,08222286 0,095778018 29,8274652 1,467077335

True I12/I12 Vb071 HS_miR_150_1 RNU 6 B 24,45416601 0,245601575 30,09518211 1,112668174

True I13/I13 Vb062 HS_miR_150_1 RNU 6 B 23,03883519 0,205978028 29,03160067 0,714782861

Experiment: 2010_04_29_2_bauer Run Protocol Active filter: SYBR Green I / HRM Dye (483-533)

Chart Pairing Sample Name Targets References Mean Cp CP Error Mean Cp Cp Error

True Kontrolle Hs_miR-576-5p_1 RNU 6 B 28,39229158 0,326340418 24,39301095 0,246702475

True C4/C4 Vb142 Hs_miR-576-5p_1 RNU 6 B 29,02646789 0,091855108 26,53860947 0,1890463

True C5/C5 Vb143 Hs_miR-576-5p_1 RNU 6 B 28,84941928 0,218170076 26,56791356 1,325848025

True C6/C6 Vb134 Hs_miR-576-5p_1 RNU 6 B 28,98178553 0,086833449 25,27544766 1,999717941

True C7/C7 Vb088 Hs_miR-576-5p_1 RNU 6 B 29,30612331 0,191084498 29,00612027 2,684202972

True C8/C8 Vb100 Hs_miR-576-5p_1 RNU 6 B 29,40724117 0,392651354 31,02540875 2,152551617

True C9/C9 Vb099 Hs_miR-576-5p_1 RNU 6 B 29,29977301 0,054758039 30,39284231 1,380428702

True C10/C10 Vb139 Hs_miR-576-5p_1 RNU 6 B 29,13979203 0,346440201 27,0676239 0,58407504

True C11/C11 Vb092 Hs_miR-576-5p_1 RNU 6 B 29,42288034 0,201318474 30,53905167 1,339458602

True C12/C12 Vb155 Hs_miR-576-5p_1 RNU 6 B 29,63366205 0,114683994 27,75705942 2,28324141

True C13/C13 Vb072 Hs_miR-576-5p_1 RNU 6 B 28,97648662 0,143566899 31,10740862 0,811319129

True F4/F4 Vb141 Hs_miR-576-5p_1 RNU 6 B 29,01685691 0,233730864 25,54029789 3,787527211

True F5/F5 Vb137 Hs_miR-576-5p_1 RNU 6 B 28,8156545 0,163552554 28,06248193 1,167540595

True F6/F6 Vb132 Hs_miR-576-5p_1 RNU 6 B 29,43224158 0,281866138 28,08705348 2,326903381

True F7/F7 Vb045 Hs_miR-576-5p_1 RNU 6 B 29,13840073 0,520180652 31,90419213 0,990948948

True F8/F8 Vb059 Hs_miR-576-5p_1 RNU 6 B 29,1530033 0,175269881 29,1817375 0,186545955

True F9/F9 Vb161 Hs_miR-576-5p_1 RNU 6 B 29,31836602 0,569252542 29,94269177 0,807914509

True F10/F10 Vb094 Hs_miR-576-5p_1 RNU 6 B 29,25629766 0,484577572 30,34457085 0,78997889

True F11/F11 Vb096 Hs_miR-576-5p_1 RNU 6 B 29,33285849 0,599926519 31,44715996 1,667537302

True F12/F12 Vb108 Hs_miR-576-5p_1 RNU 6 B 29,31491949 0,233032303 27,87056218 2,920421943

True F13/F13 Vb138 Hs_miR-576-5p_1 RNU 6 B 29,04303952 0,290976276 29,52742858 1,236019532

True I4/I4 Vb140 Hs_miR-576-5p_1 RNU 6 B 29,09006151 0,062687243 30,93931658 0,620090976

True I5/I5 Vb167 Hs_miR-576-5p_1 RNU 6 B 29,09885976 0,140340464 28,95717478 1,121583915

True I6/I6 Vb081 Hs_miR-576-5p_1 RNU 6 B 28,80242688 0,236620953 28,41105075 1,344984731

True I7/I7 Vb131 Hs_miR-576-5p_1 RNU 6 B 29,23661301 0,092519687 29,55734153 0,602085397

True I8/I8 Vb058 Hs_miR-576-5p_1 RNU 6 B 28,9424426 0,457837129 29,46442257 0,963830109

True I9/I9 Vb080 Hs_miR-576-5p_1 RNU 6 B 28,77464712 0,165552687 28,36684459 0,497888347

True I10/I10 Vb121 Hs_miR-576-5p_1 RNU 6 B 28,83607669 0,310152463 27,30070626 0,451179424

True I11/I11 Vb086 Hs_miR-576-5p_1 RNU 6 B 29,01375109 0,115289066 29,8274652 1,467077335

True I12/I12 Vb071 Hs_miR-576-5p_1 RNU 6 B 29,23317884 0,112718739 30,09518211 1,112668174

True I13/I13 Vb062 Hs_miR-576-5p_1 RNU 6 B 28,61399569 0,139014228 29,03160067 0,714782861
