# Supplementary material for: Diagnosis of Pancreatic Ductal Adenocarcinoma and Chronic Pancreatitis by Measurement of microRNA Abundance in Blood and Tissue
Source: PLoS One. 2012 Apr 12;7(4):e34151. doi: 10.1371/journal.pone.0034151 (PMC3325244; doi:10.1371/journal.pone.0034151)
Supplement: Table S4 — Basic data set of miRNA expression. (DOC) [file pone.0034151.s005.doc]

**Diagnosis of pancreatic ductal adenocarcinoma and chronic pancreatitis by measurement of microRNA abundance in blood and tissue**

**Supplemental Table S4:**

**Basic data set of miRNA expression**

**Values normal vs. cancer in blood**

name median g1 median g2 qmedian logqmedian wmw_rawp wmw_adjp ttest_rawp ttest_adjp AUC

hsa-miR-579 39.2631578947368 24.0175438596491 1.63476990504017 0.491502063583421 0.0404717852724389 0.131557315248179 0.138855425034239 0.300331408031449 0.636363636363636

hsa-miR-663b 73.5964912280701 74.0175438596491 0.994311448210476 -0.00570479322303208 0.991924219396671 0.9965431913147 0.57084378774572 0.72691853446743 0.501010101010101

hsa-miR-106b 14207.2368421053 7477.03728070175 1.90011582244938 0.641914843498342 1.6753750093366e-05 0.00236979110557927 3.92268415836997e-05 0.00308503027500963 0.786531986531987

hsa-miR-449b 39.2631578947368 71.0701754385965 0.552456183658356 -0.59338115433082 0.00197405349354005 0.0191416647744389 0.000192285437365207 0.00622962664748669 0.293939393939394

hsa-miR-561 44.7017543859649 23.6491228070176 1.89020771513353 0.636686725219801 0.161723357248398 0.342917094116383 0.228219837686386 0.406090144171858 0.592592592592593

hsa-miR-524-3p 15.7894736842105 15.8771929824561 0.994475138121547 -0.00554018037561558 0.515120468571816 0.687092680645251 0.0504947162558573 0.155176251230165 0.457575757575758

hsa-miR-655 31.8771929824561 46.9473684210526 0.678998505231689 -0.387136352851948 0.166364373635301 0.349308254772578 0.0437818041603353 0.1427521468654 0.408080808080808

hsa-miR-30c-2S 15.7894736842105 3.55701754385965 4.43896424167694 1.49042107032879 0.827167024164111 0.905894850068056 0.788523192384176 0.868232348409909 0.514141414141414

hsa-miR-299-5p 27.140350877193 31.7543859649123 0.854696132596686 -0.157009273686805 0.165592379856216 0.349079297988449 0.0477611144056032 0.150981105245552 0.407744107744108

hsa-miR-369-5p 15.6315789473684 29.6140350877193 0.527843601895734 -0.638955247685093 0.0483583930960477 0.143413378838107 0.00976733172868382 0.0554307254981124 0.36969696969697

hsa-miR-452 55.8947368421053 28.6842105263158 1.94862385321101 0.66712340713864 0.0535842406858996 0.15517852252326 0.353150450234272 0.54812746291154 0.628619528619529

hsa-miR-934 110.741228070175 91.5614035087719 1.20947499520981 0.190186377195548 0.0585584765532717 0.165370978285339 0.0440000823478523 0.1427521468654 0.626262626262626

hsa-miR-483-5p 168.44298245614 159.09649122807 1.05874731212439 0.0570864282554138 0.415512044752529 0.606793383317771 0.0263160886142235 0.103702212210388 0.445454545454545

hsa-miR-340S 10.3684210526316 20.859649122807 0.497056349873844 -0.699051879284684 0.056050491650585 0.161415121512487 0.0321878919875814 0.116540738488471 0.375757575757576

hsa-miR-340 169.236842105263 152.219298245614 1.11179623119922 0.105976933712469 0.240700974052029 0.440095213150213 0.370799756194883 0.562390491381695 0.578451178451178

hsa-miR-608 64.9122807017543 178.5 0.363654233623274 -1.01155177042193 0.000209761087642658 0.00543451823935425 0.000553729160060856 0.0122530324392954 0.252861952861953

hsa-miR-1539 96.4210526315789 84.7456140350878 1.13777041714108 0.12907057294462 0.270247170500414 0.474031114109466 0.321693826483341 0.516294260518772 0.573737373737374

hsa-miR-802 88.7368421052632 42.0350877192982 2.1110183639399 0.747170467994571 0.0187867189322662 0.0802620714779491 0.0574494051666158 0.170374009136733 0.656228956228956

hsa-miR-654-3p 25.6666666666667 23.6491228070176 1.0853115727003 0.0818671095478719 0.890835593701043 0.939271118785912 0.281659310383554 0.478317978963018 0.490572390572391

hsa-miR-507 24.0175438596491 21.0657894736842 1.14012075785967 0.131134184743515 0.334106157197569 0.538941333946732 0.114407417461191 0.267570735146363 0.436700336700337

hsa-miR-637 3.08771929824561 40.2807017543859 0.0766550522648084 -2.5684397624013 0.00861118638652296 0.0488911437603245 0.000991052569070401 0.0164500597341995 0.329292929292929

hsa-miR-1228 233.517543859649 458.607456140351 0.509188284518829 -0.674937420185661 0.0231684206544679 0.092566421411138 0.0111757587150347 0.0602792485692185 0.348484848484849

hsa-miR-626 29.8245614035088 30.140350877193 0.989522700814901 -0.010532572499893 0.822653568626923 0.904270714629757 0.382098300886744 0.572484086224409 0.484848484848485

hsa-miR-151-5p 3921.35964912281 3921.35964912281 1 0 0.57393531037834 0.7378816663937 0.327011594461347 0.522612974111375 0.537710437710438

hsa-miR-431S 11.8947368421053 16.8245614035088 0.706986444212721 -0.346743786943043 0.625856984236334 0.772696105001368 0.0917383594507832 0.230538738200371 0.467676767676768

hsa-miR-29aS 1 4.70175438596492 0.212686567164179 -1.54793571267631 0.00614951024036892 0.03845671983651 0.0161741372426245 0.0788603414711015 0.335690235690236

hsa-miR-532-5p 237.973684210526 128.53399122807 1.85144553543247 0.615966704483263 3.44739601446305e-05 0.00236979110557927 0.000143265760632474 0.00537558049677501 0.776094276094276

hsa-miR-27a 317.587719298246 357.271929824561 0.888924353654644 -0.117743138590807 0.831789517330008 0.906365640348179 0.733547486077783 0.829687392509996 0.485521885521886

hsa-miR-556-5p 155.870614035088 69.8245614035087 2.23231783919598 0.803040435454462 5.54660557904789e-05 0.00287022034727864 5.03025274355236e-05 0.00308503027500963 0.768686868686869

hsa-miR-425 9651.24561403509 12702.7105263158 0.759778442092411 -0.274728411765631 0.00304612564493989 0.0238982402871194 0.0485572266084358 0.151829299141594 0.303367003367003

hsa-miR-615-5p 51.9649122807017 48.8771929824561 1.06317300789663 0.0612578404879926 0.567559419203154 0.731223976439435 0.139732215633188 0.30055977564424 0.461616161616162

hsa-miR-519c-3p 46.5263157894737 21.2741228070176 2.18699103185239 0.782526641024305 0.116829229010857 0.269886420181406 0.0813075061657868 0.212631447942648 0.604377104377104

hsa-miR-486-3p 289.342105263158 274.609649122807 1.0536487198735 0.0522591117156348 0.91542248859497 0.95758907527813 0.863570898545592 0.922353571095106 0.507407407407407

hsa-miR-525-3p 79.0877192982456 62.563596491228 1.26411721406288 0.234374024069946 0.341725099835709 0.545122727659905 0.426444833133599 0.607296849825571 0.563636363636364

hsa-miR-140-5p 6.77192982456141 9.92982456140352 0.681978798586573 -0.382756708738351 0.676581717557485 0.809833595356602 0.138169616671272 0.299598942681678 0.473063973063973

hsa-miR-20bS 84.9912280701755 64.4736842105263 1.31823129251701 0.276290908178123 0.0281807173310215 0.105738952420311 0.1588819914575 0.324750240730857 0.646464646464646

hsa-miR-526bS 3.08771929824561 3.71710526315789 0.830678466076697 -0.185512483096562 0.440013337682852 0.62219517257345 0.028911360894434 0.109432037069722 0.450841750841751

hsa-miR-30bS 18.3684210526316 43.0877192982456 0.426302931596091 -0.852605078396496 0.0444949017487844 0.139203002052117 0.0597006888464028 0.174649811777782 0.367003367003367

hsa-miR-136S 72.8157894736842 56.3508771929825 1.29218555417185 0.256335012828437 0.167264397678921 0.349308254772578 0.896496662962861 0.941212433256629 0.592255892255892

hsa-miR-1911 10 45.2631578947369 0.22093023255814 -1.50990831708707 0.00990915938411137 0.0541240794208108 0.0179665446111923 0.0850112003295043 0.32962962962963

hsa-miR-628-3p 178.149122807018 129.587719298246 1.37473769715021 0.318262947211501 0.0169911460538906 0.0744332946421705 0.0217885028137787 0.09367402034532 0.659259259259259

hsa-miR-629S 92.5263157894737 118 0.784121320249777 -0.243191525386983 0.226779261852302 0.428861419211079 0.172075129305097 0.34295805217159 0.419191919191919

hsa-miR-603 178.5 150.850877192982 1.18328778275281 0.16829682062999 0.32906186912702 0.534803000106626 0.878778528085801 0.931869160506611 0.565319865319865

hsa-miR-518d-3p 98.4649122807017 52.9824561403509 1.85844370860927 0.619739421681594 0.0108023254748967 0.0571926802750665 0.0556219657330807 0.167253506716546 0.67003367003367

hsa-miR-23b 2872.95175438596 3749.71929824561 0.766177819158393 -0.266340996288378 4.11898801664995e-05 0.00236979110557927 2.24118880476435e-05 0.00308503027500963 0.226936026936027

hsa-miR-23aS 1 1 1 0 0.340454910196339 0.545122727659905 0.136665710765649 0.29708440400694 0.441750841750842

hsa-miR-939 46.5964912280702 77.1929824561403 0.603636363636364 -0.504783309310027 0.0319991377635532 0.114178454116742 0.0498554524574986 0.154212385200076 0.356902356902357

hsa-miR-662 53.2982456140351 71.7368421052631 0.742968941061385 -0.297101037208923 0.196878615878076 0.390589070121332 0.0546388323712527 0.165450218724179 0.413804713804714

hsa-miR-1178 19.9649122807018 34.6842105263158 0.575619625695498 -0.552308208484341 0.105268576487376 0.251602244284499 0.0787829315645614 0.208388270462998 0.392592592592593

hsa-miR-519b-5p 163.745614035088 87.9122807017544 1.86260227499501 0.621974582512926 0.000214106164702253 0.00543451823935425 0.000241102606941613 0.00743112677823616 0.746801346801347

hsa-miR-409-5p 93.7543859649123 88.9122807017544 1.05445935280189 0.0530281737754517 0.645384744276513 0.787789298883495 0.628196191854522 0.77046594405452 0.530976430976431

hsa-miR-567 61.859649122807 80.8771929824561 0.764859002169197 -0.268063773032879 0.220785499211685 0.422920256809363 0.0579200003666219 0.171181370946557 0.418181818181818

hsa-miR-600 71.0701754385965 77.1929824561403 0.920681818181818 -0.0826407767036619 0.734686854719498 0.84203753875139 0.568676162165757 0.725987467380249 0.522895622895623

hsa-miR-1207-5p 357.271929824561 334.570175438596 1.06785349099394 0.0656505504105867 0.91542248859497 0.95758907527813 0.68208856316525 0.799785910341863 0.507407407407407

hsa-miR-1291 136.513157894737 144.131578947368 0.94714259631185 -0.0543056202447668 0.812130437934784 0.896251365649256 0.755703786872879 0.846977101391292 0.516161616161616

hsa-miR-145 100.912280701754 192.657894736842 0.523790010472158 -0.646664418353924 0.100277178817672 0.244461031976416 0.0152650862467443 0.0761489562482098 0.39023569023569

hsa-miR-636 162.061403508772 114.561403508772 1.4146248085758 0.34686434298598 0.0024942200347959 0.0201169335516716 0.00604089751000866 0.0420426979930441 0.701683501683502

hsa-miR-541 132.684210526316 57.1578947368421 2.32136279926335 0.84215442669404 1.43812683510789e-06 0.000460424534777681 2.78220013449597e-05 0.00308503027500963 0.821212121212121

hsa-miR-367S 80 67.4736842105263 1.18564742589704 0.170288976359707 0.674519584817311 0.809799790900936 0.716818678490789 0.820874802561432 0.528282828282828

hsa-miR-497 181.407894736842 154.285087719298 1.17579668533417 0.161945947909215 0.155304799835406 0.333403090193919 0.404571061276295 0.589771665340275 0.594949494949495

hsa-miR-34aS 116.723684210526 116.5 1.00192003614186 0.00191819522850055 0.915424087027181 0.95758907527813 0.812917088497039 0.881341014287619 0.492592592592593

hsa-miR-126 1907.68201754386 1907.68201754386 1 0 0.491415249892927 0.66603083806427 0.61351334012681 0.759629860157011 0.546127946127946

hsa-miR-1255a 42.0350877192982 52.9824561403509 0.793377483443709 -0.231456151133575 0.323204122327961 0.528400259759746 0.121760933207927 0.27579969910352 0.434006734006734

hsa-miR-519b-3p 26.1228070175439 39.6666666666667 0.658558160106148 -0.41770243959396 0.06085527585968 0.170513321645792 0.00881393815568918 0.0520988262216422 0.375757575757576

hsa-miR-377S 1 1 1 0 0.0586367547570262 0.165370978285339 0.00474379746587041 0.0359835339371774 0.401346801346801

hsa-miR-187S 151.552631578947 191.956140350877 0.78951697664854 -0.236333942479578 0.381620380429086 0.579821106180108 0.213739753443433 0.390799591571362 0.441414141414141

hsa-miR-1538 77.0175438596491 58.3333333333333 1.3203007518797 0.277859552979262 0.598561797804424 0.751905140473388 0.613014045082727 0.759629860157011 0.535353535353535

hsa-miR-331-5p 35.3508771929824 55.8070175438597 0.633448601068846 -0.456576417368361 0.0347118123816667 0.119347785200711 0.0185385912856304 0.086950023258147 0.359259259259259

hsa-miR-219-1-3p 18.6315789473684 23.6491228070176 0.787833827893175 -0.238468089670368 0.934288408363809 0.969200602800134 0.859340731364179 0.920112966708792 0.505723905723906

hsa-miR-493S 39.2631578947368 25.9298245614035 1.51420838971583 0.414892787363722 0.767901385568594 0.866126750668853 0.70709415131478 0.81471595805695 0.51986531986532

hsa-miR-191S 59.5438596491228 57.3333333333333 1.03855569155447 0.0378309898142779 0.855467163056769 0.919387499026142 0.218693066103929 0.397330770626717 0.512457912457913

hsa-miR-323-3p 41.8771929824561 45.7192982456141 0.915963161933998 -0.087779131345961 0.983804287777295 0.995974060001018 0.958292711049618 0.981028006685433 0.498316498316498

hsa-miR-199a-5p 248.256578947368 247.412280701754 1.00341251551143 0.00340670609311164 0.557455135424548 0.721265040286934 0.6742847259991 0.794321062527226 0.539393939393939

hsa-miR-653 32.7017543859649 25.9298245614035 1.26116373477672 0.232034893737389 0.353882400212546 0.55226132257401 0.0995735635406084 0.24622345368351 0.438383838383838

hsa-miR-384 80.0350877192982 106.157894736842 0.75392497107916 -0.282462423777988 0.406855042825278 0.604405214286028 0.416193340489553 0.600512421022054 0.444444444444444

hsa-miR-125b 498.324561403509 521.372807017544 0.955793157403279 -0.0452137518893601 0.773152395108337 0.868789735649082 0.480237537615907 0.662052707607872 0.48047138047138

hsa-miR-422a 133.280701754386 143.377192982456 0.92958091159376 -0.0730214270951434 0.757715411946125 0.860405790144087 0.786970025119529 0.868232348409909 0.479124579124579

hsa-miR-431 199.447368421053 140.868421052632 1.41584158415842 0.347724113418648 0.497991228005421 0.672560922955678 0.364714606250518 0.556093118717662 0.545454545454546

hsa-miR-769-3p 1 18.6315789473684 0.0536723163841808 -2.92485793396733 0.0119457946659334 0.0602878409163773 0.00229581739838779 0.0245058837767518 0.343097643097643

hsa-miR-155S 80.0350877192982 67.719298245614 1.18186528497409 0.167093940385457 0.309308739782795 0.512348258027931 0.300989466392368 0.496974267490364 0.568013468013468

hsa-miR-548n 33.4385964912281 40.9824561403509 0.815924657534247 -0.20343325973397 0.240299312198651 0.440095213150213 0.0416949033644774 0.138004445623542 0.421885521885522

hsa-miR-9S 112.078947368421 103.719298245614 1.08059878213802 0.0775153154033403 0.843650410970383 0.910087880834301 0.917011201384821 0.958087974328208 0.513468013468014

hsa-miR-1265 1 41.8771929824561 0.0238793464599916 -3.73474135850428 0.00133414442540585 0.0150361655662008 0.00714083398719467 0.0460822998357415 0.294276094276094

hsa-miR-30c 1470.35964912281 2106.82894736842 0.69790176889272 -0.359676918368863 0.00122273410573461 0.0143528571429704 0.00829003876630224 0.0500300940931387 0.284511784511784

hsa-miR-15aS 129.394736842105 124.535087719298 1.03902232866098 0.0382802024164754 0.344259421669962 0.547142752202631 0.71938018636333 0.821849943334403 0.563299663299663

hsa-miR-502-5p 12.4517543859649 13.4385964912281 0.926566579634465 -0.0762693743875108 0.135204366871679 0.300725176830565 0.00703196204910114 0.0460822998357415 0.403367003367003

hsa-miR-1227 77.9298245614035 58.9824561403509 1.32123735871505 0.278568690485812 0.272371896104445 0.475823778012421 0.970777343723763 0.985752953652734 0.573400673400673

hsa-miR-520f 1 1 1 0 0.0307080180055938 0.111520603311205 0.00489706291474061 0.0363906225345639 0.38047138047138

hsa-miR-1910 42.2105263157895 14.8070175438597 2.85071090047393 1.04756840193867 0.0303280746268111 0.110973151787216 0.873550205794165 0.928416043842813 0.642424242424242

hsa-miR-1182 9.92982456140352 16.8245614035088 0.590198123044839 -0.527296996680255 0.432399621700953 0.617816015774706 0.389173799733504 0.58006388457688 0.448484848484848

hsa-miR-622 98.1491228070175 88.7368421052632 1.1060695927244 0.100812824017865 0.85950907612666 0.920293216746039 0.493611697485011 0.672965078877669 0.512121212121212

hsa-miR-361-5p 387.254385964912 404.478070175439 0.957417507942876 -0.0435157152232326 0.971757889267076 0.995974060001018 0.983609194043502 0.990062398821924 0.497306397306397

hsa-miR-1248 24.8421052631579 42.1052631578947 0.590000000000001 -0.527632742082371 0.193535209860323 0.387859978832596 0.0483629695633261 0.151771791756911 0.413468013468013

hsa-miR-129-3p 86.0701754385965 93.5087719298245 0.920450281425891 -0.0828922923414553 0.427223115531415 0.615306068533998 0.377051174042776 0.566890528221108 0.553198653198653

hsa-miR-330-3p 360.043859649123 264.666666666667 1.36036722789341 0.307754683808845 0.0116187184430503 0.0593310888541565 0.548549907717033 0.708941846890861 0.668350168350168

hsa-miR-298 135.688596491228 158.815789473684 0.854377243855289 -0.157382445195402 0.935508808960037 0.969200602800134 0.616425937695743 0.762142670818662 0.494276094276094

hsa-miR-619 80.0350877192982 77.7894736842105 1.02886783942264 0.0284590126680767 0.514150365841157 0.686860318453434 0.175598467085447 0.347572195171424 0.456228956228956

hsa-miR-888 57.578947368421 60.8070175438596 0.946912867859204 -0.0545481986255149 0.223233551061946 0.426218041076238 0.354409182276523 0.54812746291154 0.418855218855219

hsa-miR-944 32.7017543859649 35.3508771929824 0.925062034739454 -0.0778944791352466 0.38529680030377 0.582331241089586 0.663882774366541 0.789160928758023 0.442424242424242

hsa-miR-106a 9071.26754385965 7477.03728070175 1.21321684021459 0.193275377559052 0.0650654484025167 0.179430955132925 0.0956393737589579 0.238545605647343 0.622895622895623

hsa-miR-506 43.3377192982456 49.6491228070175 0.872879858657244 -0.135957351562211 0.383752125006957 0.581256059149886 0.123106417406862 0.276668849536775 0.441750841750842

hsa-miR-200aS 91.0526315789474 96 0.948464912280702 -0.0529104831424517 0.89138244065546 0.939271118785912 0.941420404781442 0.971825130773187 0.490572390572391

hsa-miR-649 10.3684210526316 24.0175438596491 0.431701972242513 -0.840019807882351 0.155885472023386 0.333819261429733 0.0994411820555352 0.24622345368351 0.406397306397306

hsa-miR-500 257.254385964912 148.28947368421 1.73481218574386 0.550899157247616 0.00448148697870814 0.0319630021704555 0.0302941324486054 0.113041939543687 0.68956228956229

hsa-miR-505 10.6315789473684 27.3333333333333 0.388960205391528 -0.944278240361379 0.0528036366971076 0.153951143478391 0.0209486901892501 0.0922620643667372 0.372390572390572

hsa-miR-24-1S 107.157894736842 112.078947368421 0.95609297957267 -0.0449001116928546 0.395558808663082 0.593682177176069 0.655070367109549 0.785141755738159 0.556902356902357

hsa-miR-1908 599.291666666667 494.798245614035 1.21118389561579 0.191598307390551 0.68205711177729 0.813974140892504 0.926183019269104 0.964168812580502 0.527609427609428

hsa-miR-518f 49.4035087719298 74.578947368421 0.662432368854387 -0.411836811080857 0.0426159142811957 0.136213088980266 0.0262222549307539 0.103702212210388 0.364983164983165

hsa-miR-548h 1 16.8245614035088 0.059436913451512 -2.82283980704889 0.00105772816455342 0.0136241702389493 0.0311567969105699 0.114418364824774 0.294612794612795

hsa-miR-423-5p 2106.82894736842 1405.51754385965 1.49897022386709 0.40477835492523 0.00228384837445412 0.0200503012704972 0.111327680903356 0.263221338683826 0.703367003367003

hsa-miR-1185 10 10.8771929824561 0.919354838709677 -0.0840831172105413 1 1 0.538398902689082 0.704930551107513 0.4996632996633

hsa-miR-1302 42.2105263157895 27.3333333333333 1.54428754813864 0.43456267010393 0.547305021320599 0.713611053315229 0.640103480287867 0.774767606575637 0.54006734006734

hsa-miR-18b 354.5 232.09649122807 1.52738198722552 0.423555150306836 0.0192038932251561 0.0808501207125977 0.0255125293694821 0.103702212210388 0.656228956228956

hsa-miR-29b-1S 1 12.2456140350877 0.0816618911174785 -2.50516783492782 0.00584008757121985 0.0375666422245006 0.00346683152153824 0.0305293428886479 0.32996632996633

hsa-miR-937 42.2456140350877 69.9649122807017 0.603811434302909 -0.504493324653017 0.0303472350194472 0.110973151787216 0.0350468928767412 0.121467745191276 0.355555555555556

hsa-miR-573 52.9824561403509 47.4035087719298 1.11769059955588 0.111264591848771 0.641286738888946 0.786412859471274 0.551843384015381 0.709983923769388 0.531313131313131

hsa-miR-339-3p 249.100877192982 265.793859649123 0.937195755845613 -0.0648631009058981 0.700725753811589 0.822756905495784 0.212158760208478 0.389734983784368 0.474074074074074

hsa-miR-199b-5p 1 14.8070175438597 0.0675355450236967 -2.69510122676141 0.0127752610293883 0.06302773945034 0.00334174198705037 0.0297311684002523 0.345454545454546

hsa-miR-93 4503.74342105263 3312.48684210526 1.35962605611144 0.30720970319829 0.0273454825108179 0.103919593967997 0.011110317029698 0.0602792485692185 0.647138047138047

hsa-miR-148aS 36.6666666666667 42.4561403508772 0.863636363636364 -0.146603474191875 0.838878023846495 0.910087880834301 0.536234866854979 0.704930551107513 0.486195286195286

hsa-miR-554 104.491228070175 100.175438596491 1.0430823117338 0.0421800911538437 0.116961206428558 0.269886420181406 0.203453949348989 0.379224099974465 0.604713804713805

hsa-miR-539 1 21.1842105263158 0.0472049689440994 -3.05325611769218 6.91084054614196e-05 0.00298202769566025 7.81362889827168e-06 0.00193019760961628 0.257912457912458

hsa-miR-1252 15.9649122807018 17.6491228070176 0.904572564612325 -0.10029275114879 0.678698006767853 0.81124152332501 0.552330874054168 0.709983923769388 0.472727272727273

hsa-miR-664S 64.0877192982456 69.9649122807017 0.915997993981946 -0.0877411042864465 0.871431004847342 0.927305742519428 0.54676564155673 0.708496619614802 0.488888888888889

hsa-miR-520e 1 1 1 0 0.220906788752042 0.422920256809363 0.0296079828482681 0.11109430086111 0.428282828282828

hsa-miR-329 85.6052631578948 58.7017543859649 1.4583084279737 0.377277153034747 0.0405076704513616 0.131557315248179 0.183299381058505 0.35788996799432 0.636700336700337

hsa-miR-452S 401.754385964912 306.69298245614 1.30995623945314 0.269993731657495 0.142492124105673 0.313700773222438 0.578245683991476 0.733861801889182 0.597979797979798

hsa-miR-1468 1 1 1 0 0.0853082823796865 0.218742293794467 0.0213048573506995 0.092859049967948 0.399326599326599

hsa-miR-519c-5p 147.864035087719 127.548245614035 1.15927925449606 0.147798479680366 0.167387966020091 0.349308254772578 0.195276111979836 0.367954769953272 0.592255892255892

hsa-miR-210 708.070175438597 372.427631578947 1.9012288976429 0.642500465328136 4.0163771085994e-05 0.00236979110557927 0.00958579306920328 0.0547850292630625 0.773737373737374

hsa-let-7gS 145.563596491228 154.285087719298 0.943471586548028 -0.058189029573442 0.581483640269494 0.741244377059759 0.121179795142839 0.27579969910352 0.462962962962963

hsa-let-7eS 2.45614035087718 1 2.45614035087718 0.898591154774751 0.956888502602941 0.987068849426578 0.148828190086789 0.310989656283047 0.503703703703704

hsa-miR-300 55.8947368421053 26.4561403508772 2.11273209549071 0.747981941902092 0.0114187997296711 0.0590085279443483 0.0245952295838107 0.101074681575374 0.667340067340067

hsa-miR-1268 348.622807017544 559.473684210526 0.623126371903418 -0.473005936618038 0.000260837356613093 0.00589551640089027 0.00182854301210504 0.0222258115415021 0.256565656565657

hsa-miR-484 5199.04385964912 5736.38157894737 0.90632810737865 -0.0983538890748744 0.915334435013738 0.95758907527813 0.634674490073611 0.771861211803507 0.507407407407407

hsa-miR-522 1 9.36842105263158 0.106741573033708 -2.23734457112564 0.0126943805080739 0.06302773945034 0.0107498862070822 0.0590901388325603 0.342087542087542

hsa-miR-429 49.4035087719298 57.9649122807017 0.852300242130751 -0.159816417362936 0.753021704939677 0.856893771833878 0.665510100946568 0.790007176226807 0.478787878787879

hsa-miR-497S 126.315789473684 96.719298245614 1.30600399056775 0.266972086414464 0.165843003679333 0.349079297988449 0.0918311839595561 0.230538738200371 0.592592592592593

hsa-miR-146b-5p 139.728070175439 154.741228070175 0.902978940506222 -0.102056047535643 0.879415906772958 0.932353719342829 0.357470463953895 0.549905544371144 0.48956228956229

hsa-miR-1273 103.59649122807 129.587719298246 0.799431395112705 -0.223854560130201 0.198978486677836 0.392948361562866 0.0789605613457708 0.208388270462998 0.414141414141414

hsa-miR-107 1708.83771929825 1799.94736842105 0.949382048208037 -0.051943981609059 0.228278205741119 0.430055654166929 0.325944899643778 0.521874672342449 0.41952861952862

hsa-miR-92a-1S 14.1754385964912 25.2982456140351 0.560332871012483 -0.579224259323799 0.209437635427028 0.407082611201634 0.401034870778717 0.587594386217373 0.417171717171717

hsa-miR-376aS 1 1.57894736842105 0.633333333333336 -0.456758402495711 0.0939806334440515 0.233504671880996 0.0842482025590333 0.218336933358696 0.398653198653199

hsa-miR-133b 8.17543859649124 30.140350877193 0.271245634458673 -1.30473046841855 0.0154181102149271 0.0704012122512278 0.0018647469571468 0.0223510642224679 0.341750841750842

hsa-miR-599 27.140350877193 51.8947368421053 0.522988505747127 -0.648195792697678 0.0235588919511301 0.0932629529991985 0.00816511017114699 0.0496231695612666 0.34983164983165

hsa-miR-378 266.921052631579 168.885964912281 1.58048096400561 0.45772920831477 0.00245237892081827 0.0201169335516716 0.0240344504634182 0.0997198593746632 0.702020202020202

hsa-miR-34c-3p 116.179824561404 95.578947368421 1.21553781204112 0.195186622509949 0.0446804537293584 0.139203002052117 0.172981742552971 0.343179870857964 0.634006734006734

hsa-miR-642 28.0701754385965 51.1578947368421 0.548696844993142 -0.600209184900676 0.0952094094929781 0.234759201121257 0.106889996523484 0.255889924548641 0.388888888888889

hsa-miR-582-3p 89.2280701754386 70.5087719298245 1.26548892759393 0.235458551532864 0.290540049600612 0.490677226624908 0.38712961823742 0.578015329652065 0.570707070707071

hsa-miR-34a 19.4035087719298 43.2478070175439 0.448658790122205 -0.801492613047953 0.00139777937596938 0.0150361655662008 0.000846550465843584 0.0155441074898513 0.287542087542088

hsa-miR-337-5p 39.8245614035088 36.280701754386 1.09767891682785 0.0931978748471287 0.581098477476806 0.741244377059759 0.543856236050516 0.706886175757399 0.537037037037037

hsa-miR-616 15.4736842105263 31.4385964912281 0.4921875 -0.708895537528085 0.123963098196125 0.281798757897983 0.0325520717306191 0.117028603794042 0.397979797979798

hsa-miR-1304 62.3552631578947 62.1951754385965 1.00257395719474 0.00257065024034264 0.931481687269006 0.969200602800134 0.14084232263878 0.301605271556493 0.493939393939394

hsa-miR-760 31.8771929824561 69.6842105263157 0.457452165156093 -0.782082956768892 0.00201565200791633 0.0191722042521038 0.000935265375882911 0.0164500597341995 0.294949494949495

hsa-miR-1226S 191.956140350877 142.28947368421 1.3490536958264 0.299403380604246 0.0169883561178653 0.0744332946421705 0.0292365495493665 0.110179660528836 0.659259259259259

hsa-miR-7-2S 15.4736842105263 40.2105263157895 0.384816753926702 -0.954988021827839 0.0612483282509327 0.171059246862637 0.0593186493941674 0.174531153951576 0.376094276094276

hsa-miR-518cS 61.0175438596491 58.3333333333333 1.04601503759398 0.0449877418242736 0.792532576160455 0.883663582979939 0.728656276450507 0.82740837707472 0.517845117845118

hsa-miR-132 125.894736842105 103.982456140351 1.21073055508689 0.191223941950794 0.2594097965575 0.46309480511906 0.310405234090089 0.505433428339144 0.575420875420875

hsa-miR-767-3p 42.2456140350877 23.6491228070176 1.78635014836795 0.580174514956458 0.248028832487403 0.451579920752381 0.939101999477893 0.970592844969367 0.577104377104377

hsa-miR-1281 102.304824561404 123.763157894737 0.826617761712382 -0.190412889460087 0.430176045751706 0.615662427367514 0.316106279237542 0.510114427856287 0.447138047138047

hsa-miR-1914S 153.491228070175 117.684210526316 1.30426356589147 0.265638564163164 0.0436144624583965 0.138890336168251 0.126699408568715 0.281808220605156 0.634680134680135

hsa-miR-550 135.688596491228 98.5701754385965 1.37656847913144 0.319593793096437 0.00341031249374062 0.025816663878054 0.0510724027154038 0.155743758103864 0.695286195286195

hsa-miR-330-5p 8.10526315789475 28.2280701754386 0.287134866376632 -1.24780325591064 0.00692421059885416 0.0415838606219264 0.00765464269952029 0.0474155032020666 0.321885521885522

hsa-miR-616S 52.6315789473684 55.8070175438597 0.943099654196793 -0.0585833240988974 0.294523674995098 0.493541614603437 0.185084242886132 0.359747075699848 0.57003367003367

hsa-miR-765 52.9824561403509 60.0350877192982 0.882524839275278 -0.124968344083223 0.11423985722378 0.265907339166088 0.0212848377601841 0.092859049967948 0.394612794612795

hsa-miR-520g 59.7894736842105 45.7192982456141 1.30775134305449 0.268309130264415 0.365110065279262 0.565691178341119 0.717195366316709 0.820874802561432 0.560606060606061

hsa-miR-28-3p 146.741228070175 124.986842105263 1.17405340913078 0.160462213668206 0.0192054168552521 0.0808501207125977 0.00758322412312331 0.0474155032020666 0.656228956228956

hsa-miR-296-3p 89.7543859649123 97.2675438596491 0.922757812147721 -0.080388470949405 0.238629110312453 0.439097915137839 0.128609532544769 0.283137822923815 0.421212121212121

hsa-miR-1224-3p 144.600877192982 169.236842105263 0.854429067537448 -0.157321790360703 0.213489478568828 0.413097354271073 0.510627712277608 0.685337038406805 0.416835016835017

hsa-miR-590-3p 4.2280701754386 8.52631578947369 0.495884773662551 -0.701411690409839 0.277081524217338 0.479226747611105 0.0157706312127872 0.077771741352202 0.430639730639731

hsa-miR-455-3p 184.885964912281 114.561403508772 1.61385911179173 0.478628274706625 0.000201523355014761 0.00543451823935425 0.120500330347658 0.275459347690839 0.747811447811448

hsa-miR-485-5p 34.2105263157895 32.5263157894737 1.05177993527508 0.0504839054319925 0.367181711203448 0.566865504058274 0.621554710672611 0.767384428198087 0.56026936026936

hsa-let-7a 1091.65789473684 1135.39035087719 0.961482448651634 -0.0392789682523769 0.460211881598617 0.64162011925623 0.0896885267279464 0.229674686726875 0.549494949494949

hsa-miR-1253 80 83.671052631579 0.956125176914609 -0.0448664363071956 0.738546917576511 0.844209749049284 0.658715093067943 0.785562559043648 0.477441077441077

hsa-miR-490-3p 98.1491228070175 90.125 1.08903326276857 0.0852903878081126 0.314248501763663 0.519533442570959 0.970619581961767 0.985752953652734 0.567340067340067

hsa-miR-194S 46.8771929824562 83.3026315789474 0.562733638708998 -0.574948873435809 0.00114449188914989 0.0139112183145965 0.000724494622338028 0.0140799344960282 0.283164983164983

hsa-miR-1259 23.4385964912281 40.8947368421052 0.573144573144574 -0.556617284939165 0.335493656932891 0.540169824502024 0.679783442040469 0.798167497252959 0.436363636363636

hsa-miR-1249 82.7719298245614 144.131578947368 0.574280323778225 -0.554637632924264 0.0158446646410229 0.070885047507704 0.00575351297779787 0.0406990303265538 0.339057239057239

hsa-miR-651 1 20.280701754386 0.0493079584775087 -3.00966978139777 0.00205763305693909 0.0192239060939959 0.0197910848174454 0.0908495010502946 0.305723905723906

hsa-miR-182 3612.86403508772 3467.72368421053 1.0418546470522 0.0410024394092109 0.871345478700774 0.927305742519428 0.9307292289254 0.966569584311215 0.511111111111111

hsa-miR-1324 141.5 140.868421052632 1.00448346721465 0.00447344641629668 0.406905480301486 0.604405214286028 0.697695921047953 0.811792890822156 0.555555555555556

hsa-miR-15bS 78.7368421052631 74.578947368421 1.05575158786168 0.0542529188437424 0.765347351821487 0.864521943222439 0.825840697825331 0.890875652779076 0.52020202020202

hsa-miR-543 47.4736842105263 46.2105263157895 1.02733485193622 0.026967926427507 0.460089811495037 0.64162011925623 0.0999041962248618 0.246335203834445 0.450505050505051

hsa-miR-1287 64.7719298245614 73.719298245614 0.878629224178962 -0.129392285782203 0.507624504918697 0.679193717433854 0.356435149697453 0.549899991483046 0.455555555555556

hsa-miR-220c 135.688596491228 119.964912280702 1.131069026031 0.123163226245147 0.159749564761965 0.340438384402884 0.0318611230297487 0.116017507066131 0.593939393939394

hsa-miR-611 106.385964912281 96.1403508771929 1.10656934306569 0.101264547380147 0.700695131147602 0.822756905495784 0.205563889640747 0.381174441738053 0.474074074074074

hsa-miR-1825 39.9824561403509 58.8421052631579 0.67948717948718 -0.38641691313747 0.0356535116168096 0.120191330176979 0.0202944419316337 0.0922620643667372 0.35993265993266

hsa-miR-320b 2106.82894736842 1212.00438596491 1.73830142181467 0.55290844207902 0.000700334941504715 0.0102438822799758 0.155465611566785 0.320207214277174 0.725925925925926

hsa-miR-612 77.9298245614035 82.0175438596491 0.950160427807486 -0.0511244372684152 0.831791612957314 0.906365640348179 0.749102653712172 0.841765091345839 0.514478114478115

hsa-miR-671-5p 69.9649122807017 67.719298245614 1.03316062176166 0.0326226686228524 0.730805862047165 0.839944666114324 0.552849590698758 0.709983923769388 0.523232323232323

hsa-miR-145S 86.8421052631579 42.6666666666667 2.03536184210526 0.710673612476679 0.00394482611012665 0.0288507197715195 0.00475332893260513 0.0359835339371774 0.691582491582492

hsa-miR-657 61.859649122807 58.3333333333333 1.06045112781955 0.0586944098678609 0.700581565218737 0.822756905495784 0.974330555580281 0.985752953652734 0.525925925925926

hsa-miR-1826 217.482456140351 158.043859649123 1.37608924904257 0.319245598630553 5.68933989700764e-05 0.00287022034727864 5.54736083076352e-06 0.00193019760961628 0.768350168350168

hsa-miR-99a 114.701754385965 119.40350877193 0.960622979723773 -0.0401732677766952 0.979827295764173 0.995974060001018 0.930363245151927 0.966569584311215 0.497979797979798

hsa-miR-604 90.6754385964912 67.298245614035 1.34736704900939 0.298152354005211 0.0354016189130342 0.119821972701141 0.0488069312007955 0.152059139445078 0.64040404040404

hsa-miR-101S 102.5 122.078947368421 0.839620607889632 -0.174805146446895 0.472697407443249 0.653746574717186 0.273031913051847 0.469375579609052 0.451851851851852

hsa-miR-504 22.015350877193 24.0175438596491 0.91663623082542 -0.0870445802767585 0.2755363886273 0.479226747611105 0.133741251415553 0.291461363564702 0.427272727272727

hsa-miR-424 230.30701754386 246.535087719298 0.934175413627469 -0.0680910493642152 0.652646135470821 0.794405662780421 0.305040759799718 0.501428906108869 0.46969696969697

hsa-miR-767-5p 199.447368421053 149.728070175438 1.33206397562833 0.286729600715806 0.00567153042623241 0.0368009831416434 0.00769197039199227 0.0474155032020666 0.684511784511785

hsa-miR-222S 72.7061403508771 47.7543859649123 1.52250183688464 0.420354927093563 0.0184311619266598 0.0795304637135369 0.139992833917845 0.30055977564424 0.657239057239057

hsa-miR-875-5p 33.7543859649123 40.8947368421052 0.825396825396826 -0.191891007810105 0.235598169202593 0.436429395650133 0.0611908341356455 0.177546603942006 0.420875420875421

hsa-miR-876-3p 9.71929824561404 17.0175438596491 0.571134020618557 -0.560131384750145 0.0378600591405372 0.125184793250129 0.00982723175111379 0.0554307254981124 0.364646464646465

hsa-miR-361-3p 154.285087719298 157.131578947368 0.981884664768603 -0.0182814268445236 0.491609826354853 0.66603083806427 0.439731768637563 0.61805947285703 0.453872053872054

hsa-miR-371-5p 30.3333333333333 20.280701754386 1.49567474048443 0.402577436450968 0.148444434187137 0.322689034517631 0.205825364831903 0.381174441738053 0.595959595959596

hsa-miR-29b-2S 54.4385964912281 57.9649122807017 0.939164648910412 -0.0627644701924882 0.907395757866501 0.954978706144866 0.727907903232731 0.82740837707472 0.508080808080808

hsa-miR-556-3p 1 2.87719298245614 0.347560975609756 -1.05681515998965 0.00693867431003176 0.0415838606219264 0.0018215075724518 0.0222258115415021 0.339393939393939

hsa-miR-1263 80 74.578947368421 1.0726887791108 0.0701683741496419 0.951610053333894 0.983520330571438 0.778676185050063 0.863750061308746 0.504377104377104

hsa-miR-1224-5p 19.2280701754386 36.280701754386 0.529980657640232 -0.634914768120358 0.164279956463675 0.347484319676842 0.00686300574800235 0.0459129764381863 0.408417508417508

hsa-miR-346 70.1403508771929 59.438596491228 1.18004722550177 0.1655544592884 0.540570864904385 0.710064926046399 0.674666673038768 0.794321062527226 0.541077441077441

hsa-miR-1234 555.543859649123 529.405701754386 1.04937264145082 0.0481925012719817 0.412615429405962 0.606793383317771 0.256863697101402 0.446922118142156 0.445117845117845

hsa-miR-154S 48.6315789473684 57.9649122807017 0.838983050847458 -0.175564774331074 0.240459968055313 0.440095213150213 0.0752487871035401 0.201676097112904 0.421548821548822

hsa-let-7b 965.995614035088 587.213815789474 1.64504919342942 0.497770288590514 0.00136752612502954 0.0150361655662008 0.00127281070273024 0.0192708006395824 0.713468013468013

hsa-miR-26a 6386.57456140351 7079.20175438596 0.902160269333568 -0.102963092515132 0.00789945968814876 0.0456191904438564 0.00204265281590508 0.0231880468618216 0.323232323232323

hsa-miR-631 155.53399122807 153.754385964912 1.01157433820173 0.0115078679570504 0.995965046430728 0.998278554087942 0.596944250858559 0.745532400131601 0.499326599326599

hsa-miR-379 1 1 1 0 0.471479181480217 0.653746574717186 0.0918949315653856 0.230538738200371 0.456565656565657

hsa-miR-129-5p 39.2631578947368 44.421052631579 0.88388625592417 -0.123426894392197 0.609018870792546 0.758990383481437 0.142995413590117 0.305458024574928 0.465656565656566

hsa-miR-374bS 18 58.7017543859649 0.306634787806336 -1.18209785581605 0.000828878469301785 0.0113543193493244 0.000774082528306164 0.0145224613462656 0.278451178451178

hsa-miR-615-3p 19.4035087719298 41.8771929824561 0.463343108504399 -0.76928744425655 0.0158457092417654 0.070885047507704 0.0695080898638885 0.19226115882223 0.33973063973064

hsa-miR-200a 118.526315789474 84.5855263157895 1.40126001400016 0.337371841855168 0.000126363221979223 0.00463909388307507 7.637773526646e-05 0.00346915713341868 0.755555555555556

hsa-let-7aS 1 1 1 0 0.4120648922481 0.606793383317771 0.230772207289941 0.408107407563974 0.44983164983165

hsa-miR-635 133.280701754386 101.333333333333 1.31527008310249 0.27404203093056 0.0132125676528298 0.064058684743776 0.02624213284284 0.103702212210388 0.665319865319865

hsa-miR-1251 164.131578947368 129.394736842105 1.26845637583893 0.237800709114183 0.104502493389963 0.251213514750802 0.524739119606602 0.695977639599638 0.608417508417508

hsa-miR-1225-5p 41.0877192982456 74.5614035087719 0.551058823529412 -0.5959137177608 0.323588628231267 0.528400259759746 0.0795882537011737 0.209404460195466 0.434343434343434

hsa-miR-19b 8542.95614035088 9361.25657894737 0.912586474722125 -0.0914724311968146 0.0417915079577458 0.13417932451543 0.106966457680822 0.255889924548641 0.364646464646465

hsa-miR-1184 195.69298245614 168.885964912281 1.15872850984262 0.147323291743314 0.23667318327261 0.436429395650133 0.219775448089908 0.398458427944518 0.579124579124579

hsa-miR-30c-1S 44.9122807017544 35.4385964912281 1.26732673267327 0.236909747078359 0.323592296137529 0.528400259759746 0.416597236927372 0.600512421022054 0.565319865319865

hsa-miR-216b 190.197368421053 144.131578947368 1.31960927515063 0.27733568910678 0.181844926459851 0.369252168317298 0.819272405892091 0.886428852359239 0.589225589225589

hsa-miR-182S 53.4035087719298 39.6666666666667 1.34630694383016 0.297365246703607 0.111942165691667 0.262516546173664 0.558460158735621 0.715069694717997 0.606060606060606

hsa-miR-32S 29.280701754386 106.964912280702 0.273741184188945 -1.29557220224598 0.00184617054863372 0.0183131630284012 0.00372078900135979 0.0311751544482864 0.292929292929293

hsa-miR-376b 77.9298245614035 45.561403508772 1.7104351174432 0.536747792791623 0.351014488096578 0.55145696567286 0.667649773894268 0.790916342333396 0.562289562289562

hsa-let-7bS 1 2.98245614035088 0.335294117647059 -1.09274716921571 0.0791381003833025 0.20822006289875 0.00354738820524718 0.0306068996877364 0.391582491582492

hsa-miR-483-3p 11.8947368421053 31.7543859649123 0.374585635359116 -0.981934836319476 0.278072516692295 0.479953163810902 0.169883811044701 0.339374372526798 0.428956228956229

hsa-miR-574-3p 1292.87719298246 1533.93421052632 0.842850484792859 -0.170965697573143 0.261455698796833 0.46309480511906 0.311250190699693 0.505854829705903 0.424915824915825

hsa-miR-541S 1 1 1 0 0.761729969551568 0.862694178114178 0.0594578902222056 0.174531153951576 0.482154882154882

hsa-miR-1254 135.688596491228 141.09649122807 0.961672365557974 -0.0390814626687871 0.83179789936936 0.906365640348179 0.409755404238872 0.595318036798227 0.514478114478115

hsa-miR-302bS 15.7894736842105 20.280701754386 0.778546712802768 -0.250326285908012 0.46328051455708 0.644856587197999 0.374324854345564 0.565748422592332 0.451851851851852

hsa-miR-217 175.798245614035 132.947368421053 1.32231459487992 0.279383682014937 0.00524682293825707 0.0353750640290301 0.0222569656402286 0.0946195140271787 0.686195286195286

hsa-miR-1271 228.412280701754 208.837719298246 1.09373096713221 0.0895947570591541 0.581486029280947 0.741244377059759 0.440518173276711 0.618158022012686 0.537037037037037

hsa-miR-503 303.464912280702 249.100877192982 1.21824104234528 0.197408049827335 0.193705273321957 0.387859978832596 0.517958675339783 0.691947889811506 0.586868686868687

hsa-miR-770-5p 49.0526315789474 64.2807017543859 0.763100436681223 -0.270365622440319 0.105538832480868 0.251602244284499 0.0683631044668753 0.190314061790043 0.391919191919192

hsa-miR-320c 538.96052631579 302.114035087719 1.78396388025899 0.578837787461259 0.000617867880162956 0.0100607543505779 0.187804764773662 0.361775696427836 0.728282828282828

hsa-miR-621 387.254385964912 266.921052631579 1.45081994150317 0.372128873509408 0.0345316313915603 0.119203191563666 0.191140315730394 0.364942682467543 0.641077441077441

hsa-miR-411 58.3333333333333 20.5614035087719 2.83703071672355 1.042757982953 2.41808400941798e-05 0.00236979110557927 0.0104813619256623 0.0583575183344939 0.780471380471381

hsa-miR-432S 44.421052631579 52.3508771929824 0.848525469168901 -0.164255178059584 0.256459879303039 0.46261044860492 0.105691439162981 0.254781318429197 0.424242424242424

hsa-miR-1258 1 1 1 0 0.00550068063604153 0.0359627832492715 0.0169942882945073 0.0810280154594463 0.342760942760943

hsa-miR-29a 594.078947368421 494.798245614035 1.20064885563848 0.182862123693408 0.0678623469865874 0.18372179247431 0.115880237133135 0.269554298236915 0.621885521885522

hsa-miR-548a-5p 1 17.0175438596491 0.0587628865979381 -2.83424480366288 0.178137112075121 0.366029351716261 0.227572114982902 0.40577424634348 0.413468013468013

hsa-miR-135a 1 1 1 0 3.68926025344906e-05 0.00236979110557927 5.36216154404919e-05 0.00308503027500963 0.275084175084175

hsa-miR-196b 1 3.08771929824561 0.323863636363636 -1.1274327272036 3.7474464037132e-05 0.00236979110557927 3.47854604832543e-05 0.00308503027500963 0.266666666666667

hsa-miR-769-5p 1 1 1 0 0.367155137272694 0.566865504058274 0.637871589495185 0.773496760674912 0.446127946127946

hsa-miR-1243 75.1754385964912 37.7543859649123 1.99117100371747 0.688722909750205 0.0213082314847759 0.0871516766415242 0.0390892411480234 0.131130011705052 0.653198653198653

hsa-miR-103-as 179.236842105263 162.061403508772 1.10598105548038 0.100732774093025 0.547319386584096 0.713611053315229 0.807941556406935 0.877626399805817 0.54040404040404

hsa-miR-141 50.6754385964912 41.8771929824561 1.21009635525765 0.19069998888249 0.372792940001538 0.573476483460477 0.884216832057786 0.935145987825821 0.55959595959596

hsa-miR-193b 74.578947368421 84.7456140350878 0.880033122865127 -0.127795732598775 0.302238205443343 0.503535851925879 0.306425954293782 0.502748286227251 0.430976430976431

hsa-miR-548c-3p 42.2456140350877 45.2631578947369 0.933333333333333 -0.0689928714869522 0.803853386150829 0.891677984894814 0.726296818395153 0.82740837707472 0.516835016835017

hsa-miR-520c-3p 1 1 1 0 0.00055044052377819 0.00950060344041157 0.0718098862190765 0.194880288701456 0.309090909090909

hsa-miR-1180 51.280701754386 59.5438596491228 0.86122569239835 -0.149398680682925 0.634277333244632 0.781973340843025 0.905409333419345 0.948262445073901 0.468013468013468

hsa-miR-526a 167.684210526316 100.912280701754 1.66168289290682 0.507830879737221 0.0206998146102699 0.0856385558862379 0.100326488439071 0.246671679552473 0.654208754208754

hsa-miR-383 67.4035087719298 67.8070175438596 0.99404915912031 -0.00596861769302052 0.753030834286511 0.856893771833878 0.220814429743939 0.399351956861447 0.478787878787879

hsa-miR-1284 15.7894736842105 42.1754385964912 0.374376039933444 -0.982494532330788 0.000405778568743468 0.00778193121834696 0.00749324941826945 0.0472020018099747 0.265656565656566

hsa-miR-197 449.135964912281 462.921052631579 0.97022151478976 -0.0302308677935611 0.843639675092668 0.910087880834301 0.350022268400765 0.545255166363779 0.513468013468013

hsa-miR-614 40.3333333333333 37.7543859649123 1.06830855018587 0.0660766034815065 0.870895689709097 0.927305742519428 0.1917710803088 0.365184540761267 0.488888888888889

hsa-miR-518a-5p 180.635964912281 169.236842105263 1.06735603586793 0.0651845960124309 0.29286963014168 0.491724690296245 0.27033029923233 0.465658778917167 0.57037037037037

hsa-miR-186S 103.59649122807 125.649122807018 0.824490365819603 -0.192989821926916 0.567694164790755 0.731223976439435 0.185965178672948 0.360647076842145 0.461616161616162

hsa-miR-887 116.723684210526 111.622807017544 1.0456974459725 0.0446840752100277 0.135741005167201 0.301142641283532 0.192113304178001 0.365184540761267 0.5996632996633

hsa-miR-508-5p 133.280701754386 111.622807017544 1.19402750491159 0.177332050644444 0.09415947371331 0.233504671880996 0.33862124195453 0.533355904582843 0.611784511784512

hsa-miR-499-3p 112.982456140351 119.964912280702 0.941795846738813 -0.0599667511008471 0.202364031804256 0.398721825221628 0.0456322121607844 0.145854070721322 0.414814814814815

hsa-miR-508-3p 1 32.2631578947368 0.0309951060358891 -3.47392595676977 0.000191508817044655 0.00543451823935425 4.23439500632495e-05 0.00308503027500963 0.273063973063973

hsa-miR-124 125.649122807018 107.508771929825 1.16873368146214 0.155920839137951 0.232663786456088 0.435550645795237 0.73615126003301 0.831542588231005 0.57979797979798

hsa-miR-1290 1 1 1 0 0.0472761029057141 0.142515076869079 0.0259532757381543 0.103702212210388 0.39023569023569

hsa-miR-592 57.8245614035088 51.8947368421053 1.11426639621366 0.108196247758617 0.229378246903467 0.430333537125417 0.919185803351705 0.959198728285999 0.58013468013468

hsa-miR-1307 88.7368421052632 77.1929824561403 1.14954545454546 0.139366607323558 0.711996089712822 0.828103268763026 0.756750717029951 0.847050413484887 0.524915824915825

hsa-miR-1911S 100.701754385965 79.859649122807 1.2609841827768 0.231892513507446 0.326565099298838 0.531746567348862 0.64356943207829 0.777871736531602 0.565656565656566

hsa-miR-489 249.100877192982 172.923245614035 1.44052857849416 0.365010114633509 0.0208304014796235 0.0856385558862379 0.312863334291738 0.507520784762726 0.654208754208754

hsa-miR-302e 1 15.9649122807018 0.0626373626373626 -2.77039333167635 0.00457716028055882 0.032377781328871 0.00877650491367955 0.0520988262216422 0.317508417508418

hsa-miR-1278 70.1403508771929 45.561403508772 1.53946861763573 0.431437303397596 0.00658706524590823 0.0403165766469419 0.0260735589098596 0.103702212210388 0.681144781144781

hsa-miR-1267 29.8245614035088 44.421052631579 0.671406003159558 -0.398381253219759 0.227102744588022 0.428861419211079 0.144012501721194 0.305513901229934 0.41952861952862

hsa-miR-570 95.719298245614 80.8771929824561 1.18351409978308 0.168488063532603 0.179878632682618 0.367559933947718 0.523927180810077 0.695977639599638 0.58956228956229

hsa-miR-1247 56.9824561403509 69.6842105263157 0.817724068479356 -0.201230323883494 0.415535637328059 0.606793383317771 0.420819473335248 0.602267339118274 0.445454545454545

hsa-miR-432 1 11.4561403508772 0.0872894333843798 -2.43852586144188 0.00784347381849186 0.0456191904438564 0.00463935518524134 0.0359835339371774 0.337037037037037

hsa-miR-375 1 4.35087719298246 0.229838709677419 -1.47037747833043 0.289346835004837 0.490582158367729 0.159552841332426 0.324750240730857 0.433670033670034

hsa-miR-758 47.0175438596491 56.3508771929825 0.834371108343711 -0.18107700156175 0.141091564061244 0.311411815306531 0.0713750969245954 0.194635098613405 0.402020202020202

hsa-miR-518e 84.3552631578948 77.1929824561403 1.09278409090909 0.0887286516291247 0.983860707359733 0.995974060001018 0.274804849282671 0.470548779624891 0.498316498316498

hsa-miR-620 4.70175438596492 14.8070175438597 0.317535545023697 -1.1471655140851 0.374556568120625 0.574599737910479 0.18210606383806 0.357176211573285 0.442424242424242

hsa-miR-17S 708.070175438597 498.324561403509 1.42090161770142 0.35129161216601 0.0186732188442319 0.0801740689680206 0.0755073354157613 0.201742509175857 0.656902356902357

hsa-miR-100 116.5 156.28947368421 0.745411685468934 -0.293818615283719 0.439093811943425 0.62219517257345 0.16755416842614 0.335497093623571 0.448148148148148

hsa-miR-495 97.7236842105263 84.0921052631579 1.16210295728368 0.150231258019014 0.491612565291935 0.66603083806427 0.742041948682427 0.836008096230985 0.546127946127946

hsa-miR-196aS 171.657894736842 129.587719298246 1.32464631422189 0.281145491142597 0.103446165492105 0.24936882910527 0.510276300254972 0.685337038406805 0.608754208754209

hsa-miR-877 75.1754385964912 83.671052631579 0.898464119096293 -0.107068507783747 0.198879071173436 0.392948361562866 0.0517341072594491 0.157206107622903 0.414141414141414

hsa-miR-509-3p 1 16.8245614035088 0.059436913451512 -2.82283980704889 0.0217623108614216 0.0881731186544922 0.00300534962758342 0.0285012827319175 0.36026936026936

hsa-miR-875-3p 46.5263157894737 43.2478070175439 1.07580751483191 0.0730715561755201 0.795773128789118 0.886131884058076 0.577142911716245 0.733540990885301 0.517508417508417

hsa-miR-1269 46.3684210526316 45.2631578947369 1.02441860465116 0.0241252366886258 0.61279603537637 0.762021582895976 0.276447477042783 0.472424104332518 0.465993265993266

hsa-miR-500S 259.482456140351 209.482456140351 1.23868347221641 0.214049099642796 0.00500600373484923 0.0345614497853991 0.0204424255405893 0.0922620643667372 0.687205387205387

hsa-miR-144S 716.530701754386 587.213815789474 1.22022112301812 0.19903209069717 0.0156137030836023 0.070885047507704 0.018751315563078 0.0874723531401961 0.661279461279461

hsa-miR-181c 161.657894736842 83.671052631579 1.93206479006133 0.658589270456464 0.000678066985209866 0.0100891691075192 0.000286834744834819 0.0083136235250579 0.726599326599327

hsa-miR-378S 40.2807017543859 46.9473684210526 0.857997010463378 -0.153154663808663 0.609254779876078 0.758990383481437 0.394511484100789 0.583809996845114 0.465656565656566

hsa-miR-1470 184.570175438596 173.675438596491 1.06273044093136 0.0608414838826134 0.788684867588145 0.88051104880798 0.513856225000119 0.688599258035874 0.518181818181818

hsa-miR-449a 1 1 1 0 0.109175625547057 0.258133054375645 0.101153402386506 0.247295711783441 0.405387205387205

hsa-miR-1255b 86.6666666666667 70.5263157894737 1.22885572139304 0.206083428568902 0.520407456683158 0.692398625516679 0.536120511024294 0.704930551107513 0.543097643097643

hsa-miR-450a 1 32.8245614035088 0.0304649919828969 -3.49117705843954 0.00112248771461121 0.0138387558906012 0.000734179666652685 0.0140799344960282 0.291919191919192

hsa-miR-324-3p 587.213815789474 491.008771929825 1.19593345243412 0.178927012202889 0.0137712807546927 0.0649432529579223 0.0256623768771122 0.103702212210388 0.664309764309764

hsa-miR-208b 88.1578947368421 45.4035087719299 1.94165378670788 0.663540077306485 0.000727329966405922 0.010289930508333 0.00145241069536308 0.0195848504702865 0.724915824915825

hsa-miR-93S 760.899122807018 1030.54385964912 0.738347151052927 -0.303341170601759 0.173657399385393 0.359391692253223 0.954318315749074 0.97901699343718 0.409090909090909

hsa-miR-542-5p 112.359649122807 74.5964912280701 1.50623236124177 0.409611407809353 0.0231649412091723 0.092566421411138 0.127463764201385 0.282054432066141 0.651515151515152

hsa-miR-144 2637.20614035088 2637.20614035088 1 0 0.987887277909742 0.995974060001018 0.93275835415389 0.967512571676451 0.501346801346801

hsa-miR-593S 315.552631578947 278.521929824561 1.13295434861345 0.124828688748184 0.591920887805192 0.745733906826103 0.336625675500365 0.532065857063763 0.536026936026936

hsa-miR-520c-5p 134.754385964912 125.298245614035 1.07546905628675 0.0727568978442882 0.341718994254777 0.545122727659905 0.659945371154861 0.785562559043648 0.563636363636364

hsa-miR-143S 133.280701754386 105.017543859649 1.26912796525226 0.238330023090577 0.0585840028115516 0.165370978285339 0.764006764958821 0.854064557201376 0.626262626262626

hsa-miR-212 55.8947368421053 18.4912280701754 3.02277039848197 1.10617376136869 0.000607046156903234 0.0100607543505779 0.0447755772362192 0.144184041622601 0.727946127946128

hsa-miR-16-1S 95.719298245614 101.631578947368 0.941826342137062 -0.0599343715733264 0.674672888398546 0.809799790900936 0.502701195251313 0.68105358163561 0.471717171717172

hsa-miR-338-5p 35.6820175438596 58.3333333333333 0.611691729323308 -0.491526833630472 0.0990565824563766 0.242169491954258 0.0332286315059934 0.118009502015112 0.38989898989899

hsa-miR-885-5p 15.6315789473684 67.4736842105263 0.231669266770671 -1.46244449867792 0.000150772646146647 0.00500449206248294 3.1746108427584e-05 0.00308503027500963 0.249158249158249

hsa-miR-127-3p 68.2105263157894 40.5263157894737 1.68311688311688 0.52064736206449 0.0891119961502231 0.225915928143319 0.353922233296217 0.54812746291154 0.613131313131313

hsa-miR-1303 29.280701754386 58.9824561403509 0.496430696014277 -0.700311390306725 0.0021402013841466 0.0192617247604826 0.000644480626268724 0.013311369232367 0.295622895622896

hsa-miR-548m 1 1 1 0 0.194904299968577 0.389357432576116 0.223345782982073 0.401557105653186 0.425589225589226

hsa-miR-650 140.640350877193 137.675438596491 1.02153552086652 0.021306907912783 0.685799674735994 0.81409232365497 0.155983483443837 0.320508919552456 0.472727272727273

hsa-miR-33aS 89.7543859649123 82.7719298245614 1.0843577787198 0.0809879027311127 0.663638180644291 0.802128501254934 0.600423691477897 0.747713774524423 0.529292929292929

hsa-miR-575 140.377192982456 129.201754385965 1.0864960282436 0.0829578651564467 0.537242713815888 0.706796080019112 0.77569610178549 0.86214008735538 0.541414141414141

hsa-miR-607 110.530701754386 83.671052631579 1.3210148346176 0.278400255316348 0.028173745435905 0.105738952420311 0.0168271993980998 0.0806770726697786 0.646464646464647

hsa-miR-548j 1 1.3859649122807 0.721518987341775 -0.326396584632467 0.00542786550098728 0.035757617766046 0.00199944042281154 0.0230068944651514 0.336026936026936

hsa-miR-509-3-5p 283.236842105263 201.236842105263 1.40748005753891 0.341800912220074 0.12669895658894 0.286233506639412 0.346912352859526 0.543349111647497 0.602020202020202

hsa-miR-1204 1 1 1 0 0.252452424404941 0.457702609792992 0.221193783754081 0.399351956861447 0.430639730639731

hsa-miR-1237 91.4035087719298 101.333333333333 0.902008310249307 -0.10313154582525 0.738561252065132 0.844209749049284 0.633251523632834 0.771861211803507 0.477441077441077

hsa-miR-27aS 43.6140350877193 42.0350877192982 1.03756260434057 0.0368743128353167 0.765252175961493 0.864521943222439 0.653251920609119 0.785141755738159 0.47979797979798

hsa-miR-363S 67.1929824561403 42.0350877192982 1.59849749582638 0.469064122939351 0.0871621805399825 0.222313574139708 0.189430284166324 0.362479679014496 0.614141414141414

hsa-miR-1294 1 17.0175438596491 0.0587628865979381 -2.83424480366288 0.179291344902668 0.367525963541575 0.162372830971779 0.328938387625928 0.415151515151515

hsa-miR-935 32.7368421052632 12.7478070175439 2.56803715809393 0.943141855385775 0.0697512863367352 0.188110500339383 0.119442287092915 0.274145462130813 0.619528619528619

hsa-miR-1202 231.236842105263 237.026315789474 0.975574553125347 -0.0247286962878995 0.761564416256156 0.862694178114178 0.22915719515355 0.406919052299411 0.479461279461279

hsa-miR-876-5p 35.6820175438596 21.0657894736842 1.69383718509265 0.526996478914548 0.277096346532956 0.479226747611105 0.920465789290409 0.959374367340124 0.572390572390572

hsa-miR-588 139.412280701754 125.649122807018 1.10953644233454 0.103942308578496 0.246849320937348 0.450382587672159 0.284298085174965 0.480643389728918 0.577441077441077

hsa-miR-1181 185.69298245614 211.342105263158 0.878636948491263 -0.129383494498992 0.987895016267233 0.995974060001018 0.376641987675129 0.566890528221108 0.501346801346801

hsa-miR-412 53.2982456140351 46.9473684210526 1.13527653213752 0.126876261907599 0.331515968735472 0.536853925673786 0.354151717021962 0.54812746291154 0.564983164983165

hsa-miR-22S 52.4385964912281 58.8421052631579 0.891174716756112 -0.115214780099216 0.859260501951476 0.920293216746039 0.701846657023504 0.814104388456026 0.487878787878788

hsa-miR-381 107.157894736842 96.719298245614 1.10792671866497 0.10249044774884 0.478958548944375 0.656097186887295 0.797814845399004 0.872641586285603 0.547474747474747

hsa-miR-18a 1470.35964912281 840 1.75042815371763 0.559860417278441 0.000811601796674906 0.0112969733956523 0.00143591409109801 0.0195848504702865 0.723232323232323

hsa-miR-634 144.600877192982 109.074561403509 1.32570670312437 0.281945678100425 0.0921695226613582 0.230557385671745 0.628776674432341 0.77046594405452 0.612457912457912

hsa-miR-152 213.377192982456 191.517543859649 1.11413914716255 0.108082041391089 0.346906763289594 0.549143119480075 0.62752196777384 0.77046594405452 0.562962962962963

hsa-miR-92bS 125.40350877193 136.513157894737 0.918618473895582 -0.0848843963677993 0.46648715276149 0.648274416800589 0.234949833225875 0.414645615693109 0.451178451178451

hsa-miR-891b 135.087719298246 81.0350877192982 1.66702749512882 0.51104209741117 0.00118710231557357 0.014228740254722 0.00256030166587074 0.0249896897703841 0.716161616161616

hsa-miR-501-3p 304.815789473684 238.921052631579 1.27580129970261 0.243574451560589 0.0336698975398383 0.117165812810002 0.376244915029228 0.566890528221108 0.641750841750842

hsa-miR-1295 100.175438596491 87.9122807017544 1.13949311514668 0.130583527690331 0.15085406831288 0.327103168226169 0.213165955009366 0.390577960027776 0.595959595959596

hsa-miR-363 3612.86403508772 3312.48684210526 1.09068026751513 0.0868016002102231 0.975759695123346 0.995974060001018 0.878958860547371 0.931869160506611 0.497643097643098

hsa-miR-1236 24.8421052631579 30.2456140350877 0.821345707656613 -0.196811176969973 0.374854753700579 0.574599737910479 0.438928323476948 0.617936612007514 0.441077441077441

hsa-miR-103 8542.95614035088 7079.20175438596 1.20676828218069 0.187945945375913 0.280510064002779 0.482231444690037 0.251161837416463 0.438770578320662 0.572053872053872

hsa-miR-643 69.8947368421052 52.5263157894737 1.33066132264529 0.285676053724915 0.297275541258809 0.496649345140177 0.659217453388263 0.785562559043648 0.56969696969697

hsa-miR-609 1 1 1 0 0.0854109681857503 0.218742293794467 0.0360090245079244 0.124303152601355 0.4003367003367

hsa-miR-1264 18 23.7192982456141 0.75887573964497 -0.275917230872195 0.435202870190585 0.620006778499369 0.0563516149577782 0.16827489172513 0.448148148148148

hsa-miR-138 39.0350877192982 57.4736842105263 0.679181929181929 -0.38686625037262 0.080065665157893 0.209660763966557 0.0371868150738068 0.126506219947438 0.383164983164983

hsa-miR-877S 106.631578947368 105.684210526316 1.00896414342629 0.00892420399700887 0.847533419291008 0.913135256988939 0.985473465339551 0.990062398821924 0.486868686868687

hsa-miR-646 287.973684210526 214.271929824561 1.3439636467843 0.295623193211551 0.159765406353613 0.340438384402884 0.334504808909506 0.530657445016368 0.593939393939394

hsa-miR-585 10 1 10 2.30258509299405 0.799434575232509 0.8879176813715 0.485821954221536 0.665498962687596 0.516161616161616

hsa-miR-106aS 158.956140350877 142.69298245614 1.11397307432225 0.107932970945705 0.0892668962883798 0.225915928143319 0.461961255692868 0.641984804610217 0.613468013468013

hsa-miR-27bS 70.1403508771929 29.1578947368421 2.40553549939831 0.877772539644951 0.000671855005872136 0.0100891691075192 0.0445536006217718 0.144006581784978 0.726599326599327

hsa-miR-106bS 192.921052631579 161.657894736842 1.19339085137555 0.176798710059659 0.0390899392724978 0.128268507955002 0.955169020435499 0.97901699343718 0.637710437710438

hsa-miR-220b 27.140350877193 57.9649122807017 0.468220338983051 -0.758816284067802 0.0374570978532555 0.12480878551104 0.0505266820343874 0.155176251230165 0.361616161616162

hsa-miR-569 6.87719298245615 3.94736842105263 1.74222222222222 0.555161437586044 0.957330969837828 0.987068849426578 0.728201639004105 0.82740837707472 0.503703703703704

hsa-miR-302d 1 12.0701754385965 0.0828488372093023 -2.49073757009879 0.000227211969826754 0.00544677583223579 4.61529871969957e-05 0.00308503027500963 0.280808080808081

hsa-let-7g 338.219298245614 433.879385964912 0.779523778234917 -0.24907208647885 0.987895092810501 0.995974060001018 0.360388777760643 0.551445948949353 0.498653198653199

hsa-miR-559 59.3333333333333 51.1578947368421 1.15980795610425 0.148254436329977 0.62010980567868 0.768900520546983 0.977974130765628 0.988280649708123 0.533333333333333

hsa-miR-206 1 28.0701754385965 0.035625 -3.33470764039332 0.000610746455107551 0.0100607543505779 0.00162548392761665 0.0212544337808055 0.281144781144781

hsa-miR-30eS 53.2982456140351 69.8245614035087 0.763316582914573 -0.270082415122765 0.226699935471166 0.428861419211079 0.0912167032465794 0.230538738200371 0.419191919191919

hsa-miR-193a-5p 78.3508771929824 59.438596491228 1.31818181818182 0.276253376628158 0.417966838901144 0.608272144977549 0.788588039892175 0.868232348409909 0.554208754208754

hsa-miR-602 140.868421052632 115.087719298246 1.22400914634146 0.20213165656341 0.0572113292561612 0.163278051234304 0.165903144286762 0.333739891653789 0.626936026936027

hsa-miR-487a 75.7214912280701 71.0701754385965 1.06544680325845 0.0633942447577975 0.472680609702801 0.653746574717186 0.974320433579295 0.985752953652734 0.548148148148148

hsa-miR-92b 248.256578947368 225.798245614035 1.09946194786527 0.0948209218328635 0.195450597698327 0.389547034211677 0.0899187080055245 0.229674686726875 0.586531986531987

hsa-miR-142-5p 651.495614035088 693.083333333333 0.939996076520507 -0.061879577641153 0.584816962838383 0.743481744145741 0.32974568310932 0.526008363259415 0.463299663299663

hsa-miR-191 9071.26754385965 11503.9078947368 0.78853791484282 -0.237574788962955 0.0573270562271078 0.163278051234304 0.0645055378542267 0.182928268182897 0.373737373737374

hsa-miR-1207-3p 32.7017543859649 24.0175438596491 1.36157779401023 0.308644169957087 0.427785105959213 0.615306068533998 0.584795354933614 0.737416400409028 0.552861952861953

hsa-miR-125b-1S 1 14.0701754385965 0.071072319201995 -2.64405734003196 0.36305403326479 0.563517321416392 0.304554408075228 0.501428906108869 0.442424242424242

hsa-miR-188-5p 134.421052631579 127.828947368421 1.0515697375193 0.0502840359008345 0.430178961416699 0.615662427367514 0.791444946246192 0.868232348409909 0.552861952861953

hsa-miR-1260 1907.68201754386 2523.52192982456 0.755960150374633 -0.27976661533655 0.0190882375074039 0.0808501207125977 0.0615138291757355 0.177546603942006 0.343771043771044

hsa-miR-581 7.61403508771931 20.859649122807 0.365012615643398 -1.00782336259038 0.0130729616177897 0.064058684743776 0.000398619956150394 0.00955580617104973 0.338720538720539

hsa-miR-130aS 96.1403508771929 99.859649122807 0.962754743499648 -0.0379565792676821 0.501166020084163 0.673685787122481 0.27708232669374 0.472573217266201 0.454882154882155

hsa-miR-132S 1 8.10526315789475 0.123376623376623 -2.09251362324719 1.60054878833493e-06 0.000460424534777681 0.000163153303756051 0.00563205204565887 0.231649831649832

hsa-miR-339-5p 599.291666666667 443.978070175439 1.34982267576832 0.299973232540279 0.0441333570931748 0.139033502836251 0.0437110684478471 0.1427521468654 0.634343434343434

hsa-miR-129S 51.9649122807017 57.1578947368421 0.90914671577655 -0.0952487943342465 0.408926600072972 0.606363669867655 0.316235479609633 0.510114427856287 0.444781144781145

hsa-miR-320d 559.473684210526 296.605263157895 1.8862567651495 0.63459431765386 0.00162888587226766 0.0166094241316634 0.0364989218335631 0.125492308933725 0.71010101010101

hsa-miR-1322 127.828947368421 125.649122807018 1.01734850600391 0.0171997388013816 0.777002634511569 0.871980849913503 0.97192484309734 0.985752953652734 0.480808080808081

hsa-miR-548l 1 1 1 0 0.0709871226300601 0.190846999469601 0.0874232204468994 0.225886943849324 0.407407407407407

hsa-miR-886-5p 86.8421052631579 67.719298245614 1.28238341968912 0.24872039310386 0.415544483824801 0.606793383317771 0.630608263656581 0.770842679228937 0.554545454545455

hsa-miR-98 14.1754385964912 77.7894736842105 0.182228236355435 -1.70249533165516 0.01396038256972 0.0654772291177628 0.012439239449725 0.0649397135556259 0.338383838383838

hsa-miR-15a 2637.20614035088 3612.86403508772 0.729948903346108 -0.314780742705784 0.0127808278143795 0.06302773945034 0.118899100054312 0.273626462258324 0.334006734006734

hsa-miR-597 85.6052631578948 88.9122807017544 0.962805840568272 -0.0379035068595135 0.415538586203884 0.606793383317771 0.188694025326438 0.361873208570481 0.445454545454545

hsa-miR-26a-2S 1 1 1 0 0.000382913792972309 0.00756426665967551 0.000318142313183294 0.00857990050866195 0.309090909090909

hsa-miR-184 4.70175438596492 46.2105263157895 0.101746393318147 -2.28527190179237 0.000676395423611306 0.0100891691075192 0.000135566465918563 0.00531790273125998 0.277104377104377

hsa-miR-186 23.8070175438597 35.3508771929824 0.673449131513648 -0.395342814545913 0.26186589211831 0.46309480511906 0.11120863141771 0.263221338683826 0.425589225589226

hsa-miR-518d-5p 179.622807017544 110.530701754386 1.62509424229197 0.485565809356639 0.0222700260061517 0.0898085628192005 0.0163006943416191 0.0790308944765017 0.652525252525253

hsa-miR-28-5p 315.552631578947 342.780701754386 0.920567085497864 -0.0827654015535384 0.276894706308999 0.479226747611105 0.147211553652244 0.310119789536759 0.427272727272727

hsa-miR-15b 12702.7105263158 14207.2368421053 0.894101412363973 -0.111936073593334 0.226909913954912 0.428861419211079 0.984522962640238 0.990062398821924 0.41986531986532

hsa-miR-874 171.973684210526 182.214912280702 0.943795883981225 -0.0578453608127045 0.259445947138652 0.46309480511906 0.210041395118876 0.387319923050407 0.424579124579125

hsa-miR-130a 1292.87719298246 982.480263157895 1.31593197488455 0.274545140749894 0.0149375327418763 0.0689363142044881 0.0306510009886708 0.113041939543687 0.662289562289562

hsa-miR-421 112.982456140351 168.885964912281 0.668986651430946 -0.401991172070991 0.0775823383548186 0.205379012270578 0.35002475337837 0.545255166363779 0.382154882154882

hsa-miR-18bS 84.9912280701755 83.3026315789474 1.02027062602011 0.0200679117327134 0.574572977771224 0.7378816663937 0.163812512169529 0.330304200939961 0.462289562289562

hsa-miR-34bS 46.5964912280702 42.4561403508772 1.09752066115703 0.0930536914455929 0.560847416488134 0.724567844953981 0.111743594225635 0.263482846493778 0.460942760942761

hsa-miR-219-2-3p 1 8.52631578947369 0.117283950617284 -2.14315735606594 0.0481348846307287 0.143413378838107 0.0385412251682054 0.12992608328188 0.378787878787879

hsa-miR-1250 33.7543859649123 106.157894736842 0.317963972897042 -1.14581719539667 0.000726852816582937 0.010289930508333 0.00149140860540197 0.0198013173301831 0.274747474747475

hsa-miR-194 5199.04385964912 4503.74342105263 1.15438278196452 0.143565813228139 0.0415441058973589 0.13417932451543 0.0617733723111764 0.177701401015151 0.635690235690236

hsa-miR-583 53.4035087719298 45.8771929824562 1.16405353728489 0.151908342482553 0.696954372748591 0.821713151180781 0.543884612633734 0.706886175757399 0.526262626262626

hsa-miR-568 93.5087719298245 68.4561403508772 1.36596617119426 0.311861995976834 0.0135740630844233 0.0647205328279408 0.0116151125307633 0.0618755686052393 0.664646464646465

hsa-miR-633 96.1403508771929 76.6315789473684 1.25457875457875 0.22679986251732 0.153850401133807 0.331104479248067 0.151098953717827 0.313684509358367 0.595286195286195

hsa-miR-409-3p 36.280701754386 69.8245614035087 0.519597989949749 -0.654699862650163 0.0801715551668178 0.209660763966557 0.140005828283875 0.30055977564424 0.383501683501684

hsa-miR-26bS 1 12.0701754385965 0.0828488372093023 -2.49073757009879 0.0297214698017367 0.110084242227033 0.00564305617272443 0.0405829789755099 0.361616161616162

hsa-miR-924 19.4035087719298 20.5614035087719 0.943686006825938 -0.0579617880546782 0.411564757298416 0.606793383317771 0.147044808375015 0.310119789536759 0.445791245791246

hsa-miR-610 40.5263157894737 32.5964912280702 1.24327233584499 0.217746884142055 0.637887595322615 0.784186370140809 0.887258752041722 0.936068830088027 0.531649831649832

hsa-miR-448 110.530701754386 118 0.936700862325305 -0.0653912981682397 0.537263300686602 0.706796080019112 0.40053123195661 0.587594386217373 0.458585858585859

hsa-miR-517a 31.8771929824561 45.2631578947369 0.704263565891472 -0.350602609519493 0.0535812064479634 0.15517852252326 0.0233786509762655 0.0979406591869764 0.371380471380471

hsa-miR-125a-3p 18.6315789473684 36.3859649122807 0.512054001928641 -0.669325186987588 0.0498654697738678 0.147376371283726 0.0132765843805278 0.0682005495261638 0.37003367003367

hsa-miR-100S 77.6491228070175 30.2456140350877 2.56728538283063 0.942849069387356 0.0118427505510287 0.0601193748561043 0.0345951043897952 0.12087277363722 0.667676767676768

hsa-miR-199b-3p 124.986842105263 121.90350877193 1.02529322875441 0.0249786485117651 0.727141785522293 0.837860912761269 0.349404699227644 0.545255166363779 0.476430976430976

hsa-miR-10aS 42.4561403508772 38 1.11726685133887 0.110885391589796 0.831499567451712 0.906365640348179 0.772385467110295 0.861199816687577 0.514478114478115

hsa-miR-30dS 66.8070175438596 62.1951754385965 1.07415112302105 0.0715306966357913 0.887373879325187 0.938484874825535 0.535880387601728 0.704930551107513 0.49023569023569

hsa-miR-518c 91.719298245614 87.9122807017544 1.04330472959489 0.0423932997843876 0.979827805865989 0.995974060001018 0.52003905798507 0.69365333391208 0.502020202020202

hsa-miR-377 156.080043859649 154.741228070175 1.00865196564723 0.00861475188652092 0.485248064450414 0.660519053029506 0.790170358476119 0.868232348409909 0.546801346801347

hsa-miR-373S 23.4385964912281 20.280701754386 1.15570934256055 0.144714304864269 0.824636213689014 0.904270714629757 0.184006565346049 0.358459742423566 0.514814814814815

hsa-miR-513b 51.280701754386 25.859649122807 1.98303934871099 0.684630692361963 0.032937873112312 0.116899725455251 0.792777625199315 0.868232348409909 0.642087542087542

hsa-miR-1827 42.0350877192982 29.1578947368421 1.44163658243081 0.365778983819946 0.0234049375235269 0.0930804658193719 0.016052606583303 0.078712497053355 0.65016835016835

hsa-miR-199a-3p 118.526315789474 139.09649122807 0.852115784826891 -0.160032863701931 0.424287851131411 0.613334029357467 0.395069569128096 0.583809996845114 0.446464646464646

hsa-miR-576-3p 1 5.40350877192984 0.185064935064935 -1.68704851513903 0.00141127394074422 0.0150361655662008 0.0204657601927723 0.0922620643667372 0.305723905723906

hsa-miR-302c 8.73684210526317 25.2982456140351 0.345353675450763 -1.06318624082024 0.00209969683544572 0.0192617247604826 0.147333851344231 0.310119789536759 0.2996632996633

hsa-miR-302b 1 4.70175438596492 0.212686567164179 -1.54793571267631 0.00317347214442259 0.0246117956780223 0.0220010725702151 0.0939946813272062 0.329292929292929

hsa-miR-433 92.8245614035088 97.828947368421 0.948845550325039 -0.0525092435383428 0.623716409622044 0.771156535105766 0.629407289175477 0.77046594405452 0.532996632996633

hsa-miR-1233 119.964912280702 83.671052631579 1.43376841222414 0.36030623138362 0.00229340428194242 0.0200503012704972 0.00792868125725256 0.048528027836943 0.703367003367003

hsa-miR-545S 24.8421052631579 20.5614035087719 1.20819112627986 0.189124304116707 0.682870542301475 0.813974140892504 0.151208291880742 0.313684509358367 0.527272727272727

hsa-miR-550S 370.144736842105 334.570175438596 1.10632914711203 0.101047460192758 0.0116176638243068 0.0593310888541565 0.00371142468963076 0.0311751544482864 0.668350168350168

hsa-miR-33bS 159.868421052632 135.688596491228 1.17820085981187 0.163988579872519 0.520703021970249 0.692398625516679 0.950237109247445 0.978585471695161 0.543097643097643

hsa-miR-920 26.8245614035088 58.6140350877193 0.457647410954804 -0.781656236427693 0.0458526481334301 0.141238451330756 0.00303941972206729 0.0285110784798269 0.367676767676768

hsa-miR-520h 26.1228070175439 22.5964912280702 1.15605590062112 0.14501412601971 0.0820134871500836 0.213185660875067 0.442344363748988 0.619712964148339 0.615151515151515

hsa-miR-148bS 36.6666666666667 24.5087719298246 1.49606299212598 0.402836985701894 0.350271678707977 0.55145696567286 0.891987345903405 0.938762292091022 0.561616161616162

hsa-miR-31 149.412280701754 164.324561403509 0.909251054289222 -0.0951340356004822 0.816013022228347 0.898267757231818 0.492967693676357 0.672965078877669 0.484175084175084

hsa-miR-1299 3.64473684210526 24.0175438596491 0.151753104455807 -1.88550039155289 0.234849034013922 0.436429395650133 0.225507310186039 0.403761013880813 0.422222222222222

hsa-miR-96S 196.078947368421 158.815789473684 1.23463131731566 0.210772397022096 0.331567951777668 0.536853925673786 0.434550906291371 0.61377648466359 0.564983164983165

hsa-miR-200bS 77.9298245614035 58.9824561403509 1.32123735871505 0.278568690485812 0.648896230198599 0.790956845566936 0.30919404759793 0.505368301282223 0.469360269360269

hsa-miR-517b 18.6315789473684 30.5789473684211 0.609294320137694 -0.495453843718136 0.0946791777705834 0.234120717524394 0.0550083582987868 0.165986759482004 0.39023569023569

hsa-miR-941 116.947368421053 115.688596491228 1.01088069151154 0.0108219227002242 0.578019833379723 0.741205224675633 0.0610303957012124 0.177546603942006 0.462626262626263

hsa-miR-1203 204.765350877193 168.885964912281 1.21244741079312 0.192640970347131 0.204321041807124 0.400753473954298 0.704753735761321 0.814193405571647 0.584848484848485

hsa-miR-648 56.9473684210526 27.3333333333333 2.08344030808729 0.734020521643842 0.0141067197468184 0.0658059413054287 0.638157234763079 0.773496760674912 0.663636363636364

hsa-miR-517c 1 15.0877192982456 0.0662790697674419 -2.713881121413 0.0854184855257651 0.218742293794467 0.0322748974492985 0.116540738488471 0.390909090909091

hsa-let-7f 518.004385964912 479.741228070175 1.0797579104232 0.076736858993634 0.475787465097783 0.65388263065415 0.776225779693083 0.86214008735538 0.547811447811448

hsa-miR-450b-5p 72.7061403508771 56.3508771929825 1.2902397260274 0.254828035238322 0.0336126342055831 0.117165812810002 0.704510226653529 0.814193405571647 0.641750841750842

hsa-miR-423-3p 951.71600877193 1030.54385964912 0.92350849491837 -0.0795752807729455 0.557355428002078 0.721265040286934 0.433978940622128 0.61377648466359 0.460606060606061

hsa-miR-499-5p 19.7543859649123 18.4912280701754 1.06831119544592 0.0660790795983277 0.995935616200472 0.998278554087942 0.819664222691393 0.886428852359239 0.500673400673401

hsa-let-7c 501.850877192983 280.184210526316 1.79114617576156 0.582855736588727 0.0312066553716058 0.112683445965254 0.0269720593019416 0.105804032625344 0.643771043771044

hsa-miR-601 1 42.1052631578947 0.02375 -3.74017274850149 0.000160017164459483 0.00511462270105681 0.000151585909259387 0.00545077665378547 0.25993265993266

hsa-miR-571 55.0723684210526 58.3333333333333 0.944097744360902 -0.0575255754416243 0.823899074154428 0.904270714629757 0.400804109646482 0.587594386217373 0.484848484848485

hsa-miR-520d-5p 56.9824561403509 40.2807017543859 1.41463414634146 0.346870943842112 0.174551841920015 0.360378563581275 0.484600406837952 0.664881003340465 0.590572390572391

hsa-miR-208a 70.1403508771929 80.8771929824561 0.867245119305856 -0.142433620930349 0.265743709289391 0.468316577494036 0.274290790893166 0.470548779624891 0.425589225589226

hsa-miR-617 19.4035087719298 16.8245614035088 1.15328467153285 0.142614107198842 0.416254671316929 0.606803684706942 0.594397362806984 0.743427426235401 0.446801346801347

hsa-miR-136 27.140350877193 1 27.140350877193 3.30102158273852 0.00425997668504982 0.0308937804974621 0.530774722188883 0.702543842406452 0.686195286195286

hsa-miR-99aS 30.3333333333333 37.0175438596491 0.819431279620853 -0.199144740786821 0.256212300545297 0.46261044860492 0.125189376633358 0.279699823380193 0.424242424242424

hsa-miR-379S 52.4385964912281 43.6140350877193 1.20233306516492 0.184263890213281 0.685575634586498 0.81409232365497 0.411538380906194 0.596903567600077 0.527272727272727

hsa-miR-34c-5p 67.438596491228 59.438596491228 1.13459268004723 0.126273714318238 0.835727545711541 0.908353743008892 0.505728110080813 0.683981287425536 0.485858585858586

hsa-miR-523S 151.833333333333 132.421052631579 1.1465951245363 0.136796789378012 0.550681042126692 0.716799003552541 0.703279112052294 0.814193405571647 0.54006734006734

hsa-miR-641 61.6842105263158 45.4035087719299 1.35857805255023 0.306438603184275 0.103267229941096 0.24936882910527 0.670924482282579 0.792076372380117 0.608754208754209

hsa-miR-1206 71.0701754385965 32.2631578947368 2.20282762370854 0.789741818598369 0.0103955218712823 0.0563224220977389 0.0783515686394455 0.208053549956435 0.670707070707071

hsa-miR-202S 112.078947368421 71.5087719298245 1.56734543670265 0.449383383688763 0.00241056607453847 0.0200503012704972 0.00316361467047579 0.0290833840295413 0.702356902356902

hsa-miR-496 96.4210526315789 77.7894736842105 1.23951285520974 0.214718443725929 0.191985427742164 0.387110804068896 0.582921819108673 0.736546895886947 0.587205387205387

hsa-miR-551a 53.2982456140351 17.0175438596491 3.1319587628866 1.14165861165829 0.000633108091522084 0.0100891691075192 0.00244160567668238 0.0249896897703841 0.725925925925926

hsa-miR-1308 49.0526315789474 80.8771929824561 0.606507592190889 -0.500038032636993 0.0652854228409484 0.179430955132925 0.0277369179337299 0.107340628595556 0.377104377104377

hsa-miR-221S 127.267543859649 60.8070175438596 2.09297461050202 0.738586312717577 0.00112249468405803 0.0138387558906012 0.00639871758080296 0.0438261370812139 0.717171717171717

hsa-miR-21 794.078947368421 998.964912280702 0.794901740397956 -0.229536768950245 0.292692560774874 0.491724690296245 0.844382959481213 0.906346385612297 0.42962962962963

hsa-miR-200cS 45.8771929824562 32.5964912280702 1.40742734122713 0.34176345712524 0.235017452897187 0.436429395650133 0.716224951711608 0.820874802561432 0.579124579124579

hsa-miR-545 130.114035087719 94.3508771929824 1.37904425436966 0.321390689934858 0.0441427343883346 0.139033502836251 0.598791372575971 0.746758604816566 0.634343434343434

hsa-miR-668 51.280701754386 59.438596491228 0.862750885478159 -0.147629290660699 0.0390614480182842 0.128268507955002 0.0107305111374666 0.0590901388325603 0.362289562289562

hsa-miR-147 32.9122807017544 83.3026315789474 0.395092928973832 -0.928634278526176 0.00240642821165676 0.0200503012704972 0.00378823391788009 0.031435056453178 0.297643097643098

hsa-miR-149 28.6842105263158 23.4385964912281 1.22380239520958 0.201962729234762 0.378235448930491 0.577729544118609 0.33037148823794 0.526034306917606 0.441750841750842

hsa-miR-517S 203.710526315789 168.885964912281 1.20620163091466 0.187476274143487 0.13310149749577 0.298354785295714 0.391674464431025 0.582784591041335 0.6003367003367

hsa-let-7d 2523.52192982456 2746.45614035088 0.918828410455579 -0.0846558873831887 0.843538062836564 0.910087880834301 0.78213077455871 0.866468367707531 0.486531986531987

hsa-miR-181aS 86.6666666666667 70.4912280701754 1.22946739671478 0.206581064807089 0.0627165136726787 0.17459468161136 0.44890892754798 0.624852265280495 0.624242424242424

hsa-miR-516a-5p 203.271929824561 144.28947368421 1.408778649158 0.342723122745723 0.0463157378060301 0.141238451330756 0.310278731215065 0.505433428339144 0.632996632996633

hsa-miR-453 21.0657894736842 39.4385964912281 0.534141459074733 -0.627094570468647 0.173292927566239 0.359391692253223 0.118256739353517 0.272875845085789 0.40976430976431

hsa-miR-138-2S 67.1929824561403 45.2631578947369 1.48449612403101 0.395075404259027 0.121370640368866 0.277832526892125 0.109484904941324 0.260290559130475 0.603367003367003

hsa-miR-214 331.69298245614 342.780701754386 0.967653606981089 -0.0328810997748134 0.504443719668094 0.6759859162633 0.176710063037758 0.348972046685549 0.455218855218855

hsa-let-7f-1S 24.8421052631579 12.2456140350877 2.02865329512894 0.707372171491293 0.79860818481372 0.8879176813715 0.730824113576824 0.827691876662466 0.516835016835017

hsa-miR-370 57.578947368421 57.4736842105263 1.0018315018315 0.00182982667707616 0.808209100987319 0.894210838656482 0.288355711677157 0.482909649881394 0.483501683501684

hsa-miR-20a 4503.74342105263 4018.36622807017 1.12078968551743 0.114033513212295 0.10534923007244 0.251602244284499 0.0705522846589956 0.193681098268568 0.608080808080808

hsa-miR-502-3p 615.030701754386 580.348684210526 1.05976065508107 0.0580430855346469 0.357308370531977 0.556601306442412 0.292473216415267 0.485393049550722 0.561616161616162

hsa-miR-190b 1 1 1 0 0.152781067315218 0.329625152732582 0.0285876673089944 0.108875712416069 0.418855218855219

hsa-miR-656 33.4385964912281 40.6666666666666 0.822260569456428 -0.195697939685549 0.282997726948207 0.483617897735252 0.415341121632556 0.600512421022054 0.428619528619529

hsa-miR-591 59.2280701754386 40.8947368421052 1.44830544830545 0.370394216680091 0.141082938999243 0.311411815306531 0.188349439827775 0.361873208570481 0.598316498316498

hsa-miR-153 132.157894736842 119.052631578947 1.11007957559682 0.104431702481519 0.368043508172357 0.567181334915615 0.992028933775575 0.993697492374226 0.56026936026936

hsa-miR-744 615.030701754386 454.293859649123 1.35381689338573 0.302927931508919 0.0133992556772573 0.0643918329708517 0.00909379651220641 0.0533873904083955 0.664983164983165

hsa-miR-143 197.30701754386 204.765350877193 0.963576194403093 -0.0371037134001677 0.376146915084505 0.575558134251645 0.792682172260108 0.868232348409909 0.559259259259259

hsa-miR-302a 1 1 1 0 0.0482990454924373 0.143413378838107 0.0196783819609846 0.0908152065899983 0.384511784511784

hsa-miR-137 116.723684210526 62.1951754385965 1.87673213215331 0.629532036793342 0.00304227687440349 0.0238982402871194 0.183179675291383 0.35788996799432 0.697643097643098

hsa-miR-34b 51.280701754386 60.0350877192982 0.854178842781999 -0.157614689340893 0.843622102521694 0.910087880834301 0.345394616698898 0.54195555311118 0.486531986531987

hsa-miR-595 127.828947368421 255.043859649123 0.501203783319003 -0.69074250746716 0.00319411485045017 0.0246117956780223 0.00115814043449934 0.0178477713388023 0.303367003367003

hsa-miR-1915 543.850877192983 521.372807017544 1.04311323849823 0.0422097401140551 0.839681147813002 0.910087880834301 0.285697615334237 0.480643389728918 0.513804713804714

hsa-miR-376c 88.7368421052632 82.8421052631579 1.07115628970775 0.0687387095844522 0.656292559963235 0.797718984856721 0.911391808629189 0.953371067693321 0.52996632996633

hsa-miR-148a 804.166666666666 559.473684210526 1.4373628096582 0.362810052375478 8.49364033832496e-05 0.00348161686695204 0.000289001976537355 0.0083136235250579 0.761952861952862

hsa-miR-1915S 11.1228070175439 12.7478070175439 0.872527094443489 -0.136361571584288 0.917295790573221 0.958385311458462 0.551442321524548 0.709983923769388 0.507070707070707

hsa-miR-1909S 32.7368421052632 52.1929824561403 0.627226890756303 -0.466446936572441 0.173575754949334 0.359391692253223 0.0714940049367897 0.194635098613405 0.409090909090909

hsa-miR-211 1 1 1 0 0.00332419626347661 0.0253874457998258 0.000981044322458877 0.0164500597341995 0.337710437710438

hsa-miR-29c 483.480263157895 370.144736842105 1.30619245672034 0.267116383489147 0.236667254777638 0.436429395650133 0.338677909283196 0.533355904582843 0.579124579124579

hsa-miR-371-3p 35.6820175438596 24.3859649122807 1.46321942446043 0.380639093334745 0.347430061687278 0.549143119480075 0.499841243463475 0.67931180017162 0.562626262626263

hsa-miR-92a-2S 1 27.3333333333333 0.0365853658536585 -3.30810695859614 0.00123072007946675 0.0143528571429704 0.00358203576878491 0.0306068996877364 0.297306397306397

hsa-miR-1293 19.9649122807018 58.8421052631579 0.339296362552177 -1.08088132769688 0.00346841427054578 0.0260281870911392 0.00249850889116306 0.0249896897703841 0.306060606060606

hsa-miR-493 23.2982456140351 40.3859649122807 0.576889661164206 -0.550104259245448 0.353298030447938 0.55226132257401 0.0621645256561531 0.17823251043608 0.438383838383838

hsa-miR-629 59.5438596491228 57.4736842105263 1.03601953601954 0.0353860008212112 0.658949195206428 0.798810512912622 0.172741031266103 0.343179870857964 0.529292929292929

hsa-miR-205 67.438596491228 67.8070175438596 0.994566623544631 -0.00544819093133943 0.71197694809485 0.828103268763026 0.41750573883341 0.600512421022054 0.475084175084175

hsa-miR-221 102.5 96.3508771929824 1.0638201019665 0.061866299525152 0.265903966363937 0.468316577494036 0.669492424131086 0.791468441130311 0.574410774410774

hsa-miR-515-5p 203.271929824561 156.28947368421 1.30061177527081 0.262834750133911 0.00262220440405737 0.020953355562051 0.0113812060817136 0.0610060922268248 0.700673400673401

hsa-miR-563 8.24561403508773 29.6140350877193 0.278436018957346 -1.2785669804518 0.0846539132244538 0.218731518301508 0.0663016095272827 0.18577366565599 0.387542087542088

hsa-miR-9 1 22.5964912280702 0.0442546583850931 -3.11779463882975 0.034113521629311 0.118232807895965 0.00262129594270087 0.0251353155394539 0.366329966329966

hsa-miR-511 36.6666666666667 18.6315789473684 1.96798493408663 0.677010143156972 0.0208390460441599 0.0856385558862379 0.159261254656163 0.324750240730857 0.653535353535354

hsa-miR-128 538.96052631579 421.381578947368 1.27903200624512 0.246103546712936 0.0270885063998781 0.103536028864037 0.0317391479382039 0.116017507066131 0.647474747474748

hsa-miR-185 20902.4890350877 20902.4890350877 1 0 0.404539496858344 0.602966469410623 0.194989703726159 0.367954769953272 0.554208754208754

hsa-miR-215 350.850877192982 370.144736842105 0.947874823741305 -0.0535328279124669 0.696974409251443 0.821713151180781 0.790094129652706 0.868232348409909 0.473737373737374

hsa-miR-886-3p 1 16.140350877193 0.0619565217391304 -2.78132240220854 0.015167249539751 0.0696241295361974 0.00739879006180911 0.0469496751716269 0.346127946127946

hsa-miR-216a 186.359649122807 101.333333333333 1.83907548476454 0.609262991416304 0.000236432970425106 0.00551463928315856 0.00198012865969482 0.0230068944651514 0.745117845117845

hsa-miR-1197 42.2456140350877 49.8947368421053 0.846694796061885 -0.16641498449442 0.261081284011136 0.46309480511906 0.116741314041254 0.270574440144743 0.424915824915825

hsa-miR-558 99.3333333333333 87.9122807017544 1.12991418878467 0.12214169071212 0.723289852929959 0.83785119876316 0.602778432928492 0.749564535471597 0.523905723905724

hsa-miR-888S 42.2105263157895 42.4561403508772 0.994214876033058 -0.00580192261610772 0.726535191133245 0.837860912761269 0.972775517035135 0.985752953652734 0.523569023569024

hsa-miR-380S 99.3333333333333 71.0701754385965 1.39767958528758 0.334813422469155 0.00237031041023917 0.0200503012704972 0.00143368774742603 0.0195848504702865 0.702693602693603

hsa-miR-19a 2209.25438596491 1907.68201754386 1.15808314260326 0.146766175021825 0.398102812373167 0.596099996667326 0.47311571491715 0.6553753803748 0.556565656565657

hsa-miR-148b 568.565789473684 458.607456140351 1.23976569037657 0.214922402388139 0.0336530524820555 0.117165812810002 0.0134946372613039 0.0689104849497351 0.641750841750842

hsa-miR-654-5p 204.149122807017 142.09649122807 1.43669362306315 0.362344378424527 0.00363974044563163 0.027078413832587 0.0108614709348111 0.0593256292198858 0.693939393939394

hsa-miR-23a 3467.72368421053 4503.74342105263 0.769964751544402 -0.261410542397278 0.0445320125534168 0.139203002052117 0.0507988378698893 0.155458854899697 0.365993265993266

hsa-let-7dS 56.9824561403509 43.2478070175439 1.31758024440951 0.275796906151335 0.911324041863914 0.95758907527813 0.968270086993729 0.985752953652734 0.507744107744108

hsa-miR-362-3p 196.69298245614 225.798245614035 0.871100578843091 -0.137997833660794 0.987895475505065 0.995974060001018 0.658655122963686 0.785562559043648 0.501346801346801

hsa-miR-552 6.87719298245615 12.2456140350877 0.561604584527222 -0.576957262971908 0.705436594721159 0.824991918584993 0.419773460680114 0.602267339118274 0.475084175084175

hsa-miR-562 32.7368421052632 20.5614035087719 1.59215017064846 0.465085411270331 0.281876733812503 0.482657978730536 0.896298734063188 0.941212433256629 0.571380471380471

hsa-miR-337-3p 20.280701754386 31.7543859649123 0.638674033149171 -0.448361075027549 0.209005450348293 0.407082611201634 0.127377475807342 0.282054432066141 0.416835016835017

hsa-miR-323-5p 52.9824561403509 44.421052631579 1.19273301737757 0.176247327104848 0.453165105916868 0.635819555420603 0.356829658436276 0.549899991483046 0.44983164983165

hsa-miR-548o 133.280701754386 125.649122807018 1.06073722423904 0.0589641609448436 0.773141233256973 0.868789735649082 0.507118953842886 0.683981287425536 0.51952861952862

hsa-miR-1245 47.0175438596491 10.2456140350877 4.58904109589041 1.52367109067668 0.0136865184283738 0.0648981615587176 0.739520061001744 0.834255964241183 0.663299663299663

hsa-miR-1201 84.9912280701755 49.8947368421053 1.70341068917018 0.532632528901928 0.00596365004027414 0.0375666422245006 0.0209540725560608 0.0922620643667372 0.683164983164983

hsa-miR-218-2S 64.9122807017543 58.8421052631579 1.10316040548599 0.0981791562491613 0.685760172994513 0.81409232365497 0.571090111667343 0.72691853446743 0.527272727272727

hsa-miR-95 8.24561403508773 12.0701754385965 0.683139534883722 -0.381056143229238 0.403302857430447 0.602163262910857 0.120653109417308 0.275459347690839 0.445791245791246

hsa-miR-513c 1 24.9473684210526 0.040084388185654 -3.21676834252864 0.00101788702481566 0.0133096439759987 0.00140281824011457 0.0195848504702865 0.287878787878788

hsa-miR-582-5p 22.3859649122807 38.7719298245614 0.57737556561086 -0.549262330607063 0.0376626605641027 0.125011061795464 0.00351674254041253 0.0306068996877364 0.362626262626263

hsa-miR-146aS 100.701754385965 96 1.04897660818713 0.047815030011226 0.501119992944901 0.673685787122481 0.401837509021791 0.587772492009841 0.545117845117845

hsa-miR-19b-2S 1 1 1 0 0.236305530506072 0.436429395650133 0.0251721223882677 0.102955173559597 0.431986531986532

hsa-miR-92a 11503.9078947368 12702.7105263158 0.90562623393681 -0.0991286033830009 0.52482936788233 0.695741542983795 0.807756116196564 0.877626399805817 0.457575757575758

hsa-miR-30aS 42.2105263157895 30.140350877193 1.40046565774156 0.336804793990423 0.80777839555217 0.894210838656482 0.429751838212812 0.609783508626263 0.516498316498317

hsa-miR-885-3p 188.149122807017 200.342105263158 0.939139191733438 -0.0627915767515275 0.527308630459194 0.696887209933054 0.507239888704917 0.683981287425536 0.457575757575758

hsa-miR-451 998.964912280702 1212.00438596491 0.824225492785982 -0.193311130216051 0.44196090194397 0.622380744166776 0.539112588332513 0.704930551107513 0.448484848484848

hsa-miR-7 39.9824561403509 43.6140350877193 0.916733708769107 -0.0869382428249724 0.787409405397641 0.88051104880798 0.430310726249588 0.609783508626263 0.518181818181818

hsa-miR-518b 112.982456140351 106.964912280702 1.05625717566016 0.0547316931905833 0.609478952204676 0.758990383481437 0.398538384086108 0.586925982024422 0.534343434343434

hsa-miR-498 27.140350877193 36.8070175438596 0.737368922783604 -0.304666938383175 0.235002547657518 0.436429395650133 0.0243810049700643 0.100673719086916 0.420875420875421

hsa-miR-525-5p 110.320175438596 88.9122807017544 1.24077545382794 0.215736550147394 0.0480068161708322 0.143413378838107 0.591567129941508 0.740961441421657 0.631986531986532

hsa-miR-1537 30.7368421052632 39.4385964912281 0.779359430604982 -0.249282939517245 0.4277910094095 0.615306068533998 0.116945847246801 0.270574440144743 0.447138047138047

hsa-miR-31S 207.348684210526 147.565789473684 1.40512706197058 0.340127734262906 0.0059397407972569 0.0375666422245006 0.203231364521354 0.379224099974465 0.683501683501683

hsa-miR-372 33.4385964912281 24.5087719298246 1.36435218324982 0.310679724957185 0.705497144745915 0.824991918584993 0.416854143105646 0.600512421022054 0.525252525252525

hsa-miR-1257 1 24.0175438596491 0.04163623082542 -3.1787845574539 0.0093786179747439 0.0518829955910512 0.00443431788048881 0.035433484545017 0.334680134680135

hsa-miR-30a 304.815789473684 206.504385964912 1.47607416689676 0.389385973490902 0.274666589532158 0.478863165184347 0.365501221459608 0.556309619258628 0.573063973063973

hsa-miR-200b 64.719298245614 57.3333333333333 1.12882496940024 0.121177241616782 0.131805241292265 0.29621855009173 0.268832991323983 0.464005743025194 0.600673400673401

hsa-miR-605 10 27.3333333333333 0.365853658536585 -1.0055218656021 0.111615171749578 0.262462924304866 0.0899537011746044 0.229674686726875 0.395286195286195

hsa-miR-1228S 965.995614035088 1091.65789473684 0.884888588899871 -0.122293530147532 0.553982812625104 0.718928071120999 0.40272918804324 0.588080015704427 0.46026936026936

hsa-miR-1912 182.214912280702 156.921052631579 1.16118843982336 0.149443997747921 0.0468714558683445 0.14193005759432 0.281493925622005 0.478317978963018 0.632659932659933

hsa-miR-219-5p 56.9824561403509 61.3684210526316 0.928530588907948 -0.0741519542969786 0.591738042710308 0.745733906826103 0.693480142312431 0.808747787588686 0.536026936026936

hsa-miR-325 58.3333333333333 47.7543859649123 1.22152828802351 0.200102769878543 0.553664059306251 0.718928071120999 0.995250953938775 0.995250953938775 0.53973063973064

hsa-miR-1270 23.8070175438597 18.7719298245614 1.26822429906542 0.237617732378917 0.348308418145245 0.549524981461327 0.858526067251395 0.920112966708792 0.561616161616162

hsa-miR-659 88.7368421052632 87.0175438596491 1.01975806451613 0.0195654075109375 0.73093678360586 0.839944666114324 0.746490002791689 0.839922910572656 0.476767676767677

hsa-miR-190 1 1 1 0 0.0753337130922852 0.201278620429233 0.00407841155457208 0.0328941044074365 0.396969696969697

hsa-miR-494 66.8070175438596 69.8245614035087 0.95678391959799 -0.0441777023672273 0.696976230554848 0.821713151180781 0.98113179460534 0.990062398821924 0.526262626262626

hsa-miR-553 1 1 1 0 0.687439178517956 0.814917597611258 0.209028201020283 0.386276953919709 0.476430976430976

hsa-miR-183S 95.719298245614 113.929824561404 0.840160147828765 -0.174162753139494 0.297529213716653 0.496649345140177 0.301179075199838 0.496974267490364 0.43030303030303

hsa-miR-99b 156.28947368421 124.394736842105 1.25639940765813 0.228250017222623 0.148070243095272 0.322689034517631 0.122811868707266 0.276668849536775 0.596632996632997

hsa-miR-19b-1S 64.0877192982456 90.125 0.711098133683724 -0.340944836644381 0.119341604366134 0.273914373850995 0.0721275533431196 0.195128772837342 0.395959595959596

hsa-miR-644 1 6.77192982456141 0.147668393782383 -1.91278610163028 0.890358506401525 0.939271118785912 0.774205542685785 0.862115333339139 0.490909090909091

hsa-miR-515-3p 1 1 1 0 0.0652826039847715 0.179430955132925 0.0392022514714987 0.131130011705052 0.39023569023569

hsa-miR-369-3p 6.87719298245615 1 6.87719298245615 1.92821057195591 0.118905814694621 0.273641914883888 0.161143611356279 0.327216321412868 0.597979797979798

hsa-miR-647 85.4473684210527 34.2105263157895 2.49769230769231 0.915367228653218 0.00107468272360739 0.0136389880951938 0.00456695209581616 0.0359835339371774 0.717845117845118

hsa-miR-527 174.410087719298 158.429824561403 1.1008665079453 0.0960976042000717 0.341728152565479 0.545122727659905 0.516594578706289 0.691195537090741 0.563636363636364

hsa-miR-135bS 58.3333333333333 52.1929824561403 1.11764705882353 0.111225635110224 0.670831151363666 0.808557658696709 0.187676359155918 0.361775696427836 0.528619528619529

hsa-miR-638 287.973684210526 270.30701754386 1.0653577803018 0.0633106866902092 0.963698485031536 0.99008546735978 0.291788107728057 0.485393049550722 0.503367003367003

hsa-miR-510 71.0701754385965 96.3508771929824 0.73761835396941 -0.304328723685171 0.0655174113642095 0.179496907959723 0.023792321836444 0.0991921436949333 0.377104377104377

hsa-miR-584 78.4561403508772 59.438596491228 1.31995277449823 0.27759595906299 0.338252557092548 0.543597684861953 0.28612421390445 0.480643389728918 0.563636363636364

hsa-miR-335S 1 18.6315789473684 0.0536723163841808 -2.92485793396733 0.000424092960279913 0.00795635271133837 0.00215590182910707 0.023853118955377 0.28013468013468

hsa-miR-671-3p 26.8245614035088 51.1578947368421 0.524348422496571 -0.645598887199486 0.0418241463437437 0.13417932451543 0.0371167490954827 0.126506219947438 0.364983164983165

hsa-miR-454S 9.71929824561404 41.8771929824561 0.232090490155006 -1.46062793959155 0.00148041465952483 0.0155804615996333 0.00397135180393779 0.0323327981773426 0.28956228956229

hsa-miR-520d-3p 1 8.52631578947369 0.117283950617284 -2.14315735606594 0.0112617515201141 0.0586743230403389 0.00469369809856017 0.0359835339371774 0.350505050505051

hsa-miR-17 7477.03728070175 8005.76315789474 0.933956842493949 -0.0683250490008052 0.963609620202701 0.99008546735978 0.96415374930453 0.985752953652734 0.503367003367003

hsa-miR-1277 57.8245614035088 45.2631578947369 1.27751937984496 0.244920213113699 0.252430585750778 0.457702609792992 0.162826223948249 0.329084382359108 0.576430976430976

hsa-miR-566 99.1447368421052 83.3026315789474 1.19017532775233 0.174100630516469 0.0132108269732779 0.064058684743776 0.00252586156936191 0.0249896897703841 0.665319865319865

hsa-miR-24-2S 186.956140350877 144.600877192982 1.2929115229458 0.256896669718969 0.0271137225066888 0.103536028864037 0.0460705964807228 0.146711899493962 0.647474747474747

hsa-miR-105S 6.98245614035089 1 6.98245614035089 1.94340073744989 0.745672007572402 0.85121024144839 0.237520515559901 0.418326948833051 0.520538720538721

hsa-miR-551bS 46.3684210526316 58.7017543859649 0.789898386132696 -0.23585096694248 0.315302452081746 0.520279189572746 0.0495165904444137 0.15371517105586 0.432996632996633

hsa-miR-192 4503.74342105263 4503.74342105263 1 0 0.619442041531091 0.768900520546983 0.262990082878808 0.455743858482753 0.466666666666667

hsa-miR-328 54.4385964912281 52.578947368421 1.03536870203537 0.0347575971317171 0.596688164075328 0.750644148100595 0.719951978170114 0.821849943334403 0.464646464646465

hsa-miR-342-3p 3312.48684210526 3004.33333333333 1.10256968005279 0.0976435282036503 0.780744270929801 0.875041955600543 0.715771458604805 0.820874802561432 0.518855218855219

hsa-miR-1283 125.649122807018 83.1074561403509 1.51188748449745 0.413358859973495 0.000328541208618099 0.00691539178140047 0.000991197110287801 0.0164500597341995 0.739393939393939

hsa-miR-555 39.3684210526316 42.0350877192982 0.936560934891486 -0.065540692592753 0.459859643868188 0.64162011925623 0.0347967133740625 0.121086950168613 0.450505050505051

hsa-miR-1 15.4736842105263 20.5614035087719 0.752559726962457 -0.284274914130167 0.483350117908264 0.658974963277775 0.143459653617315 0.305513901229934 0.454208754208754

hsa-miR-645 63.8947368421052 74.0175438596491 0.863237734060204 -0.147065151836785 0.383911881477909 0.581256059149886 0.187716926397877 0.361775696427836 0.441750841750842

hsa-miR-889 32.7368421052632 31.4385964912281 1.04129464285714 0.0404647878724133 0.28617813747771 0.48712373302419 0.84394123345708 0.906346385612297 0.571043771043771

hsa-miR-1321 1 16.421052631579 0.0608974358974359 -2.79856420864304 0.0165316851207028 0.0731633038931615 0.00386421325833409 0.031760152780403 0.348821548821549

hsa-miR-664 216.552631578947 267.745614035088 0.80879992137077 -0.21220370849303 0.788684867588145 0.88051104880798 0.699235119190863 0.812166766974045 0.481818181818182

hsa-miR-548f 46.8771929824562 27.3333333333333 1.71501925545571 0.539424308225843 0.0561118614759516 0.161415121512487 0.0438814006785586 0.1427521468654 0.626599326599327

hsa-miR-513a-3p 49.0526315789474 66.4912280701754 0.737730870712402 -0.304176194722771 0.414471202165022 0.606793383317771 0.531866582415305 0.702910965734162 0.445454545454545

hsa-miR-1282 1 1 1 0 0.00533544050947581 0.0354191166129048 0.000396916253462083 0.00955580617104973 0.35016835016835

hsa-miR-222 458.607456140351 372.427631578947 1.23140018960472 0.20815188746988 0.0441293383859914 0.139033502836251 0.0569018917296631 0.169332181250687 0.634343434343434

hsa-miR-424S 153.228070175439 144.28947368421 1.06194905465378 0.0601059505332935 0.923452391442348 0.963651044516018 0.298535184286579 0.494502618117693 0.493265993265993

hsa-miR-624 67.438596491228 82.7719298245614 0.814752013565069 -0.204871489877438 0.0460948853779274 0.141238451330756 0.0613820251515626 0.177546603942006 0.367003367003367

hsa-miR-125a-5p 154.285087719298 192.394736842105 0.801919482058997 -0.220747072590847 0.588440086595752 0.745703075964954 0.655231561852659 0.785141755738159 0.463636363636364

hsa-miR-139-5p 155.53399122807 164.131578947368 0.9476177115066 -0.0538041159618798 0.659961389448019 0.798810512912622 0.361566064938233 0.552268166445479 0.52962962962963

hsa-miR-572 77.0175438596491 87.9649122807018 0.875548464299959 -0.132904772649022 0.0749592431624007 0.200900083382459 0.0329635434998881 0.117551810084312 0.381144781144781

hsa-let-7e 159.482456140351 168.885964912281 0.9443203656573 -0.0572898000100628 0.983852639707974 0.995974060001018 0.886685720950858 0.936068830088027 0.501683501683502

hsa-miR-141S 107.263157894737 109.074561403509 0.983392979211066 -0.0167464633311191 0.504430244101809 0.6759859162633 0.586219802017772 0.737416400409028 0.455218855218855

hsa-miR-940 164.131578947368 215.622807017544 0.761197672999471 -0.272862200580749 0.0354051020148215 0.119821972701141 0.0626429431346293 0.178584666951016 0.35959595959596

hsa-miR-1909 111.342105263158 148.28947368421 0.750842945874002 -0.286558775753809 0.000942025207963432 0.0127026211636319 0.000725349028976019 0.0140799344960282 0.279461279461279

hsa-miR-454 180.359649122807 168.885964912281 1.06793746429128 0.0657291847892659 0.659967434190846 0.798810512912622 0.647712490032837 0.780692568293769 0.52962962962963

hsa-miR-126S 1 34.5263157894737 0.0289634146341463 -3.54172180977765 1.05690044093268e-05 0.00182421016104981 8.94645473750305e-06 0.00193019760961628 0.214814814814815

hsa-miR-660 524.741228070176 357.271929824561 1.46874462913403 0.384408042143839 0.00196841999388787 0.0191416647744389 0.131247120422996 0.287477829758998 0.706397306397306

hsa-miR-410 89.7543859649123 103.719298245614 0.865358592692828 -0.144611299929304 0.727141785522293 0.837860912761269 0.691475172392014 0.807500776419903 0.523569023569024

hsa-miR-1266 120.210526315789 100.175438596491 1.2 0.182321556793955 0.0112861386149435 0.0586743230403389 0.00720870275530139 0.0460822998357415 0.669023569023569

hsa-miR-548i 1 1 1 0 0.167570819786613 0.349308254772578 0.014872881883974 0.0746238201504042 0.415824915824916

hsa-miR-720 3004.33333333333 2295.07456140351 1.30903517639797 0.269290359292419 0.00481401619634366 0.0335039998181014 0.0809499926374187 0.212339950292074 0.687878787878788

hsa-miR-30d 4726.70175438596 4936.95614035088 0.957412142221306 -0.0435213196085313 0.879157066500376 0.932353719342829 0.587027887695252 0.737416400409028 0.51043771043771

hsa-miR-587 45.2631578947369 56.3508771929825 0.803237858032379 -0.219104397150989 0.351457723907695 0.55145696567286 0.321861311887717 0.516294260518772 0.437710437710438

hsa-miR-624S 23.8070175438597 39.1578947368421 0.607974910394266 -0.497621663666332 0.114600884436432 0.265907339166088 0.00573656219527635 0.0406990303265538 0.395959595959596

hsa-miR-548k 15.7894736842105 21.0657894736842 0.749531542785758 -0.288306877220624 0.379229795755892 0.578224935931687 0.288738562385631 0.482909649881394 0.442087542087542

hsa-miR-501-5p 32.8245614035088 51.280701754386 0.640095791994526 -0.446137438837156 0.267589387657219 0.470325135536008 0.065549883101257 0.184265632300928 0.425925925925926

hsa-miR-890 20.280701754386 21.0657894736842 0.962731626067041 -0.0379805913126116 0.877854693033358 0.932353719342829 0.5428942345846 0.706886175757399 0.51043771043771

hsa-miR-374aS 31.8771929824561 44.421052631579 0.717614533965245 -0.331822714867896 0.319889127216595 0.526840299213591 0.540117116023797 0.705175599286742 0.433670033670034

hsa-miR-1279 1 13.9649122807018 0.071608040201005 -2.63654791800983 0.000385663653563989 0.00756426665967551 0.00466558631213242 0.0359835339371774 0.281144781144781

hsa-miR-450b-3p 43.3377192982456 16.8245614035088 2.57586027111575 0.946183564191634 0.060198317569861 0.169221980660554 0.867362256849092 0.92411558970465 0.624242424242424

hsa-miR-155 95.0526315789473 67.719298245614 1.40362694300518 0.339059560186164 0.0320175966352858 0.114178454116742 0.10085888013362 0.247276174873051 0.643097643097643

hsa-miR-1286 145.563596491228 93.1578947368421 1.56254708097928 0.446317234001206 0.0452087114663844 0.139889622633554 0.992546046844244 0.993697492374226 0.633670033670034

hsa-miR-485-3p 56.9473684210526 74.4210526315789 0.765205091937766 -0.267611387050091 0.42345117741453 0.613334029357467 0.201919822546025 0.377997411837786 0.446464646464647

hsa-miR-520a-3p 1 11.8421052631579 0.0844444444444445 -2.47166142303798 0.00241625878578413 0.0200503012704972 0.0286382233122221 0.108875712416069 0.311784511784512

hsa-miR-922 169.236842105263 116.339912280702 1.45467568943092 0.37478298191702 0.050906189996166 0.149428714172419 0.212254278538416 0.389734983784368 0.63030303030303

hsa-miR-26a-1S 8.24561403508773 16.9473684210526 0.486542443064183 -0.720431139508413 0.323897725855047 0.528400259759746 0.102103726909755 0.248212722036954 0.435690235690236

hsa-miR-516b 32.8245614035088 31.7543859649123 1.03370165745856 0.0331462020142182 0.47705793739413 0.654532591369053 0.103612287149784 0.250669731928025 0.452525252525253

hsa-miR-487b 63.7543859649122 42.3157894736842 1.50663349917081 0.40987769110904 0.00529497873475507 0.0354191166129048 0.00242356307233481 0.0249896897703841 0.685858585858586

hsa-miR-204 1 12.0701754385965 0.0828488372093023 -2.49073757009879 3.26964088441508e-05 0.00236979110557927 0.00101391796690074 0.0165096453855723 0.262289562289562

hsa-miR-1471 132.421052631579 110.530701754386 1.19804769651998 0.180693312356466 0.148049730463115 0.322689034517631 0.474991163633361 0.656918868935241 0.596632996632997

hsa-miR-140-3p 17675.4912280702 20902.4890350877 0.845616576973174 -0.167689240818386 0.00963271283751608 0.0529492431769196 0.00715364433772414 0.0460822998357415 0.332659932659933

hsa-miR-18aS 188.149122807017 158.043859649123 1.19048676250208 0.174362267607095 0.0679110681336095 0.18372179247431 0.0826979301186379 0.215614240762491 0.621885521885522

hsa-miR-218-1S 100.491228070175 110.936403508772 0.905845375294048 -0.0988866549503667 0.257303609884544 0.46261044860492 0.222557141750945 0.400974558102433 0.424242424242424

hsa-miR-181a 626.328947368421 287.973684210526 2.17495202412501 0.777006606423811 3.76032027752946e-05 0.00236979110557927 0.0003954507108141 0.00955580617104973 0.774747474747475

hsa-miR-1179 23.280701754386 15.0877192982456 1.54302325581395 0.433743645084654 0.696980332171879 0.821713151180781 0.0833098583143154 0.216555444955585 0.474410774410774

hsa-miR-1289 108.355263157895 101.333333333333 1.0692953601108 0.0669998895872976 0.475826526130714 0.65388263065415 0.558466945816836 0.715069694717997 0.547811447811448

hsa-miR-665 120.210526315789 115.087719298246 1.04451219512195 0.0435499775058755 0.667305061397236 0.805432542637503 0.566838158196865 0.724713082257621 0.471043771043771

hsa-miR-223 1601.20175438596 1799.94736842105 0.889582541399846 -0.11700298090339 0.290393588212419 0.490677226624908 0.0432874186901826 0.142584131029113 0.429292929292929

hsa-miR-301b 181.407894736842 178.5 1.01629072681704 0.0161594566656042 0.523994002802584 0.695702806797893 0.379365835527299 0.56937863662619 0.542760942760943

hsa-miR-488S 88.7368421052632 60.5614035087719 1.46523754345307 0.382017375026592 0.00930540732156495 0.0518829955910512 0.0276619563992556 0.107340628595556 0.673400673400673

hsa-miR-548d-3p 45.8771929824562 24.0175438596491 1.91015339663988 0.647183551210575 0.0110581325634078 0.0581900512330546 0.0206201549505892 0.0922620643667372 0.669023569023569

hsa-miR-365 79.2631578947368 102.019736842105 0.77693944670149 -0.252392863824474 0.22101626398728 0.422920256809363 0.0913657936411685 0.230538738200371 0.418181818181818

hsa-miR-99bS 59.5438596491228 78.5614035087719 0.757927646270656 -0.277167351364529 0.482054737874024 0.658316889450965 0.072706093226705 0.19607924517077 0.452861952861953

hsa-miR-590-5p 192.657894736842 96.3508771929824 1.99954479242535 0.692919550866946 6.1530711061656e-07 0.000460424534777681 0.000303643949012848 0.00845305574187379 0.832323232323232

hsa-miR-122 10 49.6491228070175 0.201413427561838 -1.60239562980869 0.000441546156829381 0.00810753900731395 0.000488295815936165 0.0113891699771057 0.267676767676768

hsa-miR-181cS 73.719298245614 69.8245614035087 1.05577889447236 0.0542787831264626 0.0957017898978317 0.235300981999512 0.0730416500634603 0.196370542070923 0.611111111111111

hsa-miR-151-3p 471.390350877193 426.524122807017 1.10519036479087 0.10001759596078 0.64536177007316 0.787789298883495 0.525007466256505 0.695977639599638 0.530976430976431

hsa-miR-195S 64.2807017543859 77.1929824561403 0.832727272727273 -0.183049094112332 0.475589237125693 0.65388263065415 0.262443286438157 0.455711380676317 0.452188552188552

hsa-miR-522S 169.587719298246 93.4035087719298 1.81564613072878 0.596441399257555 0.000299481798350558 0.00646131979941328 0.000194901413073164 0.00622962664748669 0.741077441077441

hsa-miR-613 1 12.0701754385965 0.0828488372093023 -2.49073757009879 0.196775769875199 0.390589070121332 0.579841630681075 0.734806647984975 0.42020202020202

hsa-miR-1231 110.320175438596 89.0877192982456 1.2383320204805 0.213765329323799 0.439064872175358 0.62219517257345 0.751693275902554 0.843577759562945 0.551851851851852

hsa-miR-326 98.4649122807017 103.719298245614 0.949340324763193 -0.0519879305775281 0.584964199623358 0.743481744145741 0.242641386095047 0.424745468965569 0.463299663299663

hsa-miR-374b 506.010964912281 458.607456140351 1.1033640167364 0.0983637100650566 0.392731348895389 0.590465425255611 0.153125072624687 0.316899131115359 0.557239057239057

hsa-miR-1914 25.6666666666667 59.5438596491228 0.431054802592811 -0.841520044774046 0.125608085739337 0.284513853000125 0.239788138197241 0.421460617646068 0.397979797979798

hsa-miR-23bS 35.6820175438596 31.7543859649123 1.12368784530387 0.116615995199611 0.99186177748816 0.9965431913147 0.19258667954637 0.365279789996742 0.498989898989899

hsa-miR-596 79.4912280701754 96.2456140350877 0.825920524972658 -0.19125672683041 0.20432390329072 0.400753473954298 0.0481513756123829 0.151659259684257 0.415151515151515

hsa-miR-551b 72.8157894736842 60.0350877192982 1.21288720046756 0.193003633442951 0.424029890085996 0.613334029357467 0.668108938077689 0.790916342333396 0.553535353535354

hsa-miR-640 119.842105263158 101.631578947368 1.17918177110306 0.164820783635225 0.281409105331754 0.482657978730536 0.989406253859423 0.992857671024048 0.572053872053872

hsa-miR-490-5p 147.864035087719 106.157894736842 1.39286894728144 0.33136561088982 0.00207163762078983 0.0192239060939959 0.00322299857199805 0.0292783975540455 0.705387205387205

hsa-miR-1296 43.4758771929825 35.3508771929824 1.22983870967742 0.20688303044243 0.591651222853329 0.745733906826103 0.225324272636745 0.403761013880813 0.463973063973064

hsa-miR-10b 57.578947368421 62.1951754385965 0.925778357603752 -0.077120427625428 0.261482854184601 0.46309480511906 0.128358440372028 0.283137822923815 0.424915824915825

hsa-miR-362-5p 143.377192982456 131.666666666667 1.08894070619587 0.0852053945308027 0.526174548307203 0.696454961946497 0.509693649136365 0.685337038406805 0.542760942760943

hsa-miR-564 172.923245614035 122.078947368421 1.41648703025077 0.348179884052215 0.0044094088745589 0.0317109988228694 0.0124913006375827 0.0649397135556259 0.68989898989899

hsa-miR-20b 3921.35964912281 2637.20614035088 1.48693709950223 0.396718366315839 0.0106150814778756 0.0565482426876955 0.013003866461482 0.0671996212949641 0.67037037037037

hsa-miR-1469 168.44298245614 159.482456140351 1.05618502832627 0.0546633861535643 0.674676766694986 0.809799790900936 0.320992360538581 0.516294260518772 0.528282828282828

hsa-miR-331-3p 708.070175438597 666.407894736842 1.06251768778671 0.0606412690065353 0.734709463128385 0.84203753875139 0.970926208597043 0.985752953652734 0.522895622895623

hsa-miR-512-5p 63.7543859649122 57.9649122807017 1.0998789346247 0.0952001143152476 0.543947710848359 0.713414702829991 0.546737784610198 0.708496619614802 0.540740740740741

hsa-miR-589S 84.9912280701755 88.1578947368421 0.96407960199005 -0.0365814131022174 0.927467501403391 0.966672045544838 0.955193868452035 0.97901699343718 0.506397306397306

hsa-miR-181b 51.280701754386 40.8947368421052 1.25396825396825 0.226313126075489 0.63788972403111 0.784186370140809 0.586353154705862 0.737416400409028 0.531649831649832

hsa-miR-519a 27.140350877193 12.0701754385965 2.24854651162791 0.810284012639723 0.257938629733621 0.462788019667599 0.78874656818452 0.868232348409909 0.574410774410774

hsa-miR-1205 77.6491228070175 57.1578947368421 1.3585021485574 0.306382731449004 0.046158358120957 0.141238451330756 0.58144685134456 0.735760458519582 0.632996632996633

hsa-miR-548p 142.28947368421 127.548245614035 1.11557374230597 0.109368839658392 0.0572459446190059 0.163278051234304 0.0417371498351616 0.138004445623542 0.626936026936027

hsa-miR-943 36.6666666666667 56.3508771929825 0.650684931506849 -0.429729730107796 0.108282153563824 0.257431125414821 0.0209249509019781 0.0922620643667372 0.392929292929293

hsa-miR-520b 9.71929824561404 29.1578947368421 0.333333333333334 -1.09861228866811 0.0350630623049769 0.119821972701141 0.00232848490115139 0.0245058837767518 0.361952861952862

hsa-miR-548a-3p 84.9912280701755 62.563596491228 1.35847733884819 0.306364468735339 0.0216374710646708 0.0880808374000513 0.0168005677797117 0.0806770726697786 0.653198653198653

hsa-miR-921 64.4736842105263 63.1929824561403 1.02026651860078 0.0200638859110354 0.863478846636161 0.922255253275999 0.333243568579637 0.529630201996735 0.488215488215488

hsa-miR-708 33.7543859649123 57.1578947368421 0.590546347452425 -0.526707157936339 0.0758897335450796 0.202138395214209 0.0338486585457232 0.119230172754935 0.381818181818182

hsa-miR-198 41.8771929824561 84.0921052631579 0.497991967871486 -0.697171330859671 0.00513239867379647 0.0348760634290264 0.0083584404852603 0.0500925981859697 0.313468013468013

hsa-miR-130bS 1 16.9473684210526 0.0590062111801242 -2.83011256637797 0.000175820821064899 0.00541904887782171 6.82866744837923e-05 0.00342600819923949 0.273400673400673

hsa-miR-1306 25.6666666666667 99.1447368421052 0.258880778588808 -1.35138763758869 0.01801076084158 0.0785014475064823 0.00232646833255116 0.0245058837767518 0.344781144781145

hsa-let-7i 783.991228070176 372.427631578947 2.10508340840973 0.744355090296954 0.000227048199473518 0.00544677583223579 0.00110217297424563 0.017294095941345 0.745791245791246

hsa-miR-302cS 10.3684210526316 16.8245614035088 0.616266944734098 -0.48407505747734 0.547405002658959 0.713611053315229 0.055987145120979 0.167767035553489 0.46026936026936

hsa-miR-632 23.8070175438597 24.0175438596491 0.991234477720965 -0.00880416545357944 0.676504713737462 0.809833595356602 0.122347167774957 0.276402109397351 0.472053872053872

hsa-miR-580 46.5263157894737 36.280701754386 1.28239845261122 0.248732115677434 0.285759864603077 0.48712373302419 0.536679347023296 0.704930551107513 0.571043771043771

hsa-miR-10bS 79.2631578947368 52.1929824561403 1.51865546218487 0.417825379048218 0.260921095952088 0.46309480511906 0.801492939024812 0.873831537695639 0.575084175084175

hsa-miR-200c 112.078947368421 87.5789473684211 1.27974759615385 0.246662867982114 0.152380521873983 0.329584938288841 0.768671504481897 0.858167539932571 0.595622895622896

hsa-miR-1274b 620.679824561404 639.035087719298 0.971276595744681 -0.029143994669081 0.979825127399655 0.995974060001018 0.938286525756818 0.970592844969367 0.497979797979798

hsa-miR-488 1 3.29166666666667 0.30379746835443 -1.19139402211908 2.38267023726506e-05 0.00236979110557927 0.0226690881826535 0.0958991328511272 0.263636363636364

hsa-miR-625 70.0526315789473 78.2631578947368 0.895090786819099 -0.110830128065044 0.429825847407371 0.615662427367514 0.3681739023153 0.559390981862859 0.447138047138047

hsa-miR-299-3p 1 3.08771929824561 0.323863636363636 -1.1274327272036 0.39855121445776 0.596099996667326 0.0117264054647383 0.0620852019390747 0.446801346801347

hsa-miR-320a 12702.7105263158 6067.7149122807 2.09349165377006 0.738833319734011 2.68450855990929e-06 0.000579182721800428 8.88526772123748e-06 0.00193019760961628 0.812457912457912

hsa-miR-486-5p 39283.3070175439 39283.3070175439 1 0 0.00673686411582456 0.0409430544503986 0.0218174717142634 0.09367402034532 0.404713804713805

hsa-miR-589 90.3552631578947 58.8421052631579 1.53555456171735 0.428891593785741 0.00769073303470772 0.0451503578840324 0.0217557306546633 0.09367402034532 0.677777777777778

hsa-miR-342-5p 143.675438596491 100.175438596491 1.43423817863397 0.360633822272378 0.114604495826419 0.265907339166088 0.147893109924828 0.310539547117097 0.605387205387205

hsa-miR-639 71.5087719298245 57.3333333333333 1.24724602203182 0.220937938362721 0.333232061231951 0.538537956635156 0.60754477571193 0.754404520056684 0.564646464646465

hsa-miR-518eS 175.798245614035 152.219298245614 1.15490116982654 0.144014773062925 0.0828093094399659 0.214607910050122 0.860455899374736 0.920165354597766 0.615824915824916

hsa-miR-224 26.1228070175439 30.7368421052632 0.849885844748859 -0.162653238812327 0.62343062951844 0.771156535105766 0.39769283787935 0.586681912974153 0.467340067340067

hsa-miR-181d 15.6315789473684 26.4561403508772 0.590848806366047 -0.526195121097093 0.604216473766227 0.75680524943433 0.230670056473121 0.408107407563974 0.465656565656566

hsa-miR-192S 130.114035087719 82.7719298245614 1.57195845697329 0.452322266796047 8.87550070370161e-05 0.00348161686695204 0.000525532423458292 0.0119351179327501 0.761279461279461

hsa-miR-30b 5447.35964912281 7477.03728070175 0.728545203751017 -0.316705604824409 0.0134305097737582 0.0643918329708517 0.204922822369381 0.3811387838465 0.335353535353535

hsa-miR-183 352.675438596491 259.482456140351 1.35914945404144 0.30685910266279 0.207915525223645 0.405952711013588 0.195084332186058 0.367954769953272 0.584175084175084

hsa-miR-105 57.8245614035088 50.6754385964912 1.14107668339969 0.131972275808042 0.587764012867485 0.745703075964954 0.241399085135802 0.423429696081702 0.536363636363636

hsa-miR-1246 1 12.0701754385965 0.0828488372093023 -2.49073757009879 0.0307553923384319 0.111520603311205 0.0706947230064877 0.193681098268568 0.37037037037037

hsa-miR-20aS 175.798245614035 119.964912280702 1.46541386370284 0.382137703394236 0.000665586719979333 0.0100891691075192 0.00951140802469098 0.0547850292630625 0.726936026936027

hsa-miR-142-3p 18.3684210526316 79.4912280701754 0.231074817920989 -1.4650137337966 0.00233344217427371 0.0200503012704972 0.00938866968097117 0.0547460941532305 0.3003367003367

hsa-miR-1292 77.6491228070175 67.298245614035 1.15380604796663 0.143066084607666 0.22674400825645 0.428861419211079 0.8019404146639 0.873831537695639 0.580808080808081

hsa-miR-335 399.05701754386 409.763157894737 0.973872369575921 -0.0264750213221368 0.835724473194958 0.908353743008892 0.446917982220386 0.623502291404031 0.514141414141414

hsa-miR-301a 360.043859649123 223.010964912281 1.6144670724632 0.47900491612633 0.000129013039622018 0.00463909388307507 0.000114791851466857 0.00471739846742371 0.755218855218855

hsa-miR-526b 29.8245614035088 39.4385964912281 0.756227758007118 -0.279412680969274 0.991922070689971 0.9965431913147 0.84040316613827 0.905453099097786 0.501010101010101

hsa-miR-374a 374.71052631579 494.798245614035 0.757299625932952 -0.277996296810783 0.213466318023776 0.413097354271073 0.406698792105051 0.591873621562662 0.416835016835017

hsa-miR-628-5p 85.6052631578948 70.4912280701754 1.21441015430563 0.194258489206637 0.321581140305247 0.528400259759746 0.824947200671182 0.890875652779076 0.566329966329966

hsa-miR-1183 100.701754385965 177.114035087719 0.568570155019563 -0.564630569676817 0.00139338580218571 0.0150361655662008 0.00133765240993169 0.019566000504594 0.286868686868687

hsa-miR-519d 72.7061403508771 57.1578947368421 1.27202271332106 0.240608321142985 0.381484856596875 0.579821106180108 0.386430194417078 0.577970984024156 0.558585858585859

hsa-miR-130b 998.964912280702 828.118421052631 1.20630683593647 0.187563490435837 0.0901583209835772 0.227056557654232 0.286269643477015 0.480643389728918 0.613131313131313

hsa-miR-29b 348.622807017544 298.953947368421 1.16614217703542 0.153701016202656 0.0941303458011297 0.233504671880996 0.046453702894341 0.147388035286089 0.611784511784512

hsa-miR-380 22.3859649122807 12.0701754385965 1.8546511627907 0.617696625971392 0.387191804968271 0.583152753381532 0.339336176817929 0.533419163194668 0.556565656565657

hsa-miR-296-5p 201.236842105263 296.605263157895 0.678466861857865 -0.387919641197854 0.108908934387078 0.258133054375645 0.00953639403551989 0.0547850292630625 0.392929292929293

hsa-miR-1274a 155.53399122807 109.074561403509 1.42594193574329 0.354832602890508 0.130523686174938 0.294104285036479 0.198148558851309 0.372553826336993 0.601010101010101

hsa-miR-16 14207.2368421053 17675.4912280702 0.803781725711983 -0.218427532094869 0.00202163451557525 0.0191722042521038 0.000888069879787943 0.0159667563803541 0.296296296296296

hsa-miR-150 1405.51754385965 2005.4298245614 0.700856009343055 -0.355452820545765 0.362495093877117 0.563517321416392 0.688767507047979 0.805428670165862 0.439057239057239

hsa-miR-519aS 168 126.916666666667 1.32370321733421 0.280433276296097 0.0274550028096215 0.103919593967997 0.0895199809390933 0.229674686726875 0.647138047138047

hsa-miR-548c-5p 1 18.4912280701754 0.0540796963946869 -2.91729646126676 0.035137775431693 0.119821972701141 0.129880076634343 0.285207394746662 0.365319865319865

hsa-miR-367 101.122807017544 46.9824561403509 2.15235250186707 0.766561430868295 5.98911398422783e-05 0.00287022034727864 5.10224870043011e-05 0.00308503027500963 0.767340067340067

hsa-miR-623 42.0350877192982 71.0701754385965 0.591458899037275 -0.52516308396735 0.0358441793159693 0.120363917313936 0.0384210754744969 0.12992608328188 0.35993265993266

hsa-miR-518a-3p 71.3508771929824 31.7543859649123 2.24696132596685 0.809578781647353 0.000490003586477971 0.00863006316592835 0.0010679715343589 0.0170677672991061 0.732323232323232

hsa-miR-220a 61.0175438596491 51.1578947368421 1.19272976680384 0.176244601785679 0.453841073162331 0.635819555420603 0.973452371075107 0.985752953652734 0.55016835016835

hsa-miR-19aS 53.4035087719298 57.9649122807017 0.921307506053269 -0.0819614156592919 0.546394499763034 0.713611053315229 0.481815402666006 0.662295209621432 0.45959595959596

hsa-miR-218 42.0350877192982 24.5087719298246 1.71510379384395 0.539473599978378 0.475763360398211 0.65388263065415 0.685958142536836 0.803231854829429 0.547474747474748

hsa-miR-214S 67.1929824561403 72.8157894736842 0.922780387905072 -0.0803640057203987 0.947582478624408 0.980531989272019 0.865673876539735 0.923456805258086 0.504713804713805

hsa-miR-544 42.2456140350877 36.280701754386 1.16441005802708 0.152214570800392 0.707443774738937 0.826148819485389 0.634371941025471 0.771861211803507 0.525252525252525

hsa-miR-574-5p 937.436403508772 1470.35964912281 0.637555855173278 -0.450113389829527 0.00183032907661604 0.0183131630284012 0.00257715224746719 0.0249896897703841 0.292255892255892

hsa-miR-891a 136.513157894737 91.4035087719298 1.49352207293666 0.401137137899706 0.0367054142898742 0.122778188109153 0.0775272174774198 0.206499965071029 0.639393939393939

hsa-miR-936 52.9824561403509 60.8070175438596 0.87132140796307 -0.137744359906636 0.591906831484263 0.745733906826103 0.502369113216882 0.68105358163561 0.463973063973064

hsa-miR-193a-3p 154.285087719298 174.410087719298 0.884611032176132 -0.122607242253157 0.304620415921171 0.505552728730713 0.0971840377917725 0.241699782750143 0.431313131313131

hsa-miR-193bS 19.9649122807018 53.6666666666667 0.372016999019287 -0.988815729464627 0.0330516025620872 0.116899725455251 0.0260845227670894 0.103702212210388 0.358249158249158

hsa-miR-1297 49.4035087719298 37.7543859649123 1.30855018587361 0.268919795996258 0.975738769726446 0.995974060001018 0.39450084659791 0.583809996845114 0.502356902356902

hsa-miR-1226 55.6315789473684 88.9122807017544 0.625690607734807 -0.468899266902682 0.0254039784768395 0.0996528792068749 0.0155503778005823 0.077126299091394 0.350841750841751

hsa-miR-1262 1 4.42105263157895 0.226190476190476 -1.48637781967687 0.0024741165203231 0.0201169335516716 0.00316783093716905 0.0290833840295413 0.321548821548822

hsa-miR-1288 102.304824561404 87.9649122807018 1.16301854806542 0.151018821874887 0.645340880566701 0.787789298883495 0.422199909619565 0.603242586095504 0.469023569023569

hsa-miR-134 52.9824561403509 23.6491228070176 2.24035608308605 0.806634818896662 0.134852215208453 0.300716955361486 0.706148074088097 0.814713620238006 0.5996632996633

hsa-miR-411S 59.3333333333333 45.8771929824562 1.29330783938814 0.257203152959501 0.29200142585859 0.491724690296245 0.314400871365254 0.509058071272447 0.57037037037037

hsa-miR-302dS 54.4385964912281 60.0350877192982 0.906779661016949 -0.0978557900037583 0.279138149286018 0.480830784099469 0.124103846895924 0.278186025639436 0.427609427609428

hsa-miR-195 604.504385964912 741.741228070176 0.814980161663227 -0.204591507555047 0.181750978863378 0.369252168317298 0.265199685193891 0.458651960565787 0.410774410774411

hsa-miR-138-1S 116.5 110.530701754386 1.0540057934209 0.0525979467083299 0.605987315758933 0.75792326594197 0.253900666341998 0.442659141521503 0.534680134680135

hsa-miR-223S 23.280701754386 62.563596491228 0.372112587191981 -0.988558816757512 0.00163592242316499 0.0166094241316634 7.14578766932919e-05 0.00342600819923949 0.290572390572391

hsa-miR-744S 44.7017543859649 47.7543859649123 0.936076414401176 -0.0660581665193572 0.500618292818051 0.673685787122481 0.0671439462991827 0.187525002123607 0.454882154882155

hsa-miR-892b 32.7368421052632 40.9824561403509 0.798801369863014 -0.224642962540828 0.21865327856177 0.422142683218808 0.112462167583932 0.264454633855403 0.417845117845118

hsa-miR-578 1 20.859649122807 0.0479394449116905 -3.03781662885623 0.010584255420975 0.0565482426876955 0.113960923363931 0.267250752345305 0.338047138047138

hsa-miR-149S 454.293859649123 396.359649122807 1.14616576297444 0.136422252661846 0.409763315803927 0.606562163874424 0.420497619928546 0.602267339118274 0.555218855218855

hsa-miR-1200 84.9912280701755 69.8245614035087 1.21721105527638 0.196562221546576 0.0502202723586688 0.14791841312468 0.0691176870678051 0.19179602552899 0.630639730639731

hsa-miR-1301 229.377192982456 130.570175438597 1.75673496808868 0.563456954394207 0.00038499108557152 0.00756426665967551 0.00179636521686358 0.0222258115415021 0.736700336700337

hsa-miR-26b 409.763157894737 599.291666666667 0.683745796389744 -0.380169073140904 0.180159735874721 0.367559933947718 0.0703594768139488 0.193681098268568 0.41043771043771

hsa-let-7iS 266.921052631579 191.956140350877 1.39053146277933 0.329686021389746 0.00154616736749113 0.0160764149174077 0.028008668139638 0.107590307510121 0.711111111111111

hsa-miR-1256 64.9122807017543 34.2105263157895 1.8974358974359 0.640503447074522 0.133872812982716 0.299306315036486 0.154325380169216 0.318619146138836 0.6

hsa-miR-518fS 122.482456140351 121.90350877193 1.00474922645175 0.00473798445561271 0.442085047710583 0.622380744166776 0.716001475542919 0.820874802561432 0.551515151515152

hsa-miR-548b-3p 45.8771929824562 42.1052631578947 1.08958333333333 0.0857953601629871 0.863441217645612 0.922255253275999 0.218256301827574 0.397330770626717 0.488215488215488

hsa-miR-675 130.570175438597 158.956140350877 0.821422658793665 -0.19671749226228 0.70446106913647 0.824991918584993 0.491980138123507 0.672866654834528 0.474410774410774

hsa-miR-576-5p 1 28.6842105263158 0.0348623853211009 -3.3563468154968 0.000147696182932497 0.00500449206248294 6.42519924791758e-05 0.00342600819923949 0.263299663299663

hsa-miR-32 167.684210526316 156.921052631579 1.06858963608922 0.0663396819068337 0.114620544808557 0.265907339166088 0.121518915274519 0.27579969910352 0.605387205387205

hsa-miR-187 8.10526315789475 34.2105263157895 0.236923076923077 -1.44001976047605 0.0467517441420518 0.14193005759432 0.00629637492141923 0.0434701724574783 0.37003367003367

hsa-miR-135aS 1 16.9473684210526 0.0590062111801242 -2.83011256637797 0.0319133649703778 0.114178454116742 0.00720203512774652 0.0460822998357415 0.364646464646465

hsa-miR-524-5p 52.4385964912281 51.8947368421053 1.0104800540906 0.0104255190131926 0.272311224940988 0.475823778012421 0.282113385043078 0.478317978963018 0.573400673400673

hsa-miR-188-3p 151.833333333333 132.947368421053 1.14205595143837 0.132830104287906 0.146683274817219 0.322106020781833 0.6979725666165 0.811792890822156 0.596969696969697

hsa-miR-557 29.8245614035088 31.7543859649123 0.939226519337017 -0.0626985942155637 1 1 0.902735357370599 0.946610708883144 0.4996632996633

hsa-miR-512-3p 9.92982456140352 1 9.92982456140352 2.29554281036863 0.495784208724925 0.670629736880267 0.427326237253781 0.607549493822098 0.542760942760943

hsa-miR-302aS 44.7017543859649 44.421052631579 1.00631911532385 0.00629923342798715 0.753629632470352 0.856893771833878 0.808473914073725 0.877626399805817 0.521212121212121

hsa-miR-30e 267.745614035088 144.28947368421 1.85561432305915 0.618215812694694 0.0205519329468186 0.0856385558862379 0.0372335803785043 0.126506219947438 0.654545454545455

hsa-miR-520a-5p 107.263157894737 96.719298245614 1.10901505532378 0.103472283861097 0.715775731362509 0.830261365814309 0.646247434465097 0.780016134186544 0.524579124579125

hsa-miR-203 8.52631578947369 40.2105263157895 0.212041884816754 -1.55097145393414 0.00648893354480751 0.0399996403512063 0.0118795725660271 0.0625126288078134 0.32020202020202

hsa-miR-516bS 12.4517543859649 46.7719298245614 0.266222805701425 -1.32342170538557 0.000453496780812062 0.00815349420501687 0.00191395692358431 0.0226266414390858 0.267003367003367

hsa-miR-1272 157.342105263158 153.228070175439 1.02684909548889 0.0264949829435626 0.482104605020869 0.658316889450965 0.359263826013872 0.5507010334813 0.547138047138047

hsa-miR-1225-3p 155.53399122807 123.482456140351 1.25956347233075 0.230765210404387 0.0912046033115659 0.228806897261283 0.114908404699974 0.268016089881291 0.612794612794613

hsa-miR-7-1S 289.342105263158 287.973684210526 1.00475189618934 0.00474064157041844 0.304583718725408 0.505552728730713 0.14408361274691 0.305513901229934 0.568686868686869

hsa-miR-1305 116.5 87.9649122807018 1.32439170323095 0.280953262122066 0.00594131812313282 0.0375666422245006 0.00666341858661537 0.0450182786362359 0.683501683501683

hsa-miR-513a-5p 31.0350877192982 52.1929824561403 0.594621848739496 -0.519829623819945 0.0639929306051783 0.177575238303115 0.045061770725276 0.144566201248748 0.376430976430976

hsa-let-7cS 11.8947368421053 12.0701754385965 0.98546511627907 -0.0146415499929481 0.727181719186779 0.837860912761269 0.289861745999606 0.483850458022554 0.477104377104377

hsa-miR-509-5p 272.043859649123 202.464912280702 1.34365928685932 0.295396703161578 0.0145842485324569 0.0676677767930662 0.0651084698823419 0.183622906890396 0.662962962962963

hsa-miR-124S 119.964912280702 87.9649122807018 1.36378141204627 0.310261291481008 0.00214267158401661 0.0192617247604826 0.00139920883919937 0.0195848504702865 0.704713804713805

hsa-miR-627 179.236842105263 163.745614035088 1.09460545347405 0.0903939818768313 0.352088978083135 0.55145696567286 0.216808744353423 0.395572825321362 0.562289562289562

hsa-miR-1285 221.605263157895 202.868421052632 1.09235957971203 0.0883401085707302 0.161262112611222 0.342781288629272 0.107986509996309 0.25743745338899 0.593602693602694

hsa-miR-577 11.8421052631579 21.5043859649123 0.550683255149908 -0.596595489658814 0.440511298310983 0.62219517257345 0.159087406496178 0.324750240730857 0.449158249158249

hsa-miR-1261 1 2.87719298245614 0.347560975609756 -1.05681515998965 0.000266425422519954 0.00589551640089027 0.00129732741952932 0.0193033372940311 0.293602693602694

hsa-miR-625S 221.605263157895 233.517543859649 0.948987641335787 -0.0523595032854714 0.812129272219521 0.896251365649256 0.625775649565218 0.770391420220804 0.483838383838384

hsa-miR-29cS 1 1 1 0 0.0970004910208502 0.237816544747141 0.0207105023157434 0.0922620643667372 0.4003367003367

hsa-miR-147b 32.8245614035088 32.2631578947368 1.01740076128331 0.0172511016697686 0.963632704799025 0.99008546735978 0.284945870267592 0.480643389728918 0.503367003367003

hsa-miR-25 6067.7149122807 6386.57456140351 0.950073447658499 -0.0512159840513353 0.345990219257337 0.548877866211547 0.358367089943015 0.550303912136693 0.437037037037037

hsa-miR-873 72.7061403508771 40.8947368421052 1.77788502788503 0.575424471269218 0.0206577637319039 0.0856385558862379 0.548752205936379 0.708941846890861 0.653872053872054

hsa-miR-1275 66.8070175438596 83.671052631579 0.798448393353252 -0.225084942904372 0.257282547870674 0.46261044860492 0.125427383138047 0.279699823380193 0.424242424242424

hsa-miR-548g 11.8421052631579 47.6491228070176 0.248527245949926 -1.39220279780606 0.0301968826747765 0.110973151787216 0.0646501990681153 0.182928268182897 0.356565656565657

hsa-miR-181a-2S 79.2631578947368 84.3552631578948 0.939635002339728 -0.0622637745075285 0.85949588724615 0.920293216746039 0.982377817990554 0.990062398821924 0.512121212121212

hsa-miR-24 1799.94736842105 1405.51754385965 1.28062959888659 0.247351831131251 0.00754651705367683 0.0446071521734459 0.0144289457009305 0.0732481184700177 0.678114478114478

hsa-miR-16-2S 110.530701754386 80.8771929824561 1.36664859002169 0.312361458141281 0.228731802158309 0.430055654166929 0.373798166367076 0.565748422592332 0.58047138047138

hsa-miR-630 20.280701754386 59.5438596491228 0.340601060695345 -1.07704339656185 0.00932006154157652 0.0518829955910512 0.00490275646477033 0.0363906225345639 0.327609427609428

hsa-miR-491-5p 98.4649122807017 79.0877192982456 1.24500887311446 0.219142656890843 0.436088197623542 0.620006778499369 0.948498030383594 0.977961529535294 0.552188552188552

hsa-miR-618 9.92982456140352 24.2631578947368 0.409255242227043 -0.893416253461575 0.0270247087550646 0.103536028864037 0.090516137367993 0.230428986868962 0.354882154882155

hsa-miR-598 60.2456140350877 67.719298245614 0.889637305699482 -0.116941421001455 0.851522561540645 0.916289240161567 0.676262877436386 0.795115617476296 0.487205387205387

hsa-miR-146a 201.850877192982 119.719298245614 1.6860345838218 0.522379371719113 0.00136887201230625 0.0150361655662008 0.00247448949606373 0.0249896897703841 0.713468013468013

hsa-miR-338-3p 147.004385964912 115.087719298246 1.27732469512195 0.244767808726053 0.0285346330927198 0.106603412809598 0.178265766743591 0.351240540410317 0.646127946127946

hsa-miR-1238 15.7894736842105 16.140350877193 0.978260869565217 -0.0219789067187759 0.934288838832987 0.969200602800134 0.882843781226429 0.934839488587004 0.505723905723906

hsa-miR-96 214.640350877193 171.043859649123 1.25488486589056 0.227043828049098 0.00920266322149107 0.0518829955910512 0.039608626861427 0.13197777985101 0.673737373737374

hsa-miR-542-3p 101.122807017544 74.578947368421 1.35591625499882 0.304477428760623 0.0902437998556217 0.227056557654232 0.730496513634875 0.827691876662466 0.613131313131313

hsa-miR-491-3p 102.5 57.578947368421 1.78016453382084 0.576705794762977 0.00018781223176168 0.00543451823935425 0.000647830252328404 0.013311369232367 0.748821548821549

hsa-miR-938 105.228070175439 96.4210526315789 1.09133915574964 0.0874055254067696 0.344262473286244 0.547142752202631 0.446072741148673 0.623502291404031 0.563299663299663

hsa-miR-101 639.035087719298 615.030701754386 1.03902957347729 0.0382871751165294 0.711928206763787 0.828103268763026 0.655952729880895 0.785141755738159 0.524915824915825

hsa-miR-22 5736.38157894737 6386.57456140351 0.898193785071343 -0.107369437667353 0.0521411398845526 0.152534927865657 0.336573462823081 0.532065857063763 0.370707070707071

hsa-miR-302f 1 1 1 0 0.0819359797677723 0.213185660875067 0.00995298107134603 0.0557754718478677 0.401346801346801

hsa-miR-382 18.6315789473684 41.8771929824561 0.444909928780897 -0.809883424536945 0.0665760088942531 0.181819923024495 0.030446028054995 0.113041939543687 0.37979797979798

hsa-miR-135b 1 24.0175438596491 0.04163623082542 -3.1787845574539 0.000213550675463343 0.00543451823935425 0.000106403566197503 0.00459131388142223 0.266666666666667

hsa-miR-708S 60.2456140350877 72.0526315789473 0.836133430728025 -0.178967072499138 0.715500259903574 0.830261365814309 0.798868965692053 0.872688503028154 0.524579124579125

hsa-miR-196a 1 15.8771929824561 0.0629834254143646 -2.76488367586538 6.31914097315111e-05 0.00287022034727864 5.30283394000705e-05 0.00308503027500963 0.271043771043771

hsa-miR-122S 71.3508771929824 74.0175438596491 0.963972505333017 -0.0366925062171142 0.979823596368348 0.995974060001018 0.954285338009606 0.97901699343718 0.502020202020202

hsa-miR-521 43.0877192982456 74.0175438596491 0.582128466461247 -0.541064122857305 0.0240865676110137 0.0949164741931728 0.0145395748191213 0.073378088122232 0.349494949494949

hsa-miR-125b-2S 26.8245614035088 47.5438596491228 0.564206642066421 -0.572334707944684 0.026539792324375 0.102707806170115 0.00493360699483659 0.0363906225345639 0.353872053872054

hsa-miR-25S 49.0526315789474 74.7894736842105 0.655876143560873 -0.421783313411509 0.0873282753573129 0.222313574139708 0.033441489495081 0.118278710796127 0.385858585858586

hsa-miR-373 1 12.7478070175439 0.0784448649578531 -2.54535925818696 0.00507214110597495 0.0347401410671141 0.00176952460626124 0.0222258115415021 0.330639730639731

hsa-miR-892a 1 12.0701754385965 0.0828488372093023 -2.49073757009879 0.0289338692771789 0.107629005112954 0.00589027343736241 0.0413276908653964 0.360942760942761

hsa-miR-133a 58.3333333333333 87.9122807017544 0.663540211534624 -0.41016582186977 0.00792917562755326 0.0456191904438564 0.00176078567632793 0.0222258115415021 0.322895622895623

hsa-miR-1323 39.2631578947368 36.3859649122807 1.07907425265188 0.0761035000823981 0.768775308241416 0.866126750668853 0.393392778015103 0.583809996845114 0.48013468013468

hsa-miR-549 57.8245614035088 62.3552631578947 0.927340507842724 -0.0754344584864446 0.979823468766671 0.995974060001018 0.869958906403082 0.925739255518939 0.502020202020202

hsa-miR-593 1 4.70175438596492 0.212686567164179 -1.54793571267631 0.191964494069646 0.387110804068896 0.181456757933788 0.356713398853893 0.418855218855219

hsa-miR-33a 125.40350877193 81.4385964912281 1.53985351141749 0.431687289444936 0.00236864708544992 0.0200503012704972 0.0180267087604858 0.0850112003295043 0.702693602693603

hsa-miR-202 12.8377192982456 1 12.8377192982456 2.55238765773744 0.207205313143896 0.405483413249845 0.292343968022585 0.485393049550722 0.578451178451178

hsa-miR-146b-3p 54.4385964912281 23.6491228070176 2.30192878338279 0.833747372976127 0.00372069565474696 0.0274441055559541 0.00523572416499375 0.0382917792744882 0.692929292929293

hsa-miR-154 1 1 1 0 0.0264331031184645 0.102707806170115 0.00549206612539563 0.0398290173631632 0.369023569023569

hsa-miR-10a 42.2105263157895 21.8026315789474 1.93602896801448 0.660638951559842 0.0767735736436738 0.203863366321509 0.447216591401037 0.623502291404031 0.617171717171717

hsa-miR-1913 297.779605263158 308.570175438596 0.965030417602411 -0.0355956573084084 0.816039306685684 0.898267757231818 0.590570981362446 0.740788890865975 0.515824915824916

hsa-miR-505S 171.973684210526 136.97149122807 1.25554363662563 0.227568655383976 0.0452250344319368 0.139889622633554 0.16676999552335 0.334703502643375 0.633670033670034

hsa-miR-127-5p 154.285087719298 117.684210526316 1.31100924269529 0.270797254867533 0.0257199273817541 0.10043573452694 0.0306148043166913 0.113041939543687 0.648821548821549

hsa-miR-548b-5p 1 1 1 0 0.00466482413390768 0.0327296197362791 0.000368502374668066 0.00955580617104973 0.334680134680135

hsa-miR-514 46.8771929824562 34.8421052631579 1.34541792547835 0.296704690051419 0.420295785258053 0.610631755349663 0.43871879396499 0.617936612007514 0.553872053872054

hsa-miR-297 65.0701754385965 74.9824561403508 0.867805334581189 -0.141787858389502 0.190265691802474 0.385444347477783 0.0326812207582435 0.117028603794042 0.412457912457912

hsa-miR-532-3p 2746.45614035088 3004.33333333333 0.914164919677425 -0.0897442865210008 0.641523352338096 0.786412859471274 0.226682245530594 0.405024384871435 0.531313131313131

hsa-miR-33b 193.587719298246 168.885964912281 1.14626292006441 0.136507016118824 0.015852623602534 0.070885047507704 0.0277340521012314 0.107340628595556 0.660942760942761

hsa-miR-942 8.10526315789475 45.561403508772 0.177897574123989 -1.72654732058101 0.0104421640042158 0.0563224220977389 0.00707318930858506 0.0460822998357415 0.331986531986532

hsa-miR-139-3p 94.3508771929824 87.5789473684211 1.07732371794872 0.0744799267795461 0.883398095017399 0.935426449079774 0.623895295049163 0.769173770896325 0.51010101010101

hsa-miR-766 364.991228070175 487.219298245614 0.74913130367463 -0.288841005522515 0.193705273321957 0.387859978832596 0.101565237591469 0.24760113006056 0.413131313131313

hsa-miR-21S 119.40350877193 59.5438596491228 2.00530347672363 0.69579540926668 0.00101148085960934 0.0133096439759987 0.000604419302762425 0.0130403464570993 0.719191919191919

hsa-miR-492 89.7543859649123 70.5087719298245 1.27295347101269 0.241339768249532 0.0473949328637609 0.142515076869079 0.107040860674461 0.255889924548641 0.632323232323232

hsa-miR-1208 107.914473684211 82.0175438596491 1.3157486631016 0.274405829177972 0.0405495316987435 0.131557315248179 0.522641071626514 0.695977639599638 0.636700336700337

hsa-miR-519e 1 1 1 0 0.443032133368908 0.622698259116234 0.103695358398963 0.250669731928025 0.457912457912458

hsa-miR-652 1091.65789473684 1030.54385964912 1.05930270169047 0.0576108630659156 0.124082882967826 0.281798757897983 0.133569667548702 0.291461363564702 0.602693602693603

hsa-miR-425S 70.0526315789473 48.8771929824561 1.4332376166547 0.35993595272347 0.220393679202581 0.422920256809363 0.844346397670047 0.906346385612297 0.581818181818182

hsa-miR-1276 41.8771929824561 46.7719298245614 0.895348837209302 -0.110541874399824 0.100972723129333 0.245463267776379 0.0280507754226852 0.107590307510121 0.390572390572391

hsa-miR-933 203.271929824561 174.039473684211 1.16796451702326 0.155262504683669 0.11002976777481 0.25944177483514 0.307042081321573 0.502803256509521 0.606734006734007

hsa-miR-586 31.0350877192982 39.6666666666667 0.782397169394073 -0.245392778118185 0.935282590960352 0.969200602800134 0.463807154127691 0.643513784585526 0.494276094276094

hsa-miR-27b 217.482456140351 188.745614035088 1.15225170795185 0.141718034892802 0.175335952473533 0.361133477290356 0.148412951733682 0.310874702296523 0.590572390572391

hsa-miR-523 110.530701754386 116.5 0.948761388449665 -0.0525979467083301 0.979826530538886 0.995974060001018 0.938130528802575 0.970592844969367 0.502020202020202

hsa-miR-1280 2746.45614035088 3467.72368421053 0.792005474039419 -0.233186975525663 0.0782757967584587 0.206581078295259 0.496398075869863 0.675696434504245 0.382491582491582

hsa-miR-663 340.5 289.342105263158 1.17680763983629 0.162805382327091 0.148083063011904 0.322689034517631 0.650277851366253 0.78269147242549 0.596632996632997

hsa-miR-345 110.530701754386 84.5855263157895 1.30673303777449 0.267530158059452 0.00817176272234019 0.0467035180753615 0.0344107557497513 0.120717407365998 0.676430976430976

hsa-miR-661 48.6315789473684 45.2631578947369 1.07441860465116 0.0717796823941303 0.386517186739927 0.583152753381532 0.15051994166585 0.313684509358367 0.442087542087542

hsa-miR-519eS 69.9649122807017 35.3508771929824 1.97915632754342 0.682670656700945 0.0183605858144479 0.0795304637135369 0.635019073442051 0.771861211803507 0.656902356902357

hsa-miR-658 42.4561403508772 60.6842105263158 0.699624168834923 -0.357211989786437 0.103328333505939 0.24936882910527 0.0949569857897861 0.23753008329445 0.391245791245791

hsa-miR-185S 1 40.2807017543859 0.0248257839721254 -3.69587248960491 0.0126243936595966 0.06302773945034 0.0233380229996632 0.0979406591869764 0.338383838383838

hsa-miR-1298 50.6754385964912 52.5263157894737 0.964762859051436 -0.0358729497582533 0.536845884105467 0.706796080019112 0.538577373398286 0.704930551107513 0.541414141414141

hsa-miR-548e 43.2478070175439 42.1052631578947 1.02713541666667 0.0267737787941965 0.435768759367754 0.620006778499369 0.476545017200082 0.658013359749874 0.552188552188552

hsa-miR-324-5p 357.271929824561 302.114035087719 1.18257309601928 0.167692654288952 0.00702697565360981 0.0418226206142432 0.00231303474619509 0.0245058837767518 0.67979797979798

hsa-miR-1244 1 1 1 0 0.00632075101884536 0.0392432239515363 0.00667710274094809 0.0450182786362359 0.371380471380471

hsa-miR-376a 103.824561403509 96.3508771929824 1.07756737072105 0.0747060660722936 0.288192332542588 0.489586580677664 0.889917685746834 0.937727671305882 0.571043771043771

hsa-miR-516a-3p 1.19298245614034 16.140350877193 0.0739130434782605 -2.60486596486698 0.033626817281849 0.117165812810002 0.0193732854360891 0.0898878781255103 0.362289562289562

hsa-miR-455-5p 10 28.6842105263158 0.348623853211009 -1.05376172250276 0.0160887913314137 0.0715702418505671 0.00206892191003507 0.0231880468618216 0.342760942760943

hsa-miR-548d-5p 1 14.8070175438597 0.0675355450236967 -2.69510122676141 0.00135605503744662 0.0150361655662008 0.00327470143721695 0.0294382014616482 0.311111111111111

hsa-miR-1229 223.872807017544 198.359649122807 1.12862070490426 0.120996272037874 0.122973701242941 0.280757418446185 0.423238967479815 0.603727651132365 0.603030303030303

hsa-miR-150S 80.1754385964912 89.2280701754386 0.898545025560361 -0.106978462093815 0.581445410546316 0.741244377059759 0.48194831013008 0.662295209621432 0.537037037037037

hsa-let-7f-2S 1 1 1 0 0.603290964655499 0.756744335025721 0.199836725694732 0.374911074509899 0.530639730639731

hsa-miR-606 97.7236842105263 81.4385964912281 1.19996768634209 0.182294628383133 0.067122884394251 0.182735171079617 0.0627012214208086 0.178584666951016 0.622222222222222

**Values cancer vs. pancreatitis in blood**

name median g1 median g2 qmedian logqmedian wmw_rawp wmw_adjp ttest_rawp ttest_adjp AUC

hsa-miR-579 24.0175438596491 30.6666666666667 0.783180778032037 -0.244391730927032 0.873290831374107 0.9974938201385 0.294159364265184 0.996448344010977 0.489506172839506

hsa-miR-663b 74.0175438596491 84.7258771929825 0.87361200983564 -0.135118926563511 0.124609107853166 0.9974938201385 0.129675042026307 0.996448344010977 0.4

hsa-miR-106b 7477.03728070175 8807.11184210526 0.848977214636397 -0.163722930915034 0.326575084704617 0.9974938201385 0.179153243992091 0.996448344010977 0.436111111111111

hsa-miR-449b 71.0701754385965 45.4035087719298 1.56530139103555 0.448078387581899 0.0465310533942592 0.9974938201385 0.164442592647358 0.996448344010977 0.629320987654321

hsa-miR-561 23.6491228070176 24.4473684210526 0.967348403301041 -0.0331965554497503 0.768149654253705 0.9974938201385 0.643894042541405 0.996448344010977 0.480864197530864

hsa-miR-524-3p 15.8771929824561 20.0350877192983 0.79246935201401 -0.23260144651603 0.767183728404438 0.9974938201385 0.960749127978468 0.999654245801439 0.480864197530864

hsa-miR-655 46.9473684210526 44.719298245614 1.04982346018046 0.0486220168720037 0.452192797778955 0.9974938201385 0.776255923676805 0.997772027551652 0.548765432098765

hsa-miR-30c-2S 3.55701754385965 27.4824561403509 0.129428662623683 -2.04462541739672 0.113032728482021 0.9974938201385 0.606501330955182 0.996448344010977 0.400308641975309

hsa-miR-299-5p 31.7543859649123 43.859649122807 0.724 -0.322963886596421 0.667424022739617 0.9974938201385 0.753692364985273 0.997772027551652 0.471913580246914

hsa-miR-369-5p 29.6140350877193 40.8508771929824 0.724930212583208 -0.321679887266529 0.410167792896185 0.9974938201385 0.613568492556049 0.996448344010977 0.446604938271605

hsa-miR-452 28.6842105263158 77.1578947368421 0.371759890859482 -0.989507087783352 0.0388610305511155 0.9974938201385 0.0681623366940995 0.996448344010977 0.366049382716049

hsa-miR-934 91.5614035087719 80.0526315789473 1.14376506684199 0.134325510716011 0.266024857227469 0.9974938201385 0.478728136723137 0.996448344010977 0.572530864197531

hsa-miR-483-5p 159.09649122807 203.149122807017 0.783151258689926 -0.244429423244397 0.894127664653367 0.9974938201385 0.278723050054892 0.996448344010977 0.508950617283951

hsa-miR-340S 20.859649122807 19.9298245614035 1.04665492957746 0.0455992974096851 0.907837561973859 0.9974938201385 0.543378828380642 0.996448344010977 0.507716049382716

hsa-miR-340 152.219298245614 125.526315789474 1.21264849755416 0.192806808540006 0.147207245731811 0.9974938201385 0.373541925541097 0.996448344010977 0.594444444444444

hsa-miR-608 178.5 155.80701754386 1.14564801261119 0.13597042679574 0.905423147930763 0.9974938201385 0.759077951638838 0.997772027551652 0.508024691358025

hsa-miR-1539 84.7456140350878 97.0646929824561 0.873083831320674 -0.135723701039546 0.225549095032945 0.9974938201385 0.316442788176621 0.996448344010977 0.420987654320988

hsa-miR-802 42.0350877192982 49.0526315789474 0.856938483547925 -0.154389144118361 0.381858156980806 0.9974938201385 0.628209513270068 0.996448344010977 0.44320987654321

hsa-miR-654-3p 23.6491228070176 19.4342105263158 1.2168810652223 0.196291081392654 0.55540596881099 0.9974938201385 0.191009095040024 0.996448344010977 0.538271604938272

hsa-miR-507 21.0657894736842 25.3070175438597 0.832409012131716 -0.183431357837321 0.772648223909332 0.9974938201385 0.608418457501164 0.996448344010977 0.481172839506173

hsa-miR-637 40.2807017543859 18.5350877192983 2.17321344060577 0.776206920383345 0.689408530710397 0.9974938201385 0.40300572384108 0.996448344010977 0.525925925925926

hsa-miR-1228 458.607456140351 371.139254385965 1.23567488677289 0.211617287843792 0.475917069856839 0.9974938201385 0.447212256727402 0.996448344010977 0.546604938271605

hsa-miR-626 30.140350877193 35.1315789473684 0.85792759051186 -0.153235576398313 0.592158692848346 0.9974938201385 0.871866558837192 0.999654245801439 0.465123456790123

hsa-miR-151-5p 3921.35964912281 4726.70175438596 0.829618590909428 -0.186789212832098 0.102428104320943 0.9974938201385 0.074733433519356 0.996448344010977 0.393827160493827

hsa-miR-431S 16.8245614035088 16.5438596491228 1.016967126193 0.0168247922499806 0.782523885196493 0.9974938201385 0.24623362676165 0.996448344010977 0.517901234567901

hsa-miR-29aS 4.70175438596492 1 4.70175438596492 1.54793571267631 0.525297727276974 0.9974938201385 0.830139329799445 0.999177463900866 0.53858024691358

hsa-miR-532-5p 128.53399122807 150.811403508772 0.852282972226262 -0.159836680268664 0.735791174619528 0.9974938201385 0.630343877044226 0.996448344010977 0.477777777777778

hsa-miR-27a 357.271929824561 363.69298245614 0.982344854200333 -0.0178128569185501 0.96209129240887 0.999511784184894 0.999079116406291 0.999654245801439 0.503395061728395

hsa-miR-556-5p 69.8245614035087 88.0877192982456 0.792670782712606 -0.232347297756223 0.374021552950694 0.9974938201385 0.49447431143149 0.996448344010977 0.441975308641975

hsa-miR-425 12702.7105263158 12702.7105263158 1 0 0.769895735311891 0.9974938201385 0.73904111638206 0.997772027551652 0.519135802469136

hsa-miR-615-5p 48.8771929824561 33.5964912280702 1.45483028720627 0.374889252725005 0.389370888513177 0.9974938201385 0.380977025191155 0.996448344010977 0.556172839506173

hsa-miR-519c-3p 21.2741228070176 32.4824561403509 0.654941938968404 -0.423208690072219 0.858818436684801 0.9974938201385 0.628857699756013 0.996448344010977 0.511728395061728

hsa-miR-486-3p 274.609649122807 389.40350877193 0.705205892953685 -0.349265472064401 0.0287952216492723 0.9974938201385 0.0316196141204586 0.996448344010977 0.357716049382716

hsa-miR-525-3p 62.563596491228 56.8421052631579 1.10065586419753 0.0959062423033452 0.812120497573049 0.9974938201385 0.417786050030452 0.996448344010977 0.515740740740741

hsa-miR-140-5p 9.92982456140352 29.2982456140351 0.338922155688623 -1.08198482720762 0.40292521697371 0.9974938201385 0.649482454433526 0.996448344010977 0.446913580246914

hsa-miR-20bS 64.4736842105263 77.4298245614035 0.832672482157018 -0.183114892813351 0.392268858237029 0.9974938201385 0.857716476823485 0.999654245801439 0.444135802469136

hsa-miR-526bS 3.71710526315789 10.7456140350877 0.345918367346939 -1.06155246428417 0.791167494080117 0.9974938201385 0.784110319618791 0.997772027551652 0.483333333333333

hsa-miR-30bS 43.0877192982456 56.4210526315789 0.763681592039801 -0.269604341031823 0.939198373390967 0.9974938201385 0.735938019020349 0.997772027551652 0.494753086419753

hsa-miR-136S 56.3508771929825 48.4561403508772 1.16292541636495 0.150938741097415 0.801080828646458 0.9974938201385 0.622707582189457 0.996448344010977 0.516666666666667

hsa-miR-1911 45.2631578947369 28.1315789473684 1.6089803554724 0.475600658782454 0.158205504421571 0.9974938201385 0.27315321460524 0.996448344010977 0.591666666666667

hsa-miR-628-3p 129.587719298246 108.811403508772 1.19093877221976 0.174741880336123 0.864151916451056 0.9974938201385 0.919849740488141 0.999654245801439 0.48858024691358

hsa-miR-629S 118 155.311403508772 0.759763915167604 -0.274747531897082 0.421868682511306 0.9974938201385 0.975463089349114 0.999654245801439 0.447530864197531

hsa-miR-603 150.850877192982 155.620614035088 0.969350224765018 -0.0311293033250558 0.679268532986852 0.9974938201385 0.786175597905236 0.997772027551652 0.47283950617284

hsa-miR-518d-3p 52.9824561403509 49.4736842105263 1.0709219858156 0.0685199464367557 0.400039334757059 0.9974938201385 0.188121520684479 0.996448344010977 0.554938271604938

hsa-miR-23b 3749.71929824561 3467.72368421053 1.08132009344317 0.0781826034686379 0.105786463860924 0.9974938201385 0.115685251257542 0.996448344010977 0.605246913580247

hsa-miR-23aS 1 14.4440789473684 0.0692325210658164 -2.6702845691555 0.652959612267717 0.9974938201385 0.616645373449032 0.996448344010977 0.471913580246914

hsa-miR-939 77.1929824561403 65.1688596491228 1.18450718443988 0.169326809976007 0.637979835814346 0.9974938201385 0.796567192603133 0.997772027551652 0.530864197530864

hsa-miR-662 71.7368421052631 58.4649122807017 1.22700675168792 0.204577668311778 0.558602170137337 0.9974938201385 0.476697217046102 0.996448344010977 0.538271604938272

hsa-miR-1178 34.6842105263158 28.6666666666667 1.20991432068543 0.190549547750669 0.709353912098639 0.9974938201385 0.770286340444493 0.997772027551652 0.524382716049383

hsa-miR-519b-5p 87.9122807017544 85.9934210526316 1.02231402851095 0.0220687131858992 0.9431601557001 0.9974938201385 0.736681718474891 0.997772027551652 0.504938271604938

hsa-miR-409-5p 88.9122807017544 91.1052631578947 0.975929135374543 -0.0243653024032892 0.950737033055902 0.999511784184894 0.88001161466777 0.999654245801439 0.495679012345679

hsa-miR-567 80.8771929824561 59.1666666666666 1.36693847294292 0.312573547908784 0.565151611000337 0.9974938201385 0.533440993744122 0.996448344010977 0.537654320987654

hsa-miR-600 77.1929824561403 80.859649122807 0.954653937947494 -0.0464063728141561 0.920488850345447 0.9974938201385 0.537034436113792 0.996448344010977 0.50679012345679

hsa-miR-1207-5p 334.570175438596 313.923245614035 1.06577063060168 0.0636981343005285 0.272292207163799 0.9974938201385 0.216652163136223 0.996448344010977 0.571604938271605

hsa-miR-1291 144.131578947368 119.982456140351 1.2012721158064 0.183381091793789 0.856687542089099 0.9974938201385 0.670841463251887 0.996448344010977 0.512037037037037

hsa-miR-145 192.657894736842 136.890350877193 1.4073884207491 0.341735803110679 0.276315975701199 0.9974938201385 0.294859259874467 0.996448344010977 0.570987654320988

hsa-miR-636 114.561403508772 129.879934210526 0.882056217576118 -0.12549948625814 0.571700503011472 0.9974938201385 0.414343203947985 0.996448344010977 0.462962962962963

hsa-miR-541 57.1578947368421 56.1151315789474 1.01858256638724 0.0184120200531425 0.826872573054348 0.9974938201385 0.696757080486389 0.996448344010977 0.514506172839506

hsa-miR-367S 67.4736842105263 62.8859649122807 1.0729529920491 0.0704146528557979 0.882823972760658 0.9974938201385 0.737645071256344 0.997772027551652 0.490123456790123

hsa-miR-497 154.285087719298 179.936403508772 0.857442322457983 -0.153801364643931 0.721519844706732 0.9974938201385 0.772751560866451 0.997772027551652 0.476543209876543

hsa-miR-34aS 116.5 119.501096491228 0.974886452264073 -0.0254342739825263 0.686251323710044 0.9974938201385 0.276442125261541 0.996448344010977 0.473456790123457

hsa-miR-126 1907.68201754386 2252.16447368421 0.847043828208147 -0.166002840440784 0.413433602043922 0.9974938201385 0.550975343593974 0.996448344010977 0.446604938271605

hsa-miR-1255a 52.9824561403509 65.9561403508771 0.803298310945605 -0.219029138449166 0.703086228520595 0.9974938201385 0.40535990879371 0.996448344010977 0.475

hsa-miR-519b-3p 39.6666666666667 44.5438596491228 0.8905080740449 -0.115963109426709 0.84841723930359 0.9974938201385 0.688340999021842 0.996448344010977 0.512654320987654

hsa-miR-377S 1 1 1 0 0.805018061355041 0.9974938201385 0.57181139652486 0.996448344010977 0.485802469135802

hsa-miR-187S 191.956140350877 151.996710526316 1.26289667510695 0.233408030922003 0.336983324663528 0.9974938201385 0.464405785729318 0.996448344010977 0.562654320987654

hsa-miR-1538 58.3333333333333 82.3530701754386 0.708332223790376 -0.344842052706543 0.594089233310179 0.9974938201385 0.413691486170182 0.996448344010977 0.465123456790123

hsa-miR-331-5p 55.8070175438597 26.7719298245614 2.08453473132372 0.734545679904741 0.195716351380644 0.9974938201385 0.309704430227927 0.996448344010977 0.584259259259259

hsa-miR-219-1-3p 23.6491228070176 12.4967105263158 1.89242783188559 0.637860571856299 0.937953686751312 0.9974938201385 0.298489499826622 0.996448344010977 0.505246913580247

hsa-miR-493S 25.9298245614035 22.4385964912281 1.15559030492572 0.144611299929305 0.668248000516114 0.9974938201385 0.914746206280482 0.999654245801439 0.527777777777778

hsa-miR-191S 57.3333333333333 42.4561403508772 1.3504132231405 0.300410636829161 0.317296790312841 0.9974938201385 0.17002532045458 0.996448344010977 0.565123456790123

hsa-miR-323-3p 45.7192982456141 32.4736842105263 1.40788762830902 0.342090445111293 0.553840354458191 0.9974938201385 0.574942833338226 0.996448344010977 0.53858024691358

hsa-miR-199a-5p 247.412280701754 179.474780701754 1.37853507737612 0.321021397205206 0.438528044841824 0.9974938201385 0.494856522776691 0.996448344010977 0.550617283950617

hsa-miR-653 25.9298245614035 46.280701754386 0.560272934040941 -0.579331231769112 0.381293352237479 0.9974938201385 0.677775765358653 0.996448344010977 0.442901234567901

hsa-miR-384 106.157894736842 75.4912280701754 1.40622821287474 0.340911093783722 0.111341970693199 0.9974938201385 0.192214228972761 0.996448344010977 0.603703703703704

hsa-miR-125b 521.372807017544 313.675438596491 1.66214099946867 0.508106530060213 0.240454939858661 0.9974938201385 0.63974789727813 0.996448344010977 0.576543209876543

hsa-miR-422a 143.377192982456 130.458333333333 1.09902671082049 0.0944249797853545 0.438546143009626 0.9974938201385 0.477452298118025 0.996448344010977 0.550617283950617

hsa-miR-431 140.868421052632 185.657894736842 0.758752657689582 -0.276079433893534 0.234801542133257 0.9974938201385 0.664548111553167 0.996448344010977 0.422530864197531

hsa-miR-769-3p 18.6315789473684 10.4912280701754 1.77591973244147 0.574318447851253 0.788978879335782 0.9974938201385 0.464886438226987 0.996448344010977 0.516975308641975

hsa-miR-155S 67.719298245614 66.6337719298245 1.01629093302616 0.0161596595692569 0.815645172466001 0.9974938201385 0.650946734803462 0.996448344010977 0.484567901234568

hsa-miR-548n 40.9824561403509 27.2894736842105 1.50176792028287 0.406643027610425 0.299063609453808 0.9974938201385 0.194389112492292 0.996448344010977 0.566975308641975

hsa-miR-9S 103.719298245614 90.4583333333333 1.14659749327256 0.136798855263141 0.472923444580335 0.9974938201385 0.879826623298187 0.999654245801439 0.546913580246914

hsa-miR-1265 41.8771929824561 26.7719298245614 1.56422018348624 0.447387414494427 0.21155290193338 0.9974938201385 0.714717873630122 0.996448344010977 0.580246913580247

hsa-miR-30c 2106.82894736842 2158.04166666667 0.976268892260384 -0.0240172261482551 0.882744343283139 0.9974938201385 0.713164387788576 0.996448344010977 0.490123456790123

hsa-miR-15aS 124.535087719298 120.984649122807 1.02934619079556 0.0289238344742657 0.886617941713494 0.9974938201385 0.46593445245783 0.996448344010977 0.490432098765432

hsa-miR-502-5p 13.4385964912281 15.5350877192982 0.865047995482778 -0.144970287482297 0.876576514943928 0.9974938201385 0.376840486929159 0.996448344010977 0.510185185185185

hsa-miR-1227 58.9824561403509 45.2982456140351 1.30209140201394 0.263971742561973 0.358723707834103 0.9974938201385 0.268924861447623 0.996448344010977 0.559876543209877

hsa-miR-520f 1 1 1 0 0.919824114803094 0.9974938201385 0.934475451231338 0.999654245801439 0.493827160493827

hsa-miR-1910 14.8070175438597 46.7894736842105 0.316460442444694 -1.15055702958606 0.0331071194396746 0.9974938201385 0.528356689044425 0.996448344010977 0.363888888888889

hsa-miR-1182 16.8245614035088 9.44736842105264 1.78087279480037 0.577103578286995 0.414860163860978 0.9974938201385 0.6930058217723 0.996448344010977 0.55216049382716

hsa-miR-622 88.7368421052632 95.5964912280701 0.928243714443018 -0.0744609573531873 0.958308892439427 0.999511784184894 0.72251716923722 0.997772027551652 0.496296296296296

hsa-miR-361-5p 404.478070175439 374.833333333333 1.07908778170415 0.0761160376509898 0.801130320109912 0.9974938201385 0.927060070457354 0.999654245801439 0.483333333333333

hsa-miR-1248 42.1052631578947 38.9035087719298 1.08229988726043 0.0790883021523024 0.577220370091176 0.9974938201385 0.98115888447223 0.999654245801439 0.46358024691358

hsa-miR-129-3p 93.5087719298245 89.3859649122807 1.04612365063788 0.0450915715030647 0.530458205636945 0.9974938201385 0.524123722282428 0.996448344010977 0.458950617283951

hsa-miR-330-3p 264.666666666667 326.377192982456 0.810922675840568 -0.209582573628589 0.262035660937637 0.9974938201385 0.32858167317462 0.996448344010977 0.426851851851852

hsa-miR-298 158.815789473684 93.7478070175439 1.69407471519802 0.527136701041184 0.15250467700705 0.9974938201385 0.28707675215269 0.996448344010977 0.59320987654321

hsa-miR-619 77.7894736842105 75.9824561403508 1.02378203648118 0.02350364896163 0.905422084843617 0.9974938201385 0.688999613211032 0.996448344010977 0.508024691358025

hsa-miR-888 60.8070175438596 68.859649122807 0.883057324840764 -0.124365159940957 0.601125857165384 0.9974938201385 0.763939940734748 0.997772027551652 0.465740740740741

hsa-miR-944 35.3508771929824 36.1666666666667 0.977443609022556 -0.0228146777661716 0.4831299790872 0.9974938201385 0.24681036892257 0.996448344010977 0.454320987654321

hsa-miR-106a 7477.03728070175 7477.03728070175 1 0 0.644095148459748 0.9974938201385 0.999654245801439 0.999654245801439 0.530246913580247

hsa-miR-506 49.6491228070175 59.1666666666666 0.839140103780578 -0.175377597444627 0.699826440839388 0.9974938201385 0.958406705620193 0.999654245801439 0.474691358024691

hsa-miR-200aS 96 73.2543859649123 1.310501736319 0.270410068709102 0.587913027204857 0.9974938201385 0.585981771810456 0.996448344010977 0.535493827160494

hsa-miR-649 24.0175438596491 20.4298245614035 1.17561185057965 0.161788735964053 0.648354926165116 0.9974938201385 0.468075235630152 0.996448344010977 0.52962962962963

hsa-miR-500 148.28947368421 170.461622807018 0.869928792430255 -0.139343918464536 0.939376537502605 0.9974938201385 0.835263809955647 0.999654245801439 0.494753086419753

hsa-miR-505 27.3333333333333 27.7543859649123 0.984829329962073 -0.0152869218969055 0.4910856407128 0.9974938201385 0.365816284143553 0.996448344010977 0.544444444444445

hsa-miR-24-1S 112.078947368421 88.7543859649123 1.26279897212888 0.233330663739441 0.909195118749554 0.9974938201385 0.509785356605269 0.996448344010977 0.492283950617284

hsa-miR-1908 494.798245614035 415.677631578947 1.19034128378414 0.174240059102076 0.41097984015639 0.9974938201385 0.13287726300285 0.996448344010977 0.553703703703704

hsa-miR-518f 74.578947368421 45.8070175438597 1.62811183454615 0.48742095965966 0.143933087653846 0.9974938201385 0.229004950074305 0.996448344010977 0.595061728395062

hsa-miR-548h 16.8245614035088 19.2982456140351 0.871818181818182 -0.137174383903024 0.879812491975414 0.9974938201385 0.597000016526133 0.996448344010977 0.490123456790123

hsa-miR-423-5p 1405.51754385965 1503.35964912281 0.934917698954972 -0.0672967760757097 0.452484949436397 0.9974938201385 0.777092998261221 0.997772027551652 0.450925925925926

hsa-miR-1185 10.8771929824561 14.6293859649123 0.743516714135812 -0.296364032990542 0.494683884181864 0.9974938201385 0.473250763046369 0.996448344010977 0.456481481481481

hsa-miR-1302 27.3333333333333 34.8508771929824 0.784293984394664 -0.242971348823959 0.462657155811487 0.9974938201385 0.687328270750121 0.996448344010977 0.452469135802469

hsa-miR-18b 232.09649122807 196.771929824561 1.17952032810271 0.165107854225525 0.565230700631256 0.9974938201385 0.576129741399298 0.996448344010977 0.537654320987654

hsa-miR-29b-1S 12.2456140350877 10.8070175438597 1.13311688311688 0.124972139228852 0.875256361878112 0.9974938201385 0.508194781402521 0.996448344010977 0.510185185185185

hsa-miR-937 69.9649122807017 88.6710526315789 0.789038927635158 -0.236939621412708 0.499737806633731 0.9974938201385 0.993628283274813 0.999654245801439 0.455864197530864

hsa-miR-573 47.4035087719298 39.7894736842105 1.19135802469136 0.175093853672502 0.418380964792426 0.9974938201385 0.417624350504152 0.996448344010977 0.552469135802469

hsa-miR-339-3p 265.793859649123 283.394736842105 0.937892716748692 -0.0641197109855161 0.524221959831188 0.9974938201385 0.819849225813878 0.997772027551652 0.458333333333333

hsa-miR-199b-5p 14.8070175438597 19.6381578947368 0.753992183137912 -0.282373278219019 0.782854261551483 0.9974938201385 0.937319131668525 0.999654245801439 0.482407407407407

hsa-miR-93 3312.48684210526 3154.75 1.04999979145899 0.0487899655589306 0.890302859533686 0.9974938201385 0.754358698329014 0.997772027551652 0.509259259259259

hsa-miR-148aS 42.4561403508772 30.4824561403509 1.39280575539568 0.331320241711785 0.260224719044139 0.9974938201385 0.678430489908308 0.996448344010977 0.573148148148148

hsa-miR-554 100.175438596491 100.822368421053 0.993583469276781 -0.00643720514263011 0.746578348656376 0.9974938201385 0.484663579748302 0.996448344010977 0.521296296296296

hsa-miR-539 21.1842105263158 18.3684210526316 1.15329512893983 0.142623174656191 0.75278361134614 0.9974938201385 0.73237664092029 0.997772027551652 0.520061728395062

hsa-miR-1252 17.6491228070176 28.0263157894737 0.629733959311424 -0.462457835592005 0.265167868125117 0.9974938201385 0.128206447886974 0.996448344010977 0.428395061728395

hsa-miR-664S 69.9649122807017 68.6140350877193 1.01968805931987 0.0194967563262147 0.746567294279853 0.9974938201385 0.685766240314792 0.996448344010977 0.521296296296296

hsa-miR-520e 1 3.68092105263158 0.271671134941912 -1.3031630069057 0.332963475175415 0.9974938201385 0.723786127162422 0.997772027551652 0.441358024691358

hsa-miR-329 58.7017543859649 64.9473684210526 0.903835764451648 -0.101107611586673 0.75349190802015 0.9974938201385 0.3706836722399 0.996448344010977 0.520679012345679

hsa-miR-452S 306.69298245614 263.642543859649 1.163290939187 0.1512530049226 0.890378595078406 0.9974938201385 0.857079072421366 0.999654245801439 0.509259259259259

hsa-miR-1468 1 1 1 0 0.712728161270698 0.9974938201385 0.488622084233802 0.996448344010977 0.477777777777778

hsa-miR-519c-5p 127.548245614035 102.064692982456 1.24968039276775 0.222887832835223 0.358946392652767 0.9974938201385 0.423164903091332 0.996448344010977 0.559876543209877

hsa-miR-210 372.427631578947 425.348684210526 0.875581952886951 -0.132866524684546 0.227368544303333 0.9974938201385 0.985441218013711 0.999654245801439 0.421296296296296

hsa-let-7gS 154.285087719298 137.804824561404 1.11959133659033 0.112963740678467 0.693247532732775 0.9974938201385 0.518609349099706 0.996448344010977 0.525925925925926

hsa-let-7eS 1 10.4188596491228 0.0959797937276363 -2.34361759167254 0.639328329041504 0.9974938201385 0.783270948497269 0.997772027551652 0.471296296296296

hsa-miR-300 26.4561403508772 26.1315789473684 1.01242027526015 0.0123437764145648 0.456731712147868 0.9974938201385 0.461441657622585 0.996448344010977 0.452160493827161

hsa-miR-1268 559.473684210526 542.842105263158 1.03063796781074 0.0301779967250894 0.511887097782237 0.9974938201385 0.510025291425006 0.996448344010977 0.542901234567901

hsa-miR-484 5736.38157894737 5736.38157894737 1 0 0.988612360361303 1 0.739382334227572 0.997772027551652 0.501234567901235

hsa-miR-522 9.36842105263158 5.81578947368421 1.61085972850679 0.476768029334277 0.740750875723621 0.9974938201385 0.964274445152254 0.999654245801439 0.520987654320988

hsa-miR-429 57.9649122807017 57.125 1.01470305961841 0.01459601759195 0.358261333859428 0.9974938201385 0.0932642699840776 0.996448344010977 0.440123456790123

hsa-miR-497S 96.719298245614 131.995614035088 0.732746303372653 -0.310955744272298 0.162315817452235 0.9974938201385 0.0971577164249839 0.996448344010977 0.408950617283951

hsa-miR-146b-5p 154.741228070175 156.763157894737 0.987102008841139 -0.0129818924665158 0.562056227224605 0.9974938201385 0.807233066021167 0.997772027551652 0.462037037037037

hsa-miR-1273 129.587719298246 142.487938596491 0.909464482219949 -0.0948993337711719 0.679271953162456 0.9974938201385 0.42528924253827 0.996448344010977 0.472839506172839

hsa-miR-107 1799.94736842105 1956.55592105263 0.919957027066559 -0.0834283197402451 0.284633818755087 0.9974938201385 0.45655527607014 0.996448344010977 0.430246913580247

hsa-miR-92a-1S 25.2982456140351 42.140350877193 0.600333055786845 -0.510270684795034 0.331557883924883 0.9974938201385 0.305756465467556 0.996448344010977 0.437037037037037

hsa-miR-376aS 1.57894736842105 37.640350877193 0.04194826380797 -3.17131823410132 0.0953364741938159 0.9974938201385 0.432826963794939 0.996448344010977 0.394444444444444

hsa-miR-133b 30.140350877193 35.7543859649123 0.842983316977429 -0.170808111238469 0.543545123817648 0.9974938201385 0.888039407687465 0.999654245801439 0.460493827160494

hsa-miR-599 51.8947368421053 49.8245614035088 1.04154929577465 0.0407093121154934 0.996200348665423 1 0.888353526171192 0.999654245801439 0.499382716049383

hsa-miR-378 168.885964912281 135.798245614035 1.24365351075512 0.21805342718887 0.0853686866555081 0.9974938201385 0.0303725376672055 0.996448344010977 0.612037037037037

hsa-miR-34c-3p 95.578947368421 92.5438596491228 1.03279620853081 0.0322698894850807 0.838080067690245 0.9974938201385 0.803401420662264 0.997772027551652 0.51358024691358

hsa-miR-642 51.1578947368421 32.1578947368421 1.59083469721768 0.464258845288844 0.46936147203621 0.9974938201385 0.497274661195681 0.996448344010977 0.547222222222222

hsa-miR-582-3p 70.5087719298245 65.9561403508771 1.06902513632132 0.0667471456311199 0.617785613966353 0.9974938201385 0.607659126368053 0.996448344010977 0.532716049382716

hsa-miR-34a 43.2478070175439 47.8947368421053 0.902976190476191 -0.102059093048772 0.882822000436933 0.9974938201385 0.633974104041327 0.996448344010977 0.509876543209877

hsa-miR-337-5p 36.280701754386 33.2105263157895 1.09244585314316 0.088419084418997 0.749248990115956 0.9974938201385 0.589718396668866 0.996448344010977 0.520987654320988

hsa-miR-616 31.4385964912281 11.3070175438596 2.78044996121024 1.02261277115563 0.0347333544790938 0.9974938201385 0.0286149765931872 0.996448344010977 0.634259259259259

hsa-miR-1304 62.1951754385965 77.7719298245614 0.799712384389804 -0.223503135469589 0.274329911628207 0.9974938201385 0.414306226077348 0.996448344010977 0.428703703703704

hsa-miR-760 69.6842105263157 62.5021929824561 1.11490824883337 0.108772113484418 0.166006901081332 0.9974938201385 0.0520652778376775 0.996448344010977 0.590123456790123

hsa-miR-1226S 142.28947368421 160.421052631579 0.886975065616797 -0.119938407984478 0.268116619663502 0.9974938201385 0.230423067182621 0.996448344010977 0.427777777777778

hsa-miR-7-2S 40.2105263157895 25.859649122807 1.55495251017639 0.441445005085347 0.479005452763802 0.9974938201385 0.505416790624878 0.996448344010977 0.545987654320988

hsa-miR-518cS 58.3333333333333 48.0350877192983 1.21439006574142 0.19424194724156 0.739312342755835 0.9974938201385 0.912848235431215 0.999654245801439 0.521913580246914

hsa-miR-132 103.982456140351 129.103618421053 0.805418604157377 -0.216393131554963 0.194473787875937 0.9974938201385 0.215289622582582 0.996448344010977 0.415432098765432

hsa-miR-767-3p 23.6491228070176 34.140350877193 0.692702980472765 -0.367153971273697 0.900670186984575 0.9974938201385 0.469965908783512 0.996448344010977 0.491666666666667

hsa-miR-1281 123.763157894737 148.129385964912 0.835507128369877 -0.179716399122071 0.496773859877187 0.9974938201385 0.729903067938916 0.997772027551652 0.455555555555556

hsa-miR-1914S 117.684210526316 154.381030701754 0.762297090460988 -0.271418916785186 0.0888770875977023 0.9974938201385 0.124403932878487 0.996448344010977 0.389197530864198

hsa-miR-550 98.5701754385965 120.703947368421 0.816627604876365 -0.202572096019037 0.184856034147142 0.9974938201385 0.223198497479477 0.996448344010977 0.41358024691358

hsa-miR-330-5p 28.2280701754386 24.2719298245614 1.16299241055294 0.150996347765402 0.618870984339623 0.9974938201385 0.59157369120232 0.996448344010977 0.532407407407407

hsa-miR-616S 55.8070175438597 59.4473684210526 0.938763464659879 -0.0631917328270233 0.522930631998451 0.9974938201385 0.56327638860303 0.996448344010977 0.458333333333333

hsa-miR-765 60.0350877192982 61.7478070175438 0.972262670028767 -0.0281292743739355 0.524121192746143 0.9974938201385 0.130827263297298 0.996448344010977 0.541666666666667

hsa-miR-520g 45.7192982456141 58.0087719298245 0.788144563738092 -0.238073749429926 0.173807462836062 0.9974938201385 0.1145475401755 0.996448344010977 0.41141975308642

hsa-miR-28-3p 124.986842105263 105.105263157895 1.18915873810716 0.173246114354477 0.600893494248501 0.9974938201385 0.695595053671019 0.996448344010977 0.534259259259259

hsa-miR-296-3p 97.2675438596491 111.710526315789 0.87071063996859 -0.138445573205474 0.651633777681577 0.9974938201385 0.45931831008188 0.996448344010977 0.47037037037037

hsa-miR-1224-3p 169.236842105263 160.973684210526 1.05133235246036 0.0500582668922983 0.742980866014077 0.9974938201385 0.911190567912776 0.999654245801439 0.521604938271605

hsa-miR-590-3p 8.52631578947369 19.1754385964912 0.444647758462946 -0.810472864276044 0.353831109019492 0.9974938201385 0.542674446412651 0.996448344010977 0.441358024691358

hsa-miR-455-3p 114.561403508772 136.434210526316 0.839682386601087 -0.17473156983602 0.384389325512283 0.9974938201385 0.864875773058635 0.999654245801439 0.44320987654321

hsa-miR-485-5p 32.5263157894737 50.3859649122807 0.645543175487465 -0.437663184042314 0.0840139487815353 0.9974938201385 0.238796339460954 0.996448344010977 0.387654320987654

hsa-let-7a 1135.39035087719 1031.42763157895 1.1007949720516 0.0960326206461747 0.819482390677467 0.9974938201385 0.697808507503063 0.996448344010977 0.484876543209876

hsa-miR-1253 83.671052631579 81.3771929824561 1.0281879918077 0.0277980217223008 0.696677492356638 0.9974938201385 0.804466074165007 0.997772027551652 0.525617283950617

hsa-miR-490-3p 90.125 91.3552631578947 0.986533198905372 -0.0135583008618295 0.689742752258282 0.9974938201385 0.701250826245482 0.996448344010977 0.473765432098765

hsa-miR-194S 83.3026315789474 98.1260964912281 0.848934529729247 -0.163773210215241 0.221871079688705 0.9974938201385 0.225591189841786 0.996448344010977 0.42037037037037

hsa-miR-1259 40.8947368421052 33.3421052631579 1.22651933701657 0.204180350606454 0.74884957748745 0.9974938201385 0.658576420841825 0.996448344010977 0.520987654320988

hsa-miR-1249 144.131578947368 150.210526315789 0.959530483531885 -0.0413111938130058 0.779181754932062 0.9974938201385 0.597962670177318 0.996448344010977 0.481481481481481

hsa-miR-651 20.280701754386 25.6140350877193 0.791780821917808 -0.23347066547006 0.62056730970209 0.9974938201385 0.660666457357506 0.996448344010977 0.46820987654321

hsa-miR-182 3467.72368421053 4503.74342105263 0.769964751544402 -0.261410542397278 0.0253829619219631 0.9974938201385 0.0173451755209439 0.996448344010977 0.35462962962963

hsa-miR-1324 140.868421052632 128.621710526316 1.09521495613923 0.090950650979235 0.988624840111095 1 0.350454385280533 0.996448344010977 0.498765432098765

hsa-miR-15bS 74.578947368421 83.1425438596491 0.897001028670904 -0.108698270133693 0.886399168542461 0.9974938201385 0.945944685411277 0.999654245801439 0.509567901234568

hsa-miR-543 46.2105263157895 42.859649122807 1.07818256242325 0.0752768110454863 0.571102118263617 0.9974938201385 0.493585640973812 0.996448344010977 0.537037037037037

hsa-miR-1287 73.719298245614 63.4736842105263 1.16141514648977 0.149639215449705 0.467075806221502 0.9974938201385 0.558918704231943 0.996448344010977 0.547530864197531

hsa-miR-220c 119.964912280702 85.2478070175439 1.40724924754971 0.341636910666324 0.0933995736684735 0.9974938201385 0.0958799076054254 0.996448344010977 0.609259259259259

hsa-miR-611 96.1403508771929 105.690789473684 0.909637929245772 -0.094708638596172 0.935567235648248 0.9974938201385 0.535662700328095 0.996448344010977 0.494444444444444

hsa-miR-1825 58.8421052631579 62.3508771929824 0.943725379853686 -0.0579200663314422 0.9545142608262 0.999511784184894 0.890823856045106 0.999654245801439 0.504012345679012

hsa-miR-320b 1212.00438596491 1198.42543859649 1.01133065681944 0.0112669457347266 0.707281288186806 0.9974938201385 0.612821063178507 0.996448344010977 0.524691358024691

hsa-miR-612 82.0175438596491 69.9298245614035 1.17285499247366 0.159440940950048 0.924277488984327 0.9974938201385 0.84545113518994 0.999654245801439 0.506481481481481

hsa-miR-671-5p 67.719298245614 66.1578947368421 1.02360116679926 0.0233269652005194 0.800828398475474 0.9974938201385 0.608943526721913 0.996448344010977 0.516666666666667

hsa-miR-145S 42.6666666666667 29.2280701754386 1.45978391356543 0.378288420359269 0.510137632630028 0.9974938201385 0.519048763234343 0.996448344010977 0.542592592592593

hsa-miR-657 58.3333333333333 64.7127192982456 0.901419905791454 -0.103784085774996 0.841602806725857 0.9974938201385 0.783564108439668 0.997772027551652 0.486728395061728

hsa-miR-1826 158.043859649123 119.044956140351 1.32759811732631 0.283371382759565 0.35407255794156 0.9974938201385 0.672610603048518 0.996448344010977 0.560493827160494

hsa-miR-99a 119.40350877193 84.8947368421053 1.40648894399669 0.341096488267533 0.475932016140459 0.9974938201385 0.528909348058244 0.996448344010977 0.546604938271605

hsa-miR-604 67.298245614035 53.3421052631579 1.26163459957244 0.232408181443164 0.6241760473786 0.9974938201385 0.895598906418556 0.999654245801439 0.532098765432099

hsa-miR-101S 122.078947368421 110.666666666667 1.10312301838935 0.0981452647769788 0.77918054049487 0.9974938201385 0.358430922195295 0.996448344010977 0.518518518518518

hsa-miR-504 24.0175438596491 39.9473684210526 0.60122968818621 -0.50877824077529 0.155868717245889 0.9974938201385 0.142186704402459 0.996448344010977 0.407716049382716

hsa-miR-424 246.535087719298 284.157894736842 0.867598938074952 -0.142025724010167 0.400233650065594 0.9974938201385 0.553377334979736 0.996448344010977 0.445061728395062

hsa-miR-767-5p 149.728070175438 119.698464912281 1.25087711262767 0.223844995346951 0.542974291155897 0.9974938201385 0.363337363974639 0.996448344010977 0.539814814814815

hsa-miR-222S 47.7543859649123 47.1754385964912 1.01227222015619 0.0121975269425468 1 1 0.802228551923758 0.997772027551652 0.500308641975309

hsa-miR-875-5p 40.8947368421052 59.1666666666666 0.691178650852483 -0.369356949046154 0.501457167707231 0.9974938201385 0.584916011330635 0.996448344010977 0.456172839506173

hsa-miR-876-3p 17.0175438596491 38.5438596491228 0.441511151570324 -0.817552000887181 0.107634373713363 0.9974938201385 0.236167698475406 0.996448344010977 0.395987654320988

hsa-miR-361-3p 157.131578947368 167.842105263158 0.936186892442772 -0.0659401510230109 1 1 0.817113362184342 0.997772027551652 0.499691358024691

hsa-miR-371-5p 20.280701754386 39.280701754386 0.516301920500223 -0.661063567366432 0.426904075106524 0.9974938201385 0.441500896669719 0.996448344010977 0.448765432098765

hsa-miR-29b-2S 57.9649122807017 62.8782894736842 0.921858924056152 -0.0813630779179726 0.771694677385172 0.9974938201385 0.912395658886585 0.999654245801439 0.519135802469136

hsa-miR-556-3p 2.87719298245614 18.9035087719298 0.152204176334107 -1.8825323941537 0.109727567338075 0.9974938201385 0.333869918158302 0.996448344010977 0.398765432098765

hsa-miR-1263 74.578947368421 87.4122807017544 0.853186151530356 -0.15877752372816 0.384375898528863 0.9974938201385 0.318236922869199 0.996448344010977 0.44320987654321

hsa-miR-1224-5p 36.280701754386 17.6491228070176 2.05566600397614 0.720599884968634 0.82031542834871 0.9974938201385 0.848373973700392 0.999654245801439 0.514814814814815

hsa-miR-346 59.438596491228 82.9298245614035 0.716733657711022 -0.333050975021291 0.0502207524820785 0.9974938201385 0.0170407065982233 0.996448344010977 0.372530864197531

hsa-miR-1234 529.405701754386 577.889802631579 0.916101477035229 -0.0876281376544159 0.819550524058938 0.9974938201385 0.495325794288481 0.996448344010977 0.515123456790123

hsa-miR-154S 57.9649122807017 50.6414473684211 1.14461405218144 0.135067507853939 0.460443864474563 0.9974938201385 0.673025360956397 0.996448344010977 0.548148148148148

hsa-let-7b 587.213815789474 412.790570175439 1.42254658467587 0.352448634936166 0.197757467057516 0.9974938201385 0.436077289215335 0.996448344010977 0.583950617283951

hsa-miR-26a 7079.20175438596 6690.78070175438 1.05805317345549 0.0564305906377376 0.0917219346224608 0.9974938201385 0.0464493384061353 0.996448344010977 0.609567901234568

hsa-miR-631 153.754385964912 140.959429824561 1.09077048734005 0.0868843156460572 0.549313339720218 0.9974938201385 0.652463605406306 0.996448344010977 0.539197530864198

hsa-miR-379 1 4.24561403508773 0.235537190082644 -1.44588645832214 0.529554361969922 0.9974938201385 0.880541131875247 0.999654245801439 0.462037037037037

hsa-miR-129-5p 44.421052631579 60.8344298245614 0.730195922928571 -0.314442393275818 0.661142486746982 0.9974938201385 0.97752923064096 0.999654245801439 0.471296296296296

hsa-miR-374bS 58.7017543859649 45.4035087719299 1.29289026275116 0.256880225925977 0.293542459948012 0.9974938201385 0.166035452604978 0.996448344010977 0.568518518518519

hsa-miR-615-3p 41.8771929824561 25.3508771929825 1.65190311418685 0.501928025792297 0.230774105835826 0.9974938201385 0.179157475284976 0.996448344010977 0.577777777777778

hsa-miR-200a 84.5855263157895 71.0087719298245 1.19119827053737 0.174959750520733 0.916716072627104 0.9974938201385 0.33196010517623 0.996448344010977 0.492901234567901

hsa-let-7aS 1 18.9002192982456 0.0529094389975054 -2.93917352508006 0.579188557492816 0.9974938201385 0.615597088887148 0.996448344010977 0.465432098765432

hsa-miR-635 101.333333333333 96.7642543859649 1.04721866536731 0.046137759525861 0.502789014745869 0.9974938201385 0.536635581458772 0.996448344010977 0.456172839506173

hsa-miR-1251 129.394736842105 148.129385964912 0.873525101040758 -0.13521841365518 0.107135823274266 0.9974938201385 0.180759048042267 0.996448344010977 0.395061728395062

hsa-miR-1225-5p 74.5614035087719 74.7807017543859 0.997067448680352 -0.0029368596733096 0.396634100965239 0.9974938201385 0.535547241761042 0.996448344010977 0.444753086419753

hsa-miR-19b 9361.25657894737 9651.24561403509 0.969953201204826 -0.0305074548291848 0.588187960501974 0.9974938201385 0.607587935736742 0.996448344010977 0.535185185185185

hsa-miR-1184 168.885964912281 156.059210526316 1.08219158832539 0.0789882334420802 0.311396374556155 0.9974938201385 0.687236893230596 0.996448344010977 0.566049382716049

hsa-miR-30c-1S 35.4385964912281 8.42105263157896 4.20833333333333 1.43706668649331 0.210006820419214 0.9974938201385 0.453556859519123 0.996448344010977 0.57962962962963

hsa-miR-216b 144.131578947368 148.765350877193 0.968851806536256 -0.0316436132181416 0.710867170826276 0.9974938201385 0.952513772729519 0.999654245801439 0.475617283950617

hsa-miR-182S 39.6666666666667 29.8859649122807 1.32726739066628 0.283122235176948 0.434301174709514 0.9974938201385 0.289428377808971 0.996448344010977 0.550925925925926

hsa-miR-32S 106.964912280702 65.469298245614 1.63381791384739 0.490919554394468 0.0824761420172063 0.9974938201385 0.0292152878703107 0.996448344010977 0.612654320987654

hsa-miR-376b 45.561403508772 52.3859649122807 0.86972538513061 -0.139577766436386 0.34064364325918 0.9974938201385 0.20825329531128 0.996448344010977 0.562037037037037

hsa-let-7bS 2.98245614035088 17.8245614035088 0.167322834645669 -1.78783019108817 0.748221971192383 0.9974938201385 0.618371564470805 0.996448344010977 0.47962962962963

hsa-miR-483-3p 31.7543859649123 25.7280701754386 1.23423116263212 0.210448235842365 0.576743861422153 0.9974938201385 0.77570506065201 0.997772027551652 0.535802469135803

hsa-miR-574-3p 1533.93421052632 1907.68201754386 0.804082754054187 -0.218053087171793 0.962082304048495 0.999511784184894 0.930348347220938 0.999654245801439 0.503395061728395

hsa-miR-541S 1 1 1 0 0.593925245066602 0.9974938201385 0.913942940879703 0.999654245801439 0.469135802469136

hsa-miR-1254 141.09649122807 140.322368421053 1.00551674558895 0.00550158408388509 0.402924880920507 0.9974938201385 0.521873648965739 0.996448344010977 0.44537037037037

hsa-miR-302bS 20.280701754386 14.6228070175439 1.3869226154769 0.327087347041474 0.691887535742226 0.9974938201385 0.465472526015959 0.996448344010977 0.525617283950617

hsa-miR-217 132.947368421053 113.400219298246 1.17237311571151 0.159029998605251 0.155316495259619 0.9974938201385 0.521377815590622 0.996448344010977 0.592592592592593

hsa-miR-1271 208.837719298246 228.264254385965 0.91489453686046 -0.0889464806113827 0.530446598968614 0.9974938201385 0.551968064997944 0.996448344010977 0.458950617283951

hsa-miR-503 249.100877192982 240.30701754386 1.03659426902719 0.0359405981330606 0.574945013956432 0.9974938201385 0.512574092091863 0.996448344010977 0.536728395061728

hsa-miR-770-5p 64.2807017543859 67.6578947368421 0.95008427330481 -0.0512045895800332 0.679220639052497 0.9974938201385 0.367077925943049 0.996448344010977 0.52716049382716

hsa-miR-320c 302.114035087719 275.491228070175 1.09663758517481 0.0922487576798771 0.562060642935518 0.9974938201385 0.497141754930214 0.996448344010977 0.537962962962963

hsa-miR-621 266.921052631579 224.06798245614 1.19125030584781 0.175003432731518 0.611099679788192 0.9974938201385 0.934532593529871 0.999654245801439 0.533333333333333

hsa-miR-411 20.5614035087719 52.078947368421 0.394812194711134 -0.929345083573771 0.0645753101259839 0.9974938201385 0.178824821911441 0.996448344010977 0.380864197530864

hsa-miR-432S 52.3508771929824 63.8245614035087 0.820230896096757 -0.198169397754416 0.481301253087142 0.9974938201385 0.469930303476821 0.996448344010977 0.454012345679012

hsa-miR-1258 1 1 1 0 0.494359452193166 0.9974938201385 0.881350408168381 0.999654245801439 0.540123456790123

hsa-miR-29a 494.798245614035 536.515350877193 0.922244339896424 -0.0809450798010367 0.558779954467203 0.9974938201385 0.35566434606287 0.996448344010977 0.461728395061728

hsa-miR-548a-5p 17.0175438596491 11.3070175438596 1.50504266873545 0.408821249118187 0.793254740163869 0.9974938201385 0.569394774655827 0.996448344010977 0.516975308641975

hsa-miR-135a 1 10.7543859649123 0.0929853181076672 -2.37531366810166 0.863892596182546 0.9974938201385 0.85136995007697 0.999654245801439 0.489197530864198

hsa-miR-196b 3.08771929824561 1 3.08771929824561 1.1274327272036 0.500369721284582 0.9974938201385 0.483885957886534 0.996448344010977 0.541358024691358

hsa-miR-769-5p 1 2.35855263157895 0.423988842398884 -0.858048139193398 0.552365163510224 0.9974938201385 0.461064403742422 0.996448344010977 0.463888888888889

hsa-miR-1243 37.7543859649123 52.6666666666667 0.716855429713524 -0.332881090911732 0.329654064208756 0.9974938201385 0.345647378957909 0.996448344010977 0.436728395061728

hsa-miR-103-as 162.061403508772 164.530701754386 0.984991869485245 -0.0151218921738159 0.641376526810372 0.9974938201385 0.905535195496969 0.999654245801439 0.469444444444444

hsa-miR-141 41.8771929824561 34.4473684210526 1.2156862745098 0.195308752320766 0.90896144968633 0.9974938201385 0.619866692855197 0.996448344010977 0.507716049382716

hsa-miR-193b 84.7456140350878 100.048245614035 0.847047477094385 -0.165998532660883 0.282811420704625 0.9974938201385 0.484956807027189 0.996448344010977 0.429938271604938

hsa-miR-548c-3p 45.2631578947369 40.3157894736842 1.12271540469974 0.115750219506963 0.90121057431284 0.9974938201385 0.81425845495444 0.997772027551652 0.508333333333333

hsa-miR-520c-3p 1 1 1 0 0.695386675153665 0.9974938201385 0.309205307752194 0.996448344010977 0.523456790123457

hsa-miR-1180 59.5438596491228 72.844298245614 0.817412770569287 -0.201611084571402 0.707248162601509 0.9974938201385 0.351575502933489 0.996448344010977 0.475308641975309

hsa-miR-526a 100.912280701754 81.5888157894737 1.2368396296147 0.212559440393553 0.935550509011079 0.9974938201385 0.802564492442832 0.997772027551652 0.505555555555556

hsa-miR-383 67.8070175438596 57.4572368421052 1.18013015018797 0.165624729164801 0.815295790452736 0.9974938201385 0.683576593683379 0.996448344010977 0.515432098765432

hsa-miR-1284 42.1754385964912 42.0877192982456 1.00208420175073 0.00208203281540359 0.823042592477688 0.9974938201385 0.563437916432285 0.996448344010977 0.485185185185185

hsa-miR-197 462.921052631579 370.885964912281 1.24814928691374 0.221661883717698 0.110296502178909 0.9974938201385 0.109291755354303 0.996448344010977 0.604012345679012

hsa-miR-614 37.7543859649123 32.9298245614035 1.14651038891849 0.136722884695166 0.863536253017669 0.9974938201385 0.631368876408516 0.996448344010977 0.51141975308642

hsa-miR-518a-5p 169.236842105263 135.633771929825 1.24774854888519 0.221340766381682 0.0585605414740449 0.9974938201385 0.467533625639225 0.996448344010977 0.623148148148148

hsa-miR-186S 125.649122807018 108.881578947368 1.15399798590131 0.143232422764895 0.761004133105192 0.9974938201385 0.930656658026106 0.999654245801439 0.520061728395062

hsa-miR-887 111.622807017544 125.497807017544 0.889440299159487 -0.117162891431146 0.455549716831233 0.9974938201385 0.337117264657885 0.996448344010977 0.451234567901235

hsa-miR-508-5p 111.622807017544 113.361842105263 0.984659431644517 -0.0154594522725951 0.614433672627871 0.9974938201385 0.48943932663861 0.996448344010977 0.466975308641975

hsa-miR-499-3p 119.964912280702 129.570175438597 0.925868255365242 -0.0770233272814083 0.823253005676711 0.9974938201385 0.455437144988863 0.996448344010977 0.514814814814815

hsa-miR-508-3p 32.2631578947368 11.9473684210526 2.70044052863436 0.993414918454808 0.864073901795037 0.9974938201385 0.893660744515768 0.999654245801439 0.511111111111111

hsa-miR-124 107.508771929825 114.618421052632 0.937971147591168 -0.0640360899466837 0.943169127732347 0.9974938201385 0.590300478482835 0.996448344010977 0.504938271604938

hsa-miR-1290 1 1 1 0 0.615693020748356 0.9974938201385 0.984521849312537 0.999654245801439 0.470679012345679

hsa-miR-592 51.8947368421053 49.6666666666667 1.04486047333098 0.0438833581588456 0.988538646448777 1 0.356482820183084 0.996448344010977 0.498765432098765

hsa-miR-1307 77.1929824561403 69.438596491228 1.11167256189995 0.105865693743842 0.297840126660894 0.9974938201385 0.349999863490196 0.996448344010977 0.567901234567901

hsa-miR-1911S 79.859649122807 103.241228070175 0.773524788648625 -0.25679756210227 0.737201877209221 0.9974938201385 0.676050070532759 0.996448344010977 0.477777777777778

hsa-miR-489 172.923245614035 130.644736842105 1.32361432839829 0.280366122330309 0.381880091842633 0.9974938201385 0.224274947493242 0.996448344010977 0.557098765432099

hsa-miR-302e 15.9649122807018 7.49122807017545 2.1311475409836 0.75666058628227 0.646156135822574 0.9974938201385 0.608837903902909 0.996448344010977 0.52962962962963

hsa-miR-1278 45.561403508772 34.8508771929824 1.3073244399698 0.267982636408096 0.671353709811704 0.9974938201385 0.542397329995087 0.996448344010977 0.527777777777778

hsa-miR-1267 44.421052631579 41.6666666666667 1.0661052631579 0.064012066795326 0.92415528366532 0.9974938201385 0.55496602610946 0.996448344010977 0.506481481481482

hsa-miR-570 80.8771929824561 80.7543859649123 1.00152074733869 0.00151959217345284 0.7315639815712 0.9974938201385 0.595051282745099 0.996448344010977 0.477469135802469

hsa-miR-1247 69.6842105263157 74.5964912280701 0.93414863593603 -0.0681197142967755 0.860424368402982 0.9974938201385 0.798776810880774 0.997772027551652 0.488271604938272

hsa-miR-432 11.4561403508772 1 11.4561403508772 2.43852586144188 0.377127200202059 0.9974938201385 0.48643892943316 0.996448344010977 0.55462962962963

hsa-miR-375 4.35087719298246 34.9912280701754 0.124341940335924 -2.08471992518048 0.0533570734261958 0.9974938201385 0.112423660891011 0.996448344010977 0.377777777777778

hsa-miR-758 56.3508771929825 49.5263157894737 1.137796670209 0.129093646813163 0.969644238916701 1 0.657295436499531 0.996448344010977 0.497222222222222

hsa-miR-518e 77.1929824561403 80.6929824561403 0.956625720186977 -0.0443430610108596 0.912953983993864 0.9974938201385 0.650573485304638 0.996448344010977 0.507407407407407

hsa-miR-620 14.8070175438597 17.0438596491228 0.868759650025733 -0.140688774221538 0.833416060858782 0.9974938201385 0.706679339300779 0.996448344010977 0.48641975308642

hsa-miR-17S 498.324561403509 550.65076754386 0.904973879590238 -0.0998491980299652 0.472980936605253 0.9974938201385 0.706563557447743 0.996448344010977 0.453086419753086

hsa-miR-100 156.28947368421 113.285087719298 1.37961206395912 0.321802346576322 0.419143148042814 0.9974938201385 0.980051578466588 0.999654245801439 0.552777777777778

hsa-miR-495 84.0921052631579 91.9320175438596 0.914720545788507 -0.0891366747938937 0.546150659653489 0.9974938201385 0.410202636325403 0.996448344010977 0.460493827160494

hsa-miR-196aS 129.587719298246 162.460526315789 0.797656650738372 -0.226077036354299 0.15393488128314 0.9974938201385 0.648994942985775 0.996448344010977 0.407098765432099

hsa-miR-877 83.671052631579 112.881578947368 0.741228581419746 -0.299446224305557 0.924263418510935 0.9974938201385 0.634713914426115 0.996448344010977 0.506481481481481

hsa-miR-509-3p 16.8245614035088 1 16.8245614035088 2.82283980704889 0.0990554077617794 0.9974938201385 0.0370799116908524 0.996448344010977 0.599691358024691

hsa-miR-875-3p 43.2478070175439 38.8070175438596 1.11443264014467 0.108345432488008 0.563776085107331 0.9974938201385 0.520938662630703 0.996448344010977 0.537654320987654

hsa-miR-1269 45.2631578947369 40.5 1.11760883690708 0.11119143596862 0.394736003734336 0.9974938201385 0.284154503675296 0.996448344010977 0.555555555555556

hsa-miR-500S 209.482456140351 203.816885964912 1.02779735422125 0.0274180213571207 0.508864670556211 0.9974938201385 0.610257502368936 0.996448344010977 0.54320987654321

hsa-miR-144S 587.213815789474 574.457236842105 1.02220631603058 0.0219633461928416 0.984831892650737 1 0.938694786084459 0.999654245801439 0.498456790123457

hsa-miR-181c 83.671052631579 109.706140350877 0.762683404629593 -0.270912268761671 0.578161571319881 0.9974938201385 0.670014234554503 0.996448344010977 0.46358024691358

hsa-miR-378S 46.9473684210526 42.780701754386 1.09739594012713 0.0929400460980118 0.916676673757756 0.9974938201385 0.465295039788101 0.996448344010977 0.492901234567901

hsa-miR-1470 173.675438596491 215.59649122807 0.805557815932948 -0.216220302487844 0.16961411863004 0.9974938201385 0.214888174701171 0.996448344010977 0.410493827160494

hsa-miR-449a 1 1 1 0 0.588120465828667 0.9974938201385 0.928529591972147 0.999654245801439 0.532098765432099

hsa-miR-1255b 70.5263157894737 78.5964912280701 0.897321428571428 -0.108341143795964 0.571461448294035 0.9974938201385 0.937574366988952 0.999654245801439 0.462962962962963

hsa-miR-450a 32.8245614035088 35.7105263157895 0.919184475558831 -0.0842684416689438 0.858351185783527 0.9974938201385 0.642195882384319 0.996448344010977 0.511728395061728

hsa-miR-324-3p 491.008771929825 436.161184210526 1.12575073093351 0.118450129485151 0.562025313528504 0.9974938201385 0.47445451379979 0.996448344010977 0.537962962962963

hsa-miR-208b 45.4035087719299 71.0701754385965 0.638854603801531 -0.448078387581898 0.0387589460054352 0.9974938201385 0.0661684067204808 0.996448344010977 0.365740740740741

hsa-miR-93S 1030.54385964912 1014.75438596491 1.0155598969589 0.0154400830255313 0.700220375234427 0.9974938201385 0.46153710817451 0.996448344010977 0.525308641975309

hsa-miR-542-5p 74.5964912280701 98.1271929824561 0.760202029231663 -0.274171053091347 0.438471153707104 0.9974938201385 0.419659380641633 0.996448344010977 0.449382716049383

hsa-miR-144 2637.20614035088 2295.07456140351 1.14907209756974 0.138954745000509 0.604161815740229 0.9974938201385 0.823416618480414 0.997772027551652 0.533950617283951

hsa-miR-593S 278.521929824561 265.18201754386 1.05030473937961 0.0490803500420153 0.746553474275732 0.9974938201385 0.902924881145125 0.999654245801439 0.478703703703704

hsa-miR-520c-5p 125.298245614035 128.03125 0.978653614754484 -0.021577514427301 0.775469468758082 0.9974938201385 0.619512175508317 0.996448344010977 0.518827160493827

hsa-miR-143S 105.017543859649 144.847587719298 0.725021006654 -0.321554649852048 0.0702020782387641 0.9974938201385 0.334195955205367 0.996448344010977 0.382098765432099

hsa-miR-212 18.4912280701754 35.1666666666667 0.525816911948117 -0.642802202981253 0.203322208125281 0.9974938201385 0.281892165010846 0.996448344010977 0.417901234567901

hsa-miR-16-1S 101.631578947368 99.1600877192982 1.0249242541522 0.0246187114716528 0.779156247543636 0.9974938201385 0.980551439450997 0.999654245801439 0.481481481481481

hsa-miR-338-5p 58.3333333333333 52.8947368421053 1.1028192371476 0.0978698439286685 0.927938140455465 0.9974938201385 0.82039582955092 0.997772027551652 0.506172839506173

hsa-miR-885-5p 67.4736842105263 52.6842105263158 1.28071928071928 0.247421858165394 0.617624745701609 0.9974938201385 0.884513283535125 0.999654245801439 0.532716049382716

hsa-miR-127-3p 40.5263157894737 32.8070175438597 1.23529411764706 0.211309093667206 0.595068728710655 0.9974938201385 0.474913283665188 0.996448344010977 0.534567901234568

hsa-miR-1303 58.9824561403509 41.2982456140351 1.42820730671198 0.356420026148339 0.0527992865864201 0.9974938201385 0.425284535856075 0.996448344010977 0.625925925925926

hsa-miR-548m 1 1.51754385964912 0.658959537572255 -0.417093146103283 0.995862345515363 1 0.54125211626247 0.996448344010977 0.500617283950617

hsa-miR-650 137.675438596491 159.701754385965 0.862078435680545 -0.14840901978918 0.141968144937994 0.9974938201385 0.0948479403611516 0.996448344010977 0.404320987654321

hsa-miR-33aS 82.7719298245614 81.0526315789474 1.02121212121212 0.0209902758918358 0.672325039867855 0.9974938201385 0.428543130879717 0.996448344010977 0.527777777777778

hsa-miR-575 129.201754385965 149.982456140351 0.861445783132531 -0.149143158096635 0.634609892682421 0.9974938201385 0.414160932515516 0.996448344010977 0.468827160493827

hsa-miR-607 83.671052631579 81.4407894736842 1.02738508764844 0.0270168242955429 0.804755301482369 0.9974938201385 0.810048105456588 0.997772027551652 0.516358024691358

hsa-miR-548j 1.3859649122807 1 1.3859649122807 0.326396584632467 0.775447918745148 0.9974938201385 0.298609178423167 0.996448344010977 0.482407407407407

hsa-miR-509-3-5p 201.236842105263 186.719298245614 1.07775063421967 0.0748761231113451 0.397590289519215 0.9974938201385 0.8172206490209 0.997772027551652 0.555246913580247

hsa-miR-1204 1 14.9649122807018 0.0668229777256741 -2.70570827965713 0.189004292043558 0.9974938201385 0.527002248789266 0.996448344010977 0.41820987654321

hsa-miR-1237 101.333333333333 122.756578947368 0.825481894349465 -0.191787948812861 0.717955244925883 0.9974938201385 0.613389892522398 0.996448344010977 0.476234567901235

hsa-miR-27aS 42.0350877192982 59.359649122807 0.708142456036649 -0.34510999643694 0.0679286510968746 0.9974938201385 0.0565456320997495 0.996448344010977 0.381172839506173

hsa-miR-363S 42.0350877192982 41.1929824561403 1.02044293015332 0.0202367782873531 0.786224540025582 0.9974938201385 0.777586094333344 0.997772027551652 0.517901234567901

hsa-miR-1294 17.0175438596491 11.8157894736842 1.44023756495917 0.36480807564701 0.628426151078873 0.9974938201385 0.655796645953146 0.996448344010977 0.530555555555556

hsa-miR-935 12.7478070175439 34.359649122807 0.371010977789124 -0.991523627084371 0.0492651970408522 0.9974938201385 0.0396211174460708 0.996448344010977 0.373456790123457

hsa-miR-1202 237.026315789474 237.40350877193 0.998411173514632 -0.00159009000869182 0.458448315142806 0.9974938201385 0.868759309931472 0.999654245801439 0.451543209876543

hsa-miR-876-5p 21.0657894736842 28 0.75234962406015 -0.28455413746482 0.533130603363026 0.9974938201385 0.535234996162434 0.996448344010977 0.459567901234568

hsa-miR-588 125.649122807018 109.495614035088 1.14752653715201 0.137608788743559 0.481838992273743 0.9974938201385 0.994992291080669 0.999654245801439 0.545987654320988

hsa-miR-1181 211.342105263158 195.736842105263 1.07972573272385 0.0767070577034469 0.879120841399474 0.9974938201385 0.596170330074558 0.996448344010977 0.489814814814815

hsa-miR-412 46.9473684210526 55.6491228070175 0.843631778058008 -0.170039161506619 0.156672361972501 0.9974938201385 0.163403761833599 0.996448344010977 0.407716049382716

hsa-miR-22S 58.8421052631579 88.3728070175439 0.665839495756613 -0.406706634848722 0.0538819068156925 0.9974938201385 0.0386751061571413 0.996448344010977 0.374691358024691

hsa-miR-381 96.719298245614 93.7543859649123 1.03162425149701 0.0311345033732017 0.725084303842047 0.9974938201385 0.346634818583337 0.996448344010977 0.523148148148148

hsa-miR-18a 840 828.118421052631 1.01434768101435 0.0142457270836044 0.77185647569593 0.9974938201385 0.567526922169301 0.996448344010977 0.519135802469136

hsa-miR-634 109.074561403509 153.979714912281 0.708369680159796 -0.344789174441434 0.280629909725867 0.9974938201385 0.934285532244629 0.999654245801439 0.42962962962963

hsa-miR-152 191.517543859649 199.894736842105 0.958091978234158 -0.0428114949371696 0.867891042363036 0.9974938201385 0.987173451155007 0.999654245801439 0.488888888888889

hsa-miR-92bS 136.513157894737 155.035087719298 0.880530723096074 -0.127230458870886 0.946947279329249 0.999040956064965 0.820330372896155 0.997772027551652 0.50462962962963

hsa-miR-891b 81.0350877192982 77.3728070175439 1.04733291763505 0.0462468542540902 0.931809903354538 0.9974938201385 0.476227860710998 0.996448344010977 0.494135802469136

hsa-miR-501-3p 238.921052631579 305.342105263158 0.782470050848918 -0.245299630975788 0.097189194316102 0.9974938201385 0.250283342577534 0.996448344010977 0.391975308641975

hsa-miR-1295 87.9122807017544 100.482456140351 0.874901789611523 -0.133643639367926 0.149760768404214 0.9974938201385 0.106414276324604 0.996448344010977 0.406172839506173

hsa-miR-363 3312.48684210526 3681.29166666667 0.89981646173248 -0.105564467863019 0.226638000564388 0.9974938201385 0.450126096961519 0.996448344010977 0.421296296296296

hsa-miR-1236 30.2456140350877 43.3245614035088 0.698117027738408 -0.359368528757041 0.965625200396681 0.999511784184894 0.934635878795873 0.999654245801439 0.496913580246914

hsa-miR-103 7079.20175438596 8274.35964912281 0.855558865529425 -0.156000379683438 0.115031317695277 0.9974938201385 0.0873267977448766 0.996448344010977 0.397530864197531

hsa-miR-643 52.5263157894737 64.8793859649122 0.809599459185398 -0.211215648451384 0.180148144954513 0.9974938201385 0.536926073893936 0.996448344010977 0.412654320987654

hsa-miR-609 1 1 1 0 0.541915869841821 0.9974938201385 0.38992232690019 0.996448344010977 0.536111111111111

hsa-miR-1264 23.7192982456141 29.7105263157895 0.798346619427222 -0.225212415654916 0.873031081539068 0.9974938201385 0.705186093601423 0.996448344010977 0.489506172839506

hsa-miR-138 57.4736842105263 51.9649122807017 1.10600945307225 0.100758450145216 0.502612850197388 0.9974938201385 0.357810980356603 0.996448344010977 0.543827160493827

hsa-miR-877S 105.684210526316 87.0877192982456 1.21353746978243 0.193539623155232 0.782765217601162 0.9974938201385 0.807767387268288 0.997772027551652 0.51820987654321

hsa-miR-646 214.271929824561 211.429824561403 1.01344231008588 0.0133527638156126 0.830654825961165 0.9974938201385 0.993433941294348 0.999654245801439 0.514197530864198

hsa-miR-585 1 2.35855263157895 0.423988842398884 -0.858048139193398 0.739160126822083 0.9974938201385 0.602501259988524 0.996448344010977 0.47962962962963

hsa-miR-106aS 142.69298245614 136.495614035088 1.0454034253398 0.044402863874253 0.610863943365427 0.9974938201385 0.426692620419334 0.996448344010977 0.533333333333333

hsa-miR-27bS 29.1578947368421 29.8070175438597 0.978222483814008 -0.0220181462565388 0.767222957526296 0.9974938201385 0.553947293046605 0.996448344010977 0.519444444444444

hsa-miR-106bS 161.657894736842 179.155701754386 0.902331844054103 -0.102772928512996 0.574906150105809 0.9974938201385 0.582990299712395 0.996448344010977 0.463271604938272

hsa-miR-220b 57.9649122807017 30.0175438596491 1.93103448275862 0.658055860748675 0.110644852226804 0.9974938201385 0.0956577571553629 0.996448344010977 0.603703703703704

hsa-miR-569 3.94736842105263 23.2982456140351 0.169427710843373 -1.77532892783196 0.0666249160802213 0.9974938201385 0.644871114527209 0.996448344010977 0.383950617283951

hsa-miR-302d 12.0701754385965 16.4561403508772 0.733475479744136 -0.309961111072882 0.817648172804439 0.9974938201385 0.609566518804933 0.996448344010977 0.485185185185185

hsa-let-7g 433.879385964912 500.404605263158 0.867057140165085 -0.142650398761873 0.879117454559077 0.9974938201385 0.616209806878673 0.996448344010977 0.489814814814815

hsa-miR-559 51.1578947368421 51.9649122807017 0.984469952734639 -0.0156519016991952 0.714237565102416 0.9974938201385 0.405026464137232 0.996448344010977 0.475925925925926

hsa-miR-206 28.0701754385965 9.37719298245615 2.9934518241347 1.09642717776277 0.116643031406306 0.9974938201385 0.0718139189604084 0.996448344010977 0.600308641975309

hsa-miR-30eS 69.8245614035087 50.9824561403509 1.36958017894012 0.314504254148254 0.675512419241399 0.9974938201385 0.90244947809672 0.999654245801439 0.527469135802469

hsa-miR-193a-5p 59.438596491228 75.5021929824561 0.787243312323913 -0.239217914021001 0.597389396539799 0.9974938201385 0.775737480852663 0.997772027551652 0.465432098765432

hsa-miR-602 115.087719298246 131.90350877193 0.872514464321341 -0.13637604702424 0.4300251370631 0.9974938201385 0.297169453274744 0.996448344010977 0.448456790123457

hsa-miR-487a 71.0701754385965 74.5964912280701 0.952728127939793 -0.0484256962591478 0.775496622641343 0.9974938201385 0.95170534085269 0.999654245801439 0.481172839506173

hsa-miR-92b 225.798245614035 210.377192982456 1.07330192219489 0.0707398054241594 0.916728724685164 0.9974938201385 0.860836230542721 0.999654245801439 0.507098765432099

hsa-miR-142-5p 693.083333333333 729.178728070176 0.950498563181662 -0.0507686286995494 0.464167043243 0.9974938201385 0.602775495841171 0.996448344010977 0.452160493827161

hsa-miR-191 11503.9078947368 11503.9078947368 1 0 0.240374597472792 0.9974938201385 0.352317450385859 0.996448344010977 0.424074074074074

hsa-miR-1207-3p 24.0175438596491 23.4035087719298 1.02623688155922 0.0258985988128795 0.889678106423359 0.9974938201385 0.567334377553519 0.996448344010977 0.490740740740741

hsa-miR-125b-1S 14.0701754385965 1 14.0701754385965 2.64405734003196 0.463765383099838 0.9974938201385 0.427332011149425 0.996448344010977 0.54537037037037

hsa-miR-188-5p 127.828947368421 97.4736842105263 1.31142008639309 0.271110585485526 0.0328518134332901 0.9974938201385 0.0173472098713006 0.996448344010977 0.638888888888889

hsa-miR-1260 2523.52192982456 2580.36403508772 0.977971284481484 -0.0222749708492514 0.973443663212823 1 0.977557201256683 0.999654245801439 0.497530864197531

hsa-miR-581 20.859649122807 23.3070175438597 0.89499435453519 -0.110937868509086 0.748721154266667 0.9974938201385 0.791266969592501 0.997772027551652 0.520679012345679

hsa-miR-130aS 99.859649122807 104.410087719298 0.956417634580244 -0.0445606050691635 0.958301364566186 0.999511784184894 0.571748093994055 0.996448344010977 0.496296296296296

hsa-miR-132S 8.10526315789475 1 8.10526315789475 2.09251362324719 0.260644459313938 0.9974938201385 0.173841720689287 0.996448344010977 0.568827160493827

hsa-miR-339-5p 443.978070175439 534.070175438597 0.831310360685894 -0.184752075289398 0.220129982481613 0.9974938201385 0.198559768813557 0.996448344010977 0.420061728395062

hsa-miR-129S 57.1578947368421 52.6491228070175 1.08563812062646 0.0821679437216231 0.651129332005841 0.9974938201385 0.964755842953054 0.999654245801439 0.52962962962963

hsa-miR-320d 296.605263157895 284.921052631579 1.04100858963702 0.0401900409311802 0.339451590623426 0.9974938201385 0.230655706275755 0.996448344010977 0.562345679012346

hsa-miR-1322 125.649122807018 94.0723684210526 1.33566449868289 0.289428919837257 0.371569272845725 0.9974938201385 0.542085873653859 0.996448344010977 0.558333333333333

hsa-miR-548l 1 1 1 0 0.936786866977111 0.9974938201385 0.585703658453093 0.996448344010977 0.49537037037037

hsa-miR-886-5p 67.719298245614 66.7105263157894 1.01512163050625 0.0150084383254286 0.651609893459375 0.9974938201385 0.37899002172202 0.996448344010977 0.52962962962963

hsa-miR-98 77.7894736842105 51.8771929824562 1.49949272911735 0.405126870323779 0.129787949584654 0.9974938201385 0.106038364302362 0.996448344010977 0.597839506172839

hsa-miR-15a 3612.86403508772 3681.29166666667 0.9814120591969 -0.0187628676527961 0.699929917967969 0.9974938201385 0.670221185032488 0.996448344010977 0.474691358024691

hsa-miR-597 88.9122807017544 72.423245614035 1.22767600302801 0.20512295373034 0.199239299090701 0.9974938201385 0.249201163287937 0.996448344010977 0.583641975308642

hsa-miR-26a-2S 1 1 1 0 0.347296036118729 0.9974938201385 0.316508867578117 0.996448344010977 0.55462962962963

hsa-miR-184 46.2105263157895 23.1052631578948 2 0.693147180559945 0.326816169767001 0.9974938201385 0.216927958962 0.996448344010977 0.56358024691358

hsa-miR-186 35.3508771929824 35.8245614035088 0.986777668952008 -0.0133105243438277 0.628656224245281 0.9974938201385 0.637100694828648 0.996448344010977 0.531481481481481

hsa-miR-518d-5p 110.530701754386 113.631578947368 0.972711131696773 -0.027668125050942 0.558853228303754 0.9974938201385 0.239425422140535 0.996448344010977 0.538271604938272

hsa-miR-28-5p 342.780701754386 405.839912280702 0.844620480593095 -0.168867887909261 0.349141747000427 0.9974938201385 0.631961094406533 0.996448344010977 0.438888888888889

hsa-miR-15b 14207.2368421053 14207.2368421053 1 0 0.508135922580998 0.9974938201385 0.894304436806878 0.999654245801439 0.542901234567901

hsa-miR-874 182.214912280702 182.438596491228 0.998773920569286 -0.00122683168104147 0.490758779524237 0.9974938201385 0.682898942741785 0.996448344010977 0.545061728395062

hsa-miR-130a 982.480263157895 1135.39035087719 0.865323773800648 -0.144651537113041 0.169462298719337 0.9974938201385 0.390225487435387 0.996448344010977 0.410493827160494

hsa-miR-421 168.885964912281 143.90350877193 1.17360560804633 0.160080726334134 0.183299058933184 0.9974938201385 0.87411987146182 0.999654245801439 0.586728395061728

hsa-miR-18bS 83.3026315789474 82.2478070175439 1.01282495667244 0.0127434133684451 0.661961696077139 0.9974938201385 0.500730723383656 0.996448344010977 0.528703703703704

hsa-miR-34bS 42.4561403508772 67.6578947368421 0.627511992739531 -0.465992496223322 0.416209662132256 0.9974938201385 0.454762569336212 0.996448344010977 0.446913580246914

hsa-miR-219-2-3p 8.52631578947369 11.2456140350877 0.758190327613105 -0.276820833020175 0.738860882114266 0.9974938201385 0.574276774856666 0.996448344010977 0.479012345679012

hsa-miR-1250 106.157894736842 86.0526315789474 1.23363914373089 0.209968454622858 0.505835246008017 0.9974938201385 0.306842139860003 0.996448344010977 0.543518518518519

hsa-miR-194 4503.74342105263 4726.70175438596 0.952830039863114 -0.0483187334540862 0.757036460040442 0.9974938201385 0.574965762305846 0.996448344010977 0.47962962962963

hsa-miR-583 45.8771929824562 73.6052631578947 0.623286854963652 -0.4727484248111 0.327144924490936 0.9974938201385 0.14721290837796 0.996448344010977 0.436111111111111

hsa-miR-568 68.4561403508772 44.280701754386 1.5459587955626 0.435644297521952 0.341426013436768 0.9974938201385 0.937188304248924 0.999654245801439 0.562037037037037

hsa-miR-633 76.6315789473684 106.353070175439 0.720539414809163 -0.327755160237754 0.244302638165244 0.9974938201385 0.292436565357981 0.996448344010977 0.424074074074074

hsa-miR-409-3p 69.8245614035087 71.438596491228 0.977406679764244 -0.0228524599518753 0.767988228801666 0.9974938201385 0.546275290239056 0.996448344010977 0.480555555555556

hsa-miR-26bS 12.0701754385965 6.7280701754386 1.79400260756193 0.584449217126032 0.61447500094939 0.9974938201385 0.859820524420696 0.999654245801439 0.53179012345679

hsa-miR-924 20.5614035087719 28.0263157894737 0.733646322378717 -0.309728216114732 0.246544331944528 0.9974938201385 0.581291069557627 0.996448344010977 0.425

hsa-miR-610 32.5964912280702 32.359649122807 1.00731905665492 0.00729240233705341 0.377799570940672 0.9974938201385 0.509402989569635 0.996448344010977 0.557407407407407

hsa-miR-448 118 118 1 0 0.746556238457335 0.9974938201385 0.515442999297032 0.996448344010977 0.521296296296296

hsa-miR-517a 45.2631578947369 33.280701754386 1.36004217185029 0.307515707980649 0.243334321189024 0.9974938201385 0.273175522293199 0.996448344010977 0.575925925925926

hsa-miR-125a-3p 36.3859649122807 11.219298245614 3.24315871774824 1.17654776777057 0.177354895221386 0.9974938201385 0.859344545756426 0.999654245801439 0.587037037037037

hsa-miR-100S 30.2456140350877 47.5087719298245 0.636632200886263 -0.451563182808527 0.97333948736544 1 0.926372631304602 0.999654245801439 0.497530864197531

hsa-miR-199b-3p 121.90350877193 107.967105263158 1.12908008855849 0.121403220258735 0.204479627159465 0.9974938201385 0.256336813198784 0.996448344010977 0.582716049382716

hsa-miR-10aS 38 48.5263157894737 0.783080260303687 -0.244520084663768 0.0877790102747365 0.9974938201385 0.316092319324311 0.996448344010977 0.388888888888889

hsa-miR-30dS 62.1951754385965 52.9824561403509 1.17388245033113 0.160316588906549 0.797432103185615 0.9974938201385 0.443198434204746 0.996448344010977 0.483024691358025

hsa-miR-518c 87.9122807017544 97.8662280701754 0.898290272704865 -0.107262019365257 0.297992933611373 0.9974938201385 0.395131690621176 0.996448344010977 0.432098765432099

hsa-miR-377 154.741228070175 109.407894736842 1.41435157346161 0.346671174095088 0.206164598981625 0.9974938201385 0.24224129167873 0.996448344010977 0.582407407407407

hsa-miR-373S 20.280701754386 24.2894736842105 0.834958468761285 -0.180373293378695 0.517596679040915 0.9974938201385 0.780180929195696 0.997772027551652 0.458641975308642

hsa-miR-513b 25.859649122807 30.7368421052632 0.841324200913243 -0.172778198747054 0.992339651237695 1 0.438472130056474 0.996448344010977 0.499074074074074

hsa-miR-1827 29.1578947368421 26.4122807017544 1.10395217535702 0.0988966274936048 0.35850229315299 0.9974938201385 0.101800552430775 0.996448344010977 0.441049382716049

hsa-miR-199a-3p 139.09649122807 138.199561403509 1.00649010615846 0.00646913610250954 0.924278767729443 0.9974938201385 0.933158674260626 0.999654245801439 0.493518518518519

hsa-miR-576-3p 5.40350877192984 16.8245614035088 0.32116788321168 -1.13579129190986 0.531267579577487 0.9974938201385 0.456994558067235 0.996448344010977 0.460185185185185

hsa-miR-302c 25.2982456140351 31.5175438596491 0.802671861953799 -0.219809288708679 0.938686860759662 0.9974938201385 0.604335843166901 0.996448344010977 0.494753086419753

hsa-miR-302b 4.70175438596492 1 4.70175438596492 1.54793571267631 0.795028206908612 0.9974938201385 0.452984153998189 0.996448344010977 0.516049382716049

hsa-miR-433 97.828947368421 60.1578947368421 1.62620297462817 0.486247833979554 0.0197953338280325 0.9974938201385 0.070985817908995 0.996448344010977 0.651543209876543

hsa-miR-1233 83.671052631579 100.24451754386 0.834669612679523 -0.180719305868368 0.435750091917654 0.9974938201385 0.615663894119839 0.996448344010977 0.449074074074074

hsa-miR-545S 20.5614035087719 10.1140350877193 2.03295750216826 0.709491630427844 0.743132671799492 0.9974938201385 0.450858344253626 0.996448344010977 0.520987654320988

hsa-miR-550S 334.570175438596 313.923245614035 1.06577063060168 0.0636981343005285 0.977251735311444 1 0.691213192337146 0.996448344010977 0.50216049382716

hsa-miR-33bS 135.688596491228 154.053179824561 0.880790624677484 -0.126935337729448 0.432920280677131 0.9974938201385 0.42119521788895 0.996448344010977 0.448765432098765

hsa-miR-920 58.6140350877193 44.6666666666667 1.31225451688924 0.271746663239404 0.939197683782781 0.9974938201385 0.93062559191947 0.999654245801439 0.494753086419753

hsa-miR-520h 22.5964912280702 27.7368421052632 0.814674256799494 -0.204966930545173 0.294318645983076 0.9974938201385 0.555017970870207 0.996448344010977 0.432716049382716

hsa-miR-148bS 24.5087719298246 32.3508771929825 0.757592190889371 -0.277610044859577 0.5659593564815 0.9974938201385 0.859164296053212 0.999654245801439 0.462962962962963

hsa-miR-31 164.324561403509 158.776315789474 1.03494378608326 0.0343471122792936 0.689649709096419 0.9974938201385 0.368288804214556 0.996448344010977 0.473765432098765

hsa-miR-1299 24.0175438596491 40.9298245614035 0.586798114016288 -0.533074446732981 0.897008152134286 0.9974938201385 0.723617861970243 0.997772027551652 0.491358024691358

hsa-miR-96S 158.815789473684 136.592105263158 1.16270108852712 0.150745822898331 0.682756573809079 0.9974938201385 0.96998314083857 0.999654245801439 0.526851851851852

hsa-miR-200bS 58.9824561403509 90.5087719298245 0.651676681527428 -0.428206727276089 0.467113540794991 0.9974938201385 0.443404246593064 0.996448344010977 0.452469135802469

hsa-miR-517b 30.5789473684211 25.3508771929825 1.20622837370242 0.187498444973488 0.769653682018159 0.9974938201385 0.470682594216663 0.996448344010977 0.480864197530864

hsa-miR-941 115.688596491228 141.267543859649 0.818932596479245 -0.199753498297022 0.631197812075933 0.9974938201385 0.820508405381716 0.997772027551652 0.468518518518519

hsa-miR-1203 168.885964912281 167.684210526316 1.00716677129107 0.00714121203128663 0.909188992598071 0.9974938201385 0.63842090168747 0.996448344010977 0.492283950617284

hsa-miR-648 27.3333333333333 39.0526315789473 0.699910152740342 -0.356803305404796 0.268996623669416 0.9974938201385 0.37954423847942 0.996448344010977 0.428086419753086

hsa-miR-517c 15.0877192982456 20.3157894736842 0.7426597582038 -0.297517268885387 0.891067711752933 0.9974938201385 0.928214344319285 0.999654245801439 0.491049382716049

hsa-let-7f 479.741228070175 521.265350877193 0.920339760283049 -0.0830123724597423 0.76098843050404 0.9974938201385 0.907230112027596 0.999654245801439 0.479938271604938

hsa-miR-450b-5p 56.3508771929825 57.8421052631579 0.974218986957841 -0.0261191680050784 0.749876718154755 0.9974938201385 0.978814780518664 0.999654245801439 0.479012345679012

hsa-miR-423-3p 1030.54385964912 897.320175438596 1.14846839272884 0.138429222288423 0.236573061161155 0.9974938201385 0.249338608751873 0.996448344010977 0.577160493827161

hsa-miR-499-5p 18.4912280701754 19.0964912280702 0.968305006890216 -0.0322081515732947 0.946415225189564 0.999040956064965 0.905635115352399 0.999654245801439 0.50462962962963

hsa-let-7c 280.184210526316 395.916666666667 0.707684808738327 -0.3457564698107 0.728560421730617 0.9974938201385 0.686099882680763 0.996448344010977 0.47716049382716

hsa-miR-601 42.1052631578947 31.9649122807018 1.3172338090011 0.275533938516133 0.621872314704801 0.9974938201385 0.421008468166599 0.996448344010977 0.532098765432099

hsa-miR-571 58.3333333333333 66.219298245614 0.880911378990595 -0.126798249489725 0.361509519079543 0.9974938201385 0.601677663014866 0.996448344010977 0.440432098765432

hsa-miR-520d-5p 40.2807017543859 37.9298245614035 1.06197964847364 0.0601347592403034 0.702915955528711 0.9974938201385 0.571351274766041 0.996448344010977 0.475

hsa-miR-208a 80.8771929824561 77.7280701754386 1.04051461460332 0.0397154125033374 0.928019822250791 0.9974938201385 0.631608379997332 0.996448344010977 0.493827160493827

hsa-miR-617 16.8245614035088 29.3245614035088 0.573736165121149 -0.555585630976715 0.179820667926314 0.9974938201385 0.148186665756071 0.996448344010977 0.41358024691358

hsa-miR-136 1 22.5394736842105 0.0443666082895505 -3.11526815803779 0.182660880089311 0.9974938201385 0.346761130368377 0.996448344010977 0.418518518518519

hsa-miR-99aS 37.0175438596491 55.1578947368421 0.671119592875318 -0.398807927078985 0.184466436727279 0.9974938201385 0.385724292543652 0.996448344010977 0.41358024691358

hsa-miR-379S 43.6140350877193 52.4561403508772 0.831438127090301 -0.184598394314076 0.9053646248487 0.9974938201385 0.689145013955205 0.996448344010977 0.491975308641975

hsa-miR-34c-5p 59.438596491228 74.8245614035087 0.794372801875733 -0.230202404153835 0.136884367570868 0.9974938201385 0.0601024568958726 0.996448344010977 0.403086419753086

hsa-miR-523S 132.421052631579 127.142543859649 1.04151646342515 0.0406777890112859 0.558857667975849 0.9974938201385 0.570900096928977 0.996448344010977 0.538271604938272

hsa-miR-641 45.4035087719299 66.2828947368421 0.684995864350704 -0.378342478182365 0.186047133390198 0.9974938201385 0.312448212792088 0.996448344010977 0.413888888888889

hsa-miR-1206 32.2631578947368 51.6140350877193 0.625084976206662 -0.469867676557062 0.424869088508285 0.9974938201385 0.590696786278427 0.996448344010977 0.448148148148148

hsa-miR-202S 71.5087719298245 72.4561403508771 0.986924939467312 -0.0131612916124632 0.461278483880876 0.9974938201385 0.302861415393385 0.996448344010977 0.451851851851852

hsa-miR-496 77.7894736842105 109.302631578947 0.711688937041049 -0.340114349211034 0.122447003834183 0.9974938201385 0.197113394819781 0.996448344010977 0.399382716049383

hsa-miR-551a 17.0175438596491 29.5701754385965 0.575496885197271 -0.552521463176687 0.217809748660786 0.9974938201385 0.230737670148525 0.996448344010977 0.420987654320988

hsa-miR-1308 80.8771929824561 69.8684210526315 1.15756434400502 0.146318094206491 0.38818257275575 0.9974938201385 0.40000339377412 0.996448344010977 0.556172839506173

hsa-miR-221S 60.8070175438596 89.2302631578947 0.681461820147951 -0.383515052675437 0.100942395626466 0.9974938201385 0.147213826155404 0.996448344010977 0.39320987654321

hsa-miR-21 998.964912280702 1047.21710526316 0.953923410207924 -0.0471718935576057 0.882841719193725 0.9974938201385 0.780926431502132 0.997772027551652 0.509876543209877

hsa-miR-200cS 32.5964912280702 34.3070175438596 0.950140628995142 -0.0511452748217847 0.897431590961246 0.9974938201385 0.622713329908387 0.996448344010977 0.508641975308642

hsa-miR-545 94.3508771929824 99.25 0.950638561138362 -0.0506213505730402 0.984833519764045 1 0.528396906551988 0.996448344010977 0.498456790123457

hsa-miR-668 59.438596491228 75.6688596491228 0.785509346471526 -0.241422922602921 0.655040129440996 0.9974938201385 0.954348725126699 0.999654245801439 0.470679012345679

hsa-miR-147 83.3026315789474 52.8070175438596 1.57749169435216 0.455836050349424 0.0644173271212996 0.9974938201385 0.164984303416386 0.996448344010977 0.62037037037037

hsa-miR-149 23.4385964912281 43.2105263157895 0.542427933414535 -0.611700044023947 0.484488940979562 0.9974938201385 0.327839278495846 0.996448344010977 0.45462962962963

hsa-miR-517S 168.885964912281 164.513157894737 1.02658028739769 0.0262331691261715 0.533570461741661 0.9974938201385 0.828891739994478 0.999177463900866 0.540740740740741

hsa-let-7d 2746.45614035088 2434.5350877193 1.12812345741289 0.120555595147934 0.238113268154908 0.9974938201385 0.379668782881318 0.996448344010977 0.576851851851852

hsa-miR-181aS 70.4912280701754 54.2631578947368 1.29906239896541 0.26164277268981 0.661567204838976 0.9974938201385 0.460855608694895 0.996448344010977 0.528703703703704

hsa-miR-516a-5p 144.28947368421 158.429824561403 0.910746913238469 -0.0934902323515057 0.707325438163218 0.9974938201385 0.930640824689635 0.999654245801439 0.524691358024691

hsa-miR-453 39.4385964912281 46.9649122807018 0.839745984310796 -0.174655832506771 0.799661910751084 0.9974938201385 0.88632042870706 0.999654245801439 0.483333333333333

hsa-miR-138-2S 45.2631578947369 31.1052631578947 1.45516074450085 0.375116371841456 0.630534839527405 0.9974938201385 0.819285315643346 0.997772027551652 0.531481481481481

hsa-miR-214 342.780701754386 290.464912280702 1.18011053060731 0.165608104097011 0.617773704740179 0.9974938201385 0.678922867116184 0.996448344010977 0.532716049382716

hsa-let-7f-1S 12.2456140350877 11.6140350877193 1.05438066465257 0.052953546825364 0.728637326198425 0.9974938201385 0.604334861391102 0.996448344010977 0.521913580246914

hsa-miR-370 57.4736842105263 54.4561403508772 1.05541237113402 0.0539315636698505 1 1 0.968694476430967 0.999654245801439 0.500308641975309

hsa-miR-20a 4018.36622807017 3801.32565789474 1.05709602115375 0.0555255458593226 0.943143802859752 0.9974938201385 0.608964680436443 0.996448344010977 0.495061728395062

hsa-miR-502-3p 580.348684210526 565.981359649123 1.0253848016661 0.0250679583989303 0.51496441017876 0.9974938201385 0.291993186772356 0.996448344010977 0.457407407407407

hsa-miR-190b 1 1 1 0 0.965924482051219 0.999511784184894 0.686497354876326 0.996448344010977 0.497222222222222

hsa-miR-656 40.6666666666666 40.5 1.00411522633745 0.00410678195265328 0.789491457348504 0.9974938201385 0.853854556832766 0.999654245801439 0.517592592592593

hsa-miR-591 40.8947368421052 25.1754385964912 1.62439024390244 0.485132510842035 0.0847912459389664 0.9974938201385 0.503511442643493 0.996448344010977 0.611728395061728

hsa-miR-153 119.052631578947 132.382127192982 0.899310459072744 -0.10612696589162 0.655078384071227 0.9974938201385 0.993989777788092 0.999654245801439 0.470679012345679

hsa-miR-744 454.293859649123 464.998903508772 0.976978346015719 -0.0232907909355176 0.631228651069564 0.9974938201385 0.428019803607178 0.996448344010977 0.468518518518519

hsa-miR-143 204.765350877193 219.813596491228 0.931540878934504 -0.0709152048422194 0.127120437327922 0.9974938201385 0.921663565066134 0.999654245801439 0.400617283950617

hsa-miR-302a 1 1 1 0 0.963008812134392 0.999511784184894 0.996627345175813 0.999654245801439 0.496913580246914

hsa-miR-137 62.1951754385965 66.6491228070175 0.933173203474599 -0.0691644539121609 0.886628751850936 0.9974938201385 0.825503160685839 0.997772027551652 0.490432098765432

hsa-miR-34b 60.0350877192982 37.9298245614035 1.58279370952821 0.459191456252985 0.302102036982815 0.9974938201385 0.220941657711794 0.996448344010977 0.567283950617284

hsa-miR-595 255.043859649123 223.872807017544 1.13923554650001 0.130357464225997 0.67231807002759 0.9974938201385 0.225813414703375 0.996448344010977 0.527777777777778

hsa-miR-1915 521.372807017544 469.243421052631 1.11109241733847 0.105343691120922 0.295767685809244 0.9974938201385 0.107189895294605 0.996448344010977 0.56820987654321

hsa-miR-376c 82.8421052631579 68.6228070175438 1.207209510418 0.188311506520324 0.637981733742832 0.9974938201385 0.526767650768768 0.996448344010977 0.530864197530864

hsa-miR-148a 559.473684210526 604.529605263158 0.925469454828406 -0.0774541514803738 0.631195884382582 0.9974938201385 0.722217880616851 0.997772027551652 0.468518518518519

hsa-miR-1915S 12.7478070175439 13.8662280701754 0.919342084453582 -0.0840969903572629 0.774083598768118 0.9974938201385 0.390485221939847 0.996448344010977 0.481481481481482

hsa-miR-1909S 52.1929824561403 54.6688596491228 0.954711380320109 -0.0463462037390365 0.56835971300967 0.9974938201385 0.560407598454425 0.996448344010977 0.537345679012346

hsa-miR-211 1 1 1 0 0.570820063889706 0.9974938201385 0.49175979708457 0.996448344010977 0.533333333333333

hsa-miR-29c 370.144736842105 425.128289473684 0.87066597544085 -0.138496871158318 0.753780295682358 0.9974938201385 0.873738380933938 0.999654245801439 0.479320987654321

hsa-miR-371-3p 24.3859649122807 36.8684210526316 0.66143231025458 -0.413347628320433 0.396694089781152 0.9974938201385 0.790872913118327 0.997772027551652 0.445061728395062

hsa-miR-92a-2S 27.3333333333333 46.9649122807018 0.581994770265222 -0.541293817089659 0.741928402424748 0.9974938201385 0.60828376987375 0.996448344010977 0.478703703703704

hsa-miR-1293 58.8421052631579 54.9473684210526 1.07088122605364 0.0684818852724602 0.927938954579818 0.9974938201385 0.586872579327554 0.996448344010977 0.493827160493827

hsa-miR-493 40.3859649122807 30.0789473684211 1.34266550014581 0.294656817378852 0.763153497030056 0.9974938201385 0.824632164889636 0.997772027551652 0.480246913580247

hsa-miR-629 57.4736842105263 60.844298245614 0.944602631104704 -0.0569909361072052 0.934759923808728 0.9974938201385 0.641341388981823 0.996448344010977 0.494444444444444

hsa-miR-205 67.8070175438596 54.0350877192982 1.25487012987013 0.227032085053902 0.757354344694382 0.9974938201385 0.526137500845783 0.996448344010977 0.52037037037037

hsa-miR-221 96.3508771929824 61.2916666666666 1.57200615406634 0.45235260879982 0.0708490123128253 0.9974938201385 0.155487601349073 0.996448344010977 0.617592592592593

hsa-miR-515-5p 156.28947368421 121.705592105263 1.28416016865549 0.250104939431665 0.45273007652028 0.9974938201385 0.889194110096733 0.999654245801439 0.549074074074074

hsa-miR-563 29.6140350877193 12.5997807017544 2.35036115220608 0.85456899813805 0.28343045789783 0.9974938201385 0.391185269498165 0.996448344010977 0.568518518518519

hsa-miR-9 22.5964912280702 7.56140350877194 2.98839907192575 1.09473781656055 0.557130990803057 0.9974938201385 0.348472389184242 0.996448344010977 0.536728395061728

hsa-miR-511 18.6315789473684 28.0263157894737 0.664788732394366 -0.408285984449805 0.139409044332253 0.9974938201385 0.338921050511482 0.996448344010977 0.404320987654321

hsa-miR-128 421.381578947368 451.292763157895 0.933721108219807 -0.0685774846793476 0.996207973154461 1 0.99502874683783 0.999654245801439 0.500617283950617

hsa-miR-185 20902.4890350877 23622.6414473684 0.884849777771836 -0.122337391012812 0.124679744926864 0.9974938201385 0.114753800039438 0.996448344010977 0.404320987654321

hsa-miR-215 370.144736842105 377.464912280702 0.980607004252748 -0.0195835069669899 0.604437622268546 0.9974938201385 0.508021993175371 0.996448344010977 0.466049382716049

hsa-miR-886-3p 16.140350877193 9.50877192982457 1.69741697416974 0.529107668603439 0.682920875051269 0.9974938201385 0.903702070547958 0.999654245801439 0.525925925925926

hsa-miR-216a 101.333333333333 122.523026315789 0.827055422808101 -0.189883569507349 0.427382697186669 0.9974938201385 0.424580525680574 0.996448344010977 0.448148148148148

hsa-miR-1197 49.8947368421053 49.5087719298246 1.00779588944011 0.00776565851037212 0.909080559065674 0.9974938201385 0.815785273018712 0.997772027551652 0.492283950617284

hsa-miR-558 87.9122807017544 86.6140350877193 1.01498885963136 0.0148776367008623 0.562045187366161 0.9974938201385 0.382728319597095 0.996448344010977 0.537962962962963

hsa-miR-888S 42.4561403508772 43.7894736842105 0.969551282051281 -0.0309219103385868 0.71397770741312 0.9974938201385 0.682654538070917 0.996448344010977 0.475925925925926

hsa-miR-380S 71.0701754385965 86 0.826397388820889 -0.190679520885368 0.125916826011911 0.9974938201385 0.0332939889136158 0.996448344010977 0.400308641975309

hsa-miR-19a 1907.68201754386 1981.90570175439 0.962549336154176 -0.0381699557918608 0.875268239137664 0.9974938201385 0.833836050012817 0.999654245801439 0.510493827160494

hsa-miR-148b 458.607456140351 486.347587719298 0.942962333361139 -0.0587289405598651 0.852941357866087 0.9974938201385 0.754408684248001 0.997772027551652 0.487654320987654

hsa-miR-654-5p 142.09649122807 175.989035087719 0.807416730009595 -0.213915349933786 0.225564813775257 0.9974938201385 0.128636876144988 0.996448344010977 0.420987654320988

hsa-miR-23a 4503.74342105263 3749.71929824561 1.20108815162772 0.18322793892864 0.40524456128124 0.9974938201385 0.343360948622201 0.996448344010977 0.554320987654321

hsa-let-7dS 43.2478070175439 51.8289473684211 0.834433443344334 -0.181002295388892 0.774977765721445 0.9974938201385 0.318039212789329 0.996448344010977 0.481172839506173

hsa-miR-362-3p 225.798245614035 224.06798245614 1.00772204551015 0.00769238312173699 0.939387471378051 0.9974938201385 0.85130891530467 0.999654245801439 0.505246913580247

hsa-miR-552 12.2456140350877 21.1315789473684 0.579493565794935 -0.545600719292554 0.380749643748703 0.9974938201385 0.42661278461358 0.996448344010977 0.443827160493827

hsa-miR-562 20.5614035087719 39.2105263157895 0.524384787472036 -0.64552953691071 0.117592697442126 0.9974938201385 0.159822871516674 0.996448344010977 0.399074074074074

hsa-miR-337-3p 31.7543859649123 52.9824561403509 0.599337748344371 -0.511929986109044 0.189206658916026 0.9974938201385 0.147024545275216 0.996448344010977 0.414814814814815

hsa-miR-323-5p 44.421052631579 65.1951754385965 0.681354905984056 -0.383671954424681 0.539717525136529 0.9974938201385 0.594458810572552 0.996448344010977 0.459876543209877

hsa-miR-548o 125.649122807018 105.736842105263 1.18831923013108 0.172539897075334 0.478860684739105 0.9974938201385 0.471896298263484 0.996448344010977 0.546296296296296

hsa-miR-1245 10.2456140350877 28.6052631578947 0.358172339773076 -1.02674101240115 0.266339111045861 0.9974938201385 0.938019287535766 0.999654245801439 0.428703703703704

hsa-miR-1201 49.8947368421053 70.7807017543859 0.704920064444169 -0.349670866365429 0.165353940250114 0.9974938201385 0.136163804909108 0.996448344010977 0.409876543209877

hsa-miR-218-2S 58.8421052631579 65.3684210526315 0.900161030595813 -0.105181608778379 0.278570629393916 0.9974938201385 0.64360132382953 0.996448344010977 0.429320987654321

hsa-miR-95 12.0701754385965 28.7982456140351 0.419128845568078 -0.869576899008304 0.213295110567211 0.9974938201385 0.581231469264847 0.996448344010977 0.42037037037037

hsa-miR-513c 24.9473684210526 17.0438596491228 1.46371590324241 0.380978341545691 0.726007389986536 0.9974938201385 0.518167241753141 0.996448344010977 0.522839506172839

hsa-miR-582-5p 38.7719298245614 35.5175438596491 1.09162756236108 0.0876697590395828 0.685109667211183 0.9974938201385 0.287344207435614 0.996448344010977 0.526543209876543

hsa-miR-146aS 96 92.5899122807018 1.03683001350039 0.0361679944032769 0.901572064904857 0.9974938201385 0.956070252766502 0.999654245801439 0.508333333333333

hsa-miR-19b-2S 1 1 1 0 0.773067961153037 0.9974938201385 0.741379892801759 0.997772027551652 0.483024691358025

hsa-miR-92a 12702.7105263158 11503.9078947368 1.10420829535044 0.0991286033830008 0.376090903668666 0.9974938201385 0.457756410992642 0.996448344010977 0.557407407407407

hsa-miR-30aS 30.140350877193 51.2456140350877 0.588154741526874 -0.53076519984354 0.18909577778774 0.9974938201385 0.56106805850542 0.996448344010977 0.414506172839506

hsa-miR-885-3p 200.342105263158 196.245614035088 1.02087430716968 0.0206594240328437 0.57494717274822 0.9974938201385 0.381166113108455 0.996448344010977 0.536728395061728

hsa-miR-451 1212.00438596491 1305.42214912281 0.928438656245669 -0.0742509679587218 0.886583583107365 0.9974938201385 0.367288308224895 0.996448344010977 0.509567901234568

hsa-miR-7 43.6140350877193 50.1140350877193 0.870295816558726 -0.13892210609386 0.744424771775425 0.9974938201385 0.509193345706282 0.996448344010977 0.478703703703704

hsa-miR-518b 106.964912280702 85.1842105263158 1.25568942436412 0.227684763873407 0.875343745519639 0.9974938201385 0.46628735428375 0.996448344010977 0.489506172839506

hsa-miR-498 36.8070175438596 48.8771929824561 0.753050969131371 -0.283622365383509 0.308163904835389 0.9974938201385 0.519428374192653 0.996448344010977 0.433641975308642

hsa-miR-525-5p 88.9122807017544 98.1271929824561 0.906092164662763 -0.0986142511121557 0.457705188451986 0.9974938201385 0.268180615508357 0.996448344010977 0.451234567901235

hsa-miR-1537 39.4385964912281 44.1052631578948 0.894192521877486 -0.111834178136612 0.882124839638917 0.9974938201385 0.669061198220988 0.996448344010977 0.509876543209877

hsa-miR-31S 147.565789473684 154.078947368421 0.957728437233134 -0.0431910096374549 0.152581131072574 0.9974938201385 0.480138978047426 0.996448344010977 0.40679012345679

hsa-miR-372 24.5087719298246 32.5438596491228 0.753099730458221 -0.283557615784573 0.205637631112876 0.9974938201385 0.36531564679849 0.996448344010977 0.41820987654321

hsa-miR-1257 24.0175438596491 23.4703947368421 1.0233123102079 0.0230447289491834 0.741928402424748 0.9974938201385 0.738473401035064 0.997772027551652 0.478703703703704

hsa-miR-30a 206.504385964912 295.219298245614 0.699494874461447 -0.35739681233526 0.689744412983784 0.9974938201385 0.510653079860752 0.996448344010977 0.473765432098765

hsa-miR-200b 57.3333333333333 57.1578947368421 1.00306936771025 0.00306466681790294 0.786469684191909 0.9974938201385 0.977221497133462 0.999654245801439 0.482098765432099

hsa-miR-605 27.3333333333333 26.7719298245614 1.02096985583224 0.0207530145862912 0.821934995870102 0.9974938201385 0.573259956219368 0.996448344010977 0.485185185185185

hsa-miR-1228S 1091.65789473684 917.378289473684 1.18997572458701 0.173932907408654 0.458374708027009 0.9974938201385 0.121744362739121 0.996448344010977 0.548456790123457

hsa-miR-1912 156.921052631579 146.714912280702 1.0695644375355 0.067251497875426 0.775495388587575 0.9974938201385 0.9983546809087 0.999654245801439 0.518827160493827

hsa-miR-219-5p 61.3684210526316 53.7225877192982 1.14232064496377 0.13306184674841 0.863732948496808 0.9974938201385 0.344320903935919 0.996448344010977 0.51141975308642

hsa-miR-325 47.7543859649123 26.140350877193 1.82684563758389 0.602590784271906 0.137893830675031 0.9974938201385 0.0723542267257938 0.996448344010977 0.596296296296296

hsa-miR-1270 18.7719298245614 22.6293859649123 0.829537745905611 -0.186886665979926 0.599638874863208 0.9974938201385 0.951895698221848 0.999654245801439 0.466666666666667

hsa-miR-659 87.0175438596491 90.0723684210526 0.966084775886836 -0.0345036889030567 0.614421677849124 0.9974938201385 0.850014960335282 0.999654245801439 0.533024691358025

hsa-miR-190 1 3.90789473684211 0.255892255892256 -1.36299879851637 0.260641399327153 0.9974938201385 0.808926125298441 0.997772027551652 0.431172839506173

hsa-miR-494 69.8245614035087 69.8684210526315 0.999372253609542 -0.00062794350572043 0.875368883858981 0.9974938201385 0.919551786065476 0.999654245801439 0.489506172839506

hsa-miR-553 1 1 1 0 0.696663389911566 0.9974938201385 0.977685408691929 0.999654245801439 0.477160493827161

hsa-miR-183S 113.929824561404 125.866228070175 0.90516595522258 -0.0996369761569702 0.416396603234812 0.9974938201385 0.480273029289403 0.996448344010977 0.446913580246914

hsa-miR-99b 124.394736842105 153.342105263158 0.811223614209714 -0.209211536349837 0.368998195546369 0.9974938201385 0.472241651474288 0.996448344010977 0.441358024691358

hsa-miR-19b-1S 90.125 71.3004385964912 1.2640174699351 0.234295116780344 0.841794431574966 0.9974938201385 0.813710497745917 0.997772027551652 0.513271604938272

hsa-miR-644 6.77192982456141 29.5964912280702 0.228808535862478 -1.47486971308114 0.0509827026111341 0.9974938201385 0.250372564052551 0.996448344010977 0.375308641975309

hsa-miR-515-3p 1 16.4561403508772 0.0607675906183369 -2.80069868117167 0.469910748254813 0.9974938201385 0.813562233197434 0.997772027551652 0.455246913580247

hsa-miR-369-3p 1 8.49013157894737 0.11778380472685 -2.13890449831239 0.263465638998119 0.9974938201385 0.182828658250817 0.996448344010977 0.432716049382716

hsa-miR-647 34.2105263157895 59.3530701754386 0.576390171808609 -0.550970465993449 0.213075392114779 0.9974938201385 0.224613262888355 0.996448344010977 0.419135802469136

hsa-miR-527 158.429824561403 128.804824561404 1.22999914871882 0.207013477285567 0.530418739479588 0.9974938201385 0.586104810297738 0.996448344010977 0.541049382716049

hsa-miR-135bS 52.1929824561403 51.8870614035088 1.00589590245346 0.0058785896370403 0.710536061448589 0.9974938201385 0.706298361423485 0.996448344010977 0.475617283950617

hsa-miR-638 270.30701754386 239.65350877193 1.12790761516078 0.120364248268548 0.381882779588698 0.9974938201385 0.228592304974289 0.996448344010977 0.557098765432099

hsa-miR-510 96.3508771929824 97.1875 0.991391662435832 -0.008645603320242 0.882875218246566 0.9974938201385 0.426402901087445 0.996448344010977 0.509876543209877

hsa-miR-584 59.438596491228 39.9210526315789 1.48890353768403 0.398039968315248 0.736071029859139 0.9974938201385 0.830012801162788 0.999177463900866 0.521913580246914

hsa-miR-335S 18.6315789473684 1 18.6315789473684 2.92485793396733 0.0863712278248185 0.9974938201385 0.432818454543157 0.996448344010977 0.607098765432099

hsa-miR-671-3p 51.1578947368421 53.4835526315789 0.956516390921951 -0.044457353895335 0.563135804740507 0.9974938201385 0.268068395437568 0.996448344010977 0.537654320987654

hsa-miR-454S 41.8771929824561 65.3684210526315 0.640633387010199 -0.445297924822703 0.487249027802615 0.9974938201385 0.438668189967664 0.996448344010977 0.45462962962963

hsa-miR-520d-3p 8.52631578947369 3.90789473684211 2.18181818181818 0.780158557549575 0.912091414506159 0.9974938201385 0.380673802015318 0.996448344010977 0.507098765432099

hsa-miR-17 8005.76315789474 7178.66063596491 1.11521682997328 0.1090488523497 0.9733967091141 1 0.807605511255542 0.997772027551652 0.497530864197531

hsa-miR-1277 45.2631578947369 25.8859649122807 1.74855981023382 0.558792483532669 0.418892491560833 0.9974938201385 0.942411017024288 0.999654245801439 0.552469135802469

hsa-miR-566 83.3026315789474 70.5087719298245 1.18145060960438 0.166743013643145 0.614203578223948 0.9974938201385 0.954692867643334 0.999654245801439 0.533024691358025

hsa-miR-24-2S 144.600877192982 144.131578947368 1.00325604041142 0.00325075099043937 0.909192055828794 0.9974938201385 0.503819351078711 0.996448344010977 0.507716049382716

hsa-miR-105S 1 31.4649122807018 0.0317814329523278 -3.44887302913779 0.00936289316352516 0.9974938201385 0.238440403038283 0.996448344010977 0.336728395061728

hsa-miR-551bS 58.7017543859649 28.7982456140351 2.03837953091684 0.712155144605122 0.198653326892455 0.9974938201385 0.244207771753005 0.996448344010977 0.583333333333333

hsa-miR-192 4503.74342105263 4831.82894736842 0.932099101626005 -0.0703161377326719 0.243417142488902 0.9974938201385 0.35014536753284 0.996448344010977 0.424074074074074

hsa-miR-328 52.578947368421 44.7543859649123 1.17483339866719 0.161126349174851 0.68510627605593 0.9974938201385 0.731490944515772 0.997772027551652 0.526543209876543

hsa-miR-342-3p 3004.33333333333 3467.72368421053 0.866370451317349 -0.143442689004663 0.0528462246299863 0.9974938201385 0.0375548151558434 0.996448344010977 0.374074074074074

hsa-miR-1283 83.1074561403509 95.0263157894737 0.874573063786578 -0.134019438800739 0.35375982985215 0.9974938201385 0.421505153308822 0.996448344010977 0.439506172839506

hsa-miR-555 42.0350877192982 39.6228070175438 1.06088111578481 0.0590998041406812 0.804578895715377 0.9974938201385 0.23307544685423 0.996448344010977 0.516358024691358

hsa-miR-1 20.5614035087719 19.0657894736842 1.07844490453186 0.0755201002680568 0.679217567948739 0.9974938201385 0.645495957158212 0.996448344010977 0.473456790123457

hsa-miR-645 74.0175438596491 56.0438596491228 1.32070746595711 0.27816755208194 0.536655222836575 0.9974938201385 0.789642882557161 0.997772027551652 0.540432098765432

hsa-miR-889 31.4385964912281 22.5701754385965 1.39292654488923 0.331406961813077 0.798878457070104 0.9974938201385 0.29838992904884 0.996448344010977 0.516666666666667

hsa-miR-1321 16.421052631579 12.1228070175439 1.35455861070912 0.303475652709922 0.69109517285725 0.9974938201385 0.849389165007446 0.999654245801439 0.525308641975309

hsa-miR-664 267.745614035088 274.078947368421 0.976892302768443 -0.02337886559633 0.746565912381122 0.9974938201385 0.73754798069535 0.997772027551652 0.478703703703704

hsa-miR-548f 27.3333333333333 33.6842105263158 0.811458333333334 -0.208922238591133 0.354332981209112 0.9974938201385 0.1895363007381 0.996448344010977 0.439814814814815

hsa-miR-513a-3p 66.4912280701754 48.7894736842105 1.36281912980942 0.309555443842506 0.443896467808977 0.9974938201385 0.802550526208921 0.997772027551652 0.55

hsa-miR-1282 1 1 1 0 0.612695609931314 0.9974938201385 0.281236409995172 0.996448344010977 0.529320987654321

hsa-miR-222 372.427631578947 342.780701754386 1.08648949509942 0.0829518520982734 0.977251221650087 1 0.867493768259922 0.999654245801439 0.49783950617284

hsa-miR-424S 144.28947368421 128.621710526316 1.12181274136211 0.114945895989902 0.455516625764677 0.9974938201385 0.629937436160106 0.996448344010977 0.548765432098765

hsa-miR-624 82.7719298245614 77.9671052631579 1.06162630439063 0.0598019817908985 0.860415007961148 0.9974938201385 0.91807986995843 0.999654245801439 0.511728395061728

hsa-miR-125a-5p 192.394736842105 171.19298245614 1.12384709981554 0.116757710025698 0.658511937655784 0.9974938201385 0.85252603063595 0.999654245801439 0.529012345679012

hsa-miR-139-5p 164.131578947368 164.368421052632 0.998559077809798 -0.00144196131690141 0.62111582089311 0.9974938201385 0.53542468624247 0.996448344010977 0.532407407407407

hsa-miR-572 87.9649122807018 67.298245614035 1.30709071949948 0.267803842714911 0.0651959727489161 0.9974938201385 0.184672590884148 0.996448344010977 0.620061728395062

hsa-let-7e 168.885964912281 188.184210526316 0.897450240059666 -0.10819760298717 0.830453881941019 0.9974938201385 0.884551812751729 0.999654245801439 0.485802469135802

hsa-miR-141S 109.074561403509 109.197368421053 0.998875366510021 -0.00112526636476805 0.546141594768791 0.9974938201385 0.739362234909616 0.997772027551652 0.539506172839506

hsa-miR-940 215.622807017544 197.69298245614 1.09069530106048 0.086815383756316 0.591252653147909 0.9974938201385 0.70705146173245 0.996448344010977 0.464814814814815

hsa-miR-1909 148.28947368421 132.026315789474 1.12318118397449 0.116165001989623 0.0585548439043981 0.9974938201385 0.015918619802981 0.996448344010977 0.623148148148148

hsa-miR-454 168.885964912281 152.241228070175 1.10933133588776 0.103757433707284 0.546139328463301 0.9974938201385 0.947474736602174 0.999654245801439 0.539506172839506

hsa-miR-126S 34.5263157894737 38.2631578947368 0.902338376891335 -0.10276568858943 0.738228439202266 0.9974938201385 0.50412748320657 0.996448344010977 0.521913580246914

hsa-miR-660 357.271929824561 450.353070175438 0.793315186427803 -0.231534675497015 0.0991265694709654 0.9974938201385 0.505694245801688 0.996448344010977 0.392592592592593

hsa-miR-410 103.719298245614 99.0043859649123 1.04762326673459 0.0465240429643505 0.912959858699668 0.9974938201385 0.675928606097598 0.996448344010977 0.507407407407407

hsa-miR-1266 100.175438596491 84.5964912280702 1.18415595188718 0.16903024390894 0.871621585423528 0.9974938201385 0.606558943244648 0.996448344010977 0.510802469135802

hsa-miR-548i 1 2.35855263157895 0.423988842398884 -0.858048139193398 0.806839531768859 0.9974938201385 0.492713906657726 0.996448344010977 0.484876543209877

hsa-miR-720 2295.07456140351 2349.97368421053 0.976638409537994 -0.0236387982713363 0.916667284830348 0.9974938201385 0.632688409208085 0.996448344010977 0.492901234567901

hsa-miR-30d 4936.95614035088 4936.95614035088 1 0 0.760640874235973 0.9974938201385 0.480051489595265 0.996448344010977 0.479938271604938

hsa-miR-587 56.3508771929825 50.4210526315789 1.11760612386917 0.111189008427682 0.867736296756928 0.9974938201385 0.797062329754674 0.997772027551652 0.488888888888889

hsa-miR-624S 39.1578947368421 39.0438596491228 1.00292069197933 0.00291643504529259 0.861883534808914 0.9974938201385 0.984738801636623 0.999654245801439 0.51141975308642

hsa-miR-548k 21.0657894736842 20.7324561403509 1.01607785064523 0.0159499708715884 0.70364728677568 0.9974938201385 0.54511719528776 0.996448344010977 0.475308641975309

hsa-miR-501-5p 51.280701754386 59.0877192982456 0.867874109263658 -0.141708610250971 0.717717114256791 0.9974938201385 0.957067975294549 0.999654245801439 0.476234567901235

hsa-miR-890 21.0657894736842 9.32456140350878 2.25917215428034 0.814998442762931 0.159144617018962 0.9974938201385 0.165895927712096 0.996448344010977 0.588888888888889

hsa-miR-374aS 44.421052631579 39.0350877192982 1.13797752808989 0.129252588663727 0.150386217647923 0.9974938201385 0.154479033700557 0.996448344010977 0.593518518518519

hsa-miR-1279 13.9649122807018 9.96491228070176 1.40140845070423 0.337477767123231 0.687483385490044 0.9974938201385 0.372013012884919 0.996448344010977 0.525617283950617

hsa-miR-450b-3p 16.8245614035088 18.9912280701754 0.885912240184757 -0.121137384993207 0.827490766309439 0.9974938201385 0.51902904272328 0.996448344010977 0.485802469135802

hsa-miR-155 67.719298245614 77.3333333333333 0.87568058076225 -0.132753888373874 0.356548823724719 0.9974938201385 0.590410836319444 0.996448344010977 0.439814814814815

hsa-miR-1286 93.1578947368421 114.828947368421 0.811275352354761 -0.209147760475546 0.682700666615312 0.9974938201385 0.713317373595641 0.996448344010977 0.473148148148148

hsa-miR-485-3p 74.4210526315789 67.3508771929824 1.10497525397239 0.0998229401238299 0.890334896960976 0.9974938201385 0.607343472064853 0.996448344010977 0.509259259259259

hsa-miR-520a-3p 11.8421052631579 28.6578947368421 0.413223140495867 -0.883767540168596 0.246061555322152 0.9974938201385 0.377710980181082 0.996448344010977 0.425925925925926

hsa-miR-922 116.339912280702 103.982456140351 1.11884174118441 0.112293990538169 0.658477622134608 0.9974938201385 0.781868385293299 0.997772027551652 0.529012345679012

hsa-miR-26a-1S 16.9473684210526 5.81578947368421 2.91402714932127 1.0695360245866 0.350655232144017 0.9974938201385 0.580774053900311 0.996448344010977 0.558641975308642

hsa-miR-516b 31.7543859649123 38.5087719298245 0.824601366742597 -0.1928552012494 0.759941190566009 0.9974938201385 0.462222166787619 0.996448344010977 0.520061728395062

hsa-miR-487b 42.3157894736842 37.0526315789474 1.14204545454545 0.132820913020924 0.954312751023324 0.999511784184894 0.601604821361383 0.996448344010977 0.504012345679012

hsa-miR-204 12.0701754385965 7.13157894736842 1.69249692496925 0.526204908945478 0.790265486554632 0.9974938201385 0.990416777174631 0.999654245801439 0.516666666666667

hsa-miR-1471 110.530701754386 109.144736842105 1.01269841269841 0.0126184639592112 0.739310923528783 0.9974938201385 0.686927241771792 0.996448344010977 0.521913580246914

hsa-miR-140-3p 20902.4890350877 20902.4890350877 1 0 0.573531460376248 0.9974938201385 0.861142494683205 0.999654245801439 0.535185185185185

hsa-miR-18aS 158.043859649123 143.535087719298 1.10108170873312 0.0962930681868047 0.527306324023183 0.9974938201385 0.59676112854837 0.996448344010977 0.541358024691358

hsa-miR-218-1S 110.936403508772 86.4144736842105 1.2837710950387 0.249801914482934 0.0879565465933743 0.9974938201385 0.0753227009301165 0.996448344010977 0.611111111111111

hsa-miR-181a 287.973684210526 269.289473684211 1.0693833675364 0.0670821903316875 0.655038307475033 0.9974938201385 0.577887781178049 0.996448344010977 0.529320987654321

hsa-miR-1179 15.0877192982456 32.7368421052632 0.460878885316185 -0.774619992159735 0.565218635394363 0.9974938201385 0.199944803119157 0.996448344010977 0.462962962962963

hsa-miR-1289 101.333333333333 105.622807017544 0.959388755086787 -0.0414589107596116 0.841794431574966 0.9974938201385 0.841398260777198 0.999654245801439 0.513271604938272

hsa-miR-665 115.087719298246 115.142543859649 0.999523854870965 -0.000476258522122913 0.790136684703092 0.9974938201385 0.650962748141712 0.996448344010977 0.482407407407407

hsa-miR-223 1799.94736842105 1956.55592105263 0.919957027066559 -0.0834283197402451 0.875347237865282 0.9974938201385 0.754892094892948 0.997772027551652 0.489506172839506

hsa-miR-301b 178.5 186.777412280702 0.9556830123106 -0.0453289979955325 0.841787379406536 0.9974938201385 0.626027809469967 0.996448344010977 0.486728395061728

hsa-miR-488S 60.5614035087719 50.5350877192982 1.19840305502517 0.180989883030881 0.988611071452333 1 0.817691396191603 0.997772027551652 0.501234567901235

hsa-miR-548d-3p 24.0175438596491 24.530701754386 0.979080994099768 -0.0211409084132484 0.627281089315957 0.9974938201385 0.238257727815583 0.996448344010977 0.468518518518519

hsa-miR-365 102.019736842105 90.4035087719298 1.12849311080924 0.120883212524198 0.801134717563901 0.9974938201385 0.619270873779851 0.996448344010977 0.516666666666667

hsa-miR-99bS 78.5614035087719 71.7456140350877 1.09499938867832 0.0907538049836717 0.856596204613275 0.9974938201385 0.36745872906078 0.996448344010977 0.512037037037037

hsa-miR-590-5p 96.3508771929824 122.088815789474 0.789186761866271 -0.236752279092115 0.472873436427604 0.9974938201385 0.618923526315829 0.996448344010977 0.453086419753086

hsa-miR-122 49.6491228070175 43.1929824561404 1.14947197400487 0.139302683892884 0.241167646124924 0.9974938201385 0.141880998208743 0.996448344010977 0.576234567901235

hsa-miR-181cS 69.8245614035087 62.1513157894737 1.12346071063124 0.116413841521075 0.653836852349465 0.9974938201385 0.662866685081691 0.996448344010977 0.470679012345679

hsa-miR-151-3p 426.524122807017 479.304824561403 0.889880720890544 -0.11666784670893 1 1 0.968320902969501 0.999654245801439 0.5

hsa-miR-195S 77.1929824561403 64.5811403508772 1.19528676695304 0.178386128940354 0.644666782444832 0.9974938201385 0.46541484646885 0.996448344010977 0.530246913580247

hsa-miR-522S 93.4035087719298 83.3684210526316 1.12037037037037 0.113659318472521 0.935609358102731 0.9974938201385 0.714594866235962 0.996448344010977 0.505555555555556

hsa-miR-613 12.0701754385965 11.4100877192982 1.05785123966942 0.0562397183228757 0.792796777392612 0.9974938201385 0.838677406431752 0.999654245801439 0.483333333333333

hsa-miR-1231 89.0877192982456 59.6513157894737 1.4934745046138 0.401105287630295 0.100113433243744 0.9974938201385 0.123941715062829 0.996448344010977 0.607098765432099

hsa-miR-326 103.719298245614 76.7631578947368 1.35115986744372 0.300963384652152 0.0584523003893748 0.9974938201385 0.0373195432606472 0.996448344010977 0.623148148148148

hsa-miR-374b 458.607456140351 487.24451754386 0.941226508719144 -0.06057145771558 0.481811773329788 0.9974938201385 0.276146168622265 0.996448344010977 0.454012345679012

hsa-miR-1914 59.5438596491228 87.3267543859649 0.681851284498129 -0.382943702837392 0.0942087707797962 0.9974938201385 0.238347936956491 0.996448344010977 0.391049382716049

hsa-miR-23bS 31.7543859649123 29.1666666666667 1.08872180451128 0.0850043517298619 0.877912887659463 0.9974938201385 0.610694537576634 0.996448344010977 0.489814814814815

hsa-miR-596 96.2456140350877 86.0526315789474 1.1184505606524 0.111944299498085 0.661924099536289 0.9974938201385 0.231302177045684 0.996448344010977 0.528703703703704

hsa-miR-551b 60.0350877192982 50.9649122807018 1.17796901893287 0.163791785166127 0.954479317668641 0.999511784184894 0.566255088471353 0.996448344010977 0.495987654320988

hsa-miR-640 101.631578947368 101.0625 1.00563096051818 0.00561516592474838 0.627866797734897 0.9974938201385 0.96789934813423 0.999654245801439 0.53179012345679

hsa-miR-490-5p 106.157894736842 106.089912280702 1.00064080038035 0.000640595155449696 0.594506219394749 0.9974938201385 0.657334209982356 0.996448344010977 0.534876543209877

hsa-miR-1296 35.3508771929824 52.8771929824561 0.668546781685468 -0.402648904805658 0.490238935206592 0.9974938201385 0.673628416055073 0.996448344010977 0.454938271604938

hsa-miR-10b 62.1951754385965 77.0175438596491 0.80754555808656 -0.213755806793753 0.764595050296576 0.9974938201385 0.7424384821514 0.997772027551652 0.480246913580247

hsa-miR-362-5p 131.666666666667 138.021929824561 0.953954685563571 -0.047139108071238 0.597807833114699 0.9974938201385 0.965852681165357 0.999654245801439 0.534567901234568

hsa-miR-564 122.078947368421 116.439692982456 1.04843068752178 0.0472944628530936 0.624391588963093 0.9974938201385 0.269318816468809 0.996448344010977 0.467901234567901

hsa-miR-20b 2637.20614035088 2858.37390350877 0.922624621332289 -0.0805328213162958 0.458161334387782 0.9974938201385 0.517634986526739 0.996448344010977 0.451543209876543

hsa-miR-1469 159.482456140351 173.811403508772 0.917560372454515 -0.0860369001778614 0.96209385933801 0.999511784184894 0.689675841371215 0.996448344010977 0.496604938271605

hsa-miR-331-3p 666.407894736842 583.78125 1.14153699649799 0.132375596885625 0.693211356895343 0.9974938201385 0.790562238227856 0.997772027551652 0.525925925925926

hsa-miR-512-5p 57.9649122807017 60.0899122807017 0.964636327141345 -0.0360041117398462 0.988623876645218 1 0.982224154909554 0.999654245801439 0.498765432098765

hsa-miR-589S 88.1578947368421 82.3530701754386 1.07048704497643 0.0681137271307791 0.475897139515754 0.9974938201385 0.517789675307989 0.996448344010977 0.453395061728395

hsa-miR-181b 40.8947368421052 33.4561403508772 1.22233875196644 0.200766033435338 0.245790121785129 0.9974938201385 0.529514953571778 0.996448344010977 0.575617283950617

hsa-miR-519a 12.0701754385965 21.2291666666667 0.568565673260678 -0.564638452216041 0.593936937676284 0.9974938201385 0.955359923603334 0.999654245801439 0.466049382716049

hsa-miR-1205 57.1578947368421 25.5877192982456 2.233801851217 0.803704999854214 0.138520829037633 0.9974938201385 0.15134293598333 0.996448344010977 0.595987654320988

hsa-miR-548p 127.548245614035 113.899122807018 1.1198351881089 0.113181521004671 0.79379776432732 0.9974938201385 0.862342280562799 0.999654245801439 0.517283950617284

hsa-miR-943 56.3508771929825 42.0175438596491 1.34112734864301 0.293510565221637 0.104797010821869 0.9974938201385 0.0405111490642561 0.996448344010977 0.605246913580247

hsa-miR-520b 29.1578947368421 31.5175438596491 0.925132201502922 -0.0778186311381801 0.904598712454914 0.9974938201385 0.805012758244286 0.997772027551652 0.491975308641975

hsa-miR-548a-3p 62.563596491228 72.1140350877193 0.867564773141953 -0.142065103502467 0.527308656467554 0.9974938201385 0.45952876684263 0.996448344010977 0.458641975308642

hsa-miR-921 63.1929824561403 61.9122807017544 1.02068574667044 0.0204747020606313 0.823203910908996 0.9974938201385 0.416270128127571 0.996448344010977 0.514814814814815

hsa-miR-708 57.1578947368421 47.7368421052632 1.1973539140022 0.180114050378744 0.63053677757853 0.9974938201385 0.60775860887673 0.996448344010977 0.531481481481481

hsa-miR-198 84.0921052631579 69.9298245614035 1.20252132463623 0.184420456425762 0.672185558627787 0.9974938201385 0.716746318726894 0.997664633969853 0.527777777777778

hsa-miR-130bS 16.9473684210526 25.0263157894737 0.677181913774973 -0.389815336441037 0.462570085607568 0.9974938201385 0.590978878091775 0.996448344010977 0.453086419753086

hsa-miR-1306 99.1447368421052 57.859649122807 1.71353850818678 0.538560535489271 0.422060200785883 0.9974938201385 0.935288477090989 0.999654245801439 0.551851851851852

hsa-let-7i 372.427631578947 329.429824561403 1.13052190121156 0.122679385579243 0.838071952964283 0.9974938201385 0.918694937097438 0.999654245801439 0.48641975308642

hsa-miR-302cS 16.8245614035088 37.3684210526316 0.450234741784038 -0.797986183820032 0.409324851130357 0.9974938201385 0.390700140640879 0.996448344010977 0.447222222222222

hsa-miR-632 24.0175438596491 38.4561403508772 0.624543795620438 -0.470733822779457 0.514397049314369 0.9974938201385 0.517505454686837 0.996448344010977 0.457716049382716

hsa-miR-580 36.280701754386 31.359649122807 1.15692307692308 0.145763961060157 0.634117453697793 0.9974938201385 0.28696927693553 0.996448344010977 0.530864197530864

hsa-miR-10bS 52.1929824561403 58.7894736842105 0.88779468815279 -0.11901476975758 0.341426013436768 0.9974938201385 0.322794128425773 0.996448344010977 0.437962962962963

hsa-miR-200c 87.5789473684211 95.1403508771929 0.920523695371566 -0.0828125367917966 0.954518109925723 0.999511784184894 0.729182143094035 0.997772027551652 0.504012345679012

hsa-miR-1274b 639.035087719298 627.032894736842 1.01914124933987 0.0189603602769832 0.76098843050404 0.9974938201385 0.631542749888152 0.996448344010977 0.520061728395062

hsa-miR-488 3.29166666666667 1 3.29166666666667 1.19139402211908 0.315369050518881 0.9974938201385 0.246792169133483 0.996448344010977 0.560802469135802

hsa-miR-625 78.2631578947368 109.267543859649 0.716252558904989 -0.333722438317351 0.253909225031152 0.9974938201385 0.24292927914445 0.996448344010977 0.425617283950617

hsa-miR-299-3p 3.08771929824561 5.42105263157895 0.56957928802589 -0.562857281859594 0.755703362364378 0.9974938201385 0.698947484525027 0.996448344010977 0.480555555555556

hsa-miR-320a 6067.7149122807 6067.7149122807 1 0 0.924229702760844 0.9974938201385 0.80297511328415 0.997772027551652 0.493518518518519

hsa-miR-486-5p 39283.3070175439 39283.3070175439 1 0 0.214641194686458 0.9974938201385 0.24302304275954 0.996448344010977 0.530555555555556

hsa-miR-589 58.8421052631579 53.8256578947368 1.09319806881379 0.08910740852593 0.640727023060979 0.9974938201385 0.996665510269583 0.999654245801439 0.530555555555556

hsa-miR-342-5p 100.175438596491 106.949561403509 0.93666058356743 -0.0654342997555404 0.782820235169956 0.9974938201385 0.741718880051811 0.997772027551652 0.48179012345679

hsa-miR-639 57.3333333333333 59.9736842105263 0.955974842767296 -0.0450236813739549 0.973316373245401 1 0.390297843100297 0.996448344010977 0.497530864197531

hsa-miR-518eS 152.219298245614 127.961074561404 1.18957502324326 0.173596120014724 0.882864055088548 0.9974938201385 0.70510221054355 0.996448344010977 0.490123456790123

hsa-miR-224 30.7368421052632 18.0877192982456 1.69932104752667 0.530228787479376 0.62438392787627 0.9974938201385 0.59199625606224 0.996448344010977 0.53179012345679

hsa-miR-181d 26.4561403508772 41.2631578947368 0.641156462585035 -0.444481760450615 0.3055759486131 0.9974938201385 0.399866286021408 0.996448344010977 0.434259259259259

hsa-miR-192S 82.7719298245614 102.421052631579 0.808153477218226 -0.213003291446439 0.996206473366966 1 0.714137733157461 0.996448344010977 0.500617283950617

hsa-miR-30b 7477.03728070175 7178.66063596491 1.04156438921795 0.0407238033488949 0.226264161806662 0.9974938201385 0.107782065577938 0.996448344010977 0.578703703703704

hsa-miR-183 259.482456140351 377.66447368421 0.687071393316494 -0.375317071756673 0.0802819292044759 0.9974938201385 0.096288799923829 0.996448344010977 0.386111111111111

hsa-miR-105 50.6754385964912 35.9298245614035 1.410400390625 0.343873629061922 0.56347407962862 0.9974938201385 0.238661768880525 0.996448344010977 0.537654320987654

hsa-miR-1246 12.0701754385965 2.32236842105263 5.19735599622285 1.64815003401177 0.781550542952992 0.9974938201385 0.796708574542815 0.997772027551652 0.517283950617284

hsa-miR-20aS 119.964912280702 154.061403508772 0.778682457438935 -0.250151944662543 0.318277005357933 0.9974938201385 0.278032256708663 0.996448344010977 0.434876543209877

hsa-miR-142-3p 79.4912280701754 87.719298245614 0.9062 -0.098495246749099 0.759739392137222 0.9974938201385 0.62133738855725 0.996448344010977 0.479938271604938

hsa-miR-1292 67.298245614035 39.4473684210526 1.70602623971536 0.534166829792566 0.371179974936714 0.9974938201385 0.426417079377219 0.996448344010977 0.558333333333333

hsa-miR-335 409.763157894737 343.684210526316 1.19226646248086 0.175856086011003 0.611063440441159 0.9974938201385 0.774503227138229 0.997772027551652 0.533333333333333

hsa-miR-301a 223.010964912281 227.035087719298 0.982275326481725 -0.0178836367255791 0.849220318191957 0.9974938201385 0.58896505583874 0.996448344010977 0.487345679012346

hsa-miR-526b 39.4385964912281 30.8070175438596 1.28018223234624 0.24700243681852 0.746075295952848 0.9974938201385 0.404082776589404 0.996448344010977 0.478703703703704

hsa-miR-374a 494.798245614035 517.730263157895 0.955706631086261 -0.0453042842740332 0.714393448873095 0.9974938201385 0.286505053998033 0.996448344010977 0.475925925925926

hsa-miR-628-5p 70.4912280701754 90.0350877192982 0.782930631332813 -0.244711180360648 0.464121612511245 0.9974938201385 0.425704285091361 0.996448344010977 0.452160493827161

hsa-miR-1183 177.114035087719 145.563596491228 1.21674676469259 0.196180710753238 0.284933164055791 0.9974938201385 0.295304890470107 0.996448344010977 0.569753086419753

hsa-miR-519d 57.1578947368421 50.280701754386 1.13677599441731 0.128196180773873 0.614277653400773 0.9974938201385 0.224133159314614 0.996448344010977 0.466975308641975

hsa-miR-130b 828.118421052631 890.081140350877 0.930385313777326 -0.072156462750366 0.493683391219394 0.9974938201385 0.612262777944767 0.996448344010977 0.455246913580247

hsa-miR-29b 298.953947368421 319.333333333333 0.936181463575431 -0.0659459499538921 0.958307951678229 0.999511784184894 0.882622834078239 0.999654245801439 0.496296296296296

hsa-miR-380 12.0701754385965 1 12.0701754385965 2.49073757009879 0.265180254978567 0.9974938201385 0.338422559351676 0.996448344010977 0.568518518518519

hsa-miR-296-5p 296.605263157895 376.77850877193 0.787213857087148 -0.239255330391976 0.797445532677723 0.9974938201385 0.905316049733739 0.999654245801439 0.483024691358025

hsa-miR-1274a 109.074561403509 84.0964912280702 1.29701679357463 0.260066853264848 0.229190584947448 0.9974938201385 0.285831426037001 0.996448344010977 0.578395061728395

hsa-miR-16 17675.4912280702 17675.4912280702 1 0 0.458397837792704 0.9974938201385 0.337320905922032 0.996448344010977 0.547530864197531

hsa-miR-150 2005.4298245614 2106.82894736842 0.951871212452406 -0.0493255343794651 0.651446246611078 0.9974938201385 0.582665381765299 0.996448344010977 0.47037037037037

hsa-miR-519aS 126.916666666667 89.9166666666667 1.4114921223355 0.344647387637027 0.169604481058288 0.9974938201385 0.169780543500642 0.996448344010977 0.589506172839506

hsa-miR-548c-5p 18.4912280701754 21.8377192982456 0.846756376782486 -0.166342256420985 0.9884088305038 1 0.874309380937517 0.999654245801439 0.501234567901235

hsa-miR-367 46.9824561403509 65.7456140350877 0.714609739826551 -0.336018703725634 0.127972740519464 0.9974938201385 0.133983604076358 0.996448344010977 0.400925925925926

hsa-miR-623 71.0701754385965 57.8245614035088 1.22906553398058 0.206254152173328 0.381796754885215 0.9974938201385 0.209097217888734 0.996448344010977 0.557098765432099

hsa-miR-518a-3p 31.7543859649123 40.9649122807017 0.775160599571735 -0.254685045843126 0.749789092429983 0.9974938201385 0.9285834900393 0.999654245801439 0.479012345679012

hsa-miR-220a 51.1578947368421 62.6228070175438 0.816921137414204 -0.202212715813966 0.654415331809898 0.9974938201385 0.753271114424164 0.997772027551652 0.470679012345679

hsa-miR-19aS 57.9649122807017 52.1578947368421 1.1113353514968 0.105562311642771 0.74281271544924 0.9974938201385 0.84498897865602 0.999654245801439 0.521604938271605

hsa-miR-218 24.5087719298246 42.3508771929824 0.57870753935377 -0.546958042400514 0.755011077988787 0.9974938201385 0.90679476578516 0.999654245801439 0.47962962962963

hsa-miR-214S 72.8157894736842 58.7456140350877 1.2395102284605 0.214716324544985 0.875266839577125 0.9974938201385 0.998084786347815 0.999654245801439 0.510493827160494

hsa-miR-544 36.280701754386 55.3859649122807 0.655052264808362 -0.423040252929672 0.22620883652083 0.9974938201385 0.352211570279861 0.996448344010977 0.421296296296296

hsa-miR-574-5p 1470.35964912281 1567.56798245614 0.937987803769108 -0.0640183324387934 0.397489004195295 0.9974938201385 0.19870409574499 0.996448344010977 0.555246913580247

hsa-miR-891a 91.4035087719298 103.90350877193 0.879696074292951 -0.128178801285528 0.594295801502551 0.9974938201385 0.782313904667368 0.997772027551652 0.465123456790123

hsa-miR-936 60.8070175438596 56.0701754385965 1.08448060075094 0.0811011633893857 0.546098529196229 0.9974938201385 0.357300088290381 0.996448344010977 0.539506172839506

hsa-miR-193a-3p 174.410087719298 148.558114035088 1.17401926412518 0.16043313023651 0.39495762964411 0.9974938201385 0.41433746723728 0.996448344010977 0.555555555555556

hsa-miR-193bS 53.6666666666667 51.4385964912281 1.04331514324693 0.0424032811443612 0.91285540550448 0.9974938201385 0.980798638469327 0.999654245801439 0.507407407407407

hsa-miR-1297 37.7543859649123 25.7192982456141 1.46793997271487 0.383860038835077 0.300563959467895 0.9974938201385 0.598520511860744 0.996448344010977 0.567283950617284

hsa-miR-1226 88.9122807017544 93.7017543859649 0.948885976408913 -0.0524666388987402 0.351626193213711 0.9974938201385 0.254058373144349 0.996448344010977 0.439197530864198

hsa-miR-1262 4.42105263157895 5.15679824561404 0.857325111630873 -0.153938072193845 0.769093071618314 0.9974938201385 0.0942894671309664 0.996448344010977 0.518518518518518

hsa-miR-1288 87.9649122807018 84.8870614035088 1.0362581861865 0.0356163272542553 0.823253005676711 0.9974938201385 0.825130578160996 0.997772027551652 0.514814814814815

hsa-miR-134 23.6491228070176 56.0438596491228 0.421975269995305 -0.862808568570145 0.145346563737814 0.9974938201385 0.681062278522444 0.996448344010977 0.405555555555556

hsa-miR-411S 45.8771929824562 58.578947368421 0.783168613357293 -0.24440726344463 0.366454973396895 0.9974938201385 0.639821529655379 0.996448344010977 0.441358024691358

hsa-miR-302dS 60.0350877192982 71.2828947368421 0.842208890939855 -0.171727206506595 0.882866682001115 0.9974938201385 0.984699048708725 0.999654245801439 0.509876543209877

hsa-miR-195 741.741228070176 729.178728070176 1.0172282864494 0.0170815623284185 0.882845661750662 0.9974938201385 0.721125707885955 0.997772027551652 0.509876543209877

hsa-miR-138-1S 110.530701754386 115.202850877193 0.959444153619188 -0.0414011688560147 0.75017859474973 0.9974938201385 0.511735776587174 0.996448344010977 0.520987654320988

hsa-miR-223S 62.563596491228 76.8421052631579 0.814183789954338 -0.205569152280771 0.867684346398053 0.9974938201385 0.825339066688107 0.997772027551652 0.488888888888889

hsa-miR-744S 47.7543859649123 56.9473684210526 0.838570548367221 -0.176056564863125 0.965829154773666 0.999511784184894 0.813947934055719 0.997772027551652 0.503086419753086

hsa-miR-892b 40.9824561403509 62.1578947368421 0.659328252893029 -0.416533760917354 0.356461913473112 0.9974938201385 0.47777510984273 0.996448344010977 0.439814814814815

hsa-miR-578 20.859649122807 6.54385964912282 3.18766756032171 1.15928947704697 0.393336428902454 0.9974938201385 0.763215873603214 0.997772027551652 0.554320987654321

hsa-miR-149S 396.359649122807 348.631578947368 1.13690116747182 0.128306287042906 0.225559574285097 0.9974938201385 0.229013989567856 0.996448344010977 0.579012345679012

hsa-miR-1200 69.8245614035087 71.9495614035087 0.970465421073486 -0.0299795070197003 0.383714238647246 0.9974938201385 0.346555909095125 0.996448344010977 0.44320987654321

hsa-miR-1301 130.570175438597 147.128289473684 0.887457985854929 -0.119394098634904 0.834358289751654 0.9974938201385 0.590106825930664 0.996448344010977 0.513888888888889

hsa-miR-26b 599.291666666667 530.910087719298 1.12880067742002 0.121155721680331 0.631159252479193 0.9974938201385 0.744965890507533 0.997772027551652 0.531481481481481

hsa-let-7iS 191.956140350877 162.741228070175 1.1795175852311 0.165105528810071 0.696749125241714 0.9974938201385 0.823725368742852 0.997772027551652 0.525617283950617

hsa-miR-1256 34.2105263157895 35.5877192982456 0.96130145427656 -0.0394672310887089 0.677872580997637 0.9974938201385 0.917670169483065 0.999654245801439 0.52716049382716

hsa-miR-518fS 121.90350877193 89.9210526315789 1.35567261730563 0.304297727703181 0.172467665349604 0.9974938201385 0.212582366369874 0.996448344010977 0.588888888888889

hsa-miR-548b-3p 42.1052631578947 32.1491228070175 1.30968622100955 0.269787582575231 0.167279159126189 0.9974938201385 0.119694308388472 0.996448344010977 0.589814814814815

hsa-miR-675 158.956140350877 160.973684210526 0.987466623072312 -0.0126125821985168 0.841775917621312 0.9974938201385 0.426392738258024 0.996448344010977 0.486728395061728

hsa-miR-576-5p 28.6842105263158 5.87500000000001 4.88241881298991 1.58564075546658 0.521687867485331 0.9974938201385 0.702229874142078 0.996448344010977 0.540740740740741

hsa-miR-32 156.921052631579 153.543311403509 1.02199862173868 0.0217601431883142 0.558862107514672 0.9974938201385 0.546887561068165 0.996448344010977 0.538271604938272

hsa-miR-187 34.2105263157895 14.5087719298246 2.35792019347038 0.857779956534101 0.236409038761277 0.9974938201385 0.397468646173909 0.996448344010977 0.575925925925926

hsa-miR-135aS 16.9473684210526 21.5723684210526 0.785605367490089 -0.241300689620404 0.731294944894003 0.9974938201385 0.758215646553487 0.997772027551652 0.478086419753086

hsa-miR-524-5p 51.8947368421053 49.3552631578947 1.0514529458811 0.0501729656406387 0.348842200622344 0.9974938201385 0.213928163813376 0.996448344010977 0.438888888888889

hsa-miR-188-3p 132.947368421053 152.929824561403 0.869335780658484 -0.140025829433029 0.490753882675719 0.9974938201385 0.769988786158302 0.997772027551652 0.454938271604938

hsa-miR-557 31.7543859649123 23.8947368421053 1.32892804698972 0.284372637550413 0.635829481017336 0.9974938201385 0.708978043551124 0.996448344010977 0.530864197530864

hsa-miR-512-3p 1 19.2280701754386 0.052007299270073 -2.95637119967341 0.145096828425653 0.9974938201385 0.567274589487208 0.996448344010977 0.410493827160494

hsa-miR-302aS 44.421052631579 30.3245614035088 1.46485391958345 0.381755523902388 0.239332665392164 0.9974938201385 0.431481424244438 0.996448344010977 0.576234567901235

hsa-miR-30e 144.28947368421 233.176535087719 0.618799287114931 -0.47997431232828 0.732217725678313 0.9974938201385 0.538834031995758 0.996448344010977 0.477469135802469

hsa-miR-520a-5p 96.719298245614 69.9912280701754 1.38187742824915 0.323443029841348 0.18961868997839 0.9974938201385 0.661910621913431 0.996448344010977 0.585493827160494

hsa-miR-203 40.2105263157895 32.780701754386 1.22665239496923 0.204288828909091 0.815168906278076 0.9974938201385 0.964219900170135 0.999654245801439 0.515432098765432

hsa-miR-516bS 46.7719298245614 49.7763157894737 0.939642259229888 -0.0622560514425942 0.144445941502113 0.9974938201385 0.15570308058441 0.996448344010977 0.595061728395062

hsa-miR-1272 153.228070175439 139.383771929825 1.09932503658019 0.0946963883639702 0.696765398373808 0.9974938201385 0.538656669488474 0.996448344010977 0.525617283950617

hsa-miR-1225-3p 123.482456140351 177.552631578947 0.695469591423349 -0.363167990467569 0.114611989301496 0.9974938201385 0.0375556647807343 0.996448344010977 0.397222222222222

hsa-miR-7-1S 287.973684210526 260.506578947368 1.10543728060207 0.100240985741269 0.812149656322202 0.9974938201385 0.9719077752466 0.999654245801439 0.515740740740741

hsa-miR-1305 87.9649122807018 70.7105263157894 1.24401439027416 0.218343561994584 0.864145079250274 0.9974938201385 0.418219312810747 0.996448344010977 0.51141975308642

hsa-miR-513a-5p 52.1929824561403 36.0175438596491 1.44909887968826 0.370941900960797 0.0835618315841057 0.9974938201385 0.0228041380572807 0.996448344010977 0.612654320987654

hsa-let-7cS 12.0701754385965 14.6140350877193 0.825930372148859 -0.191244804233499 0.855509146715861 0.9974938201385 0.541686299962901 0.996448344010977 0.488271604938272

hsa-miR-509-5p 202.464912280702 185.18201754386 1.09332922799995 0.0892273788428009 0.992416288891977 1 0.642010440639598 0.996448344010977 0.499074074074074

hsa-miR-124S 87.9649122807018 92.0526315789474 0.955593672574805 -0.0454224849684729 0.924268535954185 0.9974938201385 0.496567763906147 0.996448344010977 0.493518518518519

hsa-miR-627 163.745614035088 133.412280701754 1.22736537576435 0.204869901160228 0.475854780090577 0.9974938201385 0.89291731944652 0.999654245801439 0.546604938271605

hsa-miR-1285 202.868421052632 226.622807017544 0.89518095606735 -0.110729395593913 0.325206825714814 0.9974938201385 0.39802593244314 0.996448344010977 0.435802469135802

hsa-miR-577 21.5043859649123 35.5964912280702 0.604115327747659 -0.503990159294471 0.521748701577107 0.9974938201385 0.370970442422317 0.996448344010977 0.458950617283951

hsa-miR-1261 2.87719298245614 1 2.87719298245614 1.05681515998965 0.699135613100112 0.9974938201385 0.749524551839049 0.997772027551652 0.523456790123457

hsa-miR-625S 233.517543859649 220.017543859649 1.0613587433219 0.0595499205995782 0.614403683588303 0.9974938201385 0.25293578871854 0.996448344010977 0.533024691358025

hsa-miR-29cS 1 9.40350877192984 0.106343283582089 -2.24108289323625 0.768844530369645 0.9974938201385 0.917080868221003 0.999654245801439 0.48179012345679

hsa-miR-147b 32.2631578947368 36.1578947368421 0.892285298398836 -0.113969356286138 0.760279915718326 0.9974938201385 0.71204453400006 0.996448344010977 0.479938271604938

hsa-miR-25 6386.57456140351 5591.87061403509 1.14211772807722 0.132884195305076 0.58097451135576 0.9974938201385 0.675971012429806 0.996448344010977 0.536111111111111

hsa-miR-873 40.8947368421052 37.1425438596491 1.10102143236701 0.0962383238244714 0.751979640605283 0.9974938201385 0.313913226014966 0.996448344010977 0.520679012345679

hsa-miR-1275 83.671052631579 117.684210526316 0.710979427549195 -0.341111784127472 0.337007884303741 0.9974938201385 0.886028721395252 0.999654245801439 0.437345679012346

hsa-miR-548g 47.6491228070176 30.5263157894737 1.56091954022989 0.445275096470012 0.266661371042677 0.9974938201385 0.506467872847606 0.996448344010977 0.572222222222222

hsa-miR-181a-2S 84.3552631578948 93.4035087719298 0.903127347858753 -0.101891707979525 0.302399784594337 0.9974938201385 0.209513841145006 0.996448344010977 0.432716049382716

hsa-miR-24 1405.51754385965 1470.35964912281 0.955900513658789 -0.0451014365563667 0.996205765706804 1 0.587943321751695 0.996448344010977 0.499382716049383

hsa-miR-16-2S 80.8771929824561 66.8508771929824 1.20981498491012 0.190467442884523 0.187500481016884 0.9974938201385 0.331613859651802 0.996448344010977 0.585802469135802

hsa-miR-630 59.5438596491228 63.1589912280701 0.942761410392181 -0.0589420396104383 0.749787722547563 0.9974938201385 0.510910074146954 0.996448344010977 0.479012345679012

hsa-miR-491-5p 79.0877192982456 94.7719298245614 0.834505738615328 -0.180915659192894 0.581410514575764 0.9974938201385 0.821210149667077 0.997772027551652 0.463888888888889

hsa-miR-618 24.2631578947368 20.5438596491228 1.18104184457728 0.166396968067041 0.810556452679258 0.9974938201385 0.550600274186835 0.996448344010977 0.515740740740741

hsa-miR-598 67.719298245614 79.5109649122807 0.851697603221446 -0.160523740950314 0.140509991738599 0.9974938201385 0.139131029882948 0.996448344010977 0.404012345679012

hsa-miR-146a 119.719298245614 129.922149122807 0.921469503498215 -0.0817855968331138 0.458448315142806 0.9974938201385 0.394618336079138 0.996448344010977 0.451543209876543

hsa-miR-338-3p 115.087719298246 90.1447368421053 1.27669926531407 0.244278048387017 0.291393564672822 0.9974938201385 0.266777952926769 0.996448344010977 0.568827160493827

hsa-miR-1238 16.140350877193 15.1929824561404 1.06235565819861 0.0604887614806507 0.930383636046512 0.9974938201385 0.655983945671691 0.996448344010977 0.494135802469136

hsa-miR-96 171.043859649123 184.447368421053 0.927331526133067 -0.0754441439828475 0.473005927350754 0.9974938201385 0.201308661768039 0.996448344010977 0.453086419753086

hsa-miR-542-3p 74.578947368421 83.3103070175439 0.895194724858184 -0.110714014698182 0.306850280003582 0.9974938201385 0.540004782183602 0.996448344010977 0.433333333333333

hsa-miR-491-3p 57.578947368421 39.9210526315789 1.44232036914964 0.36625318419334 0.717112988038565 0.9974938201385 0.797330132849412 0.997772027551652 0.523765432098765

hsa-miR-938 96.4210526315789 93.0175438596491 1.03658996605055 0.0359364470532143 0.648129525343413 0.9974938201385 0.683961955100342 0.996448344010977 0.529938271604938

hsa-miR-101 615.030701754386 638.912280701754 0.962621505848756 -0.0380949809449124 0.916722633673327 0.9974938201385 0.836502086677617 0.999654245801439 0.492901234567901

hsa-miR-22 6386.57456140351 6538.67763157895 0.976737946302652 -0.023536885739789 0.789320731040506 0.9974938201385 0.48925564216987 0.996448344010977 0.517592592592593

hsa-miR-302f 1 1 1 0 0.686283669997197 0.9974938201385 0.609242457201429 0.996448344010977 0.523456790123457

hsa-miR-382 41.8771929824561 46.0877192982456 0.908641035401598 -0.0958051633467102 0.897342542600488 0.9974938201385 0.125121169105439 0.996448344010977 0.491358024691358

hsa-miR-135b 24.0175438596491 8.31578947368422 2.88818565400844 1.06062850359337 0.308000152477215 0.9974938201385 0.354153725032276 0.996448344010977 0.564814814814815

hsa-miR-708S 72.0526315789473 67.4561403508771 1.06814044213264 0.0659192320168143 0.429965198600825 0.9974938201385 0.338625903302325 0.996448344010977 0.448456790123457

hsa-miR-196a 15.8771929824561 2.32236842105263 6.83663833805477 1.92229613977836 0.777682219578678 0.9974938201385 0.791863060051532 0.997772027551652 0.517592592592593

hsa-miR-122S 74.0175438596491 64.9934210526315 1.13884671188042 0.130016094091591 0.996207823255754 1 0.935734992911567 0.999654245801439 0.500617283950617

hsa-miR-521 74.0175438596491 82.3004385964912 0.899357829944842 -0.106074292619513 0.725087282341016 0.9974938201385 0.57024539178087 0.996448344010977 0.476851851851852

hsa-miR-125b-2S 47.5438596491228 55.1754385964912 0.861685214626391 -0.148865255260794 0.778184063158551 0.9974938201385 0.572983232957373 0.996448344010977 0.481481481481481

hsa-miR-25S 74.7894736842105 37.8859649122807 1.9740680713128 0.680096424364454 0.0464180172867803 0.9974938201385 0.0526743233993582 0.996448344010977 0.62962962962963

hsa-miR-373 12.7478070175439 3.40241228070175 3.7466967450854 1.32087458381292 0.702737349769745 0.9974938201385 0.421929363575344 0.996448344010977 0.524074074074074

hsa-miR-892a 12.0701754385965 1 12.0701754385965 2.49073757009879 0.527457693241172 0.9974938201385 0.950444929513491 0.999654245801439 0.539506172839506

hsa-miR-133a 87.9122807017544 88.390350877193 0.994591375973801 -0.0054233035878738 0.562056227224605 0.9974938201385 0.260381390120528 0.996448344010977 0.462037037037037

hsa-miR-1323 36.3859649122807 26.0350877192983 1.39757412398922 0.334737965062146 0.186011466179104 0.9974938201385 0.526577277590093 0.996448344010977 0.585802469135802

hsa-miR-549 62.3552631578947 62.1754385964912 1.00289221218962 0.00288803779083555 0.418932937842885 0.9974938201385 0.632859344269115 0.996448344010977 0.552777777777778

hsa-miR-593 4.70175438596492 8.67543859649124 0.541961577350859 -0.61256017055191 0.715320629705475 0.9974938201385 0.265055426009381 0.996448344010977 0.522839506172839

hsa-miR-33a 81.4385964912281 80.0701754385965 1.0170902716915 0.0169458758524163 0.358951821208715 0.9974938201385 0.345599516616233 0.996448344010977 0.440123456790123

hsa-miR-202 1 8.78947368421054 0.11377245508982 -2.17355483325032 0.345958209533739 0.9974938201385 0.985533536085722 0.999654245801439 0.442283950617284

hsa-miR-146b-3p 23.6491228070176 11.4100877192982 2.07265039400346 0.728828171861785 0.256989955913496 0.9974938201385 0.365792984118934 0.996448344010977 0.57283950617284

hsa-miR-154 1 1 1 0 0.55579870531734 0.9974938201385 0.128843777798769 0.996448344010977 0.535802469135803

hsa-miR-10a 21.8026315789474 28.9912280701754 0.752042360060514 -0.284962626751059 0.394046388035678 0.9974938201385 0.666867669439952 0.996448344010977 0.445061728395062

hsa-miR-1913 308.570175438596 334.719298245614 0.921877456889774 -0.0813429743567315 0.797454484189021 0.9974938201385 0.694235776974818 0.996448344010977 0.483024691358025

hsa-miR-505S 136.97149122807 152.523026315789 0.898038116188957 -0.10754276593601 0.400236310644227 0.9974938201385 0.577612896964962 0.996448344010977 0.445061728395062

hsa-miR-127-5p 117.684210526316 106.300438596491 1.107090545252 0.101735443743842 0.631203594975085 0.9974938201385 0.982712087973626 0.999654245801439 0.531481481481481

hsa-miR-548b-5p 1 11.2894736842105 0.0885780885780886 -2.42387075920163 0.939715859488368 0.9974938201385 0.552515993929781 0.996448344010977 0.504938271604938

hsa-miR-514 34.8421052631579 64.1315789473684 0.543290931473123 -0.610110317128252 0.113725048576551 0.9974938201385 0.318808200379588 0.996448344010977 0.397530864197531

hsa-miR-297 74.9824561403508 80.7543859649123 0.928524875081468 -0.0741581079381489 0.771892801639969 0.9974938201385 0.298726510236298 0.996448344010977 0.519135802469136

hsa-miR-532-3p 3004.33333333333 2809.70394736842 1.06927042478878 0.0669765699193183 0.801029098099515 0.9974938201385 0.752307585969301 0.997772027551652 0.516666666666667

hsa-miR-33b 168.885964912281 145.359649122807 1.16184901333655 0.150012713099542 0.581331536142502 0.9974938201385 0.546289823474688 0.996448344010977 0.536111111111111

hsa-miR-942 45.561403508772 49.7017543859649 0.916696081891988 -0.0869792881677675 0.577220370091176 0.9974938201385 0.889765277915869 0.999654245801439 0.46358024691358

hsa-miR-139-3p 87.5789473684211 79.2631578947368 1.10491367861886 0.0997672130213153 0.527161645543136 0.9974938201385 0.504967334984853 0.996448344010977 0.541358024691358

hsa-miR-766 487.219298245614 393.155701754386 1.23925278476564 0.214508605053923 0.841778562869755 0.9974938201385 0.506680311872577 0.996448344010977 0.513271604938272

hsa-miR-21S 59.5438596491228 77.4923245614035 0.76838396558799 -0.263465715577727 0.82317246878165 0.9974938201385 0.80376462454222 0.997772027551652 0.485185185185185

hsa-miR-492 70.5087719298245 73.7017543859649 0.956676981671031 -0.0442894767201809 0.363820525929333 0.9974938201385 0.168487415088333 0.996448344010977 0.440740740740741

hsa-miR-1208 82.0175438596491 83.5986842105263 0.981086540227171 -0.0190946069700209 0.555650157885448 0.9974938201385 0.242372346800727 0.996448344010977 0.53858024691358

hsa-miR-519e 1 1.19298245614035 0.838235294117649 -0.176456437341554 0.19258192369534 0.9974938201385 0.262859530400511 0.996448344010977 0.424382716049383

hsa-miR-652 1030.54385964912 990.375 1.04055924235681 0.0397583016620731 0.306680858858653 0.9974938201385 0.261756550654308 0.996448344010977 0.566666666666667

hsa-miR-425S 48.8771929824561 52.3859649122807 0.933020763563296 -0.0693278237593844 0.457917174626762 0.9974938201385 0.578208942203516 0.996448344010977 0.451543209876543

hsa-miR-1276 46.7719298245614 52.578947368421 0.889556222889557 -0.117032566578009 0.981030008288512 1 0.778199791090346 0.997772027551652 0.501851851851852

hsa-miR-933 174.039473684211 154.320175438596 1.12778172516697 0.120252628293508 0.467146237702991 0.9974938201385 0.352265857537621 0.996448344010977 0.547530864197531

hsa-miR-586 39.6666666666667 39.0350877192982 1.0161797752809 0.0160502776776296 0.741367137564191 0.9974938201385 0.667749320007005 0.996448344010977 0.478395061728395

hsa-miR-27b 188.745614035088 171.426535087719 1.10102916061686 0.0962453429636994 0.229224766088861 0.9974938201385 0.238723280182432 0.996448344010977 0.578395061728395

hsa-miR-523 116.5 126.394736842105 0.921715594420154 -0.0815185689326837 0.334662398916597 0.9974938201385 0.387021618983517 0.996448344010977 0.437037037037037

hsa-miR-1280 3467.72368421053 3835.53947368421 0.904103244928834 -0.100811716119238 0.511758336703864 0.9974938201385 0.70674629891135 0.996448344010977 0.457098765432099

hsa-miR-663 289.342105263158 279.324561403509 1.03586345507647 0.0352353350316537 0.764632461698401 0.9974938201385 0.633004639568638 0.996448344010977 0.480246913580247

hsa-miR-345 84.5855263157895 101.600877192982 0.832527519965465 -0.183289000658335 0.408246310866578 0.9974938201385 0.798365349212967 0.997772027551652 0.445987654320988

hsa-miR-661 45.2631578947369 62.3881578947368 0.725508805230413 -0.320882069956337 0.257809611218107 0.9974938201385 0.98577264393482 0.999654245801439 0.426234567901235

hsa-miR-519eS 35.3508771929824 25.0263157894737 1.41254819488258 0.345395303727228 0.900196571293204 0.9974938201385 0.285739906163457 0.996448344010977 0.508333333333333

hsa-miR-658 60.6842105263158 41.3245614035088 1.46847803014222 0.384226510797073 0.223713955163777 0.9974938201385 0.184782385319319 0.996448344010977 0.579320987654321

hsa-miR-185S 40.2807017543859 40.3947368421052 0.997176981541802 -0.00282701069000492 0.93093040747511 0.9974938201385 0.87999096858525 0.999654245801439 0.505864197530864

hsa-miR-1298 52.5263157894737 54.1239035087719 0.970482769798019 -0.0299616304739843 0.568372825276834 0.9974938201385 0.435457560207857 0.996448344010977 0.462654320987654

hsa-miR-548e 42.1052631578947 66.6052631578947 0.632161201106282 -0.458610852317251 0.0180396163060868 0.9974938201385 0.440800467094721 0.996448344010977 0.346296296296296

hsa-miR-324-5p 302.114035087719 287.412280701754 1.05115214405616 0.0498868426511788 1 1 0.923472949048198 0.999654245801439 0.500308641975309

hsa-miR-1244 1 1 1 0 0.728397075244116 0.9974938201385 0.985170643878916 0.999654245801439 0.480555555555556

hsa-miR-376a 96.3508771929824 106.22149122807 0.907075169808204 -0.0975299548940822 0.962061306318837 0.999511784184894 0.611720387050911 0.996448344010977 0.503395061728395

hsa-miR-516a-3p 16.140350877193 20.3157894736842 0.79447322970639 -0.230075988089854 0.81867523398383 0.9974938201385 0.931799542097247 0.999654245801439 0.514814814814815

hsa-miR-455-5p 28.6842105263158 30.9122807017544 0.927922814982974 -0.0748067231647709 0.315718220430459 0.9974938201385 0.148781651227799 0.996448344010977 0.435185185185185

hsa-miR-548d-5p 14.8070175438597 27.8333333333333 0.531988654270407 -0.631133116427293 0.258463960046442 0.9974938201385 0.353009405007502 0.996448344010977 0.429320987654321

hsa-miR-1229 198.359649122807 210.291666666667 0.943259674845922 -0.0584136632638182 0.714394991002668 0.9974938201385 0.571720612342388 0.996448344010977 0.475925925925926

hsa-miR-150S 89.2280701754386 50.1929824561403 1.77770010485844 0.575320452931955 0.172312447269719 0.9974938201385 0.23448165243568 0.996448344010977 0.588888888888889

hsa-let-7f-2S 1 8.4561403508772 0.118257261410788 -2.13489284621605 0.0391921073079844 0.9974938201385 0.165200038083958 0.996448344010977 0.377777777777778

hsa-miR-606 81.4385964912281 88.4561403508772 0.920666402221341 -0.082657520913483 0.173965579202604 0.9974938201385 0.0635979481581621 0.996448344010977 0.41141975308642

**Values normal vs. pancreatitis in blood**

name median g1 median g2 qmedian logqmedian wmw_rawp wmw_adjp ttest_rawp ttest_adjp AUC

hsa-miR-579 39.2631578947368 30.6666666666667 1.28032036613272 0.247110332656389 0.278022284993281 0.477954645317134 0.965290951506057 0.982365673525622 0.575757575757576

hsa-miR-663b 73.5964912280701 84.7258771929825 0.868642422673741 -0.140823719786543 0.13005648507867 0.303487100183256 0.0294668550916909 0.127788421829795 0.393518518518519

hsa-miR-106b 14207.2368421053 8807.11184210526 1.61315503842962 0.478191912583307 0.00362895756288658 0.033845312920759 0.00398984982323603 0.0480398021371946 0.703703703703704

hsa-miR-449b 39.2631578947368 45.4035087719298 0.864760432766615 -0.145302766748921 0.247580711350177 0.445129487281672 0.0713503417608062 0.215298408879636 0.418771043771044

hsa-miR-561 44.7017543859649 24.4473684210526 1.82848941514173 0.603490169770051 0.242820592447494 0.440239855634847 0.485431184305867 0.663909844779656 0.581649831649832

hsa-miR-524-3p 15.7894736842105 20.0350877192983 0.788091068301225 -0.238141626891646 0.273725401589461 0.47539354880457 0.151591408354818 0.33257612491588 0.423821548821549

hsa-miR-655 31.8771929824561 44.719298245614 0.712828560219694 -0.338514335979944 0.649456054310267 0.796137180212728 0.20820963753788 0.407448791825829 0.468013468013468

hsa-miR-30c-2S 15.7894736842105 27.4824561403509 0.574529205234599 -0.554204347067936 0.16526259960602 0.347087231069668 0.31631069851999 0.519954538710003 0.40530303030303

hsa-miR-299-5p 27.140350877193 43.859649122807 0.6188 -0.479973160283226 0.0201080362315615 0.095134780701831 0.00612453899678627 0.0559838577610877 0.337542087542088

hsa-miR-369-5p 15.6315789473684 40.8508771929824 0.382649774532961 -0.960635134951622 0.0081661113993692 0.0558695127064355 0.00151143233790395 0.0343254238845028 0.316498316498317

hsa-miR-452 55.8947368421053 77.1578947368421 0.724420190995907 -0.322383680644712 0.354937085018275 0.557942995210877 0.227623622691691 0.437264792634766 0.43476430976431

hsa-miR-934 110.741228070175 80.0526315789473 1.38335524873986 0.324511887911559 0.00201966532679894 0.0232396156936998 0.0174779817663745 0.0954651788884885 0.716750841750842

hsa-miR-483-5p 168.44298245614 203.149122807017 0.82915929012479 -0.187342994988984 0.26883209018809 0.469639866057332 0.122820401415817 0.295529259377029 0.422138047138047

hsa-miR-340S 10.3684210526316 19.9298245614035 0.520246478873239 -0.653452581874998 0.0907302181871377 0.237273267562121 0.100960942552996 0.262437630792879 0.384259259259259

hsa-miR-340 169.236842105263 125.526315789474 1.3482180293501 0.298783742252476 0.0488305877040752 0.166241897837262 0.116227038887927 0.288229697012301 0.638468013468014

hsa-miR-608 64.9122807017543 155.80701754386 0.41661975002815 -0.875581343626188 1.8464668664874e-05 0.00199187613222328 0.000529078107724153 0.0228297203482972 0.19949494949495

hsa-miR-1539 96.4210526315789 97.0646929824561 0.993368954960858 -0.00665312809492623 0.918663458751132 0.957495851331192 0.9768296094413 0.987124066683655 0.492424242424242

hsa-miR-802 88.7368421052632 49.0526315789474 1.80901287553648 0.59278132387621 0.150690710710708 0.330483635323399 0.135420343366004 0.317575424795819 0.601010101010101

hsa-miR-654-3p 25.6666666666667 19.4342105263158 1.32069510268562 0.278158190940526 0.62516361370273 0.778522653139186 0.785776625459102 0.889652403868437 0.534511784511784

hsa-miR-507 24.0175438596491 25.3070175438597 0.949046793760832 -0.0522971730938061 0.214055772221237 0.401587242232451 0.0597972854327162 0.195473702001644 0.414141414141414

hsa-miR-637 3.08771929824561 18.5350877192983 0.16658778987222 -1.79223284201796 0.0312414242640308 0.126579103942998 0.00727166497949254 0.0614741901618286 0.352272727272727

hsa-miR-1228 233.517543859649 371.139254385965 0.629191175818884 -0.46332013234187 0.182376726027254 0.364331283707223 0.105695600523845 0.270204732241416 0.406144781144781

hsa-miR-626 29.8245614035088 35.1315789473684 0.848938826466917 -0.163768148898206 0.385093825944339 0.582024468984176 0.354972432016415 0.554965958025664 0.438973063973064

hsa-miR-151-5p 3921.35964912281 4726.70175438596 0.829618590909428 -0.186789212832098 0.39957280947826 0.60075145397167 0.533097807019547 0.703826631720299 0.440656565656566

hsa-miR-431S 11.8947368421053 16.5438596491228 0.71898197242842 -0.329918994693062 0.657688495691054 0.802807880878896 0.499328005428314 0.67648362430869 0.468855218855219

hsa-miR-29aS 1 1 1 0 0.0705640124441575 0.203105413703674 0.0109720241493789 0.0748948766909616 0.394781144781145

hsa-miR-532-5p 237.973684210526 150.811403508772 1.57795550385343 0.456130024214599 0.000451757089573871 0.010262112993706 0.00070385456294291 0.0253094369924888 0.746212121212121

hsa-miR-27a 317.587719298246 363.69298245614 0.873230264585997 -0.135555995509357 0.980832142988493 0.992330761311921 0.75479740283631 0.874349206238571 0.497895622895623

hsa-miR-556-5p 155.870614035088 88.0877192982456 1.76949312885879 0.570693137698239 0.00022083105203244 0.00794071657933316 0.000343193851293688 0.0185110183541533 0.759259259259259

hsa-miR-425 9651.24561403509 12702.7105263158 0.759778442092411 -0.274728411765631 0.0104632575226783 0.0654332698700826 0.0858642329414599 0.235240739772952 0.321127946127946

hsa-miR-615-5p 51.9649122807017 33.5964912280702 1.5467362924282 0.436147093212998 0.605198752461618 0.765862641390888 0.697290488045652 0.826595729647524 0.536616161616162

hsa-miR-519c-3p 46.5263157894737 32.4824561403509 1.43235214690791 0.359317950952086 0.197357190168286 0.378487233589401 0.260413764659838 0.468202247711333 0.589646464646465

hsa-miR-486-3p 289.342105263158 389.40350877193 0.743039286357902 -0.297006360348766 0.0405306564519809 0.149478446658374 0.0235212031537873 0.112942349983056 0.356060606060606

hsa-miR-525-3p 79.0877192982456 56.8421052631579 1.39135802469136 0.330280266373292 0.253683483574879 0.453268832971263 0.877834103934133 0.944602034532615 0.580387205387205

hsa-miR-140-5p 6.77192982456141 29.2982456140351 0.231137724550898 -1.46474153594597 0.160563111441596 0.343836141871209 0.0583977868546395 0.193418987440169 0.403619528619529

hsa-miR-20bS 84.9912280701755 77.4298245614035 1.09765492239719 0.0931760153647719 0.261357138961451 0.460308593721902 0.141448360192783 0.325383824558984 0.579124579124579

hsa-miR-526bS 3.08771929824561 10.7456140350877 0.28734693877551 -1.24706494738073 0.190166140750637 0.370459095864108 0.0216876988523294 0.106536193590243 0.412037037037037

hsa-miR-30bS 18.3684210526316 56.4210526315789 0.325559701492537 -1.12220941942832 0.0581206492673082 0.18042489322909 0.084061627348676 0.231140615020453 0.368686868686869

hsa-miR-136S 72.8157894736842 48.4561403508772 1.50271542360608 0.407273753925852 0.077390646421487 0.214064512377382 0.432368534728745 0.618796095308303 0.624158249158249

hsa-miR-1911 10 28.1315789473684 0.355472404115996 -1.03430765830461 0.266451274336035 0.466489161968094 0.292158733666272 0.498286535877457 0.422979797979798

hsa-miR-628-3p 178.149122807018 108.811403508772 1.63722842516829 0.493004827547624 0.0474600993203927 0.163179544675295 0.0381220824502829 0.147098541709968 0.639309764309764

hsa-miR-629S 92.5263157894737 155.311403508772 0.595747084239361 -0.517939057284065 0.0296824525011723 0.121402637481098 0.132238401482223 0.310958420924137 0.347222222222222

hsa-miR-603 178.5 155.620614035088 1.14702027817313 0.137167517304935 0.552091097463332 0.729639536157512 0.850877495903595 0.930207833296191 0.542087542087542

hsa-miR-518d-3p 98.4649122807017 49.4736842105263 1.99024822695035 0.68825936811835 0.00054059786573029 0.0115635881377831 0.000503274967411204 0.0228297203482972 0.742845117845118

hsa-miR-23b 2872.95175438596 3467.72368421053 0.828483471006436 -0.18815839281974 0.0148367514375656 0.0803650356664428 0.00352720072713626 0.0447643268752735 0.329124579124579

hsa-miR-23aS 1 14.4440789473684 0.0692325210658164 -2.6702845691555 0.11942102257472 0.287076162902462 0.29067572873467 0.49743015556513 0.396043771043771

hsa-miR-939 46.5964912280702 65.1688596491228 0.715011609516439 -0.335456499334021 0.182260850165214 0.364331283707223 0.0865333988724132 0.23557830670944 0.406144781144781

hsa-miR-662 53.2982456140351 58.4649122807017 0.911627906976744 -0.0925233688971453 0.596557076599915 0.764515778737737 0.24540724702514 0.456426715911051 0.462542087542088

hsa-miR-1178 19.9649122807018 28.6666666666667 0.696450428396573 -0.361758660733672 0.234805064368983 0.430226689066736 0.0675621269112112 0.210333188624078 0.417508417508417

hsa-miR-519b-5p 163.745614035088 85.9934210526316 1.90416443526381 0.644043295698826 4.89787775198985e-05 0.00352239041663937 3.01444137062566e-05 0.00835154206723173 0.78493265993266

hsa-miR-409-5p 93.7543859649123 91.1052631578947 1.02907760446755 0.0286628713721625 0.81479077652258 0.901492871973059 0.762197648417151 0.877597199200403 0.516835016835017

hsa-miR-567 61.859649122807 59.1666666666666 1.04551519644181 0.0445097748759046 0.576363941293783 0.750229383614683 0.265837383046162 0.47498480656074 0.46043771043771

hsa-miR-600 71.0701754385965 80.859649122807 0.878932523323931 -0.129047149517818 0.60967280954292 0.765862641390888 0.834508560646262 0.922656211440404 0.536195286195286

hsa-miR-1207-5p 357.271929824561 313.923245614035 1.13808688848682 0.129348684711115 0.241502281761128 0.438771514020744 0.277924644940002 0.488490771045258 0.582491582491583

hsa-miR-1291 136.513157894737 119.982456140351 1.13777599064191 0.129075471549022 0.493513534967381 0.680354921209025 0.862321653909503 0.936079984055222 0.548400673400673

hsa-miR-145 100.912280701754 136.890350877193 0.737175995642562 -0.304928615243245 0.674059251338221 0.810195546950309 0.251787063736439 0.456426715911051 0.470117845117845

hsa-miR-636 162.061403508772 129.879934210526 1.24777860794171 0.22136485672784 0.0538499778506802 0.175368041075989 0.0580959117238099 0.193418987440169 0.635521885521885

hsa-miR-541 132.684210526316 56.1151315789474 2.36449967758954 0.860566446747182 5.43567133430965e-06 0.0019173508627659 0.0255065781756787 0.117499875359226 0.819023569023569

hsa-miR-367S 80 62.8859649122807 1.27214395313154 0.240703629215505 0.674067674055993 0.810195546950309 0.516748167722019 0.691800554082699 0.529882154882155

hsa-miR-497 181.407894736842 179.936403508772 1.00817784061133 0.00814458326528367 0.336521290902248 0.535826335883099 0.711893821599281 0.837279562498664 0.567760942760943

hsa-miR-34aS 116.723684210526 119.501096491228 0.976758269486627 -0.0235160787540257 0.572347368199374 0.746126554012174 0.421528391423829 0.612422561950782 0.460016835016835

hsa-miR-126 1907.68201754386 2252.16447368421 0.847043828208147 -0.166002840440784 0.904325844878461 0.950588555578699 0.91834252694151 0.956006756031994 0.491161616161616

hsa-miR-1255a 42.0350877192982 65.9561403508771 0.637318792392606 -0.450485289582741 0.173522400619412 0.354857421171925 0.0545797798756052 0.186175296571728 0.404461279461279

hsa-miR-519b-3p 26.1228070175439 44.5438596491228 0.586451358802679 -0.533665549020669 0.106143662537853 0.264754680162801 0.0257328811621017 0.117499875359226 0.387205387205387

hsa-miR-377S 1 1 1 0 0.0235822237027596 0.103833974772865 0.0256567556396312 0.117499875359226 0.37037037037037

hsa-miR-187S 151.552631578947 151.996710526316 0.99707836474993 -0.00292591155757597 0.768521852530143 0.869245555351918 0.715285386657405 0.839852093449442 0.521043771043771

hsa-miR-1538 77.0175438596491 82.3530701754386 0.935211567651053 -0.0669824997272809 0.838104304193943 0.913324815248521 0.661336035054346 0.808403680243486 0.48526936026936

hsa-miR-331-5p 35.3508771929824 26.7719298245614 1.32044560943643 0.27796926253638 0.672563773247125 0.810195546950309 0.322782351421875 0.526580660259128 0.470117845117845

hsa-miR-219-1-3p 18.6315789473684 12.4967105263158 1.490918662806 0.399392482185931 0.995089657846209 0.999841688867204 0.386508757127308 0.583141708742774 0.500841750841751

hsa-miR-493S 39.2631578947368 22.4385964912281 1.74980453479281 0.559504087293027 0.482059273550199 0.667958204296826 0.675524768778169 0.815179424209318 0.549242424242424

hsa-miR-191S 59.5438596491228 42.4561403508772 1.40247933884297 0.338241626643439 0.275367984767528 0.476171056439897 0.559659287687757 0.723486268120741 0.576599326599327

hsa-miR-323-3p 41.8771929824561 32.4736842105263 1.28957320367369 0.254311313765333 0.612407703949446 0.768180012366819 0.583027138220516 0.744308314030037 0.535774410774411

hsa-miR-199a-5p 248.256578947368 179.474780701754 1.38323934971072 0.324428103298317 0.178460413759336 0.361091321429182 0.669863416631582 0.813069097824269 0.59469696969697

hsa-miR-653 32.7017543859649 46.280701754386 0.706595905989386 -0.347296338031723 0.0452382057845223 0.160966696034345 0.0282289215796052 0.124293670016323 0.359848484848485

hsa-miR-384 80.0350877192982 75.4912280701754 1.06019056472229 0.0584486700057338 0.330529440474296 0.531186046795749 0.554476471477861 0.722829599524765 0.568602693602694

hsa-miR-125b 498.324561403509 313.675438596491 1.5886629939316 0.462892778170853 0.163452999418048 0.344906058526738 0.894179573881414 0.948026697264837 0.598063973063973

hsa-miR-422a 133.280701754386 130.458333333333 1.0216342517104 0.0214035526902111 0.60966954323569 0.765862641390888 0.395072246535491 0.591515020638515 0.536195286195286

hsa-miR-431 199.447368421053 185.657894736842 1.07427356484763 0.0716446795251146 0.732068699993153 0.848028699163306 0.156153713959013 0.3358082323788 0.475589225589226

hsa-miR-769-3p 1 10.4912280701754 0.0953177257525083 -2.35053948611608 0.0318557224275171 0.128114014038856 0.00504117392090554 0.0538419241478691 0.36026936026936

hsa-miR-155S 80.0350877192982 66.6337719298245 1.20111897317756 0.183253599954714 0.410487146168352 0.607722250584458 0.596366074008903 0.756858708631887 0.558080808080808

hsa-miR-548n 33.4385964912281 27.2894736842105 1.22532947605272 0.203209767876455 0.966048767841941 0.984719894587695 0.477791552611919 0.659734575846538 0.496632996632997

hsa-miR-9S 112.078947368421 90.4583333333333 1.23901185483284 0.214314170666481 0.243894240055353 0.440336253489057 0.810200825370406 0.906499706166643 0.582070707070707

hsa-miR-1265 1 26.7719298245614 0.0373525557011795 -3.28735394400985 0.0242263149458386 0.105061858282707 0.158916134972225 0.340309241888412 0.349747474747475

hsa-miR-30c 1470.35964912281 2158.04166666667 0.681339786823459 -0.383694144517118 0.00163861844728541 0.0216814088946127 0.0147733946602992 0.0867308815771307 0.279040404040404

hsa-miR-15aS 129.394736842105 120.984649122807 1.06951367615871 0.067204036890741 0.485958993805975 0.67101217864729 0.485160279510652 0.663909844779656 0.549242424242424

hsa-miR-502-5p 12.4517543859649 15.5350877192982 0.801524562394128 -0.221239661869808 0.225347131975809 0.41912623899811 0.0510464563258331 0.177633434714492 0.416666666666667

hsa-miR-1227 77.9298245614035 45.2982456140351 1.72037180480248 0.542540433047784 0.109929630188075 0.270730290290341 0.164273626586739 0.34925017025842 0.612373737373737

hsa-miR-520f 1 1 1 0 0.0207486093054909 0.0967894585439927 0.0340931026525551 0.136848128321652 0.363636363636364

hsa-miR-1910 42.2105263157895 46.7894736842105 0.902137232845894 -0.10298862764739 0.643055893907306 0.793013699490819 0.475238156520034 0.658315455982005 0.467171717171717

hsa-miR-1182 9.92982456140352 9.44736842105264 1.05106778087279 0.0498065816067397 0.990039055032797 0.999302578354741 0.705743195408119 0.833182459148025 0.501262626262626

hsa-miR-622 98.1491228070175 95.5964912280701 1.02670214718297 0.0263518666646777 0.861719275197257 0.9226610590424 0.777267225632153 0.88713162664257 0.487373737373737

hsa-miR-361-5p 387.254385964912 374.833333333333 1.03313753481079 0.0326003224277572 0.909133745572341 0.952168327703228 0.904792684180934 0.949646635292392 0.491582491582492

hsa-miR-1248 24.8421052631579 38.9035087719298 0.638556933483653 -0.448544439930069 0.101412372108338 0.255903149501449 0.0227159460409477 0.110681447377901 0.38510101010101

hsa-miR-129-3p 86.0701754385965 89.3859649122807 0.962904808635918 -0.0378007208383905 0.890124819739483 0.942234945566877 0.779195565463579 0.88713162664257 0.51010101010101

hsa-miR-330-3p 360.043859649123 326.377192982456 1.10315263256914 0.098172110180256 0.256260308746379 0.456023486286352 0.532724106052973 0.703826631720299 0.57996632996633

hsa-miR-298 135.688596491228 93.7478070175439 1.44737888605581 0.369754255845782 0.142733958804748 0.319946510255837 0.180561902842431 0.369253370030849 0.603114478114478

hsa-miR-619 80.0350877192982 75.9824561403508 1.05333641191411 0.0519626616297066 0.759334277845635 0.865661138415829 0.337153591610869 0.541831563426779 0.478114478114478

hsa-miR-888 57.578947368421 68.859649122807 0.836178343949045 -0.178913358566472 0.168667944003424 0.350556892200509 0.24050395746912 0.451206337599675 0.403619528619529

hsa-miR-944 32.7017543859649 36.1666666666667 0.904195973805481 -0.100709156901418 0.170130545907466 0.352092712513533 0.164034850122893 0.34925017025842 0.404461279461279

hsa-miR-106a 9071.26754385965 7477.03728070175 1.21321684021459 0.193275377559052 0.0968102763481655 0.247799031998263 0.129641851425739 0.30652306241209 0.616582491582492

hsa-miR-506 43.3377192982456 59.1666666666666 0.732468495181616 -0.311334949006838 0.199267432505993 0.381303313198829 0.100791022497674 0.262437630792879 0.40993265993266

hsa-miR-200aS 91.0526315789474 73.2543859649123 1.24296491438151 0.21749958556665 0.536132472460137 0.718470773011667 0.632912540967156 0.780651180252918 0.543771043771044

hsa-miR-649 10.3684210526316 20.4298245614035 0.507513954486904 -0.678231071918298 0.377008573010367 0.574838159908033 0.333122970862203 0.537355371689872 0.438552188552189

hsa-miR-500 257.254385964912 170.461622807018 1.50916306983745 0.41155523878308 0.0165448714368307 0.0834983862572216 0.0130148488005167 0.0819840475536195 0.668350168350168

hsa-miR-505 10.6315789473684 27.7543859649123 0.383059418457649 -0.959565162258284 0.19621768323966 0.377140001416095 0.0668098508924615 0.208901816377516 0.411195286195286

hsa-miR-24-1S 107.157894736842 88.7543859649123 1.207353231864 0.188430552046587 0.361264472814553 0.563781627556888 0.731410511188569 0.854079937923934 0.564393939393939

hsa-miR-1908 599.291666666667 415.677631578947 1.44172219320598 0.365838366492627 0.18041865620862 0.362135994376879 0.137522375709567 0.319897062634383 0.594276094276094

hsa-miR-518f 49.4035087719298 45.8070175438597 1.07851397931827 0.0755841485788037 0.690818602670433 0.819551758407212 0.426087431555058 0.614905440521765 0.471801346801347

hsa-miR-548h 1 19.2982456140351 0.0518181818181818 -2.96001419095191 0.00375077253811251 0.0340728073725379 0.00880668058415062 0.0660883942967129 0.311447811447811

hsa-miR-423-5p 2106.82894736842 1503.35964912281 1.40141379249984 0.33748157884952 0.022042407496481 0.100118935102438 0.0268060115804982 0.120487437468593 0.660774410774411

hsa-miR-1185 10 14.6293859649123 0.683555688802279 -0.380447150201083 0.583882724396591 0.755458457502636 0.892224079286889 0.948026697264837 0.462121212121212

hsa-miR-1302 42.2105263157895 34.8508771929824 1.21117543418072 0.191591321279971 0.758382565561976 0.865661138415829 0.363690657782546 0.562352875240527 0.478114478114478

hsa-miR-18b 354.5 196.771929824561 1.80157810271041 0.588663004532361 0.00662760733694852 0.0484713994219201 0.0119442187413401 0.0792912367213575 0.690656565656566

hsa-miR-29b-1S 1 10.8070175438597 0.0925324675324675 -2.38019569569897 0.00398747783974196 0.0351142181193603 0.0201517372342963 0.102299701371751 0.311447811447811

hsa-miR-937 42.2456140350877 88.6710526315789 0.476430726616214 -0.741432946065724 0.00217766435241888 0.0244068095602272 0.00428535013662693 0.050357858827868 0.28493265993266

hsa-miR-573 52.9824561403509 39.7894736842105 1.331569664903 0.286358445521273 0.236680002949035 0.432743310476732 0.687982090232429 0.822338703421864 0.582912457912458

hsa-miR-339-3p 249.100877192982 283.394736842105 0.878989073575386 -0.128982811891414 0.373995399205715 0.571299816537464 0.149100181088309 0.331632619276315 0.437289562289562

hsa-miR-199b-5p 1 19.6381578947368 0.0509212730318258 -2.97747450498043 0.00979844416756644 0.0626374616045173 0.00320119815266352 0.0438513334245813 0.330808080808081

hsa-miR-93 4503.74342105263 3154.75 1.42760707537923 0.355999668757221 0.0537339060037975 0.175368041075989 0.0419993516233092 0.15760925839842 0.635521885521885

hsa-miR-148aS 36.6666666666667 30.4824561403509 1.20287769784173 0.184716767519909 0.421995686300484 0.616596713512132 0.96419624131059 0.982365673525622 0.556397306397306

hsa-miR-554 104.491228070175 100.822368421053 1.03638934203371 0.0357428860112137 0.11011162444022 0.270730290290341 0.049130486417586 0.173059631748476 0.612373737373737

hsa-miR-539 1 18.3684210526316 0.0544412607449857 -2.91063294303599 0.000325266129981522 0.00935682233913512 0.000294510004997125 0.0169441422875013 0.273569023569024

hsa-miR-1252 15.9649122807018 28.0263157894737 0.569640062597809 -0.562750586740794 0.145203364234297 0.324638609674088 0.0573553512915736 0.192597930601666 0.399410774410774

hsa-miR-664S 64.0877192982456 68.6140350877193 0.934032216824342 -0.0682443479602317 0.741131130005149 0.856219765989885 0.887016109743249 0.948026697264837 0.523569023569024

hsa-miR-520e 1 3.68092105263158 0.271671134941912 -1.3031630069057 0.0394210004714594 0.147914449595085 0.0269732174841211 0.120610811859049 0.367845117845118

hsa-miR-329 85.6052631578948 64.9473684210526 1.31807131280389 0.276169541448074 0.00961952935716968 0.0619526405614734 0.00805451289958354 0.0655758927579302 0.681818181818182

hsa-miR-452S 401.754385964912 263.642543859649 1.52386022408731 0.421246736580095 0.0973393648289818 0.247799031998263 0.766602823266996 0.879758293190715 0.616582491582492

hsa-miR-1468 1 1 1 0 0.0551441599423247 0.176866146894919 0.0132331614926394 0.0827552055662882 0.381313131313131

hsa-miR-519c-5p 147.864035087719 102.064692982456 1.44872855408614 0.370686312515588 0.032481066615982 0.129175854790749 0.0460151174291807 0.165555214145727 0.650252525252525

hsa-miR-210 708.070175438597 425.348684210526 1.66468171108327 0.50963394064359 0.00136502887268112 0.0206069475498116 0.00246726991262921 0.0400276533854518 0.724747474747475

hsa-let-7gS 145.563596491228 137.804824561404 1.05630261461831 0.054774711105025 0.824124394062469 0.906017125434733 0.409814847184245 0.603532786894204 0.484006734006734

hsa-let-7eS 2.45614035087718 10.4188596491228 0.235739844243316 -1.44502643689778 0.510329115666591 0.696857637373842 0.106140676975481 0.270204732241416 0.455808080808081

hsa-miR-300 55.8947368421053 26.1315789473684 2.13897280966767 0.760325718316656 0.0489286698153704 0.166241897837262 0.164710103470657 0.34925017025842 0.638047138047138

hsa-miR-1268 348.622807017544 542.842105263158 0.64221769762782 -0.442827939892949 0.00175130167488542 0.0216814088946127 0.00182315281674712 0.034907679426664 0.28030303030303

hsa-miR-484 5199.04385964912 5736.38157894737 0.90632810737865 -0.0983538890748744 0.889992942016711 0.942234945566877 0.91160847714898 0.951934643195463 0.51010101010101

hsa-miR-522 1 5.81578947368421 0.171945701357466 -1.76057654179137 0.075602107512944 0.211833177869061 0.0098870331098675 0.0706245759573506 0.385942760942761

hsa-miR-429 49.4035087719298 57.125 0.864831663403586 -0.145220399770986 0.174341618828599 0.355406925533536 0.0657129045843489 0.206971666628807 0.404882154882155

hsa-miR-497S 126.315789473684 131.995614035088 0.956969596278451 -0.043983657857834 0.687370475966103 0.818207890701719 0.785876867734263 0.889652403868437 0.471380471380471

hsa-miR-146b-5p 139.728070175439 156.763157894737 0.891332326114935 -0.115037940002159 0.407177951988751 0.607722250584458 0.201989376694233 0.399390697541133 0.441498316498317

hsa-miR-1273 103.59649122807 142.487938596491 0.727054459826548 -0.318753893901373 0.124122409840477 0.294279229923988 0.143669435990742 0.328095023184096 0.391835016835017

hsa-miR-107 1708.83771929825 1956.55592105263 0.873390686619827 -0.135372301349304 0.0616010788048015 0.187850639606161 0.0978103323556521 0.256566312531695 0.368686868686869

hsa-miR-92a-1S 14.1754385964912 42.140350877193 0.33638634471274 -1.08949494411883 0.0273919662827267 0.113834734915465 0.0944387042722865 0.252323844541744 0.346380471380471

hsa-miR-376aS 1 37.640350877193 0.0265672337450478 -3.62807663659703 0.00128877209950945 0.0206069475498116 0.00878991421061298 0.0660883942967129 0.284511784511784

hsa-miR-133b 8.17543859649124 35.7543859649123 0.22865554465162 -1.47553857965702 0.00196805219594529 0.0229517438527133 0.00182050473076854 0.034907679426664 0.285353535353535

hsa-miR-599 27.140350877193 49.8245614035088 0.544718309859155 -0.607486480582185 0.0335751708013615 0.13051969550259 0.0168241780194033 0.0930722155816988 0.351430976430976

hsa-miR-378 266.921052631579 135.798245614035 1.96557069956721 0.67578263550364 2.41514741166398e-05 0.0021131246582711 2.1844287538502e-05 0.00835154206723173 0.796296296296296

hsa-miR-34c-3p 116.179824561404 92.5438596491228 1.2554028436019 0.22745651199503 0.0388312843765769 0.146979817618359 0.149773383642289 0.332273599185849 0.644781144781145

hsa-miR-642 28.0701754385965 32.1578947368421 0.872885979268958 -0.135950339611832 0.338311365432183 0.537684545797374 0.385616486713172 0.582814409865967 0.432659932659933

hsa-miR-582-3p 89.2280701754386 65.9561403508771 1.35283947333422 0.302205697163984 0.133188883078594 0.307331567103816 0.189841439677214 0.383684221174322 0.605639730639731

hsa-miR-34a 19.4035087719298 47.8947368421053 0.405128205128205 -0.903551706096725 0.00553325048877189 0.042918418992642 0.00183063387573679 0.034907679426664 0.306397306397306

hsa-miR-337-5p 39.8245614035088 33.2105263157895 1.19915478077126 0.181616959266126 0.603964908525914 0.765862641390888 0.268294672971992 0.47838492308849 0.536616161616162

hsa-miR-616 15.4736842105263 11.3070175438596 1.36850271528317 0.313717233627549 0.547581680983641 0.726159689356536 0.975675604151012 0.987113770670954 0.541666666666667

hsa-miR-1304 62.3552631578947 77.7719298245614 0.801770809835326 -0.220932485229246 0.170850850567216 0.352737521625616 0.0252275138123673 0.117499875359226 0.403619528619529

hsa-miR-760 31.8771929824561 62.5021929824561 0.510017192379215 -0.673310843284473 0.0863364558883137 0.232113275487896 0.070022983333989 0.214324486475867 0.38047138047138

hsa-miR-1226S 191.956140350877 160.421052631579 1.1965769903762 0.179464972619768 0.200776337884753 0.383340662819783 0.451178518352095 0.635182808055234 0.59006734006734

hsa-miR-7-2S 15.4736842105263 25.859649122807 0.598371777476255 -0.513543016742491 0.179122047142363 0.361173660476307 0.250470079391364 0.456426715911051 0.406144781144781

hsa-miR-518cS 61.0175438596491 48.0350877192983 1.27027027027027 0.239229689065834 0.634466185774633 0.787516199717767 0.838195214101132 0.922656211440404 0.533670033670034

hsa-miR-132 125.894736842105 129.103618421053 0.97514491368877 -0.0251691896041693 0.838184534967357 0.913324815248521 0.855716589754872 0.932428556765725 0.48526936026936

hsa-miR-767-3p 42.2456140350877 34.140350877193 1.23741007194245 0.213020543682761 0.439155495246455 0.629553475743672 0.477203725279156 0.659734575846538 0.554292929292929

hsa-miR-1281 102.304824561404 148.129385964912 0.690645032347847 -0.370129288582158 0.180438560350009 0.362135994376879 0.183762984372657 0.374797538235181 0.405723905723906

hsa-miR-1914S 153.491228070175 154.381030701754 0.994236321473343 -0.00578035262202166 0.966463210562894 0.984719894587695 0.571369435011979 0.734356968187489 0.503367003367003

hsa-miR-550 135.688596491228 120.703947368421 1.12414382006141 0.1170216970774 0.284983799254581 0.483214554570394 0.97885935763058 0.988018275596714 0.5753367003367

hsa-miR-330-5p 8.10526315789475 24.2719298245614 0.333935670401157 -1.09680690814524 0.0905666791514238 0.237273267562121 0.0243364979483085 0.115397789721924 0.383838383838384

hsa-miR-616S 52.6315789473684 59.4473684210526 0.885347498893316 -0.121775056925921 0.64323243295895 0.793013699490819 0.434979452302745 0.620196236099349 0.532828282828283

hsa-miR-765 52.9824561403509 61.7478070175438 0.85804595660049 -0.153097618457159 0.367087088351024 0.569777261235492 0.273173157433557 0.484522025242166 0.436447811447811

hsa-miR-520g 59.7894736842105 58.0087719298245 1.03069711174958 0.0302353808344891 0.613811549751469 0.768823465073321 0.169770190153297 0.35584480228465 0.464225589225589

hsa-miR-28-3p 146.741228070175 105.105263157895 1.39613587047237 0.333708328022683 0.0116291862817269 0.0687396421995224 0.00547065447828587 0.0559838577610877 0.677188552188552

hsa-miR-296-3p 89.7543859649123 111.710526315789 0.803455045151158 -0.218834044154879 0.0846882271064124 0.231000015423936 0.0563560363887389 0.191477399226306 0.378787878787879

hsa-miR-1224-3p 144.600877192982 160.973684210526 0.898288921584654 -0.107263523468405 0.536147367114153 0.718470773011667 0.62010701444167 0.76951863118166 0.456228956228956

hsa-miR-590-3p 4.2280701754386 19.1754385964912 0.220494053064959 -1.51188455468588 0.0274364135138086 0.113834734915465 0.00513559664844819 0.0538419241478691 0.349747474747475

hsa-miR-455-3p 184.885964912281 136.434210526316 1.35512907062719 0.303896704870604 0.00493874923445114 0.0402088734842579 0.146137582823455 0.33084969527719 0.697390572390572

hsa-miR-485-5p 34.2105263157895 50.3859649122807 0.678969359331477 -0.387179278610322 0.448824797122879 0.638580685569542 0.500520172690908 0.677035907574065 0.446548821548822

hsa-let-7a 1091.65789473684 1031.42763157895 1.05839504519158 0.0567536523937978 0.588724058359397 0.760582129287664 0.152221980697303 0.33257612491588 0.538299663299663

hsa-miR-1253 80 81.3771929824561 0.98307642556861 -0.0170684145848948 0.9856162747341 0.996003331493593 0.872226027451855 0.94139779250849 0.498316498316498

hsa-miR-490-3p 98.1491228070175 91.3552631578947 1.07436746843343 0.0717320869462832 0.460043350978183 0.645556767307597 0.537952947488576 0.707783854806441 0.552188552188552

hsa-miR-194S 46.8771929824562 98.1260964912281 0.477724016940251 -0.73872208365105 0.000173838145193446 0.0078027816629123 0.000142724331047987 0.0123171097694413 0.236531986531987

hsa-miR-1259 23.4385964912281 33.3421052631579 0.70297290186793 -0.352436934332712 0.495002888187272 0.681319764761747 0.986714490131628 0.99478341703691 0.452441077441077

hsa-miR-1249 82.7719298245614 150.210526315789 0.551039476757767 -0.59594882673727 0.00458105886353309 0.0386816881202372 0.00615290817811004 0.0559838577610877 0.300925925925926

hsa-miR-651 1 25.6140350877193 0.0390410958904109 -3.24314044686783 0.00311240782998835 0.0313381813178616 0.00390290816355618 0.0480398021371946 0.306397306397306

hsa-miR-182 3612.86403508772 4503.74342105263 0.80219135446293 -0.220408102988067 0.0219489497594764 0.100118935102438 0.00842076769033694 0.0660883942967129 0.339225589225589

hsa-miR-1324 141.5 128.621710526316 1.10012531648807 0.0954240973955317 0.417434155698962 0.612675202420622 0.445388565696829 0.630115298682562 0.557239057239057

hsa-miR-15bS 78.7368421052631 83.1425438596491 0.947010260332867 -0.0544453512899507 0.791404164791597 0.884849671579158 0.892014572483303 0.948026697264837 0.518939393939394

hsa-miR-543 47.4736842105263 42.859649122807 1.1076545231273 0.102244737472993 0.828368841349171 0.90755164106274 0.117467864430858 0.290472111758826 0.484427609427609

hsa-miR-1287 64.7719298245614 63.4736842105263 1.02045328911001 0.0202469296675021 0.97124694544355 0.986106375266926 0.814835720841303 0.908531301144761 0.497053872053872

hsa-miR-220c 135.688596491228 85.2478070175439 1.59169603580892 0.464800136911471 0.000578190676489103 0.0118804417573832 0.000285797432062455 0.0169441422875013 0.741582491582492

hsa-miR-611 106.385964912281 105.690789473684 1.00657744579313 0.00655590878397494 0.524295460752419 0.708086044803345 0.43066334420148 0.61840676546735 0.45496632996633

hsa-miR-1825 39.9824561403509 62.3508771929824 0.641249296567248 -0.444336979468912 0.0396613073700743 0.148171897231057 0.0966499217462242 0.255073035067252 0.355639730639731

hsa-miR-320b 2106.82894736842 1198.42543859649 1.75799751867399 0.564175387813746 0.00133424736987941 0.0206069475498116 0.012734218405249 0.0814621023931436 0.72516835016835

hsa-miR-612 77.9298245614035 69.9298245614035 1.11440040140492 0.108316503681633 0.687370475966103 0.818207890701719 0.618057458585988 0.76920821087709 0.528619528619529

hsa-miR-671-5p 69.9649122807017 66.1578947368421 1.05754441792628 0.0559496338233717 0.547775153848326 0.726159689356536 0.998681697860201 0.998681697860201 0.542508417508417

hsa-miR-145S 86.8421052631579 29.2280701754386 2.97118847539016 1.08896203283595 0.000669701100603386 0.013440745344668 0.00105216766828778 0.0288321188652814 0.737794612794613

hsa-miR-657 61.859649122807 64.7127192982456 0.955911755735538 -0.0450896759071346 0.838104304193943 0.913324815248521 0.75029019270436 0.872860355212562 0.51473063973064

hsa-miR-1826 217.482456140351 119.044956140351 1.82689349630189 0.602616981390117 1.33303652104234e-05 0.0019173508627659 0.00332832544113708 0.0444867358954597 0.805555555555556

hsa-miR-99a 114.701754385965 84.8947368421053 1.35110560033065 0.300923220490837 0.374025951730785 0.571299816537464 0.614511632234647 0.767619948572308 0.562710437710438

hsa-miR-604 90.6754385964912 53.3421052631579 1.69988488735405 0.530560535448375 0.0270045810054149 0.113130841784821 0.194626765981513 0.390611393121036 0.65530303030303

hsa-miR-101S 102.5 110.666666666667 0.926204819277108 -0.076659881669916 0.540097568011849 0.720408348059081 0.669257903304043 0.813069097824269 0.456649831649832

hsa-miR-504 22.015350877193 39.9473684210526 0.55110891523935 -0.595822821052049 0.0228430664259502 0.101616321265954 0.00473628855588321 0.052402782355477 0.340909090909091

hsa-miR-424 230.30701754386 284.157894736842 0.810489596838921 -0.210116773374382 0.130116137969646 0.303487100183256 0.107862608447841 0.273780679677902 0.393518518518518

hsa-miR-767-5p 199.447368421053 119.698464912281 1.66624833966931 0.510574596062757 0.00300476529867848 0.0313381813178616 0.000810097923745646 0.0268890195458651 0.708333333333333

hsa-miR-222S 72.7061403508771 47.1754385964912 1.5411863146151 0.43255245403611 0.0152386177271479 0.0811785623365964 0.421495461195957 0.612422561950782 0.670454545454545

hsa-miR-875-5p 33.7543859649123 59.1666666666666 0.570496664195701 -0.561247956856259 0.0853601243257858 0.2312201274557 0.0254948075235329 0.117499875359226 0.379208754208754

hsa-miR-876-3p 9.71929824561404 38.5438596491228 0.252162039144288 -1.37768338563733 0.000446503002552997 0.010262112993706 0.000248764851630875 0.0165141589967265 0.257575757575758

hsa-miR-361-3p 154.285087719298 167.842105263158 0.919227553046931 -0.0842215778675345 0.54408096037613 0.723485159945455 0.617306690103177 0.76920821087709 0.457070707070707

hsa-miR-371-5p 30.3333333333333 39.280701754386 0.772219740955784 -0.258486130915464 0.608181448396525 0.765862641390888 0.599305693236253 0.758129797335532 0.536195286195286

hsa-miR-29b-2S 54.4385964912281 62.8782894736842 0.865777312756126 -0.144127548110461 0.647877469693606 0.795493859653857 0.84851294591648 0.929846130144737 0.532407407407407

hsa-miR-556-3p 1 18.9035087719298 0.0529002320185615 -2.93934755414335 2.44858013704647e-05 0.0021131246582711 0.000738886230631333 0.0255063526813936 0.220538720538721

hsa-miR-1263 80 87.4122807017544 0.915203211239337 -0.0886091495785184 0.434891822375707 0.624478606838993 0.504261702320947 0.681029497813736 0.44486531986532

hsa-miR-1224-5p 19.2280701754386 17.6491228070176 1.08946322067594 0.0856851168482761 0.394604871260677 0.595356650171267 0.0335610469482991 0.135341979048515 0.440656565656566

hsa-miR-346 70.1403508771929 82.9298245614035 0.845779564205627 -0.167496515732891 0.0973363071117197 0.247799031998263 0.0460408475028672 0.165555214145727 0.383417508417508

hsa-miR-1234 555.543859649123 577.889802631579 0.961331826793452 -0.0394356363824342 0.745653382745706 0.859159525731844 0.657643462203528 0.8075811491464 0.476851851851852

hsa-miR-154S 48.6315789473684 50.6414473684211 0.960311789542059 -0.0404972664771354 0.776993157208065 0.874243930470092 0.29108021849408 0.49743015556513 0.47979797979798

hsa-let-7b 965.995614035088 412.790570175439 2.34015911173682 0.850218923526679 0.000403212873619334 0.010262112993706 0.000673673149028285 0.0252773881570178 0.748316498316498

hsa-miR-26a 6386.57456140351 6690.78070175438 0.95453353593384 -0.0465325018773946 0.178662797508992 0.361091321429182 0.10610307669976 0.270204732241416 0.405723905723906

hsa-miR-631 155.53399122807 140.959429824561 1.10339543386099 0.0983921836031077 0.449152347787615 0.638580685569542 0.940887174297498 0.969367995268598 0.553451178451178

hsa-miR-379 1 4.24561403508773 0.235537190082644 -1.44588645832214 0.185931815665926 0.369461826681007 0.0524317908407694 0.181721427693108 0.414983164983165

hsa-miR-129-5p 39.2631578947368 60.8344298245614 0.645410140408428 -0.437869287668014 0.226823612846188 0.420965113733893 0.148594403886032 0.331361681017172 0.414983164983165

hsa-miR-374bS 18 45.4035087719299 0.39644513137558 -0.925217629890076 0.00143269623753035 0.0206069475498116 0.0117656099511074 0.0792912367213575 0.276936026936027

hsa-miR-615-3p 19.4035087719298 25.3508771929825 0.765397923875432 -0.267359418464253 0.22154489263982 0.412944367922601 0.184141548333391 0.374797538235181 0.41456228956229

hsa-miR-200a 118.526315789474 71.0087719298245 1.66917850525016 0.512331592375901 0.000996492811083552 0.0182973041694703 0.0641202824427076 0.203440454956091 0.731060606060606

hsa-let-7aS 1 18.9002192982456 0.0529094389975054 -2.93917352508006 0.176813134030312 0.359034669807433 0.155187576142913 0.334817195528335 0.410774410774411

hsa-miR-635 133.280701754386 96.7642543859649 1.37737538102415 0.320179790456421 0.0892543458946853 0.236277608917526 0.0806929515853758 0.226215169674796 0.619528619528619

hsa-miR-1251 164.131578947368 148.129385964912 1.10802848387049 0.102582295459003 0.875900442309517 0.934367184284178 0.262518719944077 0.471005520398624 0.511363636363636

hsa-miR-1225-5p 41.0877192982456 74.7807017543859 0.549442815249267 -0.598850577434109 0.0833215086713024 0.228274482486774 0.0364490268654742 0.143185354970995 0.378367003367003

hsa-miR-19b 8542.95614035088 9651.24561403509 0.885166172532952 -0.121979886026 0.13456388725125 0.308739345616617 0.295964359293288 0.500818121706094 0.39520202020202

hsa-miR-1184 195.69298245614 156.059210526316 1.2539662465045 0.226311525185394 0.0360610298805624 0.138931557084488 0.15405313194688 0.334039831332054 0.647306397306397

hsa-miR-30c-1S 44.9122807017544 8.42105263157896 5.33333333333333 1.67397643357167 0.051004459973149 0.17060794169313 0.151468321588056 0.33257612491588 0.634259259259259

hsa-miR-216b 190.197368421053 148.765350877193 1.27850583015169 0.245692075888638 0.417442663989949 0.612675202420622 0.867266255318135 0.939085041831305 0.557239057239057

hsa-miR-182S 53.4035087719298 29.8859649122807 1.78690930437335 0.580487481880555 0.012583994729631 0.0728858218232989 0.0413816778939175 0.15760925839842 0.675084175084175

hsa-miR-32S 29.280701754386 65.469298245614 0.447243250485697 -0.804652647851513 0.0935911191064775 0.241579234944707 0.0419492452495833 0.15760925839842 0.382575757575758

hsa-miR-376b 77.9298245614035 52.3859649122807 1.48760884125921 0.397170026355237 0.0545537348540003 0.175817382454783 0.0837454427518421 0.231140615020453 0.634680134680135

hsa-let-7bS 1 17.8245614035088 0.0561023622047244 -2.88057736030388 0.0128506327058791 0.0732038737785599 0.0110127879671999 0.0748948766909616 0.333333333333333

hsa-miR-483-3p 11.8947368421053 25.7280701754386 0.462325264234572 -0.771486600477111 0.604386851831538 0.765862641390888 0.315125914521772 0.519954538710003 0.464646464646465

hsa-miR-574-3p 1292.87719298246 1907.68201754386 0.677721539068149 -0.389018784744936 0.304043335392254 0.50319517262114 0.362998618008461 0.562352875240527 0.427609427609428

hsa-miR-541S 1 1 1 0 0.369766088918265 0.570783139437775 0.080250423348772 0.226215169674796 0.443181818181818

hsa-miR-1254 135.688596491228 140.322368421053 0.966977667338679 -0.0335798785849021 0.544069924718526 0.723485159945455 0.194447713362195 0.390611393121036 0.457070707070707

hsa-miR-302bS 15.7894736842105 14.6228070175439 1.07978404319136 0.0767610611334614 0.735001527227677 0.850276565680275 0.855311875262464 0.932428556765725 0.476430976430976

hsa-miR-217 175.798245614035 113.400219298246 1.55024608155017 0.438413680620188 0.000200775752721673 0.0078027816629123 0.00998400726164168 0.0706245759573506 0.760942760942761

hsa-miR-1271 228.412280701754 228.264254385965 1.00064848662436 0.000648276447771584 0.923439062947324 0.960154110028362 0.872674662812042 0.94139779250849 0.507154882154882

hsa-miR-503 303.464912280702 240.30701754386 1.26282168278883 0.233348647960395 0.0706044311832008 0.203105413703674 0.206847586437915 0.406627487690024 0.627104377104377

hsa-miR-770-5p 49.0526315789474 67.6578947368421 0.725009723842863 -0.321570212020352 0.315680589013863 0.518918758702788 0.275452665111983 0.48687630342874 0.429292929292929

hsa-miR-320c 538.96052631579 275.491228070175 1.9563618416863 0.671086545141136 0.000421850492268874 0.010262112993706 0.00432109008310088 0.050357858827868 0.747474747474748

hsa-miR-621 387.254385964912 224.06798245614 1.72828969904575 0.547132306240926 0.0128941955431676 0.0732038737785599 0.345016268631939 0.549352472009895 0.6746632996633

hsa-miR-411 58.3333333333333 52.078947368421 1.12009432373252 0.113412899379224 0.0629319535706974 0.189234410911191 0.493825884610108 0.671136595934682 0.630471380471381

hsa-miR-432S 44.421052631579 63.8245614035087 0.695986805937329 -0.362424575814 0.0571926872514008 0.1788307576013 0.0313322375631025 0.130994294631827 0.366582491582492

hsa-miR-1258 1 1 1 0 0.0626212081342773 0.189140017480406 0.0571069819737817 0.192512990013178 0.396885521885522

hsa-miR-29a 594.078947368421 536.515350877193 1.10729161131571 0.101917043892371 0.410504252750006 0.607722250584458 0.726069220503945 0.850173895135105 0.558080808080808

hsa-miR-548a-5p 1 11.3070175438596 0.0884406516679597 -2.42542355454469 0.275881260973289 0.476171056439897 0.185656994414384 0.376992908657914 0.426767676767677

hsa-miR-135a 1 10.7543859649123 0.0929853181076672 -2.37531366810166 9.11501391220492e-06 0.0019173508627659 0.000137037943541967 0.0123171097694413 0.242424242424242

hsa-miR-196b 1 1 1 0 0.000311209196993977 0.00929037107299972 0.00065523251797083 0.0252773881570178 0.297138047138047

hsa-miR-769-5p 1 2.35855263157895 0.423988842398884 -0.858048139193398 0.187314913119029 0.369461826681007 0.251389383927192 0.456426715911051 0.416666666666667

hsa-miR-1243 75.1754385964912 52.6666666666667 1.427381745503 0.355841818838473 0.0983334187057229 0.248861408630613 0.46769920913672 0.649958804323655 0.616161616161616

hsa-miR-103-as 179.236842105263 164.530701754386 1.08938234745288 0.0856108819192098 0.932982590720593 0.966583404311971 0.704188464353848 0.832485814708727 0.506313131313131

hsa-miR-141 50.6754385964912 34.4473684210526 1.47109752992106 0.386008741203256 0.370285685496951 0.570783139437775 0.657855791251355 0.8075811491464 0.563131313131313

hsa-miR-193b 74.578947368421 100.048245614035 0.745429836482398 -0.293794265259658 0.0764124482753061 0.212722396327707 0.0956592309325825 0.253232872069996 0.375420875420875

hsa-miR-548c-3p 42.2456140350877 40.3157894736842 1.04786771105309 0.0467573480200107 0.713363841490913 0.833062239792501 0.931040149219143 0.964571006934118 0.526094276094276

hsa-miR-520c-3p 1 1 1 0 0.00322516919697864 0.031853882632714 0.00257439996729173 0.0400276533854518 0.337121212121212

hsa-miR-1180 51.280701754386 72.844298245614 0.703976879308788 -0.351009765254327 0.383648927183401 0.581061901951541 0.346377260812791 0.549491867796762 0.438552188552189

hsa-miR-526a 167.684210526316 81.5888157894737 2.05523525379994 0.720390320130774 0.00627806167670882 0.0471127584956497 0.0604019842160037 0.195965836009065 0.691919191919192

hsa-miR-383 67.4035087719298 57.4572368421052 1.17310738344688 0.15965611147178 0.913836655363723 0.954797468660277 0.491659810866878 0.669246714161066 0.507996632996633

hsa-miR-1284 15.7894736842105 42.0877192982456 0.375156315131305 -0.980412499515384 0.00054937093122724 0.0115635881377831 0.00673398582490983 0.0587013107767393 0.259259259259259

hsa-miR-197 449.135964912281 370.885964912281 1.21098129183321 0.191431015924137 0.0734602573864088 0.209091750474475 0.0451101154960493 0.164957752852078 0.625841750841751

hsa-miR-614 40.3333333333333 32.9298245614035 1.22482685135855 0.202799488176672 0.923185420796639 0.960154110028362 0.235214683464144 0.445154104889378 0.507154882154882

hsa-miR-518a-5p 180.635964912281 135.633771929825 1.33179194489806 0.286525362394113 0.00186269254197802 0.0223264397739865 0.144979251507828 0.329255510661198 0.718434343434343

hsa-miR-186S 103.59649122807 108.881578947368 0.951460221550856 -0.0497573991620202 0.773103005874362 0.871011557178635 0.144032295075091 0.328095023184096 0.479377104377104

hsa-miR-887 116.723684210526 125.497807017544 0.930085449176088 -0.0724788162211177 0.648009482429504 0.795493859653857 0.994692118715354 0.998161974943431 0.532407407407407

hsa-miR-508-5p 133.280701754386 113.361842105263 1.17571044435417 0.161872598371848 0.361255700543068 0.563781627556888 0.89200386581698 0.948026697264837 0.564393939393939

hsa-miR-499-3p 112.982456140351 129.570175438597 0.871978877530296 -0.136990078382255 0.209301046484696 0.394381666192779 0.169191518049378 0.35584480228465 0.411616161616162

hsa-miR-508-3p 1 11.9473684210526 0.0837004405286343 -2.48051103831496 0.000380069773830696 0.010262112993706 0.00156438994657082 0.034617141638221 0.272306397306397

hsa-miR-124 125.649122807018 114.618421052632 1.0962384724295 0.0918847491912672 0.241463586718227 0.438771514020744 0.294280252475423 0.498946675611572 0.582491582491583

hsa-miR-1290 1 1 1 0 0.0189928396133714 0.0923027567139672 0.00199382095636624 0.0358472392780013 0.363636363636364

hsa-miR-592 57.8245614035088 49.6666666666667 1.16425291416461 0.152079605917463 0.33151431066724 0.531433178084382 0.285607074613999 0.493945702188138 0.568181818181818

hsa-miR-1307 88.7368421052632 69.438596491228 1.27791814047499 0.2452323010674 0.136267431265598 0.308739345616617 0.427677738163472 0.616170096886604 0.60479797979798

hsa-miR-1911S 100.701754385965 103.241228070175 0.975402523471685 -0.0249050485948244 0.709594241739729 0.829782968321662 0.992216333208895 0.997998479672816 0.526515151515151

hsa-miR-489 249.100877192982 130.644736842105 1.9067042669621 0.645376236963818 0.00118085837909955 0.0206069475498116 0.00996152069410072 0.0706245759573506 0.727693602693603

hsa-miR-302e 1 7.49122807017545 0.133489461358314 -2.01373274539408 0.0215726881903507 0.0995573791886236 0.021726964162089 0.106536193590243 0.346801346801347

hsa-miR-1278 70.1403508771929 34.8508771929824 2.01258494840171 0.699419939805692 0.00163861844728541 0.0216814088946127 0.0034022300916574 0.0444867358954597 0.720959595959596

hsa-miR-1267 29.8245614035088 41.6666666666667 0.715789473684211 -0.334369186424434 0.331914812268229 0.531433178084382 0.243643592472272 0.455117792864871 0.431818181818182

hsa-miR-570 95.719298245614 80.7543859649123 1.18531392570063 0.170007655706056 0.295885478750403 0.494899632421219 0.870708086148446 0.94139779250849 0.573653198653199

hsa-miR-1247 56.9824561403509 74.5964912280701 0.763875823142051 -0.26935003818027 0.304366025617885 0.50319517262114 0.339081324316888 0.542907574926669 0.427609427609428

hsa-miR-432 1 1 1 0 0.0854683901023965 0.2312201274557 0.0490029621987548 0.173059631748476 0.393518518518518

hsa-miR-375 1 34.9912280701754 0.0285785911255954 -3.55509740351091 0.00638913939559114 0.0475329939516823 0.00578780985748524 0.0559838577610877 0.316498316498317

hsa-miR-758 47.0175438596491 49.5263157894737 0.949344668792065 -0.051983354748587 0.163130082886336 0.344906058526738 0.062134650538885 0.199339046152631 0.402356902356902

hsa-miR-518e 84.3552631578948 80.6929824561403 1.04538536797478 0.0443855906182652 0.754782712247714 0.865661138415829 0.532260503709411 0.703826631720299 0.522306397306397

hsa-miR-620 4.70175438596492 17.0438596491228 0.275862068965518 -1.28785428830664 0.319841022262178 0.522739739218778 0.144088080865321 0.328095023184096 0.432239057239057

hsa-miR-17S 708.070175438597 550.65076754386 1.2858788494873 0.251442414136044 0.0553347157145168 0.176866146894919 0.125606554378184 0.300272732488568 0.634680134680135

hsa-miR-100 116.5 113.285087719298 1.02837895388904 0.027983731292603 0.86172052559464 0.9226610590424 0.318372812454846 0.521640344864857 0.512626262626263

hsa-miR-495 97.7236842105263 91.9320175438596 1.06299945134897 0.0610945832251206 0.956889563687402 0.984261851564038 0.566026075318168 0.729075377611312 0.504208754208754

hsa-miR-196aS 171.657894736842 162.460526315789 1.05661294241516 0.0550684547882979 0.99520742843213 0.999841688867204 0.633204897076526 0.780651180252918 0.500841750841751

hsa-miR-877 75.1754385964912 112.881578947368 0.665967284454287 -0.406514732089305 0.0891393864381241 0.236277608917526 0.0982264487972694 0.256877046400132 0.38047138047138

hsa-miR-509-3p 1 1 1 0 0.443836991085093 0.634157820043767 0.455660109793966 0.638367978493819 0.452861952861953

hsa-miR-875-3p 46.5263157894737 38.8070175438596 1.19891500904159 0.181416988663528 0.453016306631569 0.639857729333951 0.280027475710141 0.489675766599593 0.552609427609428

hsa-miR-1269 46.3684210526316 40.5 1.14489928525016 0.135316672657246 0.592764125570796 0.763746770799555 0.949487631665766 0.973889783397329 0.537878787878788

hsa-miR-500S 259.482456140351 203.816885964912 1.27311559546161 0.241467120999917 0.00272397805138418 0.0297568741562601 0.0138111936588946 0.0839048283005464 0.71043771043771

hsa-miR-144S 716.530701754386 574.457236842105 1.24731773890305 0.220995436890012 0.0238943039303746 0.104145375211683 0.0297665166195642 0.127803501704895 0.658670033670034

hsa-miR-181c 161.657894736842 109.706140350877 1.47355375204893 0.387677001694793 0.00364729328114784 0.033845312920759 0.00103091915909598 0.0288321188652814 0.704124579124579

hsa-miR-378S 40.2807017543859 42.780701754386 0.941562435923723 -0.0602146177106506 0.609401427592644 0.765862641390888 0.172852359468594 0.356869823496163 0.463804713804714

hsa-miR-1470 184.570175438596 215.59649122807 0.856090812922125 -0.15537881860523 0.13165437184338 0.304613314800016 0.0807349620623839 0.226215169674796 0.393939393939394

hsa-miR-449a 1 1 1 0 0.326065366223965 0.52695582593873 0.202240712428129 0.399390697541133 0.441077441077441

hsa-miR-1255b 86.6666666666667 78.5964912280701 1.10267857142857 0.0977422847729375 0.732075759996133 0.848028699163306 0.444945565335581 0.630115298682562 0.524410774410774

hsa-miR-450a 1 35.7105263157895 0.028002947678703 -3.57544550010848 0.00348737535304158 0.0331210362881238 0.0043764071982504 0.050357858827868 0.306397306397306

hsa-miR-324-3p 587.213815789474 436.161184210526 1.34632295822555 0.297377141688039 0.003061037060792 0.0313381813178616 0.0029322851780531 0.0428908831976241 0.707912457912458

hsa-miR-208b 88.1578947368421 71.0701754385965 1.24043446062701 0.215461689724586 0.202878004030987 0.385646954798991 0.252806454467534 0.456426715911051 0.589646464646465

hsa-miR-93S 760.899122807018 1014.75438596491 0.749835756643212 -0.287901087576227 0.364375221377458 0.566587055943687 0.620608305101316 0.76951863118166 0.436026936026936

hsa-miR-542-5p 112.359649122807 98.1271929824561 1.14504089751039 0.135440354718005 0.159841843610226 0.343208584918843 0.792439341179753 0.893747495361328 0.598905723905724

hsa-miR-144 2637.20614035088 2295.07456140351 1.14907209756974 0.138954745000509 0.904324974105092 0.950588555578699 0.784296296174281 0.889652403868437 0.508838383838384

hsa-miR-593S 315.552631578947 265.18201754386 1.18994732184944 0.173909038790199 0.861709270796961 0.9226610590424 0.282041171516742 0.490728893183364 0.512626262626263

hsa-miR-520c-5p 134.754385964912 128.03125 1.05251167949163 0.0511793834169872 0.192432870845135 0.372353290446977 0.327760378364137 0.532393151924321 0.591750841750842

hsa-miR-143S 133.280701754386 144.847587719298 0.920144434939933 -0.0832246267614706 0.601275196073496 0.765339961963757 0.373328393814594 0.569226861946987 0.462962962962963

hsa-miR-212 55.8947368421053 35.1666666666667 1.58942379645797 0.463371558387432 0.0380992058564479 0.144844117419007 0.799702773906319 0.898624340991085 0.645622895622896

hsa-miR-16-1S 95.719298245614 99.1600877192982 0.965300661255722 -0.0353156601016736 0.552076570702733 0.729639536157512 0.519358537897056 0.692745623191901 0.457912457912458

hsa-miR-338-5p 35.6820175438596 52.8947368421053 0.674585406301824 -0.393656989701803 0.18974282193364 0.370459095864108 0.038180849760177 0.147098541709968 0.407828282828283

hsa-miR-885-5p 15.6315789473684 52.6842105263158 0.296703296703297 -1.21502264051252 0.000285223802081332 0.00911659782208109 0.00349740283592243 0.0447643268752735 0.246632996632997

hsa-miR-127-3p 68.2105263157894 32.8070175438597 2.07914438502674 0.731956455731697 0.008689070600458 0.0576820609861173 0.773799348262216 0.885661588262988 0.683922558922559

hsa-miR-1303 29.280701754386 41.2982456140351 0.709005947323705 -0.343891364158387 0.299074893924716 0.497305652133005 0.0584963565722876 0.193418987440169 0.427188552188552

hsa-miR-548m 1 1.51754385964912 0.658959537572255 -0.417093146103283 0.149859722609533 0.330483635323399 0.574011073819696 0.734972636062904 0.410774410774411

hsa-miR-650 140.640350877193 159.701754385965 0.880643743820719 -0.127102111876397 0.0442081271898629 0.158965057353549 0.0143385629387145 0.0850392241152702 0.358585858585859

hsa-miR-33aS 89.7543859649123 81.0526315789474 1.10735930735931 0.101978178622949 0.290457158570373 0.488624810616437 0.667350503629111 0.813069097824269 0.57449494949495

hsa-miR-575 140.377192982456 149.982456140351 0.935957421920693 -0.066185292940188 0.796190407293548 0.888890454714531 0.480687378688962 0.661101796159223 0.481481481481482

hsa-miR-607 110.530701754386 81.4407894736842 1.35719094164849 0.305417079611891 0.0157419811942409 0.0824001758628353 0.0124960838422744 0.0810836116983671 0.669612794612795

hsa-miR-548j 1 1 1 0 0.0129782070545998 0.0732038737785599 0.00119489797571354 0.0297568803291275 0.349747474747475

hsa-miR-509-3-5p 283.236842105263 186.719298245614 1.5169125246641 0.416677035331419 0.0176644802886024 0.0876117614314017 0.32881292001815 0.532393151924321 0.666666666666667

hsa-miR-1204 1 14.9649122807018 0.0668229777256741 -2.70570827965713 0.0276667700465846 0.114241256221065 0.0435511979954819 0.161307656094853 0.35479797979798

hsa-miR-1237 91.4035087719298 122.756578947368 0.744591528663558 -0.29491949463811 0.556106832524657 0.73270259002867 0.363604561531117 0.562352875240527 0.458333333333333

hsa-miR-27aS 43.6140350877193 59.359649122807 0.734742130929511 -0.308235683601623 0.0537755120156367 0.175368041075989 0.0297337591485037 0.127803501704895 0.364478114478115

hsa-miR-363S 67.1929824561403 41.1929824561403 1.63117546848382 0.489300901226704 0.056717890108993 0.178559552788495 0.46233116604211 0.644318410290979 0.633838383838384

hsa-miR-1294 1 11.8157894736842 0.0846325167037862 -2.46943672801587 0.276474600456974 0.47624267503866 0.395485709047999 0.591515020638515 0.427609427609428

hsa-miR-935 32.7368421052632 34.359649122807 0.952769977023232 -0.0483817716985965 0.708442787864746 0.829782968321662 0.496829108186008 0.674156478560574 0.473484848484849

hsa-miR-1202 231.236842105263 237.40350877193 0.974024534436891 -0.0263187862965914 0.348727408932152 0.550186021770469 0.0902116994045493 0.244052340395379 0.433922558922559

hsa-miR-876-5p 35.6820175438596 28 1.27435776942356 0.242442341449728 0.763475079388154 0.867465493768325 0.579175491286682 0.740486591082083 0.521464646464647

hsa-miR-588 139.412280701754 109.495614035088 1.27322251151612 0.241551097322055 0.0488264635386994 0.166241897837262 0.404957535385595 0.599448289944715 0.638468013468014

hsa-miR-1181 185.69298245614 195.736842105263 0.948686923007977 -0.0526764367955449 0.665402915875475 0.805389504068071 0.221642134489698 0.429836319246312 0.469276094276094

hsa-miR-412 53.2982456140351 55.6491228070175 0.957755359394704 -0.0431628995990199 0.705132987562491 0.829782968321662 0.629783026199686 0.778657237264081 0.473063973063973

hsa-miR-22S 52.4385964912281 88.3728070175439 0.593379324035932 -0.521921414947938 0.0249553568621635 0.107086549208665 0.0160336258457969 0.0898507734085891 0.342592592592593

hsa-miR-381 107.157894736842 93.7543859649123 1.14296407185629 0.133624951122042 0.287724983961256 0.48497394757532 0.424520498407171 0.614130662014604 0.574915824915825

hsa-miR-18a 1470.35964912281 828.118421052631 1.7755427385057 0.574106144362045 0.00457177943293524 0.0386816881202372 0.0106191680149106 0.0745068455029908 0.699074074074074

hsa-miR-634 144.600877192982 153.979714912281 0.939090433277908 -0.0628434963410089 0.800834617811826 0.891768096995621 0.614629645959982 0.767619948572308 0.518097643097643

hsa-miR-152 213.377192982456 199.894736842105 1.06744777953309 0.0652705464539197 0.41742990141692 0.612675202420622 0.677205609966545 0.815179424209318 0.557239057239057

hsa-miR-92bS 125.40350877193 155.035087719298 0.808871789068689 -0.212114855238685 0.441991219671784 0.632567864969734 0.364404553285214 0.562352875240527 0.445707070707071

hsa-miR-891b 135.087719298246 77.3728070175439 1.74593277025112 0.55728895166526 0.00409056089283134 0.035658121722358 0.150690159062765 0.33257612491588 0.701599326599327

hsa-miR-501-3p 304.815789473684 305.342105263158 0.998276307851418 -0.0017251794151996 0.861709270796961 0.9226610590424 0.598398319820482 0.758129797335532 0.487373737373737

hsa-miR-1295 100.175438596491 100.482456140351 0.996944565691838 -0.00306011167759477 0.852263482012211 0.9226610590424 0.546753633312549 0.716006654853914 0.486531986531987

hsa-miR-363 3612.86403508772 3681.29166666667 0.9814120591969 -0.0187628676527961 0.448463411541926 0.638580685569542 0.690683617141877 0.823287239769945 0.446548821548822

hsa-miR-1236 24.8421052631579 43.3245614035088 0.573395424174934 -0.556179705727014 0.334684294434492 0.53388640683358 0.367117912476752 0.564746450031083 0.433080808080808

hsa-miR-103 8542.95614035088 8274.35964912281 1.0324613024594 0.0319455656924749 0.695702206085743 0.823028572191168 0.677950615070899 0.815179424209318 0.472222222222222

hsa-miR-643 69.8947368421052 64.8793859649122 1.07730268717255 0.0744604052735302 0.639362572570976 0.791635437774393 0.251067496727179 0.456426715911051 0.466750841750842

hsa-miR-609 1 1 1 0 0.243702889673025 0.440336253489057 0.283001699708248 0.491409390036656 0.43013468013468

hsa-miR-1264 18 29.7105263157895 0.605845881310895 -0.501129646527111 0.471431817259517 0.65726277592078 0.0421874144728099 0.15760925839842 0.44949494949495

hsa-miR-138 39.0350877192982 51.9649122807017 0.75118163403106 -0.286107800227404 0.251046001425334 0.449486927863201 0.238815144960976 0.449014096081312 0.419191919191919

hsa-miR-877S 106.631578947368 87.0877192982456 1.22441579371475 0.20246382715224 0.961668698425894 0.984490894517879 0.834461258490423 0.922656211440404 0.503787878787879

hsa-miR-646 287.973684210526 211.429824561403 1.36202962286852 0.308975957027163 0.114168651924373 0.276762771378465 0.388854977372366 0.585657670981417 0.611111111111111

hsa-miR-585 10 2.35855263157895 4.23988842398885 1.44453695380065 0.893679103932869 0.94399640966226 0.786564060430611 0.889652403868437 0.490740740740741

hsa-miR-106aS 158.956140350877 136.495614035088 1.16455126763279 0.152335834819958 0.045452055766942 0.160966696034345 0.122937432348034 0.295529259377029 0.640572390572391

hsa-miR-27bS 70.1403508771929 29.8070175438597 2.35314891112419 0.855754393388413 0.00128959179859323 0.0206069475498116 0.00223824198254753 0.0371462082872793 0.725589225589226

hsa-miR-106bS 192.921052631579 179.155701754386 1.076834567599 0.0740257815466626 0.321639399218043 0.522739739218778 0.401187767017111 0.595912294209581 0.56986531986532

hsa-miR-220b 27.140350877193 30.0175438596491 0.904149620105202 -0.100760423319127 0.64075856659947 0.792227282199632 0.636407413911574 0.783480168624377 0.467171717171717

hsa-miR-569 6.87719298245615 23.2982456140351 0.295180722891567 -1.22016749024592 0.153837009849645 0.333008656740415 0.3544782339953 0.554965958025664 0.402356902356902

hsa-miR-302d 1 16.4561403508772 0.0607675906183369 -2.80069868117167 1.28281056524895e-05 0.0019173508627659 0.000131181741420818 0.0123171097694413 0.220538720538721

hsa-let-7g 338.219298245614 500.404605263158 0.675891657847049 -0.391722485240723 0.786909704844994 0.881952045819779 0.628558115273222 0.7782577524832 0.480639730639731

hsa-miR-559 59.3333333333333 51.9649122807017 1.14179608372721 0.132602534630781 0.852215418501061 0.9226610590424 0.406799837714359 0.601144280732007 0.513468013468013

hsa-miR-206 1 9.37719298245615 0.106641721234799 -2.23828046263055 0.0357479114123528 0.13834281412045 0.10483766938357 0.26927056154173 0.362373737373737

hsa-miR-30eS 53.2982456140351 50.9824561403509 1.0454232622161 0.0444218390254897 0.828679829846395 0.90755164106274 0.164407676772176 0.34925017025842 0.484427609427609

hsa-miR-193a-5p 78.3508771929824 75.5021929824561 1.03772982079061 0.0370354626071574 0.763815274652823 0.867465493768325 0.963708178484273 0.982365673525622 0.521464646464647

hsa-miR-602 140.868421052632 131.90350877193 1.06796568464454 0.0657556095391705 0.918659015275148 0.957495851331192 0.718529713789207 0.842515139945768 0.507575757575758

hsa-miR-487a 75.7214912280701 74.5964912280701 1.01508113828786 0.0149685484986497 0.773111069291813 0.871011557178635 0.96970503182968 0.984535814669428 0.520622895622896

hsa-miR-92b 248.256578947368 210.377192982456 1.18005462202393 0.165560727257023 0.168982233088542 0.350556892200509 0.172733790258773 0.356869823496163 0.596801346801347

hsa-miR-142-5p 651.495614035088 729.178728070176 0.893464920129141 -0.112648206340702 0.163383751755959 0.344906058526738 0.127785207776993 0.302963281075674 0.401936026936027

hsa-miR-191 9071.26754385965 11503.9078947368 0.78853791484282 -0.237574788962955 0.0139940288901426 0.0774156854627761 0.013546369052922 0.0836419451793613 0.328282828282828

hsa-miR-1207-3p 32.7017543859649 23.4035087719298 1.39730134932534 0.334542768769967 0.567257320968195 0.742822282650008 0.905630568766673 0.949646635292392 0.54040404040404

hsa-miR-125b-1S 1 1 1 0 0.890919716781659 0.942234945566877 0.764512734294741 0.878527948996487 0.490740740740741

hsa-miR-188-5p 134.421052631579 97.4736842105263 1.37904967602592 0.32139462138636 0.00186197548942663 0.0223264397739865 0.00134442482484504 0.0322288506622575 0.718434343434343

hsa-miR-1260 1907.68201754386 2580.36403508772 0.739307319278696 -0.302041586185802 0.0936264333280051 0.241579234944707 0.104175811611075 0.269172830599874 0.382154882154882

hsa-miR-581 7.61403508771931 23.3070175438597 0.326684230334965 -1.11876123109946 0.0238400216936672 0.104145375211683 0.00259614532088213 0.0400276533854518 0.347222222222222

hsa-miR-130aS 96.1403508771929 104.410087719298 0.920795614458843 -0.0825171843368457 0.46369514922956 0.649624860040764 0.188159070448627 0.38117670844405 0.448232323232323

hsa-miR-132S 1 1 1 0 6.2694643944465e-05 0.00416195982492871 0.00189422636290722 0.034907679426664 0.287457912457912

hsa-miR-339-5p 599.291666666667 534.070175438597 1.12212157545496 0.115221157250881 0.597074113066331 0.764515778737737 0.605282414928753 0.76044952567656 0.537457912457912

hsa-miR-129S 51.9649122807017 52.6491228070175 0.98700433188937 -0.0130808506126234 0.691348412654056 0.819551758407212 0.42483894000315 0.614130662014604 0.471801346801347

hsa-miR-320d 559.473684210526 284.921052631579 1.96360949478156 0.674784358585041 0.000140037807344916 0.00746434700102983 0.000209576935592383 0.0150720746180189 0.767255892255892

hsa-miR-1322 127.828947368421 94.0723684210526 1.3588362822575 0.306628658638639 0.410547012851378 0.607722250584458 0.604670483367874 0.76044952567656 0.558080808080808

hsa-miR-548l 1 1 1 0 0.0739744198330951 0.209091750474475 0.0710805165402461 0.215298408879636 0.404882154882155

hsa-miR-886-5p 86.8421052631579 66.7105263157894 1.30177514792899 0.263728831429289 0.213709422050596 0.401587242232451 0.147923670618626 0.33084969527719 0.587542087542087

hsa-miR-98 14.1754385964912 51.8771929824562 0.273249915454853 -1.29736846133138 0.150342320736711 0.330483635323399 0.308456900030665 0.514890337962213 0.400673400673401

hsa-miR-15a 2637.20614035088 3681.29166666667 0.716380656341423 -0.33354361035858 0.00800328359289634 0.0552546699253564 0.0593819786166504 0.194854173179351 0.313973063973064

hsa-miR-597 85.6052631578948 72.423245614035 1.18201362604088 0.167219446870827 0.420815869174863 0.616577411032099 0.941286651257341 0.969367995268598 0.556818181818182

hsa-miR-26a-2S 1 1 1 0 0.00683865693353844 0.0491813411136973 0.0122137061186076 0.0804612853462473 0.359427609427609

hsa-miR-184 4.70175438596492 23.1052631578948 0.203492786636295 -1.59212472123242 0.0235489463389682 0.103833974772865 0.00839799925369294 0.0660883942967129 0.344276094276094

hsa-miR-186 23.8070175438597 35.8245614035088 0.664544564152792 -0.408653338889741 0.515290981868403 0.702521512405105 0.297105924661462 0.501765974526109 0.454545454545455

hsa-miR-518d-5p 179.622807017544 113.631578947368 1.58074725953373 0.457897684305697 0.0013678035434666 0.0206069475498116 0.0010569656235991 0.0288321188652814 0.724747474747475

hsa-miR-28-5p 315.552631578947 405.839912280702 0.77752981417139 -0.2516332894628 0.0376699362714975 0.143845818594258 0.0328447392433356 0.134925243804191 0.353956228956229

hsa-miR-15b 12702.7105263158 14207.2368421053 0.894101412363973 -0.111936073593334 0.578197961216477 0.751483193569005 0.937019749430772 0.96844077096857 0.460858585858586

hsa-miR-874 171.973684210526 182.438596491228 0.942638715261083 -0.059072192493746 0.678541687236602 0.813307605673871 0.55904716133344 0.723486268120741 0.470538720538721

hsa-miR-130a 1292.87719298246 1135.39035087719 1.13870722257203 0.129893603636854 0.241222701140911 0.438771514020744 0.120281712958737 0.29390214753219 0.582491582491583

hsa-miR-421 112.982456140351 143.90350877193 0.785126485827492 -0.241910445736857 0.723048008059751 0.842092349467699 0.434156640439319 0.620196236099349 0.474747474747475

hsa-miR-18bS 84.9912280701755 82.2478070175439 1.03335555259299 0.0328113251011583 0.852288829967587 0.9226610590424 0.523747957511113 0.695376134357063 0.486531986531987

hsa-miR-34bS 46.5964912280702 67.6578947368421 0.688707377155452 -0.372938804777729 0.062408159349008 0.189140017480406 0.0366485650657755 0.143185354970995 0.369107744107744

hsa-miR-219-2-3p 1 11.2456140350877 0.0889235569422777 -2.41997818908612 0.0335408293705131 0.13051969550259 0.0185830724981592 0.0983876783184749 0.363636363636364

hsa-miR-1250 33.7543859649123 86.0526315789474 0.392252803261978 -0.935848740773812 0.00848568493890468 0.0568409848087073 0.013903117551539 0.0839048283005464 0.31523569023569

hsa-miR-194 5199.04385964912 4726.70175438596 1.09993059215654 0.095247079774053 0.171949525977938 0.353426369498298 0.351329460552251 0.552271993545707 0.595959595959596

hsa-miR-583 53.4035087719298 73.6052631578947 0.725539268263616 -0.320840082328547 0.373794518011126 0.571299816537464 0.06639655490056 0.208364461378848 0.437289562289562

hsa-miR-568 93.5087719298245 44.280701754386 2.11172741679873 0.747506293498786 0.00520845502686047 0.0412375842952347 0.0656945524201807 0.206971666628807 0.696127946127946

hsa-miR-633 96.1403508771929 106.353070175439 0.903973441656185 -0.100955297720434 0.966462904399976 0.984719894587695 0.772850787058707 0.885661588262988 0.496632996632997

hsa-miR-409-3p 36.280701754386 71.438596491228 0.507858546168959 -0.677552322602038 0.0533706236328554 0.175368041075989 0.0721489163098719 0.216196231859095 0.364478114478114

hsa-miR-26bS 1 6.7280701754386 0.148631029986962 -1.90628835297276 0.140216406544571 0.315944540073016 0.0137516089401814 0.0839048283005464 0.403198653198653

hsa-miR-924 19.4035087719298 28.0263157894737 0.692331768388107 -0.36769000416941 0.0393002327827554 0.147914449595085 0.0150178904383542 0.0869828150892598 0.356060606060606

hsa-miR-610 40.5263157894737 32.359649122807 1.25237191650854 0.225039286479109 0.285722545225682 0.483487365744635 0.435502803101049 0.620196236099349 0.574915824915825

hsa-miR-448 110.530701754386 118 0.936700862325305 -0.0653912981682397 0.937759452936992 0.970367395545113 0.890918348255952 0.948026697264837 0.494107744107744

hsa-miR-517a 31.8771929824561 33.280701754386 0.957828149710068 -0.0430869015388434 0.483721685907418 0.668993293170035 0.311414286451779 0.516902930395369 0.450757575757576

hsa-miR-125a-3p 18.6315789473684 11.219298245614 1.66067240031274 0.507222580782987 1 1 0.114407980650727 0.285358633819589 0.5

hsa-miR-100S 77.6491228070175 47.5087719298245 1.63441654357459 0.491285886578829 0.0138833735413141 0.0772990410719618 0.0236901515944238 0.112953595723689 0.672558922558923

hsa-miR-199b-3p 124.986842105263 107.967105263158 1.15763816952044 0.1463818687705 0.42436712834778 0.616596713512132 0.69607759241676 0.826292932951395 0.556397306397306

hsa-miR-10aS 42.4561403508772 48.5263157894737 0.874909616775126 -0.133634693073972 0.191957200731072 0.372353290446977 0.141766301314227 0.325383824558984 0.408249158249158

hsa-miR-30dS 66.8070175438596 52.9824561403509 1.26092715231788 0.23184728554234 0.635030394540654 0.787516199717767 0.213687556171528 0.41722253614486 0.466329966329966

hsa-miR-518c 91.719298245614 97.8662280701754 0.93719049006207 -0.0648687195808691 0.312941835356791 0.51539848074983 0.140764452381621 0.324812091992885 0.428872053872054

hsa-miR-377 156.080043859649 109.407894736842 1.42658849468831 0.355285925981609 0.0371396350901167 0.142451133701203 0.120890991601963 0.29390214753219 0.646464646464646

hsa-miR-373S 23.4385964912281 24.2894736842105 0.964969302997472 -0.0356589885144255 0.518393520333823 0.704926301914208 0.0840998297988671 0.231140615020453 0.45496632996633

hsa-miR-513b 51.280701754386 30.7368421052632 1.66837899543379 0.511852493614909 0.0736845090935589 0.209091750474475 0.347777797245553 0.550701356005343 0.625420875420875

hsa-miR-1827 42.0350877192982 26.4122807017544 1.59149784124875 0.464675611313551 0.361969469111897 0.563862187443262 0.660568776335633 0.808403680243486 0.563973063973064

hsa-miR-199a-3p 118.526315789474 138.199561403509 0.857646106729717 -0.153563727599422 0.455215845549591 0.641913847564211 0.443630318315563 0.629692376161728 0.446969696969697

hsa-miR-576-3p 1 16.8245614035088 0.059436913451512 -2.82283980704889 0.000202919915905782 0.0078027816629123 0.0791745027426346 0.226215169674796 0.258417508417508

hsa-miR-302c 8.73684210526317 31.5175438596491 0.277205677706652 -1.28299552952892 0.00689836115371159 0.0492007080632487 0.0127432025759842 0.0814621023931436 0.314814814814815

hsa-miR-302b 1 1 1 0 0.0112605933327485 0.068094793794037 0.0169470312828971 0.0931547006187272 0.350589225589226

hsa-miR-433 92.8245614035088 60.1578947368421 1.54301545640128 0.433738590441212 0.00849650873733864 0.0568409848087073 0.0290284808953784 0.126523126326826 0.68476430976431

hsa-miR-1233 119.964912280702 100.24451754386 1.19672292530326 0.179586925515251 0.0561303403525581 0.178482553064073 0.0404318159266342 0.155078476198601 0.634259259259259

hsa-miR-545S 24.8421052631579 10.1140350877193 2.45620121422376 0.898615934544551 0.537432133049187 0.71907586173868 0.125519289009883 0.300272732488568 0.542929292929293

hsa-miR-550S 370.144736842105 313.923245614035 1.17909311277061 0.164745594493286 0.0188466223385685 0.0923027567139672 0.0110216098954254 0.0748948766909616 0.664983164983165

hsa-miR-33bS 159.868421052632 154.053179824561 1.03774827130925 0.0370532421430711 0.709566381263311 0.829782968321662 0.428700020035496 0.616613528817722 0.473484848484849

hsa-miR-920 26.8245614035088 44.6666666666667 0.600549882168107 -0.509909573188289 0.0400238523443725 0.148242852245466 0.00609757549845228 0.0559838577610877 0.356060606060606

hsa-miR-520h 26.1228070175439 27.7368421052632 0.94180898165718 -0.0599528045254625 0.518688530377199 0.704926301914208 0.900358550079233 0.948729461194601 0.545454545454545

hsa-miR-148bS 36.6666666666667 32.3508771929825 1.13340563991323 0.125226940842317 0.757604743333469 0.865661138415829 0.953663017189194 0.976288474299258 0.521885521885522

hsa-miR-31 149.412280701754 158.776315789474 0.941023728626281 -0.0607869233211884 0.605462596955126 0.765862641390888 0.152702880674458 0.332784308136509 0.463383838383838

hsa-miR-1299 3.64473684210526 40.9298245614035 0.0890484354907844 -2.41857483828587 0.16203834462606 0.344906058526738 0.338861425274095 0.542907574926669 0.403198653198653

hsa-miR-96S 196.078947368421 136.592105263158 1.43550717657258 0.361518219920427 0.163460692859138 0.344906058526738 0.590072814028896 0.751080882753595 0.598063973063973

hsa-miR-200bS 77.9298245614035 90.5087719298245 0.861019577437487 -0.149638036790278 0.109841868136368 0.270730290290341 0.0826832799330035 0.229439455248174 0.387626262626263

hsa-miR-517b 18.6315789473684 25.3508771929825 0.734948096885813 -0.307955398744649 0.0880718416809613 0.235312691550061 0.0332099260584229 0.134925243804191 0.381313131313131

hsa-miR-941 116.947368421053 141.267543859649 0.827843149430284 -0.188931575596798 0.134747973808773 0.308739345616617 0.0301365423159633 0.12870338384871 0.394781144781145

hsa-miR-1203 204.765350877193 167.684210526316 1.22113674408872 0.199782182378418 0.159872365164977 0.343208584918843 0.84903679090852 0.929846130144737 0.598905723905724

hsa-miR-648 56.9473684210526 39.0526315789473 1.45822102425876 0.377217216239047 0.500911259854205 0.686415488397678 0.514698862599186 0.690801117298753 0.547558922558923

hsa-miR-517c 1 20.3157894736842 0.0492227979274611 -3.01139839029839 0.0726421968489398 0.207583496293494 0.0372794281282035 0.144919578714593 0.37962962962963

hsa-let-7f 518.004385964912 521.265350877193 0.993744136442614 -0.00627551346610827 0.696188711123468 0.823028572191168 0.86537490663627 0.93821425179284 0.527777777777778

hsa-miR-450b-5p 72.7061403508771 57.8421052631579 1.25697603882317 0.228708867233244 0.192400367451806 0.372353290446977 0.562506551973335 0.725625043875917 0.591750841750842

hsa-miR-423-3p 951.71600877193 897.320175438596 1.06062031683033 0.0588539415154772 0.564088671035343 0.739830582224165 0.67791636520847 0.815179424209318 0.540824915824916

hsa-miR-499-5p 19.7543859649123 19.0964912280702 1.03445107946716 0.0338709280250331 0.975861149381462 0.988592264142906 0.911560681134892 0.951934643195463 0.502525252525252

hsa-let-7c 501.850877192983 395.916666666667 1.26756693881621 0.237099266778027 0.0660233561124102 0.194595863360854 0.0700341890917665 0.214324486475867 0.629208754208754

hsa-miR-601 1 31.9649122807018 0.0312843029637761 -3.46463880998535 0.000265494520395756 0.00881237581159759 0.00214521674312039 0.0368643945666792 0.255892255892256

hsa-miR-571 55.0723684210526 66.219298245614 0.831666445886873 -0.184323824931349 0.342596126827853 0.542496252206306 0.156425155754667 0.3358082323788 0.433080808080808

hsa-miR-520d-5p 56.9824561403509 37.9298245614035 1.50231267345051 0.407005703082415 0.263667376737145 0.463431662167325 0.946049856759229 0.971953602837161 0.578703703703704

hsa-miR-208a 70.1403508771929 77.7280701754386 0.902381221081142 -0.102718208427012 0.459961365596865 0.645556767307597 0.167259858771783 0.352922391491562 0.447811447811448

hsa-miR-617 19.4035087719298 29.3245614035088 0.661681124738259 -0.412971523777873 0.0685015501361693 0.199046591809812 0.0799579736559815 0.226215169674796 0.373737373737374

hsa-miR-136 27.140350877193 22.5394736842105 1.20412531620938 0.185753424700728 0.321606869187188 0.522739739218778 0.61857531674241 0.76920821087709 0.569023569023569

hsa-miR-99aS 30.3333333333333 55.1578947368421 0.549936386768448 -0.597952667865807 0.0113605836251475 0.068094793794037 0.0119408751172403 0.0792912367213575 0.322390572390572

hsa-miR-379S 52.4385964912281 52.4561403508772 0.999665551839465 -0.000334504100794429 0.759172169420363 0.865661138415829 0.661127918786699 0.808403680243486 0.521885521885522

hsa-miR-34c-5p 67.438596491228 74.8245614035087 0.901289566236811 -0.103928689835597 0.136303233696328 0.308739345616617 0.0211142503861753 0.104721828064766 0.39520202020202

hsa-miR-523S 151.833333333333 127.142543859649 1.19419769908757 0.177474578389297 0.282312793478868 0.482447407469828 0.372369543236632 0.568769762501262 0.575757575757576

hsa-miR-641 61.6842105263158 66.2828947368421 0.930620347394541 -0.0719038749980897 0.759283121067466 0.865661138415829 0.447383581423375 0.63086933132087 0.478114478114478

hsa-miR-1206 71.0701754385965 51.6140350877193 1.37695445275323 0.319874142041307 0.127847790348787 0.300646623756293 0.194119056569685 0.390611393121036 0.606902356902357

hsa-miR-202S 112.078947368421 72.4561403508771 1.54685230024213 0.4362220920763 0.0283577663281789 0.116536915910564 0.0954828760102692 0.253232872069996 0.654040404040404

hsa-miR-496 96.4210526315789 109.302631578947 0.882147586372938 -0.125395905485106 0.805478659042364 0.894630737134569 0.32873312545124 0.532393151924321 0.482323232323232

hsa-miR-551a 53.2982456140351 29.5701754385965 1.80243251260753 0.589137148481602 0.0335194396684401 0.13051969550259 0.0566626602985136 0.191764218971048 0.648989898989899

hsa-miR-1308 49.0526315789474 69.8684210526315 0.702071563088513 -0.353719938430502 0.297501477324812 0.495644353149253 0.171452159973175 0.35653786519723 0.426767676767677

hsa-miR-221S 127.267543859649 89.2302631578947 1.42628228759616 0.35507126004214 0.044843012851574 0.160578921539039 0.446332170277053 0.630416796970699 0.640993265993266

hsa-miR-21 794.078947368421 1047.21710526316 0.758275378980631 -0.276708662507851 0.330405719457457 0.531186046795749 0.993775315266581 0.998161974943431 0.431397306397306

hsa-miR-200cS 45.8771929824562 34.3070175438596 1.3372538992585 0.290618182303455 0.187715408254137 0.369461826681007 0.36165896765297 0.562352875240527 0.592592592592593

hsa-miR-545 130.114035087719 99.25 1.31097264572009 0.270769339361818 0.0903016128593507 0.237273267562121 0.132192803952453 0.310958420924137 0.619107744107744

hsa-miR-668 51.280701754386 75.6688596491228 0.677698884219679 -0.38905221326362 0.0228090346459181 0.101616321265954 0.00878715132058318 0.0660883942967129 0.34006734006734

hsa-miR-147 32.9122807017544 52.8070175438596 0.623255813953488 -0.472798228176752 0.188151857217296 0.369461826681007 0.200204350460158 0.397658453737 0.407407407407407

hsa-miR-149 28.6842105263158 43.2105263157895 0.663824604141291 -0.409737314789184 0.0915233347641801 0.238624283690294 0.0789752211345679 0.226215169674796 0.382575757575758

hsa-miR-517S 203.710526315789 164.513157894737 1.23826281692394 0.213709443269659 0.0228177372047107 0.101616321265954 0.305017717055858 0.511126776347971 0.65993265993266

hsa-let-7d 2523.52192982456 2434.5350877193 1.03655188317233 0.0358997077647457 0.568091201099658 0.742822282650008 0.341098730918297 0.54512630515276 0.54040404040404

hsa-miR-181aS 86.6666666666667 54.2631578947368 1.59715486582606 0.468223837496899 0.0329328774880861 0.130371895744121 0.0528839201517779 0.182183550796505 0.64983164983165

hsa-miR-516a-5p 203.271929824561 158.429824561403 1.28304080615691 0.249232890394217 0.0126773235256944 0.0729368680178286 0.413157886460003 0.606469872188053 0.675084175084175

hsa-miR-453 21.0657894736842 46.9649122807018 0.448543145311916 -0.801750402975418 0.0944039768003219 0.24247211898416 0.139393976144666 0.322512068130956 0.383417508417508

hsa-miR-138-2S 67.1929824561403 31.1052631578947 2.16018048505358 0.770191776100483 0.0472211610040262 0.163179544675295 0.0933112350933775 0.250864784690295 0.639309764309764

hsa-miR-214 331.69298245614 290.464912280702 1.14193821157853 0.132727004322197 0.814782470509054 0.901492871973059 0.33639267734587 0.541617314457996 0.483164983164983

hsa-let-7f-1S 24.8421052631579 11.6140350877193 2.13897280966768 0.760325718316657 0.527486195534159 0.709824819640201 0.848932327822557 0.929846130144737 0.543350168350168

hsa-miR-370 57.578947368421 54.4561403508772 1.05734536082474 0.0557613903469266 0.76393253217141 0.867465493768325 0.284407296850982 0.492858428077103 0.478535353535354

hsa-miR-20a 4503.74342105263 3801.32565789474 1.18478231711063 0.169559059071618 0.150881288896199 0.330483635323399 0.228005975302022 0.437264792634766 0.601010101010101

hsa-miR-502-3p 615.030701754386 565.981359649123 1.08666246912384 0.0831110439335774 0.838169956425253 0.913324815248521 0.896424966504458 0.948026697264837 0.51473063973064

hsa-miR-190b 1 1 1 0 0.13602238389532 0.308739345616617 0.0443895813376588 0.163013654018721 0.410774410774411

hsa-miR-656 33.4385964912281 40.5 0.825644357808101 -0.191591157732896 0.424401445916809 0.616596713512132 0.400590090795581 0.595912294209581 0.444023569023569

hsa-miR-591 59.2280701754386 25.1754385964912 2.35261324041812 0.855526727522125 0.00349248470709069 0.0331210362881238 0.0951453920421031 0.253232872069996 0.704545454545455

hsa-miR-153 132.157894736842 132.382127192982 0.99830617273725 -0.00169526341010096 0.875901566727578 0.934367184284178 0.998563637085527 0.998681697860201 0.511363636363636

hsa-miR-744 615.030701754386 464.998903508772 1.32264978930812 0.279637140573402 0.0937764121743649 0.241579234944707 0.0529873363266777 0.182183550796505 0.617845117845118

hsa-miR-143 197.30701754386 219.813596491228 0.897610615054622 -0.108018918242387 0.471030949012056 0.65726277592078 0.555430119722123 0.722980683740863 0.449074074074074

hsa-miR-302a 1 1 1 0 0.059662186372483 0.183887381569474 0.0303467047589961 0.12870338384871 0.384680134680135

hsa-miR-137 116.723684210526 66.6491228070175 1.75131613582522 0.560367582881181 0.0069997657441273 0.0495147363703431 0.0710244590320827 0.215298408879636 0.689393939393939

hsa-miR-34b 51.280701754386 37.9298245614035 1.35198889916744 0.301576766912093 0.231902266170103 0.425890350448966 0.678214167534519 0.815179424209318 0.584175084175084

hsa-miR-595 127.828947368421 223.872807017544 0.570989165997297 -0.560385043241163 0.00142551118688079 0.0206069475498116 0.0107961938522847 0.0748948766909616 0.276094276094276

hsa-miR-1915 543.850877192983 469.243421052631 1.15899520972076 0.147553431234977 0.168958875681364 0.350556892200509 0.303092182527883 0.509356862701694 0.596801346801347

hsa-miR-376c 88.7368421052632 68.6228070175438 1.29311006007925 0.257050216104776 0.434820481044208 0.624478606838993 0.549180217924847 0.717008363190837 0.55513468013468

hsa-miR-148a 804.166666666666 604.529605263158 1.330235375845 0.285355900895104 0.000441077541893291 0.010262112993706 0.000635381286352112 0.0252773881570178 0.746632996632997

hsa-miR-1915S 11.1228070175439 13.8662280701754 0.802150877747905 -0.220458561941551 0.814520805438219 0.901492871973059 0.794454927618079 0.893891267971841 0.483585858585859

hsa-miR-1909S 32.7368421052632 54.6688596491228 0.59882065064784 -0.512793140311477 0.409861760370037 0.607722250584458 0.248434689847402 0.456426715911051 0.441919191919192

hsa-miR-211 1 1 1 0 0.0155672282811254 0.0824001758628353 0.00739635758058442 0.0614741901618286 0.366161616161616

hsa-miR-29c 483.480263157895 425.128289473684 1.1372573294439 0.12861951233083 0.482199259880559 0.667958204296826 0.462893875295953 0.644318410290979 0.5496632996633

hsa-miR-371-3p 35.6820175438596 36.8684210526316 0.96782060433024 -0.0327085349856885 0.971208262383496 0.986106375266926 0.21450329705814 0.417869854088431 0.502946127946128

hsa-miR-92a-2S 1 46.9649122807018 0.0212924915950691 -3.8494007756858 0.00114315094371009 0.020552901342121 0.00616276533870606 0.0559838577610877 0.287037037037037

hsa-miR-1293 19.9649122807018 54.9473684210526 0.363346104725415 -1.01239944242442 0.00396979514470819 0.0351142181193603 0.00860277062529273 0.0660883942967129 0.299242424242424

hsa-miR-493 23.2982456140351 30.0789473684211 0.774569845435988 -0.255447441866595 0.202711949336047 0.385646954798991 0.0763449432744901 0.221222831389372 0.410774410774411

hsa-miR-629 59.5438596491228 60.844298245614 0.978626779599928 -0.0216049352859939 0.70815373380145 0.829782968321662 0.320540165302988 0.523913186849391 0.526094276094276

hsa-miR-205 67.438596491228 54.0350877192982 1.24805194805195 0.221583894122562 0.824102264826883 0.906017125434733 0.836890244789313 0.922656211440404 0.484006734006734

hsa-miR-221 102.5 61.2916666666666 1.67233174711081 0.514218908324972 0.0162570690723279 0.0830168675113547 0.0943267966760211 0.252323844541744 0.668771043771044

hsa-miR-515-5p 203.271929824561 121.705592105263 1.67019383668707 0.512939689565575 0.000147038121688884 0.00746434700102983 0.0195696628147486 0.101246842168735 0.766414141414141

hsa-miR-563 8.24561403508773 12.5997807017544 0.654425202332261 -0.423997982313747 0.506350536118257 0.692520622298028 0.303371294818854 0.509356862701694 0.45496632996633

hsa-miR-9 1 7.56140350877194 0.132250580046403 -2.0230568222692 0.12848873562927 0.301320051217555 0.018393372425777 0.097984446934849 0.400252525252525

hsa-miR-511 36.6666666666667 28.0263157894737 1.30829420970266 0.268724158707166 0.42694499812773 0.619249635939884 0.813267110222168 0.907955389549458 0.555976430976431

hsa-miR-128 538.96052631579 451.292763157895 1.1942591823198 0.177526062033589 0.0429116880579083 0.15494889871956 0.0288189309376203 0.126247397965311 0.642255892255892

hsa-miR-185 20902.4890350877 23622.6414473684 0.884849777771836 -0.122337391012812 0.770898810147025 0.87079276591215 0.88930084811816 0.948026697264837 0.47979797979798

hsa-miR-215 350.850877192982 377.464912280702 0.929492691315562 -0.073116334879457 0.501091260359835 0.686415488397678 0.349610574970585 0.551579389761636 0.452441077441077

hsa-miR-886-3p 1 9.50877192982457 0.105166051660517 -2.2522147336051 0.0767661487822355 0.213019891958422 0.0167869674334297 0.0930722155816988 0.385521885521886

hsa-miR-216a 186.359649122807 122.523026315789 1.52101735262795 0.419379421908955 0.0114432963890764 0.0681073433363653 0.0198270177595784 0.101246842168735 0.677609427609428

hsa-miR-1197 42.2456140350877 49.5087719298246 0.853295535081502 -0.158649325984048 0.217291252451043 0.406772995369306 0.104631464269766 0.26927056154173 0.413299663299663

hsa-miR-558 99.3333333333333 86.6140350877193 1.14685031395584 0.137019327412982 0.318693278340246 0.52196255474819 0.54270913385814 0.711790246990235 0.570286195286195

hsa-miR-888S 42.2105263157895 43.7894736842105 0.963942307692307 -0.0367238329546944 1 1 0.689340240954642 0.822822445288874 0.499579124579125

hsa-miR-380S 99.3333333333333 86 1.15503875968992 0.144133901583787 0.274329069877956 0.47539354880457 0.967981932629983 0.983943943297615 0.577020202020202

hsa-miR-19a 2209.25438596491 1981.90570175439 1.11471216012411 0.108596219229964 0.403525223983921 0.605638727474997 0.3826478546031 0.579342278109606 0.558922558922559

hsa-miR-148b 568.565789473684 486.347587719298 1.16905234821857 0.156193461828274 0.0545858872031655 0.175817382454783 0.0314204159777384 0.130994294631827 0.63510101010101

hsa-miR-654-5p 204.149122807017 175.989035087719 1.16001046715929 0.148429028490742 0.127081388912372 0.30046914693528 0.879006970879435 0.944686196598945 0.607323232323232

hsa-miR-23a 3467.72368421053 3749.71929824561 0.924795540250966 -0.0781826034686379 0.260894968074234 0.460308593721902 0.271530424344433 0.483156198369579 0.420875420875421

hsa-let-7dS 56.9824561403509 51.8289473684211 1.0994330202251 0.0947946107624435 0.99519512256078 0.999841688867204 0.342413571720533 0.546216104241811 0.500841750841751

hsa-miR-362-3p 196.69298245614 224.06798245614 0.877827257156839 -0.130305450539057 0.85699762429706 0.9226610590424 0.801546660599488 0.899525056043378 0.486952861952862

hsa-miR-552 6.87719298245615 21.1315789473684 0.325446243254463 -1.12255798226446 0.278656114662855 0.478091902493129 0.136894094338209 0.319296225442903 0.425084175084175

hsa-miR-562 32.7368421052632 39.2105263157895 0.834899328859061 -0.18044412564038 0.519801580024107 0.70507433264588 0.112449045088666 0.281974984886236 0.454545454545455

hsa-miR-337-3p 20.280701754386 52.9824561403509 0.382781456953642 -0.960291061136593 0.0219754513142401 0.100118935102438 0.0207061926314775 0.103892117680029 0.340488215488215

hsa-miR-323-5p 52.9824561403509 65.1951754385965 0.812674492919372 -0.207424627319833 0.141729217073315 0.318521651912164 0.150610280107062 0.33257612491588 0.396885521885522

hsa-miR-548o 133.280701754386 105.736842105263 1.26049444167911 0.231504058020178 0.266488014890232 0.466489161968094 0.857828766502024 0.933551356231081 0.578282828282828

hsa-miR-1245 47.0175438596491 28.6052631578947 1.64366758662987 0.496930078275528 0.14693668098791 0.326820504362284 0.559667084622084 0.723486268120741 0.601851851851852

hsa-miR-1201 84.9912280701755 70.7807017543859 1.20076837278473 0.182961662536499 0.172413095664871 0.353426369498298 0.960927647520439 0.981397112201348 0.595959595959596

hsa-miR-218-2S 64.9122807017543 65.3684210526315 0.993022007514761 -0.00700245252921821 0.478398988034298 0.664828223306923 0.252105100510641 0.456426715911051 0.449915824915825

hsa-miR-95 8.24561403508773 28.7982456140351 0.286323484617728 -1.25063304223754 0.0320964040641333 0.128237021793273 0.0271885621044753 0.120947057196712 0.351851851851852

hsa-miR-513c 1 17.0438596491228 0.0586721564590839 -2.83579000098295 0.00506651325455754 0.0404851938766959 0.00313493575087858 0.0438185094059494 0.310606060606061

hsa-miR-582-5p 22.3859649122807 35.5175438596491 0.630279081254631 -0.46159257156748 0.0464801551536034 0.163058430477885 0.0345989567064522 0.137598615841789 0.361111111111111

hsa-miR-146aS 100.701754385965 92.5899122807018 1.08761043082826 0.0839830244145031 0.456350175237261 0.64246362353957 0.381893630582841 0.579342278109606 0.552609427609428

hsa-miR-19b-2S 1 1 1 0 0.118010785352972 0.284478513295014 0.0180239680493436 0.0978282039407767 0.40530303030303

hsa-miR-92a 11503.9078947368 11503.9078947368 1 0 0.88486283001069 0.939739648449552 0.453621081769811 0.636544704987557 0.510521885521885

hsa-miR-30aS 42.2105263157895 51.2456140350877 0.823690516946251 -0.193960405853117 0.168947196445857 0.350556892200509 0.120901852867821 0.29390214753219 0.403198653198653

hsa-miR-885-3p 188.149122807017 196.245614035088 0.958743071696764 -0.0421321527186838 0.971251933924551 0.986106375266926 0.837887333565589 0.922656211440404 0.497053872053872

hsa-miR-451 998.964912280702 1305.42214912281 0.765242808965642 -0.267562098174773 0.928200290693944 0.963943262176743 0.250810605666229 0.456426715911051 0.493265993265993

hsa-miR-7 39.9824561403509 50.1140350877193 0.797829511640119 -0.225860348918833 1 1 0.82113186648038 0.91201647461077 0.500420875420875

hsa-miR-518b 112.982456140351 85.1842105263158 1.32633096488518 0.282416457063991 0.4971667981375 0.683208514001056 0.900337562347074 0.948729461194601 0.547979797979798

hsa-miR-498 27.140350877193 48.8771929824561 0.555276381909548 -0.588289303766684 0.0143512814099506 0.0788863430368624 0.00513212047262476 0.0538419241478691 0.328282828282828

hsa-miR-525-5p 110.320175438596 98.1271929824561 1.12425691681938 0.117122299035238 0.424371360234264 0.616596713512132 0.368793839736602 0.565476341809186 0.556397306397306

hsa-miR-1537 30.7368421052632 44.1052631578948 0.69689737470167 -0.361117117653857 0.297488169197241 0.495644353149253 0.246793379460208 0.456426715911051 0.426767676767677

hsa-miR-31S 207.348684210526 154.078947368421 1.34573014517506 0.296936724625451 0.106147299346847 0.264754680162801 0.669796237521641 0.813069097824269 0.613636363636364

hsa-miR-372 33.4385964912281 32.5438596491228 1.02749326145553 0.0271221091726117 0.346750563954445 0.54806911482177 0.0606532732634343 0.196044100473198 0.433922558922559

hsa-miR-1257 1 23.4703947368421 0.0426068675543097 -3.15573982850472 0.00558897908073996 0.042918418992642 0.00208001193027073 0.0366336795066049 0.313552188552189

hsa-miR-30a 304.815789473684 295.219298245614 1.03250631406923 0.0319891611556422 0.635123146006449 0.787516199717767 0.901838142823043 0.949019697215036 0.533670033670034

hsa-miR-200b 64.719298245614 57.1578947368421 1.13228974831185 0.124241908434685 0.274320303011754 0.47539354880457 0.246322308435122 0.456426715911051 0.577020202020202

hsa-miR-605 10 26.7719298245614 0.373525557011796 -0.984768851015806 0.10709978050619 0.266360549212801 0.0601649947771365 0.195933549028939 0.388047138047138

hsa-miR-1228S 965.995614035088 917.378289473684 1.0529959397549 0.0516393772611218 0.819395135226711 0.904324025411098 0.481079870206248 0.661101796159223 0.516414141414141

hsa-miR-1912 182.214912280702 146.714912280702 1.24196586051239 0.216695495623347 0.0165448714368307 0.0834983862572216 0.364910324605673 0.562352875240527 0.668350168350168

hsa-miR-219-5p 56.9824561403509 53.7225877192982 1.06067966118992 0.058909892451431 0.528050445201633 0.709824819640201 0.472479039689194 0.655545677253657 0.544612794612795

hsa-miR-325 58.3333333333333 26.140350877193 2.23154362416107 0.802693554150449 0.0265528620203164 0.111781072797722 0.0438457281299486 0.161704544342503 0.655723905723906

hsa-miR-1270 23.8070175438597 22.6293859649123 1.05203992634945 0.0507310663989911 0.593828601629781 0.763746770799555 0.808695757957813 0.906369401451419 0.537457912457912

hsa-miR-659 88.7368421052632 90.0723684210526 0.985172741216858 -0.0149382813921193 0.819445408889315 0.904324025411098 0.938316069137892 0.968620535485646 0.516414141414141

hsa-miR-190 1 3.90789473684211 0.255892255892256 -1.36299879851637 0.00381528606904921 0.0342978320582237 0.00217854475423017 0.0368643945666792 0.313131313131313

hsa-miR-494 66.8070175438596 69.8684210526315 0.956183301946014 -0.0448056458729477 0.648009482429504 0.795493859653857 0.897139580039387 0.948026697264837 0.532407407407407

hsa-miR-553 1 1 1 0 0.4059556707968 0.607722250584458 0.226236965522606 0.436784119118588 0.447811447811448

hsa-miR-183S 95.719298245614 125.866228070175 0.760484362749368 -0.273799729296464 0.090292789731159 0.237273267562121 0.109438040710202 0.276964894817902 0.380892255892256

hsa-miR-99b 156.28947368421 153.342105263158 1.01922086837137 0.0190384808727862 0.661049785965121 0.803501359560422 0.369558119096618 0.565476341809186 0.531144781144781

hsa-miR-19b-1S 64.0877192982456 71.3004385964912 0.898840463814474 -0.106649719864037 0.318741907708339 0.52196255474819 0.154578897252867 0.334339820373995 0.429713804713805

hsa-miR-644 1 29.5964912280702 0.033787788974511 -3.38765581471142 0.135182092389438 0.308739345616617 0.399109457668612 0.594872991309174 0.397727272727273

hsa-miR-515-3p 1 16.4561403508772 0.0607675906183369 -2.80069868117167 0.0160174876795116 0.0827730051941228 0.0149020647760513 0.0868951479846775 0.345538720538721

hsa-miR-369-3p 6.87719298245615 8.49013157894737 0.810021955314479 -0.210693926356478 0.684925697239011 0.818207890701719 0.60000307251468 0.758129797335532 0.527777777777778

hsa-miR-647 85.4473684210527 59.3530701754386 1.43964529835581 0.364396762659769 0.0763183961197144 0.212722396327707 0.200441978418998 0.397658453737 0.624579124579125

hsa-miR-527 174.410087719298 128.804824561404 1.35406486762578 0.303111081485639 0.147681190920241 0.32763205080763 0.243490756633703 0.455117792864871 0.601851851851852

hsa-miR-135bS 58.3333333333333 51.8870614035088 1.12423659685974 0.117104224747265 0.909125478803396 0.952168327703228 0.250748310138518 0.456426715911051 0.491582491582492

hsa-miR-638 287.973684210526 239.65350877193 1.20162515327318 0.183674934958757 0.520431459902 0.70507433264588 0.732351279332226 0.854079937923934 0.545454545454545

hsa-miR-510 71.0701754385965 97.1875 0.731268686184916 -0.312974327005413 0.0568605437066004 0.178559552788495 0.0731715107902141 0.217748323489499 0.366161616161616

hsa-miR-584 78.4561403508772 39.9210526315789 1.96528235552626 0.675635927378238 0.195307098550939 0.376227736717546 0.237073228928953 0.447689708021195 0.590488215488216

hsa-miR-335S 1 1 1 0 0.0981482559049668 0.248861408630613 0.0696336366772675 0.214324486475867 0.39983164983165

hsa-miR-671-3p 26.8245614035088 53.4835526315789 0.501547860672038 -0.69005624109482 0.121003542345329 0.289760589889944 0.233174477468069 0.443236947257585 0.392255892255892

hsa-miR-454S 9.71929824561404 65.3684210526315 0.148684916800859 -1.90592586441425 0.00140617467462796 0.0206069475498116 0.000966376617164612 0.0288321188652814 0.279461279461279

hsa-miR-520d-3p 1 3.90789473684211 0.255892255892256 -1.36299879851637 0.0100480411831975 0.0637607319198489 0.0235569212015644 0.112942349983056 0.339225589225589

hsa-miR-17 7477.03728070175 7178.66063596491 1.04156438921795 0.0407238033488949 0.97599143574711 0.988592264142906 0.851522813793732 0.930207833296191 0.502525252525253

hsa-miR-1277 57.8245614035088 25.8859649122807 2.23381904439173 0.803712696646368 0.0205603003219349 0.0964322781403795 0.31145947138539 0.516902930395369 0.662457912457912

hsa-miR-566 99.1447368421052 70.5087719298245 1.40613336650908 0.340843644159614 0.00466152440846428 0.0386816881202372 0.00829314185049525 0.0660883942967129 0.698653198653199

hsa-miR-24-2S 186.956140350877 144.131578947368 1.2971212951129 0.260147420709408 0.0157543789309013 0.0824001758628353 0.00857162656097282 0.0660883942967129 0.669612794612795

hsa-miR-105S 6.98245614035089 31.4649122807018 0.221912461667132 -1.5054722916879 0.0108923924641425 0.066737883164994 0.00652538257170079 0.0580557232925545 0.324074074074074

hsa-miR-551bS 46.3684210526316 28.7982456140351 1.61011270179714 0.476304177662642 0.748720409432086 0.86152761778652 0.478852297451015 0.660143023482789 0.522727272727273

hsa-miR-192 4503.74342105263 4831.82894736842 0.932099101626005 -0.0703161377326719 0.0591717851057194 0.183029571850308 0.0277106429261107 0.122637358180685 0.367845117845118

hsa-miR-328 54.4385964912281 44.7543859649123 1.21638573108585 0.195883946306568 0.961332175007074 0.984490894517879 0.990952393257791 0.997890216314438 0.496212121212121

hsa-miR-342-3p 3312.48684210526 3467.72368421053 0.95523379131616 -0.0457991608010123 0.172334564592325 0.353426369498298 0.135810183930137 0.317626527728206 0.404040404040404

hsa-miR-1283 125.649122807018 95.0263157894737 1.32225606941752 0.279339421172756 0.00822181704949862 0.0558695127064355 0.0421302525313926 0.15760925839842 0.685606060606061

hsa-miR-555 39.3684210526316 39.6228070175438 0.993579809608147 -0.00644088845207174 0.622287135048704 0.777183498620885 0.292923958339467 0.498606264392426 0.46506734006734

hsa-miR-1 15.4736842105263 19.0657894736842 0.81159420289855 -0.208754813862111 0.305450954341234 0.504023276475115 0.0801185345722427 0.226215169674796 0.429292929292929

hsa-miR-645 63.8947368421052 56.0438596491228 1.14008452026921 0.131102400245155 0.622166191787601 0.777183498620885 0.233799125796492 0.443447572664556 0.46506734006734

hsa-miR-889 32.7368421052632 22.5701754385965 1.45044694908667 0.37187174968549 0.152482161719935 0.333008656740415 0.179358764094597 0.368762515987097 0.60016835016835

hsa-miR-1321 1 12.1228070175439 0.0824891461649783 -2.49508855593312 0.0626813962913048 0.189140017480406 0.0252037213949898 0.117499875359226 0.377946127946128

hsa-miR-664 216.552631578947 274.078947368421 0.790110417666827 -0.23558257408936 0.41743840988997 0.612675202420622 0.482594190097769 0.662128435698529 0.442760942760943

hsa-miR-548f 46.8771929824562 33.6842105263158 1.39166666666667 0.330502069634709 0.408631044889948 0.607722250584458 0.48731057139162 0.665425669479379 0.558080808080808

hsa-miR-513a-3p 49.0526315789474 48.7894736842105 1.00539374325782 0.00537924911973593 0.932809684052985 0.966583404311971 0.762344570569796 0.877597199200403 0.493686868686869

hsa-miR-1282 1 1 1 0 0.0149231042469168 0.0803650356664428 0.00696373296755635 0.0595019955544666 0.365319865319865

hsa-miR-222 458.607456140351 342.780701754386 1.33790337026896 0.291103739568153 0.0724867017195744 0.207583496293494 0.121238892840625 0.29390214753219 0.626262626262626

hsa-miR-424S 153.228070175439 128.621710526316 1.19130798018806 0.175051846523196 0.434887626559868 0.624478606838993 0.686894692269701 0.822177696849864 0.55513468013468

hsa-miR-624 67.438596491228 77.9671052631579 0.864962169155908 -0.14506950808654 0.0415760786502947 0.151393062764575 0.0717935842392066 0.215881056440541 0.356902356902357

hsa-miR-125a-5p 154.285087719298 171.19298245614 0.901234884197581 -0.10398936256515 0.824129134955117 0.906017125434733 0.608331122176138 0.761959010795366 0.484006734006734

hsa-miR-139-5p 155.53399122807 164.368421052632 0.946252268118262 -0.0552460772787812 0.333523266610584 0.533019590898025 0.146908234811959 0.33084969527719 0.568181818181818

hsa-miR-572 77.0175438596491 67.298245614035 1.1444212721585 0.134899070065888 0.942510763159359 0.974115914498834 0.517046764059491 0.691800554082699 0.505471380471381

hsa-let-7e 159.482456140351 188.184210526316 0.847480538852375 -0.165487402997233 1 1 0.787884528573176 0.889979513296664 0.499579124579125

hsa-miR-141S 107.263157894737 109.197368421053 0.982287022532835 -0.0178717296958871 0.909138704550939 0.952168327703228 0.902833338833772 0.949019697215036 0.491582491582492

hsa-miR-940 164.131578947368 197.69298245614 0.830234725118694 -0.186046816824433 0.0250654495250874 0.107086549208665 0.00965560815345348 0.0700234440036164 0.342592592592593

hsa-miR-1909 111.342105263158 132.026315789474 0.843332668925653 -0.170393773764186 0.188369876870965 0.369461826681007 0.198045057434522 0.394717978212453 0.407407407407407

hsa-miR-454 180.359649122807 152.241228070175 1.18469649390683 0.16948661849655 0.279628357392095 0.478808080217019 0.762219341254539 0.877597199200403 0.576178451178451

hsa-miR-126S 1 38.2631578947368 0.0261348005502063 -3.64448749836708 3.68979211732159e-05 0.0028948096338623 3.87093490949327e-05 0.00835154206723173 0.221380471380471

hsa-miR-660 524.741228070176 450.353070175438 1.1651774192763 0.152873366646824 0.13947378976482 0.315093928185967 0.391228964305274 0.588206613580926 0.603956228956229

hsa-miR-410 89.7543859649123 99.0043859649123 0.906569795773712 -0.0980872569649539 0.580544109187197 0.752271095734112 0.910949875806762 0.951934643195463 0.539141414141414

hsa-miR-1266 120.210526315789 84.5964912280702 1.42098714226462 0.351351800702894 0.00503132827457542 0.0404851938766959 0.00190111347978355 0.034907679426664 0.696969696969697

hsa-miR-548i 1 2.35855263157895 0.423988842398884 -0.858048139193398 0.194832235432698 0.376152615611674 0.0154985320000437 0.0879949547107742 0.416666666666667

hsa-miR-720 3004.33333333333 2349.97368421053 1.2784540327066 0.245651561021083 0.013282983322479 0.0744364584889568 0.00517830788444164 0.0538419241478691 0.673821548821549

hsa-miR-30d 4726.70175438596 4936.95614035088 0.957412142221306 -0.0435213196085313 0.880473272670258 0.937104333677717 0.950191422503536 0.973889783397329 0.489057239057239

hsa-miR-587 45.2631578947369 50.4210526315789 0.897703549060543 -0.107915388723307 0.370097228881745 0.570783139437775 0.251710649403429 0.456426715911051 0.436868686868687

hsa-miR-624S 23.8070175438597 39.0438596491228 0.609750617838689 -0.494705228621039 0.18820747460972 0.369461826681007 0.0143867053543794 0.0850392241152702 0.409090909090909

hsa-miR-548k 15.7894736842105 20.7324561403509 0.761582398984556 -0.272356906349035 0.284123551423637 0.483214554570394 0.119312098731983 0.293351399446443 0.425925925925926

hsa-miR-501-5p 32.8245614035088 59.0877192982456 0.555522565320665 -0.587846049088128 0.112272846404446 0.274480074920785 0.0494888130282381 0.173613193672234 0.388468013468013

hsa-miR-890 20.280701754386 9.32456140350878 2.17497648165569 0.77701785145032 0.0848516858509706 0.231000015423936 0.346010299305469 0.549491867796762 0.618265993265993

hsa-miR-374aS 31.8771929824561 39.0350877192982 0.816629213483146 -0.202570126204169 0.654692221675803 0.801417570647117 0.522589050024553 0.694906548799984 0.531565656565657

hsa-miR-1279 1 9.96491228070176 0.100352112676056 -2.2990701508866 0.00222325602429434 0.0245983326790514 0.0026437731668259 0.0400276533854518 0.305555555555556

hsa-miR-450b-3p 43.3377192982456 18.9912280701754 2.28198614318707 0.825046179198427 0.113303391986107 0.275438950095803 0.281868399079617 0.490728893183364 0.610690235690236

hsa-miR-155 95.0526315789473 77.3333333333333 1.22912885662432 0.20630567181229 0.162652930730931 0.344906058526738 0.229851682992224 0.439438175101173 0.598484848484849

hsa-miR-1286 145.563596491228 114.828947368421 1.26765593369237 0.237169473525661 0.0545991407854945 0.175817382454783 0.548867910039365 0.717008363190837 0.63510101010101

hsa-miR-485-3p 56.9473684210526 67.3508771929824 0.845532690804897 -0.167788446926261 0.555984116601363 0.73270259002867 0.413214698547596 0.606469872188053 0.458333333333333

hsa-miR-520a-3p 1 28.6578947368421 0.0348943985307621 -3.35542896320658 0.000176043616305252 0.0078027816629123 0.033301363766272 0.134925243804191 0.252946127946128

hsa-miR-922 169.236842105263 103.982456140351 1.62755188122153 0.487076972455189 0.0495208674580532 0.166939486782422 0.147198493183122 0.33084969527719 0.638047138047138

hsa-miR-26a-1S 8.24561403508773 5.81578947368421 1.41779788838612 0.349104885078188 0.99497465910494 0.999841688867204 0.315231346822294 0.519954538710003 0.499158249158249

hsa-miR-516b 32.8245614035088 38.5087719298245 0.852391799544419 -0.159708999235182 0.599418258644467 0.765233664512093 0.348554892335539 0.550921011145733 0.462962962962963

hsa-miR-487b 63.7543859649122 37.0526315789474 1.72064393939394 0.542698604129964 0.0159340609441798 0.0827730051941228 0.0619800399187702 0.199339046152631 0.669191919191919

hsa-miR-204 1 7.13157894736842 0.140221402214022 -1.96453266115332 0.00020795362485166 0.0078027816629123 0.00277397133139329 0.0412747803274553 0.287878787878788

hsa-miR-1471 132.421052631579 109.144736842105 1.21326100060277 0.193311776315677 0.0643194996265783 0.190947924637123 0.245613298281221 0.456426715911051 0.630050505050505
[truncated: 491,955 more chars]
